# Enantioselective Synthesis of a New Class of Stable and Functionalized Cyclopentadienes via CuH‐Catalysis

**DOI:** 10.1002/anie.6630116

**Published:** 2026-06-09

**Authors:** Piero Soppelsa, Riccardo Bellin, Besma Boulila, Francesco Vaghi, Manuel Orlandi

**Affiliations:** ^1^ Department of Chemical Sciences University of Padova Padova Italy

**Keywords:** [1,5]‐hydrogen shift, Cu‐H catalysis, cyclopentadienes, enantioselective catalysis, nitrile activation

## Abstract

Cyclopentadienes (CpHs) are fundamental building blocks across chemistry, yet their inherent acidity and tendency to undergo rapid [1,5]‐hydrogen shifts have long limited access to structurally and configurationally stable chiral variants. Overcoming this intrinsic fluxionality remains a major synthetic challenge that limits the potential of CpHs in asymmetric synthesis. Here we present a CuH‐catalyzed transformation that converts simple precursors into highly functionalized and enantioenriched CpHs in high yield and with exceptional enantioselectivity. Computations uncover the origins of the observed selectivity and show the reaction mechanism to involve a rare example of nitrile activation in Cu‐catalysis. The disclosed new class of CpHs is unexpectedly stable, which was found to be attributed to a conjugated push–pull electronic effect that delineates a new general design principle for taming the intrinsic instability of CpHs.

## Graphical abstract

Cyclopentadienes (CpHs) are fundamental building blocks with widespread applications in chemistry. However, their inherent acidity and isomeric instability have long limited access to chiral variants in an asymmetric fashion. Here, we introduce a CuH‐catalyzed asymmetric transformation that provides an unprecedented entry into a new class of functionalized and stable CpHs in high yield and enantioselectivity.

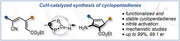

## 1 Introduction

**FIGURE 1:**
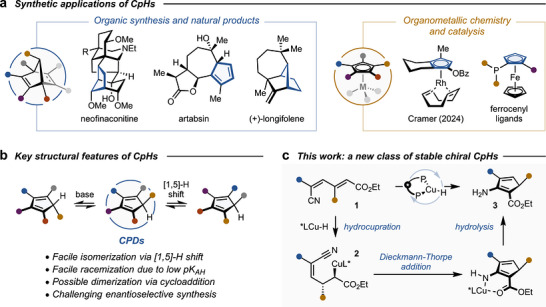
(a) Main fields of investigation and application of chiral CpHs. The shown natural products feature either a CpH core or a substituted 5‐membered ring formed through reactivity of a CpH unit. (b) Structural features of CpHs that make their synthesis challenging and underexplored. (c) Mechanistic rationale for a Cu‐catalyzed enantioselective synthesis of chiral CpHs.

Cyclopentadienes (CpHs) are a distinctive and versatile class of five‐membered conjugated dienes that occupy a central position in both fundamental and applied chemistry (Figure 1a) [1]. As highly reactive archetypal 1,3‐diene, cyclopentadiene and its functionalized derivatives are widely employed in pericyclic reactions—particularly (hetero)Diels–Alder cycloadditions—for the construction of polycyclic frameworks and stereochemically complex molecules, which are crucial in polymer science, pharmaceuticals, agrochemicals, and natural product synthesis [2, 3]. Additionally, CpHs serve as key precursors to cyclopentadienyl ligands, which are constituents of metallocene complexes, organometallic species that play a foundational role in transition metal chemistry and catalysis [4, 5]. Curiously, beyond terrestrial applications, cyclopentadiene also functions as a molecular building block for aromatic hydrocarbons in the interstellar medium, making it an intermediate relevant to astrochemical processes as well [6].

Given the importance covered by CpHs, several synthetic methods have been developed for their formation [7]. However, examples of enantioselective syntheses of chiral CpHs bearing a stereogenic center onto the ring are limited. While a few asymmetric catalytic methods have been reported [8, 9, 10], the only general approach for accessing enantiomerically enriched CpHs relies on Au‐catalyzed cycloisomerization from optically pure enynes or related substrates [11, 12, 13, 14], thus demanding the stereochemical problem to a preceding synthetic step. However, these approaches suffer limitations in terms of accessible scaffolds and functionalities, which significantly hamper their scope. Moreover, in some cases, the CpHs obtained could not be isolated due to their intrinsic instability toward hydrolysis or isomerization.

The challenges encountered in the synthesis of CpHs—especially in enantioselective fashion—can be related to the unique structure of these molecules, which are often highly reactive and undergo dimerization [15] and/or isomerization via [1,5]‐hydrogen shift [3]. Notably, in the case of CpHs that are not penta‐substituted, isomerization via [1,5]‐H shift can lead to racemization via the formation of a methylene ring member, and thus resulting in an achiral isomer (vide infra). Moreover, racemization due to the low p*K*_AH_ associated with the favorable formation of the corresponding aromatic cyclopentadienyl anions is often facile (e.g., p*K*_AH_ of C_5_H_6_ in DMSO is 18, Figure 1b) [16]. In spite of these challenges, devising new methods for the enantioselective synthesis of new classes of stable and synthetically versatile CpHs would enable the downstream formation of a large variety of molecular scaffolds in a stereochemically controlled fashion, thus enabling or facilitating access to a large chemical space.

In this work, we disclose an enantioselective Cu‐catalyzed strategy for the synthesis of a new class of functionalized and stable CpHs (Figure 1c). This relies on an unprecedented cascade transformation that commences by hydrometallation [17, 18, 19, 20, 21, 22] of a specifically designed substrate **1** to give the Cu‐enolate **2**, which, upon Dieckmann–Thorpe addition to the nitrile group, tautomerization, and hydrolysis, delivers CpH products with general structure **3**. Several points are noteworthy about this process. This represents a unique example of enantioselective synthesis of functionalized and stable chiral CpHs, which can be achieved by the catalytic activation of a nitrile. Such activation is rare in Cu‐catalysis, as nitriles are generally poorly reactive under mild conditions [23, 24, 25]. Finally, CpHs with general structure **3** are stereochemically stable for months at room temperature, and are synthetically modular due to the presence of a number of functionalities, including a diene, an ester, an amino group, and other substituents that can be easily introduced in positions β and δ of substrates **1** (i.e., positions 5 and 3 of CpHs **3**). Thus, these CpHs constitute a new platform for the divergent synthesis of diverse structural motifs in a stereochemically controlled fashion. Here, we further elucidate the reaction mechanism via DFT and explore the reasons for the observed high stability of compounds **3**, thus providing new guidelines for the design of new stable CpHs.

## 2 Results and Discussion

### 2.1 Reaction Optimization

**FIGURE 2:**
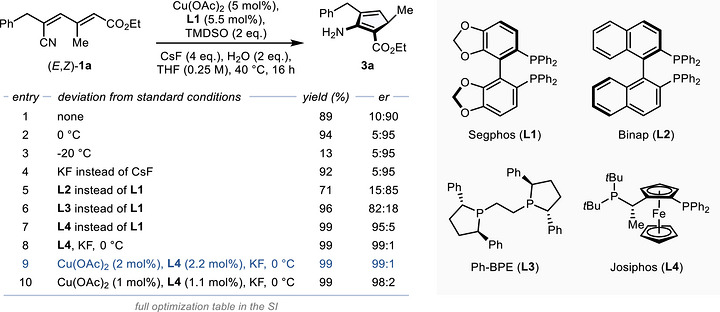
Reaction optimization. Yields were determined by ^1^H NMR analysis with an internal standard, and er's were determined by chiral stationary phase‐HPLC analysis. TMDSO = 1,1,3,3‐tetramethyldisiloxane.

At the onset of our investigation, we submitted substrate **1a** to the reaction conditions previously developed in our group in the context of a CuH‐catalyzed cyclopropanation reaction [26]. Here, employing a fluoride salt as the base is crucial in order to activate the hydride source while avoiding unproductive substrate enolization and post reaction racemization via the formation of an aromatic cyclopentadienyl anion. Thus, by using Cu(OAc)_2_ in combination with (*R*)‐Segphos (**L1**) as the ligand, CsF/H_2_O, and TMDSO as the hydride source in THF at 40°C, we observed the formation of the desired CpH product **3a** in 89% yield and 10:90 er at the first trial (Figure 2, entry 1). We subsequently initiated an optimization campaign aiming at the identification of the ideal catalyst and reaction conditions. While a full table is reported in the Supporting Information, a selection of the most relevant factors affecting reactivity can be inferred from the table in Figure 2. The reaction temperature could be lowered down to 0°C to give **3a** in 94% yield, and 5:95 er (entry 2), and the base could be switched from CsF to the more convenient KF without any significant loss in yield and enantioselectivity (entry 4). Further exploring the role of the chiral ligand allowed us to identify Josiphos (SL‐J002‐2, **L4**) as an excellent candidate, giving **3a** in quantitative yield and 95:5 er already at 40°C with CsF (entry 7). Employing **L4** in the presence of KF at 0°C resulted in excellent enantiocontrol (99%, 99:1 er, entry 8), which, notably, was also maintained with reduced catalyst loading (entries 9 and 10).

It is worth mentioning that the obtainment of diastereomerically pure γ‐olefins in substrates **1** is not necessary, which greatly simplifies the devised synthetic route toward this new class of chiral CpHs. This is because only the depicted (*E*,*Z*)‐**1a** isomer has the correct geometry for the Dieckmann‐Thorpe cyclization to occur, thus giving place to a parallel resolution where (*E*,*Z*)‐**1a** formed the desired CpH **3a**, while (*E*,*E*)‐**1** underwent simple enantioselective conjugate reduction to give an easily separable semi‐hydrogenated byproduct.

### 2.2 Reaction Generality

**FIGURE 3:**
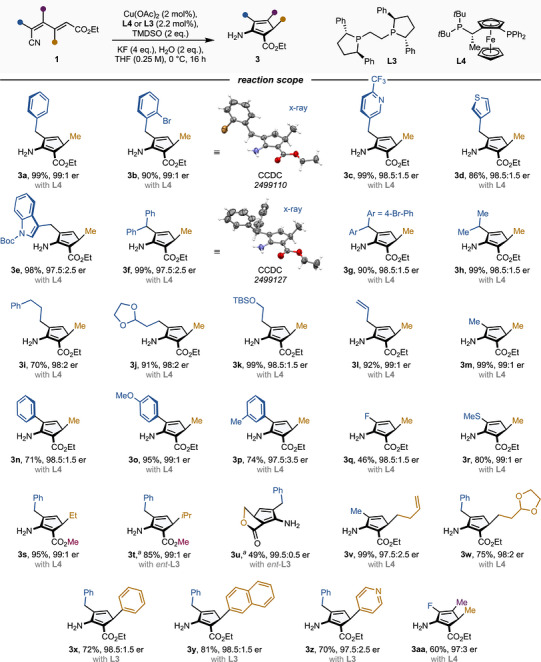
Reaction scope. Isolated yields are reported. Er's were determined by chiral stationary phase‐HPLC analysis. TMDSO = 1,1,3,3‐tetramethyldisiloxane. *^a^* 10 equiv of H_2_O were employed.

With a set of optimized reaction conditions in hand, we began exploring the reaction generality by varying the substituents in positions β and δ of substrate **1**. Products **3a** through **3r** in Figure 3 exemplify the tolerance of this synthetic method toward different functionalities placed in the substrate's δ‐position. CpH **3b**, which features a C(sp^2^)─Br bond, was obtained in 90% yield and 99:1 er. Different classes of heteroaryl groups were included in products **3c‐3e**, which present a pyridine, a thiophene, or an indole group, and were isolated in excellent yields and er. Bulky substituents such as *i*Pr or benzhydryl groups incorporated in **3f**‐**3h** did not significantly affect reactivity (>90%, >97.5:2.5 er). Products **3i‐3l** feature linear alkyl chains decorated with different functional groups, such as a dioxolane (**3j**, 91%, 98:2 er), an allyl group (**3l**, 92%, 99:1 er), or even a silyl ether (**3k**, 99%, 98.5:1.5 er), which nicely tolerated the presence of fluorides during the reaction. CpH **3m**, which presents the ubiquitous, small methyl group, was also obtained in 99% yield and 99:1 er. Aryl groups can also be directly attached to the diene δ‐position, resulting in products such as **3n‐3p**, which were all obtained in high yields and enantioselectivities (>71%, >97.5:2.5 er). Interestingly, heteroatoms can be incorporated into the CpH scaffold. For instance, **3q**, featuring a fluorine atom, was isolated in 46% yield and 98.5:1.5 er, whereas **3r**, featuring a methylthio substituent, was accessed in 80% yield and 99:1 er.

Good generality was observed also by varying the substrate's β‐substituent, which resulted in the formation of a variety of CpHs featuring different substituents at the stereocenter. Here, increasing the steric hindrance from Me to Et, and then to *i*Pr (**3a**, **3s‐3t**) resulted in only slightly diminished yield (99%, 95%, and 85%, respectively) but conserved high enantioselectivity (99:1 er). In some cases, and in particular when changing from linear to branched β‐substituents (e.g., *i*Pr, aryl), a ligand switch from **L4** to **L3** was necessary to maintain high levels of enantioselectivity. Moreover, in the case of **3t**, the use of an increased amount of H_2_O (10 eq) as an additive was found to be beneficial in order to increase reactivity, supposedly due to a more challenging hydrolysis of the product‐catalyst adduct. A similar caveat was also needed to access **3u** from an unsaturated lactone in useful yield and 99.5:0.5 er. Products **3v** and **3w** bearing an olefin and a dioxolane moiety were obtained in high yields and >97.5:2.5 er. (Hetero)aryl substituents were also tolerated, leading to products **3x‐3z** that were obtained in high yields and enantioselectivities. This is notable considering the p*K*_AH_ lowering associated with the installation of an aryl group, which should facilitate racemization via the stabilization of the conjugated cyclopentadienyl anion. To the best of our knowledge, **3x‐3z** are the only existing examples of stereochemically and isomerically stable, chiral CpHs bearing an aromatic substituent onto the ring's stereocenter. Finally, **3aa** was obtained in 60% yield and 97:3 er, which represents an example of fully substituted CpH.

Single crystals of compounds **3b** and **3f** were obtained and analyzed by SC‐XRD analysis [27], which allowed for unambiguous assignment of configuration (*R*) to these products. The configuration of other compounds was assigned accordingly. Note that for products bearing an aryl group at the stereocenter (**3x‐3z**), the assigned configuration is (*S*), by virtue of a change in the substituents CIP‐priority.

### 2.3 Reaction Mechanism

**FIGURE 4:**
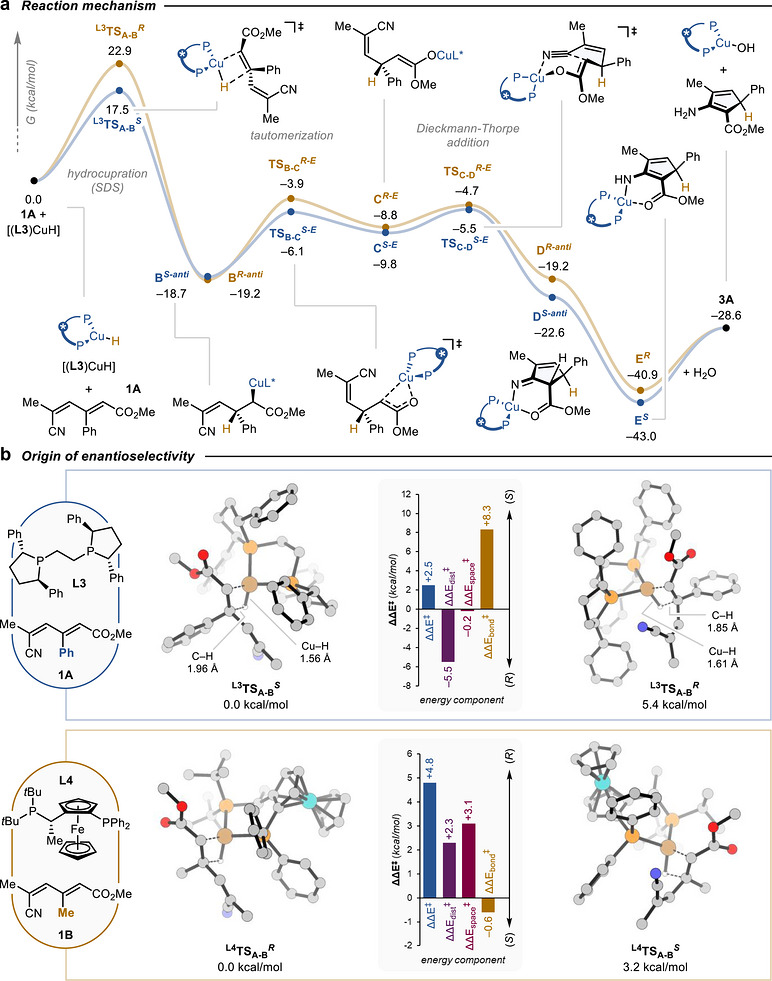
(a) Reaction energy profile in kcal/mol. The Gibbs free energy values are calculated at the ωB97XD/def2TZVPP[SMD = THF]//def2SV level of theory. (b) Analysis of the enantio‐determining hydrocupration step via energy decomposition analysis. This analysis was performed at the ωB97XD/def2TZVPP level in the gas phase. See Supporting Information for further details. SDS = stereo‐determining step.

In order to gain insight into the reaction mechanism, we performed a DFT analysis of the key catalytic steps (Figure 4a, computational details given in Figure 4 caption). Based on the known mechanistic paradigm of CuH‐catalysis and D‐labeling experiments described in the Supporting Information, we began considering the hydrocupration of the model substrate **1A** by [(**L3**)CuH] via TSs **^L3^TS_A‐B_ *^S^*** and **^L3^TS_A‐B_ *^R^***, eventually leading to products (*S*)‐**3A** and (*R*)‐**3A**, respectively. The activation Gibbs free energy for the most stable **^L3^TS_A‐B_ *^S^*** is 17.5 kcal/mol, and the ΔΔG^‡^ associated with the two TSs is 5.4 kcal/mol, which accounts for the high enantioselectivity observed, favoring the formation of (*S*)‐**3A**, in agreement with the experimental outcome. The hydrocupration event leads to the formation of the C‐bound Cu‐enolates **B*^S‐anti^*** and **B*^R‐anti^*** (–18.7 and –19.2 kcal/mol, respectively), which can equilibrate to their corresponding diastereomeric C‐bound enolates **B*^S‐syn^*** and **B*^R‐syn^*** (–21.2 and –23.1 kcal/mol, respectively, not shown) via the formation of the O‐bound enolates **C*^S^ ^‐E^*** (–9.8 kcal/mol), **C*^S‐Z^*** (–10.1 kcal/mol), **C*^R^ ^‐E^*** (–8.8 kcal/mol), and **C*^R‐Z^*** (–10.1 kcal/mol); only **C*^S‐E^*** is shown in Figure 4a. Each one of these eight intermediates can undergo a Dieckmann–Thorpe addition to the nitrile group as detailed in the Supporting Information. However, even though intermediates **B** are energetically more stable by virtue of the soft nature of the Cu(I) metal center, ring closure from **C*^S‐E^*** and **C*^R‐E^*** occurs more favorably via the six‐membered cyclic TSs **TS_C‐D_ *^S^ ^‐E^*** and **TS_C‐D_ *^R^ ^‐E^*** (–5.5 and –4.7 kcal/mol, respectively) to give **D*^S‐anti^*** and **D*^R‐anti^***. Tautomerization to **E*^S^*** and **E*^R^*** is thermodynamically favorable, and subsequent hydrolysis delivers **3A** and restores the catalyst.

After elucidating the general mechanism depicted in Figure 4a, we undertook further analyses to better understand the origins of the observed enantioselectivity with both ligands **L3** and **L4**. We thus computed also the hydrocupration step of the model substrate **1B** by [(**L4**)CuH] via TSs **^L4^TS_A‐B_ *^R^*** and **^L4^TS_A‐B_ *^S^*** (Figure 4b), which were found to have a ΔΔ*G* ^‡^ = 3.4 kcal/mol in favor of the product enantiomer (*R*), in agreement with experiments. An energy decomposition analysis [26, 28, 29, 30, 31] was applied to both systems to dissect the computed ΔΔE^‡^ according to the equation: ΔΔ*E* ^‡^ = ΔΔ*E*_dist_^‡^ + ΔΔ*E*_space_^‡^ + ΔΔ*E*_bond_^‡^, where ΔΔ*E*_dist_^‡^ accounts for the relative distortion energies of the catalyst and substrate at the TS; ΔΔ*E*_space_^‡^ is the relative noncovalent interaction energy between the ligand and the substrate at the TS; and ΔΔ*E*_bond_^‡^ is the relative through‐bond interaction energy at the TS (see Supporting Information for details). Interestingly, the two catalysts were found to impart enantioselectivity by emphasizing different energy contributions (Figure 4b). With **L3**, the only positive contribution to ΔΔ*E* ^‡^ is given by ΔΔ*E*_bond_^‡^ (+8.3 kcal/mol) as opposed to the unfavorable distortion energy (ΔΔE_dist_^‡^ = ‐5.5 kcal/mol), whereas noncovalent interactions were found to be negligible. This suggests that in **^L3^TS_A‐B_ *^S^*** the substrate and catalyst are in less stable yet more reactive conformations, which allow for better orbitals overlapping between the fragments. On the other hand, in **^L3^TS_A‐B_ *^R^***, the substrate and catalyst fragments are less reactive and pair in a later TS in order to interact effectively. Consistently, the forming C─H bond length in **^L3^TS_A‐B_ *^R^*** is shorter than the one in **^L3^TS_A‐B_ *^S^*** (1.85 vs. 1.96 Å), while the breaking Cu─H bond follows the opposite trend (1.61 vs. 1.56 Å). With **L4**, only ΔΔ*E*_dist_^‡^ and ΔΔ*E*_space_^‡^ (+2.3 and +3.1 kcal/mol, respectively) add positively to ΔΔ*E* ^‡^, whereas through‐bond interactions have minor importance. This suggests that the substrate and catalyst in **^L4^TS_A‐B_ *^R^*** need to suffer less distortion in order to minimize steric repulsion and maximize noncovalent contacts between the fragments.

### 2.4 Synthetic Manipulations

**FIGURE 5:**
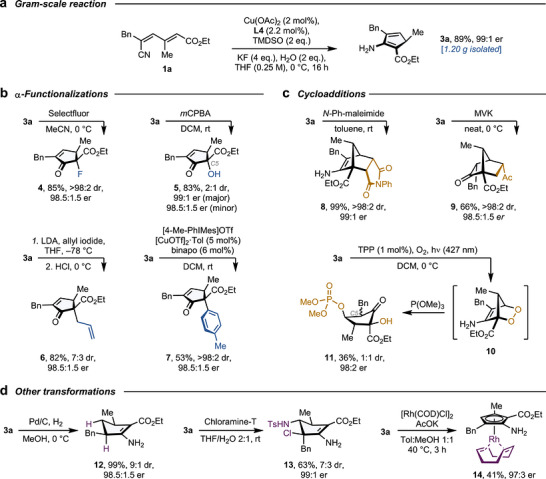
Gram‐scale reaction and manipulation of **3a**. Isolated yields are reported. Er's were determined by chiral stationary phase‐HPLC analysis, and dr's by NMR analysis of the crude reaction mixture.

Having optimized and investigated our catalytic reaction, we set out to evaluate the synthetic versatility of the accessed new class of CpHs. We thus synthesized the benchmark CpH **3a** on a gram scale (1.2 g obtained, 89% yield, 99:1 er) and submitted this to a range of different types of transformations (Figure 5). First, we recognized that the β‐amino enoate moiety could be employed as a nucleophile for the installation of diverse groups to access variously decorated cyclopentenones. For instance, by treatment of **3a** with Selectfluor, the fluorinated product **4** was obtained as a single diastereomer in 85% yield and 98.5:1.5 er (Figure 5). Hydroxylation with *m*CPBA worked similarly to give product **5** in 83% yield and 2:1 dr. The two diastereomers, with opposite configurations at C5, were separable via column chromatography and displayed 99:1 (major diastereomer) and 98.5:1.5 er (minor diastereomer). Formation of a C─C(*sp^3^*) bond was demonstrated by *N*‐allylation of the amino group followed by Claisen rearrangement to give **6** in 82% yield, 7:3 dr, and 98.5:1.5 er; whereas a C─C(*sp^2^*) bond was formed by α‐arylation employing a Cu‐(bis)phosphine oxide catalytic system [32], which allowed us to isolate **7** as a single diastereomer in 53% yield and 98.5:1.5 er.

We then moved to cycloaddition reactions, as these are privileged transformations for CpHs. We found that Diels‐Alder reactions with classical dienophiles such as *N*‐phenyl maleimide or methyl vinyl ketone (MVK) proceeded at low temperature to give products **8** and **9** as single *endo*‐isomers in good yields and conserved optical purity. Furthermore, **3a** reacted with photochemically generated ^1^O_2_ to give the corresponding peroxide adduct **10** via cycloaddition. This could not be isolated due to facile decomposition, but could be trapped with trimethylphosphite to give the highly functionalized cyclopentanone **11** in 36% yield, 98:2 er, and ca. 1:1 dr at C5.

We found that, besides nucleophilic reactivity and pericyclic reactions, **3a** could be selectively hydrogenated with Pd/C to give **12** in quantitative yield, 9:1 dr, and 98.5:1.5 er. Reaction with Chloramine‐T also provided aminochlorination at the less hindered alkene toward **13** (63%, 7:3 dr, 99:1 er) [33]; notably, the depicted major diastereomer could be isolated in 42% yield by column chromatography. Finally, we evaluated the possibility of employing **3a** as a cyclopentadienyl ligand precursor in the stereospecific synthesis of Rh‐complex **14**, which was obtained in 41% yield and 97:3 er under Cramer's conditions [13].

### 2.5 Isomeric and Stereochemical Stability

Most applicative issues of CpHs chemistry in asymmetric synthesis relate to their poor stability due to facile isomerization via [1,5]‐H shift [1]. In this regard, our CpHs were found to be stereochemically stable for months at room temperature. We thus set out to investigate which factors should be ascribed to the stability of this new class of compounds. In 2008, Gleason and coworkers reported that the installation of electron‐donating groups onto the ring inhibits this process [3]. In agreement with computational studies by Houk, Alabugin, and others, this can be ascribed to the importance of aromaticity as a stabilizing factor in the [1,5]‐H shift TS [34, 35, 36, 37, 38]. Such aromaticity would be accompanied by the build‐up of charge separation to give a positively charged migrating hydrogen atom and a partial cyclopentadienyl anion that should be destabilized by the presence of electron‐donating groups on the ring. Accordingly, much of the stability of CpHs **3** should be ascribed to the NH_2_ group, whereas the ester moiety should facilitate isomerization instead.

**FIGURE 6:**
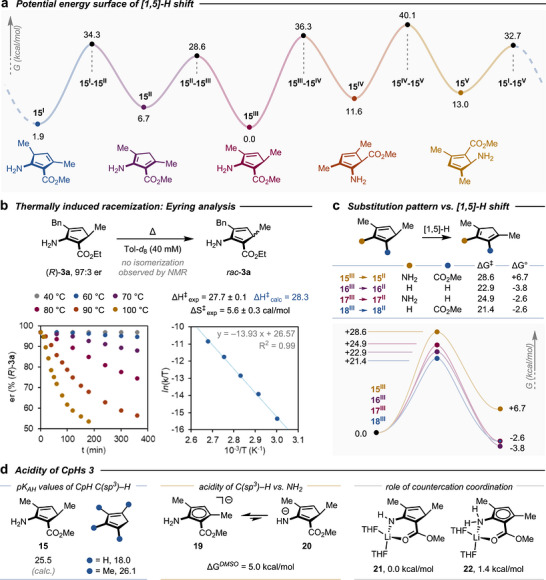
All computations were performed at the ωB97XD/def2TZVPP[SMD = THF]//def2SVP level with the exception of those reported in Figure 6b, where implicit solvation model SMD = Toluene was employed to match the experiment. All the reported energies are in kcal/mol unless otherwise stated. (a) Reaction energy profile for the [1,5]‐H shift of **15** to form each possible isomer. (b) Eyring analysis of the racemization reaction of **3a** via thermal treatment. (c) Computational investigation of the influence of different substituents on the [1,5]‐H shift process. (d) Computational evaluation of the absolute and relative acidity of the N─H and C(sp^3^)─H bonds in the model CpH **15** (SMD solvation model for DMSO was employed). The reported p*K*_AH_ values obtained from Bordwell's tables are in DMSO. For the calculation of the Li‐complexes, SMD solvation for THF was employed.

In order to clarify this picture, we investigated the [1,5]‐H shift process of model CpH **15**. First, the energies of the five isomers **15^I^‐15^V^** and of their connecting TSs were calculated by DFT (Figure 6a). Interestingly, **15^III^** is the most stable isomer, with **15^I^** being energetically close (Δ*G*_rel_ = 1.9 kcal/mol), but also kinetically inaccessible due to the high energies of TSs **15^I‐II^** and **15^III‐IV^** (ΔG^‡^ = 34.3 and 36.3 kcal/mol, respectively). On the contrary, although higher in energy with respect to **15^III^**, **15^II^** (Δ*G*_rel_ = 6.7 kcal/mol) could be accessible via the lowest energy TS **15^II‐III^** (Δ*G* ^‡^ = 28.6 kcal/mol). In principle, these computations suggest that, with sufficient thermal energy available, racemization of **15^III^** could occur via reversible micro‐population of isomer **15^II^**—the only achiral isomer of the set—through [1,5]‐H shift TS **15^II‐III^**. This should occur in the absence of the evident formation of any other isomer. Validation of this picture was accessed via investigation of the thermal stability of a standard solution of (*R*)‐**3a** (97:3 er, 40 mM in toluene‐*d*_8_), which was monitored over time at different temperatures in the range 40°C–100°C (Figure 6b). **3a** was observed to be stable via NMR as no other isomers were observed even after 6 h at 100°C. However, racemization occurred over time with a first order equilibrium kinetics, and Eyring analysis of the profiles obtained allowed us to extrapolate the racemization enthalpy and entropy of activation: Δ*H* ^‡^_exp_ = 27.7 ± 0.1 kcal/mol, and Δ*S* ^‡^_exp_ = 5.6 ± 0.3 cal/mol. The small Δ*S* ^‡^_exp_ value is consistent with a unimolecular process and in agreement with previous reports by McLean and coworkers. Moreover, the measured Δ*H* ^‡^_exp_ value is in good agreement with the calculated barrier for accessing the achiral isomer **3a^II^** via TS **3a^II‐III^** (Δ*H* ^‡^_calc_ = 28.3 kcal/mol). As a control experiment, we heated an analogous toluene solution of the penta‐substituted CpH **3aa** at 80°C for 6 h and observed no racemization (not shown).

Based on these data, we set out to evaluate the influence of the NH_2_ and CO_2_Et moieties on the rate of [1,5]‐H shift for **15**. The [1,5]‐H shift activation barriers of compounds **15^III^‐18^III^** toward **15^II^‐18^II^** were thus calculated by DFT. As expected, the calculated ΔG^‡^ increased with the order **18** > **16** > **17**, thus confirming that the NH_2_ and CO_2_Me groups alone provide opposite contributions to the stabilization of the [1,5]‐H shift TS (*vide infra*). However, surprisingly, when the NH_2_ and CO_2_Me groups were both present as in **15**, these displayed a synergistic stabilizing effect. The origin of this effect can be better understood by considering the relative energies of **15^II^** when compared to **16^II^‐18^II^** in light of the Hammond postulate. In fact, **15** was the only compound in this set of CpHs for which the [1,5]‐H shift was calculated to be endergonic. We attribute such positive Δ*G*° to the break in conjugation between the NH_2_ and CO_2_Me groups when isomerizing from **15^III^** to **15^II^**. The push‐pull system of the β‐amino enoate framework is therefore the main driving force for the stabilization of our CpHs.

In addition to the [1,5]‐H shift pathway, racemization could also occur under basic conditions via deprotonation of the ring C(sp^3^)─H to give the corresponding achiral cyclopentadienyl anion. We thus set out to investigate the acidity of model CpH **15** via DFT. Deprotonation of the C(sp^3^)─H bond by the cyclopentadienyl anion in DMSO to give **19** was calculated to have a Δ*G* ^DMSO^ = 10.2 kcal/mol, which translates into a computed p*K*_AH_ value of 25.5, in line with the experimental p*K*_AH_^DMSO^ values of 18 and 26.1 for cyclopentadiene and tetramethylcyclopentadiene respectively (Figure 6d). Interestingly, deprotonation of the NH_2_ moiety to give **20** was computed to be energetically unfavored with respect to **19** by 5.0 kcal/mol. However, this last observation somehow contrasts experimental evidence for the synthesis of derivative **6**. Such compound was obtained via the initial treatment of **3a** with LDA at −78°C followed by quench with allyl iodide to give the corresponding N‐allyl derivative (Figure 5b). Upon treatment of such an intermediate with HCl_aq_, an aza‐Claisen rearrangement occurs to give **6** with conservation of optical purity. This suggests that, under kinetic conditions, deprotonation of the NH_2_ by LDA occurs preferentially, thus preventing the formation of a cyclopentadienyl anion. Nevertheless, treatment of **3a** with 2 equiv of *t*BuOLi in toluene (40 mM) at room temperature resulted in complete racemization within 10 min (not shown), thus suggesting that, under thermodynamic conditions, deprotonation at the ring can occur. To further investigate these observations, we calculated the relative energies of Li‐complexes **19** and **20** (Figure 6d), and found **19**—featuring deprotonation at the NH_2_ group—to be more stable by 1.4 kcal/mol. Overall, these evidences suggest that deprotonation of CpHs **3** at the ring is accessible, but can be avoided in the presence of hard cations such as Li^+^ under kinetic conditions. In fact, under controlled conditions, the β‐amino enoate framework can act as a chelating acidic moiety, thus preventing deprotonation at the CpH ring and consequent racemization.

## 3 Conclusion

We have developed a novel CuH‐catalyzed synthesis of chiral CpHs, enabling the preparation of highly functionalized products with excellent yields and enantioselectivities of up to >99:1 er. DFT studies provided insight into the reaction mechanism and the origin of the enantioselectivity, revealing a sequential hydrocupration/Dieckmann–Thorpe process of specifically designed substrates **1**. This transformation represents a rare example of nitrile activation in copper catalysis and, to the best of our knowledge, the first reported case involving the addition of a Cu‐enolate to a nitrile group.

The newly accessed class of CpHs exhibits notable features, including high synthetic versatility and exceptional stability toward [1,5]‐H shifts –an otherwise common rearrangement that often undermines the utility of CpHs in asymmetric synthesis. Mechanistic investigations attribute this stability to the thermodynamic stabilization provided by the vinylogous conjugation of the β‐amino enoate framework. This insight offers a new, valuable design principle for the development of more stable and synthetically useful CpHs. Given the broad synthetic relevance of this class of compounds, the structures and mechanistic concepts presented here provide new guidelines for accessing diverse chemical space, with promising implications for organic synthesis, catalysis, and organometallic chemistry.

## Author Contributions

**Piero Soppelsa**: conceptualization, data curation, investigation, methodology, formal analysis, validation. **Riccardo Bellin**: data curation, investigation, methodology, writing – review and editing, formal analysis, validation, visualization. **Francesco Vaghi**: conceptualization. **Manuel Orlandi**: conceptualization, writing – original draft, writing – review and editing, project administration, supervision, funding acquisition, formal analysis, visualization. **Besma Boulila**: investigation, data curation, validation.

## Conflicts of Interest

The authors declare no conflicts of interest.

## Supporting information



The authors have cited additional references within the Supporting Information [39–54].
**Supporting File 1**: anie73053‐sup‐0001‐SuppMat.pdf.

**Supporting File 1**: anie73053‐sup‐0002‐Data.zip.

## Data Availability

The data that supports the findings of this study are available in the supplementary material of this article
