# Supplementary material for: Enantioselective Synthesis of a New Class of Stable and Functionalized Cyclopentadienes via CuH‐Catalysis
Source: Angew Chem Int Ed Engl. 2026 Jun 9;65(32):e6630116. doi: 10.1002/anie.6630116 (PMC13427131; doi:10.1002/anie.6630116)
Supplement: Supplementary file 1 — The authors have cited additional references within the Supporting Information [39–54]. Supporting File 1: anie73053‐sup‐0001‐SuppMat.pdf. [file ANIE-65-e6630116-s001.pdf]

Enantioselective Synthesis of a New Class of Stable and Functionalized Cyclopentadienes via CuH-catalysis

Piero Soppelsa,[1] Riccardo Bellin,[1] Besma Boulila,[1] Francesco Vaghi,[1] Manuel Orlandi[1,*]

[1]Department of Chemical Sciences, University of Padova; Padova, 35131, Italy.

*Corresponding author. Email: manuel.orlandi@unpd.it.

Supporting Information

1	General Information	S2
2	Reaction Optimization	S3
3	General Procedures	S6
4	Synthesis of Starting Materials	S10
5	Synthesis and Characterization of Cyclopentadiens	S32
6	Manipulations of Cyclopentadiens	S68
7	X-Ray Crystallographic Analysis	S83
8	Deuterium Labelling Experiment	S91
8	Computational Details	S93
9	NMR spectra	S148
10	References	S266

1 General Information

All reactions were carried out in oven- or flame-dried glassware under an atmosphere of dry nitrogen unless otherwise noted. Except as otherwise indicated, all reactions were magnetically stirred and monitored by analytical thin layer chromatography (TLC) using Merck pre-coated silica gel plates with F254 indicator. Visualization was accomplished by UV light (254 nm), with potassium permanganate solution as an indicator. Flash column chromatography was performed using silica gel pore size 60 Å, 230-400 mesh particle size, 40-63 µm particle size or Aluminium oxide 90 active neutral. Yields refer to chromatographically and spectrographically pure compounds, unless otherwise noted. Commercial grade reagents and solvents were used without further purification. ^{1}H NMR, ^{13}C NMR, ^{19}F and ^{31}P spectra were recorded on Bruker Avance300 spectrometer, Bruker Avance400 III HD and Bruker NEO 600 MHz equipped with a TCI CryoProbe Prodigy. The proton spectra are reported as follows δ (position of proton, multiplicity, coupling constant J, number of protons). Multiplicities are indicated by s (singlet), d (doublet), t (triplet), q (quartet), p (quintet), h (septet), m (multiplet) and br (broad). HRMS data were obtained using a Xevo G2-S Q-ToF mass spectrometer and an Agilent 6550 iFunnel Q-TOF mass spectrometer with electrospray ionization (ESI) in positive ion mode. Enantiomeric excess was determined on a Shimadzu HPLC SPD-10A with PDA detector and a Shimadzu LC-40D with a SPD-M40 detector using chiral stationary phase columns (0.46 cm x 25 cm) from Phenomenex or Daicel. Unless otherwise noted, all reagents were obtained commercially and used without further purification.

2 Reaction Optimization

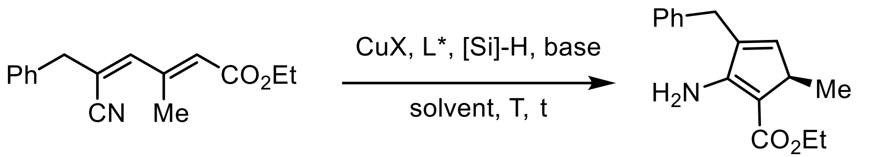

	Cu salt (mol%)	Ligand (mol%) | CAS number		Silane (2 eq)	Base (4 eq)	Solvent (M)	T (°C)	y (%)[a]	*er*[b]
**1**	$Cu(OAc)_2$ (5%)	**(*R*,*R*)-Ph-BPE (5.5%)**	**528565-79-9**	TMDSO	CsF	THF (0.1 M)	40	96	82.5:17.5
**2**	$Cu(OAc)_2$ (5%)	**(*S*)-DTBM-SEGPHOS (5.5%)**	**210169-40-7**	TMDSO	CsF	THF (0.1 M)	40	81	51:49
**3**	$Cu(OAc)_2$ (5%)	**(*R*)-BINAP (5.5%)**	**76189-55-4**	TMDSO	CsF	THF (0.1 M)	40	71	15:85
**4**	$Cu(OAc)_2$ (5%)	**(*R*)-(S_P)-Ph-JOSIPHOS SL-J001-1 (5.5%)**	**155806-35-2**	TMDSO	CsF	THF (0.1 M)	40	80	16.5:83.5
**5**	$Cu(OAc)_2$ (5%)	**(*S*)-1-(1-Hydroxy-3,3-dimethylbutan-2-yl)-3-mesityl-4,5-dihydro-1H-imidazol-3-ium hexafluorophosphate(V)**	**850469-04-4**	TMDSO	CsF	THF (0.1 M)	40	0	-
**6**	$Cu(OAc)_2$ (5%)	**(*R*)-SEGPHOS (5.5%)**	**244261-66-3**	TMDSO	CsF	THF (0.1 M)	40	89	10:90
**7**	$Cu(OAc)_2$ (5%)	**(*S*)-DM-SEGPHOS (5.5%)**	**210169-57-6**	TMDSO	CsF	THF (0.1 M)	40	99	78:21
**8**	$Cu(OAc)_2$ (5%)	**(*R*)-Tol-BINAP (5.5%)**	**99646-28-3**	TMDSO	CsF	THF (0.1 M)	40	97	16:84
**9**	$Cu(OAc)_2$ (5%)	**(*S*)-(R_P)-*t*Bu-JOSIPHOS SL-J002-2 (5.5%)**	**277306-29-3**	TMDSO	CsF	THF (0.1 M)	40	99	94.5:5.5
**10**	$Cu(OAc)_2$ (5%)	**(*S*,*S*)-QUINOX-P (5.5%)**	**1107608-80-9**	TMDSO	CsF	THF (0.1 M)	40	99	74:26
**11**	$Cu(OAc)_2$ (5%)	**(*R*,*R*)-MANDYPHOS (5.5%)**	**174467-31-3**	TMDSO	CsF	THF (0.1 M)	40	16	60:40
**12**	$Cu(OAc)_2$ (5%)	**(*R*)-(R_P)-CF_3-WALPHOS SL-W001-2 (5.5%)**	**387868-06-6**	TMDSO	CsF	THF (0.1 M)	40	59	99:1
**13**	$Cu(OAc)_2$ (5%)	**(S_P)-(*S*)-TANIAPHOS SL-T001-2 (5.5%)**	**850444-36-9**	TMDSO	CsF	THF (0.1 M)	40	18	49:51
**14**	$Cu(OAc)_2$ (5%)	**(*R*,*R*)-Me-DUPHOS (5.5%)**	**147253-67-6**	TMDSO	CsF	THF (0.1 M)	40	27	46:54
**15**	$Cu(OAc)_2$ (5%)	**(*S*,*S*)-Et-FERROCELANE (5.5%)**	**436863-50-2**	TMDSO	CsF	THF (0.1 M)	40	40	49:51
**16**	$Cu(OAc)_2$ (5%)	(*R*)-SEGPHOS (5.5%)	244261-66-3	TMDSO	CsF	THF (0.1 M)	**25**	88	6.5:93.5
**17**	$Cu(OAc)_2$ (5%)	(*R*)-SEGPHOS (5.5%)	244261-66-3	TMDSO	CsF	THF (0.1 M)	**0**	94	5.5:94.5
**18**	$Cu(OAc)_2$ (5%)	(*R*)-SEGPHOS (5.5%)	244261-66-3	TMDSO	CsF	THF (0.1 M)	**-20**	13	5.5:94.5
**19**	$Cu(OAc)_2$ (5%)	(*R*)-SEGPHOS (5.5%)	244261-66-3	TMDSO	CsF	THF (0.1 M)	**-78**	5	4:96
**20**	$Cu(OAc)_2$ (5%)	(*R*)-SEGPHOS (5.5%)	244261-66-3	TMDSO	CsF	**Et_2O (0.1 M)**	40	97	33:67
**21**	$Cu(OAc)_2$ (5%)	(*R*)-SEGPHOS (5.5%)	244261-66-3	TMDSO	CsF	**Toluene (0.1 M)**	40	99	40:60
**22**	$Cu(OAc)_2$ (5%)	(*R*)-SEGPHOS (5.5%)	244261-66-3	TMDSO	CsF	**Dioxane (0.1 M)**	40	64	10.5:89.5
**23**	**CuCl (5%)**	(*R*)-SEGPHOS (5.5%)	244261-66-3	TMDSO	CsF	THF (0.1 M)	40	92	20:80
**24**	**CuTC (5%)**	(*R*)-SEGPHOS (5.5%)	244261-66-3	TMDSO	CsF	THF (0.1 M)	40	96	50:50
**25**	**CuBr (5%)**	(*R*)-SEGPHOS (5.5%)	244261-66-3	TMDSO	CsF	THF (0.1 M)	40	96	26:74
**26**	**$Cu(OTf)_2$ (5%)**	(*R*)-SEGPHOS (5.5%)	244261-66-3	TMDSO	CsF	THF (0.1 M)	40	84	19:81

**27**	Cu(OAc)$_2$ (5%)	(*R*)-SEGPHOS (5.5%)	244261-66-3	TMDSO	**AcONa**	THF (0.1 M)	40	14	6:94
**28**	Cu(OAc)$_2$ (5%)	(*R*)-SEGPHOS (5.5%)	244261-66-3	TMDSO	**KF**	THF (0.1 M)	40	92	5.5:94.5
**29**	Cu(OAc)$_2$ (5%)	(*R*)-SEGPHOS (5.5%)	244261-66-3	TMDSO	**NaF**	THF (0.1 M)	40	17	7:93
**30**	Cu(OAc)$_2$ (5%)	(*R*)-SEGPHOS (5.5%)	244261-66-3	TMDSO	**LiF**	THF (0.1 M)	40	32	6:94
**31**	Cu(OAc)$_2$ (5%)	(*R*)-SEGPHOS (5.5%)	244261-66-3	**PMHS**	KF	THF (0.1 M)	40	6	8:92
**32**	Cu(OAc)$_2$ (5%)	(*R*)-SEGPHOS (5.5%)	244261-66-3	**Ph$_2$SiH$_2$**	KF	THF (0.1 M)	40	85	7:93
**33**	Cu(OAc)$_2$ (5%)	(*R*)-SEGPHOS (5.5%)	244261-66-3	**Me$_2$PhSiH**	KF	THF (0.1 M)	40	85	6:94
**34**	Cu(OAc)$_2$ (5%)	(*R*)-SEGPHOS (5.5%)	244261-66-3	**EtO$_2$MeSiH**	KF	THF (0.1 M)	40	34	6:94
**35**	Cu(OAc)$_2$ (5%)	(*R*)-SEGPHOS (5.5%)	244261-66-3	TMDSO	**KF**	**THF (0.1 M)**	**0**	67	5:95
**36**	Cu(OAc)$_2$ (5%)	(*R*)-SEGPHOS (5.5%)	244261-66-3	TMDSO	KF	**THF (0.2 M)**	0	70	5:95
**37**	Cu(OAc)$_2$ (5%)	(*R*)-SEGPHOS (5.5%)	244261-66-3	TMDSO	KF	**THF (0.5 M)**	0	69	5:95
**38**	Cu(OAc)$_2$ (5%)	**(*S*)-(R_P)-*t*Bu-JOSIPHOS SL-J002-2 (5.5%)**	**277306-29-3**	TMDSO	KF	THF (0.1 M)	0	99	99:1
**39**	Cu(OAc)$_2$ (5%)	**(*R*)-(R_P)-CF$_3$-WALPHOS SL-W001-2 (5.5%)**	**387868-06-6**	TMDSO	KF	THF (0.1 M)	0	28	99.7:0.3
**40**	Cu(OAc)$_2$ (5%)	**(*R*,*R*)-CH$_3$-WALPHOS SL-W009-1 (5.5%)**	**894771-28-9**	TMDSO	KF	THF (0.1 M)	0	37	99.5:0.5
**41**	**Cu(OAc)$_2$ (2.5%)**	**(*S*)-(R_P)-*t*Bu-JOSIPHOS SL-J002-2 (2.75%)**	**277306-29-3**	TMDSO	KF	**THF (0.25 M)**	0	99	99:1
**42**	**Cu(OAc)$_2$ (1.0%)**	**(*S*)-(R_P)-*t*Bu-JOSIPHOS SL-J002-2 (1.1%)**	**277306-29-3**	TMDSO	KF	**THF (0.5 M)**	0	99	98.6:1.4

Table S1. Optimization of the reaction conditions. All the experiments were run for 16 hours. [a)] Determined by ^{1}H NMR analysis of the reaction crude using ethylene carbonate as internal standard. [b)] Determined by chiral stationary phase HPLC analysis.

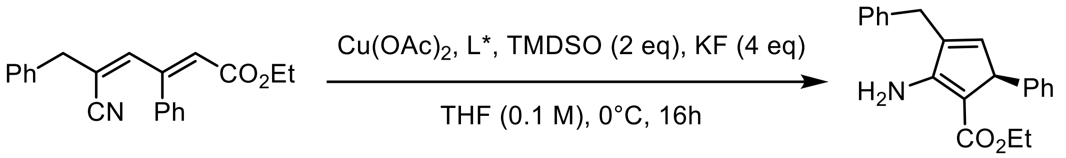

	Cu salt (mol%)	Ligand (mol%) | CAS number		y (%) [a]	*er* [b]
**1**	Cu(OAc)$_2$ (2%)	**(*R*)-BINAP (2.2%)**	**76189-55-4**	67	90:10
**2**	Cu(OAc)$_2$ (2%)	**(*R*)-SEGPHOS (2.2%)**	**244261-66-3**	50	93:7
**3**	Cu(OAc)$_2$ (2%)	**(*S*)-DM-SEGPHOS (2.2%)**	**210169-57-6**	80	90:10
**4**	Cu(OAc)$_2$ (2%)	**(*R*)-Tol-BINAP (2.2%)**	**99646-28-3**	75	91:9
**5**	Cu(OAc)$_2$ (2%)	**(*R*,*R*)-Ph-BPE (2.2%)**	**528565-79-9**	41	97:3
**6**	Cu(OAc)$_2$ (2%)	**(*R*)-(S_P)-Ph-JOSIPHOS SL-J001-1 (2.2%)**	**155806-35-2**	85	93.5:6.5
**7**	Cu(OAc)$_2$ (2%)	**(*S*,*S*)-QUINOX-P (2.2%)**	**1107608-80-9**	16	60:40
**8**	Cu(OAc)$_2$ (2%)	**H-WALPHOS (2.2%)**		5	50:50
**9**	Cu(OAc)$_2$ **(5%)**	**(*R*,*R*)-Ph-BPE (5.5%)**	**528565-79-9**	72[c]	99:1

Table S2. Optimization of the reaction conditions. All the experiments were run for 16 hours. a) Determined by ^{1}H NMR analysis of the reaction crude using ethylene carbonate as internal standard. b) Determined by chiral stationary phase HPLC analysis. c) Yield after 24 hours.

3 General Procedures

Preparation of alkyl cyanomethyl-phosphonates - General Procedure A

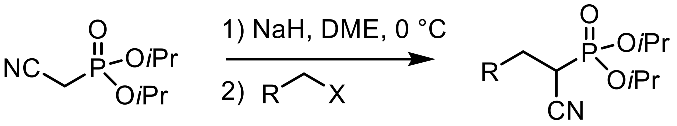

In a two neck round bottom flask, cyanomethylphosphonic acid diisopropyl ester (1 eq) was diluted in dry DME (1.0 M) under nitrogen atmosphere and cooled to 0 °C. 60% wt NaH (1.1 eq) was added portionwise and the mixture was stirred for 30 minutes. The alkyl halide (1.2 eq) was then added dropwise and the mixture was stirred for 4 hours, while warming to room temperature, until disappearance of the starting material (monitored by TLC). The reaction was quenched with NH_4Cl saturated solution and extracted 3 times with EtOAc. The organic layers were collected, dried over anhydrous Na_2SO_4 and concentrated under reduced pressure. The desired product was purified by silica gel flash column chromatography. The dialkylation byproduct is sometimes present and not readily separable. Nevertheless, this does not affect the subsequent olefination step (General Procedure F).

Preparation of alkyl cyanomethyl-phosphonates - General Procedure B

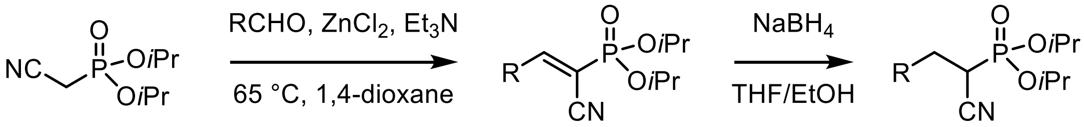

In a two neck round bottom flask, $ZnCl_2$ (1 eq) and the cyanomethylphosphonic acid diisopropyl ester (1 eq) were diluted in dioxane (0.5 M). The flask was equipped with a condenser, the aldehyde was added and the mixture was stirred for 30 minutes at 65 °C. Then, triethyl amine was added and the mixture was stirred for 2 hours at the same temperature. The mixture was cooled to room temperature, diluted with EtOAc and quenched with NH_4Cl saturated solution. The organic phase was extracted 3 times with EtOAc and dried over anhydrous Na_2SO_4 and concentrated under reduced pressure. The desired product was purified by silica gel flash column chromatography (hexane/EtOAc 3:7). The product was then diluted in EtOH/THF (1:1 mixture, 0.5 M) and cooled to 0 °C. $NaBH_4$ (2 eq) was added portionwise and the mixture was stirred for 1 hour, until disappearance of the starting material (TLC). The reaction was quenched with NH_4Cl saturated solution and extracted 3 times with EtOAc. The organic layers were collected, dried over Na_2SO_4 and concentrated under reduced pressure, affording the pure product.

Preparation of aryl and alkyl cyanomethyl-phosphonates - General Procedure C

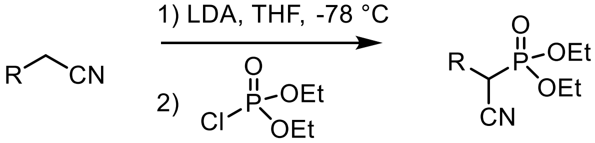

In a two neck round bottom flask, *n*BuLi (1.2 eq) was diluted in dry THF (0.8 M) under nitrogen atmosphere and cooled to -78 °C. A solution of diisopropylamine (1.2 eq) in THF (1.0 M) was added dropwise and the mixture was stirred 10 minutes at the same temperature. The nitrile (1 eq) was diluted in dry THF (1.0 M) and the solution was added dropwise to the main flask. After stirring for 30 minutes at the same temperature, diethyl chlorophosphate was added dropwise and the mixture was gradually warmed to 0 °C. The reaction was quenched with NH_4Cl saturated solution and extracted 3 times with EtOAc. The organic layers were collected, dried over anhydrous Na_2SO_4 and concentrated under reduced pressure. The compound was filtered through a silica plug and used without further purification.

Preparation of 3-alkyl-4-oxocrotonates - General Procedure D

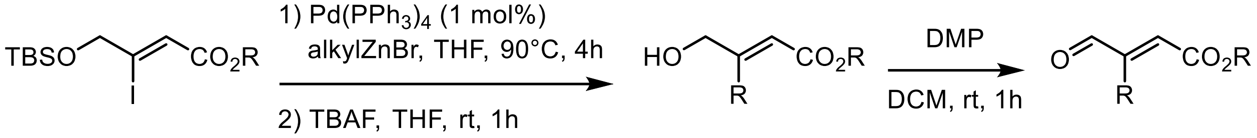

A two neck round bottom flask containing Zn powder (2 eq) and iodine (5 mol%) was backfilled with N_2. DMA was added (1 M with respect to the following halide) and, upon disappearance of the brown color (iodine consumption), the alkyl bromide was added dropwise to the mixture. The reaction was stirred at 80 °C for 5h and the concentration of organozinc reagent was determined by iodometric titration using Knochel's procedure. In a two neck round bottom flask, the starting material ethyl (*Z*)-4-((*tert*-butyldimethylsilyl)oxy)-3-iodobut-2-enoate (0.5 eq) and $Pd(PPh_3)_4$ (0.01 eq) were dissolved in dry THF under nitrogen atmosphere. The solution was stirred for 5 minutes at room temperature and then the organozinc solution was added dropwise. The mixture was stirred 4-16 hours until disappearance of the starting material, monitored by TLC. The reaction mixture was treated with a 1 M solution of TBAF (1.5 eq) in THF at room temperature for 1 hour. The reaction was quenched with NH_4Cl saturated solution and extracted 3 times with EtOAc. The organic layers were collected, dried with anhydrous Na_2SO_4 and concentrated under reduced pressure. The desired product was purified by silica gel flash column chromatography. The pure product was diluted in dry DCM (0.5 M) and Dess-Martin periodinane (1.1 eq) was added portionwise. The mixture was stirred at room temperature for 1 hour and then quenched with $Na_2S_2O_3$ and extracted with EtOAc. The organic layers were collected, dried over anhydrous Na_2SO_4 and concentrated under reduced pressure. The crude product was purified by filtration through a silica plug (hexane/EtOAc 9:1) affording the desired product.

Preparation of 3-aryl-4-oxocrotonates - General Procedure E

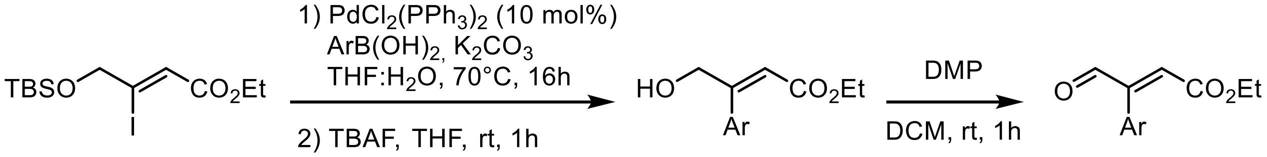

In a two neck round bottom flask, $PdCl_2(PPh_3)_2$ (0.10 eq), K_2CO_3 (2 eq) and $ArB(OH)_2$ (1.5 eq) were added under nitrogen atmosphere. A solution of the starting material ethyl (*Z*)-4-((tert-butyldimethylsilyl)oxy)-

3-iodobut-2-enoate (1 eq) in THF:H_2O (2:1 mixture, 0.15 M) was added to the flask and the mixture was stirred at 70 °C for 16 hours. The reaction was then quenched with NH_4Cl saturated solution and extracted 3 times with EtOAc. The organic layers were collected, dried over anhydrous Na_2SO_4 and concentrated under reduced pressure. The crude product was diluted in dry THF (1 M) and treated with a 1 M solution of TBAF (1.5 eq) in THF at room temperature for 1 hour. The reaction was quenched with NH_4Cl saturated solution and extracted 3 times with EtOAc. The organic layers were collected, dried over anhydrous Na_2SO_4 and concentrated under reduced pressure. The desired product was purified by silica gel flash column chromatography. The pure product was diluted in dry DCM (0.5 M) and Dess-Martin periodinane (1.1 eq) was added portionwise. The mixture was stirred at room temperature for 1 hour. The mixture was quenched with $Na_2S_2O_3$ and extracted with EtOAc. The organic layers were collected, dried with anhydrous Na_2SO_4 and concentrated under reduced pressure. The desired product was purified by silica gel flash column chromatography.

Preparation of 3-alkyl-4-oxocrotonates - General Procedure F

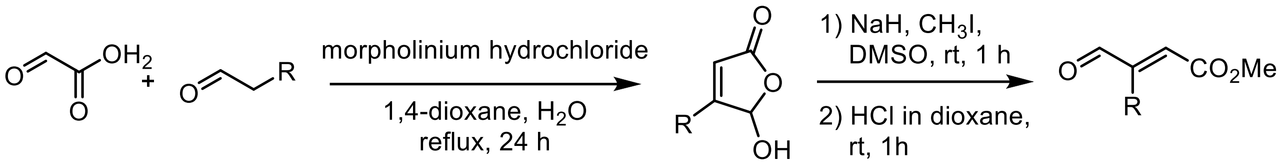

Glyoxylic acid (1.0 eq) and morpholinium hydrochloride (1.1 eq) were dispersed in 1,4-dioxane (2.5 M). Water (3.0 eq) was added dropwise to the medium, which became homogeneous. The aldehyde (1.05 eq) was then added dropwise and the mixture was stirred at room temperature for 1 hour and then refluxed for 24 hours. The solvent was evaporated to dryness, and the residue was extracted 3 times with DCM. The organic layers were collected, dried over anhydrous Na_2SO_4 and concentrated under reduced pressure. The crude oil was purified by recrystallization. A solution of the pure product (1.0 eq) in DMSO (3.0 M) was added dropwise to a suspension of 60% wt NaH (1.2 eq) in DMSO (3.0 M) at 0 °C. After stirring for 1 hour, iodomethane (1.5 eq) was added dropwise. The mixture was stirred at room temperature for 1 hour and then a 4 M solution of HCl in dioxane (0.5 eq) was added. After stirring for 1 h, the reaction was quenched with NH_4Cl saturated solution and the product was extracted 3 times with DCM. The organic layers were collected, dried with anhydrous Na_2SO_4 and concentrated under reduced pressure. The desired product was purified by silica gel flash column chromatography.

Preparation of (*Z,E*)-1-cyan-4-alkoxycarbonyl-butadienes - General Procedure G

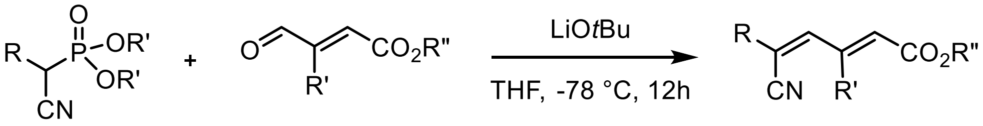

In a two neck round bottom flask, LiO*t*Bu (1.2 eq) was suspended in dry THF (0.3 M) and cooled to -78 °C. The substituted cyanomethyl-phosphonate was added dropwise and the mixture was stirred for 30 minutes at the same temperature. The aldehyde was added dropwise and the mixture was stirred for 12 hours at -78 °C. The reaction was warmed at 0 °C and quenched with NH_4Cl saturated solution. The product was extracted 3 times with EtOAc. The organic layers were collected, dried with anhydrous

Na_2SO_4 and concentrated under reduced pressure. When necessary, the excess of aldehyde was reduced with $NaBH_4$ (1.5 eq) after dilution of the crude product in MeOH (0.5 M), in order to ease the target compound purification by flash chromatography over silica gel.

CuH-catalyzed synthesis of cyclopentadienes - General procedure H

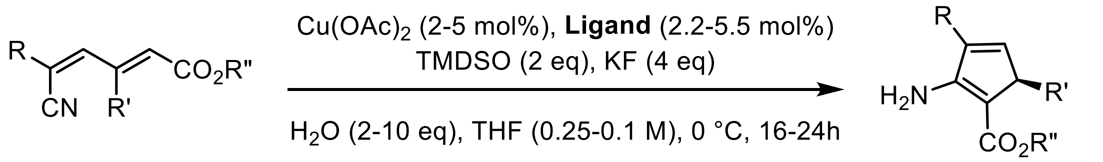

In a nitrogen-filled glove box, an oven dried 4 mL vial was charged with the chiral ligand ((*S*)-(R_P)-*t*Bu-JOSIPHOS SL-J002-2 (2.2 mol%), (*R,R*)-Ph-BPE (5.5 mol%) or (*S,S*)-Ph-BPE (5.5 mol%)) and with $Cu(OAc)_2$ (2 mol% when Josiphos was used or 5 mol% when Ph-BPE was used). The vial was sealed with a ptfe screw cap and removed from the glove box. THF was added to the vial via a syringe (copper concentration: 5 mM) and the mixture was stirred for 15 minutes at room temperature. Then the silane was added and the mixture was stirred for 20 minutes, during which the mixture turned dark yellow, and was then cooled to 0 °C. In another oven dried 4 mL vial, the starting diene (1 eq), KF (4 eq) and H_2O (2-10 eq) were charged. The vial was then sealed with a ptfe screw cap, filled with nitrogen, and placed into a cooling bath at 0 °C. The catalyst solution was then added to the second vial and the mixture was stirred at 0 °C for 16-24 h. The reaction was filtered through a silica plug (ca. 2 ml) and the solvent was evaporated under vacuum. Purification by silica gel flash column chromatography afforded the desired product.

4 Synthesis of the Starting Materials

4.1 Synthesis of cyanomethyl-phosphonates

Diisopropyl (1-cyano-2-phenylethyl)phosphonate (P1)

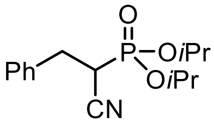

Prepared according to **General Procedure A** using cyanomethylphosphonic acid diisopropyl ester (10.0 g, 48.8 mmol), benzyl chloride (6.2 g, 48.8 mmol) and 60% wt NaH (2.1 g, 53.7 mmol) in dimethoxyethane (50.0 mL). Purification by silica gel flash column chromatography (hexane/EtOAc 3:7) provided the title compound (7.2 g, 42.5 mmol, 87% yield) as a pale yellow oil.

^{1}H NMR (400 MHz, Chloroform-*d*) δ 7.35 – 7.26 (m, 5H), 4.84 (m, 2H), 3.32 – 3.19 (m, 1H), 3.13 – 2.96 (m, 2H), 1.39 – 1.36 (m, 12H).

^{13}C NMR (101 MHz, Chloroform-*d*) δ 136.49 (d, *J* = 13.7 Hz), 128.88, 128.82, 127.57, 116.15 (d, *J* = 9.1 Hz), 73.00 (dd, *J* = 27.6, 7.1 Hz), 33.40 (d, *J* = 141.5 Hz), 33.24 (d, *J* = 4.0 Hz), 23.90 (dd, *J* = 16.2, 5.1 Hz).

^{31}P NMR (162 MHz, Chloroform-*d*) δ 15.23 – 14.75 (m). (major)

HRMS (ESI-TOF) m/z $[M+H]^+$ Calcd for $C_{15}H_{23}NO_3P$ 296.1416; found 296.1421.

Diisopropyl (2-(2-bromophenyl)-1-cyanoethyl)phosphonate (P2)

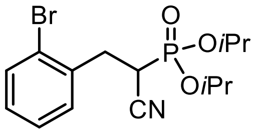

Prepared according to **General Procedure B** using diisopropyl (cyanomethyl)phosphonate (2000 mg, 9.75 mmol), 2-bromobenzaldehyde (1804 mg, 9.75 mmol), $ZnCl_2$ (1328 mg, 9.75 mmol) and Et_3N (1480 mg, 14.63 mmol) in 1,4-dioxane (20.0 mL). Then $NaBH_4$ (650 mg, 17.16 mmol) in a EtOH/THF 1:1 solution (21.5 mL). Purification by silica gel flash column chromatography (hexane/EtOAc 1:1) provided the title compound (2.8 g, 7.46 mmol, 77% yield) as a pale yellow oil.

^{1}H NMR (400 MHz, Chloroform-*d*) δ 7.59 (d, *J* = 8.0 Hz, 1H), 7.41 (d, *J* = 7.3 Hz, 1H), 7.34 (t, *J* = 7.5 Hz, 1H), 7.20 (t, *J* = 7.1 Hz, 1H), 4.90 (dh, *J* = 19.0, 6.4 Hz, 2H), 3.54 – 3.31 (m, 2H), 3.09 (ddd, *J* = 13.5, 11.6, 7.3 Hz, 1H), 1.46 – 1.41 (m, 12H).

^{13}C NMR (101 MHz, Chloroform-*d*) δ 135.60 (d, *J* = 14.1 Hz), 133.15, 131.81, 129.51, 128.00, 124.13, 115.80 (d, *J* = 8.7 Hz), 73.16 (dd, *J* = 34.1, 7.1 Hz), 33.92 (d, *J* = 3.5 Hz), 31.20 (d, *J* = 142.1 Hz), 23.99 (ddd, *J* = 14.6, 4.5, 2.2 Hz).

^{31}P NMR (162 MHz, Chloroform-*d*) δ 14.71 (dp, *J* = 22.0, 7.3 Hz).

HRMS (ESI-TOF) m/z $[M+Na]^+$ Calcd for $C_{15}H_{21}BrNO_3PNa$ 396.0340; found 396.0343.

Diethyl (*E*)-(1-cyano-2-(6-(trifluoromethyl)pyridin-3-yl)vinyl)phosphonate (P3)

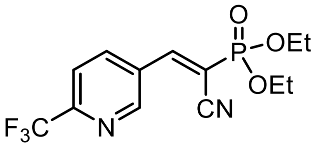

Prepared according to **General Procedure B** using diethyl (cyanomethyl)phosphonate (500 mg, 2.82 mmol), 6-(trifluoromethyl)nicotinaldehyde (495 mg, 2.82 mmol), $ZnCl_2$ (770 mg, 5.64

mmol) and Et_3N (428 mg, 4.23 mmol) in 1,4-dioxane (6.0 mL). Purification by silica gel flash column chromatography (hexane/EtOAc 9:1 to 3:7) provided the title compound (635 mg, 1.90 mmol, 67% yield) as a pale yellow oil.

^{1}H NMR (400 MHz, Chloroform-*d*) δ 9.01 (d, *J* = 2.3 Hz, 1H), 8.63 (d, *J* = 8.2 Hz, 1H), 8.06 (d, *J* = 20.8 Hz, 1H), 7.82 (d, *J* = 8.3 Hz, 1H), 4.25 (h, *J* = 7.1 Hz, 4H), 1.41 (t, *J* = 7.1 Hz, 6H).

^{13}C NMR (101 MHz, Chloroform-*d*) δ 152.98 (d, *J* = 7.3 Hz), 151.76, 150.61 (q, *J* = 35.5 Hz), 137.23 (d, *J* = 1.5 Hz), 130.84 (d, *J* = 18.1 Hz), 120.80 (q, *J* = 2.8 Hz), 114.36 (d, *J* = 9.2 Hz), 106.61 (d, *J* = 195.1 Hz), 64.19 (d, *J* = 6.2 Hz), 30.85, 16.21 (d, *J* = 6.1 Hz).

^{31}P NMR (162 MHz, Chloroform-*d*) δ 8.46.

^{19}F NMR (377 MHz, Chloroform-*d*) δ -68.37.

HRMS (ESI-TOF) m/z $[M+H]^+$ Calcd for $C_{13}H_{15}F_3N_2O_3P$ 335.0772; found 335.0882.

Diethyl (1-cyano-2-(6-(trifluoromethyl)pyridin-3-yl)ethyl)phosphonate (P3')

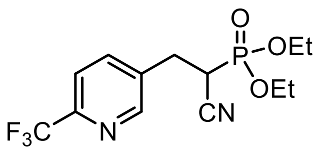

Prepared according to **General Procedure B** using diethyl (*E*)-(1-cyano-2-(6-(trifluoromethyl)pyridin-3-yl)vinyl)phosphonate **P3** (635 mg, 1.90 mmol) and $NaBH_4$ (145 mg, 3.80 mmol) in in a EtOH/THF 1:1 solution (5.0 mL). The crude product (580 mg) was employed in **General Procedure G** without further purification.

Diisopropyl (*E*)-(1-cyano-2-(thiophen-3-yl)vinyl)phosphonate (P4)

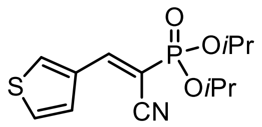

Prepared according to **General Procedure B** using diisopropyl (cyanomethyl)phosphonate (1000 mg, 4.87 mmol), thiophene-3-carbaldehyde (547 mg, 4.87 mmol), $ZnCl_2$ (665 mg, 4.87 mmol) and Et_3N (740 mg, 7.32 mmol) in 1,4-dioxane (10.0 mL). Purification by silica gel flash column chromatography (hexane/EtOAc 9:1 to 3:7) provided the title compound (1420 mg, 4.74 mmol, 97% yield) as a pale yellow oil.

^{1}H NMR (400 MHz, Chloroform-*d*) δ 8.13 – 8.08 (m, 1H), 8.00 (d, *J* = 20.5 Hz, 1H), 7.84 (d, *J* = 5.1 Hz, 1H), 7.45 (dd, *J* = 5.2, 2.9 Hz, 1H), 4.76 (dq, *J* = 12.6, 6.2 Hz, 2H), 1.41 (dd, *J* = 15.0, 6.2 Hz, 12H).

^{13}C NMR (101 MHz, Chloroform-*d*) δ 151.00 (d, *J* = 7.6 Hz), 135.68 (d, *J* = 18.9 Hz), 134.19, 127.33 (d, *J* = 17.5 Hz), 116.11 (d, *J* = 10.2 Hz), 100.33, 98.35, 72.64 (d, *J* = 6.0 Hz), 23.87 (dd, *J* = 10.4, 4.6 Hz).

^{31}P NMR (162 MHz, Chloroform-*d*) δ 9.12 (dt, *J* = 20.9, 7.9 Hz).

HRMS (ESI-TOF) m/z $[M+Na]^+$ Calcd for $C_{13}H_{18}NO_3PSNa$ 322.0643; found 322.0655.

Diisopropyl (1-cyano-2-(thiophen-3-yl)ethyl)phosphonate (P4')

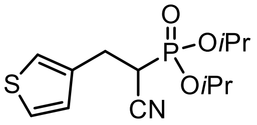

Prepared according to **General Procedure B** using diisopropyl (*E*)-(1-cyano-2-(thiophen-3-yl)vinyl)phosphonate **P4** (1.4 g, 4.74 mmol) and $NaBH_4$ (360 mg, 9.48 mmol) in in a EtOH/THF 1:1 solution (12.0 mL). The crude product (1.3 g) was employed in **General Procedure G** without further purification.

***tert*-butyl 3-(2-cyano-2-(diisopropoxyphosphoryl)ethyl)-1H-indole-1-carboxylate (P5)**

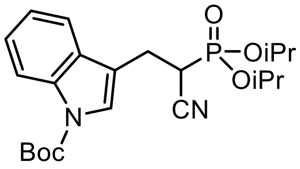

Prepared according to **General Procedure B** using cyanomethylphosphonic acid diisopropyl ester (1.2 g, 5.9 mmol), *tert*-butyl 3-formyl-1H-indole-1-carboxylate (1.4 g, 5.9 mmol), $ZnCl_2$ (800 mg, 5.9 mmol), Et_3N (1.2 mL, 8.9 mmol) in 1,4-dioxane (12.0 mL). Then $NaBH_4$ (336 mg, 8.9 mmol) in a EtOH/THF 1:1 solution (15.0 mL). Purification by silica gel flash column chromatography (hexane/EtOAc 1:1) provided the title compound (1.2 g, 2.8 mmol, 47% yield) as a yellow solid.

^{1}H NMR (400 MHz, Chloroform-*d*) δ 8.19 (d, *J* = 8.3 Hz, 1H), 7.62 (s, 1H), 7.53 (d, *J* = 7.7 Hz, 1H), 7.37 (t, *J* = 7.5 Hz, 1H), 7.28 (d, *J* = 14.7 Hz, 1H), 4.91 (dq, *J* = 13.4, 6.2 Hz, 2H), 3.42 (dt, *J* = 18.8, 10.6 Hz, 1H), 3.29 – 3.13 (m, 2H), 1.69 (s, 9H), 1.44 (t, *J* = 6.3 Hz, 12H).

^{13}C NMR (101 MHz, Chloroform-*d*) δ 149.47, 135.64, 129.27, 124.83, 124.29, 122.75, 118.29, 116.36 (d, *J* = 8.9 Hz), 115.58, 115.34 (d, *J* = 14.9 Hz), 83.93, 73.15 (dd, *J* = 29.1, 7.1 Hz), 31.81 (d, *J* = 141.7 Hz), 28.20, 24.44 – 23.74 (m), 23.45 (d, *J* = 3.7 Hz).

^{31}P NMR (162 MHz, Chloroform-*d*) δ 15.03 (dp, *J* = 17.9, 9.7, 8.7 Hz).

HRMS (ESI-TOF) m/z $[M+H]^+$ Calcd for $C_{22}H_{32}N_2O_5P$ 435.2049; found 435.2047.

Diethyl (*E*)-(1-cyano-2-phenylvinyl)phosphonate (P6)

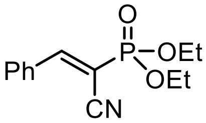

Prepared according to **General Procedure B** using cyanomethylphosphonic acid diethyl ester (3.0 g, 16.9 mol), benzaldehyde (1.8 g, 16.9 mol), $ZnCl_2$ (2.3 g, 16.9 mol) and Et_3N (3.5 mL, 25.4 mol) in 1,4-dioxane (34.0 mL). Purification by silica gel flash column chromatography (hexane/EtOAc 3:7) provided the title compound (3.7 g, 14.0 mmol, 83% yield) as a pale yellow oil.

^{1}H NMR (400 MHz, Chloroform-*d*) δ 8.05 – 7.86 (m, 3H), 7.46 (dd, *J* = 14.8, 7.1 Hz, 3H), 4.19 (dq, *J* = 7.9, 7.4 Hz, 4H), 1.36 (t, *J* = 7.0 Hz, 6H).

^{13}C NMR (101 MHz, Chloroform-*d*) δ 158.9 (d, *J* = 7.0 Hz), 133.1, 132.4 (d, *J* = 17.9 Hz), 130.5, 129.2, 115.4 (d, *J* = 10.2 Hz), 100.1 (d, *J* = 197.6 Hz), 63.6 (d, *J* = 5.9 Hz), 16.2 (d, *J* = 6.3 Hz).

^{31}P NMR (162 MHz, Chloroform-*d*) δ 11.04 (dp, *J* = 21.9, 8.4 Hz).

HRMS (ESI-TOF) m/z $[M+H]^+$ Calcd for $C_{13}H_{16}NO_3P$ 266.0946; found 266.0953.

Diethyl (*E*)-(2-(4-bromophenyl)-1-cyanovinyl)phosphonate (P7)

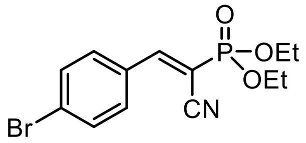

Prepared according to **General Procedure B** using cyanomethylphosphonic acid diethyl ester (2.4 g, 13.4 mmol), benzaldehyde (3.0 g, 13.4 mmol), $ZnCl_2$ (3.8 g, 13.4 mmol) and Et_3N (3.4 mL, 20.1 mmol) in 1,4-dioxane (27.0 mL). Purification by silica gel flash column chromatography (hexane/EtOAc 3:7) provided the title compound (4.1 g, 11.8 mmol, 88% yield) as a pale yellow oil.

^{1}**H NMR** (400 MHz, Chloroform-*d*) δ 7.92 (ddd, *J* = 21.1, 12.6, 5.3 Hz, 1H), 7.85 – 7.74 (m, 2H), 7.66 – 7.53 (m, 2H), 4.21 (dt, *J* = 13.9, 7.0 Hz, 4H), 1.40 (dt, *J* = 13.9, 6.2 Hz, 6H).

^{13}**C NMR** (101 MHz, Chloroform-*d*) δ 157.28, 132.56, 131.69, 131.25 (d, *J* = 18.2 Hz), 127.86, 115.16 (d, *J* = 9.9 Hz), 101.01 (d, *J* = 197.0 Hz), 63.70 (d, *J* = 5.0 Hz), 16.24 (d, *J* = 6.2 Hz).

^{31}**P NMR** (162 MHz, Chloroform-*d*) δ 10.51 (dq, *J* = 18.5, 9.1, 8.5 Hz).

HRMS (ESI-TOF) m/z $[M+H]^+$ Calcd for $C_{13}H_{15}BrNO_3P$ 344.0051; found 344.0047.

Diethyl (*E*)-(1-cyanoprop-1-en-1-yl)phosphonate (P8)

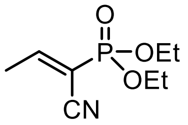

Prepared according to **General Procedure B** at room temperature using cyanomethylphosphonic acid diethyl ester (10.0 g, 56.5 mmol), acetaldehyde (2.5 g, 56.5 mmol), $ZnCl_2$ (7.8 g, 56.5 mmol) and Et_3N (11.8 mL, 84.7 mmol) in 1,4-dioxane (115.0 mL). Purification by silica gel flash column chromatography (hexane/EtOAc 3:7) provided the title compound (7.0 g, 34.5 mmol, 61% yield) as a colorless oil.

^{1}**H NMR** (400 MHz, Chloroform-*d*) δ 7.43 (ddtd, *J* = 16.3, 12.5, 9.2, 7.9, 4.0 Hz, 1H), 4.16 – 4.01 (m, 4H), 2.14 (q, *J* = 5.8, 4.7 Hz, 3H), 1.29 (tt, *J* = 6.7, 2.6 Hz, 6H).

^{13}**C NMR** (101 MHz, Chloroform-*d*) δ 163.34, 113.48 (d, *J* = 13.3 Hz), 108.01 (d, *J* = 200.2 Hz), 63.31 (d, *J* = 5.9 Hz), 18.93 (d, *J* = 15.5 Hz), 16.12 (d, *J* = 6.3 Hz).

^{31}**P NMR** (162 MHz, Chloroform-*d*) δ 8.76 (dp, *J* = 18.0, 9.0 Hz).

HRMS (ESI-TOF) m/z $[M+H]^+$ Calcd for $C_8H_{15}NO_3P$ 204.0790; found 204.0777.

Diisopropyl (1-cyano-4-phenylbutyl)phosphonate (P9)

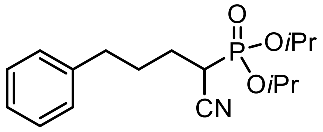

Prepared according to **General Procedure A** using diisopropyl (cyanomethyl)phosphonate (1000 mg, 4.87 mmol), (3-bromopropyl)benzene (970 mg, 4.87 mmol), 60% wt NaH (215 mg, 5.36 mmol) in dimethoxyethane (5.0 mL). Purification by silica gel flash column chromatography (hexane/EtOAc 1:1) provided the title compound (1.05 g, 3.26 mmol, 67% yield) as a pale yellow oil.

^{1}**H NMR** (400 MHz, Chloroform-*d*) δ 7.33 – 7.18 (m, 5H), 4.87 – 4.74 (m, 2H), 2.85 (ddd, *J* = 23.7, 9.8, 4.8 Hz, 1H), 2.70 (td, *J* = 7.1, 2.1 Hz, 2H), 2.08 – 1.79 (m, 4H), 1.40 – 1.35 (m, 12H).

^{13}C NMR (101 MHz, Chloroform-*d*) δ 140.91, 128.51, 128.38, 126.15, 116.47 (d, *J* = 9.2 Hz), 72.81 (dd, *J* = 27.1, 7.0 Hz), 34.96, 30.43 (d, *J* = 199.8 Hz), 29.77 (d, *J* = 43.2 Hz), 26.56 (d, *J* = 4.3 Hz), 23.90 (dd, *J* = 16.2, 5.1 Hz).

^{31}P NMR (162 MHz, Chloroform-*d*) δ 15.81 (dp, *J* = 25.0, 7.8 Hz).

HRMS (ESI-TOF) m/z $[M+Na]^+$ Calcd for $C_{17}H_{26}NO_3PNa$ 346.1548; found 346.1597.

Diisopropyl (1-cyano-3-(1,3-dioxolan-2-yl)propyl)phosphonate (P10)

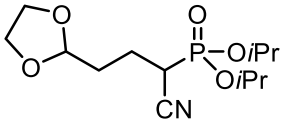

Prepared according to **General Procedure A** using cyanomethylphosphonic acid diisopropyl ester (4.0 g, 19.6 mmol), 2-(3-bromopropyl)-1,3-dioxolane (3.9 g, 19.6 mmol) and 60% wt NaH (868 mg, 36.2 mmol). Purification by silica gel flash column chromatography (hexane/Et_2O 2:8) provided the title compound (3.9 g, 12.8 mmol, 76% yield) as a yellow oil.

^{1}H NMR (300 MHz, Chloroform-*d*) δ 4.93 (t, *J* = 3.4 Hz, 1H), 4.82 (dq, *J* = 12.4, 6.2 Hz, 2H), 4.00 – 3.95 (m, 2H), 3.89 – 3.87 (m, 2H), 2.09 – 1.95 (m, 4H), 1.41 – 1.38 (m, 12H).

^{13}C NMR (101 MHz, Chloroform-*d*) δ 116.53 (d, *J* = 9.2 Hz), 103.41 (d, *J* = 7.2 Hz), 72.78 (dd, *J* = 21.6, 7.1 Hz), 65.05 (d, *J* = 7.4 Hz), 31.34, 29.91, 24.26 – 23.00 (m), 21.58 (d, *J* = 4.2 Hz). (major)

^{31}P NMR (162 MHz, Chloroform-*d*) δ 15.88 (dp, *J* = 24.4, 8.3 Hz). (major)

HRMS (ESI-TOF) m/z $[M+H]^+$ Calcd for $C_{13}H_{25}NO_5P$ 306.1470; found 306.1514.

Diisopropyl (3-((tert-butyldimethylsilyl)oxy)-1-cyanopropyl)phosphonate (P11)

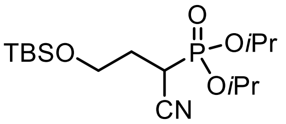

Prepared according to **General Procedure A** using diisopropyl (cyanomethyl)phosphonate (500 mg, 2.44 mmol), (2-bromoethoxy)(tert-butyl)dimethylsilane (583 mg, 2.44 mmol), 60% wt NaH (107 mg, 2.68 mmol) in dimethoxyethane (2.5 mL). Purification by silica gel flash column chromatography (hexane/Et_2O 1:9) provided the title compound (633 mg, 1.74 mmol, 71% yield) as a pale yellow oil.

^{1}H NMR (400 MHz, Chloroform-*d*) δ 4.83 (hept, *J* = 6.6 Hz, 2H), 3.91 – 3.72 (m, 2H), 3.19 (ddd, *J* = 22.8, 11.2, 3.9 Hz, 1H), 2.19 – 1.87 (m, 2H), 1.40 (d, *J* = 6.2 Hz, 12H), 0.91 (s, 9H), 0.09 (d, *J* = 3.8 Hz, 6H).

^{13}C NMR (101 MHz, Chloroform-*d*) δ 116.31 (d, *J* = 9.1 Hz), 72.73 (dd, *J* = 23.6, 7.0 Hz), 59.45 (d, *J* = 13.4 Hz), 30.33 (d, *J* = 4.1 Hz), 27.10 (d, *J* = 146.5 Hz), 25.83, 23.90 (ddd, *J* = 17.1, 5.7, 3.8 Hz), 18.22, -5.48 (d, *J* = 6.0 Hz).

^{31}P NMR (162 MHz, Chloroform-*d*) δ 16.64 (dp, *J* = 23.5, 7.6 Hz).

HRMS (ESI-TOF) m/z $[M+H]^+$ Calcd for $C_{16}H_{35}NO_4PSi$ 364.2073; found 364.2141.

Diisopropyl (1-cyanobut-3-en-1-yl)phosphonate (P12)

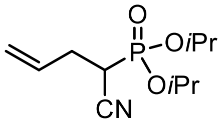

Prepared according to **General Procedure A** using diisopropyl (cyanomethyl)phosphonate (2000 mg, 9.75 mmol), 3-chloroprop-1-ene (746 mg, 9.75 mmol), 60% wt NaH (430 mg, 10.7 mmol) in dimethoxyethane (10.0 mL). Purification by silica gel flash column chromatography (hexane/Et_2O 2:8) provided the title compound (1.55 g, 6.34 mmol, 65% yield) as a pale yellow oil.

^{1}H NMR (400 MHz, Chloroform-*d*) δ 5.87 (ddt, *J* = 17.0, 10.1, 6.9 Hz, 1H), 5.31 – 5.19 (m, 2H), 4.82 (hept, *J* = 6.1 Hz, 2H), 2.90 (ddd, *J* = 23.0, 10.6, 4.4 Hz, 1H), 2.72 – 2.48 (m, 2H), 1.39 (d, *J* = 6.5 Hz, 12H).

^{13}C NMR (101 MHz, Chloroform-*d*) δ 132.56 (d, *J* = 13.4 Hz), 119.19, 116.09 (d, *J* = 9.1 Hz), 72.94 (dd, *J* = 23.2, 7.1 Hz), 31.35 (d, *J* = 4.0 Hz), 30.89 (d, *J* = 143.6 Hz), 23.91 (ddd, *J* = 14.6, 4.5, 2.2 Hz).

^{31}P NMR (162 MHz, Chloroform-*d*) δ 15.51 – 14.95 (m).

HRMS (ESI-TOF) m/z m/z $[M+Na]^+$ Calcd for $C_{11}H_{20}NO_3PNa$ 268.1079; found 268.1105.

Diethyl (1-cyanoethyl)phosphonate (P13)

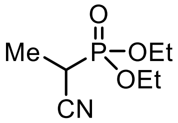

Prepared according to **General Procedure C** using propionitrile (914 µL, 12.8 mmol), diethylchlorophosphate (1.9 mL, 12.8 mmol), *n*BuLi (2.5 M, 10.2 mL, 25.6 mmol) and diisopropylamine (3.6 mL, 25.6 mmol). Purification by silica gel flash column chromatography (hexane/EtOAc 3:7) provided the title compound (2.3 g, 11.8 mmol, 92% yield) as a colorless oil.

^{1}H NMR (400 MHz, Chloroform-*d*) δ 4.18 (hept, *J* = 7.7, 7.1 Hz, 4H), 3.07 – 2.83 (m, 1H), 1.50 (dt, *J* = 16.6, 6.4 Hz, 3H), 1.32 (dt, *J* = 7.0, 6.2 Hz, 6H).

^{13}C NMR (101 MHz, Chloroform-*d*) δ 117.09 (d, *J* = 9.4 Hz), 63.83 (dd, *J* = 25.8, 6.9 Hz), 23.66 (d, *J* = 145.5 Hz), 16.31 (d, *J* = 5.7 Hz), 12.57 (d, *J* = 5.8 Hz).

^{31}P NMR (162 MHz, Chloroform-*d*) δ 18.79 (dtp, *J* = 33.1, 16.8, 8.3 Hz).

HRMS (ESI-TOF) m/z $[M+H]^+$ Calcd for $C_7H_{15}NO_3P$ 192.0790; found 192.0790.

Diethyl (cyano(phenyl)methyl)phosphonate (P14)

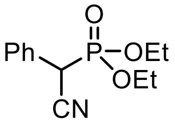

Prepared according to **General Procedure C** using phenylacetonitrile (1.5 g, 12.8 mmol), *n*BuLi (2.5 M, 10.2 mL, 25.6 mmol), diisopropylamine (4.1 g, 25.6 mmol) and diethyl chlorophosphite (2.2 g, 12.8 mmol). The crude product was employed in **General Procedure G** without further purification.

Diethyl (cyano(4-methoxyphenyl)methyl)phosphonate (P15)

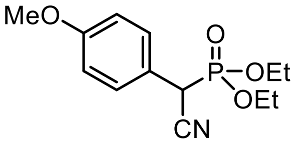

Prepared according to **General Procedure C** using (*p*-methoxyphenyl)acetonitrile (1.0 g, 6.8 mmol), *n*BuLi (2.5 M, 5.4 mL, 13.6 mmol), diisopropylamine (1.4 g, 13.6 mmol) and diethyl chlorophosphite (1.2 g, 6.8 mmol). The crude product was employed in **General Procedure G** without further purification.

Diethyl (cyano(m-tolyl)methyl)phosphonate (P16)

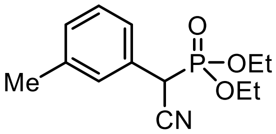

Prepared according to **General Procedure C** using (*m*-methylphenyl)acetonitrile (1.0 g, 7.6 mmol), *n*BuLi (2.5 M, 6.1 mL, 15.2 mmol), diisopropylamine (2.1 g, 15.2 mmol) and diethyl chlorophosphite (1.3 g, 7.6 mmol). The crude product was employed in **General Procedure G** without further purification.

Diisopropyl (cyanofluoromethyl)phosphonate (P17)

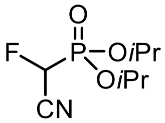

In a two neck round bottom flask, cyanomethylphosphonic acid diisopropyl ester (1.0 g, 4.9 mmol, 1.0 eq) was diluted in dry THF (0.4 M) under nitrogen atmosphere and cooled to -78 °C. *n*BuLi (2.5 M, 2.3 mL, 5.9 mmol, 1.2 eq) was added and the mixture was stirred for 20 minutes. N-fluorobenzenesulfonimide (2.3 g, 5.9 mmol, 1.2 eq) was diluted in dry THF (0.4 M), cooled to -78 °C and then added dropwise and the mixture was stirred for 4 hours, while warming to room temperature, until disappearance of the starting material (monitored by TLC). The reaction was cooled to 0 °C and quenched with 2 N HCl. The mixture was extracted 3 times with Et_2O. The organic layers were collected, dried with anhydrous Na_2SO_4 and concentrated under reduced pressure. Purification by silica gel flash column chromatography (hexane/EtOAc 1:1) provided the title compound (492 mg, 2.2 mmol, 45% yield) as a colorless oil.

^{1}H NMR (400 MHz, Chloroform-*d*) δ 5.30 (dd, *J* = 46.1, 12.7 Hz, 1H), 4.94 – 4.77 (m, 2H), 1.38 – 1.33 (m, 12H).

^{13}C NMR (101 MHz, Chloroform-*d*) δ 112.28 (dd, *J* = 26.6, 1.7 Hz), 74.89 (ddd, *J* = 9.0, 6.9, 2.2 Hz), 74.61 (dd, *J* = 193.5, 172.8 Hz), 24.00 – 23.49 (m).

^{31}P NMR (162 MHz, Chloroform-*d*) δ 3.83 (ddt, *J* = 69.9, 13.5, 7.2 Hz).

^{19}F NMR (377 MHz, Chloroform-*d*) δ -212.39 (dddd, *J* = 69.5, 46.2, 20.0, 7.4 Hz).

HRMS (ESI-TOF) m/z $[M+H]^+$ Calcd for $C_8H_{16}FNO_3P$ 224.0852; found 224.0854.

Diethyl (cyano(methylthio)methyl)phosphonate (P18)

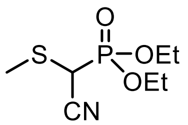

In a two neck round bottom flask, cyanomethylphosphonic acid diethyl ester (1.0 g, 6.0 mmol, 1 eq) was added dropwise to a suspension of 60% wt NaH (360 mg, 9.0 mmol, 1.5 eq) in dry THF (6 mL) under nitrogen atmosphere. After stirring at room temperature for 30 minutes, dimethyldisulfide (848 mg, 9.0 mmol, 1.5 eq) was added dropwise and the reaction mixture stirred overnight. The reaction was quenched with NH_4Cl saturated solution and the mixture extracted 3 times with EtOAc. The organic layers were collected, dried over anhydrous Na_2SO_4 and concentrated under reduced pressure. Purification by silica gel flash column chromatography (hexane/EtOAc 3:7) provided the title compound (1.2 g, 5.5 mmol, 92% yield) as a yellow oil.

^{1}H NMR (400 MHz, Chloroform-*d*) δ 4.27 (p, *J* = 7.6 Hz, 4H), 3.69 (d, *J* = 22.3 Hz, 1H), 2.43 (s, 3H), 1.37 (td, *J* = 6.8, 4.5 Hz, 6H).

^{13}C NMR (75 MHz, Chloroform-*d*) δ 113.23 (d, *J* = 3.8 Hz), 64.44 (dd, *J* = 20.1, 7.1 Hz), 28.78 (d, *J* = 150.9 Hz), 15.92 (dd, *J* = 5.8, 2.8 Hz), 14.77 (d, *J* = 3.1 Hz).

^{31}P NMR (162 MHz, Chloroform-*d*) δ 12.81 (dp, *J* = 25.1, 8.5 Hz).

HRMS (ESI-TOF) m/z $[M+H]^+$ Calcd for $C_7H_{15}NO_3PS$ 224.0510; found 224.0510.

4.2 Synthesis of aldehydes

4-ethyl-5-hydroxyfuran-2(5H)-one (A1)

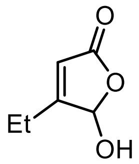

Prepared according to **General Procedure F** using glyoxilic acid monohydrate (2.0 g, 21.7 mmol), butyraldehyde (2.1 mL, 22.8 mmol) and morpholinium hydrochloride (2.9 g, 23.9 mmol) in 1,4-dioxane (9 mL) and H_2O (1.2 mL). Purification by recrystallization from EtOAc/hexane provided the title compound (1.8 g, 14.1 mmol, 65% yield) as a pale yellow solid.

^{1}H NMR (400 MHz, Chloroform-*d*) δ 6.01 (s, 1H), 5.79 (s, 1H), 2.55 – 2.45 (m, 1H), 2.37 – 2.26 (m, 1H), 1.17 (t, *J* = 7.4 Hz, 3H).

^{13}C NMR (101 MHz, Chloroform-*d*) δ 172.60, 172.21, 116.51, 99.54, 21.10, 10.89.

HRMS (ESI-TOF) m/z $[M+H]^+$ Calcd for $C_6H_9O_3$ 129.0552; found 129.0543.

Methyl (*E*)-3-formylpent-2-enoate (A2)

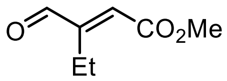

Prepared according to **General Procedure F** using **A1** (1.0 g, 7.8 mmol), 60% wt NaH (374 mg, 9.4 mmol) and iodomethane (729 µL, 11.7 mmol) in DMSO (2.5 mL). Then a solution of HCl 4M in 1,4-dioxane (1 mL, 3.9 mmol) was added. Purification by silica gel flash column chromatography (hexane/EtOAc 9:1) provided the title compound (1.0 g, 7.0 mmol, 90% yield, *dr*=70:30) as a colorless oil.

^{1}H NMR (400 MHz, Chloroform-*d*) δ 9.51 (s, 1H), 6.44 (s, 1H), 3.82 (s, 3H), 2.69 (q, *J* = 7.5 Hz, 2H), 1.04 (t, *J* = 7.6 Hz, 3H). (major)

^{13}C NMR (101 MHz, Chloroform-*d*) δ 194.40, 165.80, 156.35, 134.71, 52.14, 18.46, 13.29. (major)

HRMS (ESI-TOF) m/z $[M+H]^+$ Calcd for $C_7H_{11}O_3$ 143.0708; found 143.0712.

5-hydroxy-4-isopropylfuran-2(5H)-one (A3)

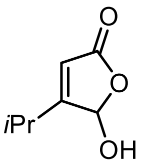

Prepared according to **General Procedure F** using glyoxilic acid monohydrate (2 g, 21.7 mmol), 3-methylbutanal (2 g, 22.8 mmol) and morpholinium hydrochloride (2.9 g, 23.9 mmol) in 1,4-dioxane (9 mL) and H_2O (1.2 mL). Purification by recrystallization from isopropylether/hexane provided the title compound (2.7 g, 17.7 mmol, 80% yield) as a white solid.

^{1}H NMR (400 MHz, Chloroform-*d*) δ 6.12 (s, 1H), 5.79 (s, 1H), 5.37 (br s, 1H), 2.75 (p, *J* = 7.0 Hz, 1H), 1.24 – 1.18 (m, 6H).

^{13}C NMR (101 MHz, Chloroform-*d*) δ 175.86, 172.45, 115.98, 98.95, 27.55, 20.61 (d, *J* = 92.6 Hz).

HRMS (ESI-TOF) m/z $[M+H]^+$ Calcd for $C_7H_{11}O_3$ 143.0708; found 143.0688.

Methyl (*Z*)-3-formyl-4-methylpent-2-enoate (A4)

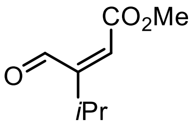

Prepared according to **General Procedure F** using **A3** (1 g, 7.0 mmol), 60% wt NaH (337 mg, 8.4 mmol) and iodomethane (657 µL, 10.5 mmol) in DMSO (2.3 mL). Purification by silica gel flash column chromatography (hexane/EtOAc 9:1) provided the title compound (820 mg, 5.3 mmol, 75% yield, *dr*=80:20) as a yellow oil.

^{1}H NMR (400 MHz, Chloroform-*d*) δ 10.54 (s, 1H), 6.44 (s, 1H), 3.82 (s, 3H), 3.00 (hept, *J* = 6.7 Hz, 1H), 1.10 (d, *J* = 6.9 Hz, 6H). (major)

^{13}C NMR (101 MHz, Chloroform-*d*) δ 193.34, 165.75, 160.12, 126.83, 52.15, 28.25, 21.36. (major)

HRMS (ESI-TOF) m/z $[M+H]^+$ Calcd for $C_8H_{13}O_3$ 157.0865; found 157.0848.

5-oxo-2,5-dihydrofuran-3-carbaldehyde (A5)

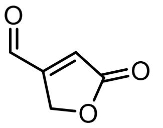

In a two neck round bottom flask, $Ph_3P{=}CHCO_2Et$ (5.8 g, 16.65 mmol, 1.2 eq) was added to a solution of 1,3-dihydroxypropan-2-one (1.25 g, 13.8 mmol, 1.0 eq) in DCM (35 mL, 0.4 M). The reaction was stirred at rt for 16 h, until full consumption of the starting material. The reaction was quenched with NH_4Cl saturated solution and extracted 3 times with EtOAc. The organic layers were collected, dried over anhydrous Na_2SO_4 and concentrated under reduced pressure. The desired intermediate 4-(hydroxymethyl)furan-2(5H)-one was purified by flash column chromatography (hexane/EtOAc 2:8). In a two neck round bottom flask, 4-(hydroxymethyl)furan-2(5H)-one (1.12 g, 9.85 mmol, 1.0 eq) was diluted in dry DCM (25.0 mL, 0.4 M) under nitrogen atmosphere and

Dess-Martin periodinane (4.60 g, 10.84 mmol, 1.1 eq) was added. The mixture was then stirred at room temperature for 1 hour. Purification by silica gel flash column chromatography (hexane/EtOAc 45:55) provided the title compound (773 mg, 6.90 mmol, 50% yield over two steps) as a pale yellow oil.

^{1}H NMR (400 MHz, Chloroform-*d*): δ (ppm) 10.17 (s, 1H), 6.38 (t, *J* = 2.2 Hz, 1H), 5.03 (d, *J* = 2.2 Hz, 2H).

^{13}C NMR (101 MHz, Chloroform-*d*): δ (ppm) 186.1, 171.6, 159.0, 129.1, 69.5.

The NMR data are consistent with those reported in the literature.[39]

Ethyl (*E*)-3-formylhepta-2,6-dienoate (A6)

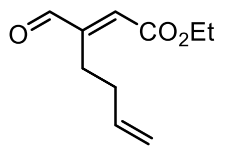

Prepared according to **General Procedure D** using zinc powder (726 mg, 11.1 mmol), iodine (70.5 mg, 0.28 mmol) and 4-bromobut-1-ene (564 µL, 750 mg, 5.55 mmol) in DMA (6.0 mL). The concentration of organozinc reagent was determined by iodometric titration using Knochel's procedure (0.85 M). Then the organozinc was added to a solution of ethyl (*Z*)-4-((*tert*-butyldimethylsilyl)oxy)-3-iodobut-2-enoate (850 mg, 2.30 mmol) and $Pd(PPh_3)_4$ (53.0 mg, 0.046 mmol) in dry THF (2.5 mL). Afterwards, TBAF (1 M, 3.5 mL, 3.5 mmol) and Dess-Martin periodinane (1.4 g, 3.5 mmol) in DCM (6.0 mL) were added. Purification by silica gel flash column chromatography (hexane/EtOAc 9:1) provided the title compound (252 mg, 1.38 mmol, 60% yield) as a pale yellow oil.

^{1}H NMR (400 MHz, Chloroform-*d*) δ 9.51 (s, 1H), 6.49 (s, 1H), 5.90 – 5.65 (m, 1H), 5.07 – 4.86 (m, 2H), 4.28 (q, *J* = 7.1 Hz, 2H), 2.80 (t, *J* = 7.7 Hz, 2H), 2.19 (q, *J* = 7.2 Hz, 2H), 1.35 (t, *J* = 7.1 Hz, 3H).

^{13}C NMR (101 MHz, Chloroform-*d*) δ 194.23, 165.13, 153.52, 137.25, 135.97, 115.29, 61.03, 32.73, 24.15, 14.06.

HRMS (ESI-TOF) m/z $[M+H]^+$ Calcd for $C_{10}H_{15}O_3$ 183.1021; found 183.1019.

Ethyl (*E*)-5-(1,3-dioxolan-2-yl)-3-formylpent-2-enoate (A7)

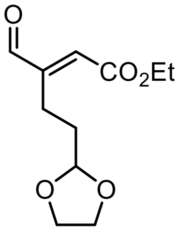

Prepared according to **General Procedure D** using zinc powder (1.44 g, 22.10 mmol), iodine (140 mg, 0.55 mmol) and 2-(2-bromoethyl)-1,3-dioxolane (2.0 g, 11.05 mmol) in DMA (11.0 mL). The concentration of organozinc reagent was determined by iodometric titration using Knochel's procedure (0.85 M). Then the organozinc was added to a solution of ethyl (*Z*)-4-((*tert*-butyldimethylsilyl)oxy)-3-iodobut-2-enoate (1.8 g, 4.86 mmol) and $Pd(PPh_3)_4$ (112 mg, $9.72 \cdot 10^{-2}$ mmol) in THF (5.5 mL). Afterwards, TBAF (1 M, 7.3 mL, 7.29 mmol) and Dess-Martin periodinane (1.9 g, 4.54 mmol) in DCM (10.0 mL) were added. Purification by silica gel flash column chromatography (hexane/EtOAc 8:2) provided the title compound (866 mg, 3.80 mmol, 78% yield) as a pale yellow oil.

^{1}H NMR (300 MHz, Chloroform-*d*) δ 9.49 (s, 1H), 6.46 (s, 1H), 4.87 (t, *J* = 4.5 Hz, 1H), 4.25 (q, *J* = 7.1 Hz, 2H), 3.99 – 3.72 (m, 4H), 2.83 – 2.72 (m, 2H), 1.78 (ddd, *J* = 9.2, 7.0, 4.5 Hz, 2H), 1.31 (t, *J* = 7.1 Hz, 3H).

^{13}C NMR (75 MHz, Chloroform-*d*) δ 194.15, 165.15, 153.83, 135.48, 103.81, 64.83, 61.11, 32.42, 19.30, 14.09.

HRMS (ESI-TOF) m/z $[M+H]^+$ Calcd for $C_{11}H_{17}O_5$ 229.1076; found 229.1088.

Ethyl (*E*)-4-oxo-3-phenylbut-2-enoate (A8)

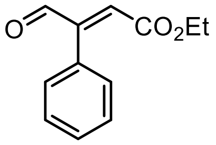

Prepared according to **General Procedure E** using $PdCl_2(PPh_3)_2$ (204 mg, 0.29 mmol), K_2CO_3 (802 mg, 5.80 mmol), phenylboronic acid (531 mg, 4.35 mmol), ethyl (Z)-4-((tert-butyldimethylsilyl)oxy)-3-phenylbut-2-enoate (930 mg, 2.90 mmol) in THF (12.0 mL) and H_2O (6.0 mL). Afterwards, the crude mixture was treated with TBAF (4.4 mL, 4.35 mmol) and Dess-Martin periodinane (1.48 g, 3.48 mmol) in DCM (7.0 mL). Purification by silica gel flash column chromatography (hexane/EtOAc 9:1) provided the title compound (486 mg, 2.38 mmol, 82% yield) as a pale yellow oil.

^{1}H NMR (300 MHz, Chloroform-*d*) δ 9.75 (s, 1H), 7.42 – 7.38 (m, 3H), 7.25 – 7.16 (m, 2H), 6.70 (s, 1H), 4.16 (q, *J* = 7.1 Hz, 2H), 1.12 (t, *J* = 7.1 Hz, 3H).

^{13}C NMR (75 MHz, Chloroform-*d*) δ 192.85, 165.08, 149.47, 136.21, 131.31, 129.07, 128.97, 128.00, 61.27, 13.82.

HRMS (ESI-TOF) m/z $[M+H]^+$ Calcd for $C_{12}H_{13}O_3$ 205.0865; found 205.0869.

Ethyl (*E*)-3-(naphthalen-2-yl)-4-oxobut-2-enoate (A9)

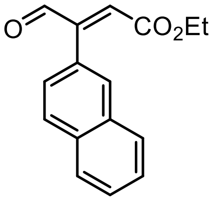

Prepared according to **General Procedure E** using $PdCl_2(PPh_3)_2$ (118 mg, 0.17 mmol), K_2CO_3 (465 mg, 3.37 mmol), 2-naphtylboronic acid (434 mg, 2.53 mmol), ethyl (*Z*)-4-((tert-butyldimethylsilyl)oxy)-3-iodobut-2-enoate (600 mg, 1.68 mmol) in THF (8.0 mL) and H_2O (4.0 mL). Afterwards, the crude mixture was treated with TBAF (2.5 mL, 2.52 mmol) and Dess-Martin periodinane (857g, 2.02 mmol) in DCM (4.0 mL). Purification by silica gel flash column chromatography (hexane/EtOAc 9:1) provided the title compound (343 mg, 1.36 mmol, 80% yield) as a pale yellow oil.

^{1}H NMR (400 MHz, Chloroform-*d*) δ 9.81 (s, 1H), 7.90 – 7.85 (m, 3H), 7.78 (d, *J* = 1.7 Hz, 1H), 7.55 – 7.48 (m, 2H), 7.35 (dd, *J* = 8.5, 1.7 Hz, 1H), 6.78 (s, 1H), 4.13 (q, *J* = 7.1 Hz, 2H), 1.09 (t, *J* = 7.1 Hz, 3H).

^{13}C NMR (101 MHz, Chloroform-*d*) δ 192.87, 165.05, 149.23, 136.31, 133.30, 132.68, 128.72, 128.70, 128.30, 127.68, 127.47, 126.75, 126.45, 126.27, 61.21, 13.78.

HRMS (ESI-TOF) m/z $[M+H]^+$ Calcd for $C_{16}H_{15}O_3$ 255.1021; found 255.1256.

Ethyl (*E*)-4-oxo-3-(pyridin-4-yl)but-2-enoate (A10)

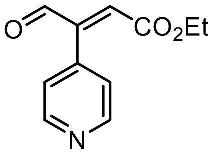

Prepared according to **General Procedure E** using $PdCl_2(PPh_3)_2$ (138 mg, 0.20 mmol), K_2CO_3 (543 mg, 3.93 mmol), 4-pyridylboronic acid (434 mg, 2.53 mmol), ethyl (*Z*)-4-((tert-butyldimethylsilyl)oxy)-3-iodobut-2-enoate (700 mg, 1.96 mmol) in THF (8.0 mL) and H_2O (4.0 mL). Afterwards, the crude mixture was treated with TBAF (2.9 mL, 2.94 mmol) and Dess-Martin periodinane (1.0 g, 2.36 mmol) in DCM (4.0 mL). Purification by silica gel flash

column chromatography (hexane/EtOAc 6:4) provided the title compound (282 mg, 1.36 mmol, 70% yield, *dr*=60:40) as a yellow oil.

^{1}H NMR (400 MHz, Chloroform-*d*) δ 9.75 (s, 1H), 8.66 (d, *J* = 5.9 Hz, 2H), 7.13 (d, *J* = 5.9 Hz, 2H), 6.79 (s, 1H), 4.15 (q, *J* = 7.1 Hz, 2H), 1.15 (t, *J* = 7.1 Hz, 3H). (major)

^{13}C NMR (101 MHz, Chloroform-*d*) δ 191.48, 164.33, 149.64, 147.63, 137.91, 131.90, 123.70, 61.88, 13.91. (major)

HRMS (ESI-TOF) m/z $[M+H]^+$ Calcd for $C_{11}H_{12}NO_3$ 206.0817; found 206.0877.

Ethyl (E)-3-methyl-4-oxopent-2-enoate (A11)

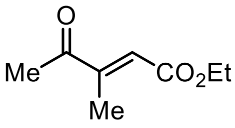

In a two neck round bottom flask, ethyl 3-methy-4-oxocrotonate (0.3 mL, 2.2 mmol) was added and a nitrogen atmosphere was created. Dry Et_2O (9 mL) was added and the solution was cooled to -78°C. 1.6 M methyllithium solution in Et_2O (1.37 mL, 2.2 mmol) was added dropwise and the reaction mixture was stirred for 2 hours at the same temperature. After completion, a saturated solution of NH_4Cl was added and the mixture was warmed to room temperature under vigorous stirring. The product was extracted 3 times with Et_2O. The organic layers were dried with Na_2SO_4 and concentrated under reduced pressure. Afterwards, the crude mixture was treated with Dess-Martin periodinane (1.9 g, 4.4 mmol) in DCM (5.0 mL). Purification by silica gel flash column chromatography (hexane/EtOAc 7:3) provided the title compound (312 mg, 2.0 mmol, 90% yield) as a colorless oil.

^{1}H NMR (400 MHz, Chloroform-*d*) δ 6.54 (s, 1H), 4.21 (q, *J*=7.1 Hz, 2H), 2.35 (s, 3H), 2.16 (s, 3H), 1.29 (t, *J*=7.1 Hz, 3H)

^{13}C NMR (101 MHz, Chloroform-*d*) δ 199.8, 166.1, 150.3, 126.5, 60.7, 26.1, 14.1, 13.0

HRMS could not be determined.

4.3 Synthesis of substrates

Ethyl (2*E*,4*Z*)-5-cyano-3-methyl-6-phenylhexa-2,4-dienoate (1a)

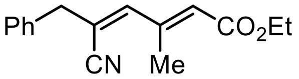

Prepared according to **General Procedure G** using **P1** (1.8 g, 6.1 mmol), ethyl 3-methy-4-oxocrotonate (1.0 mL, 7.3 mmol) and LiO*t*Bu (586 mg, 7.3 mmol) in THF (20.0 mL). Purification by silica gel flash column chromatography (hexane/EtOAc 9:1) provided the title compound (1.4 g, 5.6 mmol, 91% yield, *dr*=90:10) as a white solid.

^{1}H NMR (300 MHz, Chloroform-*d*) δ 7.48 – 7.10 (m, 5H), 6.53 (s, 1H), 6.01 (s, 1H), 4.20 (q, *J* = 7.1 Hz, 2H), 3.63 (s, 2H), 2.51 (d, *J* = 1.3 Hz, 3H), 1.30 (t, *J* = 7.1 Hz, 3H). (major)

^{13}C NMR (75 MHz, Chloroform-*d*) δ 165.88, 148.47, 146.29, 135.82, 128.98, 128.95, 127.56, 124.64, 117.73, 115.18, 60.32, 42.52, 16.04, 14.23. (major)

HRMS (ESI-TOF) m/z $[M+H]^+$ Calcd for $C_{16}H_{18}NO_2$ 256.1338; found 256.1475.

Ethyl (2*E*,4*Z*)-6-(2-bromophenyl)-5-cyano-3-methylhexa-2,4-dienoate (1b)

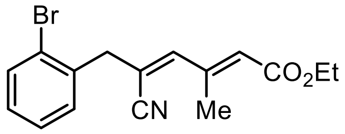

Prepared according to **General Procedure G** using **P2** (2450 mg, 6.46 mmol), ethyl 3-methy-4-oxocrotonate (1.1 mL, 7.75 mmol) and LiO*t*Bu (621 mg, 7.75 mmol) in THF (21.0 mL). The crude oil was dissolved in 1.0 mL of DCM, and crystallization occurred by cooling to 0 °C followed by addition of hexane. Then the suspension was filtered and the title compound (1.55 g, 4.65 mmol, 72% yield, *dr*>20:1) was obtained as a white solid.

^{1}H NMR (400 MHz, Chloroform-*d*) δ 7.62 (d, *J* = 8.0 Hz, 1H), 7.35 (d, *J* = 6.9 Hz, 2H), 7.21 (t, *J* = 7.2 Hz, 1H), 6.51 (s, 1H), 5.99 (s, 1H), 4.21 (q, *J* = 7.1 Hz, 2H), 3.79 (s, 2H), 2.51 (s, 3H), 1.31 (t, *J* = 7.1 Hz, 3H).

^{13}C NMR (101 MHz, Chloroform-*d*) δ 165.90, 148.42, 147.20, 135.06, 133.35, 131.41, 129.41, 128.03, 124.89, 124.73, 117.53, 113.01, 60.37, 42.20, 16.08, 14.22.

HRMS (ESI-TOF) m/z $[M+H]^+$ Calcd for $C_{16}H_{17}BrNO_2$ 334.0443; found 334.0522.

Ethyl (2*E*,4*Z*)-5-cyano-3-methyl-6-(6-(trifluoromethyl)pyridin-3-yl)hexa-2,4-dienoate (1c)

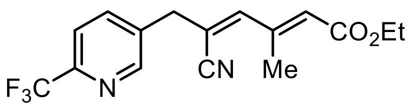

Prepared according to **General Procedure G** using crude product **P3'** (500 mg, 1.49 mmol), ethyl 3-methy-4-oxocrotonate (244 µL, 1.79 mmol) and LiO*t*Bu (143 mg, 1.79 mmol) in THF (5.0 mL). Purification by silica gel flash column chromatography (hexane/EtOAc 8:2) provided the title compound (295 mg, 0.91 mmol, 61% yield, *dr*>20:1) as a colorless oil.

^{1}H NMR (400 MHz, Chloroform-*d*) δ 8.63 (s, 1H), 7.81 (d, *J* = 8.0 Hz, 1H), 7.71 (d, *J* = 8.1 Hz, 1H), 6.64 (s, 1H), 6.04 (s, 1H), 4.21 (q, *J* = 7.1 Hz, 2H), 3.75 (s, 2H), 2.50 (s, 3H), 1.30 (t, *J* = 7.1 Hz, 3H).

^{13}C NMR (101 MHz, Chloroform-*d*) δ 165.64, 150.27, 147.70, 147.61, 137.69, 134.89, 125.80, 122.77, 120.67 (q, *J* = 2.8 Hz), 117.01, 112.75, 60.53, 39.44, 15.91, 14.17.

^{19}F NMR (377 MHz, Chloroform-*d*) δ -67.88.

HRMS (ESI-TOF) m/z $[M+H]^+$ Calcd for $C_{16}H_{16}F_3N_2O_2$ 325.1164; found 325.1188.

Ethyl (2*E*,4*Z*)-5-cyano-3-methyl-6-(thiophen-3-yl)hexa-2,4-dienoate (1d)

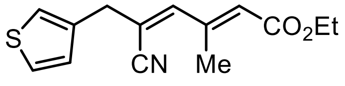

Prepared according to **General Procedure G** using crude product **P4'** (1270 mg, 4.22 mmol), ethyl 3-methy-4-oxocrotonate (692 µL, 5.98 mmol) and LiO*t*Bu (405 mg, 5.06 mmol) in THF (14.0 mL). Purification by silica gel flash column chromatography (hexane/EtOAc 9:1) provided the title compound (816 mg, 3.12 mmol, 74% yield, *dr*=69:31) as a white solid.

^{1}H NMR (400 MHz, Chloroform-*d*) δ 7.36 (t, *J* = 4.6 Hz, 1H), 7.15 (s, 1H), 6.98 (d, *J* = 5.0 Hz, 1H), 6.51 (s, 1H), 6.00 (s, 1H), 4.21 (q, *J* = 6.5 Hz, 2H), 3.68 (s, 2H), 2.52 (s, 3H), 1.31 (t, *J* = 5.2 Hz, 3H). (major)

^{13}C NMR (101 MHz, Chloroform-*d*) δ 165.90, 148.40, 146.21, 135.82, 127.80, 126.71, 124.70, 123.16, 122.44, 114.59, 60.37, 37.04, 16.06, 14.23. (major)

HRMS (ESI-TOF) m/z [M+H]$^+$ Calcd for $C_{14}H_{16}NO_2S$ 262.0902; found 262.0922.

Tert-butyl 3-((2*Z*,4*E*)-2-cyano-6-ethoxy-4-methyl-6-oxohexa-2,4-dien-1-yl)-1H-indole-1-carboxylate (1e)

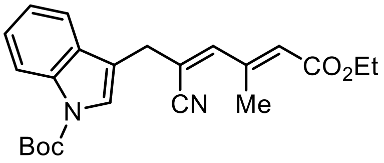

Prepared according to **General Procedure G** using **P5** (210 mg, 0.48 mmol), ethyl 3-methy-4-oxocrotonate (100 μL, 0.72 mmol) and LiO*t*Bu (58 mg, 0.72 mmol) in THF (1.6 mL). Purification by silica gel flash column chromatography (hexane/EtOAc 9:1) provided the title compound (150 mg, 0.38 mmol, 79% yield, *dr*=83:17) as a yellow solid.

^{1}H NMR (400 MHz, Chloroform-*d*) δ 8.18 (d, *J* = 7.2 Hz, 1H), 7.56 (s, 1H), 7.45 (d, *J* = 7.8 Hz, 1H), 7.36 (d, *J* = 8.0 Hz, 1H), 7.28 (d, *J* = 7.2 Hz, 1H), 6.58 (s, 1H), 5.97 (s, 1H), 4.22 – 4.16 (m, 2H), 3.75 (s, 2H), 2.51 (s, 3H), 1.70 (s, 12H), 1.29 (t, *J* = 7.1 Hz, 3H). (major)

^{13}C NMR (101 MHz, Chloroform-*d*) δ 171.11, 165.87, 148.35, 146.78, 146.37, 129.50, 124.84, 124.69, 122.84, 122.68, 118.73, 117.81, 115.55, 114.67, 113.73, 84.02, 60.33, 32.22, 28.20, 16.07, 14.21. (major)

HRMS (ESI-TOF) m/z [M+H]$^+$ Calcd for $C_{23}H_{27}N_2O_4$ 395.1971; found 395.1991.

Ethyl (2*E*,4*Z*)-5-cyano-3-methyl-6,6-diphenylhexa-2,4-dienoate (1f)

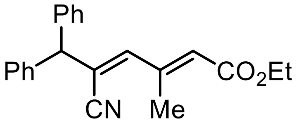

In a two neck round bottom flask equipped with a condenser, magnesium shavings (367 mg, 15.1 mmol) and a granule of iodine were covered by dry THF. While stirring, phenyl bromide (1.8 g, 11.3 mmol) was added dropwise. After disappearance of the brown color (iodine consumption), the remaining halide was diluted in dry THF (2 M) and the solution was added dropwise to the main flask. The mixture was stirred under reflux for 2 hours. After cooling, the Grignard reagent was added dropwise to a stirred solution of CuBr (162 mg, 1.13 mmol) in dry THF (0.5 M), and the reaction was stirred for 15 minutes. Then cyanomethyl phosphonate **P6** (2.0 g, 7.5 mmol) was added and the mixture was stirred overnight at room temperature. The reaction was quenched with a NH_4Cl saturated solution and extracted 3 times with EtOAc. The organic layers were collected, dried over anhydrous Na_2SO_4 and concentrated under reduced pressure. The crude product was diluted in MeOH (0.5 M) and cooled to 0 °C. $NaBH_4$ (428 mg, 11.3 mmol) was added portionwise to reduce the excess of aldehyde. The reaction was quenched with NH_4Cl saturated solution and extracted 3 times with EtOAc. The organic layers were collected, dried with anhydrous Na_2SO_4 and concentrated under reduced pressure. Purification by silica gel flash column chromatography (hexane/EtOAc 9:1) provided the title compound (2.1 g, 6.3 mmol, 85% yield, *dr*>20:1) as a white solid.

^{1}H NMR 400 MHz, Chloroform-*d*) δ 7.43 – 7.30 (m, 6H), 7.24 (d, *J* = 7.2 Hz, 4H), 6.42 (s, 1H), 5.99 (s, 1H), 5.11 (s, 1H), 4.22 (q, *J* = 7.1 Hz, 2H), 2.56 (s, 3H), 1.31 (t, *J* = 7.1 Hz, 3H).

^{13}C NMR (101 MHz, Chloroform-*d*) δ 165.84, 148.41, 147.63, 139.25, 128.99, 128.94, 127.71, 124.87, 118.85, 117.75, 60.37, 56.89, 16.38, 14.24.

HRMS (ESI-TOF) m/z $[M+H]^+$ Calcd for $C_{22}H_{22}NO_2$ 332.1651; found 332.1823.

Ethyl (2*E*,4*Z*)-6,6-bis(4-bromophenyl)-5-cyano-3-methylhexa-2,4-dienoate (1g)

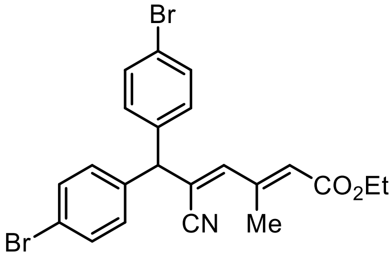

Prepared according to the reported AOCA procedure[40] using **P7** (1.6 g, 5.0 mmol), CuBr (71 mg, 0.50 mmol), NHC-carbene 3-(2-hydroxyethyl)-1-mesityl-4,5-dihydro-1H-imidazol-3-ium hexafluorophosphate(V) (282 mg, 0.75 mmol), LiO*t*Bu (597 mg, 7.5 mmol), 2-(4-bromophenyl)-5,5-dimethyl-1,3,2-dioxaborinane (2.0 g, 7.5 mmol) and ethyl 3-methy-4-oxocrotonate (1.9 mL, 14.0 mmol). Purification by silica gel flash column chromatography (hexane/EtOAc 9:1, *dr*>20:1) provided the title compound (2.1 g, 4.2 mmol, 85% yield) as a colorless oil.

^{1}H NMR (400 MHz, Chloroform-*d*) δ 7.51 (d, *J* = 8.4 Hz, 4H), 7.07 (d, *J* = 8.4 Hz, 4H), 6.41 (s, 1H), 5.99 (s, 1H), 5.00 (s, 1H), 4.21 (q, *J* = 7.1 Hz, 2H), 2.53 (s, 3H), 1.30 (t, *J* = 7.1 Hz, 3H).

^{13}C NMR (101 MHz, Chloroform-*d*) δ 165.68, 148.09, 147.89, 137.76, 132.23, 130.53, 125.59, 122.10, 117.56, 117.31, 60.50, 55.72, 16.26, 14.23.

HRMS (ESI-TOF) m/z $[M+H]^+$ Calcd for $C_{22}H_{20}Br_2NO_2$ 487.9861; found 487.9850.

Ethyl (2*E*,4*Z*)-5-cyano-3,6-dimethylhepta-2,4-dienoate (1h)

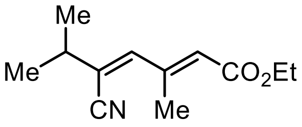

Prepared according to the reported AOCA procedure[40] using diethyl (*E*)-(1-cyanoprop-1-en-1-yl)phosphonate **P8** (640 mg, 3.2 mmol), CuTc (24.03 mg, 0.13 mmol), Me_2Zn (1M in hexane, 4.7 mL, 3.8 mmol) and ethyl 3-methy-4-oxocrotonate (0.86 mL, 6.3 mmol). Purification by silica gel flash column chromatography (hexane/EtOAc 9:1) provided the title compound (524 mg, 2.5 mmol, 79% yield, *dr*>20:1) as a colorless oil.

^{1}H NMR (300 MHz, Chloroform-*d*) δ 6.50 (s, 1H), 5.99 (s, 1H), 4.18 (q, *J* = 7.1 Hz, 2H), 2.59 (hept, *J* = 6.9 Hz, 1H), 2.48 (d, *J* = 0.9 Hz, 3H), 1.28 (t, *J* = 7.1 Hz, 3H), 1.19 (d, *J* = 6.8 Hz, 6H).

^{13}C NMR (101 MHz, Chloroform-*d*) δ 165.94, 148.79, 143.39, 123.88, 122.49, 116.74, 60.16, 35.49, 21.24, 16.22, 14.17.

HRMS (ESI-TOF) m/z $[M+H]^+$ Calcd for $C_{12}H_{18}NO_2$ 208.1338; found 208.1411.

Ethyl (2*E*,4*Z*)-5-cyano-3-methyl-8-phenylocta-2,4-dienoate (1i)

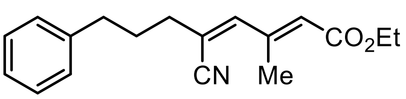

Prepared according to **General Procedure G** using **P9** (470 mg, 1.46 mmol), ethyl 3-methy-4-oxocrotonate (240 µL, 1.75 mmol) and LiO*t*Bu (140 mg, 1.75 mmol) in THF (5.0 mL). Purification by silica gel flash column chromatography (hexane/EtOAc 9:1) provided the title compound (348 mg, 1.23 mmol, 84% yield, *dr*=78:22) as a colorless oil.

^{1}H NMR (400 MHz, Chloroform-*d*) δ 7.36 – 7.17 (m, 5H), 6.47 (s, 1H), 5.99 (s, 1H), 4.22 (q, *J* = 7.1 Hz, 2H), 2.69 (t, *J* = 7.5 Hz, 2H), 2.52 (s, 3H), 2.38 (t, *J* = 7.6 Hz, 2H), 1.98 (p, *J* = 7.2 Hz, 2H), 1.32 (t, *J* = 7.1 Hz, 3H). (major)

^{13}C NMR (101 MHz, Chloroform-*d*) δ 165.93, 148.59, 145.97, 140.94, 128.51, 128.43, 126.16, 124.15, 117.68, 115.49, 60.26, 36.01, 34.69, 29.45, 16.07, 14.24. (major)

HRMS (ESI-TOF) m/z $[M+H]^+$ Calcd for $C_{18}H_{22}NO_2$ 284.1651; found 284.1702.

Ethyl (2*E*,4*Z*)-5-cyano-7-(1,3-dioxolan-2-yl)-3-methylhepta-2,4-dienoate (1j)

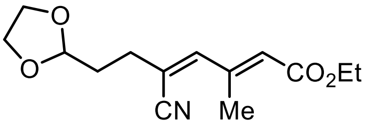

Prepared according to **General Procedure G** using **P10** (393 mg, 1.29 mmol), ethyl 3-methy-4-oxocrotonate (210 µL, 1.54 mmol) and LiO*t*Bu (124 mg, 1.54 mmol) in THF (4.3 mL). Purification by silica gel flash column chromatography (hexane/EtOAc 9:1) provided the title compound (301 mg, 1.13 mmol, 88% yield, *dr*>20:1) as a yellow solid.

^{1}H NMR (400 MHz, Chloroform-*d*) δ 6.53 (s, 1H), 5.99 (s, 1H), 4.93 (t, *J* = 4.1 Hz, 1H), 4.20 (q, *J* = 7.1 Hz, 2H), 4.01 – 3.84 (m, 4H), 2.51 – 2.47 (m, 2H), 2.50 (s, 3H), 2.02 – 1.92 (m, 2H), 1.30 (t, *J* = 7.1 Hz, 3H).

^{13}C NMR (75 MHz, Chloroform-*d*) δ 166.00, 148.55, 145.86, 124.17, 117.55, 115.12, 102.84, 65.05, 60.31, 32.03, 30.87, 16.09, 14.21.

HRMS (ESI-TOF) m/z $[M+H]^+$ Calcd for $C_{14}H_{20}NO_4$ 266.1392; found 266.1521.

Ethyl (2*E*,4*Z*)-7-((tert-butyldimethylsilyl)oxy)-5-cyano-3-methylhepta-2,4-dienoate (1k)

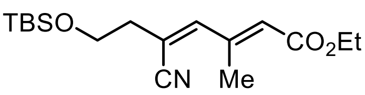

Prepared according to **General Procedure G** using **P11** (250 mg, 0.69 mmol), ethyl 3-methy-4-oxocrotonate (140 µL, 1.0 mmol) and LiO*t*Bu (66 mg, 0.83 mmol) in THF (2.3 mL). Purification by silica gel flash column chromatography (hexane/EtOAc 95:5) provided the title compound (198 mg, 0.61 mmol, 89% yield, *dr*>20:1) as a white solid.

^{1}H NMR (400 MHz, Chloroform-*d*) δ 6.55 (s, 1H), 6.01 (s, 1H), 4.22 (q, *J* = 7.1 Hz, 2H), 3.85 (t, *J* = 5.9 Hz, 2H), 2.55 – 2.52 (m, 5H), 1.32 (t, *J* = 7.2 Hz, 3H), 0.90 (s, 9H), 0.08 (s, 6H).

^{13}C NMR (101 MHz, Chloroform-*d*) δ 166.07, 148.61, 147.67, 124.18, 117.76, 112.81, 60.40, 60.34, 39.96, 25.81, 18.21, 16.09, 14.23, -5.41.

HRMS (ESI-TOF) m/z $[M+H]^+$ Calcd for $C_{17}H_{30}NO_3Si$ 324.1995; found 324.2177.

Ethyl (2*E*,4*Z*)-5-cyano-3-methylocta-2,4,7-trienoate (1l)

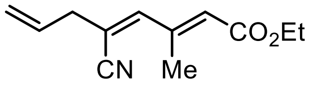

Prepared according to **General Procedure G** using **P12** (623 mg, 2.54 mmol), ethyl 3-methy-4-oxocrotonate (420 µL, 3.0 mmol) and LiO*t*Bu (342 mg, 3.05 mmol) in THF (8.5 mL). Purification by silica gel flash column chromatography (hexane/EtOAc 9:1) provided the title compound (490 mg, 2.39 mmol, 94% yield, *dr*=92:8) as a white solid.

^{1}H NMR (400 MHz, Chloroform-*d*) δ 6.51 (s, 1H), 5.99 (s, 1H), 5.80 (td, *J* = 17.4, 6.7 Hz, 1H), 5.28 – 5.14 (m, 2H), 4.18 (q, *J* = 7.1 Hz, 2H), 3.06 (d, *J* = 6.6 Hz, 2H), 2.49 (s, 3H), 1.28 (t, *J* = 7.1 Hz, 3H). (major)

^{13}C NMR (101 MHz, Chloroform-*d*) δ 165.91, 148.44, 146.20, 132.13, 124.43, 119.30, 117.62, 113.85, 60.29, 40.35, 16.03, 14.19. (major)

HRMS (ESI-TOF) m/z [M+H]$^+$ Calcd for $C_{12}H_{16}NO_2$ 206.1181; found 206.1222.

Ethyl (2*E*,4*Z*)-5-cyano-3-methylhexa-2,4-dienoate (1m)

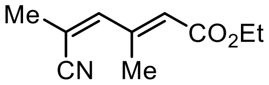

Prepared according to **General Procedure G** using **P13** (2.1 g, 10.8 mmol), ethyl 3-methy-4-oxocrotonate (1.9 mL, 14.0 mmol) and LiO*t*Bu (1.3 g, 16.2 mmol) in THF (36.0 mL). Purification by silica gel flash column chromatography (hexane/EtOAc 9:1) provided the title compound (1.8 g, 9.9 mmol, 92% yield, *dr*>20:1) as a colorless oil.

^{1}H NMR (400 MHz, Chloroform-*d*) δ 6.51 (s, 1H), 5.99 (s, 1H), 4.22 (q, *J* = 7.1 Hz, 2H), 2.51 (s, 3H), 2.13 (s, 3H), 1.32 (t, *J* = 7.1 Hz, 3H).

^{13}C NMR (101 MHz, Chloroform-*d*) δ 166.05, 148.59, 146.34, 123.94, 118.22, 110.49, 60.32, 22.79, 16.00, 14.23.

HRMS (ESI-TOF) m/z [M+H]$^+$ Calcd for $C_{10}H_{14}NO_2$ 180.1025; found 180.1098.

Ethyl (2*E*,4*Z*)-5-cyano-3-methyl-5-phenylpenta-2,4-dienoate (1n)

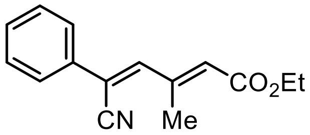

Prepared according to **General Procedure G** using crude product **P14** (400 mg, 1.58 mmol), ethyl 3-methy-4-oxocrotonate (260 µL, 1.9 mmol) and LiO*t*Bu (152 mg, 1.90 mmol) in THF (5.0 mL). Purification by silica gel flash column chromatography (hexane/EtOAc 9:1) provided the title compound (301 mg, 1.25 mmol, 79% yield, *dr*=83:17) as a white solid.

^{1}H NMR (400 MHz, Chloroform-*d*) δ 7.63 (d, *J* = 7.9 Hz, 2H), 7.45 – 7.39 (m, 3H), 7.09 (s, 1H), 6.21 (s, 1H), 4.24 (q, *J* = 7.1 Hz, 2H), 2.64 (s, 3H), 1.33 (t, *J* = 7.1 Hz, 3H). (major)

^{13}C NMR (101 MHz, Chloroform-*d*) δ 165.90, 148.67, 144.37, 133.99, 129.93, 129.16, 126.33, 125.66, 116.99, 115.58, 60.43, 16.32, 14.25. (major)

HRMS (ESI-TOF) m/z [M+H]$^+$ Calcd for $C_{15}H_{16}NO_2$ 242.1181; found 242.1220.

Ethyl (2*E*,4*Z*)-5-cyano-5-(4-methoxyphenyl)-3-methylpenta-2,4-dienoate (1o)

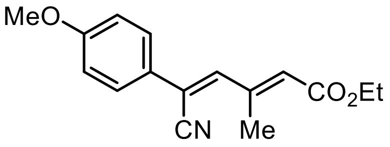

Prepared according to **General Procedure G** using crude product **P15** (1.0 g, 3.6 mmol), ethyl 3-methy-4-oxocrotonate (580 µL, 4.3 mmol) and LiO*t*Bu (343 mg, 4.3 mmol) in THF (12.0 mL). Purification by silica gel flash column chromatography (hexane/EtOAc 9:1) provided the title compound (697 mg, 2.6 mmol, 72% yield, *dr*>20:1) as a white solid.

^{1}H NMR (400 MHz, Chloroform-*d*) δ 7.57 (d, *J* = 8.7 Hz, 2H), 7.04 – 6.88 (m, 3H), 6.17 (s, 1H), 4.24 (q, *J* = 7.1 Hz, 2H), 3.87 (s, 3H), 2.64 (s, 3H), 1.33 (t, *J* = 7.1 Hz, 3H).

^{13}C NMR (101 MHz, Chloroform-*d*) δ 166.05, 161.04, 148.97, 142.06, 127.75, 126.45, 124.87, 117.15, 115.09, 114.54, 60.35, 55.48, 16.35, 14.26.

HRMS (ESI-TOF) m/z [M+H]$^+$ Calcd for $C_{16}H_{18}NO_3$ 272.1287; found 272.1449.

Ethyl (2*E*,4*Z*)-5-cyano-3-methyl-5-(m-tolyl)penta-2,4-dienoate (1p)

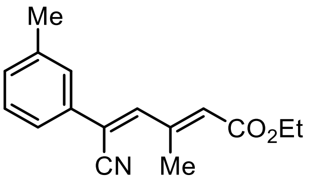

Prepared according to **General Procedure G** using crude product **P16** (2.0 g, 7.6 mmol), ethyl 3-methy-4-oxocrotonate (1.3 mL, 9.1 mmol) and LiO*t*Bu (730 mg, 9.1 mmol) in THF (25.0 mL). Purification by silica gel flash column chromatography (hexane/EtOAc 9:1) provided the title compound (1.6 g, 6.2 mmol, 81% yield, *dr*>20:1) as a white solid.

^{1}H NMR (300 MHz, Chloroform-*d*) δ 7.43 (d, *J* = 7.4 Hz, 2H), 7.34 (t, *J* = 8.1 Hz, 1H), 7.24 (d, *J* = 7.5 Hz, 1H), 7.08 (s, 1H), 6.21 (s, 1H), 4.25 (q, *J* = 7.1 Hz, 2H), 2.64 (s, 3H), 2.42 (s, 3H), 1.34 (t, *J* = 7.1 Hz, 3H).

^{13}C NMR (75 MHz, Chloroform-*d*) δ 165.95, 148.76, 144.16, 139.01, 133.95, 130.73, 129.02, 127.02, 125.47, 123.43, 117.07, 115.69, 60.42, 21.42, 16.35, 14.24.

HRMS (ESI-TOF) m/z [M+H]$^+$ Calcd for $C_{16}H_{18}NO_2$ 256.1338; found 256.1424.

Ethyl (2*E*,4*E*)-5-cyano-5-fluoro-3-methylpenta-2,4-dienoate (1q)

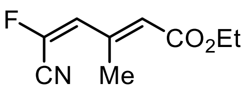

Prepared according to **General Procedure G** using **P17** (240 mg, 1.08 mmol), ethyl 3-methy-4-oxocrotonate (180 µL, 1.29 mmol) and LiO*t*Bu (145 mg, 1.29 mmol) in THF (3.6 mL). Purification by silica gel flash column chromatography (hexane/EtOAc 9:1) provided the title compound (177 mg, 0.97 mmol, 90% yield, *dr*=86:14) as a white solid.

^{1}H NMR (400 MHz, Chloroform-*d*) δ 6.64 (d, *J* = 17.1 Hz, 1H), 6.02 (s, 1H), 4.22 (q, *J* = 7.1 Hz, 2H), 2.48 (s, 3H), 1.32 (t, *J* = 7.1 Hz, 3H).

^{13}C NMR (101 MHz, Chloroform-*d*) δ 165.27, 143.18 (d, *J* = 6.1 Hz), 133.47 (d, *J* = 244.3 Hz), 128.75 (d, *J* = 25.3 Hz), 125.77, 111.61 (d, *J* = 46.4 Hz), 60.58, 15.58, 14.17.

^{19}F NMR (377 MHz, Chloroform-*d*) δ -114.63 (d, *J* = 17.1 Hz).

HRMS (ESI-TOF) m/z $[M+H]^+$ Calcd for $C_9H_{11}FNO_2$ 184.0774; found 184.0779.

Ethyl (2*E*,4*E*)-5-cyano-3-methyl-5-(methylthio)penta-2,4-dienoate (1r)

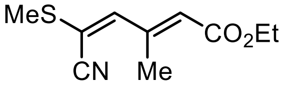

Prepared according to **General Procedure G** using **P18** (475 mg, 2.1 mmol), ethyl 3-methy-4-oxocrotonate (436 µL, 3.2 mmol) and LiO*t*Bu (255 mg, 3.2 mmol) in THF (7.0 mL). Purification by silica gel flash column chromatography (hexane/EtOAc 8:2) provided the title compound (410 mg, 1.9 mmol, 91% yield, *dr*>20:1) as a pale yellow oil.

^{1}H NMR (400 MHz, Chloroform-*d*) δ 6.70 (s, 1H), 5.95 (s, 1H), 4.17 (q, *J* = 7.1 Hz, 2H), 2.50 (d, *J* = 1.4 Hz, 3H), 2.47 (s, 3H), 1.27 (t, *J* = 7.1 Hz, 3H).

^{13}C NMR (101 MHz, Chloroform-*d*) δ 166.02, 147.91, 143.78, 124.59, 114.16, 112.81, 60.44, 16.43, 15.80, 14.28.

HRMS (ESI-TOF) m/z $[M+H]^+$ Calcd for $C_{10}H_{14}NO_2S$ 212.0745; found 212.0757.

Methyl (2*E*,4*Z*)-5-cyano-3-ethyl-6-phenylhexa-2,4-dienoate (1s)

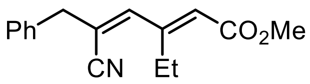

Prepared according to **General Procedure G** using **P1** (270 mg, 1.0 mmol), **A2** (215 mg, 1.5 mmol) and LiO*t*Bu (121 mg, 1.5 mmol) in THF (3.3 mL). Purification by silica gel flash column chromatography (hexane/EtOAc 9:1) provided the title compound (220 mg, 0.86 mmol, 85% yield, *dr*>20:1) as a yellow solid.

^{1}H NMR (400 MHz, Chloroform-*d*) δ 7.64 (s, 1H), 7.38 – 7.34 (m, 2H), 7.30 – 7.27 (m, 3H), 5.88 – 5.87 (m, 1H), 3.71 (s, 3H), 3.66 (s, 2H), 2.64 (q, *J* = 7.4, 2H), 1.13 (t, *J* = 7.4 Hz, 3H).

^{13}C NMR (101 MHz, Chloroform-*d*) δ 166.14, 154.08, 142.03, 136.22, 129.11, 129.01, 127.52, 120.41, 117.88, 115.19, 51.62, 42.54, 28.70, 12.82.

HRMS (ESI-TOF) m/z $[M+H]^+$ Calcd for $C_{16}H_{18}NO_2$ 256.1338; found 256.1351.

Methyl (2*Z*,4*Z*)-5-cyano-3-isopropyl-6-phenylhexa-2,4-dienoate (1t)

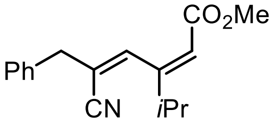

Prepared according to a modified **General Procedure G** using **P1** (220 mg, 0.82 mmol), **A4** (193 mg, 1.2 mmol) and LiO*t*Bu (99 mg, 1.2 mmol) in THF (2.7 mL). Purification by silica gel flash column chromatography (hexane/EtOAc 9:1) provided the title compound (210 mg, 0.78 mmol, 95% yield, *dr*>20:1) as a white solid.

^{1}H NMR (400 MHz, Chloroform-*d*) δ 7.38 – 7.34 (m, 2H), 7.30 – 7.27 (m, 3H), 7.22 (s, 1H), 5.88 (s, 1H), 3.69 (s, 3H), 3.66 (s, 2H), 2.95 – 2.88 (m, 1H), 1.12 (t, *J* = 6.8 Hz, 6H).

^{13}C NMR (101 MHz, Chloroform-*d*) δ 166.35, 158.98, 143.42, 136.23, 129.16, 129.01, 127.49, 118.12, 117.55, 115.32, 51.65, 41.79, 34.50, 21.75.

HRMS (ESI-TOF) m/z $[M+H]^+$ Calcd for $C_{17}H_{20}NO_2$ 270.1494; found 270.0887.

(*Z*)-2-benzyl-3-(5-oxo-2,5-dihydrofuran-3-yl)acrylonitrile (1u)

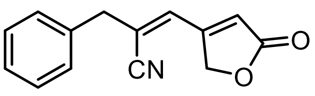

Prepared according to **General Procedure G** using **P1** (1.0 g, 3.72 mmol), **A5** (500 mg, 4.46 mmol) and LiO*t*Bu (357 mg, 4.46 mmol) in THF (12.0 mL). Purification by silica gel flash column chromatography (hexane/EtOAc 6:4) provided the title compound (587 mg, 2.60 mmol, 70% yield, *dr*>20:1) as a colorless oil.

^{1}H NMR (400 MHz, Chloroform-*d*) δ 7.37 (dq, *J* = 14.0, 7.1 Hz, 3H), 7.24 (d, *J* = 7.4 Hz, 2H), 6.86 (s, 1H), 6.30 (s, 1H), 5.25 (s, 2H), 3.72 (s, 2H).

^{13}C NMR (101 MHz, Chloroform-*d*) δ 172.17, 157.48, 134.52, 133.27, 129.26, 129.07, 128.02, 122.25, 120.11, 117.24, 71.11, 41.86.

HRMS (ESI-TOF) m/z [M+H]$^+$ Calcd for $C_{14}H_{12}NO_2$ 226.0868; found 226.1032.

Ethyl (*E*)-3-((*Z*)-2-cyanoprop-1-en-1-yl)hepta-2,6-dienoate (1v)

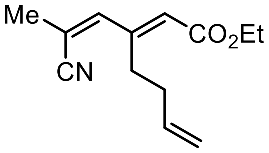

Prepared according to **General Procedure G** using **P13** (252 mg, 1.3 mmol), **A6** (200 mg, 1.1 mmol) and LiO*t*Bu (123 mg, 1.5 mmol) in THF (4.3 mL). Purification by silica gel flash column chromatography (hexane/EtOAc 9:1) provided the title compound (229 mg, 1.0 mmol, 95% yield, *dr*>20:1) as a yellow solid.

^{1}H NMR (300 MHz, Chloroform-*d*) δ 6.46 (s, 1H), 6.07 (s, 1H), 5.86 (ddt, *J* = 16.9, 10.2, 6.7 Hz, 1H), 5.20 – 4.90 (m, 2H), 4.25 – 4.10 (m, 2H), 3.06 – 2.91 (m, 2H), 2.26 (q, *J* = 7.3, 6.9 Hz, 2H), 2.10 (s, 3H), 1.30 (t, *J* = 7.2 Hz, 3H).

^{13}C NMR (75 MHz, Chloroform-*d*) δ 165.63, 152.25, 145.22, 137.00, 122.85, 117.86, 115.51, 110.43, 60.31, 32.98, 29.02, 22.45, 14.19.

HRMS (ESI-TOF) m/z [M+H]$^+$ Calcd for $C_{13}H_{18}NO_2$ 220.1338; found 220.1344.

Ethyl (2*E*,4*Z*)-3-(2-(1,3-dioxolan-2-yl)ethyl)-5-cyano-6-phenylhexa-2,4-dienoate (1w)

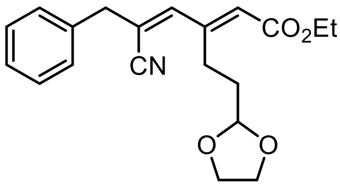

Prepared according to **General Procedure G** using **P1** (576 mg, 2.16 mmol), **A7** (400 mg, 1.75 mmol) and LiO*t*Bu (173 mg, 2.16 mmol) in THF (7.0 mL). Purification by silica gel flash column chromatography (hexane/EtOAc 8:2) provided the title compound (490 mg, 1.44 mmol, 82% yield, *dr*>20:1) as a colorless oil.

^{1}H NMR (400 MHz, Chloroform-*d*) δ 7.40 – 7.27 (m, 3H), 7.23 (d, *J* = 7.4 Hz, 2H), 6.51 (s, 1H), 6.13 (s, 1H), 4.95 (t, *J* = 4.6 Hz, 1H), 4.20 (q, *J* = 7.1 Hz, 2H), 4.01 – 3.80 (m, 4H), 3.63 (s, 2H), 3.01 – 2.93 (m, 2H), 1.90 – 1.83 (m, 2H), 1.29 (t, *J* = 7.1 Hz, 3H).

^{13}C NMR (101 MHz, Chloroform-*d*) δ 165.43, 152.17, 145.18, 135.72, 129.00, 128.91, 127.55, 122.73, 117.29, 115.20, 103.67, 64.92, 60.39, 42.08, 32.73, 24.78, 14.20.

HRMS (ESI-TOF) m/z $[M+H]^+$ Calcd for $C_{20}H_{24}NO_4$ 342.1705; found 342.1886.

Ethyl (2*Z*,4*Z*)-5-cyano-3,6-diphenylhexa-2,4-dienoate (1x)

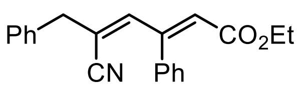

Prepared according to **General Procedure G** using **P1** (1.6 g, 5.6 mmol), **A8** (950 mg, 4.7 mmol) and LiO*t*Bu (447 mg, 5.6 mmol) in THF (19.0 mL). Purification by silica gel flash column chromatography (hexane/EtOAc 9:1) provided the title compound (1.7 g, 5.4 mmol, 96% yield, *dr*>20:1) as a white solid.

^{1}H NMR (400 MHz, Chloroform-*d*) δ 7.45 – 7.19 (m, 10H), 6.69 (s, 1H), 6.27 (s, 1H), 4.04 (q, *J* = 7.1 Hz, 2H), 3.65 (s, 2H), 1.09 (t, *J* = 7.1 Hz, 3H). (major)

^{13}C NMR (101 MHz, Chloroform-*d*) δ 165.10, 149.56, 144.55, 135.67, 135.56, 129.01, 128.97, 128.45, 128.28, 127.62, 127.57, 124.59, 117.78, 115.93, 60.51, 42.57, 13.86. (major)

HRMS (ESI-TOF) m/z $[M+H]^+$ Calcd for $C_{21}H_{20}NO_2$ 318.1494; found 318.1435.

Ethyl (2*Z*,4*Z*)-5-cyano-3-(naphthalen-2-yl)-6-phenylhexa-2,4-dienoate (1y)

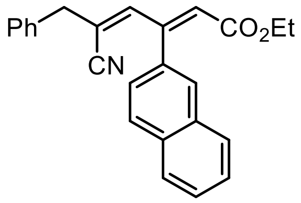

Prepared according to **General Procedure G** using **P1** (220 mg, 0.82 mmol), **A9** (314 mg, 1.2 mmol) and LiO*t*Bu (114 mg, 1.2 mmol) in THF (2.7 mL). Purification by silica gel flash column chromatography (hexane/EtOAc 9:1) provided the title compound (278 mg, 0.76 mmol, 92% yield, *dr*>20:1) as a yellow oil.

^{1}H NMR (400 MHz, Chloroform-*d*) δ 7.90 – 7.81 (m, 3H), 7.68 (d, *J* = 1.8 Hz, 1H), 7.51 – 7.49 (m, 2H), 7.39 – 7.29 (m, 4H), 7.24 (d, *J* = 7.1 Hz, 2H), 6.74 (s, 1H), 6.39 (s, 1H), 4.01 (q, *J* = 7.1 Hz, 2H), 3.65 (s, 2H), 1.02 (t, *J* = 7.1 Hz, 3H).

^{13}C NMR (101 MHz, Chloroform-*d*) δ 165.23, 149.43, 144.54, 135.75, 133.44, 133.32, 133.05, 129.11, 129.07, 128.31, 127.99, 127.97, 127.81, 127.68, 126.69, 126.45, 126.29, 124.37, 117.66, 116.32, 60.63, 42.47, 13.94.

HRMS (ESI-TOF) m/z $[M+H]^+$ Calcd for $C_{25}H_{22}NO_2$ 368.1651; found 368.1644.

Ethyl (2*Z*,4*Z*)-5-cyano-6-phenyl-3-(pyridin-4-yl)hexa-2,4-dienoate (1z)

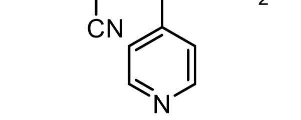

Prepared according to **General Procedure G** using **P1** (230 mg, 0.86 mmol), **A10** (265 mg, 1.29 mmol) and LiO*t*Bu (119 mg, 1.29 mmol) in THF (3.0 mL). **A10** was employed as a 6:4 *dr* mixture in excess, which resulted in preferential olefination of *trans*-**A10**. Purification by silica gel flash column chromatography (hexane/EtOAc 6:4) allowed to further separate undesired minor α-cis isomers and provided the title compound (238 mg, 0.75 mmol, 87% yield, *dr*=80:20 at γ-olefin) as a yellow oil.

^{1}H NMR (400 MHz, Chloroform-*d*) δ 8.59 (d, *J* = 6.1 Hz, 2H), 7.45 – 7.37 (m, 4H), 7.19 – 7.15 (m, 3H), 6.75 (s, 1H), 6.28 (s, 1H), 4.01 (q, *J* = 7.1 Hz, 2H), 3.62 (s, 2H), 1.06 (t, *J* = 7.1 Hz, 3H). (major)

^{13}C NMR (101 MHz, Chloroform-*d*) δ 164.49, 149.83, 146.86, 143.92, 142.43, 135.31, 129.18, 129.04, 127.82, 125.85, 123.30, 118.57, 115.73, 60.97, 42.62, 13.86. (major)

HRMS (ESI-TOF) m/z $[M+H]^+$ Calcd for $C_{20}H_{19}N_2O_2$ 319.1447; found 319.1456.

Ethyl (2E,4E)-5-cyano-5-fluoro-3,4-dimethylpenta-2,4-dienoate (1aa)

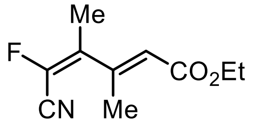

Prepared according to **General Procedure G**, but stirring for 2 hours at room temperature, using **P17** (214 mg, 1.10 mmol), **A11** (156 mg, 1.00 mmol) and KO*t*Bu (146 mg, 1.30 mmol) in THF (3.0 mL). Purification by silica gel flash column chromatography (hexane/EtOAc 9:1) provided the title compound (120 mg, 0.61 mmol, 61% yield, *dr*=1:1) as a colorless oil.

^{1}H NMR (400 MHz, Chloroform-*d*) δ 5.9 (s, 1H), 4.2 (q, *J*=7.1 Hz, 2H), 2.3 (s, 3H), 2.1 (d, *J*=3.9 Hz, 3H), 1.3 (t, *J*=7.1 Hz, 3H)

^{13}C NMR (101 MHz, Chloroform-*d*) δ 165.6, 148.2 (d, *J*=2.3 Hz), 135.2 (d, *J*=10.1 Hz), 129.2 (d, *J*=244.7 Hz), 121.6 (d, *J*=4.2 Hz), 111.8 (d, *J*=47.4 Hz), 60.4, 17.2, 17.0, 14.2.

^{19}F NMR (377 MHz, Chloroform-*d*) δ -121.50.

HRMS (ESI-TOF) m/z $[M+H]^+$ Calcd for $C_{10}H_{13}FNO_2$ 198.0930; found 198.0930.

5 Synthesis and Characterization of Cyclopentadienes

Ethyl (*R*)-2-amino-3-benzyl-5-methylcyclopenta-1,3-diene-1-carboxylate (3a)

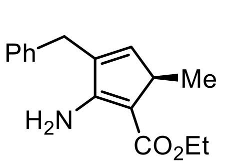

Prepared according to **General Procedure H** using **1a** (37 mg, 0.145 mmol), $Cu(OAc)_2$ (0.66 mg, 2.89 µmol), Josiphos (1.73 mg, 3.19 µmol), TMDSO (55 µL, 0.290 mmol), KF (36.4 mg, 0.580 mmol) and H_2O (6 µL, 0.290 mmol) in THF (0.58 mL). Purification by silica gel flash column chromatography (hexane/EtOAc 9:1) provided the title compound (37 mg, 0.143 mmol, 99% yield, *er*=99:1) as a white waxy solid.

HPLC: Phenomenex cellulose-1, Hexane:2-propanol 90:10, flow: 1.0 mL/min, 315 nm, t_R = 13.7 min (major) and t_R = 17.0 min (minor).

^{1}H NMR (400 MHz, Chloroform-*d*) δ 7.38 – 7.16 (m, 5H), 6.20 (s, 1H), 5.60 (s, 1H), 4.31 – 4.13 (m, 2H), 3.65 (s, 2H), 3.34 (q, *J* = 7.4, 6.9 Hz, 1H), 1.32 (t, *J* = 7.1 Hz, 3H), 1.27 (d, *J* = 7.4 Hz, 3H).

^{13}C NMR (75 MHz, Chloroform-*d*) δ 166.81, 160.13, 145.04, 137.78, 137.37, 128.77, 128.57, 126.70, 103.38, 58.51, 42.66, 33.04, 15.86, 14.77.

HRMS (ESI-TOF) m/z $[M+H]^+$ Calcd for $C_{16}H_{20}NO_2$ 258.1494; found 258.1576.

$[\alpha]^{25}_D$ = -46.0 (c = 1.0, $CHCl_3$).

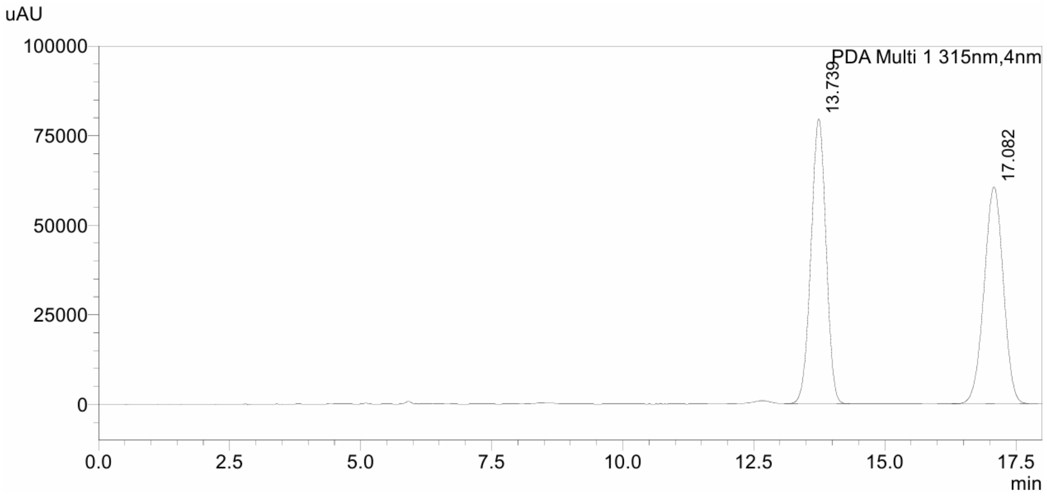

PDA Ch1 315nm

Peak#	Ret. Time	Area	Area%
1	13.739	1595786	50.766
2	17.082	1547610	49.234
Total		3143395	100.000

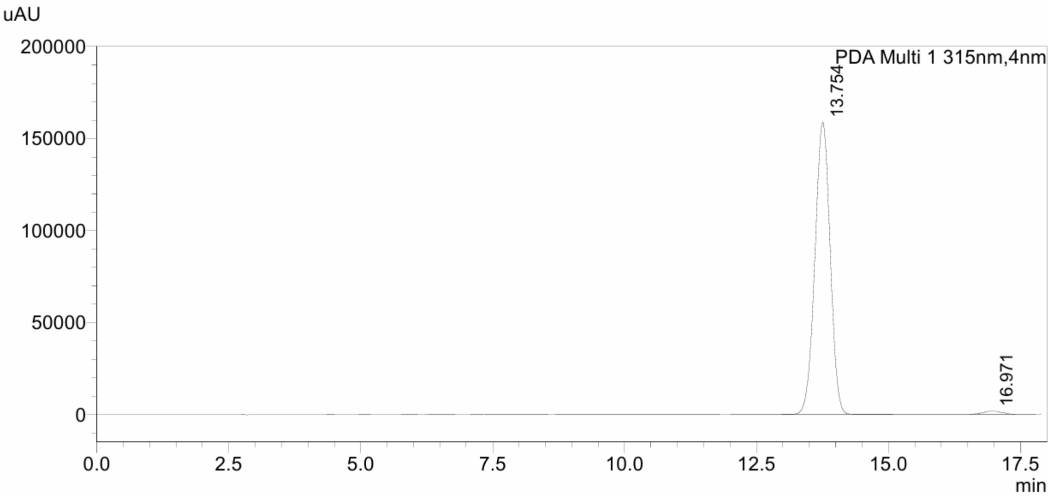

PDA Ch1 315nm

Peak#	Ret. Time	Area	Area%
1	13.754	3195946	98.732
2	16.971	41060	1.268
Total		3237006	100.000

Ethyl (*R*)-2-amino-3-(2-bromobenzyl)-5-methylcyclopenta-1,3-diene-1-carboxylate (3b)

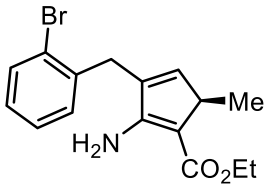

Prepared according to **General Procedure H** using **1b** (66 mg, 0.196 mmol), $Cu(OAc)_2$ (0.71 mg, 3.91 µmol), Josiphos (2.34 mg, 4.31 µmol), TMDSO (69 µL, 0.392 mmol), KF (45.5 mg, 0.784 mmol) and H_2O (7 µL, 0.392 mmol) in THF (0.78 mL). Purification by silica gel flash column chromatography (hexane/EtOAc 9:1) provided the title compound (59 mg, 0.176 mmol, 90% yield, *er*=99:1) as a translucent white solid.

HPLC: Phenomenex cellulose-1, Hexane:2-propanol 96:4, flow: 1.0 mL/min, 338 nm, t_R = 25.5 min (minor) and t_R = 28.6 min (major).

^{1}H NMR (400 MHz, Chloroform-*d*) δ 7.59 (d, *J* = 8.0 Hz, 1H), 7.28 (t, *J* = 7.4 Hz, 1H), 7.23 – 7.08 (m, 2H), 6.14 (s, 1H), 5.66 (s, 2H), 4.31 – 4.15 (m, 2H), 3.74 (s, 2H), 3.33 (q, *J* = 7.8, 7.1 Hz, 1H), 1.32 (t, *J* = 7.1 Hz, 3H), 1.24 (d, *J* = 7.4 Hz, 3H).

^{13}C NMR (101 MHz, Chloroform-*d*) δ 166.81, 145.56, 145.52, 137.39, 136.29, 132.97, 130.42, 128.42, 127.76, 124.61, 103.31, 58.62, 42.80, 33.05, 15.83, 14.77.

HRMS (ESI-TOF) m/z $[M+H]^+$ Calcd for $C_{16}H_{19}BrNO_2$ 336.0599; found 336.0531.

$[\alpha]^{25}_D$ = -64.7 (c = 0.37, $CHCl_3$).

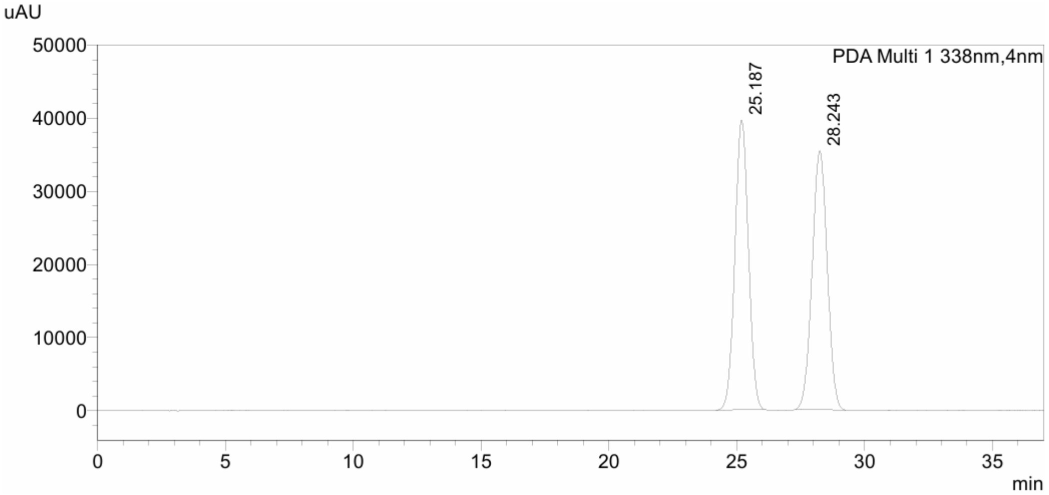

PDA Ch1 338nm

Peak#	Ret. Time	Area	Area%
1	25.187	1453788	50.001
2	28.243	1453714	49.999
Total		2907501	100.000

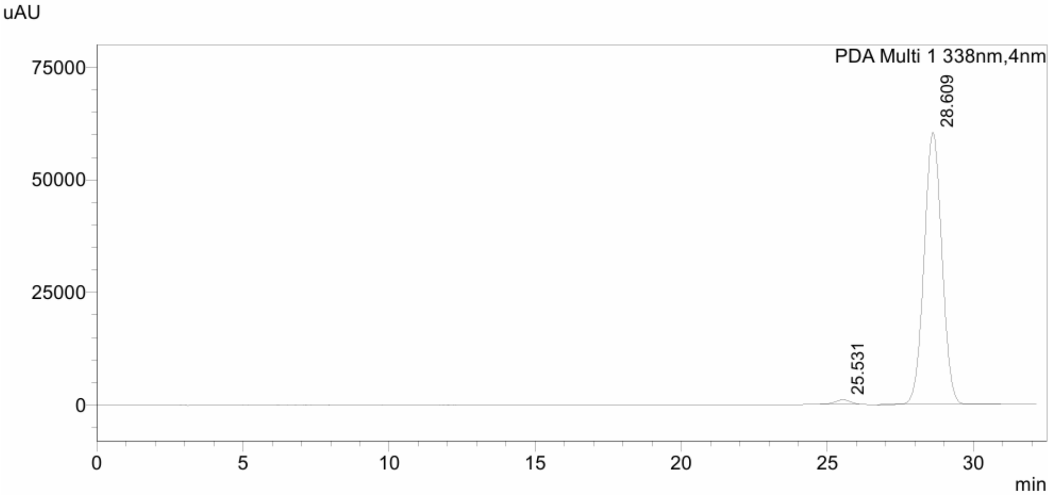

PDA Ch1 338nm

Peak#	Ret. Time	Area	Area%
1	25.531	32331	1.250
2	28.609	2554682	98.750
Total		2587013	100.000

Ethyl (*R*)-2-amino-5-methyl-3-((6-(trifluoromethyl)pyridin-3-yl)methyl)cyclopenta-1,3-diene-1-carboxylate (3c)

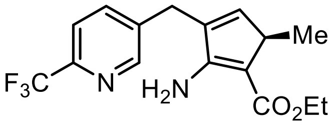

Prepared according to **General Procedure H** using **1c** (64 mg, 0.196 mmol), $Cu(OAc)_2$ (0.71 mg, 3.92 μmol), Josiphos (2.34 mg, 4.31 μmol), TMDSO (69 μL, 0.392 mmol), KF (45.5 mg, 0.784 mmol) and H_2O (7 μL, 0.392 mmol) in THF (0.78 mL). Purification by silica gel flash column chromatography (hexane/EtOAc 7:3) provided the title compound (63.30 mg, 0.194 mmol, 99% yield, *er*=98.5:1.5) as a transparent oil.

HPLC: Phenomenex cellulose-1, Hexane:2-propanol 80:20, flow: 1.0 mL/min, 350 nm, t_R = 27.8 min (minor) and t_R = 43.0 min (major).

^{1}H NMR (400 MHz, Chloroform-*d*) δ 8.61 (s, 1H), 7.71 (d, *J* = 8.3 Hz, 1H), 7.65 (d, *J* = 8.1 Hz, 1H), 6.11 (s, 1H), 5.61 (s, 2H), 4.30 – 4.12 (m, 2H), 3.69 (s, 2H), 3.31 (q, *J* = 7.5 Hz, 1H), 1.30 (t, *J* = 7.1 Hz, 3H), 1.22 (d, *J* = 7.4 Hz, 3H).

^{13}C NMR (101 MHz, Chloroform-*d*) δ 166.71, 158.75, 150.27, 146.76 (q, *J* = 34.8 Hz), 145.60, 137.46, 136.93, 135.84, 122.92, 120.43 (q, *J* = 2.8 Hz), 104.03, 58.69, 42.93, 29.79, 15.63, 14.69.

^{19}F NMR (377 MHz, Chloroform-*d*) δ -67.78.

HRMS (ESI-TOF) m/z $[M+H]^+$ Calcd for $C_{16}H_{18}F_3N_2O_2$ 327.1320; found 327.1322.

$[\alpha]^{25}_D$ = -51.0 (c = 2.42, $CHCl_3$)

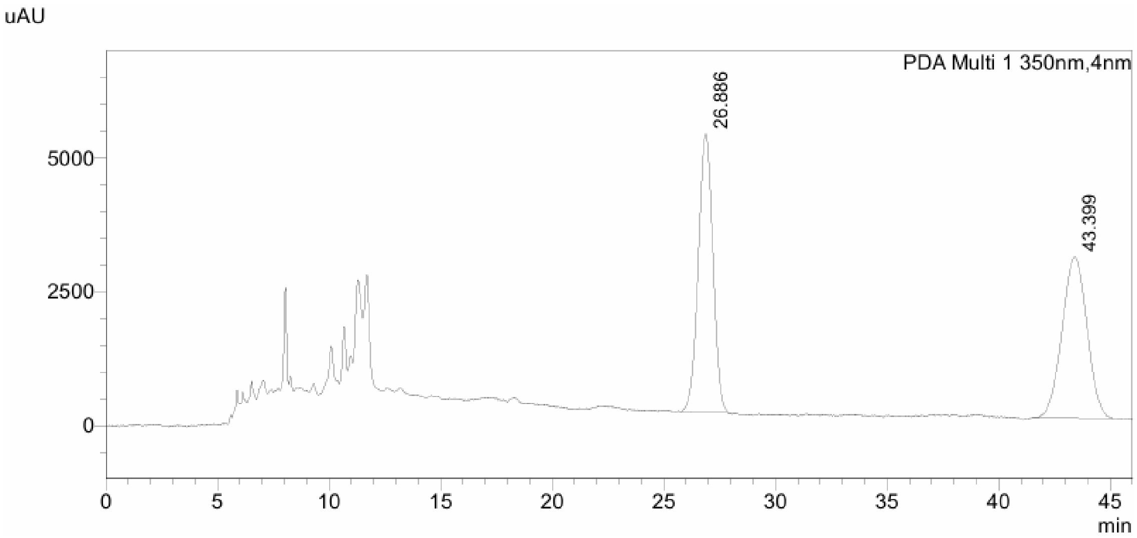

PDA Ch1 350nm

Peak#	Ret. Time	Area	Area%
1	26.886	236859	50.171
2	43.399	235243	49.829
Total		472102	100.000

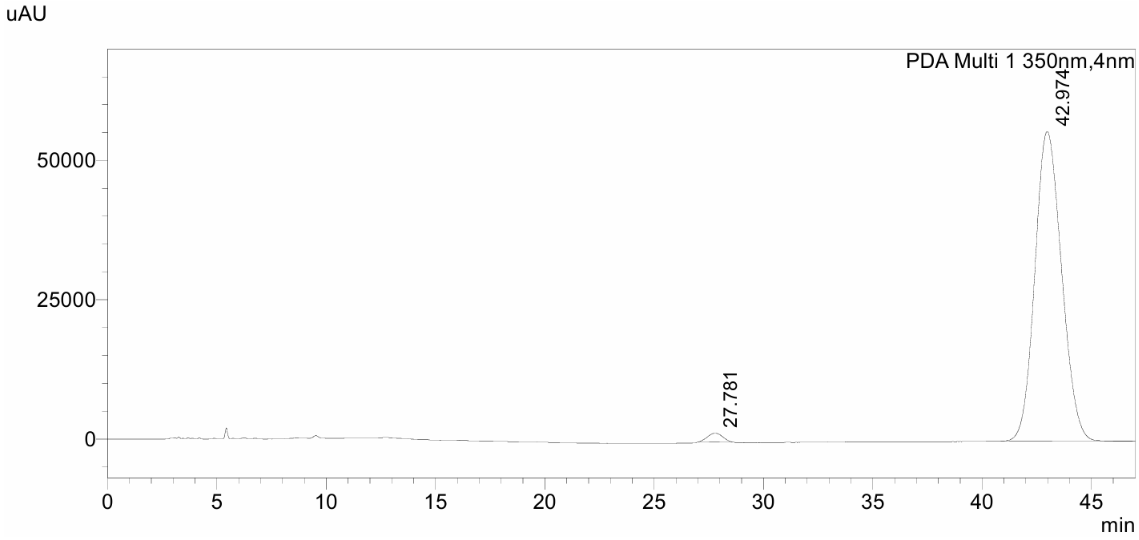

PDA Ch1 350nm

Peak#	Ret. Time	Area	Area%
1	27.781	78640	1.633
2	42.974	4738506	98.367
Total		4817146	100.000

Ethyl (*R*)-2-amino-5-methyl-3-(thiophen-3-ylmethyl)cyclopenta-1,3-diene-1-carboxylate (3d)

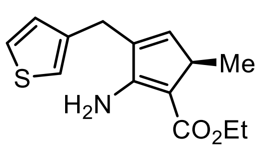

Prepared according to **General Procedure H** using **1d** (51 mg, 0.196 mmol), $Cu(OAc)_2$ (0.71 mg, 3.92 µmol), Josiphos (2.34 mg, 4.31 µmol), TMDSO (69 µL, 0.392 mmol), KF (45.5 mg, 0.784 mmol) and H_2O (7 µL, 0.392 mmol) in THF (0.78 mL). Purification by silica gel flash column chromatography (hexane/diethyl ether 55:45) provided the title compound (44.23 mg, 0.168 mmol, 86% yield, *er*=98.5:1.5) as a white solid.

HPLC: Phenomenex cellulose-1, Hexane:2-propanol 94:6, flow: 1.0 mL/min, 317 nm, t_R = 25.1 min (major) and t_R = 29.0 min (minor).

^{1}H NMR (400 MHz, Chloroform-*d*) δ 7.31 (t, *J* = 2.4 Hz, 1H), 7.03 (s, 1H), 6.95 (d, *J* = 5.0 Hz, 1H), 6.26 (s, 1H), 5.57 (s, 2H), 4.22 (dtd, *J* = 17.7, 10.8, 7.1 Hz, 1H), 3.67 (s, 2H), 3.33 (q, *J* = 7.4 Hz, 1H), 1.32 (t, *J* = 7.2 Hz, 3H), 1.25 (d, *J* = 6.4 Hz, 3H).

^{13}C NMR (101 MHz, Chloroform-*d*) δ 166.81, 159.94, 144.72, 138.24, 136.95, 128.09, 126.28, 121.56, 103.54, 58.55, 42.64, 27.88, 15.85, 14.77.

HRMS (ESI-TOF) m/z $[M+H]^+$ Calcd for $C_{14}H_{18}NO_2S$ 264.1058; found 264.1070.

$[\alpha]^{25}_D$ = -74.6 (c = 1.42, $CHCl_3$)

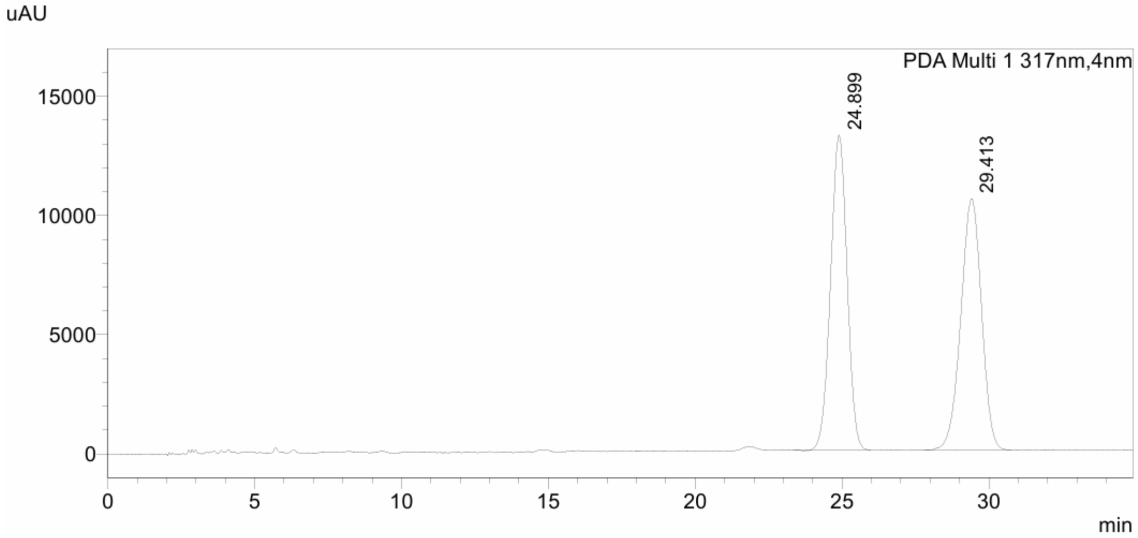

PDA Ch1 317nm

Peak#	Ret. Time	Area	Area%
1	24.899	515513	49.875
2	29.413	518107	50.125
Total		1033620	100.000

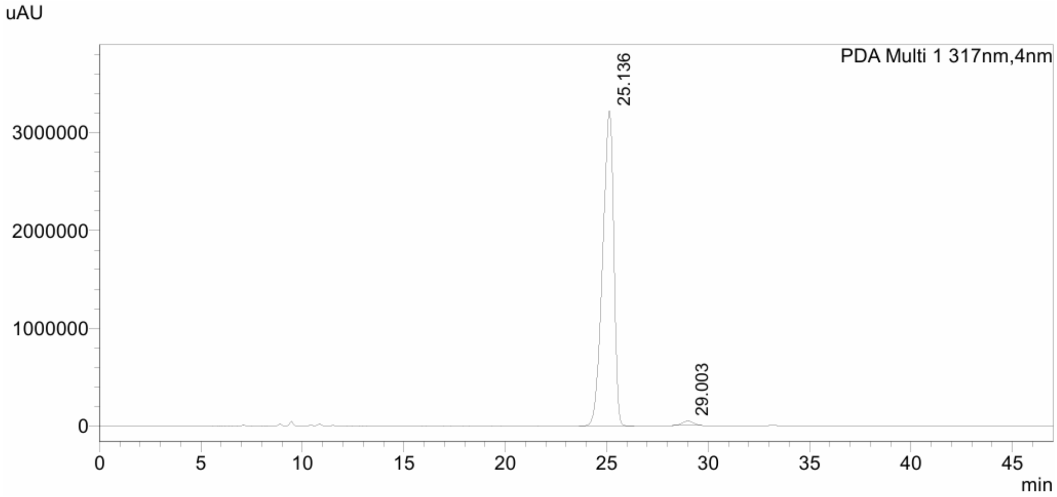

PDA Ch1 317nm

Peak#	Ret. Time	Area	Area%
1	25.136	122924100	98.519
2	29.003	1848144	1.481
Total		124772244	100.000

***Tert*-butyl (*R*)-3-((5-amino-4-(ethoxycarbonyl)-3-methylcyclopenta-1,4-dien-1-yl)methyl)-1H-indole-1-carboxylate (3e)**

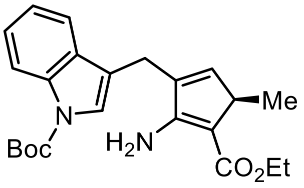

Prepared according to **General Procedure H** using **1e** (77 mg, 0.196 mmol), $Cu(OAc)_2$ (0.71 mg, 3.91 µmol), Josiphos (2.34 mg, 4.31 µmol), TMDSO (69 µL, 0.392 mmol), KF (45.5 mg, 0.784 mmol) and H_2O (7 µL, 0.392 mmol) in THF (0.78 mL). Purification by silica gel flash column chromatography (hexane/EtOAc 9:1) provided the title compound (76 mg, 0.192 mmol, 98% yield, *er*=97.5:2.5) as a yellow solid.

HPLC: Phenomenex cellulose-1, Hexane:2-propanol 90:10, flow: 1.0 mL/min, 342 nm, t_R = 17.3 min (minor) and t_R = 21.7 min (major).

^{1}H NMR (400 MHz, Chloroform-*d*) δ 8.14 (d, *J* = 7.7 Hz, 1H), 7.50 (d, *J* = 7.8 Hz, 1H), 7.35 (t, *J* = 7.8 Hz, 1H), 7.29 – 7.22 (m, 1H), 6.29 (s, 1H), 5.67 (s, 2H), 4.23 (tdd, *J* = 18.3, 9.1, 5.6 Hz, 2H), 3.71 (s, 2H), 3.33 (q, *J* = 7.5 Hz, 1H), 1.69 (s, 9H), 1.33 (t, *J* = 7.1 Hz, 3H), 1.25 (d, *J* = 7.4 Hz, 3H).

^{13}C NMR (101 MHz, Chloroform-*d*) δ 166.81, 149.69, 144.91, 144.90, 135.96, 135.68, 130.11, 124.64, 123.56, 122.57, 119.18, 116.97, 115.37, 103.55, 83.80, 58.54, 42.66, 28.22, 22.86, 15.79, 14.78.

HRMS (ESI-TOF) m/z $[M+H]^+$ Calcd for $C_{23}H_{29}N_2O_4$ 397.2127; found 397.2262.

$[\alpha]^{25}_D$ = -24.5 (c = 1.5, $CHCl_3$).

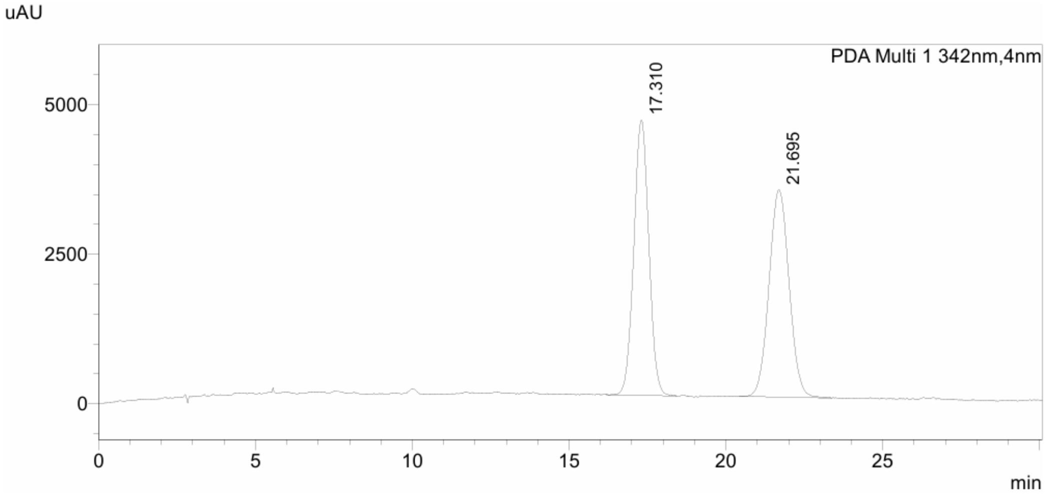

PDA Ch1 342nm

Peak#	Ret. Time	Area	Area%
1	17.310	153351	49.866
2	21.695	154173	50.134
Total		307525	100.000

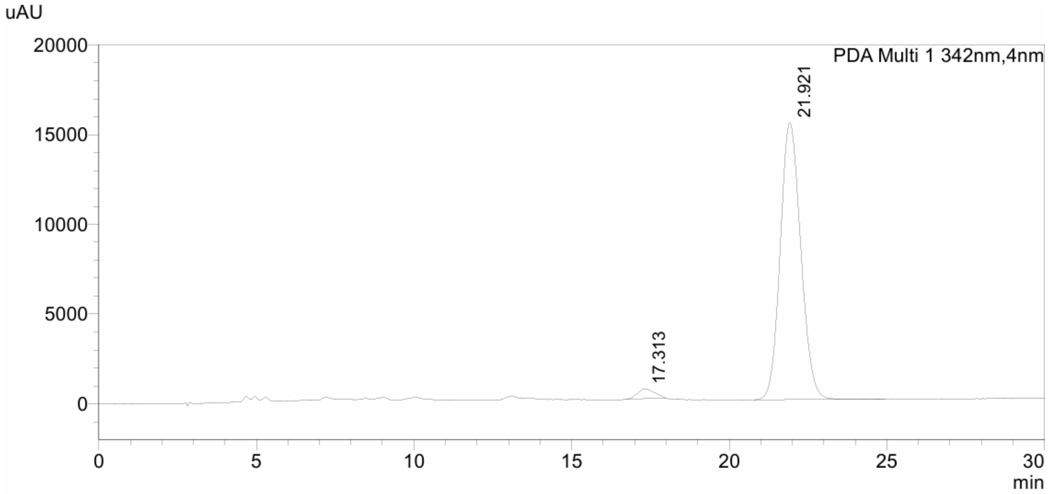

PDA Ch1 342nm

Peak#	Ret. Time	Area	Area%
1	17.313	20964	2.903
2	21.921	701097	97.097
Total		722061	100.000

Ethyl (*R*)-2-amino-3-benzhydryl-5-methylcyclopenta-1,3-diene-1-carboxylate (3f)

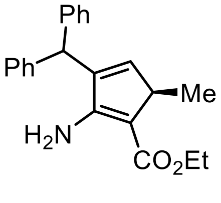

Prepared according to **General Procedure H** using **1f** (65 mg, 0.196 mmol), $Cu(OAc)_2$ (0.71 mg, 3.91 μmol), Josiphos (2.34 mg, 4.31 μmol), TMDSO (69 μL, 0.392 mmol), KF (45.5 mg, 0.784 mmol) and H_2O (7 μL, 0.392 mmol) in THF (0.78 mL). Purification by silica gel flash column chromatography (hexane/EtOAc 9:1) provided the title compound (65 mg, 0.194 mmol, 99% yield, *er*=97.5:2.5) as a pale yellow oil.

HPLC: Phenomenex cellulose-1, Hexane:2-propanol 90:10, flow: 1.0 mL/min, 350 nm, t_R = 6.0 min (minor) and t_R = 9.0 min (major).

^{1}H NMR (300 MHz, Chloroform-*d*) δ 7.40 – 7.25 (m, 6H), 7.25 – 7.14 (m, 4H), 5.88 (s, 1H), 5.41 (s, 2H), 5.05 (s, 1H), 4.30 – 4.11 (m, 2H), 3.33 (q, *J* = 7.4 Hz, 1H), 1.32 (t, *J* = 7.1 Hz, 3H), 1.24 (d, *J* = 7.4 Hz, 3H).

^{13}C NMR (75 MHz, Chloroform-*d*) δ 166.90, 146.44, 141.33, 141.13, 141.03, 128.86, 128.75, 127.01, 103.85, 58.55, 49.50, 42.54, 15.94, 14.77.

HRMS (ESI-TOF) m/z [M+H]$^+$ Calcd for $C_{22}H_{24}NO_2$ 334.1807; found 334.2050.

$[\alpha]^{25}_D$ = -32.6 (c = 1.0, $CHCl_3$).

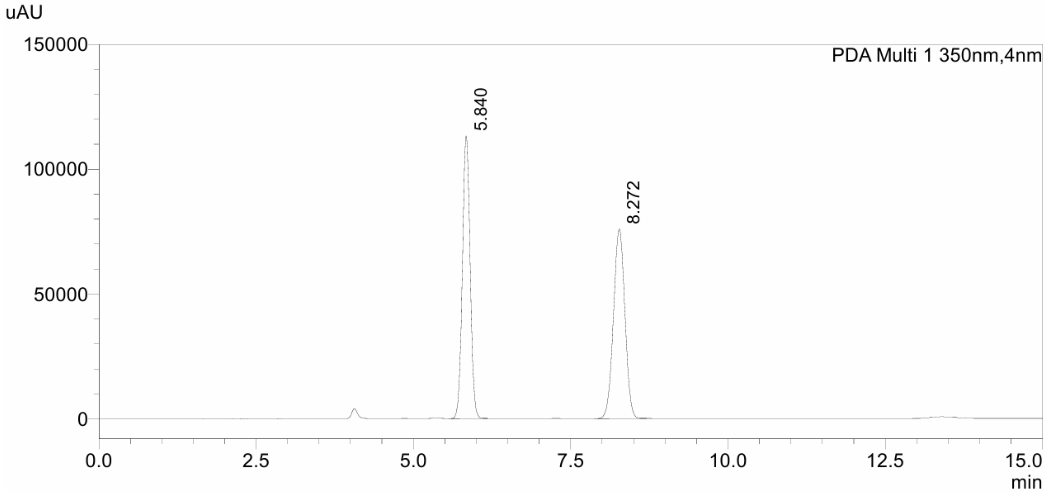

PDA Ch1 350nm

Peak#	Ret. Time	Area	Area%
1	5.840	966948	50.084
2	8.272	963719	49.916
Total		1930667	100.000

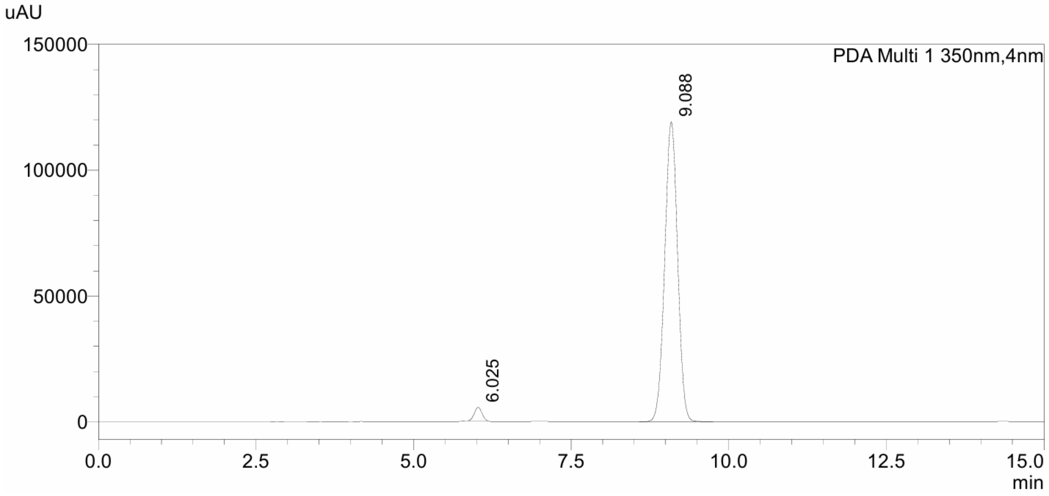

PDA Ch1 350nm

Peak#	Ret. Time	Area	Area%
1	6.025	44709	2.569
2	9.088	1695519	97.431
Total		1740227	100.000

Ethyl (*R*)-2-amino-3-(bis(4-bromophenyl)methyl)-5-methylcyclopenta-1,3-diene-1-carboxylate (3g)

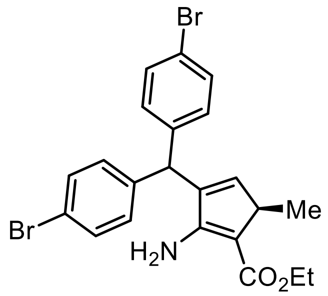

Prepared according to **General Procedure H** using **1g** (96 mg, 0.196 mmol), $Cu(OAc)_2$ (0.71 mg, 3.91 μmol), Josiphos (2.34 mg, 4.31 μmol), TMDSO (69 μL, 0.392 mmol), KF (45.48 mg, 0.784 mmol) and H_2O (7 μL, 0.392 mmol) in THF (0.78 mL). Purification by silica gel flash column chromatography (hexane/EtOAc 9:1) provided the title compound (86 mg, 0.176 mmol, 90% yield, *er*=98.5:1.5) as a transparent oil.

HPLC: Phenomenex cellulose-4, Hexane:2-propanol 90:10, flow: 1.0 mL/min, 332 nm, t_R = 11.1 min (minor) and t_R = 13.6 min (major).

^{1}H NMR (300 MHz, Chloroform-*d*) δ 7.47 (dq, *J* = 9.1, 2.7 Hz, 4H), 7.07 – 6.99 (m, 4H), 5.88 (s, 1H), 5.79 – 5.07 (m, 2H), 4.98 (s, 1H), 4.31 – 4.12 (m, 2H), 3.32 (q, *J* = 7.3 Hz, 1H), 1.32 (t, *J* = 7.1 Hz, 3H), 1.23 (d, *J* = 7.4 Hz, 3H).

^{13}C NMR (75 MHz, Chloroform-*d*) δ 166.73, 159.03, 146.76, 140.44, 139.67, 131.97, 130.54, 121.21, 103.98, 58.66, 48.15, 42.69, 15.98, 14.82.

HRMS (ESI-TOF) m/z $[M+H]^+$ Calcd for $C_{22}H_{22}Br_2NO_2$ 490.0017; found 490.0006.

$[\alpha]^{25}_D$ = -28.1 (c = 1.0, $CHCl_3$).

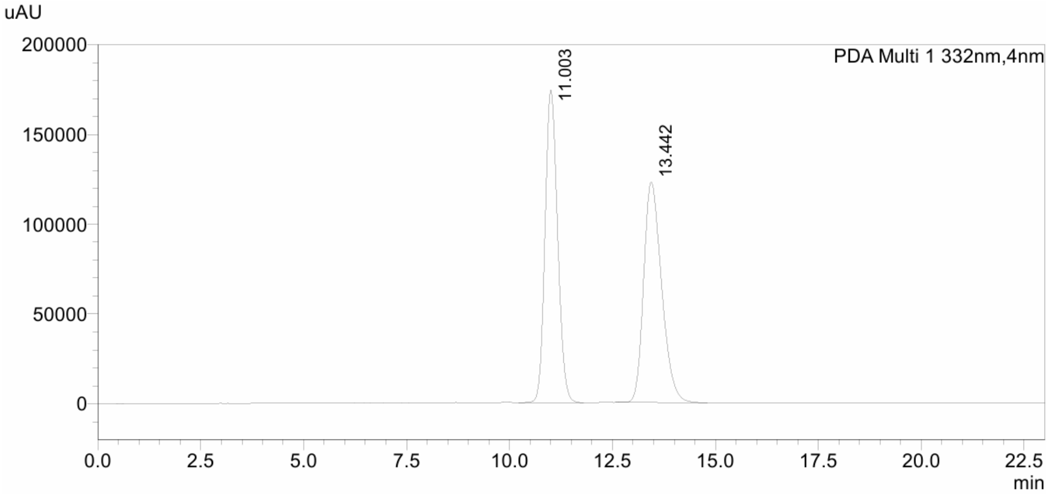

PDA Ch1 332nm

Peak#	Ret. Time	Area	Area%
1	11.003	3690609	49.737
2	13.442	3729598	50.263
Total		7420207	100.000

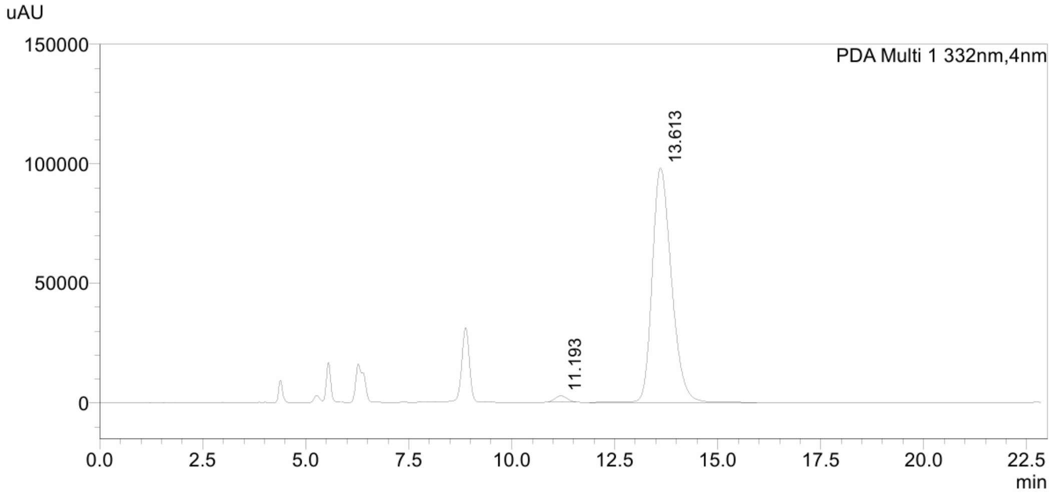

PDA Ch1 332nm

Peak#	Ret. Time	Area	Area%
1	11.193	50380	1.552
2	13.613	3194882	98.448
Total		3245262	100.000

Ethyl (*R*)-2-amino-3-isopropyl-5-methylcyclopenta-1,3-diene-1-carboxylate (3h)

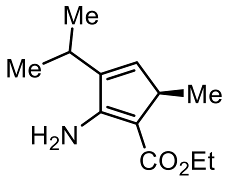

Prepared according to **General Procedure H** using **1h** (41 mg, 0.196 mmol), $Cu(OAc)_2$ (0.71 mg, 3.91 µmol), Josiphos (2.34 mg, 4.31 µmol), TMDSO (69 µL, 0.392 mmol), KF (45.48 mg, 0.784 mmol) and H_2O (7 µL, 0.392 mmol) in THF (0.78 mL). Purification by silica gel flash column chromatography (hexane/EtOAc 9:1) provided the title compound (41 mg, 0.194 mmol, 99% yield, *er*=98.5:1.5) as a transparent oil.

HPLC: Phenomenex cellulose-1, Hexane:2-propanol 90:10, flow: 1.0 mL/min, 349 nm, t_R = 5.1 min (minor) and t_R = 6.7 min (major).

^{1}H NMR (300 MHz, Chloroform-*d*) δ 6.18 (s, 1H), 5.76 (s, 2H), 4.31 – 4.11 (m, 2H), 3.24 (q, *J* = 7.4 Hz, 1H), 2.61 – 2.38 (m, 1H), 1.31 (t, *J* = 7.1 Hz, 3H), 1.18 (q, *J* = 6.5, 6.1 Hz, 9H).

^{13}C NMR (75 MHz, Chloroform-*d*) δ 166.98, 160.09, 145.13, 140.39, 103.10, 58.43, 42.25, 25.29, 21.90, 15.91, 14.77.

HRMS (ESI-TOF) m/z $[M+H]^+$ Calcd for $C_{12}H_{20}NO_2$ 210.1494; found 210.1680.

$[\alpha]^{25}_D$ = -123.1 (c = 1.0, $CHCl_3$).

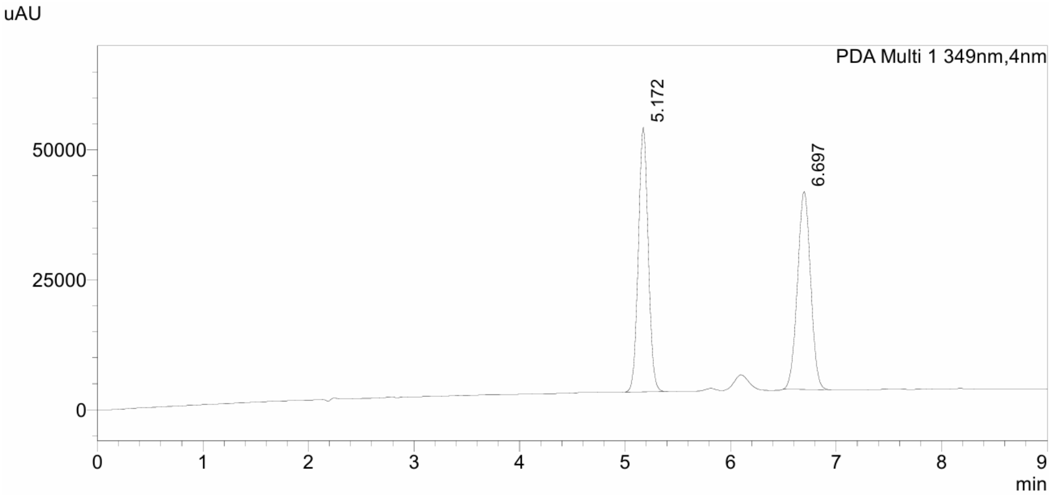

PDA Ch1 349nm

Peak#	Ret. Time	Area	Area%
1	5.172	330370	50.038
2	6.697	329875	49.962
Total		660245	100.000

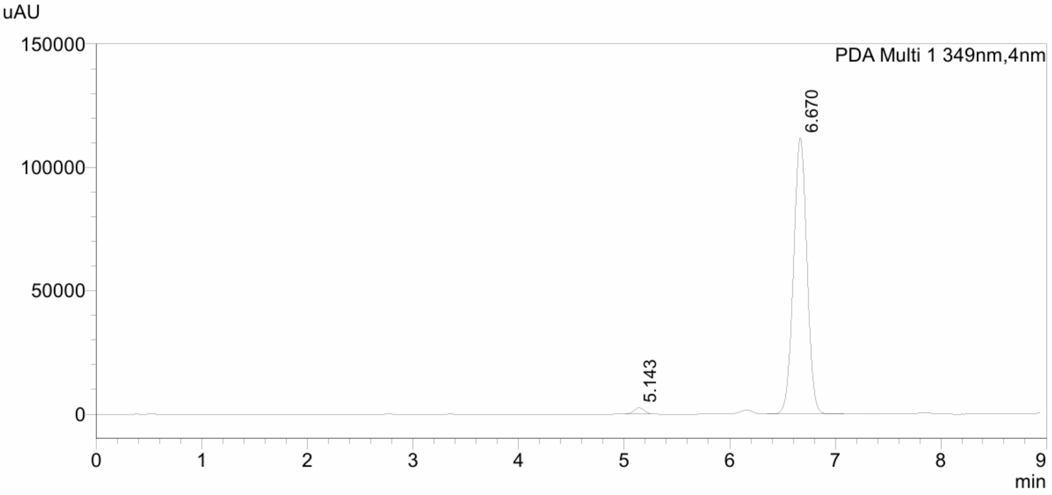

PDA Ch1 349nm

Peak#	Ret. Time	Area	Area%
1	5.143	14626	1.459
2	6.670	987972	98.541
Total		1002598	100.000

Ethyl (*R*)-2-amino-5-methyl-3-(3-phenylpropyl)cyclopenta-1,3-diene-1-carboxylate (3i)

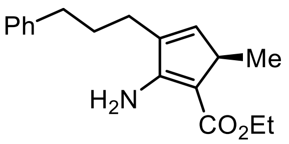

Prepared according to **General Procedure H** using **1i** (56 mg, 0.196 mmol), $Cu(OAc)_2$ (0.71 mg, 3.91 μmol), Josiphos (2.34 mg, 4.31 μmol), TMDSO (69 μL, 0.392 mmol), KF (45.48 mg, 0.784 mmol) and H_2O (7 μL, 0.392 mmol) in THF (0.78 mL). Purification by silica gel flash column chromatography (hexane/EtOAc 9:1) provided the title compound (40 mg, 0.137 mmol, 70% yield, *er*=98:2) as a transparent oil.

HPLC: Phenomenex cellulose-4, Hexane:2-propanol 90:10, flow: 1.0 mL/min, 325 nm, t_R = 7.0 min (minor) and t_R = 7.9 min (major).

^{1}H NMR (400 MHz, Chloroform-*d*) δ 7.34 – 7.30 (m, 2H), 7.24 – 7.20 (m, 3H), 6.23 (d, *J* = 1.8 Hz, 1H), 5.64 (s, 2H), 4.33 – 4.13 (m, 2H), 3.28 (q, *J* = 7.3, 6.4 Hz, 1H), 2.71 (t, *J* = 7.5 Hz, 2H), 2.23 (t, *J* = 7.3 Hz, 2H), 1.94 (p, *J* = 7.6 Hz, 2H), 1.33 (t, *J* = 7.1 Hz, 3H), 1.23 (d, *J* = 7.4 Hz, 3H).

^{13}C NMR (101 MHz, Chloroform-*d*) δ 166.92, 160.28, 142.76, 141.78, 138.30, 128.46, 128.45, 125.99, 102.90, 58.47, 42.60, 35.38, 29.19, 25.23, 15.91, 14.81.

HRMS (ESI-TOF) m/z $[M+H]^+$ Calcd for $C_{18}H_{24}NO_2$ 286.1807; found 286.1879.

$[\alpha]^{25}_D$ = -205.6 (c = 0.49, $CHCl_3$).

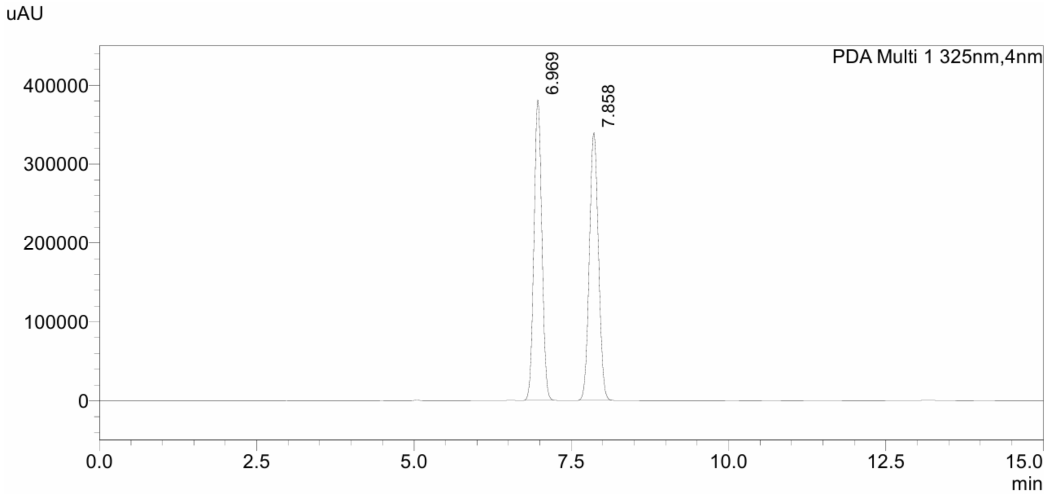

PDA Ch1 325nm

Peak#	Ret. Time	Area	Area%
1	6.969	3315156	49.228
2	7.858	3419155	50.772
Total		6734311	100.000

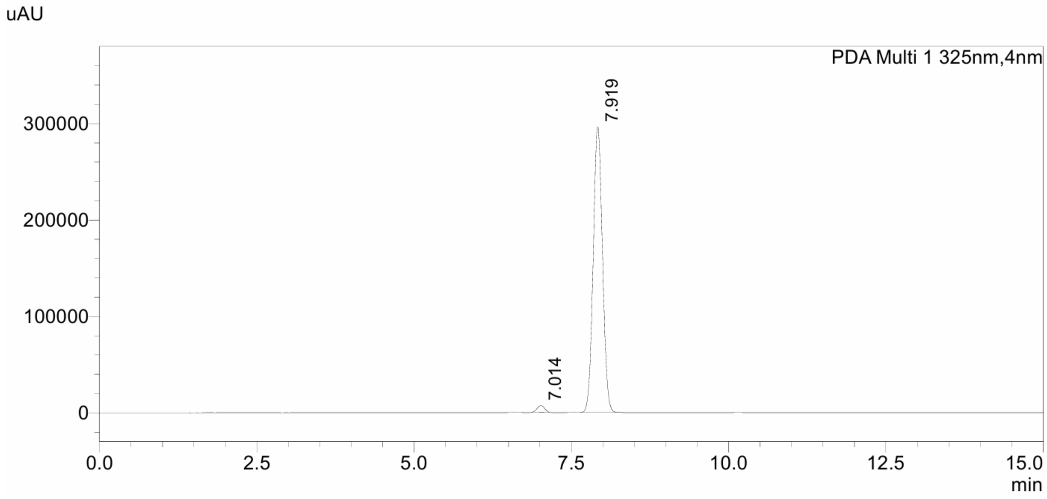

PDA Ch1 325nm

Peak#	Ret. Time	Area	Area%
1	7.014	58809	1.892
2	7.919	3048983	98.108
Total		3107793	100.000

Ethyl (*R*)-3-(2-(1,3-dioxolan-2-yl)ethyl)-2-amino-5-methylcyclopenta-1,3-diene-1-carboxylat (3j)

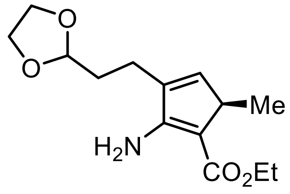

Prepared according to **General Procedure H** using **1j** (48 mg, 0.196 mmol), $Cu(OAc)_2$ (0.71 mg, 3.91 μmol), Josiphos (2.34 mg, 4.31 μmol), TMDSO (69 μL, 0.392 mmol), KF (45.48 mg, 0.784 mmol) and H_2O (7 μL, 0.392 mmol) in THF (0.78 mL). Purification by silica gel flash column chromatography (hexane/EtOAc 7:3) provided the title compound (47 mg, 0.178 mmol, 91% yield, *er*=98:2) as a transparent oil.

HPLC: Phenomenex cellulose-1, Hexane:2-propanol 90:10, flow: 1.0 mL/min, 350 nm, t_R = 14.3 min (minor) and t_R = 19.3 min (major).

^{1}H NMR (400 MHz, Chloroform-*d*) δ 6.21 (s, 1H), 5.84 (s, 2H), 4.93 (t, *J* = 4.6 Hz, 1H), 4.20 (tdd, *J* = 18.0, 8.9, 5.4 Hz, 2H), 4.04 – 3.85 (m, 4H), 3.26 (q, *J* = 7.5 Hz, 1H), 2.35 (t, *J* = 7.8 Hz, 2H), 1.97 – 1.88 (m, 2H), 1.31 (t, *J* = 7.1 Hz, 3H), 1.20 (d, *J* = 7.4 Hz, 3H).

^{13}C NMR (101 MHz, Chloroform-*d*) δ 166.87, 142.86, 138.06, 132.04, 103.49, 102.74, 65.03, 58.41, 42.57, 32.10, 19.99, 15.82, 14.78.

HRMS (ESI-TOF) m/z $[M+H]^+$ Calcd for $C_{14}H_{22}NO_4$ 268.1549; found 268.1786.

$[\alpha]^{25}_D$ = -25.9 (c = 1.0, $CHCl_3$).

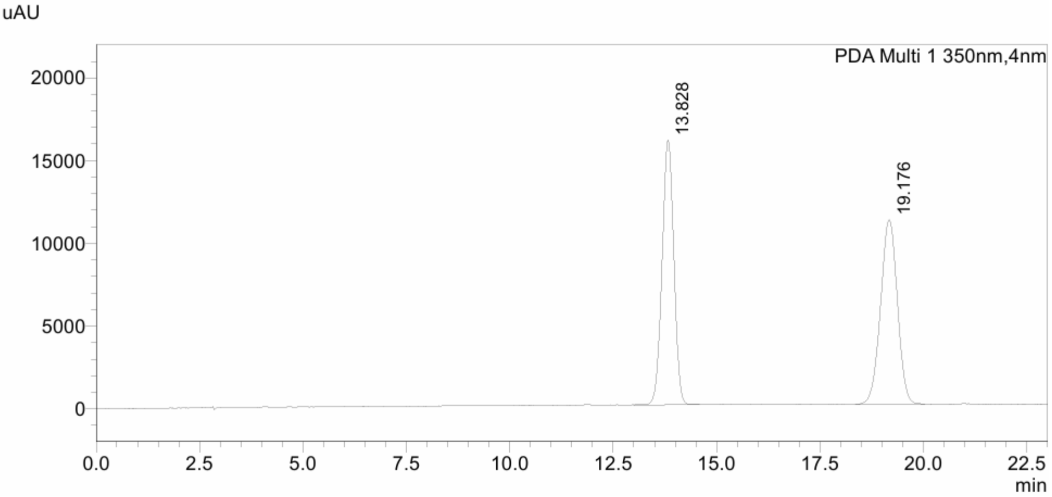

PDA Ch1 350nm

Peak#	Ret. Time	Area	Area%
1	13.828	316424	50.206
2	19.176	313823	49.794
Total		630247	100.000

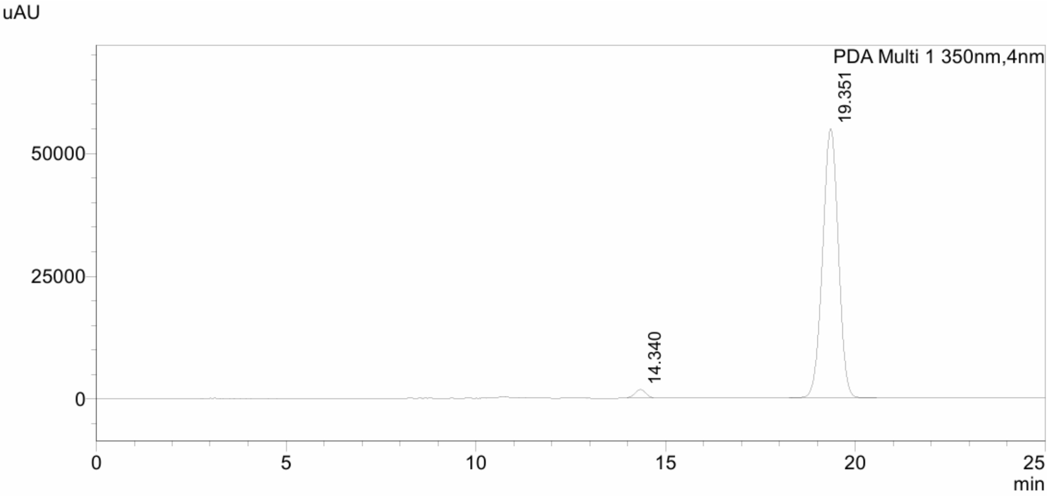

PDA Ch1 350nm

Peak#	Ret. Time	Area	Area%
1	14.340	32231	2.005
2	19.351	1575363	97.995
Total		1607595	100.000

Ethyl (*R*)-2-amino-3-(2-((tert-butyldimethylsilyl)oxy)ethyl)-5-methylcyclopenta-1,3-diene-1-carboxylate (3k)

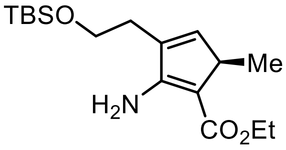

Prepared according to **General Procedure H** using **1k** (63 mg, 0.196 mmol), $Cu(OAc)_2$ (0.71 mg, 3.91 μmol), Josiphos (2.34 mg, 4.31 μmol), TMDSO (69 μL, 0.392 mmol), KF (45.48 mg, 0.784 mmol) and H_2O (7 μL, 0.392 mmol) in THF (0.78 mL). Purification by silica gel flash column chromatography (hexane/EtOAc 9:1) provided the title compound (63 mg, 0.194 mmol, 99% yield, *er*=98:1.5) as a transparent oil.

HPLC: Phenomenex cellulose-1, Hexane:2-propanol 99:1, flow: 1.0 mL/min, 350 nm, t_R = 15.6 min (major) and t_R = 17.2 min (minor).

^{1}H NMR (400 MHz, Chloroform-*d*) δ 6.22 (s, 1H), 6.14 (s, 2H), 4.33 – 4.12 (m, 2H), 3.79 (hept, *J* = 5.9, 4.9 Hz, 2H), 3.28 (q, *J* = 7.5 Hz, 1H), 2.52 (t, *J* = 5.7 Hz, 2H), 1.32 (t, *J* = 7.1 Hz, 3H), 1.22 (d, *J* = 7.4 Hz, 3H), 0.91 (s, 9H), 0.07 (s, 6H).

^{13}C NMR (101 MHz, Chloroform-*d*) δ 166.84, 161.38, 144.67, 137.87, 102.52, 63.31, 58.33, 42.69, 30.68, 25.92, 18.32, 15.85, 14.81, -5.49.

HRMS (ESI-TOF) m/z $[M+H]^+$ Calcd for $C_{17}H_{32}NO_3Si$ 326.2151; found 326.2693.

$[\alpha]^{25}_D$ = -38.3 (c = 0.68, $CHCl_3$).

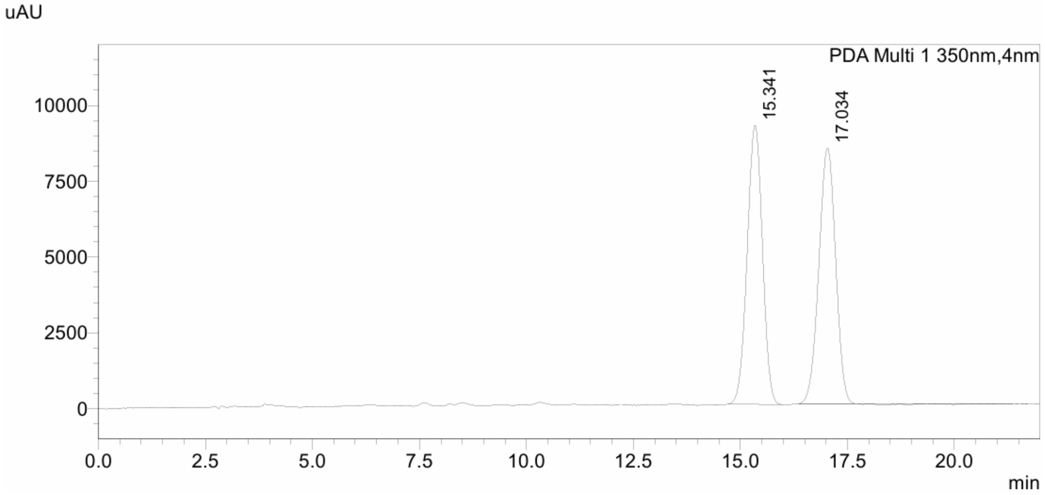

PDA Ch1 350nm

Peak#	Ret. Time	Area	Area%
1	15.341	228036	49.877
2	17.034	229162	50.123
Total		457197	100.000

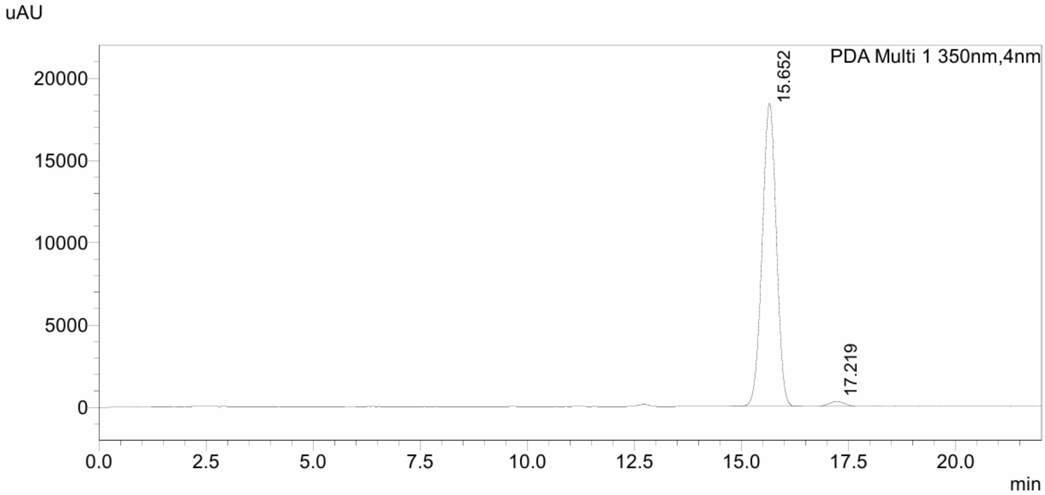

PDA Ch1 350nm

Peak#	Ret. Time	Area	Area%
1	15.652	423691	98.428
2	17.219	6769	1.572
Total		430460	100.000

Ethyl (*R*)-3-allyl-2-amino-5-methylcyclopenta-1,3-diene-1-carboxylate (3l)

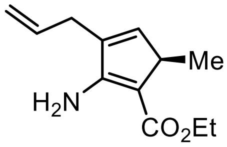

Prepared according to **General Procedure H** using **1l** (40 mg, 0.196 mmol), $Cu(OAc)_2$ (0.71 mg, 3.91 µmol), Josiphos (2.34 mg, 4.31 µmol), TMDSO (69 µL, 0.392 mmol), KF (45.48 mg, 0.784 mmol) and H_2O (7 µL, 0.392 mmol) in THF (0.78 mL). Purification by silica gel flash column chromatography (hexane/EtOAc 9:1) provided the title compound (37 mg, 0.180 mmol, 92% yield, *er*=99:1) as a pale yellow oil.

HPLC: Phenomenex cellulose-1, Hexane:2-propanol 90:10, flow: 1.0 mL/min, 350 nm, t_R = 7.5 min (major) and t_R = 7.9 min (minor).

^{1}H NMR (400 MHz, Chloroform-*d*) δ 6.25 (s, 1H), 5.91 (ddt, *J* = 16.6, 10.1, 6.3 Hz, 1H), 5.70 (s, 2H), 5.24 – 5.09 (m, 2H), 4.31 – 4.12 (m, 2H), 3.29 (q, *J* = 7.3 Hz, 1H), 3.06 (d, *J* = 6.1 Hz, 2H), 1.32 (t, *J* = 7.1 Hz, 3H), 1.23 (d, *J* = 7.5 Hz, 3H).

^{13}C NMR (101 MHz, Chloroform-*d*) δ 166.83, 160.18, 144.31, 136.22, 134.76, 117.07, 103.25, 58.47, 42.63, 31.12, 15.80, 14.77.

HRMS (ESI-TOF) m/z $[M+H]^+$ Calcd for $C_{12}H_{18}NO_2$ 208.1338; found 208.1327.

$[\alpha]^{25}_D$ = -369.4 (c = 0.27, $CHCl_3$).

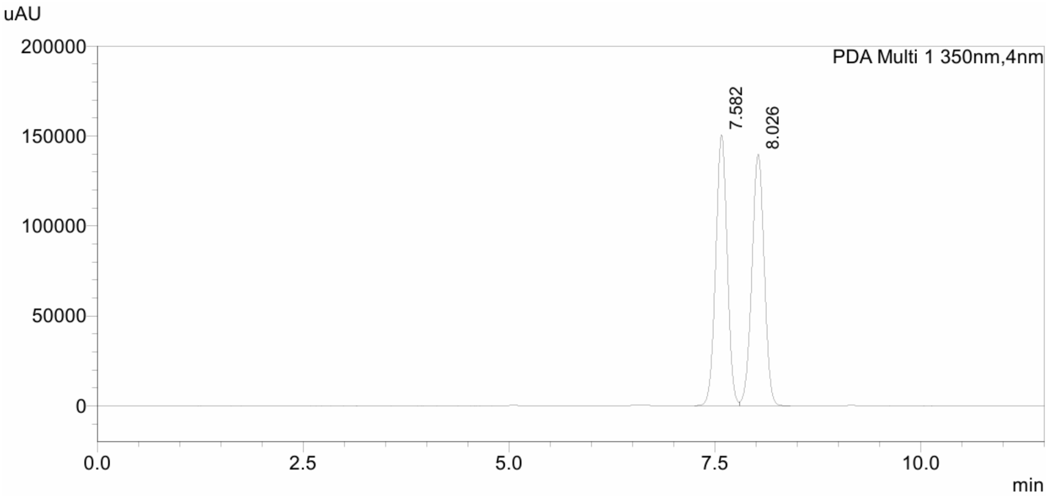

PDA Ch1 350nm

Peak#	Ret. Time	Area	Area%
1	7.582	1460871	50.099
2	8.026	1455122	49.901
Total		2915993	100.000

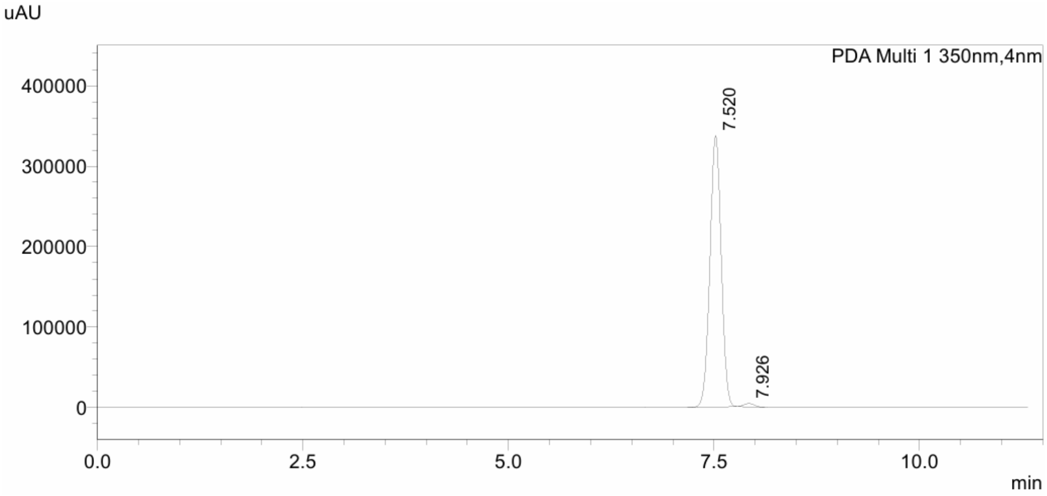

PDA Ch1 350nm

Peak#	Ret. Time	Area	Area%
1	7.520	3220434	98.899
2	7.926	35841	1.101
Total		3256275	100.000

Ethyl (*R*)-2-amino-3,5-dimethylcyclopenta-1,3-diene-1-carboxylate (3m)

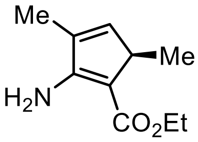

Prepared according to **General Procedure H** using **1m** (35 mg, 0.196 mmol), $Cu(OAc)_2$ (0.71 mg, 3.91 µmol), Josiphos (2.34 mg, 4.31 µmol), TMDSO (69 µL, 0.392 mmol), KF (45.48 mg, 0.784 mmol) and H_2O (7 µL, 0.392 mmol) in THF (0.78 mL). Purification by silica gel flash column chromatography (hexane/EtOAc 9:1) provided the title compound (35 mg, 0.194 mmol, 99% yield, *er*=99:1) as a transparent oil.

HPLC: Phenomenex cellulose-1, Hexane:2-propanol 90:10, flow: 1.0 mL/min, 317 nm, t_R = 9.6 min (major) and t_R = 13.2 min (minor).

^{1}H NMR (400 MHz, Chloroform-*d*) δ 6.21 (s, 1H), 5.67 (s, 2H), 4.30 – 4.13 (m, 2H), 3.25 (q, *J* = 7.5 Hz, 1H), 1.90 (s, 3H), 1.32 (t, *J* = 7.1 Hz, 3H), 1.21 (d, *J* = 7.5 Hz, 3H).

^{13}C NMR (101 MHz, Chloroform-*d*) δ 166.83, 160.55, 143.72, 134.02, 102.70, 58.43, 42.54, 15.74, 14.78, 11.54.

HRMS (ESI-TOF) m/z $[M+H]^+$ Calcd for $C_{10}H_{16}NO_2$ 182.1181; found 182.1176.

$[\alpha]^{25}_D$ = -94.8 (c = 1.5, $CHCl_3$).

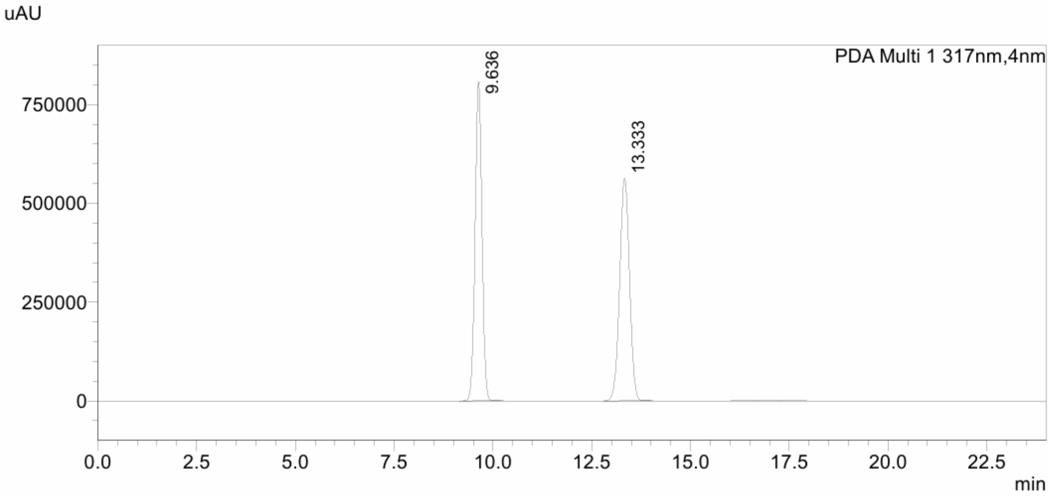

PDA Ch1 317nm

Peak#	Ret. Time	Area	Area%
1	9.636	9756297	49.994
2	13.333	9758658	50.006
Total		19514955	100.000

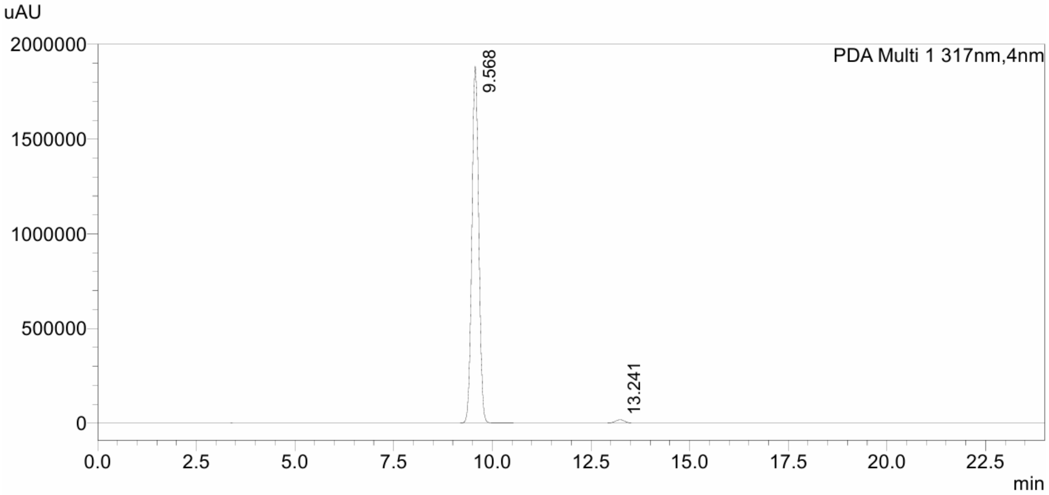

PDA Ch1 317nm

Peak#	Ret. Time	Area	Area%
1	9.568	22749847	99.000
2	13.241	229822	1.000
Total		22979669	100.000

Ethyl (*R*)-2-amino-5-methyl-3-phenylcyclopenta-1,3-diene-1-carboxylate (3n)

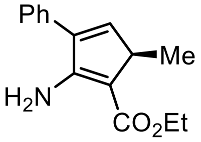

Prepared according to **General Procedure H** using **1n** (47 mg, 0.196 mmol), $Cu(OAc)_2$ (0.71 mg, 3.91 μmol), Josiphos (2.34 mg, 4.31 μmol), TMDSO (69 μL, 0.392 mmol), KF (45.48 mg, 0.784 mmol) and H_2O (7 μL, 0.392 mmol) in THF (0.78 mL). Purification by silica gel flash column chromatography (hexane/EtOAc 9:1) provided the title compound (34 mg, 0.139 mmol, 71% yield, *er*=98.5:1.5) as a pale yellow oil.

HPLC: Phenomenex cellulose-3, Hexane:2-propanol 90:10, flow: 1.0 mL/min, 350 nm, t_R = 5.5 min (major) and t_R = 6.0 min (minor).

^{1}H NMR (300 MHz, Chloroform-*d*) δ 7.46 – 7.36 (m, 5H), 6.52 (s, 1H), 5.85 (s, 2H), 4.34 – 4.17 (m, 2H), 3.47 (q, *J* = 7.4 Hz, 1H), 1.35 (t, *J* = 7.1 Hz, 6H).

^{13}C NMR (101 MHz, Chloroform-*d*) δ 166.97, 145.47, 139.88, 133.74, 129.20, 129.01, 128.13, 127.75, 103.09, 58.62, 43.10, 15.82, 14.81.

HRMS (ESI-TOF) m/z $[M+H]^+$ Calcd for $C_{15}H_{18}NO_2$ 244.1338; found 244.1346.

$[\alpha]^{25}_D$ = -46.4 (c = 1.5, $CHCl_3$).

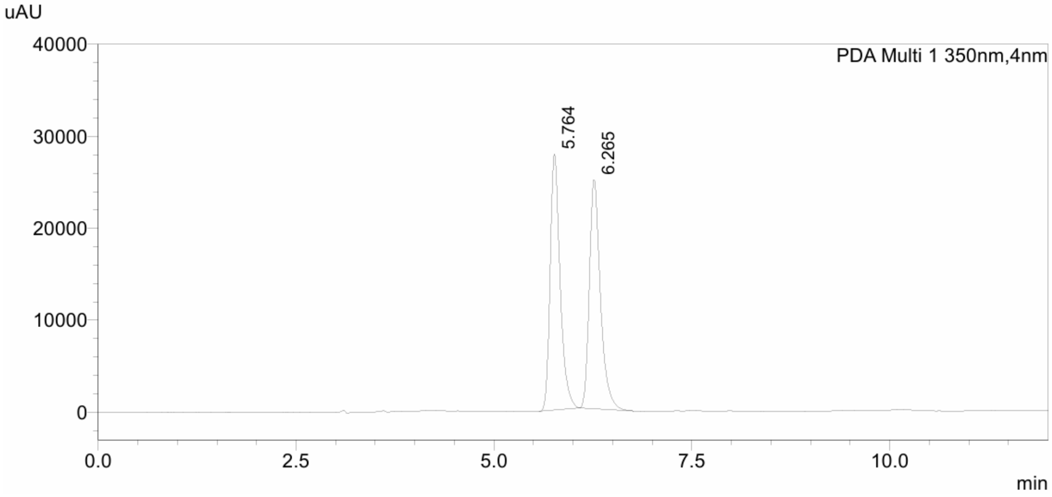

PDA Ch1 350nm

Peak#	Ret. Time	Area	Area%
1	5.764	243585	50.164
2	6.265	241995	49.836
Total		485580	100.000

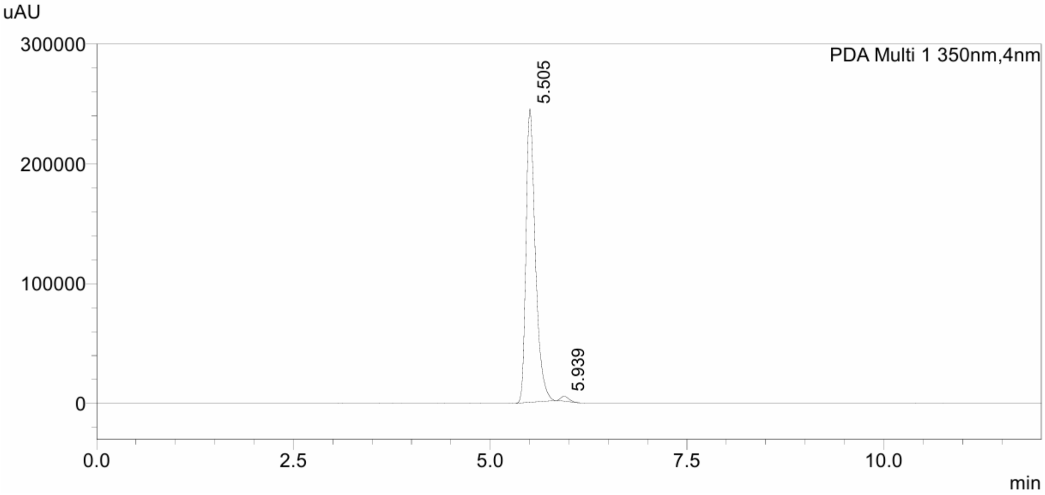

PDA Ch1 350nm

Peak#	Ret. Time	Area	Area%
1	5.505	2030492	98.670
2	5.939	27366	1.330
Total		2057859	100.000

Ethyl (*R*)-2-amino-3-(4-methoxyphenyl)-5-methylcyclopenta-1,3-diene-1-carboxylate (3o)

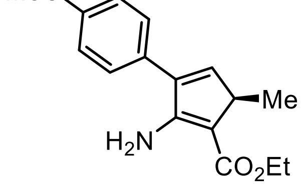

Prepared according to **General Procedure H** using **1o** (53 mg, 0.196 mmol), $Cu(OAc)_2$ (0.71 mg, 3.91 μmol), Josiphos (2.34 mg, 4.31 μmol), TMDSO (69 μL, 0.392 mmol), KF (45.48 mg, 0.784 mmol) and H_2O (7 μL, 0.392 mmol) in THF (0.78 mL). Purification by silica gel flash column chromatography (hexane/EtOAc 9:1) provided the title compound (51 mg, 0.186 mmol, 95% yield, *er*=99:1) as a pale yellow oil.

HPLC: Phenomenex cellulose-1, Hexane:2-propanol 96:4, flow: 1.0 mL/min, 335 nm, t_R = 12.8 min (minor) and t_R = 13.7 min (major).

^{1}H NMR (400 MHz, Chloroform-*d*) δ 7.33 (d, *J* = 7.6 Hz, 2H), 6.97 (d, *J* = 7.6 Hz, 2H), 6.46 (s, 1H), 5.84 (s, 2H), 4.36 – 4.15 (m, 2H), 3.85 (s, 3H), 3.44 (q, *J* = 7.4 Hz, 1H), 1.44 – 1.22 (m, 6H).

^{13}C NMR (101 MHz, Chloroform-*d*) δ 166.97, 159.54, 159.27, 144.62, 139.42, 128.96, 126.08, 114.42, 102.99, 58.58, 55.35, 42.94, 15.87, 14.81.

HRMS (ESI-TOF) m/z $[M+H]^+$ Calcd for $C_{16}H_{20}NO_3$ 274.1443; found 274.1449.

$[\alpha]^{25}_D$ = -173.9 (c = 0.3, $CHCl_3$).

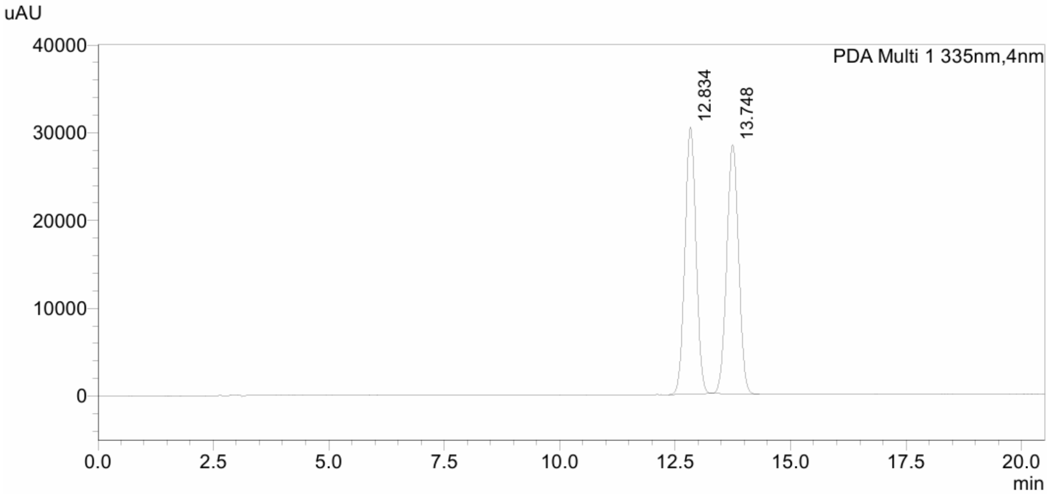

PDA Ch1 335nm

Peak#	Ret. Time	Area	Area%
1	12.834	508794	49.996
2	13.748	508869	50.004
Total		1017663	100.000

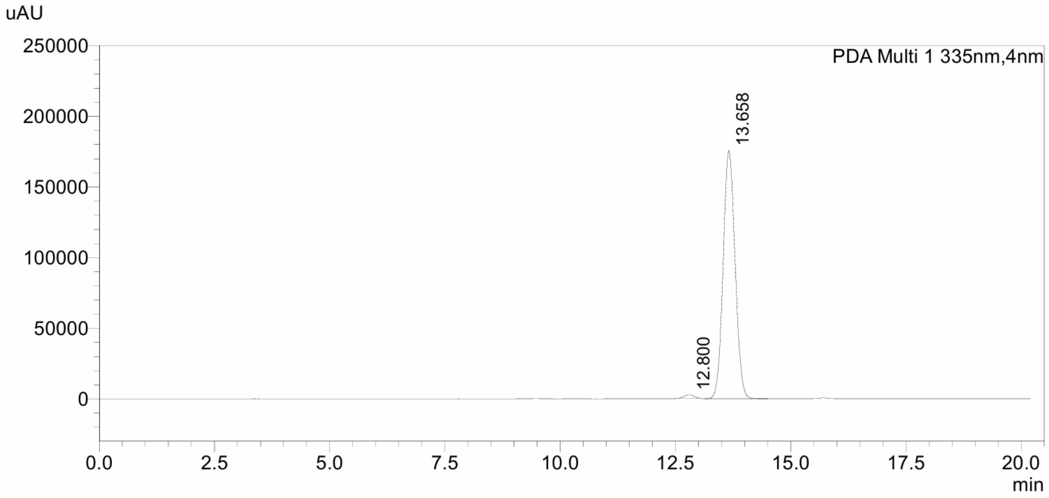

PDA Ch1 335nm

Peak#	Ret. Time	Area	Area%
1	12.800	38216	1.191
2	13.658	3171434	98.809
Total		3209650	100.000

Ethyl (*R*)-2-amino-5-methyl-3-(m-tolyl)cyclopenta-1,3-diene-1-carboxylate (3p)

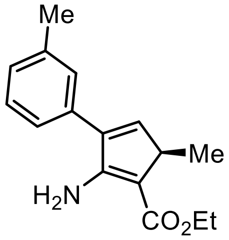

Prepared according to **General Procedure H** using **1p** (50 mg, 0.196 mmol), $Cu(OAc)_2$ (0.71 mg, 3.91 μmol), Josiphos (2.34 mg, 4.31 μmol), TMDSO (69 μL, 0.392 mmol), KF (45.48 mg, 0.784 mmol) and H_2O (7 μL, 0.392 mmol) in THF (0.78 mL). Purification by silica gel flash column chromatography (hexane/EtOAc 9:1) provided the title compound (37 mg, 0.145 mmol, 74% yield, *er*=97.5:2.5) as a transparent oil.

HPLC: Phenomenex cellulose-1, Hexane:2-propanol 96:4, flow: 1.0 mL/min, 261 nm, t_R = 6.8 min (minor) and t_R = 7.6 min (major).

^{1}H NMR (400 MHz, Chloroform-*d*) δ 7.33 (t, *J* = 7.5 Hz, 1H), 7.20 (d, *J* = 7.9 Hz, 3H), 6.51 (s, 1H), 5.86 (s, 2H), 4.34 – 4.17 (m, 2H), 3.46 (q, *J* = 7.4 Hz, 1H), 2.41 (s, 3H), 1.41 – 1.30 (m, 6H).

^{13}C NMR (101 MHz, Chloroform-*d*) δ 166.96, 158.95, 145.35, 145.23, 139.97, 138.75, 133.67, 128.92, 128.41, 124.68, 102.99, 58.58, 43.03, 21.51, 15.89, 14.76.

HRMS (ESI-TOF) m/z [M+Na]$^+$ Calcd for $C_{16}H_{19}NO_2Na$ 280.1313; found 280.1319.

$[\alpha]^{25}_D$ = -46.2 (c = 1.7, $CHCl_3$).

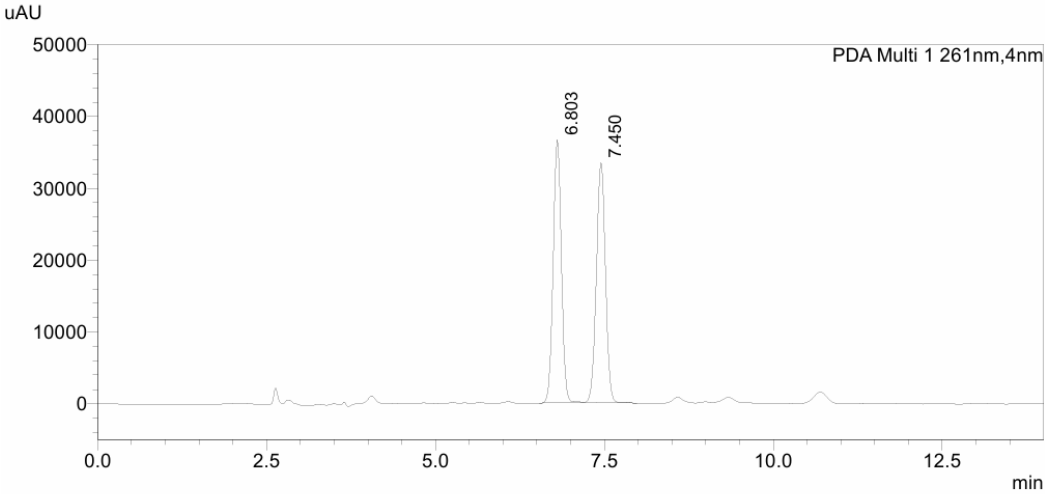

PDA Ch1 261nm

Peak#	Ret. Time	Area	Area%
1	6.803	310647	49.877
2	7.450	312177	50.123
Total		622824	100.000

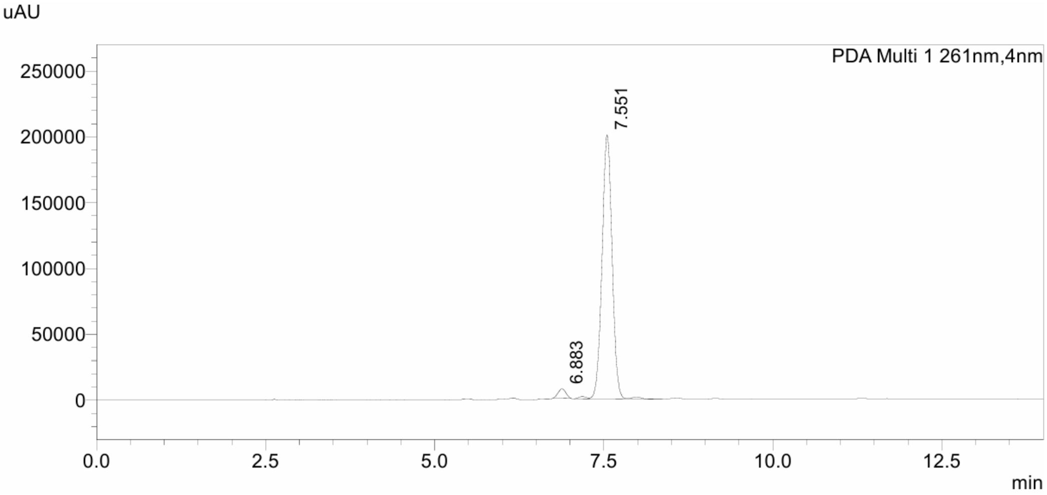

PDA Ch1 261nm

Peak#	Ret. Time	Area	Area%
1	6.883	57675	2.672
2	7.551	2100609	97.328
Total		2158284	100.000

Ethyl (*R*)-2-amino-3-fluoro-5-methylcyclopenta-1,3-diene-1-carboxylate (3q)

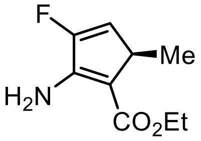

Prepared according to **General Procedure H** using **1q** (36 mg, 0.196 mmol), $Cu(OAc)_2$ (0.71 mg, 3.91 μmol), Josiphos (2.34 mg, 4.31 μmol), TMDSO (69 μL, 0.392 mmol), KF (45.48 mg, 0.784 mmol) and H_2O (7 μL, 0.392 mmol) in THF (0.78 mL). Purification by silica gel flash column chromatography (hexane/EtOAc 9:1) provided the title compound (17 mg, 0.090 mmol, 46% yield, *er*=98.5:1.5) as a transparent oil.

HPLC: Phenomenex cellulose-1, Hexane:2-propanol 96:4, flow: 1.0 mL/min, 324 nm, t_R = 8.3 min (major) and t_R = 8.7 min (minor).

^{1}H NMR (400 MHz, Chloroform-*d*) δ 5.76 (s, 1H), 5.54 (s, 2H), 4.31 – 4.16 (m, 2H), 3.26 (t, *J* = 6.9 Hz, 1H), 1.33 (t, *J* = 7.1 Hz, 3H), 1.27 (d, *J* = 7.3 Hz, 3H).

^{13}C NMR (101 MHz, Chloroform-*d*) δ 166.02, 153.82 (d, *J* = 273.5 Hz), 117.90, 117.87, 102.66, 58.91, 37.41, 15.85, 14.69.

^{19}F NMR (377 MHz, Chloroform-*d*) δ -137.91.

HRMS (ESI-TOF) m/z $[M+H]^+$ Calcd for $C_9H_{13}FNO_2$ 186.0930; found 186.0923.

$[\alpha]^{25}_D$ = -11.0 (c = 0.4, $CHCl_3$).

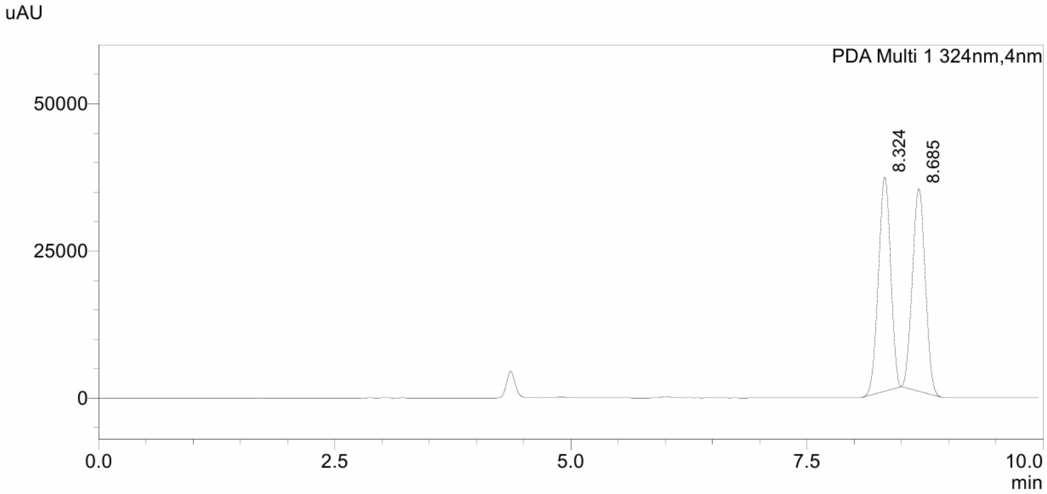

PDA Ch1 324nm

Peak#	Ret. Time	Area	Area%
1	8.324	330374	50.231
2	8.685	327340	49.769
Total		657714	100.000

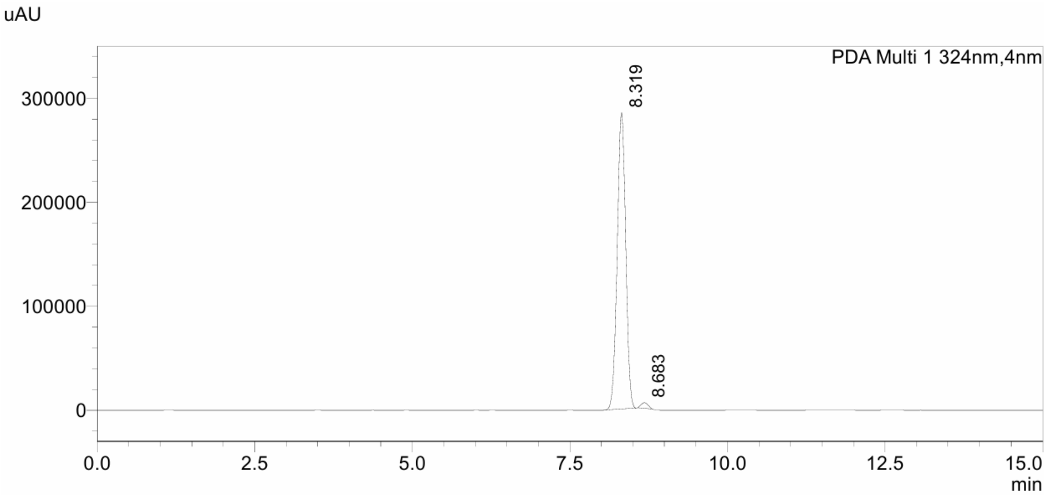

PDA Ch1 324nm

Peak#	Ret. Time	Area	Area%
1	8.319	2669943	98.456
2	8.683	41858	1.544
Total		2711801	100.000

Ethyl (*S*)-2-amino-5-methyl-3-(methylthio)cyclopenta-1,3-diene-1-carboxylate (3r)

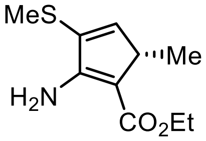

Prepared according to **General Procedure H** using **1r** (34 mg, 0.161 mmol), $Cu(OAc)_2$ (1.20 mg, 3.22 µmol), (*S*,*S*)-Josiphos (1.92 mg, 3.54 µmol), TMDSO (46.6 µL, 0.264 mmol), KF (28.12 mg, 0.528 mmol) and H_2O (2.4 µL, 0.264 mmol) in THF (0.65 mL). Purification by silica gel flash column chromatography (hexane/EtOAc 8:2) provided the title compound (28 mg, 0.129 mmol, 80% yield, *er*=99:1) as a yellow oil.

HPLC: Phenomenex cellulose-1, Hexane:2-propanol 90:10, flow: 1.0 mL/min, 272 nm, t_R = 23.9 min (major) and t_R = 29.2 min (minor).

^{1}H NMR (400 MHz, Chloroform-*d*) δ 6.27 (d, *J* = 1.8 Hz, 1H), 5.73 (br s, 2H), 4.22 (d, *J* = 7.1 Hz, 2H), 3.36 (qd, *J* = 7.4, 1.8 Hz, 1H), 2.37 (s, 3H), 1.31 (t, *J* = 7.1 Hz, 3H), 1.24 (d, *J* = 7.4 Hz, 3H).

^{13}C NMR (101 MHz, Chloroform-*d*) δ 167.20, 150.91, 122.47, 116.32, 114.94, 59.68, 45.19, 17.45, 14.51, 12.88.

HRMS (ESI-TOF) m/z $[M+H]^+$ Calcd for $C_{10}H_{16}NO_2S$ 214.0902; found 214.0894.

$[\alpha]^{25}_D$ = -83.7 (c = 0.1, $CHCl_3$).

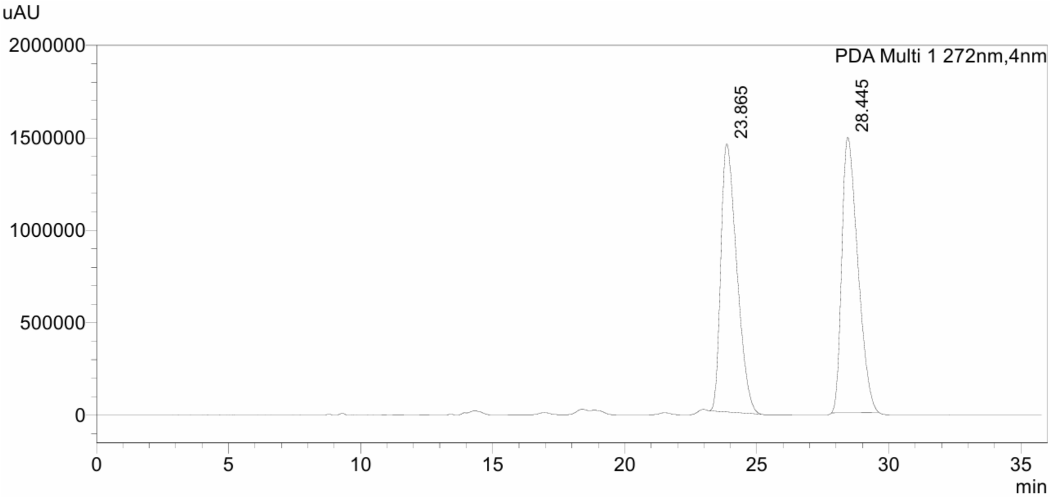

PDA Ch1 272nm

Peak#	Ret. Time	Area	Area%
1	23.865	61125112	49.248
2	28.445	62990839	50.752
Total		124115951	100.000

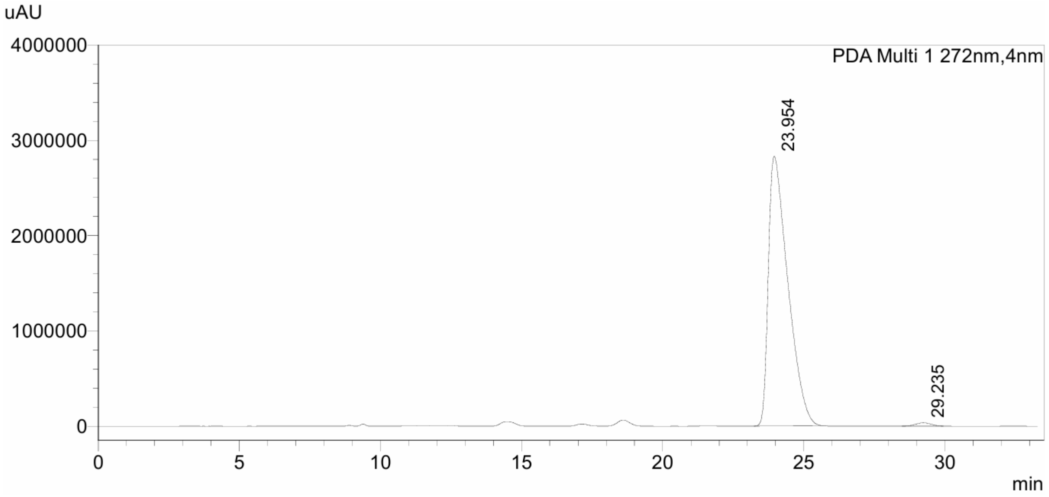

PDA Ch1 272nm

Peak#	Ret. Time	Area	Area%
1	23.954	136268708	99.031
2	29.235	1333219	0.969
Total		137601927	100.000

Methyl (*S*)-2-amino-3-benzyl-5-ethylcyclopenta-1,3-diene-1-carboxylate (3s)

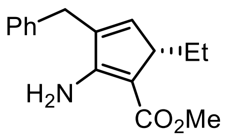

Prepared according to **General Procedure H** using **1s** (34 mg, 0.133 mmol), $Cu(OAc)_2$ (0.48 mg, 2.66 µmol), Josiphos (1.59 mg, 2.93 µmol), TMDSO (47.1 µL, 0.266 mmol), KF (30.91 mg, 0.533 mmol) and H_2O (4.8 µL, 0.266 mmol) in THF (0.53 mL). Purification by silica gel flash column chromatography (hexane/EtOAc 8:2) provided the title compound (33 mg, 0.126 mmol, 95% yield, *er*=99:1) as a pale yellow oil.

HPLC: Phenomenex cellulose-1, Hexane:2-propanol 90:10, flow: 1.0 mL/min, 300 nm, t_R = 15.4 min (minor) and t_R = 18.4 min (major).

^{1}H NMR (400 MHz, Chloroform-*d*) δ 7.32 – 7.27 (m, 2H), 7.24 – 7.17 (m, 3H), 6.29 (d, *J* = 1.3 Hz, 1H), 5.54 (br s, 2H), 3.69 (s, 3H), 3.64 (s, 2H), 3.24 (ddd, *J* = 7.2, 4.0, 1.3 Hz, 1H), 2.08 – 1.98 (m, 1H), 1.46 (ddq, *J* = 13.3, 8.2, 7.4 Hz, 1H), 0.82 (t, *J* = 7.4 Hz, 3H).

^{13}C NMR (101 MHz, Chloroform-*d*) δ 167.27, 160.85, 143.27, 138.39, 137.96, 128.95, 128.66, 126.87, 101.42, 50.16, 49.24, 33.36, 23.48, 11.05.

HRMS (ESI-TOF) m/z $[M+H]^+$ Calcd for $C_{16}H_{20}NO_2$ 258.1494; found 258.1500.

$[\alpha]^{25}_D$ = +17.1 (c = 0.1, $CHCl_3$).

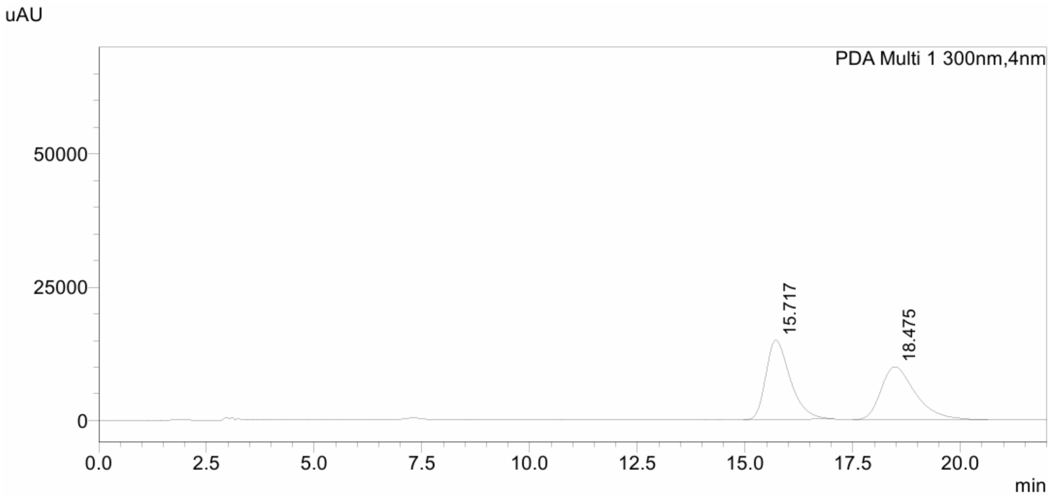

PDA Ch1 300nm

Peak#	Ret. Time	Area	Area%
1	15.717	585216	51.578
2	18.475	549410	48.422
Total		1134626	100.000

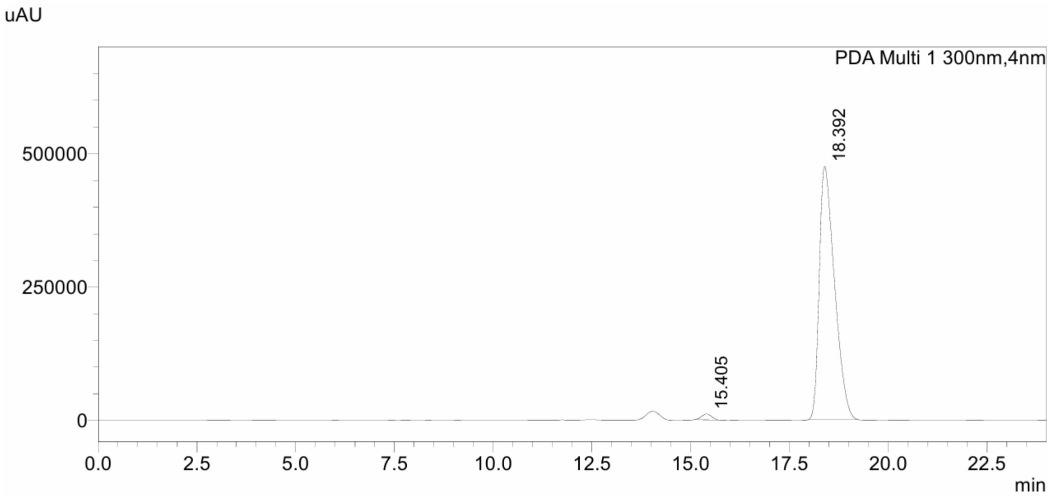

PDA Ch1 300nm

Peak#	Ret. Time	Area	Area%
1	15.405	198205	1.528
2	18.392	12776565	98.472
Total		12974770	100.000

Methyl (*R*)-2-amino-3-benzyl-5-isopropylcyclopenta-1,3-diene-1-carboxylate (3t)

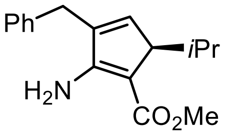

Prepared according to **General Procedure H** using **1t** (35 mg, 0.130 mmol), $Cu(OAc)_2$ (1.18 mg, 6.50 µmol), (*S*,*S*)-Ph-BPE (3.62 mg, 7.15 µmol), TMDSO (45.9 µL, 0.260 mmol), KF (30.24 mg, 0.520 mmol) and H_2O (23.4 µL, 1.30 mmol) in THF (1.30 mL). Purification by silica gel flash column chromatography (hexane/EtOAc 8:2) provided the title compound (30 mg, 0.110 mmol, 85% yield, *er*=99:1) as a yellow oil. The (*R*)-enantiomer was obtained since the starting material **1t** bears a *Z*,*Z* configuration of the double bonds, in contrast to all other compounds **1**.

HPLC: Phenomenex cellulose-1, Hexane:2-propanol 94:06, flow: 1.0 mL/min, 350 nm, t_R = 24.2 min (major) and t_R = 25.7 min (minor).

^{1}H NMR (400 MHz, Chloroform-*d*) δ 7.33 – 7.29 (m, 2H), 7.25 – 7.19 (m, 3H), 6.29 (d, *J* = 1.2 Hz, 1H), 5.59 (br s, 2H), 3.71 (s, 3H), 3.67 (s, 2H), 3.30 (dd, *J* = 4.0, 1.2 Hz, 1H), 2.59 (qd, *J* = 6.9, 4.0 Hz, 1H), 1.13 (d, *J* = 6.9 Hz, 3H) , 0.57 (d, *J* = 6.9 Hz, 3H).

^{13}C NMR (101 MHz, Chloroform-*d*) δ 167.21, 161.07, 140.62, 139.32, 137.94, 128.85, 128.55, 126.75, 100.70, 54.30, 50.07, 33.38, 28.32, 22.43, 16.16.

HRMS (ESI-TOF) m/z $[M+H]^+$ Calcd for $C_{17}H_{22}NO_2$ 272.1651; found 272.1653.

$[\alpha]^{25}_D$ = +217.0 (c = 0.1, $CHCl_3$).

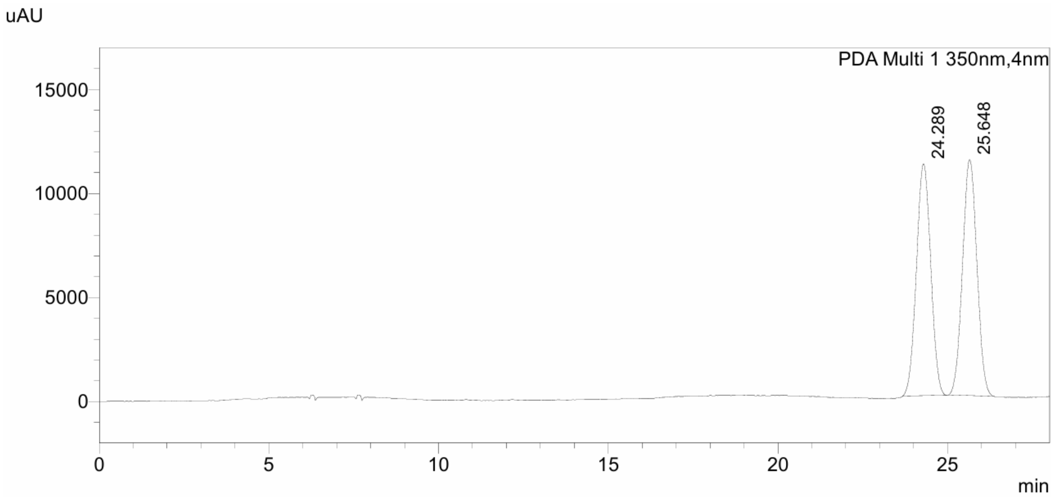

PDA Ch1 350nm

Peak#	Ret. Time	Area	Area%
1	24.289	327331	49.426
2	25.648	334931	50.574
Total		662262	100.000

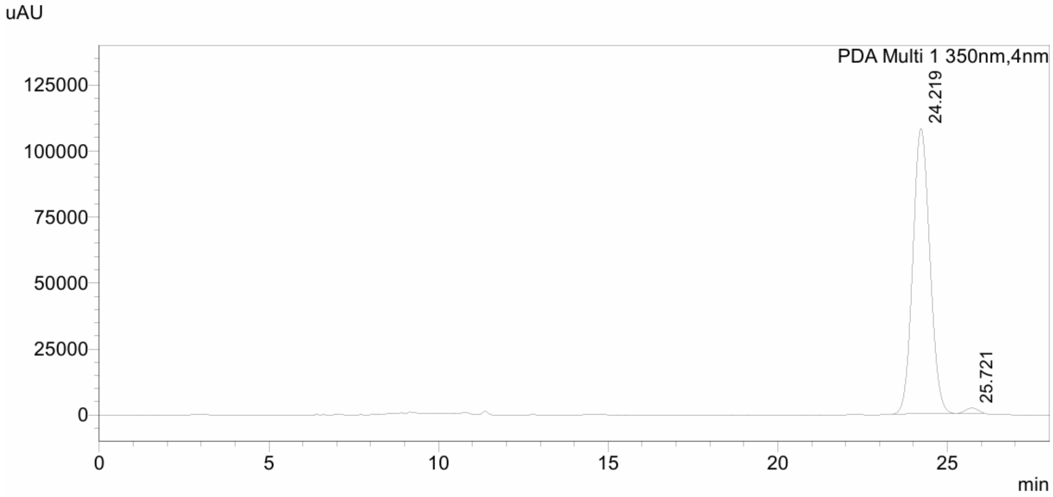

PDA Ch1 350nm

Peak#	Ret. Time	Area	Area%
1	24.219	3760154	98.771
2	25.721	46792	1.229
Total		3806946	100.000

(*S*)-6-amino-5-benzyl-3,3a-dihydro-1H-cyclopenta[c]furan-1-one (3u)

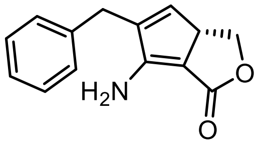

Prepared according to **General Procedure H** using **1u** (60 mg, 0.266 mmol), $Cu(OAc)_2$ (2.42 mg, 13.3 μmol), (*S*,*S*)-Ph-BPE (7.42 mg, 14.7 μmol), TMDSO (95 μL, 0.533 mmol), KF (62.10 mg, 1.065 mmol) and H_2O (47.9 μL, 2.66 mmol) in THF (2.7 mL). Purification by silica gel flash column chromatography (hexane/EtOAc 4:6) provided the title compound (30 mg, 0.130 mmol, 49% yield, *er*=99.5:0.5) as a transparent oil.

HPLC: Phenomenex cellulose-1, Hexane:2-propanol 70:30, flow: 1.0 mL/min, 213 nm, t_R = 22.5 min (major) and t_R = 27.4 min (minor).

^{1}H NMR (300 MHz, Chloroform-*d*) δ 7.34 (t, *J* = 7.2 Hz, 2H), 7.19 (d, *J* = 7.3 Hz, 2H), 7.07 (s, 1H), 4.52 (t, *J* = 9.0 Hz, 1H), 4.24 (dd, *J* = 9.5, 2.5 Hz, 1H), 3.76 (dt, *J* = 8.2, 3.8 Hz, 1H), 3.60 (d, *J* = 6.3 Hz, 1H), 3.54 (d, *J* = 4.5 Hz, 1H). (The compound is hygroscopic)

^{13}C NMR (101 MHz, Chloroform-*d*) δ 197.10, 169.62, 156.59 (d, *J* = 3.2 Hz), 146.81, 137.35, 128.97, 128.83, 126.83, 69.67, 51.38, 40.21, 31.45.

HRMS (ESI-TOF) m/z $[M+H]^+$ Calcd for $C_{14}H_{14}NO_2$ 228.1025; found 228.1264.

$[\alpha]^{25}_D$ = -75.1 (c = 0.25, $CHCl_3$)

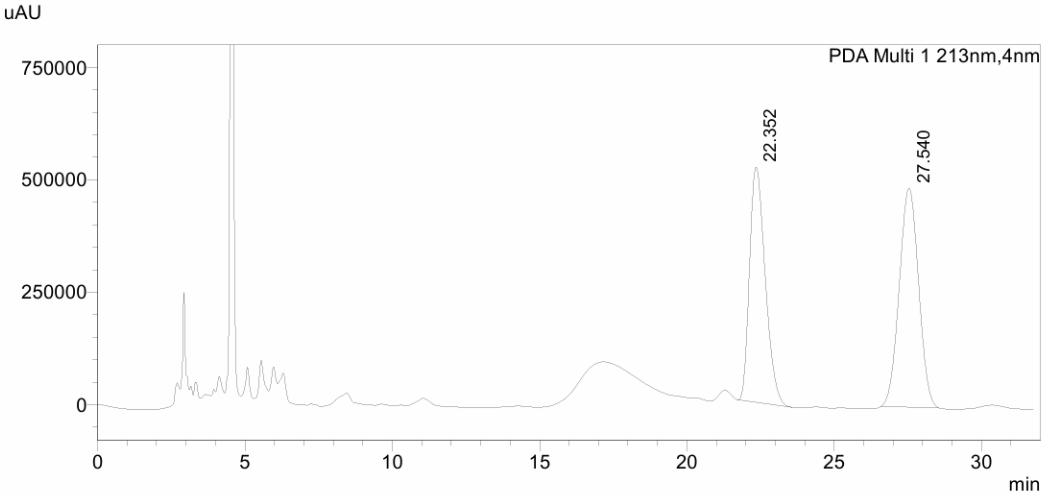

PDA Ch1 213nm

Peak#	Ret. Time	Area	Area%
1	22.352	19427803	46.614
2	27.540	22250142	53.386
Total		41677945	100.000

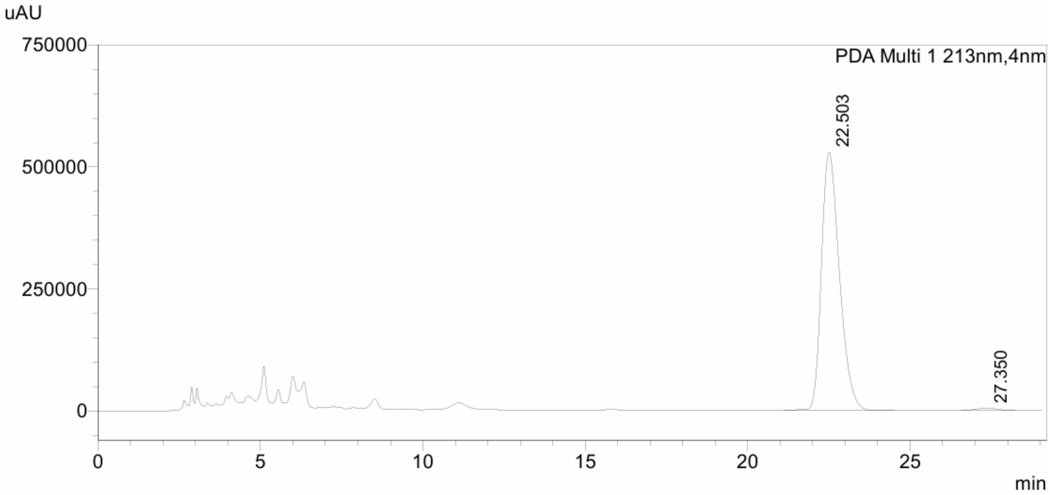

PDA Ch1 213nm

Peak#	Ret. Time	Area	Area%
1	22.503	20429581	98.883
2	27.350	230684	1.117
Total		20660265	100.000

The racemic mixture was obtained by combining the two enantiomers synthesized using (*R*,*R*)-Ph-BPE or (*S*,*S*)-Ph-BPE as chiral ligand in equimolar amounts.

Ethyl (*R*)-2-amino-5-(but-3-en-1-yl)-3-methylcyclopenta-1,3-diene-1-carboxylate (3v)

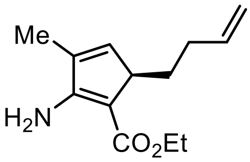

Prepared according to **General Procedure H** using **1v** (46 mg, 0.196 mmol), $Cu(OAc)_2$ (0.71 mg, 3.91 μmol), Josiphos (2.34 mg, 4.31 μmol), TMDSO (69 μL, 0.392 mmol), KF (45.48 mg, 0.784 mmol) and H_2O (7 μL, 0.392 mmol) in THF (0.78 mL). Purification by silica gel flash column chromatography (hexane/EtOAc 9:1) provided the title compound (46 mg, 0.194 mmol, 99% yield, *er*=97.5:2.5) as a transparent oil.

HPLC: Phenomenex cellulose-1, Hexane:2-propanol 96:4, flow: 1.0 mL/min, 350 nm, t_R = 10.1 min (major) and t_R = 12.5 min (minor).

^{1}H NMR (400 MHz, Chloroform-*d*) δ 6.33 (s, 1H), 5.87 – 5.78 (m, 1H), 5.78 – 5.31 (m, 2H), 5.06 – 4.89 (m, 2H), 4.31 – 4.12 (m, 2H), 3.29 (ddq, *J* = 8.9, 3.4, 1.6 Hz, 1H), 2.17 – 1.95 (m, 3H), 1.92 (s, 3H), 1.56 – 1.47 (m, 1H), 1.32 (t, *J* = 7.1 Hz, 3H).

^{13}C NMR (101 MHz, Chloroform-*d*) δ 166.76, 160.97, 141.38, 139.23, 135.04, 114.14, 100.79, 58.45, 47.32, 31.01, 29.52, 14.78, 11.63.

HRMS (ESI-TOF) m/z $[M+H]^+$ Calcd for $C_{13}H_{20}NO_2$ 222.1494; found 222.1494.

$[\alpha]^{25}_D$ = -187.8 (c = 0.6, $CHCl_3$).

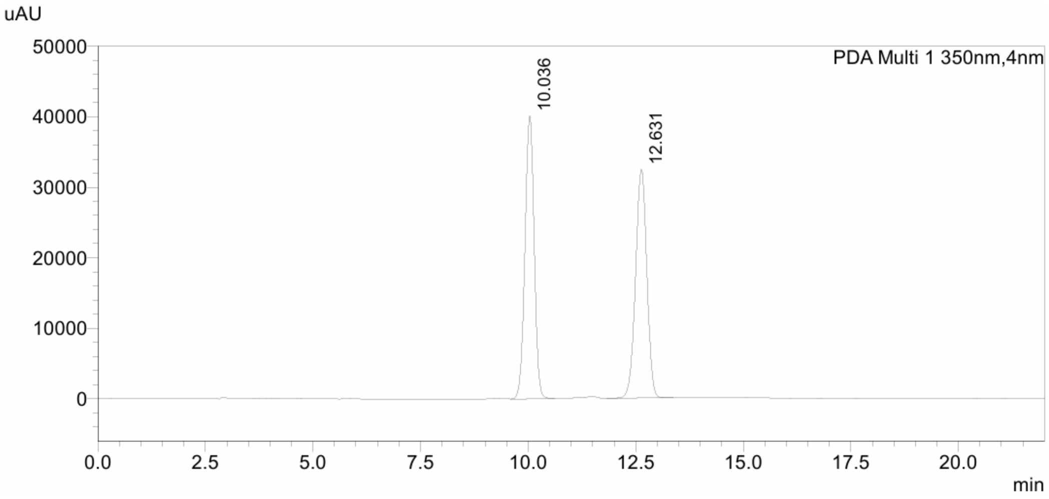

PDA Ch1 350nm

Peak#	Ret. Time	Area	Area%
1	10.036	595111	50.197
2	12.631	590450	49.803
Total		1185561	100.000

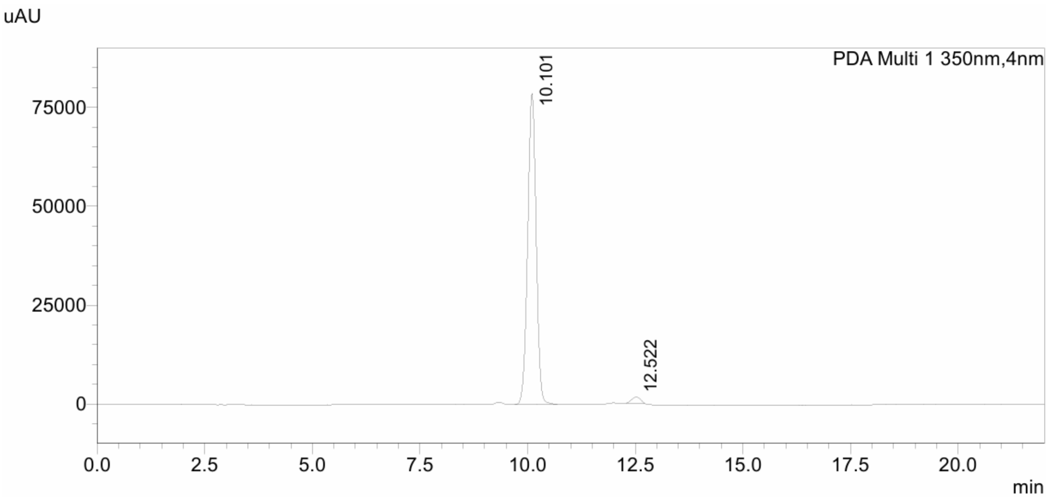

PDA Ch1 350nm

Peak#	Ret. Time	Area	Area%
1	10.101	1078438	98.069
2	12.522	21239	1.931
Total		1099677	100.000

Ethyl (*R*)-5-(2-(1,3-dioxolan-2-yl)ethyl)-2-amino-3-benzylcyclopenta-1,3-diene-1-carboxylate (3w)

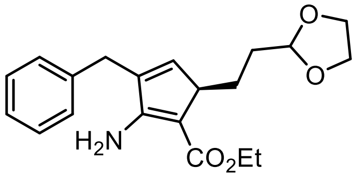

Prepared according to **General Procedure H** using **1w** (67 mg, 0.196 mmol), $Cu(OAc)_2$ (0.71 mg, 3.92 μmol), Josiphos (2.34 mg, 4.31 μmol), TMDSO (69 μL, 0.392 mmol), KF (45.55 mg, 0.784 mmol) and H_2O (7 μL, 0.392 mmol) in THF (0.78 mL). Purification by silica gel flash column chromatography (hexane/EtOAc 7:3) provided the title compound (51 mg, 0.147 mmol, 75% yield, *er*=98:2) as a transparent oil.

HPLC: Phenomenex cellulose-1, Hexane:2-propanol 90:10, flow: 1.0 mL/min, 316 nm, t_R = 18.2 min (major) and t_R = 20.6 min (minor).

^{1}H NMR (400 MHz, Chloroform-*d*) δ 7.41 – 7.14 (m, 5H), 6.27 (s, 1H), 5.57 (s, 2H), 4.83 (t, *J* = 4.7 Hz, 1H), 4.29 – 4.13 (m, 2H), 3.96 (s, 2H), 3.84 (s, 2H), 3.66 (s, 2H), 3.43 – 3.37 (m, 1H), 2.12 (tt, *J* = 10.6, 4.9 Hz, 1H), 1.75 (dq, *J* = 14.6, 8.0, 6.8 Hz, 1H), 1.65 – 1.52 (m, 2H), 1.31 (t, *J* = 7.1 Hz, 3H).

^{13}C NMR (101 MHz, Chloroform-*d*) δ 166.71, 160.62, 142.66, 138.61, 137.66, 128.82, 128.56, 126.74, 104.91, 101.12, 64.87, 64.80, 58.52, 47.29, 33.19, 30.39, 24.47, 14.77.

HRMS (ESI-TOF) m/z $[M+H]^+$ Calcd for $C_{20}H_{26}NO_4$ 344.1862; found 344.2006.

$[\alpha]^{25}_D$ = 36.5 (c = 0.18, $CHCl_3$).

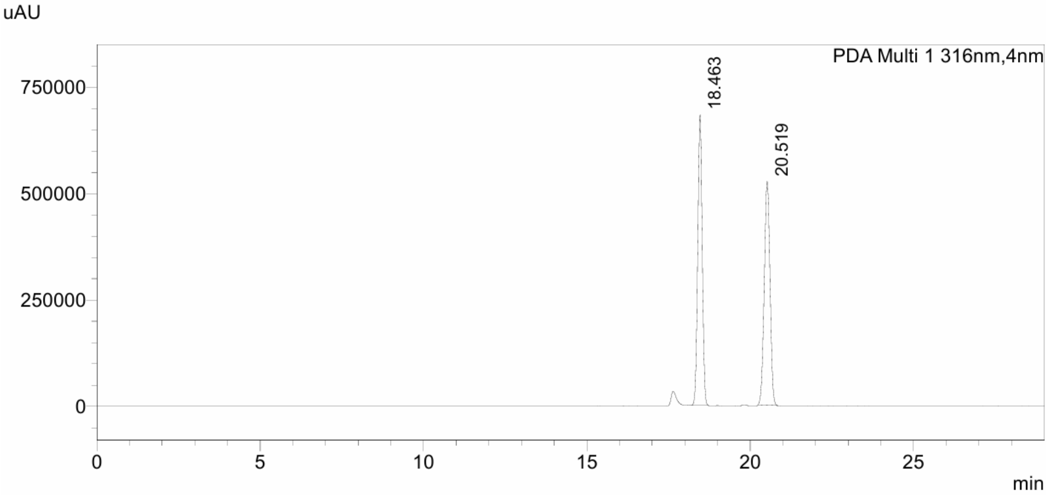

PDA Ch1 316nm

Peak#	Ret. Time	Area	Area%
1	18.463	6510572	50.579
2	20.519	6361503	49.421
Total		12872074	100.000

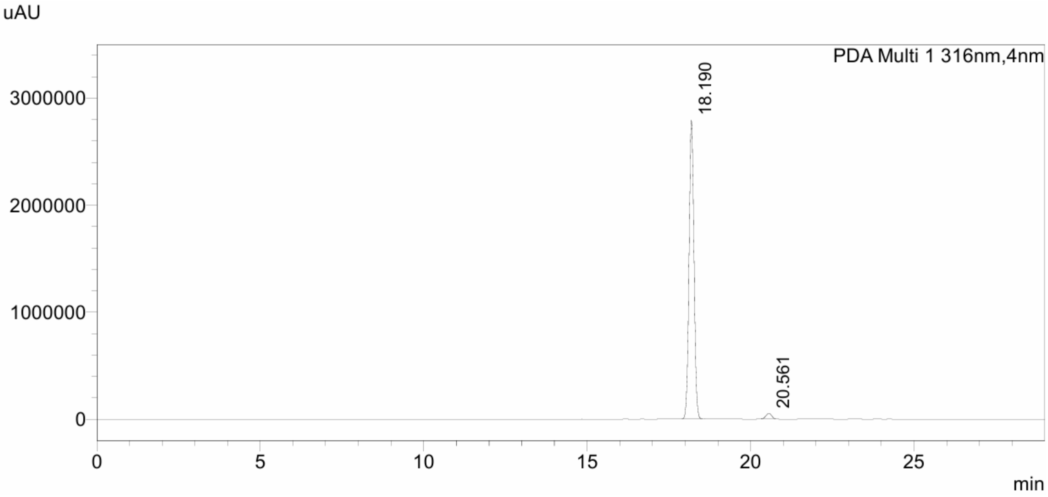

PDA Ch1 316nm

Peak#	Ret. Time	Area	Area%
1	18.190	28977045	97.912
2	20.561	618063	2.088
Total		29595108	100.000

Ethyl (*S*)-2-amino-3-benzyl-5-phenylcyclopenta-1,3-diene-1-carboxylate (3x)

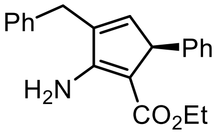

Prepared according to **General Procedure H** using **1x** (25 mg, 0.079 mmol), $Cu(OAc)_2$ (0.72 mg, 3.94 μmol), (*R,R*)-Ph-BPE (2.19 mg, 4.33 μmol), TMDSO (42 μL, 0.158 mmol), KF (18.30 mg, 0.315 mmol) and H_2O (2.8 μL, 0.158 mmol) in THF (0.79 mL). Purification by silica gel flash column chromatography (hexane/EtOAc 9:1) provided the title compound (18 mg, 0.057 mmol, 72% yield, *er*=98.5:1.5) as a brown oil.

HPLC: Phenomenex cellulose-1, Hexane:2-propanol 96:4, flow: 1.0 mL/min, 338 nm, t_R = 29.4 min (minor) and t_R = 31.4 min (major).

^{1}H NMR (300 MHz, Chloroform-*d*) δ 7.24 (ddd, *J* = 37.9, 22.1, 7.0 Hz, 10H), 6.31 (s, 1H), 5.68 (s, 2H), 4.45 (s, 1H), 4.12 – 3.92 (m, 2H), 3.73 (s, 2H), 1.05 (t, *J* = 7.0 Hz, 3H).

^{13}C NMR (101 MHz, Chloroform-*d*) δ 166.53, 161.07, 143.43, 139.35, 138.31, 137.49, 128.90, 128.57, 128.09, 127.73, 126.86, 126.19, 103.17, 58.49, 53.61, 33.15, 14.38.

HRMS (ESI-TOF) m/z [M+H]$^+$ Calcd for $C_{21}H_{22}NO_2$ 320.1651; found 320.1687.

[α]$^{25}_D$ = -513.3 (c = 0.2, $CHCl_3$).

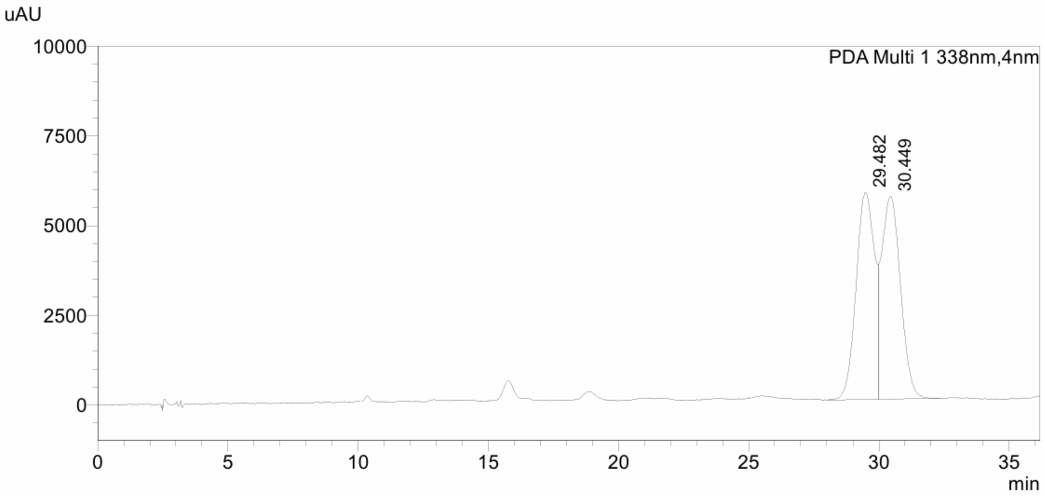

PDA Ch1 338nm

Peak#	Ret. Time	Area	Area%
1	29.482	284811	49.776
2	30.449	287379	50.224
Total		572190	100.000

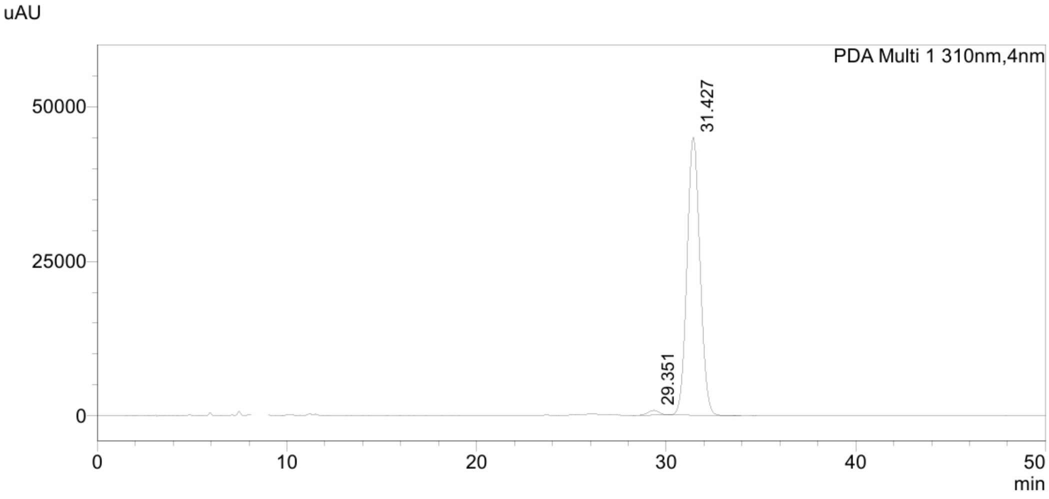

PDA Ch1 310nm

Peak#	Ret. Time	Area	Area%
1	29.351	30857	1.415
2	31.427	2149085	98.585
Total		2179942	100.000

Ethyl (*S*)-2-amino-3-benzyl-5-(naphthalen-2-yl)cyclopenta-1,3-diene-1-carboxylate (3y)

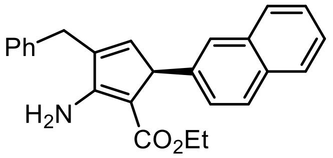

Prepared according to **General Procedure H** using **1y** (40 mg, 0.109 mmol), $Cu(OAc)_2$ (0.99 mg, 5.45 μmol), (*R*,*R*)-Ph-BPE (3.03 mg, 6.00 μmol), TMDSO (38.5 μL, 0.218 mmol), KF (32.12 mg, 0.55 mmol) and H_2O (3.9 μL, 0.218 mmol) in THF (1.09 mL). Purification by silica gel flash column chromatography (hexane/EtOAc 8:2) provided the title compound (32 mg, 0.088 mmol, 81% yield, *er*=98.5:1.5) as a dark yellow oil.

HPLC: Phenomenex cellulose-1, Hexane:2-propanol 90:10, flow: 1.0 mL/min, 350 nm, t_R = 8.6 min (minor) and t_R = 12.8 min (major).

^{1}H NMR (300 MHz, Chloroform-*d*) δ 7.32 – 7.77 (m, 3H), 7.69 (d, *J* = 1.3 Hz, 1H), 7.48 – 7.42 (m, 2H), 7.35 (d, *J* = 8.1 Hz, 1H), 7.24 – 7.12 (m, 5H), 6.33 (s, 1H), 5.68 (br s, 2H), 4.45 (s, 1H), 3.99 (q, *J* = 7.0 Hz, 2H), 3.87 (s, 2H), 1.03 (t, *J* = 7.0 Hz, 3H).

^{13}C NMR (101 MHz, Chloroform-*d*) δ 166.72, 161.30, 143.50, 138.71, 137.58, 137.07, 133.81, 132.57, 129.04, 128.72, 127.72, 127.67, 127.58, 127.02, 126.67, 125.94, 125.80, 125.23, 103.30, 58.64, 53.80, 33.31, 14.52.

HRMS (ESI-TOF) m/z $[M+H]^+$ Calcd for $C_{22}H_{24}NO_2$ 370.1807; found 370.1790.

$[\alpha]^{25}_D$ = -24.4 (c = 0.1, $CHCl_3$).

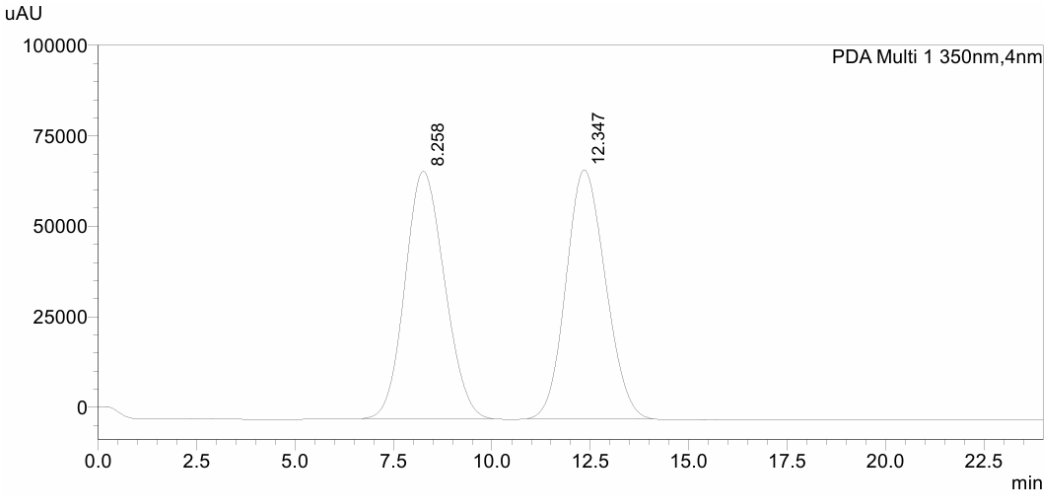

PDA Ch1 350nm

Peak#	Ret. Time	Area	Area%
1	8.258	4768533	49.849
2	12.347	4797399	50.151
Total		9565932	100.000

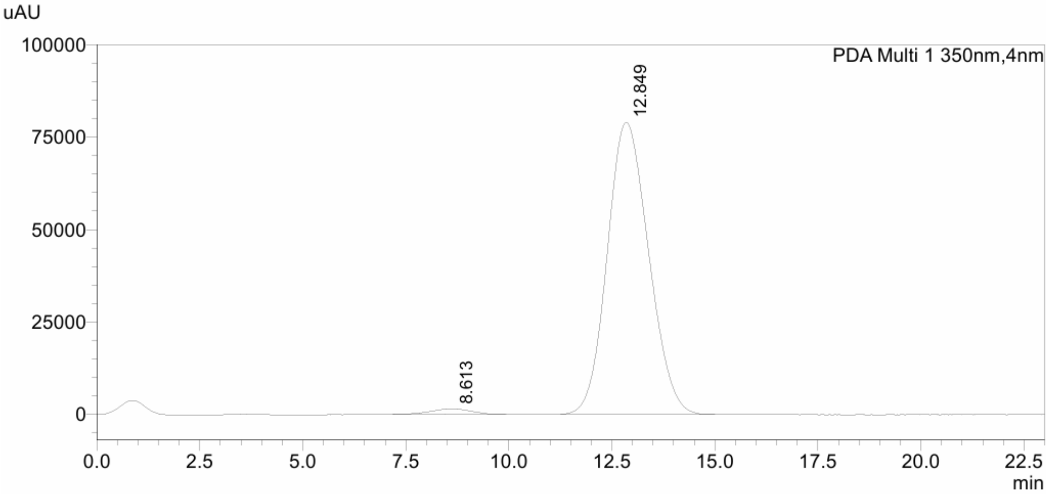

PDA Ch1 350nm

Peak#	Ret. Time	Area	Area%
1	8.613	90715	1.616
2	12.849	5524416	98.384
Total		5615131	100.000

Ethyl (*S*)-2-amino-3-benzyl-5-(pyridin-4-yl)cyclopenta-1,3-diene-1-carboxylate (3z)

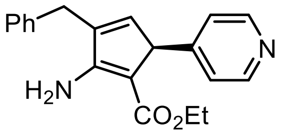

Prepared according to **General Procedure H** using **1z** (42 mg, 0.132 mmol), $Cu(OAc)_2$ (1.20 mg, 6.60 µmol), (*R*,*R*)-Ph-BPE (3.67 mg, 7.26 µmol), TMDSO (46.6 µL, 0.264 mmol), KF (28.10 mg, 0.528 mmol), H_2O (4.8 µL, 0.264 mmol) in THF (1.32 mL). Purification by silica gel flash column chromatography (hexane/EtOAc 1:1) provided the title compound (30 mg, 0.092 mmol, 70% yield, *er*=97.5:2.5) as a dark yellow oil.

HPLC: Phenomenex cellulose-1, Hexane:2-propanol 80:20, flow: 1.0 mL/min, 325 nm, t_R = 15.3 min (minor) and t_R = 25.6 min (major).

^{1}H NMR (400 MHz, Chloroform-*d*) δ 8.55 (d, *J* = 6.0 Hz, 2H), 7.25 – 7.21 (m, 2H), 7.18 – 7.17 (m, 3H),7.09 (d, *J* = 6.0 Hz, 2H), 6.27 (d, *J* = 1.4 Hz, 1H), 5.65 (br s, 2H), 4.44 (d, *J* = 1.4 Hz, 1H), 4.01 (q, *J* = 7.1 Hz, 2H), 3.68 (s, 2H), 1.03 (t, *J* = 7.1 Hz, 3H).

^{13}C NMR (101 MHz, Chloroform-*d*) δ 166.59, 160.25, 150.33, 146.84, 144.28, 138.95, 136.65, 128.32, 127.77, 126.49, 124.03, 103.58, 58.75, 53.91, 32.51, 14.47.

HRMS (ESI-TOF) m/z $[M+H]^+$ Calcd for $C_{20}H_{21}N_2O_2$ 321.1603; found 321.1763.

$[\alpha]^{25}_D$ = +26.4 (c = 0.1, $CHCl_3$).

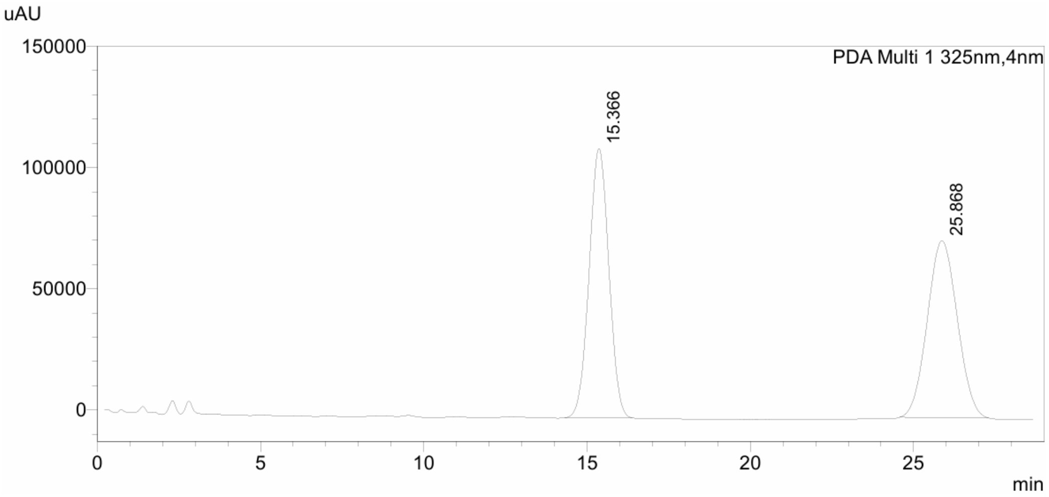

PDA Ch1 325nm

Peak#	Ret. Time	Area	Area%
1	15.366	4677564	50.078
2	25.868	4662921	49.922
Total		9340484	100.000

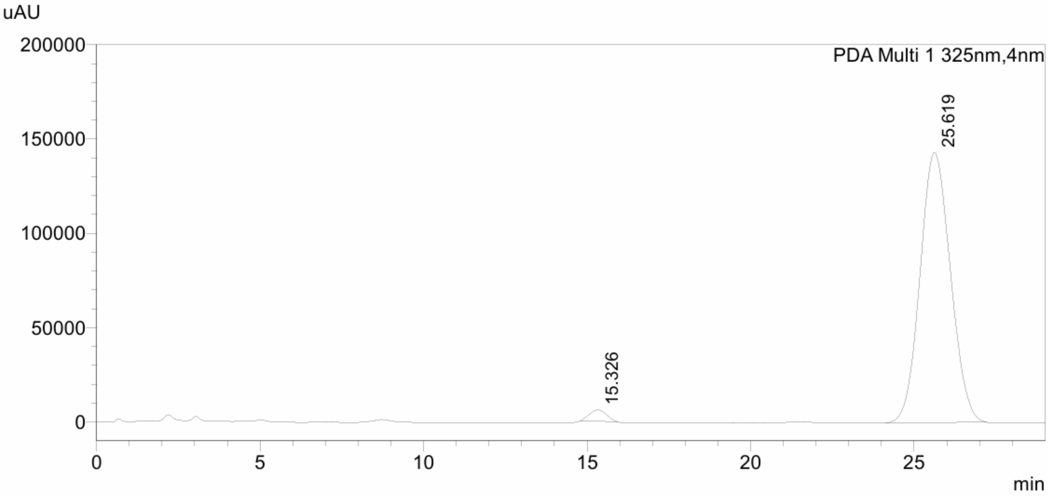

PDA Ch1 325nm

Peak#	Ret. Time	Area	Area%
1	15.326	215608	2.274
2	25.619	9266903	97.726
Total		9482511	100.000

Ethyl (S)-2-amino-3-fluoro-4,5-dimethylcyclopenta-1,3-diene-1-carboxylate (3aa)

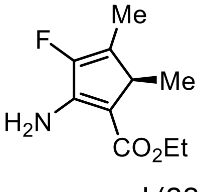

Prepared according to **General Procedure H** using **1aa** (39 mg, 0.196 mmol), $Cu(OAc)_2$ (0.71 mg, 3.91 µmol), Josiphos (2.34 mg, 4.31 µmol), TMDSO (69 µL, 0.392 mmol), KF (45.48 mg, 0.784 mmol) and H_2O (7 µL, 0.392 mmol) in THF (0.78 mL). Purification by silica gel flash column chromatography (hexane/EtOAc 9:1) provided the title compound (23 mg, 0.118 mmol, 60% yield, *er*=97:3) as a pale yellow oil.

HPLC: Phenomenex cellulose-3, Hexane:2-propanol 99:1, flow: 1.0 mL/min, 303 nm, t_R = 9.2 min (major) and t_R = 10.1 min (minor).

^{1}H NMR (400 MHz, Chloroform-*d*) δ 5.5 (br s, 1H), 4.7 – 4.0 (m, 2H), 3.0 (t, *J*=7.0 Hz, 1H), 1.9 (s, 3H), 1.3 (t, *J*=7.1 Hz, 3H), 1.2 (d, *J*=7.2 Hz, 3H).

^{13}C NMR (101 MHz, Chloroform-*d*) δ 166.0, 149.3 (d, *J*=267.6 Hz), 135.0, 128.1, 99.7 (d, *J*=3.8 Hz), 58.7, 40.5 (d, *J*=5.8 Hz), 15.3 (d, *J*=2.1 Hz), 14.7, 9.2.

^{19}F NMR (377 MHz, Chloroform-*d*) δ -147.04.

HRMS (ESI-TOF) m/z $[M+H]^+$ Calcd for $C_{10}H_{15}FNO_2$ 200.1087; found 200.1082.

$[\alpha]^{25}_D$ = -54.5 (c = 0.1, $CHCl_3$).

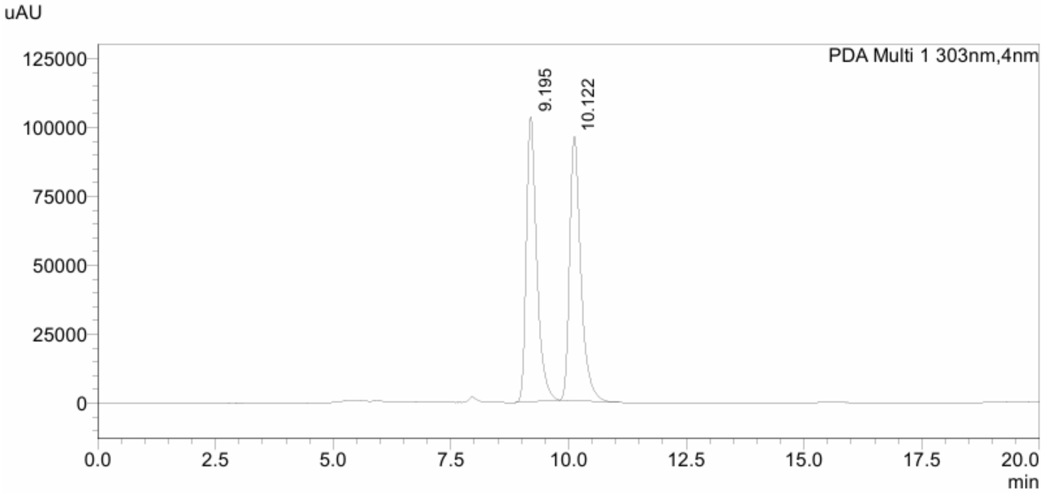

PDA Ch1 303nm

Peak#	Ret. Time	Area	Area%
1	9.195	1585747	50.046
2	10.122	1582809	49.954
Total		3168556	100.000

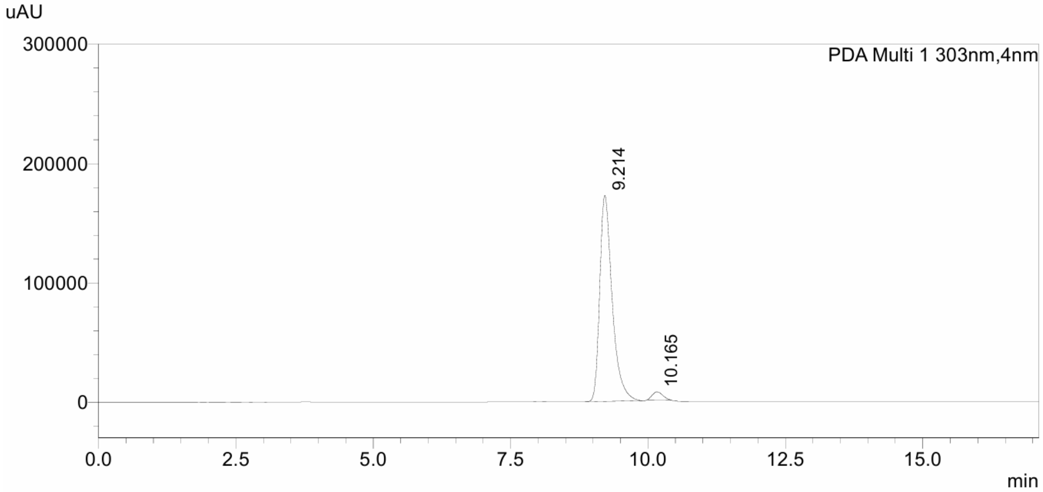

PDA Ch1 303nm

Peak#	Ret. Time	Area	Area%
1	9.214	2718235	96.680
2	10.165	93342	3.320
Total		2811577	100.000

6 Manipulations of Cyclopentadienes

Ethyl (1*S*,5*R*)-3-benzyl-1-fluoro-5-methyl-2-oxocyclopent-3-ene-1-carboxylate (4)

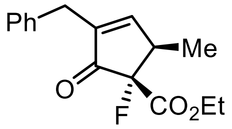

Cyclopentadiene **3a** (50 mg, 0.194 mmol, 1 eq) was charged in a 4 mL sealed vial under nitrogen atmosphere. Dry acetonitrile was added (0.2 M) and the vial was heated to 40 °C. In a separate vial, Selectfluor® (103 mg, 0.291 mmol, 1.5 eq) was dissolved in dry acetonitrile (0.1 M) and heated to 40 °C. The latter solution was then added to the first vial and the mixture was stirred at 40 °C for 1.5 hours. The reaction was quenched with a saturated solution of NH_4Cl and the product was extracted 3 times with EtOAc. The organic layers were collected and the solvent was evaporated under reduced pressure. Column chromatography (hexane/EtOAc 8:2) afforded the desired product (46 mg, 0.165 mmol, 85% yield, *er*=98.5:1.5, *dr*>20:1) as a colorless oil.

HPLC: Phenomenex cellulose-1, Hexane:2-propanol 96:4, flow: 1.0 mL/min, 230 nm, t_R = 9.6 min (minor) and t_R = 10.2 min (major).

^{1}H NMR (400 MHz, Chloroform-*d*) δ 7.31 (t, *J* = 7.3 Hz, 3H), 7.23 (d, *J* = 7.4 Hz, 1H), 7.19 (d, *J* = 7.3 Hz, 2H), 4.25 (q, *J* = 7.1 Hz, 2H), 3.61 – 3.46 (m, 2H), 3.36 – 3.18 (m, 1H), 1.26 (t, *J* = 7.1 Hz, 3H), 1.17 (dd, *J* = 7.3, 4.4 Hz, 3H).

^{13}C NMR (101 MHz, Chloroform-*d*) δ 197.73 (d, *J* = 19.1 Hz), 167.32 (d, *J* = 28.1 Hz), 163.05, 160.20, 142.52, 137.40, 128.79 (d, *J* = 19.4 Hz), 126.68, 93.63 (d, *J* = 205.9 Hz), 62.48, 42.30, 42.09, 31.25, 14.01.

^{19}F NMR (377 MHz, Chloroform-*d*) δ -178.88.

HRMS (ESI-TOF) m/z $[M+Na]^+$ Calcd for $C_{16}H_{17}FO_3Na$ 299.1059; found 299.1064.

$[\alpha]^{25}_D$ = 5.3 (c = 1.0, $CHCl_3$).

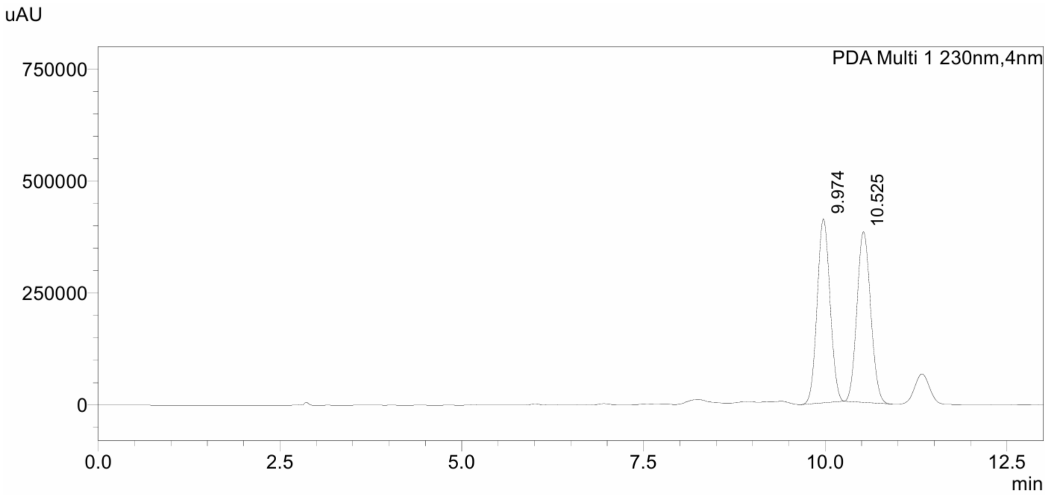

PDA Ch1 230nm

Peak#	Ret. Time	Area	Area%
1	9.974	5077968	50.204
2	10.525	5036763	49.796
Total		10114730	100.000

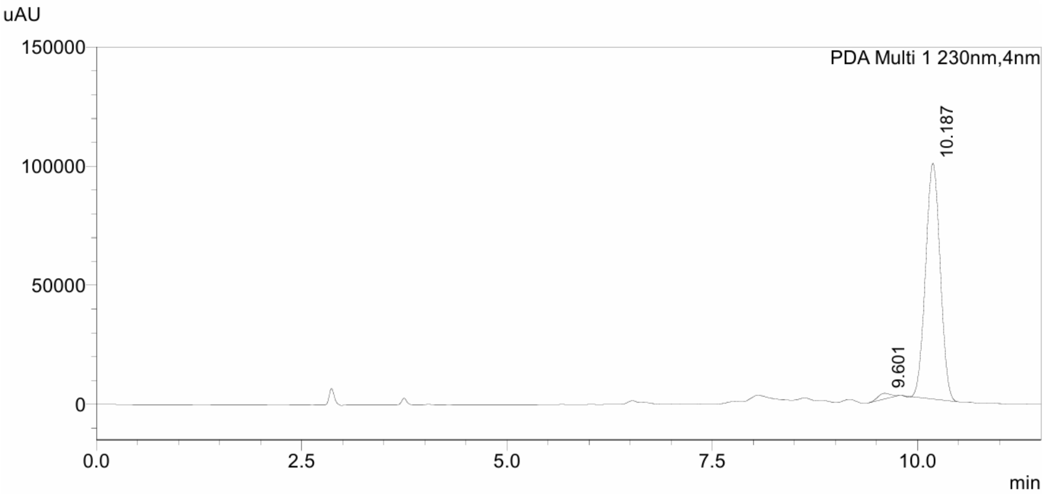

PDA Ch1 230nm

Peak#	Ret. Time	Area	Area%
1	9.601	27307	2.176
2	10.187	1227831	97.824
Total		1255138	100.000

Ethyl (1*S*,5*R*)-3-benzyl-1-hydroxy-5-methyl-2-oxocyclopent-3-ene-1-carboxylate (5)

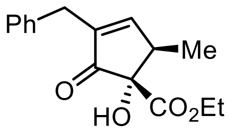

Cyclopentadiene **3a** (50 mg, 0.194 mmol, 1 eq) was charged in a 4 mL sealed vial under nitrogen atmosphere. The compound was diluted in dry DCM (0.2 M) and cooled to 0 °C. mCPBA (40 mg, 0.233 mmol, 1.2 eq) was added in one portion, the N_2 atmosphere was restored and the mixture was stirred at 0 °C for 1 hour. The reaction was quenched with a saturated solution of NH_4Cl and the product was extracted 3 times with EtOAc. The organic layers were collected and the solvent was evaporated under reduced pressure. Column chromatography (hexane/EtOAc 8:2) afforded the desired product as a pair of separable diastereomers in 2:1 ratio (*major*: 20 mg, 0.071 mmol, 55% yield, *er*=99:1; *minor*: 9 mg, 0.035 mmol, 26% yield, *er*=98.5:1.5).

Major diastereomer:

^{1}H NMR (400 MHz, Chloroform-*d*) δ 7.32 (t, *J* = 7.4 Hz, 2H), 7.29 – 7.16 (m, 4H), 4.22 (qd, *J* = 7.1, 2.9 Hz, 2H), 3.69 – 3.45 (m, 2H), 3.28 – 3.12 (m, 1H), 1.22 (t, *J* = 7.1 Hz, 3H), 1.13 (d, *J* = 7.3 Hz, 3H).

^{13}C NMR (101 MHz, Chloroform-*d*) δ 203.10, 171.69, 164.31, 141.86, 137.91, 128.85, 128.58, 126.50, 79.80, 62.76, 42.62, 31.33, 14.59, 13.95.

HPLC: Phenomenex cellulose-1, Hexane:2-propanol 80:20, flow: 1.0 mL/min, 236 nm, t_R = 6.9 min (major) and t_R = 7.4 min (minor).

HRMS (ESI-TOF) m/z $[M+H]^+$ Calcd for $C_{16}H_{19}O_4$ 275.1283; found 275.1491.

$[\alpha]^{25}_D$ = -39.5 (c = 1.0, $CHCl_3$).

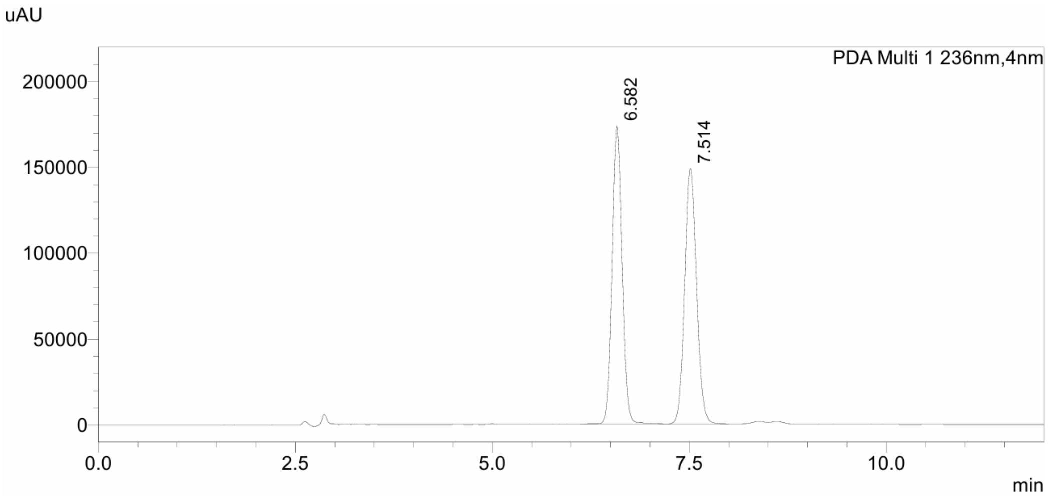

PDA Ch1 236nm

Peak#	Ret. Time	Area	Area%
1	6.582	1540092	49.620
2	7.514	1563685	50.380
Total		3103777	100.000

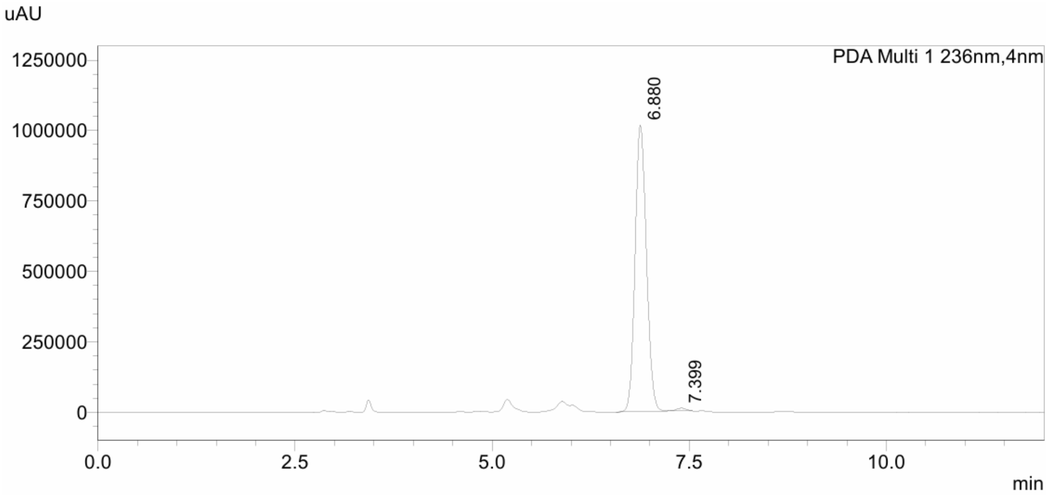

PDA Ch1 236nm

Peak#	Ret. Time	Area	Area%
1	6.880	10159497	99.137
2	7.399	88409	0.863
Total		10247906	100.000

Minor diastereomer:

^{1}H NMR (400 MHz, Chloroform-*d*) δ 7.37 – 7.29 (m, 2H), 7.24 (dd, *J* = 12.6, 7.1 Hz, 3H), 7.02 (s, 1H), 4.21 (qt, *J* = 6.8, 3.5 Hz, 2H), 3.62 – 3.49 (m, 2H), 3.04 (dtt, *J* = 7.5, 5.0, 2.4 Hz, 1H), 1.55 – 0.99 (m, 6H).

^{13}C NMR (101 MHz, $CDCl_3$) δ 203.09, 170.56, 160.73, 143.27, 137.90, 128.90, 128.56, 126.50, 83.51, 77.35, 77.03, 76.71, 62.50, 45.44, 31.47, 14.00, 13.41.

HPLC: Phenomenex cellulose-1, Hexane:2-propanol 94:6, flow: 1.0 mL/min, 231 nm, t_R = 15.3 min (major) and t_R = 16.4 min (minor).

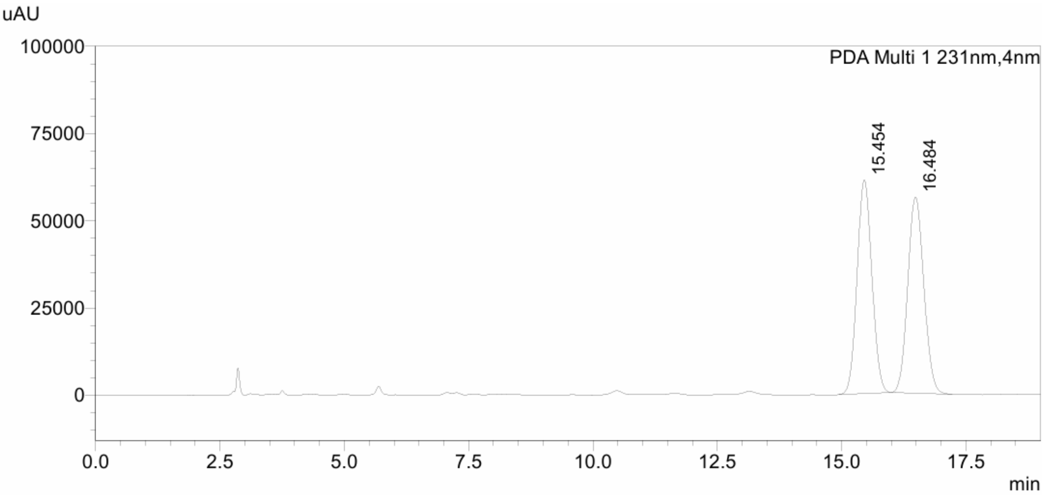

PDA Ch1 231nm

Peak#	Ret. Time	Area	Area%
1	15.454	1270701	50.103
2	16.484	1265482	49.897
Total		2536183	100.000

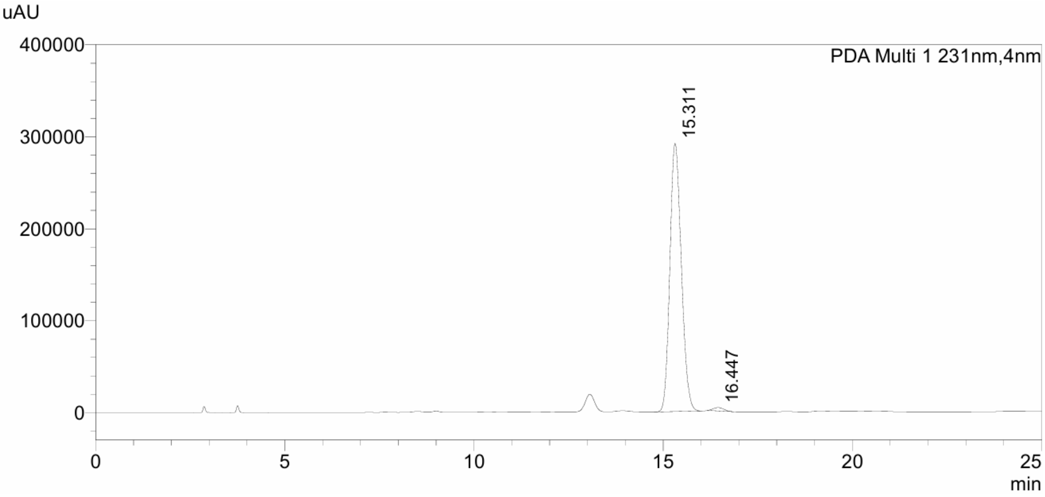

PDA Ch1 231nm

Peak#	Ret. Time	Area	Area%
1	15.311	6138536	98.822
2	16.447	73163	1.178
Total		6211700	100.000

Ethyl (1*R*,5*R*)-1-allyl-3-benzyl-5-methyl-2-oxocyclopent-3-ene-1-carboxylate (6)

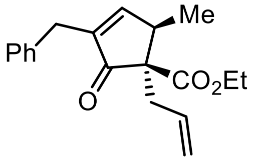

A 4 ml oven dried vial filled with nitrogen was charged via syringe with *n*BuLi (2.5 M in THF, 74 µL, 0.185 mmol, 0.95 eq), which was further diluted to 0.4 M with dry THF. The mixture was cooled to -78 °C and diisopropylamine (26 µL, 0.185 mmol, 0.95 eq) was added and stirred for 10 min. Cyclopentadiene **3a** (50 mg, 0.194 mmol, 1 eq) was diluted in dry THF (0.4 M) in a 4 mL vial and cooled to -78 °C. The LDA solution was then added to the substrate and stirred for 5 minutes at the same temperature. Allyl iodide (35 µL, 0.389 mmol, 2 eq) was then added and the mixture was gradually warmed to room temperature. The reaction was quenched with a saturated solution of NH_4Cl and the product was extracted 3 times with EtOAc. The organic layers were collected and the solvent was evaporated under

reduced pressure. Column chromatography (hexane/EtOAc 70:30) afforded the *N*-allylated product as a yellow oil. This compound was then diluted in THF/HCl 1N 1:1 (0.25 M) and vigorously stirred for 16 hours. The product was extracted 3 times with EtOAc, the organic layers were collected and the solvent was evaporated under reduced pressure. Column chromatography (hexane/EtOAc 70:30) afforded the rearranged product (48 mg, 0.159 mmol, 82% yield, *er*=98.5:1.5, *dr*=7:3) as a pale yellow oil.

HPLC: Phenomenex cellulose-1, Hexane:2-propanol 90:10, flow: 1.0 mL/min, 218 nm, t_R = 6.3 min (major) and t_R = 6.9 min (minor).

^{1}H NMR (400 MHz, Chloroform-*d*) δ 7.35 – 7.27 (m, 2H), 7.25 (q, *J* = 6.6, 6.1 Hz, 3H), 5.76 (ddt, *J* = 17.2, 10.8, 7.2 Hz, 1H), 5.18 – 5.00 (m, 2H), 4.10 – 3.96 (m, 2H), 3.47 – 3.02 (m, 2H), 2.71 – 2.52 (m, 2H), 1.15 (t, *J* = 7.2 Hz, 3H), 1.01 (d, *J* = 7.6 Hz, 3H). (major)

^{13}C NMR (101 MHz, Chloroform-*d*) δ 204.50, 170.38, 161.43, 144.44, 134.92, 133.32, 129.84, 128.39, 126.85, 118.75, 66.23, 61.13, 39.42, 38.06, 31.22, 14.08, 13.40. (major)

HRMS (ESI-TOF) m/z $[M+H]^+$ Calcd for $C_{19}H_{23}O_3$ 299.1647; found 299.1697.

$[\alpha]^{25}_D$ = -19.3 (c = 1.0, $CHCl_3$).

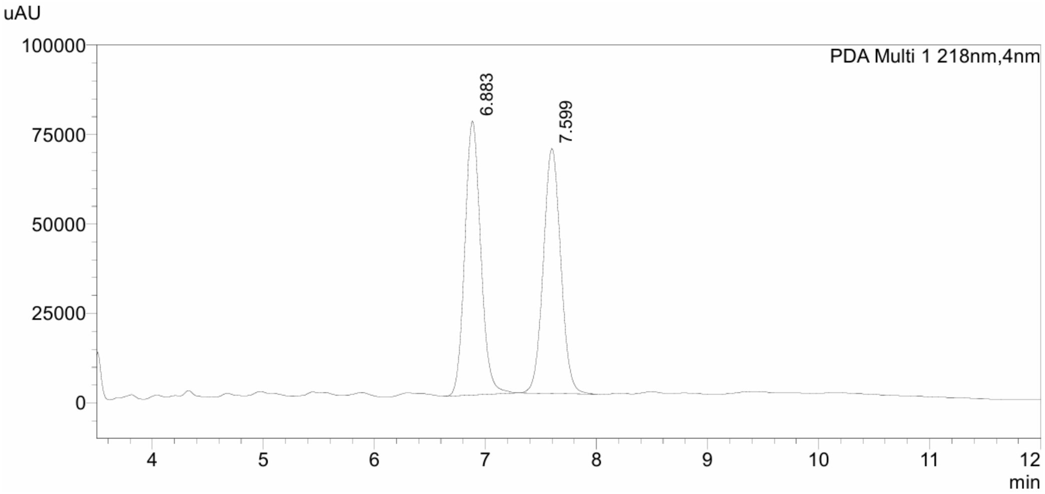

PDA Ch1 218nm

Peak#	Ret. Time	Area	Area%
1	6.883	763750	50.474
2	7.599	749418	49.526
Total		1513167	100.000

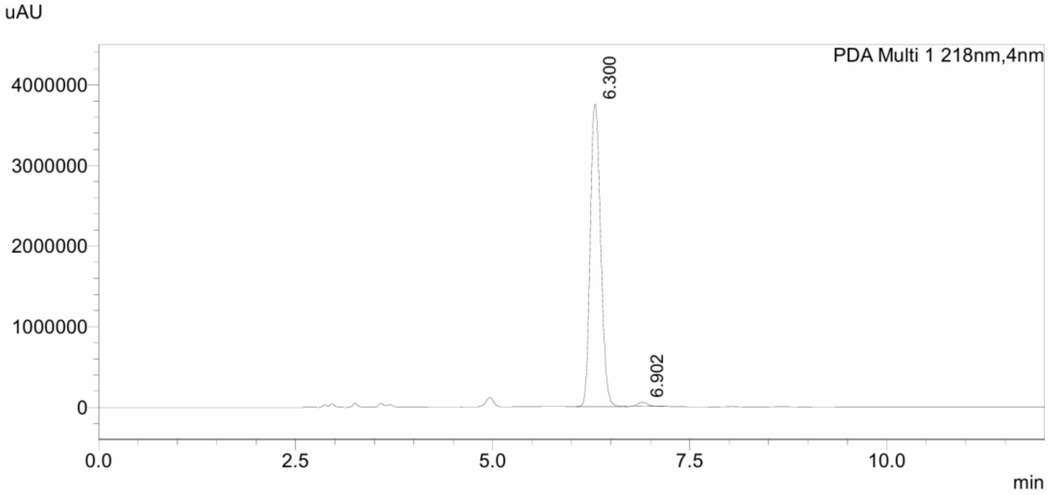

PDA Ch1 218nm

Peak#	Ret. Time	Area	Area%
1	6.300	34678675	98.830
2	6.902	410580	1.170
Total		35089255	100.000

Ethyl (1*S*,5*R*)-3-benzyl-5-methyl-2-oxo-1-(p-tolyl)cyclopent-3-ene-1-carboxylate (7)

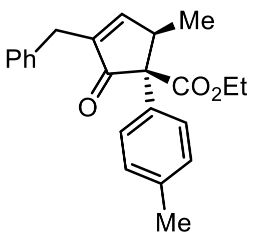

In a 4 mL vial, $(CuOTf)_2$ (1.24 mg, 0.010 mmol, 5 mol%), *rac*-BINAPO (4.58 mg, 0.012 mmol, 6 mol%) and mesityl(*p*-tolyl)iodonium triflate (85 mg, 0.291 mmol, 1.5 eq) were charged and a nitrogen atmosphere was generated. Dry DCM (1 mL) was added and the mixture was stirred at room temperature for 5 minutes. The cyclopentadiene **3a** (50 mg, 0.194 mmol, 1 eq) was added and the mixture was stirred at room temperature for 16 hours. The reaction was quenched with a saturated solution of NH_4Cl and the product was extracted 3 times with EtOAc. The organic layers were collected and the solvent was evaporated under reduced pressure. Column chromatography (hexane/EtOAc 95:5) afforded the desired product (36 mg, 0.102 mmol, 53% yield, *er*=98.5:1.5, *dr*>20:1) as a yellow oil.

HPLC: Phenomenex cellulose-4, Hexane:2-propanol 96:4, flow: 1.0 mL/min, 337 nm, t_R = 4.4 min (minor) and t_R = 5.2 min (major).

^{1}H NMR (300 MHz, Chloroform-*d*) δ 7.20 – 7.16 (m, 3H), 7.08 (d, *J* = 8.0 Hz, 2H), 6.91 (m, 4H), 6.08 (s, 1H), 4.24 (dddd, *J* = 17.8, 14.1, 8.9, 5.4 Hz, 2H), 3.36 (q, *J* = 8.4, 7.3 Hz, 1H), 3.23 (s, 2H), 2.35 (s, 3H), 1.34 (t, *J* = 7.1 Hz, 3H), 1.25 (d, *J* = 7.4 Hz, 3H).

^{13}C NMR (101 MHz, Chloroform-*d*) δ 167.15, 159.48, 146.97, 140.04, 138.97, 138.29, 134.45, 129.34, 128.84, 128.10, 125.98, 124.57, 109.42, 58.83, 42.45, 35.70, 20.93, 15.93, 14.72.

HRMS (ESI-TOF) m/z $[M+H]^+$ Calcd for $C_{23}H_{25}O_3$ 349.1804; found 349.2008.

$[\alpha]^{25}_D$ = -45.5 (c = 1.0, $CHCl_3$).

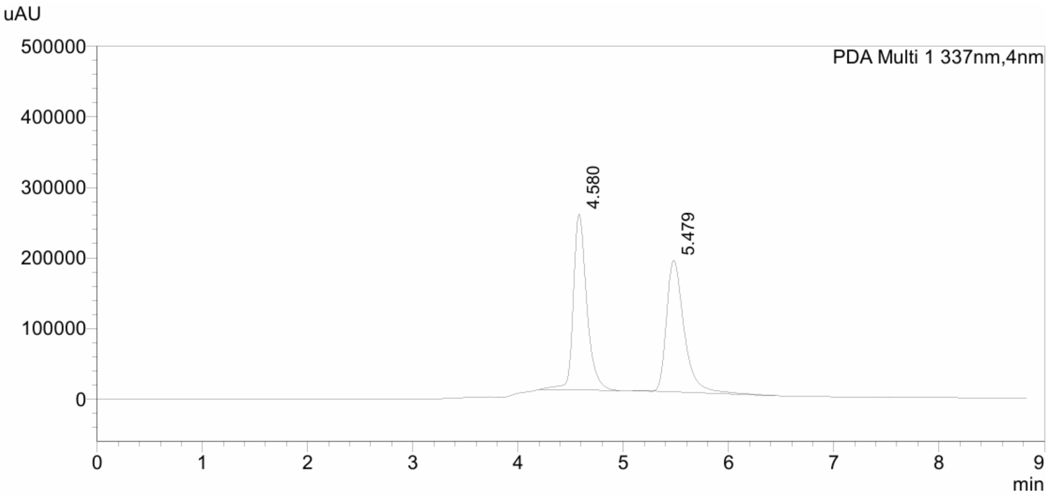

PDA Ch1 337nm

Peak#	Ret. Time	Area	Area%
1	4.580	2205160	50.274
2	5.479	2181162	49.726
Total		4386322	100.000

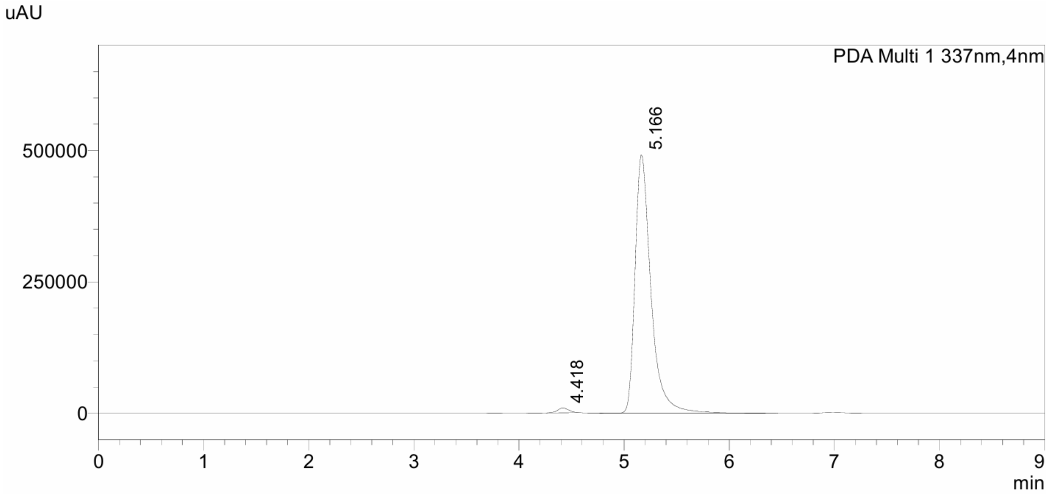

PDA Ch1 337nm

Peak#	Ret. Time	Area	Area%
1	4.418	71506	1.362
2	5.166	5177173	98.638
Total		5248679	100.000

Ethyl (3a*S*,4*R*,7*S*,7a*S*,8*R*)-5-amino-6-benzyl-8-methyl-1,3-dioxo-2-phenyl-1,2,3,3a,7,7a-hexahydro-4H-4,7-methanoisoindole-4-carboxylate (8)

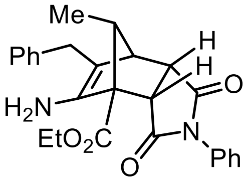

Cyclopentadiene **3a** (30 mg, 0.117 mmol, 1 eq) was charged in a 4 mL sealed vial under nitrogen atmosphere. The compound was diluted in dry toluene (0.2 M) and cooled to 0 °C. *N*-phenyl-maleimide (30 mg, 0.175 mmol, 1.5 eq) was added in one portion and the mixture was stirred for 20 hours while gradually warming to room temperature. After disappearance of the starting material (monitored by TLC), the mixture was filtered through a silica gel plug and the solvent was evaporated. The crude mixture was purified by silica gel flash column chromatography (hexane/EtOAc 7:3), affording the desired product (61 mg, 0.115 mmol, 99% yield, *er*=99:1, *dr*>20:1) as a white solid.

HPLC: Phenomenex cellulose-3, Hexane:2-propanol 40:60, flow: 1.0 mL/min, 248 nm, t_R = 11.8 min (major) and t_R = 23.0 min (minor).

^{1}H NMR (400 MHz, Chloroform-*d*) δ 7.47 (t, *J* = 7.6 Hz, 2H), 7.40 (d, *J* = 7.3 Hz, 1H), 7.28 (q, *J* = 8.0 Hz, 4H), 7.21 (t, *J* = 6.8 Hz, 3H), 4.36 (q, *J* = 7.1 Hz, 2H), 3.97 (d, *J* = 7.7 Hz, 1H), 3.61 (dd, *J* = 7.7, 4.3 Hz, 1H), 3.43 (d, *J* = 15.8 Hz, 1H), 3.07 (d, *J* = 15.7 Hz, 1H), 2.91 (d, *J* = 3.6 Hz, 1H), 2.30 (q, *J* = 6.0 Hz, 1H), 1.36 (t, *J* = 7.1 Hz, 3H), 0.85 (d, *J* = 6.3 Hz, 3H).

^{13}C NMR (101 MHz, Chloroform-*d*) δ 176.03, 174.76, 170.68, 139.26, 137.01, 132.06, 129.11, 128.96, 128.51, 128.48, 126.73, 126.16, 107.03, 65.45, 61.40, 58.60, 51.55, 50.85, 49.51, 32.68, 14.33, 10.07.

HRMS (ESI-TOF) m/z $[M]^{\cdot+}$ Calcd for $C_{26}H_{26}N_2O_4$ 430.1893; found 430.1875.

$[\alpha]^{25}_D$ = -51.0 (c = 1.0, $CHCl_3$).

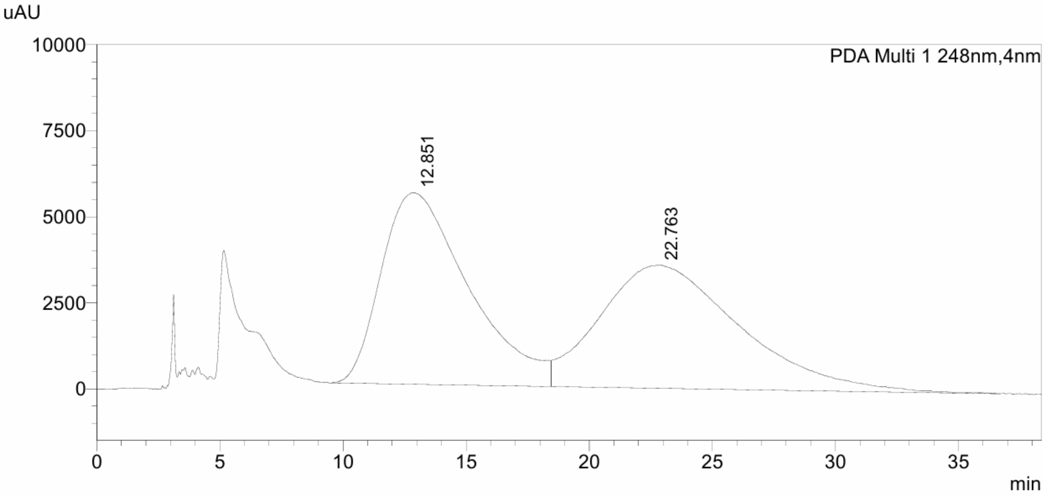

PDA Ch1 248nm

Peak#	Ret. Time	Area	Area%
1	12.851	1374056	49.272
2	22.763	1414673	50.728
Total		2788728	100.000

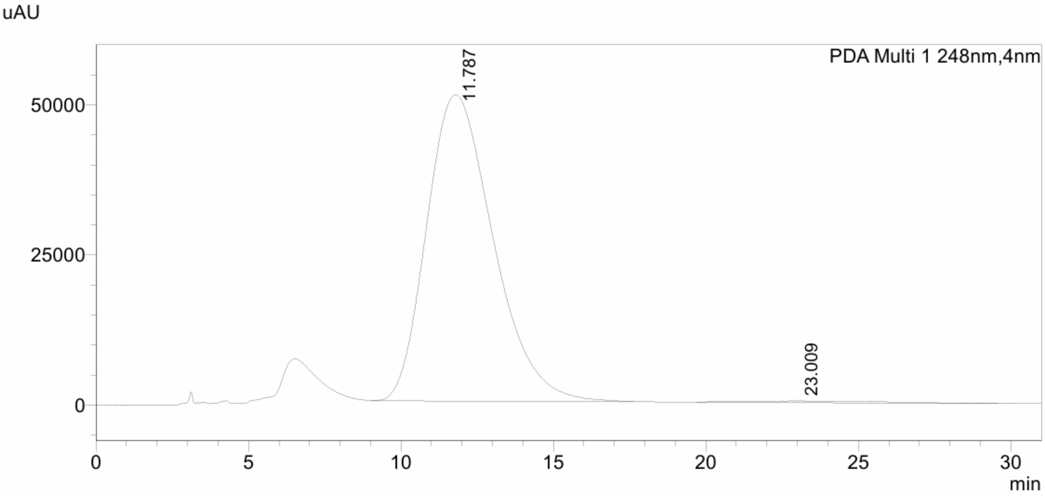

PDA Ch1 248nm

Peak#	Ret. Time	Area	Area%
1	11.787	7755036	98.812
2	23.009	93262	1.188
Total		7848298	100.000

Ethyl (1*S*,3*R*,4*S*,5*R*,7*R*)-5-acetyl-3-benzyl-7-methyl-2-oxobicyclo[2.2.1]heptane-1-carboxylate (9)

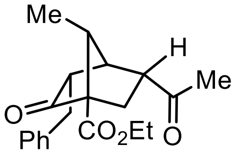

Cyclopentadiene **3a** (30 mg, 0.117 mmol, 1 eq) was charged in a 4 mL sealed vial under nitrogen atmosphere. Vinyl-methyl ketone (200 µL, 2.332 mmol, 20 eq) was cooled to 0 °C and added to the vial. The mixture was stirred at 0 °C for 8 hours, after which the reaction was quenched with HCl 1N and the product was extracted 3 times with EtOAc. The organic layers were collected and the solvent was evaporated under reduced pressure. Column chromatography (hexane/EtOAc 8:2) afforded the desired product (25 mg, 0.077 mmol, 66% yield, *er*=98.5:1.5, *dr*>20:1) as a colorless oil.

HPLC: Phenomenex cellulose-4, Hexane:2-propanol 96:4, flow: 1.0 mL/min, 206 nm, t_R = 40.0 min (minor) and t_R = 46.3 min (major).

^{1}H NMR (300 MHz, Chloroform-*d*) δ 7.49 – 7.27 (m, 2H), 7.22 (d, *J* = 7.4 Hz, 1H), 7.15 (d, *J* = 6.9 Hz, 2H), 4.27 (qd, *J* = 7.1, 2.3 Hz, 2H), 3.33 (dd, *J* = 13.7, 4.0 Hz, 1H), 3.04 (ddd, *J* = 10.3, 6.2, 3.8 Hz, 1H), 2.58 (dd, *J* = 10.1, 3.9 Hz, 2H), 2.53 (d, *J* = 1.9 Hz, 1H), 2.47 (dd, *J* = 9.0, 5.3 Hz, 1H), 2.21 (dd, *J* = 14.3, 10.5 Hz, 1H), 2.00 (dt, *J* = 11.8, 3.6 Hz, 1H), 1.57 (s, 3H), 1.40 – 1.25 (m, 6H).

^{13}C NMR (101 MHz, Chloroform-*d*) δ 209.40, 207.08, 169.35, 139.65, 128.86, 128.74, 126.55, 66.15, 60.99, 53.48, 49.23, 48.34, 45.19, 35.62, 28.35, 28.18, 14.34, 14.16.

HRMS (ESI-TOF) m/z $[M+Na]^+$ Calcd for $C_{20}H_{24}O_4Na$ 351.1572; found 351.1633.

$[\alpha]^{25}_D$ = -5.7 (c = 1.0, $CHCl_3$).

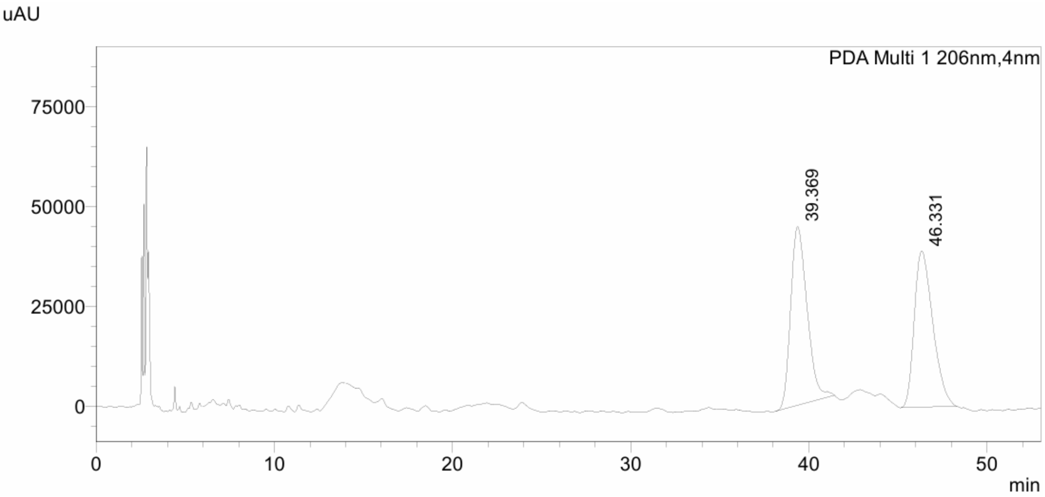

PDA Ch1 206nm

Peak#	Ret. Time	Area	Area%
1	39.369	2869945	50.349
2	46.331	2830197	49.651
Total		5700142	100.000

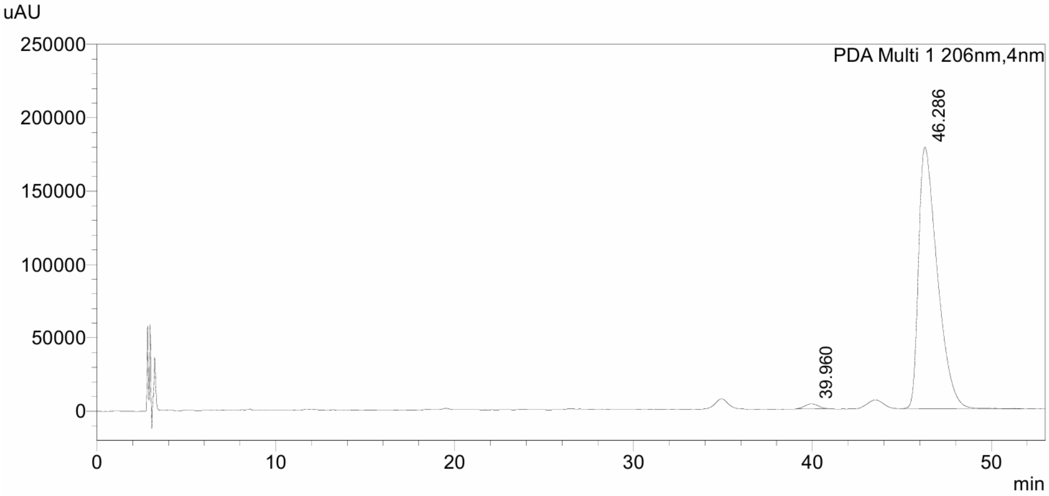

PDA Ch1 206nm

Peak#	Ret. Time	Area	Area%
1	39.960	176305	1.333
2	46.286	13045903	98.667
Total		13222208	100.000

Ethyl (1*S*,4*R*,5*R*)-3-benzyl-4-((dimethoxyphosphoryl)oxy)-1-hydroxy-5-methyl-2-oxocyclopentane-1-carboxylate (11)

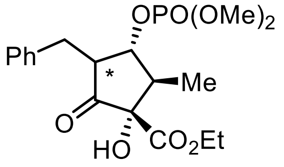

Cyclopentadiene **3a** (50 mg, 0.194 mmol, 1 eq) and tetraphenylporphyrine (1.19 mg, 0.002 mmol, 1 mol%) were charged in a 4 mL vial and $CHCl_3$ (0.05 M) was added. While bubbling O_2 through the solution, the reaction mixture was irradiated at 427 nm (Kessil lamp, I=25%) under stirring at room temperature for 5 minutes, after which irradiation was stopped. The reaction mixture was quenched with an excess of trimethyl phosphite (120 mg, 0.970 mmol, 5 eq) and stirred for 10 minutes at the same temperature. The mixture was filtered through a silica gel plug and the solvent was evaporated under reduced pressure. Column chromatography (hexane/EtOAc 4:6) afforded the desired product (28 mg, 0.070 mmol, 36% yield, *er*=98:2, *dr*=1:1) as a yellow oil.

HPLC analysis of the product: Phenomenex cellulose-4, Hexane:2-propanol 90:10, flow: 1.0 mL/min, 204 nm, t_R = 31.2, 33.2 min (major) and t_R = 36.3, 54.8 min (minor).

^{1}H NMR (400 MHz, Chloroform-*d*) δ 7.37 – 7.32 (m, 2H), 7.24 – 7.19 (m, 3H), 6.21 (dd, *J* = 26.2, 10.3 Hz, 1H), 4.78 (ddd, *J* = 25.0, 8.2, 3.9 Hz, 1H), 4.28 – 4.15 (m, 2H), 3.96 – 3.68 (m, 6H), 3.54 (d, *J* = 14.2 Hz, 2H), 3.42 – 3.32 (m, 1H), 1.31 (t, *J* = 7.1 Hz, 2H), 1.28 – 1.22 (m, 3H), 1.16 (d, *J* = 7.0 Hz, 1H).

^{13}C NMR (101 MHz, Chloroform-*d*) δ 168.40, 146.55, 145.49, 136.00, 135.92, 128.87 (d, *J* = 1.3 Hz), 128.80 (d, *J* = 7.8 Hz), 127.35, 127.34, 116.63, 116.40, 116.03, 78.15, 78.09, 62.03, 61.95, 54.87 (d, *J* = 3.7 Hz), 54.81 (d, *J* = 4.1 Hz), 54.69 (d, *J* = 1.6 Hz), 54.63 (d, *J* = 2.0 Hz), 40.60, 40.48, 39.51 (d, *J* = 6.5 Hz), 39.30 (d, *J* = 6.0 Hz), 16.69, 14.77, 14.14, 13.99.

^{31}P NMR (162 MHz, Chloroform-*d*) δ 0.99 – 0.24 (m).

^{31}P NMR, decoupled (162 MHz, Chloroform-*d*) δ 0.73, 0,47.

HRMS (ESI-TOF) m/z $[M+H]^+$ Calcd for $C_{18}H_{26}O_8P$ 401.1365; found 401.1366.

[α]$^{25}_{D}$ = -21.0 (c = 1.0, $CHCl_3$).

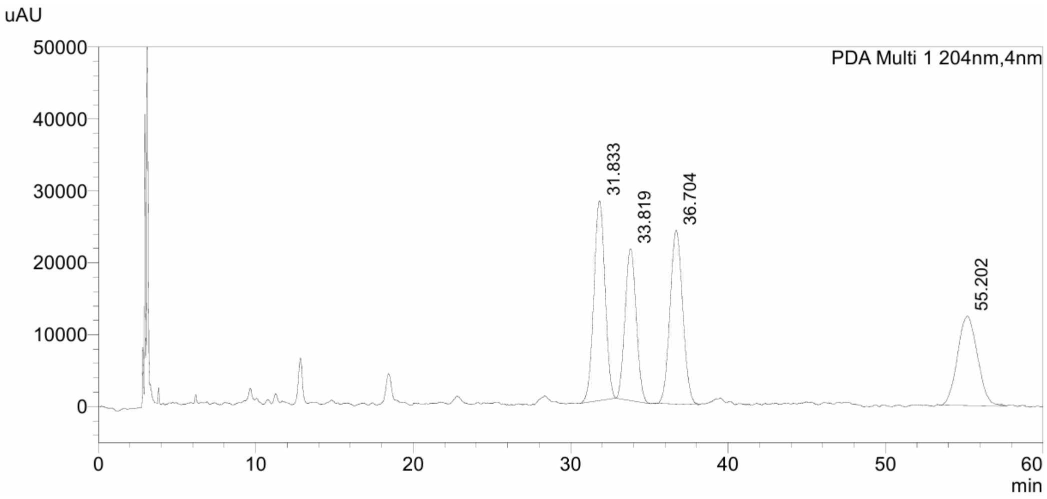

PDA Ch1 204nm

Peak#	Ret. Time	Area	Area%
1	31.833	1371388	28.159
2	33.819	1048879	21.537
3	36.704	1355681	27.836
4	55.202	1094288	22.469
Total		4870236	100.000

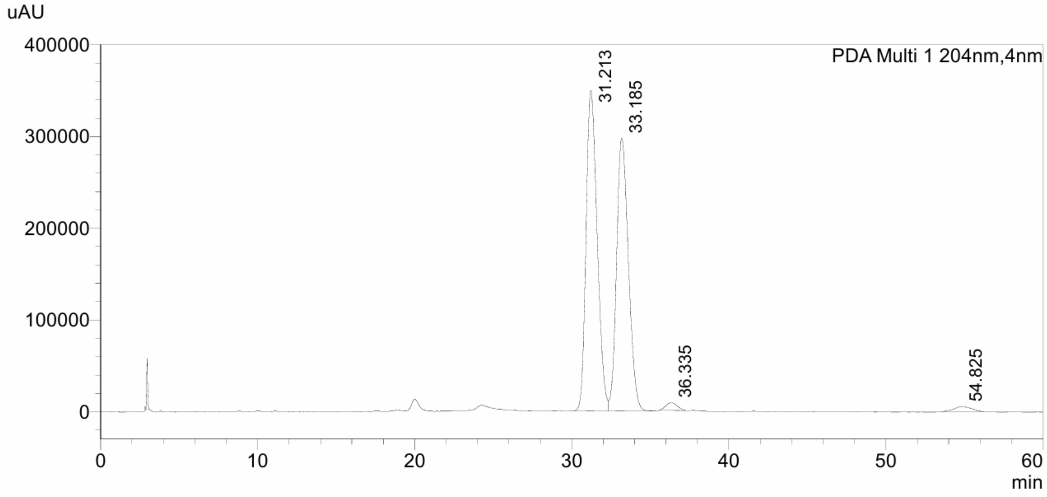

PDA Ch1 204nm

Peak#	Ret. Time	Area	Area%
1	31.213	17153311	51.063
2	33.185	15612418	46.476
3	36.335	420130	1.251
4	54.825	406361	1.210
Total		33592220	100.000

Ethyl (3*R*,5*R*)-2-amino-3-benzyl-5-methylcyclopent-1-ene-1-carboxylate (12)

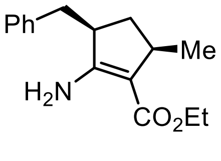

Cyclopentadiene **3a** (50 mg, 0.194 mmol, 1 eq) and Pd/C (10%w, 8 mg, 0.019 mmol, 10 mol%) were charged in a 4 mL sealed vial. Dry DCM (0.4 M) was added and the mixture was cooled to 0 °C. A H_2 atmosphere was applied through vacuum/H_2 cycles and the mixture was stirred at 0 °C for 16 hours. The reaction was filtered through a

short silica plug and characterized without further purification. The desired product (50 mg, 0.194 mmol, 99% yield, *er*=98.5:1.5, *dr*=9:1) was obtained as a pale yellow oil.

HPLC analysis of the product: Phenomenex cellulose-3, Hexane:2-propanol 90:10, flow: 1.0 mL/min, 277 nm, t_R = 5.8, 11.4 min (major) and t_R = 6.2, 7.4 min (minor).

^{1}H NMR ^{1}H NMR (400 MHz, Chloroform-*d*) δ 7.33 (t, *J* = 7.5 Hz, 2H), 7.25 (d, *J* = 6.3 Hz, 3H), 5.88 (d, *J* = 271.3 Hz, 2H), 4.29 – 4.09 (m, 2H), 3.03 – 2.91 (m, 2H), 2.88 (dd, *J* = 13.4, 6.6 Hz, 1H), 2.84 – 2.74 (m, 1H), 2.22 (dt, *J* = 13.3, 8.3 Hz, 1H), 1.30 (t, *J* = 7.1 Hz, 3H), 1.21 – 1.17 (m, 4H).

^{13}C NMR (101 MHz, Chloroform-*d*) δ 168.62, 163.76, 139.82, 128.96, 128.61, 126.44, 100.72, 58.62, 46.60, 40.97, 36.80, 35.53, 22.68, 14.61. (major)

HRMS (ESI-TOF) m/z [M+H]$^+$ Calcd for $C_{16}H_{22}NO_2$ 260.1651; found 260.1633.

[α]$^{25}_D$ = -16.0 (c = 1.0, $CHCl_3$).

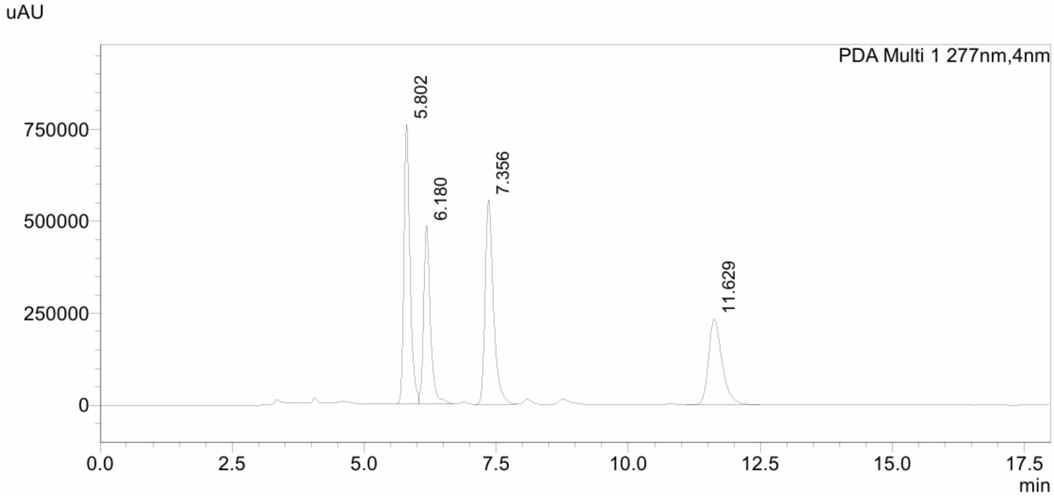

PDA Ch1 277nm

Peak#	Ret. Time	Area	Area%
1	5.802	6110925	29.489
2	6.180	4288642	20.695
3	7.356	6151816	29.686
4	11.629	4171346	20.129
Total		20722728	100.000

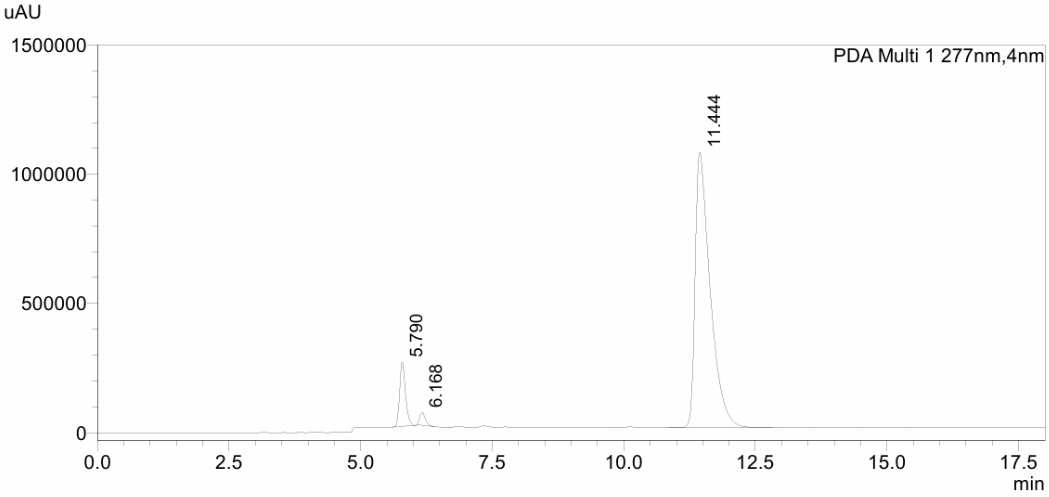

PDA Ch1 277nm

Peak#	Ret. Time	Area	Area%
1	5.790	1926678	8.255
2	6.168	363769	1.559
3	11.444	21048873	90.186
Total		23339319	100.000

Ethyl (3S,4R,5S)-2-amino-3-benzyl-3-chloro-5-methyl-4-((4-methylphenyl)sulfonamido) cyclopent-1-ene-1-carboxylate (13)

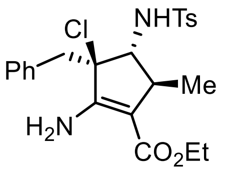

In a 4 mL sealed vial, cyclopentadiene **3a** (50 mg, 0.194 mmol, 1 eq) was diluted in a 2:1 solution of THF and water (0.2 M). Chloramine-T (66 mg, 0.291 mmol, 1.5 eq) was added in one portion and the mixture was stirred at room temperature for 12 hours. The reaction was quenched with a saturated solution of NH_4Cl and the product was extracted with EtOAc. The organic layers were collected and the solvent was evaporated under reduced pressure. Column chromatography (hexane/EtOAc 8:2) afforded the desired product (56 mg, 0.122 mmol, 63% yield, *er*=99:1, *dr*=70:30) as a pale yellow oil.

HPLC: Phenomenex cellulose-1, Hexane:2-propanol 90:10, flow: 1.0 mL/min, 300 nm, t_R = 29.7 min (minor) and t_R = 37.0 min (major).

^{1}H NMR (400 MHz, Chloroform-*d*) δ 7.79 (d, *J* = 8.2 Hz, 2H), 7.30 (t, *J* = 7.2 Hz, 7H), 5.67 (s, 2H), 5.20 (d, *J* = 10.0 Hz, 1H), 4.21 – 4.08 (m, 2H), 3.51 (dd, *J* = 10.0, 6.6 Hz, 1H), 3.39 (d, *J* = 14.7 Hz, 1H), 3.17 (d, *J* = 14.7 Hz, 1H), 2.59 (p, *J* = 6.6 Hz, 1H), 2.44 (s, 3H), 1.25 (t, *J* = 7.1 Hz, 3H), 0.79 (d, *J* = 6.6 Hz, 3H).

^{13}C NMR (101 MHz, $CDCl_3$) δ 167.40, 156.80, 143.61, 138.41, 134.40, 130.86, 129.67, 128.45, 127.56, 127.15, 99.98, 79.78, 63.22, 59.50, 44.33, 42.33, 21.55, 17.24, 14.32.

HRMS (ESI-TOF) m/z $[M+H]^+$ Calcd for $C_{23}H_{28}ClN_2O_4S$ 463.1458; found 463.1429.

$[\alpha]^{22}_D$ = -2.7 (c = 1.0, $CHCl_3$).

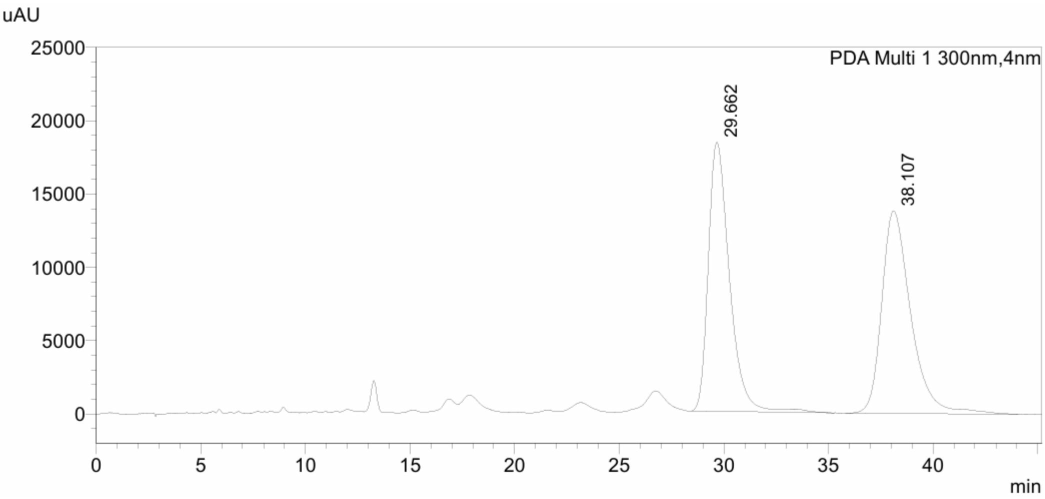

PDA Ch1 300nm

Peak#	Ret. Time	Area	Area%
1	29.662	1291878	49.164
2	38.107	1335824	50.836
Total		2627702	100.000

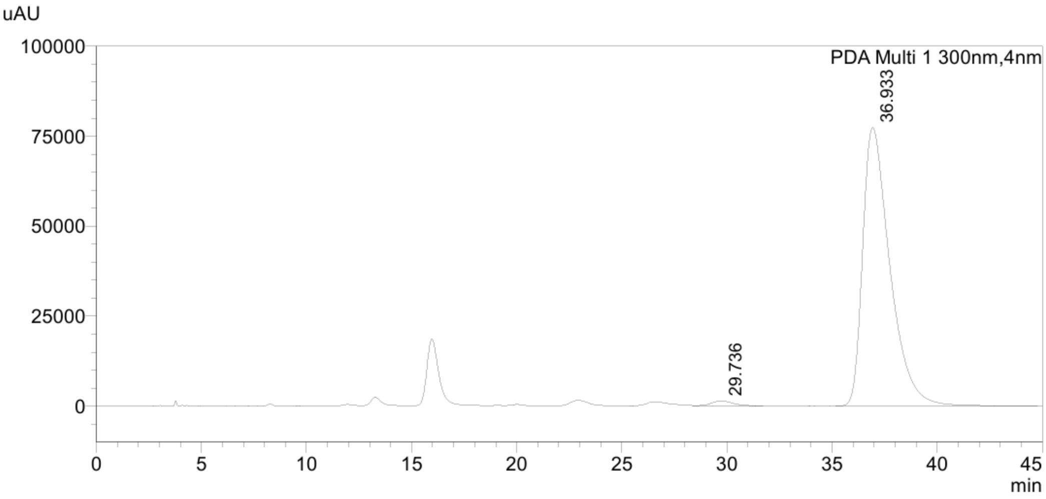

PDA Ch1 300nm

Peak#	Ret. Time	Area	Area%
1	29.736	91105	1.287
2	36.933	6986445	98.713
Total		7077550	100.000

Rhodium Cyclopentadienyl complex Rh(COD)($C_{16}H_{18}NO_2$) (14)

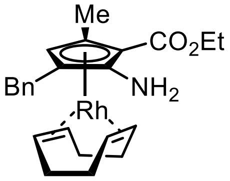

Prepared according to the procedure reported by Cramer and co-workers,[41] using cyclopentadiene **3a** (50 mg, 0.194 mmol, 1 eq), $[Rh(COD)Cl]_2$ (49 mg, 0.099 mmol, 0.51 eq) and KOAc (23 mg, 0.233 mmol, 1.2 eq). Purification through preparative HPLC (H_2O/CH_3CN from 100:0 to 35:65, 17 mL/min) afforded the desired product (37 mg, 0.080 mmol, 41% yield, *er*=97:3) as an orange solid.

HPLC: Phenomenex cellulose-1, Hexane:2-propanol 90:10, flow: 1.0 mL/min, 265 nm, t_R = 10.3 min (minor) and t_R = 13.3 min (major).

¹H NMR (300 MHz, Chloroform-*d*) δ 7.41 – 7.13 (m, 5H), 4.91 (s, 1H), 4.41 – 4.23 (m, 2H), 4.14 (br s, 2H), 3.49 (dd, *J* = 17.1 Hz, 2H), 3.32 (dt, *J* = 5.5, 2.2 Hz, 4H), 2.32 – 2.12 (m, 4H), 2.07 – 2.01 (m, 2H), 1.94 – 1.85 (m, 2H), 1.68 (s, 3H), 1.36 (t, *J* = 7.1 Hz, 3H).

¹³C NMR (101 MHz, Chloroform-*d*) δ 167.25, 138.90, 128.79, 128.50, 126.44, 88.55, 74.26 (d, *J* = 13.7 Hz), 73.54 (d, *J* = 13.7 Hz), 73.09 (d, *J* = 13.6 Hz), 72.30 (d, *J* = 13.5 Hz), 59.29, 32.94, 31.72, 31.38, 29.71, 14.75, 14.65, 12.34.

HRMS (ESI-TOF) m/z $[M+H]^+$ Calcd for $C_{24}H_{31}NO_2Rh$ 468.1410; found 468.1461.

$[\alpha]^{25}_D$ = -74.3 (c = 1.0, $CHCl_3$).

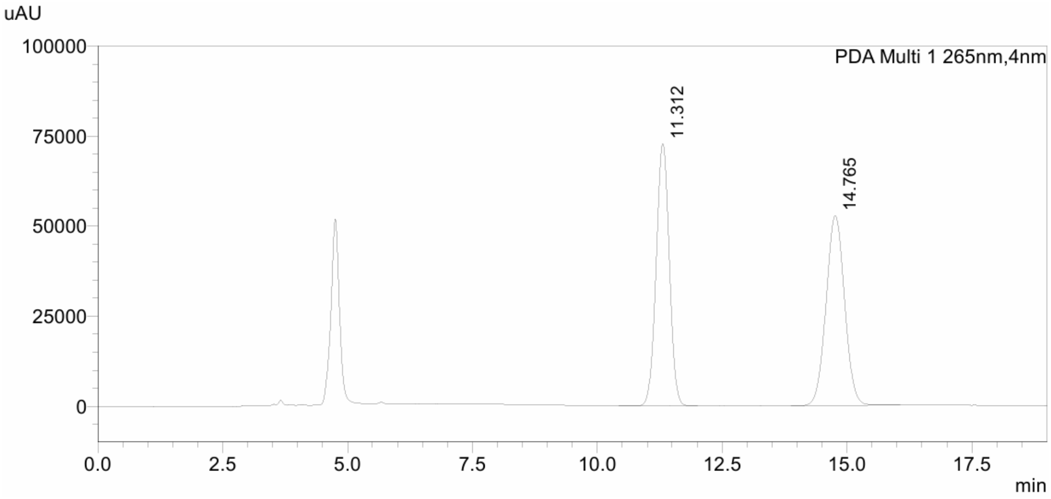

PDA Ch1 265nm

Peak#	Ret. Time	Area	Area%
1	11.312	1329955	49.421
2	14.765	1361128	50.579
Total		2691083	100.000

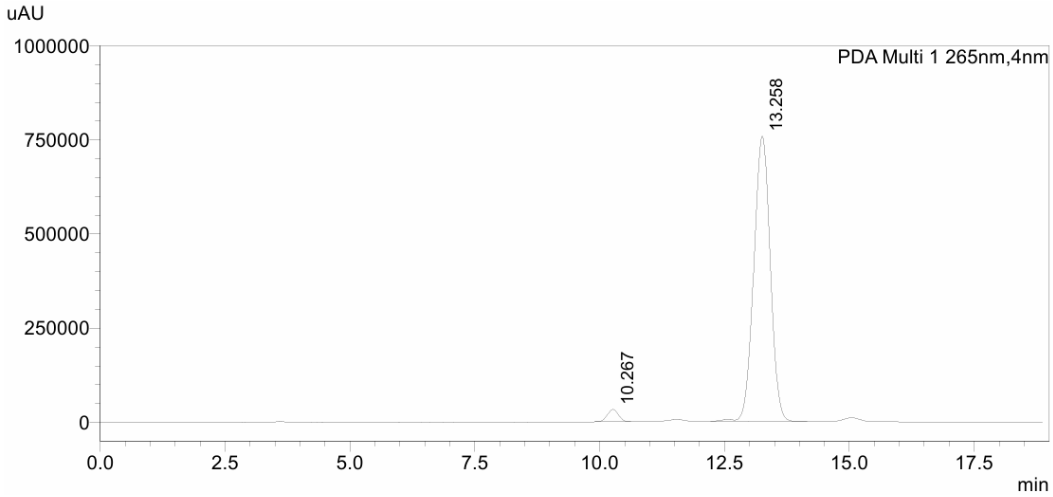

PDA Ch1 265nm

Peak#	Ret. Time	Area	Area%
1	10.267	507767	2.828
2	13.258	17447283	97.172
Total		17955050	100.000

7 X-Ray Crystallographic Analysis

7.1 CpH 3b

A translucent light colourless, needle-shaped crystal was mounted on the goniometer. Data were collected from a single crystal at 298.00 K on a Bruker D8 KAPPA diffractometer with a microfocus sealed tube using a multilayer mirror as monochromator and a PhotonIII_C14 CPAD detector. The diffractometer used CuK_α radiation (λ = 1.54178 Å). All data were integrated with SAINT V8.41, yielding 33785 reflections of which 2799 where independent and 99.2% were greater than 2σ(F^2).[42] A Multi-Scan absorption correction using SADABS 2016/2 was applied.[43] The structure was solved by dual methods with SHELXT 2018/2 and refined by full-matrix least-squares methods against F^2 using SHELXL 2018/3.[44,45] All non-hydrogen atoms were refined with anisotropic displacement parameters. All C-bound hydrogen atoms were refined isotropic on calculated positions using a riding model with their U_{iso} values constrained to 1.5 times the U_{eq} of their pivot atoms for terminal sp^3 carbon atoms and 1.2 times for all other carbon atoms. Crystallographic data for the structures reported in this paper have been deposited with the Cambridge Crystallographic Data Centre.[46] CCDC 2499110 contains the supplementary crystallographic data for this paper. These data can be obtained free of charge from The Cambridge Crystallographic Data Centre via www.ccdc.cam.ac.uk/structures.

CCDC number	2499110
Empirical formula	$C_{16}H_{18}BrNO_2$
Formula weight	336.22
Temperature [K]	298.00
Crystal system	trigonal
Space group (number)	$R3{:}H$ (146)
a [Å]	23.8128(9)
b [Å]	23.8128(9)
c [Å]	7.0664(4)
α [°]	90
β [°]	90
γ [°]	120
Volume [Å^3]	3470.2(3)
Z	9.0
ρ_{calc} [gcm^{-3}]	1.448
μ [mm^{-1}]	3.639
F(000)	1548
Crystal size [mm^3]	0.025×0.05×0.8
Crystal colour	translucent light colourless
Crystal shape	needle
Radiation	CuK_α (λ=1.54178 Å)
2θ range [°]	7.42 to 137.92 (0.83 Å)
Index ranges	−28 ≤ h ≤ 28<br>−28 ≤ k ≤ 28<br>−8 ≤ l ≤ 8
Reflections collected	33785
Independent reflections	2799<br>R_{int} = 0.0563<br>R_{sigma} = 0.0267
Completeness to θ = 67.679°	100.0
Data / Restraints / Parameters	2799 / 1 / 184
Goodness-of-fit on F^2	1.050
Final *R* indexes [I≥2σ(I)]	R_1 = 0.0252<br>wR_2 = 0.0677
Final *R* indexes [all data]	R_1 = 0.0253<br>wR_2 = 0.0678
Largest peak/hole [eÅ^{-3}]	0.36/−0.45
Flack X parameter	0.15(2)

Table S3. Crystal data and structure refinement of CpH **3b**.

The structure was refined as a 2-component inversion twin.

Atom	*x*	*y*	*z*	U_{eq} [a]
Br1	0.48283(2)	0.49905(2)	−0.50725(5)	0.05785(15)
O1	0.40194(11)	0.31058(12)	0.5189(4)	0.0432(5)
O2	0.48826(11)	0.29790(12)	0.5782(3)	0.0440(5)
N1	0.41238(15)	0.37676(17)	0.1620(4)	0.0454(7)
H1A	0.384387	0.358574	0.251092	0.055
H1B	0.403787	0.393915	0.067346	0.055
C1	0.61191(19)	0.4067(3)	0.3982(7)	0.0695(12)
H1C	0.613285	0.447220	0.418662	0.104
H1D	0.652862	0.414745	0.348964	0.104
H1E	0.603308	0.383688	0.515862	0.104
C2	0.55814(16)	0.36594(17)	0.2563(5)	0.0440(7)
H2	0.556293	0.324265	0.237581	0.053
C3	0.56864(17)	0.39928(17)	0.0696(5)	0.0489(8)
H3	0.605614	0.412894	−0.003899	0.059
C4	0.51912(15)	0.40730(14)	0.0204(4)	0.0385(6)
C5	0.46937(14)	0.37836(14)	0.1706(4)	0.0340(6)
C6	0.49162(14)	0.35393(14)	0.3084(4)	0.0350(6)
C7	0.45569(14)	0.31972(14)	0.4736(4)	0.0347(6)
C8	0.45800(17)	0.26264(16)	0.7489(5)	0.0415(7)
H8A	0.416875	0.224230	0.720318	0.050
H8B	0.450303	0.289584	0.835932	0.050
C9	0.5043(2)	0.2442(2)	0.8338(6)	0.0607(10)
H9A	0.511981	0.218160	0.745232	0.091
H9B	0.486008	0.219860	0.947771	0.091
H9C	0.544543	0.282671	0.862582	0.091
C10	0.51255(17)	0.43892(15)	−0.1563(5)	0.0404(7)
H10A	0.467410	0.416261	−0.194917	0.049
H10B	0.537429	0.433713	−0.256465	0.049
C11	0.53478(15)	0.51045(14)	−0.1366(5)	0.0366(6)
C12	0.52409(16)	0.54357(16)	−0.2804(5)	0.0418(6)
C13	0.5432(2)	0.60874(19)	−0.2678(7)	0.0588(10)
H13	0.535574	0.629556	−0.367414	0.071
C14	0.5735(2)	0.64212(19)	−0.1070(8)	0.0697(13)
H14	0.585737	0.685688	−0.095714	0.084
C15	0.5858(2)	0.6113(2)	0.0378(7)	0.0640(11)
H15	0.607021	0.634403	0.146021	0.077
C16	0.56675(16)	0.54621(18)	0.0242(5)	0.0473(8)
H16	0.575418	0.526038	0.123480	0.057

Table S4. Atomic coordinates and U_{eq} *[Å^2]* of **3b**. [a]U_{eq} is defined as 1/3 of the trace of the orthogonalized U_{ij} tensor.

Atom	U_{11}	U_{22}	U_{33}	U_{23}	U_{13}	U_{12}
Br1	0.0790(3)	0.0705(3)	0.0309(2)	0.00426(16)	−0.00047(17)	0.0425(2)
O1	0.0397(12)	0.0495(12)	0.0377(12)	0.0096(10)	0.0063(9)	0.0203(10)
O2	0.0439(11)	0.0517(12)	0.0378(12)	0.0131(10)	0.0036(9)	0.0250(10)
N1	0.0440(14)	0.0591(17)	0.0377(15)	0.0122(12)	0.0028(12)	0.0292(13)
C1	0.0387(18)	0.086(3)	0.065(3)	0.011(2)	−0.0047(18)	0.0168(19)

C2	0.0400(16)	0.0491(17)	0.0450(19)	0.0065(14)	0.0053(13)	0.0238(14)
C3	0.0445(17)	0.0572(19)	0.045(2)	0.0102(16)	0.0148(14)	0.0258(15)
C4	0.0431(15)	0.0351(14)	0.0318(15)	0.0038(11)	0.0073(12)	0.0154(12)
C5	0.0368(14)	0.0324(13)	0.0297(14)	0.0006(10)	0.0008(11)	0.0150(11)
C6	0.0336(14)	0.0355(14)	0.0332(15)	0.0027(11)	0.0022(11)	0.0152(11)
C7	0.0379(15)	0.0331(13)	0.0298(15)	0.0011(11)	−0.0029(11)	0.0152(11)
C8	0.0475(16)	0.0432(16)	0.0298(16)	0.0068(12)	−0.0005(13)	0.0197(13)
C9	0.070(2)	0.073(2)	0.047(2)	0.0125(19)	−0.0053(18)	0.042(2)
C10	0.0518(18)	0.0370(15)	0.0266(15)	0.0014(11)	0.0043(12)	0.0179(13)
C11	0.0350(13)	0.0368(14)	0.0315(16)	0.0015(11)	0.0104(11)	0.0130(12)
C12	0.0449(16)	0.0417(15)	0.0371(16)	0.0024(12)	0.0088(13)	0.0204(13)
C13	0.062(2)	0.0443(18)	0.072(3)	0.0091(18)	0.0112(19)	0.0276(17)
C14	0.061(2)	0.0365(18)	0.104(4)	−0.009(2)	0.007(2)	0.0179(17)
C15	0.051(2)	0.051(2)	0.073(3)	−0.023(2)	−0.0008(19)	0.0127(16)
C16	0.0401(16)	0.0478(17)	0.0428(18)	−0.0061(14)	0.0036(13)	0.0135(13)

Table S5. Anisotropic displacement parameters (Å^2) of **3b**. The anisotropic displacement factor exponent takes the form: $-2\pi^2[h^2(a^*)^2U_{11}+k^2(b^*)^2U_{22}+...+2hka^*b^*U_{12}]$.

Atom–Atom	Length [Å]
Br1–C12	1.902(4)
O1–C7	1.229(4)
O2–C7	1.349(4)
O2–C8	1.440(4)
N1–H1A	0.8600
N1–H1B	0.8600
N1–C5	1.340(4)
C1–H1C	0.9600
C1–H1D	0.9600
C1–H1E	0.9600
C1–C2	1.531(5)
C2–H2	0.9800
C2–C3	1.494(5)
C2–C6	1.508(4)
C3–H3	0.9300
C3–C4	1.332(5)
C4–C5	1.479(4)
C4–C10	1.506(5)
C5–C6	1.369(4)
C6–C7	1.436(4)
C8–H8A	0.9700
C8–H8B	0.9700
C8–C9	1.501(5)
C9–H9A	0.9600
C9–H9B	0.9600
C9–H9C	0.9600
C10–H10A	0.9700
C10–H10B	0.9700
C10–C11	1.516(4)
C11–C12	1.386(5)
C11–C16	1.395(5)
C12–C13	1.384(5)
C13–H13	0.9300
C13–C14	1.367(7)
C14–H14	0.9300
C14–C15	1.373(8)
C15–H15	0.9300
C15–C16	1.384(6)
C16–H16	0.9300

Atom–Atom–Atom	Angle [°]
C7–O2–C8	117.8(3)
H1A–N1–H1B	120.0
C5–N1–H1A	120.0
C5–N1–H1B	120.0
H1C–C1–H1D	109.5
H1C–C1–H1E	109.5
H1D–C1–H1E	109.5
C2–C1–H1C	109.5
C2–C1–H1D	109.5
C2–C1–H1E	109.5
C1–C2–H2	109.3
C3–C2–C1	112.1(3)
C3–C2–H2	109.3
C3–C2–C6	101.4(3)
C6–C2–C1	115.2(3)
C6–C2–H2	109.3
C2–C3–H3	123.8
C4–C3–C2	112.4(3)
C4–C3–H3	123.8
C3–C4–C5	107.8(3)
C3–C4–C10	127.5(3)
C5–C4–C10	124.6(3)

N1–C5–C4	123.0(3)
N1–C5–C6	128.4(3)
C6–C5–C4	108.5(3)
C5–C6–C2	109.8(3)
C5–C6–C7	124.9(3)
C7–C6–C2	125.3(3)
O1–C7–O2	122.5(3)
O1–C7–C6	125.8(3)
O2–C7–C6	111.7(3)
O2–C8–H8A	110.5
O2–C8–H8B	110.5
O2–C8–C9	106.2(3)
H8A–C8–H8B	108.7
C9–C8–H8A	110.5
C9–C8–H8B	110.5
C8–C9–H9A	109.5
C8–C9–H9B	109.5
C8–C9–H9C	109.5
H9A–C9–H9B	109.5
H9A–C9–H9C	109.5
H9B–C9–H9C	109.5
C4–C10–H10A	108.6
C4–C10–H10B	108.6
C4–C10–C11	114.8(3)
H10A–C10–H10B	107.5
C11–C10–H10A	108.6
C11–C10–H10B	108.6
C12–C11–C10	120.8(3)
C12–C11–C16	116.7(3)
C16–C11–C10	122.6(3)
C11–C12–Br1	119.4(2)
C13–C12–Br1	117.9(3)
C13–C12–C11	122.6(4)
C12–C13–H13	120.4
C14–C13–C12	119.2(4)
C14–C13–H13	120.4
C13–C14–H14	120.0
C13–C14–C15	120.0(4)
C15–C14–H14	120.0
C14–C15–H15	119.7
C14–C15–C16	120.6(4)
C16–C15–H15	119.7
C11–C16–H16	119.6
C15–C16–C11	120.9(4)
C15–C16–H16	119.6

Table S6. Bond lengths and angles of **3b**.

Atom–Atom–Atom–Atom	Torsion Angle [°]
Br1–C12–C13–C14	−179.5(3)
N1–C5–C6–C2	178.9(3)
N1–C5–C6–C7	1.3(5)
C1–C2–C3–C4	−122.2(4)
C1–C2–C6–C5	120.4(4)
C1–C2–C6–C7	−62.0(5)
C2–C3–C4–C5	−1.1(4)
C2–C3–C4–C10	179.7(3)
C2–C6–C7–O1	178.8(3)
C2–C6–C7–O2	−1.1(4)
C3–C2–C6–C5	−0.9(4)
C3–C2–C6–C7	176.7(3)
C3–C4–C5–N1	−178.2(3)
C3–C4–C5–C6	0.5(4)
C3–C4–C10–C11	−93.5(4)
C4–C5–C6–C2	0.3(3)
C4–C5–C6–C7	−177.3(3)
C4–C10–C11–C12	−172.0(3)
C4–C10–C11–C16	8.2(5)
C5–C4–C10–C11	87.5(4)
C5–C6–C7–O1	−3.9(5)
C5–C6–C7–O2	176.2(3)
C6–C2–C3–C4	1.3(4)
C7–O2–C8–C9	179.5(3)
C8–O2–C7–O1	0.0(4)
C8–O2–C7–C6	179.9(3)
C10–C4–C5–N1	1.0(5)
C10–C4–C5–C6	179.7(3)
C10–C11–C12–Br1	−1.5(4)
C10–C11–C12–C13	179.6(3)
C10–C11–C16–C15	−179.3(3)
C11–C12–C13–C14	−0.5(6)
C12–C11–C16–C15	0.8(5)
C12–C13–C14–C15	1.3(7)
C13–C14–C15–C16	−1.1(7)
C14–C15–C16–C11	0.0(6)
C16–C11–C12–Br1	178.4(2)
C16–C11–C12–C13	−0.6(5)

Table S7. Torsion angles of **3b**.

7.2 CpH 3f

A translucent light colourless-gold, block-shaped crystal was mounted on the goniometer. Data were collected from a single crystal at 298.00 K on a Bruker D8 KAPPA diffractometer with a microfocus sealed tube using a multilayer mirror as monochromator and a PhotonIII_C14 CPAD detector. The diffractometer used CuK_α radiation (λ = 1.54178 Å). All data were integrated with SAINT V8.41, yielding 35168 reflections of which 3857 where independent and 99.1% were greater than 2σ(F^2).[42] A Multi-Scan absorption correction using TWINABS Bruker was applied.[43] The structure was solved by Intrinsic Phasing methods with XT, VERSION 2018/2 and refined by full-matrix least-squares methods against F^2 using SHELXL-2019/2.[44,45] All non-hydrogen atoms were refined with anisotropic displacement parameters. All C-bound hydrogen atoms were refined isotropic on calculated positions using a riding model with their U_{iso} values constrained to 1.5 times the U_{eq} of their pivot atoms for terminal sp^3 carbon atoms and 1.2 times for all other carbon atoms. Crystallographic data for the structures reported in this paper have been deposited with the Cambridge Crystallographic Data Centre.[46] CCDC 2499127 contains the supplementary crystallographic data for this paper. These data can be obtained free of charge from The Cambridge Crystallographic Data Centre via www.ccdc.cam.ac.uk/structures.

CCDC number	2499127
Empirical formula	$C_{22}H_{23}NO_2$
Formula weight	333.41
Temperature [K]	273(2)
Crystal system	orthorhombic
Space group (number)	$P2_12_12_1$ (19)
a [Å]	22.132(3)
b [Å]	8.5694(11)
c [Å]	9.9736(13)
α [°]	90
β [°]	90
γ [°]	90
Volume [Å^3]	1891.6(4)
Z	4
ρ_{calc} [gcm^{-3}]	1.171
μ [mm^{-1}]	0.587
F(000)	712
Crystal size [mm^3]	0.424×0.495×0.663
Crystal colour	translucent light colourless-gold
Crystal shape	block
Radiation	CuK_α (λ=1.54178 Å)
2θ range [°]	9.73 to 149.45 (0.80 Å)
Index ranges	$-27 \le h \le 27$<br>$-10 \le k \le 10$<br>$-12 \le l \le 12$
Reflections collected	35168
Independent reflections	3857<br>R_{int} = 0.054<br>R_{sigma} = 0.0540
Completeness to θ = 67.679°	99.6
Data / Restraints / Parameters	3857 / 0 / 229
Goodness-of-fit on F^2	1.358
Final R indexes [I≥2σ(I)]	R_1 = 0.0438<br>wR_2 = 0.1428
Final R indexes [all data]	R_1 = 0.0442<br>wR_2 = 0.1434
Largest peak/hole [eÅ^{-3}]	0.27/−0.23
Extinction coefficient	0.023(4)
Flack X parameter	-0.08(19)

Table S8. Crystal data and structure refinement of CpH **3f**.

Atom	*x*	*y*	*z*	U_{eq}
O2	0.37943(6)	0.72603(16)	0.08333(13)	0.0484(4)
O3	0.29396(6)	0.58349(18)	0.08962(14)	0.0495(4)
N1	0.27818(6)	0.40895(19)	0.32777(15)	0.0458(4)
H1A	0.257711	0.437048	0.258564	0.055
H1B	0.261652	0.351776	0.388415	0.055
C1	0.39810(7)	0.31014(19)	0.68716(16)	0.0387(4)
C2	0.38743(11)	0.3852(2)	0.8072(2)	0.0573(5)
H2	0.351208	0.438126	0.820120	0.069
C3	0.43034(15)	0.3826(3)	0.9089(2)	0.0776(8)
H3	0.422607	0.433896	0.989248	0.093
C4	0.48399(13)	0.3049(3)	0.8919(2)	0.0680(6)
H4	0.513183	0.306225	0.958925	0.082
C5	0.49396(10)	0.2259(3)	0.7759(2)	0.0638(6)
H5	0.529688	0.170186	0.765051	0.077
C6	0.45160(9)	0.2273(3)	0.67331(19)	0.0544(5)
H6	0.459125	0.172192	0.594707	0.065
C7	0.35165(7)	0.31647(18)	0.57492(15)	0.0362(3)
H7	0.315896	0.368703	0.611591	0.043
C8	0.33211(8)	0.15129(19)	0.53678(16)	0.0419(4)
C9	0.35087(11)	0.0795(3)	0.4194(2)	0.0591(5)
H9	0.375360	0.132975	0.359106	0.071
C10	0.33288(16)	−0.0732(3)	0.3920(3)	0.0838(9)
H10	0.345465	−0.121194	0.313229	0.101
C11	0.29693(17)	−0.1529(3)	0.4799(4)	0.0911(10)
H11	0.285515	−0.254975	0.460954	0.109
C12	0.27722(15)	−0.0814(3)	0.5985(4)	0.0839(9)
H12	0.252665	−0.135082	0.658463	0.101
C13	0.29509(10)	0.0715(2)	0.6248(2)	0.0591(5)
H13	0.281921	0.120548	0.702697	0.071
C14	0.37335(7)	0.41493(18)	0.45909(15)	0.0367(4)
C15	0.36920(7)	0.54529(18)	0.25436(15)	0.0364(4)
C16	0.43023(7)	0.5775(2)	0.31567(16)	0.0423(4)
H16	0.432655	0.688832	0.337425	0.051
C17	0.42657(7)	0.4872(2)	0.44393(17)	0.0429(4)
H17	0.457797	0.481845	0.506169	0.052
C18	0.34364(7)	0.61653(18)	0.13734(16)	0.0378(4)
C19	0.35601(10)	0.8086(3)	−0.0325(2)	0.0564(5)
H19A	0.313636	0.833197	−0.019475	0.068
H19B	0.359798	0.744391	−0.112161	0.068
C20	0.39127(16)	0.9531(4)	−0.0483(4)	0.0953(11)
H20A	0.433423	0.927891	−0.054935	0.143
H20B	0.384782	1.019301	0.028054	0.143
H20C	0.378592	1.006349	−0.128131	0.143
C21	0.48510(8)	0.5339(4)	0.2319(2)	0.0660(6)
H21A	0.482856	0.425560	0.207890	0.099
H21B	0.521195	0.552217	0.282891	0.099
H21C	0.485882	0.596375	0.152025	0.099
C22	0.33615(6)	0.45454(17)	0.34121(14)	0.0342(3)

Table S9. Atomic coordinates and U_{eq} [Å^2] of **3f**. $^{a}U_{eq}$ is defined as 1/3 of the trace of the orthogonalized U_{ij} tensor.

Atom	U_{11}	U_{22}	U_{33}	U_{23}	U_{13}	U_{12}
O2	0.0463(6)	0.0541(7)	0.0447(6)	0.0120(5)	−0.0074(5)	−0.0079(5)
O3	0.0392(6)	0.0616(7)	0.0476(7)	0.0096(5)	−0.0128(5)	−0.0043(5)
N1	0.0328(6)	0.0582(8)	0.0464(7)	0.0084(6)	−0.0054(5)	−0.0079(6)
C1	0.0415(8)	0.0395(7)	0.0351(7)	0.0026(6)	−0.0021(6)	0.0011(6)
C2	0.0662(12)	0.0574(10)	0.0483(9)	−0.0112(8)	−0.0116(8)	0.0169(9)
C3	0.1002(19)	0.0797(15)	0.0529(11)	−0.0193(11)	−0.0286(13)	0.0229(14)
C4	0.0787(14)	0.0681(13)	0.0572(12)	0.0060(10)	−0.0288(11)	0.0041(11)
C5	0.0495(10)	0.0805(14)	0.0616(12)	0.0131(11)	−0.0083(9)	0.0143(10)
C6	0.0506(10)	0.0699(11)	0.0427(8)	0.0004(8)	−0.0011(7)	0.0168(9)
C7	0.0360(7)	0.0385(7)	0.0340(7)	−0.0004(6)	−0.0003(5)	0.0030(6)
C8	0.0436(8)	0.0382(7)	0.0438(8)	−0.0009(6)	−0.0043(6)	0.0026(6)
C9	0.0701(12)	0.0506(10)	0.0565(10)	−0.0107(8)	0.0006(9)	0.0080(9)
C10	0.105(2)	0.0539(12)	0.092(2)	−0.0258(12)	−0.0191(16)	0.0131(14)
C11	0.106(2)	0.0425(11)	0.125(3)	−0.0071(14)	−0.027(2)	−0.0060(12)
C12	0.0884(18)	0.0514(12)	0.112(2)	0.0181(14)	−0.0116(17)	−0.0225(12)
C13	0.0651(12)	0.0486(10)	0.0635(12)	0.0074(9)	0.0005(9)	−0.0066(9)
C14	0.0361(7)	0.0397(7)	0.0341(7)	−0.0001(6)	−0.0035(6)	0.0011(6)
C15	0.0331(7)	0.0413(7)	0.0347(7)	0.0022(6)	−0.0044(5)	−0.0020(5)
C16	0.0364(8)	0.0523(8)	0.0382(7)	0.0056(7)	−0.0077(6)	−0.0108(6)
C17	0.0382(8)	0.0538(9)	0.0369(7)	0.0036(6)	−0.0083(6)	−0.0056(7)
C18	0.0363(7)	0.0409(7)	0.0363(7)	0.0002(6)	−0.0017(6)	0.0007(6)
C19	0.0592(11)	0.0610(11)	0.0491(9)	0.0183(8)	−0.0097(8)	−0.0051(9)
C20	0.0883(18)	0.0880(19)	0.110(2)	0.0522(19)	−0.0275(17)	−0.0263(15)
C21	0.0342(9)	0.1133(18)	0.0507(10)	0.0106(12)	−0.0019(7)	−0.0011(10)
C22	0.0314(7)	0.0363(7)	0.0348(7)	−0.0007(5)	−0.0027(5)	0.0011(5)

Table S10. Anisotropic displacement parameters (Å^2)of **3f**. The anisotropic displacement factor exponent takes the form: $-2\pi^2[h^2(a^*)^2U_{11}+k^2(b^*)^2U_{22}+...+2hka^*b^*U_{12}]$.

Atom–Atom	Length [Å]
O2–C18	1.341(2)
O2–C19	1.451(2)
O3–C18	1.231(2)
N1–C22	1.3480(19)
C1–C2	1.379(3)
C1–C6	1.388(2)
C1–C7	1.521(2)
C2–C3	1.390(3)
C3–C4	1.372(4)
C4–C5	1.358(4)
C5–C6	1.388(3)
C7–C14	1.509(2)
C7–C8	1.528(2)
C8–C13	1.382(3)
C8–C9	1.386(3)
C9–C10	1.395(3)
C10–C11	1.367(5)
C11–C12	1.401(6)
C12–C13	1.394(3)
C14–C17	1.339(2)
C14–C22	1.475(2)
C15–C22	1.375(2)
C15–C18	1.433(2)
C15–C16	1.5081(19)
C16–C17	1.497(2)
C16–C21	1.521(3)
C19–C20	1.472(4)

Atom–Atom–Atom	Angle [°]
C18–O2–C19	116.77(14)
C2–C1–C6	118.12(17)
C2–C1–C7	120.42(15)
C6–C1–C7	121.45(15)
C1–C2–C3	120.6(2)
C4–C3–C2	120.6(2)
C5–C4–C3	119.2(2)
C4–C5–C6	120.93(19)
C1–C6–C5	120.48(19)
C14–C7–C1	111.61(13)
C14–C7–C8	114.67(13)
C1–C7–C8	109.97(12)

C13–C8–C9	119.66(19)
C13–C8–C7	117.89(16)
C9–C8–C7	122.44(16)
C8–C9–C10	119.7(3)
C11–C10–C9	120.7(3)
C10–C11–C12	120.3(2)
C13–C12–C11	118.8(3)
C8–C13–C12	120.9(3)
C17–C14–C22	107.13(14)
C17–C14–C7	128.67(14)
C22–C14–C7	124.15(13)
C22–C15–C18	122.96(14)
C22–C15–C16	108.93(13)

C18–C15–C16	127.27(14)
C17–C16–C15	101.74(12)
C17–C16–C21	112.68(16)
C15–C16–C21	116.59(15)
C14–C17–C16	112.51(14)
O3–C18–O2	122.22(15)
O3–C18–C15	124.70(15)
O2–C18–C15	113.08(14)
O2–C19–C20	107.81(19)
N1–C22–C15	127.42(14)
N1–C22–C14	122.95(14)
C15–C22–C14	109.61(13)

Table S11. Bond lengths and angles of **3f**.

8 Deuterium Labelling Experiment

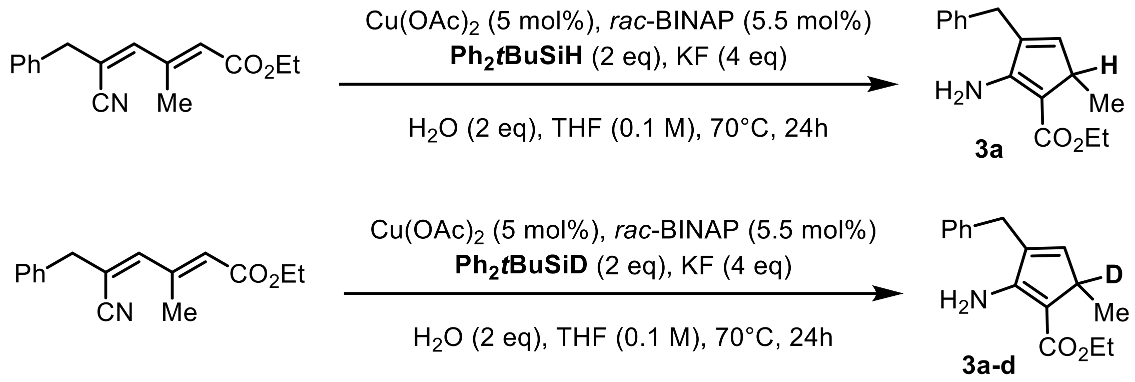

***tert*-butyldiphenylsilane-d**

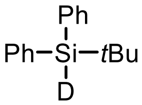

$LiAlD_4$ (55 mg, 1.46 mmol) was suspended in dry THF (1.5 mL) under nitrogen atmosphere and cooled to 0°C. Ph_2*t*BuSiCl (187 µL, 0.73 mmol) was added dropwise and the mixture was warmed at room temperature. After stirring for 30 minutes, the mixture was filtered through silica plug and the solvent was evaporated under reduced pressure, affording the desired product (99%, 99%-D) as colorless oil. The ^{1}H-NMR data matches the assigned structure and is consistent with literature values.[47]

^{1}H-NMR (400 MHz, Chloroform-*d*)

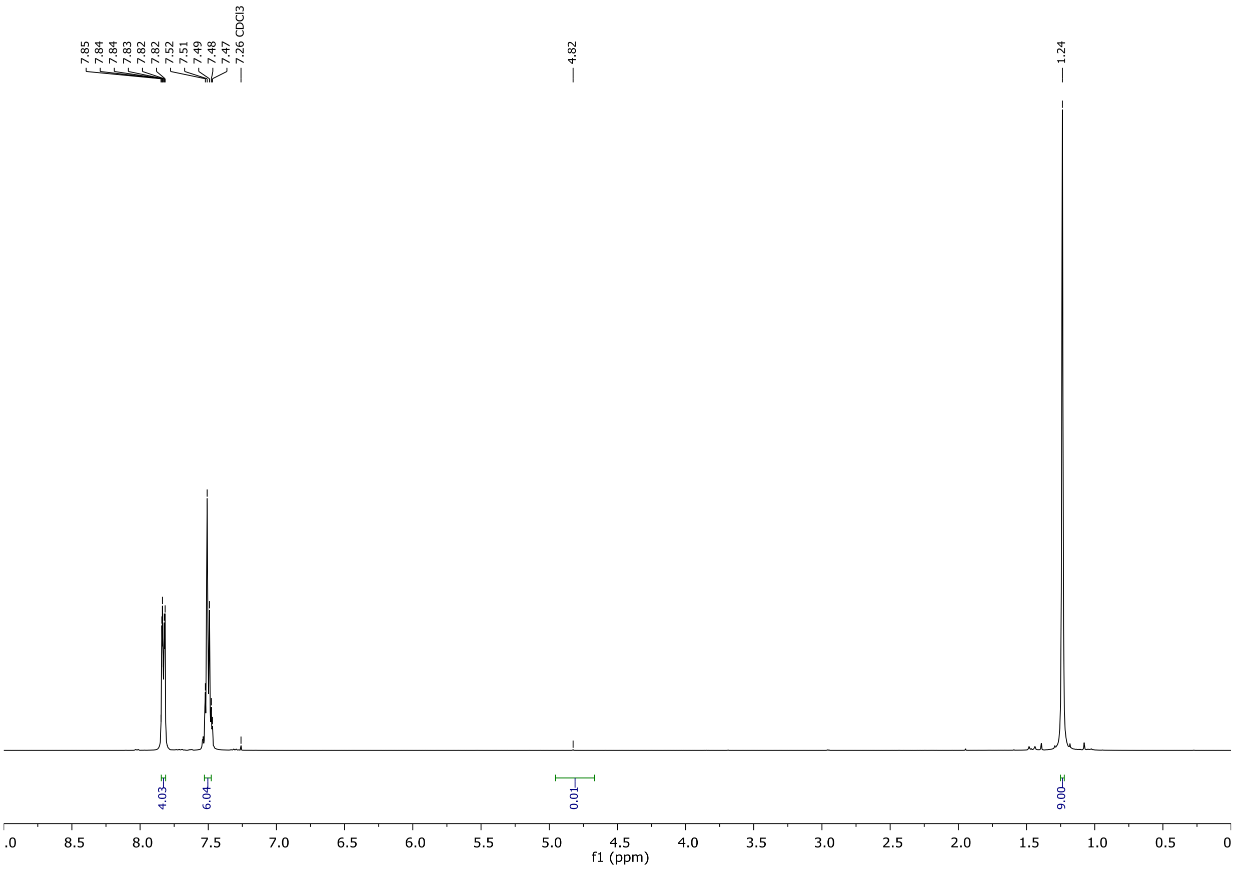

Ethyl 2-amino-3-benzyl-5-methylcyclopenta-1,3-diene-1-carboxylate-5-d (3a-d)

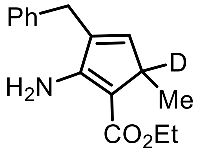

Prepared according to **General Procedure H**, but heating at 70 °C for 24h, using **1a** (65 mg, 0.255 mmol), $Cu(OAc)_2$ (2.31 mg, 12.7 μmol), rac-BINAP (8.71 mg, 14.0 μmol), Ph_2*t*BuSiD (123 mg, 0.509 mmol), KF (59 mg, 1.02 mmol) and H_2O (9 μL, 0.509 mmol) in THF (2.5 mL). Purification by silica gel flash column chromatography (hexane/EtOAc 9:1) provided the title compound (21 mg, 0.082 mmol, 32% yield, 91%-D) as a colorless oil.

^{1}H NMR (400 MHz, Chloroform-*d*) δ 7.3 (t, *J*=7.4 Hz, 2H), 7.3 (m, 3H), 6.2 (s, 1H), 5.5 (br s, 1H), 4.2 (dtd, *J*=18.0, 10.7, 7.1 Hz, 2H), 3.7 (s, 2Hs), 3.3 (q, *J*=6.9 Hz, 0.09H), 1.3 (t, *J*=7.1 Hz, 3H), 1.2 (s, 1H)

^{13}C NMR (75 MHz, Chloroform-*d*) δ 166.8, 160.1, 145.0, 137.8, 137.4, 128.8, 128.6, 126.7, 103.4, 58.5, 45.5 – 40.0 (t, *J* = 19.7 Hz), 33.1, 15.8, 14.8

HRMS (ESI-TOF) m/z $[M+H]^+$ Calcd for $C_{16}H_{18}DNO_2$ 259.1557; found 259.1540.

9 Computational Details

9.1 Reaction mechanism

All calculations were carried out using the *Gaussian 16*[48] suite of programs at the C_3P facility of the University of Padova, while visualization of the optimized structures was performed with *CYLView*[49]. For the mechanistic investigation of the hydrocupration/Dieckmann–Thorpe cascade reaction, conformational analyses were conducted manually due to the relatively high rigidity of the system. Geometry optimizations and conformational analyses were performed at the ωB97XD/def2SV level of theory in the gas phase. The ωB97XD functional was selected for its proven reliability in describing systems containing transition metals, as it includes long-range exchange interactions and empirical dispersion corrections to account for weak noncovalent interactions, which are crucial in asymmetric catalysis[50-52]. Stationary points on the potential energy surface (PES) were characterized as minima (no imaginary frequencies) or transition states (TS, one imaginary frequency) by vibrational frequency analysis at the same level of theory. Thermal corrections were obtained from the vibrational analysis on the optimized geometries. Single-point energy (SPE) calculations were then performed at the ωB97XD/def2TZVPP level of theory, including solvation effects via the SMD implicit solvation model for tetrahydrofuran (THF), the same solvent employed in the experimental hydrocupration/Dieckmann–Thorpe cascade reaction. The computed reaction energy profile is shown in Figure S1, while Figure S2 provides, as an example, the equilibria between intermediates $\mathbf{B}^{S}$ and $\mathbf{C}^{S}$. The corresponding electronic energies of all optimized structures are summarized in Table S12, and the Cartesian coordinates of all optimized geometries are reported in Section 8.5.

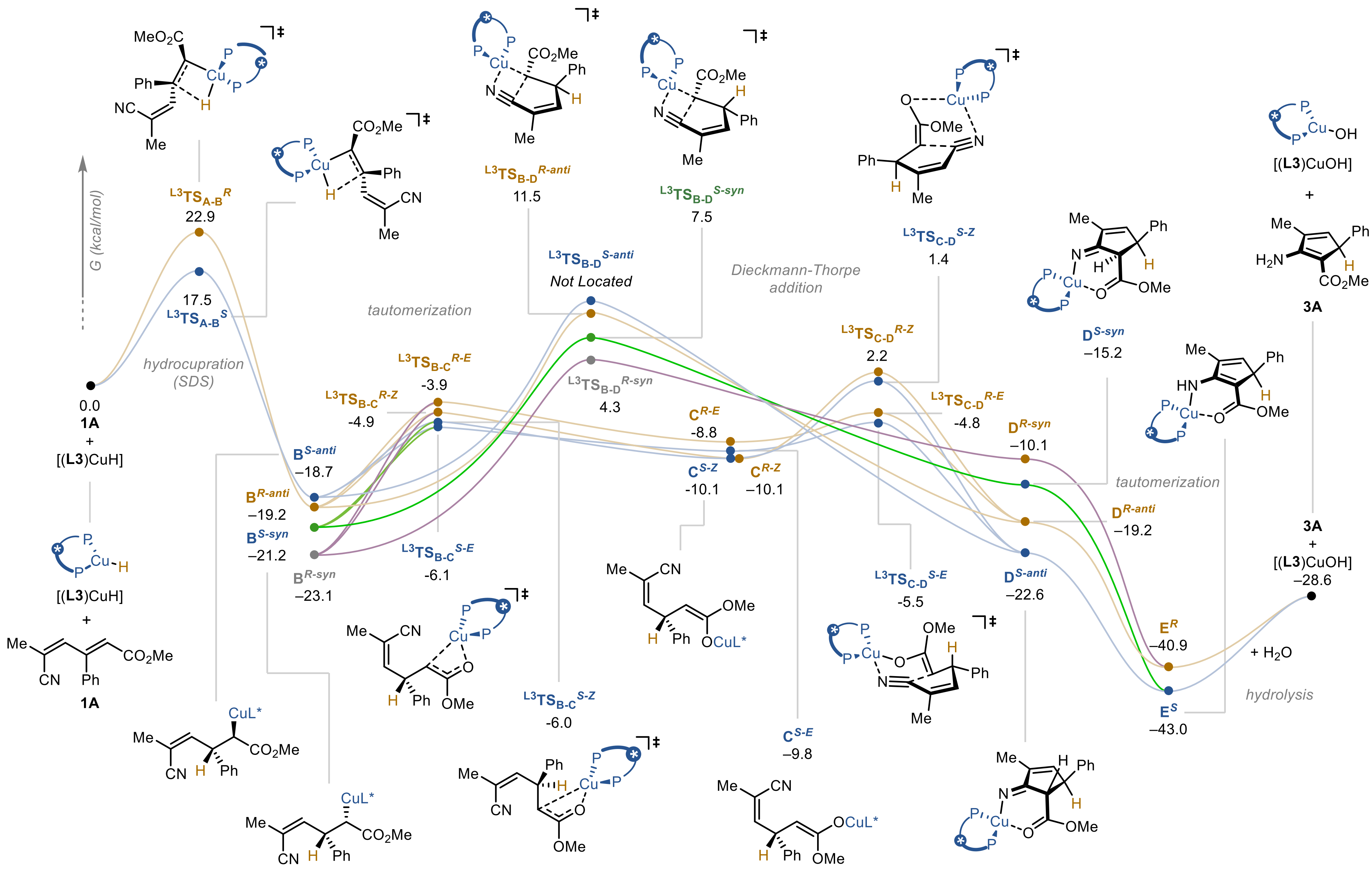

Figure S1. Reaction energy profile of the hydrocupration/Dieckmann–Thorpe reaction sequence.

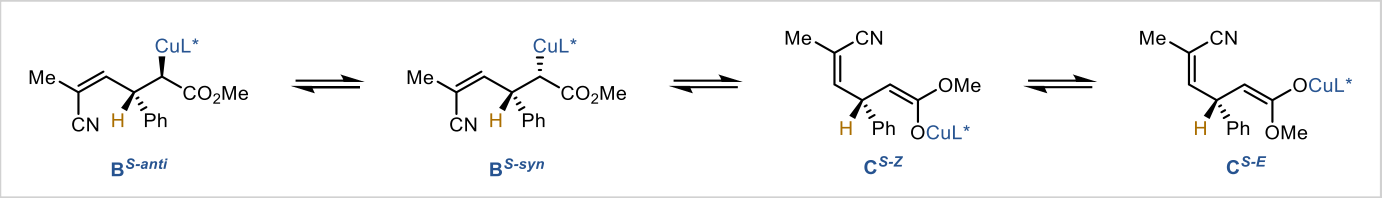

Figure S2. Equilibria between intermediates **B**^{S} and **C**^{S}. Isomers **B** equilibrate via *O*-bound enolates **C**.

	ωB97XD/def2SV		*ωB97XD/def2TZVPP*		
**Conformer**	**Energy (Hartree)**	**Thermal correction to Gibbs Free Energy (Hartree)**	**Electronic energy (SMD = THF, Hartree)**	**Gibbs Free Energy (Hartree)**	**ΔG (kcal/mol)**
[(**L3**)CuH]	-3639.373076	0.557531	-3641.360807	-3640.803276	/
**1A**	-745.657657	0.192425	-746.525104	-746.332679	/
[(**L3**)CuH]+**1A**	-4.385.030733	0.749956	-4387.885911	-4387.135955	0.00
$^{L3}\mathbf{TS}_{A\text{-}B}{}^{R}$	-4385.061287	0.782235	-4387.881669	-4387.099434	22.92
$^{L3}\mathbf{TS}_{A\text{-}B}{}^{S}$	-4385.065013	0.780274	-4387.888293	-4387.108019	17.53
$^{L4}\mathbf{TS}_{A\text{-}B}{}^{R}$	-5383.376966	0.759125	-5386.107161	-5385.348036	/
$^{L4}\mathbf{TS}_{A\text{-}B}{}^{S}$	-5383.385899	0.760556	-5386.113723	-5385.353167	/
$\mathbf{B}^{R\text{-}anti}$	-4385.133326	0.785181	-4387.951765	-4.387.166584	-19.22
$\mathbf{B}^{S\text{-}anti}$	-4385.129352	0.783911	-4387.949639	-4.387.165728	-18.68
$\mathbf{B}^{R\text{-}syn}$	-4385.132168	0.782787	-4387.955543	-4.387.172756	-23.09
$\mathbf{B}^{S\text{-}syn}$	-4385.134933	0.784975	-4387.954666	-4.387.169691	-21.17
$^{L3}\mathbf{TS}_{B\text{-}C}{}^{R\text{-}E}$	-4385.105680	0.784058	-4387.926181	-4387.142123	-3.87
$^{L3}\mathbf{TS}_{B\text{-}C}{}^{S\text{-}E}$	-4385.102330	0.779852	-4387.925567	-4387.145715	-6.12
$^{L3}\mathbf{TS}_{B\text{-}C}{}^{R\text{-}Z}$	-4385.104682	0.783392	-4387.927074	-4387.143682	-4.85
$^{L3}\mathbf{TS}_{B\text{-}C}{}^{S\text{-}Z}$	-4385.103940	0.784089	-4387.929547	-4387.145458	-5.96
$\mathbf{C}^{R\text{-}Z}$	-4385.117910	0.780759	-4387.932879	-4387.152120	-10.14
$\mathbf{C}^{S\text{-}Z}$	-4385.121178	0.784809	-4387.936808	-4.387.151999	-10.07
$\mathbf{C}^{R\text{-}E}$	-4385.110758	0.781462	-4387.931403	-4387.149941	-8.78
$\mathbf{C}^{S\text{-}Z}$	-4385.110192	0.781192	-4387.932701	-4.387.151509	-9.76
$^{L3}\mathbf{TS}_{C\text{-}D}{}^{R\text{-}Z}$	-4385.103603	0.784053	-4387.916502	-4387.132449	2.20
$^{L3}\mathbf{TS}_{C\text{-}D}{}^{S\text{-}Z}$	-4385.103627	0.785532	-4387.919187	-4387.133655	1.44
$^{L3}\mathbf{TS}_{C\text{-}D}{}^{R\text{-}E}$	-4385.109885	0.783182	-4387.926709	-4387.143527	-4.75
$^{L3}\mathbf{TS}_{C\text{-}D}{}^{S\text{-}E}$	-4385.109526	0.784647	-4387.929368	-4387.144721	-5.50
$^{L3}\mathbf{TS}_{B\text{-}D}{}^{R\text{-}anti}$	-4385.089120	0.787604	-4387.905170	-4387.117566	11.54
$^{L3}\mathbf{TS}_{B\text{-}D}{}^{S\text{-}anti}$	Could not be located				
$^{L3}\mathbf{TS}_{B\text{-}D}{}^{R\text{-}syn}$	-4385.090137	0.784237	-4387.913345	-4387.129108	4.30
$^{L3}\mathbf{TS}_{B\text{-}D}{}^{S\text{-}syn}$	-4385.084688	0.783578	-4387.907559	-4387.123981	7.51
$\mathbf{D}^{R\text{-}anti}$	-4385.139902	0.788273	-4387.954823	-4387.166550	-19.20
$\mathbf{D}^{S\text{-}anti}$	-4385.140841	0.784120	-4387.956025	-4387.171905	-22.56
$\mathbf{D}^{R\text{-}syn}$	-4385.123120	0.787349	-4387.939457	-4387.152108	-10.14
$\mathbf{D}^{S\text{-}syn}$	-4385.130663	0.786154	-4387.946302	-4387.160148	-15.18
$\mathbf{E}^{R}$	-4385.176725	0.788453	-4387.989513	-4.387.201060	-40.85
$\mathbf{E}^{S}$	-4385.180600	0.788756	-4387.993259	-4.387.204503	-43.01
**3A**	-746.914862	0.220052	-747.784959	-747.564907	/
[(**L3**)CuOH]	-3714.540693	0.563585	-3716.624491	-3716.060906	/
H_2O	-76.317440	0.003421	-76.447657	-76.444236	/

**3A**+[(**L3**)CuOH]-H_2O	-4.385.138115	0.780216	-4387.961793	-4387.181577	-28.63

Table S12. Electronic energies of the optimized structures involved in the hydrocupration/Dieckmann–Thorpe reaction sequence.

9.2 Energy decomposition analysis

To identify and analyze the factors governing the enantioselectivity of the process, an energy decomposition analysis (EDA) was performed. The two diastereomeric transition states, **TS$_{A\text{-}B}$**^{R} and **TS$_{A\text{-}B}$**^{S}, corresponding to the hydrocupration step, were analyzed using both ligands **L3** and **L4**. Since the energy decomposition analysis requires the artificial fragmentation of optimized molecular structures, the harmonic approximation -valid only for fully optimized geometries- cannot be applied. Therefore, electronic energies (E) were used instead of Gibbs free energies. The corresponding reaction energy difference (ΔE‡), computed at the ωB97XD/def2TZVPP level of theory in the gas phase, can be decomposed into three components, as shown in the following equation:

$$\Delta\Delta E^‡ = \Delta\Delta E^‡_{dist} + \Delta\Delta E^‡_{space} + \Delta\Delta E^‡_{bond}$$

The computed ΔΔE‡ values are:

$$^{L3}\Delta\Delta E^‡ = -4387.836646 + 4387.840666 = 0.004020 \text{ Hartree} = 2.52 \text{ kcal/mol}$$

$$^{L4}\Delta\Delta E^‡ = -5386.069679 + 5386.077307 = 0.007628 \text{ Hartree} = 4.79 \text{ Kcal/mol}$$

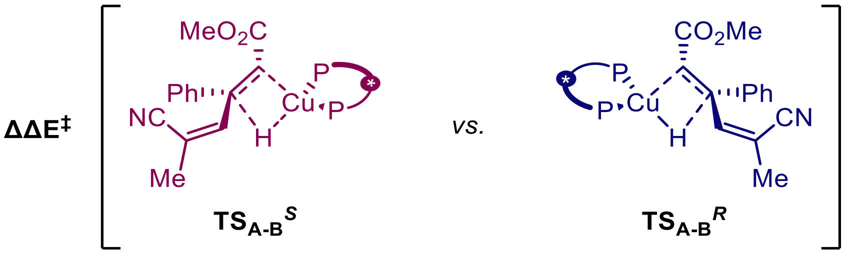

Subsequently, the components contributing to the reaction ΔΔE‡ were computed as follows:

- $\Delta\Delta E^‡_{dist} = \Delta\Delta E^‡_{dist\_sub} + \Delta\Delta E^‡_{dist\_L*CuH}$, accounts for the sum of the relative distortion energies of the separated L*CuH complex and substrate at the TS geometry.

$$^{L3}\Delta\Delta E^‡_{dist\_sub} = -746.472142 + 746.469111 = -0.003031 \text{ Hartree} = -1.90 \text{ kcal/mol}$$

$$^{L3}\Delta\Delta E^‡_{dist\_L*CuH} = -3641.294072 + 3641.288269 = -0.005803 \text{ Hartree} = -3.64 \text{ kcal/mol}$$

$$^{L3}\Delta\Delta E^‡_{dist} = -0.003031 - 0.005803 = -0.008834 \text{ Hartree} = -5.54 \text{ kcal/mol}$$

$$^{L4}\Delta\Delta E^‡_{dist\_sub} = -554.746255 + 554.743773 = -0.002482 \text{ Hartree} = -1.56 \text{ kcal/mol}$$

$$^{L4}\Delta\Delta E^‡_{dist\_L*CuH} = -4831.259357 + 4831.265569 = 0.006212 \text{ Hartree} = 3.90 \text{ kcal/mol}$$

$$^{L4}\Delta\Delta E^‡_{dist} = -0.002482 + 0.006212 = 0.003730 \text{ Hartree} = 2.34 \text{ kcal/mol}$$

$\Delta\Delta E_{dist\_sub}^{\ddagger}$

$\Delta\Delta E_{dist\_L^*CuH}^{\ddagger}$

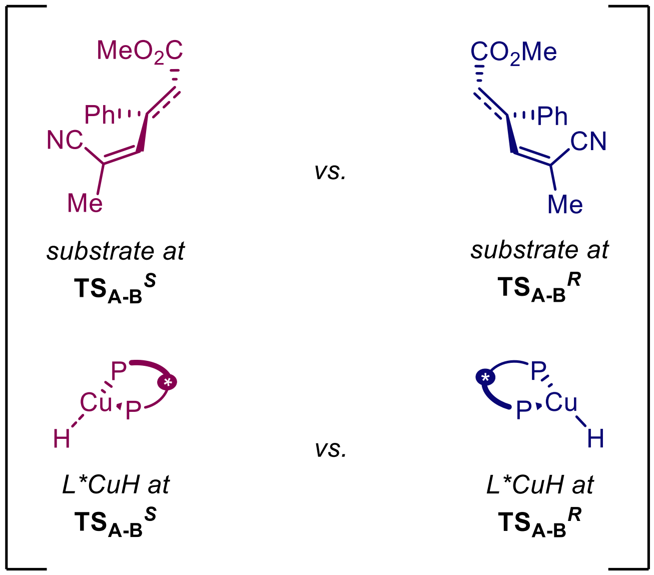

- $\Delta\Delta E^{\ddagger}_{\mathrm{space}}$ accounts for the relative through-space (i.e., noncovalent) interaction energy present between the ligand and the substrate, and is calculated from a supramolecular complex with the TS geometry in which the CuH moiety has been removed.

$$^{L3}\Delta\Delta E^{\ddagger}_{\mathrm{space}} = -2746.590169 + 2746.589828 = -0.000341 \text{ Hartree} = -0.21 \text{ kcal/mol}$$

$$^{L4}\Delta\Delta E^{\ddagger}_{\mathrm{space}} = -3744.820031 + 3744.824909 = 0.004878 \text{ Hartree} = 3.06 \text{ kcal/mol}$$

$\Delta\Delta E_{space}^{\ddagger}$

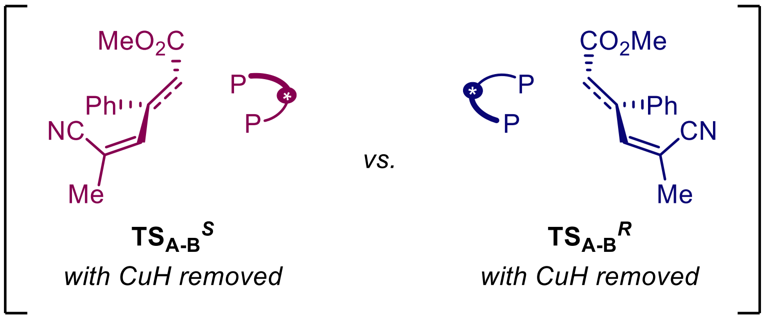

- $\Delta\Delta E^{\ddagger}_{\mathrm{bond}}$ is the relative through-bond interaction energy occurring at the TS and is obtained by subtracting $\Delta\Delta E^{\ddagger}_{\mathrm{dist}}$ and $\Delta\Delta E^{\ddagger}_{\mathrm{space}}$ from $\Delta\Delta E^{\ddagger}$, according to the equation $\Delta\Delta E^{\ddagger} = \Delta\Delta E^{\ddagger}_{\mathrm{dist}} + \Delta\Delta E^{\ddagger}_{\mathrm{space}} + \Delta\Delta E^{\ddagger}_{\mathrm{bond}}$.

$$^{L3}\Delta\Delta E^{\ddagger}_{\mathrm{bond}} = \Delta\Delta E^{\ddagger} - \Delta\Delta E^{\ddagger}_{\mathrm{dist}} - \Delta\Delta E^{\ddagger}_{\mathrm{space}} = 2.52 + 5.54 + 0.21 = 8.27 \text{ kcal/mol.}$$

$$^{L4}\Delta\Delta E^{\ddagger}_{\mathrm{bond}} = \Delta\Delta E^{\ddagger} - \Delta\Delta E^{\ddagger}_{\mathrm{dist}} - \Delta\Delta E^{\ddagger}_{\mathrm{space}} = 4.79 - 2.34 - 3.06 = -0.61 \text{ kcal/mol.}$$

9.3 Isomeric and stereochemical stability of CpHs

In this section, all five isomers and the corresponding transition states (TSs) of CpH **15** accessible via the [1,5]-hydrogen shift were investigated through DFT calculations. In addition, compounds **16**''', **17**''', and **18**''', together with their corresponding isomers **16**'', **17**'', and **18**'', and the associated TSs, were also computed (Table S13). Geometry optimizations and conformational analyses were performed at the ωB97XD/def2SV level of theory in the gas phase and thermal

corrections were obtained from the vibrational analysis on the optimized geometries. Single-point energy (SPE) calculations were then performed using three different functionals (PBE-D3, M06-2X and ωB97XD) with def2TZVVP as the basis set, including solvation effects via the SMD implicit solvation model for tetrahydrofuran (THF). Final energies were reported at the ωB97XD/def2TZVPP level of theory (see Section 8.4).

	ωB97XD/def2SV		1)*PBE-D3/def2TZVPP*<br>2)*M06-2X/def2TZVPP*<br>3)*ωB97XD/def2TZVPP*		
**Isomer**	**Energy (Hartree)**	**Thermal correction to Gibbs Free Energy (Hartree)**	**Electronic energy (SMD = THF, Hartree)**	**Gibbs Free Energy (Hartree)**	**ΔG (kcal/mol)**
**15^{I}**	-594.697779	0.198897	1) -594.827868<br>2) -595.296465<br>3) -595.365570	1) -594.628971<br>2) -595.097568<br>3) -595.166673	3) 1.87
**15^{I}-15^{II}**	-594.642127	0.194562	1) -594.779335<br>2) -595.242622<br>3) -595.309603	1) -594.584773<br>2) -595.048060<br>3) -595.115041	3) 34.27
**15^{II}**	-594.687797	0.196609	1) -594.818690<br>2) -595.286551<br>3) -595.355604	1) -594.622081<br>2) -595.089942<br>3) -595.158995	3) 6.69
**15^{II}-15^{III}**	-594.650550	0.194446	1) -594.787004<br>2) -595.251167<br>3) -595.318470	1) -594.592558<br>2) -595.056721<br>3) -595.124024	3) 28.63
**15^{III}**	-594.699980	0.198645	1) -594.831167<br>2) -595.298895<br>3) -595.368296	1) -594.632522<br>2) -595.100250<br>3) -595.169651	3) 0.00
**15^{III}-15^{IV}**	-594.638544	0.193898	1) -594.775734<br>2) -595.238489<br>3) -595.305724	1) -594.581836<br>2) -595.044591<br>3) -595.111826	3) 36.29
**15^{IV}**	-594.680090	0.197170	1) -594.809927<br>2) -595.279869<br>3) -595.348296	1) -594.612757<br>2) -595.082699<br>3) -595.151126	3) 11.62
**15^{IV}-15^{V}**	-594.631580	0.193413	1) -594.768160<br>2) -595.232029<br>3) -595.299112	1) -594.574747<br>2) -595.038616<br>3) -595.105699	3) 40.13
**15^{V}**	-594.678368	0.197905	1) -594.808483<br>2) -595.278160<br>3) -595.346922	1) -594.610578<br>2) -595.080255<br>3) -595.149017	3) 12.95
**15^{V}-15^{I}**	-594.645662	0.195514	1) -594.780384<br>2) -595.245755<br>3) -595.313045	1) -594.584870<br>2) -595.050241<br>3) -595.117531	3) 32.71
**18^{III}**	-539.380303	0.182226	1) -539.489354<br>2) -539.920532<br>3) -539.984483	1) -539.307128<br>2) -539.738306<br>3) -539.802257	3) 2.62
**18^{III}-18^{II}**	-539.341016	0.177420	1) -539.456108<br>2) -539.883640<br>3) -539.945537	1) -539.278688<br>2) -539.706220<br>3) -539.768117	3) 24.05
**18^{II}**	-539.382994	0.180897	1) -539.492335	1) -539.311438	3) 0.00

			2) -539.923182<br>3) -539.987337	2) -539.742285<br>3) -539.806440	
**17^{III}**	-327.758075	0.134569	1) -327.800044<br>2) -328.076755<br>3) -328.124701	1) -327.665475<br>2) -327.942186<br>3) -327.990132	3) 2.57
**17^{III}-17^{II}**	-327.714243	0.130100	1) -327.762449<br>2) -328.034078<br>3) -328.080502	1) -327.632349<br>2) -327.903978<br>3) -327.950402	3) 27.50
**17^{II}**	-327.760910	0.132823	1) -327.803306<br>2) -328.078776<br>3) -328.127052	1) -327.670483<br>2) -327.945953<br>3) -327.994229	3) 0.00
**16^{III}**	-272.454172	0.118771	1) -272.471656<br>2) -272.710532<br>3) -272.753384	1) -272.352885<br>2) -272.591761<br>3) -272.634613	3) 3.77
**16^{III}-16^{II}**	-272.413449	0.114372	1) -272.437571<br>2) -272.671512<br>3) -272.712537	1) -272.323199<br>2) -272.557140<br>3) -272.598165	3) 26.64
**16^{II}**	-272.459530	0.117815	1) -272.477572<br>2) -272.715353<br>3) -272.758437	1) -272.359757<br>2) -272.597538<br>3) -272.640622	3) 0.00

Table S13. Electronic energies of the optimized structures involved in the [1,5]-hydrogen shift study.

9.4 Study of the rate of the racemization process of CpHs at different temperatures

The rate of the racemization process was studied by monitoring the evolution of the enantiomeric ratio (*er*) of an enantioenriched stock 10 mg/mL solution of CpH **3a** (*er*=97:3) in dry toluene over time at 40, 60, 70, 80, 90, and 100 °C, using chiral HPLC (Phenomenex cellulose-1, Hexane:2-propanol 90:10, flow: 1.0 mL/min, 315 nm) (Fig. S3). Experimental data are summarized in Table S14.

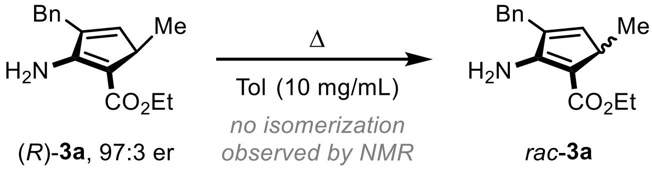

Figure S3. Racemization of CpH (*R*)-**3a**.

t (min)	[3a^R]%	[3a^R] (mg/mL)	ln([3a^R])	ln(2[3a^R]-([3a^R]+[3a^S])
**T = 40 °C**				
0	96.9	9.69	2.271094	2.23858
60	96.9	9.69	2.271094	2.23858
120	96.9	9.69	2.271094	2.23858
180	96.9	9.69	2.271094	2.23858
240	96.9	9.69	2.271094	2.23858
300	96.9	9.69	2.271094	2.23858
360	96.9	9.69	2.271094	2.23858
**T = 60 °C**				
0	96.9	9.69	2.271094	2.238580
60	96.7	9.67	2.269028	2.234306
120	96.5	9.65	2.266958	2.230014

180	96.1	9.61	2.262804	2.221375
240	95.5	9.55	2.256541	2.208274
300	95.0	9.50	2.251292	2.197225
360	94.7	9.47	2.248129	2.190536
**T = 70 °C**				
0	96.9	9.69	2.271094	2.238580
60	96.0	9.60	2.261763	2.219203
120	94.8	9.48	2.249184	2.192770
180	92.7	9.27	2.226783	2.144761
240	91.1	9.11	2.209373	2.106570
300	89.3	8.93	2.189416	2.061787
360	88.0	8.80	2.174752	2.028148
**T = 80 °C**				
0	96.9	9.69	2.271094	2.238580
60	93.5	9.35	2.235376	2.163323
120	89.2	8.92	2.188296	2.059239
180	84.8	8.48	2.137710	1.940179
240	80.9	8.09	2.090629	1.821318
300	77.5	7.75	2.047693	1.704748
360	74.5	7.45	2.008214	1.589235
**T = 90 °C**				
0	96.9	9.69	2.271094	2.238580
20	94.9	9.49	2.250239	2.195000
40	90.6	9.06	2.203869	2.094330
60	85.8	8.58	2.149434	1.968510
80	82.1	8.21	2.105353	1.859418
100	78.6	7.86	2.061787	1.743969
120	74.9	7.49	2.013569	1.605430
150	70.9	7.09	1.958685	1.430311
180	68.0	6.80	1.916923	1.280934
240	62.8	6.28	1.837370	0.940007
300	59.0	5.90	1.774952	0.587787
360	56.5	5.65	1.731656	0.262364
**T = 100 °C**				
0	96.9	9.69	2.271094	2.238580
10	94.6	9.46	2.247072	2.188296
20	89.4	8.94	2.190536	2.064328
30	84.2	8.42	2.130610	1.922788
40	78.4	7.84	2.059239	1.736951
50	75.2	7.52	2.017566	1.617406
60	71.8	7.18	1.971299	1.472472
80	66.2	6.62	1.890095	1.175573
100	61.8	6.18	1.821318	0.858662
120	58.9	5.89	1.773256	0.576613
150	56.0	5.60	1.722767	0.182322
180	53.5	5.35	1.677097	-0.356670

Table S14. Experimental data of the thermally induced racemization study.

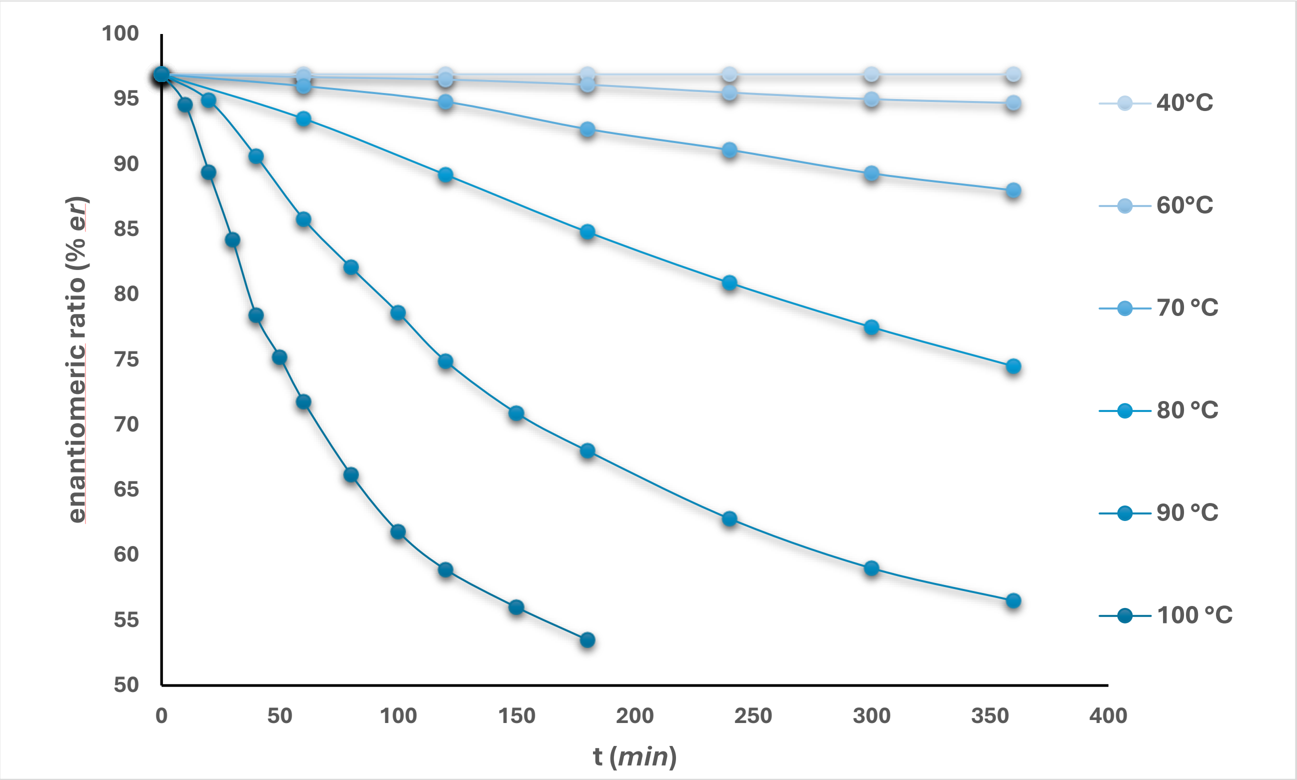

Graph S1. Kinetic profile of the enantiomeric ratio at different temperatures of compound **3a**.

The racemization process can be described by a first-order equilibrium kinetics. The approach used to determine the correct kinetic law is outlined below.

$$\mathrm{R} \rightleftharpoons \mathrm{S}$$

Boundary conditions**:**

- $k_1 = k_{-1}$
- [R] + [S] = C

$$-\frac{\mathrm{d[R]}}{\mathrm{dt}} = k_1[\mathrm{R}] - k_{-1}[\mathrm{S}] = k_1[\mathrm{R}] - k_1(\mathrm{C} - [\mathrm{R}]) = k_1(2[\mathrm{R}] - \mathrm{C})$$

$$\int_{\mathrm{C}}^{[\mathrm{R}]} \frac{\mathrm{d[R]}}{k_1(2[\mathrm{R}] - \mathrm{C})} = \int_0^{\mathrm{t}} -\mathrm{dt}$$

$$\frac{1}{2k_1}\ln|2[\mathrm{R}] - \mathrm{C}| - \frac{1}{2k_1}\ln\mathrm{C} = -\mathrm{t}$$

$$\ln|2[\mathrm{R}] - \mathrm{C}| - \ln\mathrm{C} = -2k_1\mathrm{t}$$

$$\mathbf{\ln|2[R] - C| = -2\boldsymbol{k_1}t + \ln C}$$

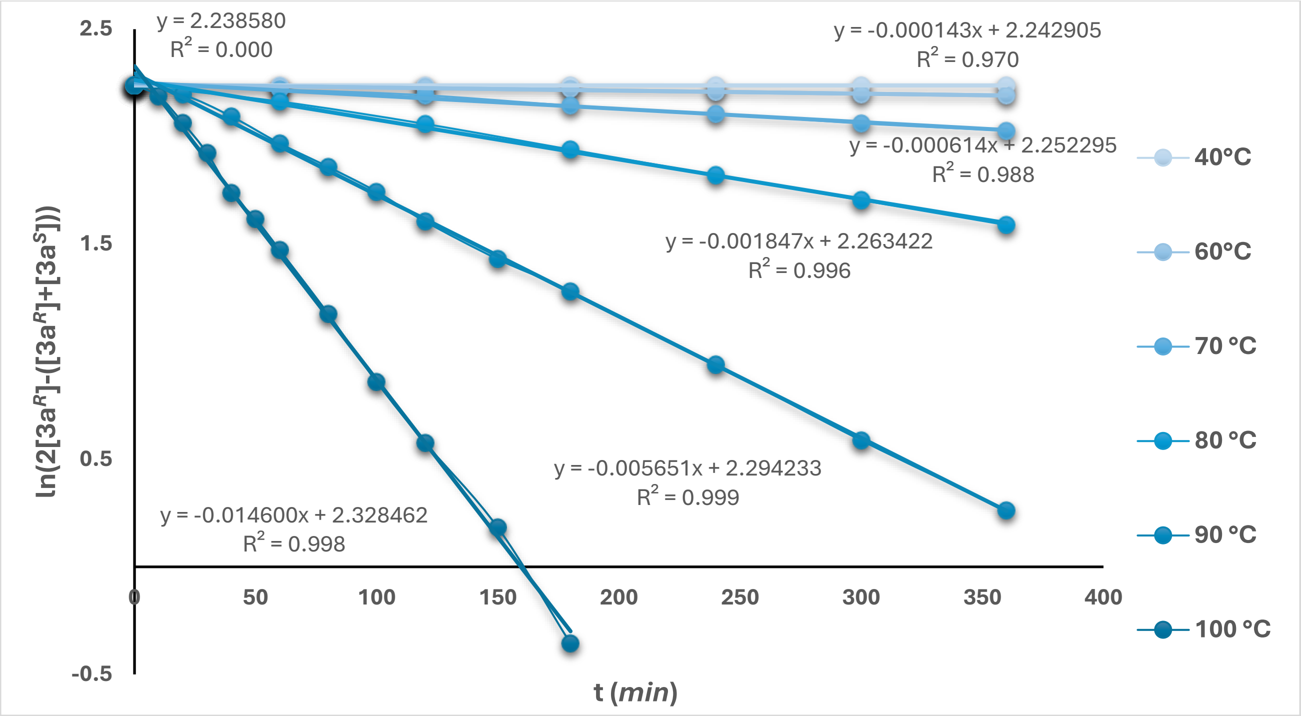

Graph S2. Linearized kinetic plot for the racemization of CpH **3a**.

The rate constants at each temperature were then employed in the linearized form of the Eyring–Evans–Polanyi equation to evaluate the activation parameters of the racemization process.

$$\ln\frac{k}{T} = -\frac{\Delta H^{\ddagger}}{R}\cdot\frac{1}{T} + \ln\frac{k_B}{h} + \frac{\Delta S^{\ddagger}}{R}$$

T (°C)	*k* (s^{-1})	ln (*k*/T)
40	/	/
60	$7.2\cdot10^{-5}$	-15.35
70	$30.7\cdot10^{-5}$	-13.93
80	$92.4\cdot10^{-5}$	-12.85
90	$282.6\cdot10^{-5}$	-11.76
100	$730.0\cdot10^{-5}$	-10.84

Table S15. Rate constants (*k*) at different temperatures (T).

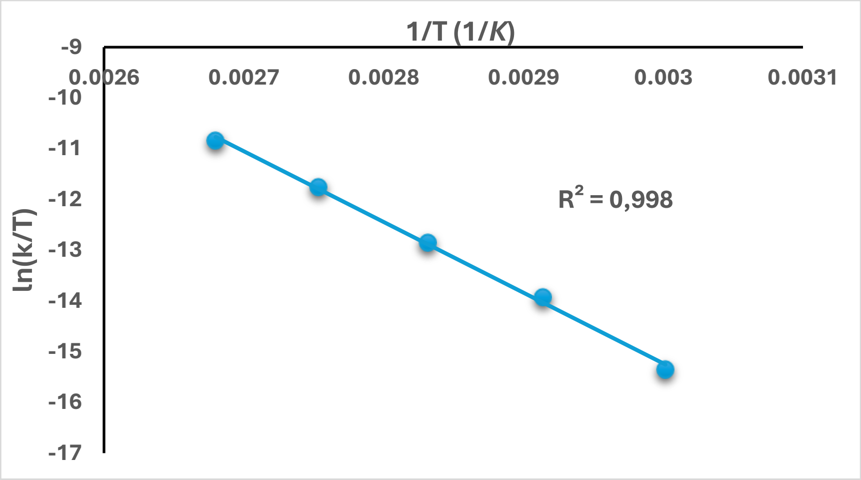

Graph S3. Eyring–Evans–Polanyi plot.

$$\Delta H^{\ddagger}_{exp} = 27.7 \pm 0.1 \text{ kcal/mol}$$

$$\Delta S^{\ddagger}_{exp} = 5.6 \pm 0.3 \text{ cal/mol}$$

The standard errors of the experimental activation enthalpy and entropy were determined from the regression analysis of the experimental data, at the 95% confidence level.

Experimental results were compared with those obtained from DFT calculations. The activation barrier of the process leading to the racemization of CpH was determined, corresponding to the reaction that converts the chiral compound **3a**^{III} into the achiral isomer **3a**^{II} (Fig. S4). Previous calculations (see Section 8.3) showed that, for CpHs, this step involves the transition state (TS) with the lowest energy among all those associated with the overall isomerization process.
Geometry optimizations and conformational analyses were performed at the ωB97XD/def2SV level of theory in the gas phase (Table S16). Thermal corrections were obtained from the vibrational analysis on the optimized geometries. Single-point energy (SPE) calculations were then performed using different functionals (PBE-D3, M06-2X, ωB97XD and B3LYP-D3). In all cases, def2TZVPP was used as basis set, and solvation effects were considered using the SMD implicit solvation model for tetrahydrofuran (THF).

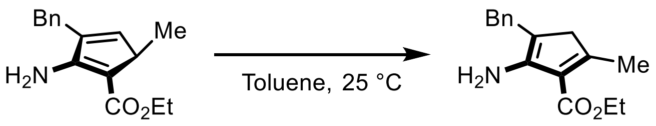

3a^{III} **3a**^{II}

Figure S4. Isomerization of CpH **3a**^{III}.

	ωB97XD/def2SV				*1)PBE-D3/def2TZVPP*<br>*2)M06-2X/def2TZVPP*<br>*3)ωB97XD/def2TZVPP*<br>*4)B3LYP-D3/def2TZVPP*	
**Conformer**	**Energy (Hartree)**	**Thermal correction to Gibbs Free Energy (Hartree)**	**Thermal correction to Enthalpy (Hartree)**	**Entropy (cal·mol^{-1}·K^{-1})**	**Electronic energy (SMD = Toluene, Hartree)**	**ΔH‡ (kcal/mol)**
**3a**^{III}	-825.512849	0.274340	0.341614	141.589	1) -825.675311<br>2) -826.341991<br>3) -826.424469<br>4) -826.736968	1) 24.704<br>2) 26.200<br>3) 28.327<br>4) 29.200
**3a**^{III}**-3a**^{II}	-825.463697	0.269499	0.337228	142.547	1) -825.631556<br>2) -826.294474<br>3) -826.374940<br>4) -826.686049	

Table S16. Electronic energies of the optimized structures **3a**^{III} and **3a**^{III}**-3a**^{II}.

$$\Delta H^{\ddagger}_{calc} = 28.3 \text{ kcal/mol}$$

$$\Delta S^{\ddagger}_{calc} = 1.0 \text{ cal/mol}$$

Among the tested functionals, ωB97XD provided the best agreement with the experimental data ($\Delta H^{\ddagger}_{calc}$ = 28.3 kcal/mol *vs* $\Delta H^{\ddagger}_{exp}$ = 27.7 kcal/mol).

9.5 Computed acidity of CpHs

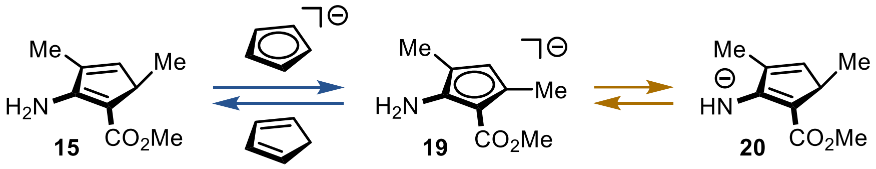

	ωB97XD/def2SVP		*ωB97XD/def2TZVPP*	
**Compound**	**Energy (Hartree)**	**Thermal correction to Gibbs Free Energy (Hartree)**	**Electronic energy (SMD = DMSO, Hartree)**	**Gibbs Free Energy (Hartree)**
**15**	-594.69998	0.198645	-595.366609	-595.167964
**Cyclopentadiene**	-193.897345	0.066553	-194.111667	-194.045114
**Cyclopentadienyl anion**	-193.309945	0.053162	-193.622536	-193.569374
**19**	-594.119005	0.184464	-594.86045	-594.675986
**20**	-594.108582	0.184847	-594.852875	-594.668028

Using cyclopentadiene as a reference, the pK_{AH} was obtained through the following equations[53]:

$$\Delta G^*_{\mathrm{DMSO,\ exchange}} = \Delta G^*_{\mathrm{gas,\ exchange}} + \Delta G^*_{\mathrm{DMSO}}(\mathbf{19}) + \Delta G^*_{\mathrm{DMSO}}(\mathbf{Cyclopentadiene}) - \Delta G^*_{\mathrm{DMSO}}(\mathbf{15}) - \Delta G^*_{\mathrm{DMSO}}(\mathbf{Cyclopentadienyl\ anion}) = 10.2\ \mathrm{kcal/mol}$$

$$\mathrm{pK_{AH}}(\mathbf{15}) = \frac{\Delta G^*_{\mathrm{DMSO,\ exchange}}}{\mathrm{RTln}(10)} + \mathrm{pK_{AH}}(\mathbf{Cyclopentadiene}) = 25.5$$

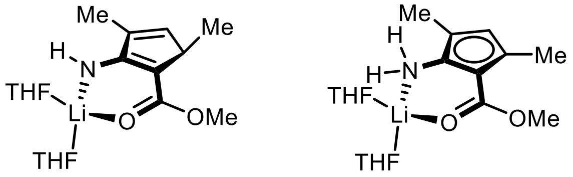

21, 0.0 kcal/mol **22**, 1.4 kcal/mol

	ωB97XD/def2SVP		*ωB97XD/def2TZVPP*	
**Compound**	**Energy (Hartree)**	**Thermal correction to Gibbs Free Energy (Hartree)**	**Electronic energy (SMD = THF, Hartree)**	**Gibbs Free Energy (Hartree)**
**21**	-1066.194827	0.408081	-1067.356095	-1066.948014
**22**	-1066.195103	0.407944	-1067.353752	-1066.945808

9.6 Cartesian geometries

[(**L3**)CuH]

C	-2.87128	-1.70772	0.4049
C	-0.40285	-2.85731	0.14414
C	-1.46735	-3.70321	0.8491
C	-2.80834	-3.24612	0.27097
H	-3.07399	-1.47312	1.46447
H	-0.37973	-3.16609	-0.9159
H	-1.29172	-4.78238	0.69735
H	-1.46084	-3.52024	1.93883
H	-2.87021	-3.52438	-0.79664
H	-3.66936	-3.71483	0.77711
C	-0.68078	-0.35418	1.71476
C	0.68083	0.354	1.71482
H	-1.48718	0.37652	1.90647
H	-0.72242	-1.10524	2.52386
H	0.72245	1.10495	2.52403
H	1.48725	-0.3767	1.90646
C	0.40298	2.85729	0.14441
C	2.87136	1.70766	0.40506
C	1.46743	3.70311	0.84954
H	0.37999	3.16616	-0.91561
C	2.80842	3.2461	0.27132
H	3.07418	1.47297	1.46459
H	1.46092	3.51998	1.93924
H	1.29181	4.7823	0.69794
H	3.66947	3.71473	0.7775
H	2.87025	3.5245	-0.79625
P	1.11895	1.12065	0.07726
P	-1.11886	-1.12066	0.07707
Cu	-0.00015	0.00019	-1.60948
H	-0.00005	0.00012	-3.18416
C	-1.01609	2.84458	0.65198
C	-3.66432	2.56038	1.5612
C	-2.03119	2.39885	-0.20941
C	-1.35625	3.16735	1.96947
C	-2.67024	3.02289	2.42168
C	-3.34151	2.25707	0.23687
H	-1.78515	2.14127	-1.24648
H	-0.59252	3.52992	2.66283
H	-2.9153	3.27617	3.45749
H	-4.11321	1.90217	-0.45179
H	-4.69245	2.44261	1.91526
C	3.9437	1.05081	-0.43335
C	5.98499	-0.15448	-1.94695
C	3.73922	0.73377	-1.78227
C	5.18785	0.76339	0.14013
C	6.2025	0.16598	-0.60686
C	4.75057	0.13337	-2.53088
H	2.7638	0.9144	-2.24672
H	5.3607	0.9997	1.19561
H	7.16548	-0.05617	-0.13749
H	4.56458	-0.1198	-3.57853
H	6.77518	-0.63045	-2.5348
C	-3.94366	-1.05075	-0.43336
C	-5.98515	0.15468	-1.94661
C	-5.18771	-0.76325	0.14032
C	-3.73939	-0.7337	-1.7823
C	-4.75083	-0.13324	-2.53075
C	-6.20245	-0.16577	-0.60648
H	-5.36041	-0.99958	1.19583
H	-2.76407	-0.91438	-2.24692
H	-4.565	0.11992	-3.57842
H	-7.16533	0.05645	-0.13696
H	-6.77541	0.6307	-2.53432
C	1.01616	-2.84466	0.65187
C	3.66436	-2.56066	1.5613
C	2.03134	-2.39884	-0.2094
C	1.35624	-3.16765	1.96931
C	2.6702	-3.02327	2.42164
C	3.34164	-2.25716	0.23698
H	1.78538	-2.1411	-1.24644
H	0.59247	-3.53032	2.66259
H	2.91518	-3.27673	3.45742
H	4.11339	-1.90216	-0.45157
H	4.69247	-2.44298	1.91544

1A

C	-1.18199	3.17902	0.00002
C	-0.95115	2.52017	-1.20682
C	-0.50038	1.20308	-1.2078
C	-0.28074	0.53649	0.00004
C	-0.50068	1.20299	1.20787
C	-0.95144	2.52008	1.20688
H	-1.54454	4.2108	0.00002
H	-1.12799	3.03458	-2.15548
H	-0.32442	0.68225	-2.1536
H	-0.32494	0.68209	2.15367
H	-1.12852	3.03443	2.15552
C	0.13265	-0.89567	0.00002
C	1.42163	-1.30009	0.00002
H	1.6557	-2.36881	-0.00001
C	-0.89951	-1.93964	0.00002
H	-0.51195	-2.96473	0.00018

C	-2.24571	-1.82501	-0.00002
C	2.60173	-0.401	0.00006
O	2.59966	0.80203	0.0002
O	3.72777	-1.13333	-0.0001
C	4.94308	-0.40692	-0.00007
H	5.01694	0.23259	-0.89506
H	5.01698	0.23246	0.895
H	5.74611	-1.15672	-0.00015
C	-3.14991	-3.03484	0.00005
H	-3.80509	-3.0298	-0.88839
H	-2.56281	-3.96645	0.00011
H	-3.80511	-3.02967	0.88848
C	-2.96691	-0.57657	-0.00016
N	-3.6777	0.33783	-0.00026

$^{L3}TS_{A-B}^{R}$

C	2.47682	-1.68239	2.02873
C	0.0663	-0.91596	3.07074
C	1.12742	-1.42162	4.06128
C	2.49907	-1.11242	3.45762
H	2.37264	-2.77794	2.12301
H	-0.01963	0.17602	3.19334
H	0.98966	-0.95673	5.05228
H	1.04661	-2.51277	4.20474
H	2.67516	-0.0208	3.42726
H	3.32309	-1.55688	4.041
C	0.18905	-2.68319	0.63436
C	-1.1735	-2.55785	-0.04496
H	0.95877	-2.98676	-0.09852
H	0.15244	-3.46428	1.41349
H	-1.36072	-3.43625	-0.68685
H	-1.97409	-2.53596	0.71513
C	-0.86368	-1.39595	-2.77437
C	-3.23558	-1.17282	-1.42233
C	-2.11729	-1.9186	-3.48843
H	-0.6203	-0.40788	-3.20122
C	-3.2983	-1.09745	-2.96216
H	-3.47376	-2.2165	-1.14802
H	-2.28937	-2.98509	-3.25684
H	-2.00991	-1.83404	-4.58299
H	-4.26592	-1.48194	-3.32827
H	-3.20678	-0.04987	-3.29503
P	-1.41164	-0.99736	-1.02056
P	0.83033	-1.09834	1.34596
Cu	-0.05805	0.60554	0.00987
H	-0.7594	1.62896	1.04025
C	0.39333	-2.23121	-2.74331
C	2.75695	-3.70798	-2.33141
C	1.61376	-1.58674	-2.48986
C	0.38719	-3.6282	-2.82582
C	1.55974	-4.3601	-2.62336
C	2.78118	-2.31342	-2.27411
H	1.64404	-0.49544	-2.45028
H	-0.5407	-4.16584	-3.03957
H	1.53159	-5.45195	-2.689
H	3.71337	-1.78786	-2.04563
H	3.67152	-4.28151	-2.15512
C	-4.21543	-0.29205	-0.67925
C	-6.11544	1.25517	0.71278
C	-3.85483	0.91778	-0.08164
C	-5.54779	-0.71373	-0.5681
C	-6.49078	0.04964	0.11681
C	-4.79313	1.6836	0.61054
H	-2.81573	1.24962	-0.1173
H	-5.84916	-1.66555	-1.01886
H	-7.52383	-0.30225	0.19273
H	-4.4773	2.6179	1.08448
H	-6.85052	1.8526	1.25971
C	-1.32821	-1.48931	3.14592
C	-3.9355	-2.55154	3.01408
C	-2.41609	-0.69051	2.76279
C	-1.57777	-2.82492	3.48652
C	-2.86804	-3.35188	3.41992
C	-3.7054	-1.21383	2.69174
H	-2.2398	0.35207	2.48152
H	-0.75813	-3.47844	3.79772
H	-3.0374	-4.39967	3.68537
H	-4.53209	-0.57289	2.37161
H	-4.94575	-2.96628	2.95428
O	3.11258	2.31798	-0.78532
C	2.20838	2.0076	-1.5296
C	0.76804	2.04923	-1.30144
H	0.19474	1.93194	-2.22029
C	-1.14868	3.4012	-0.32278
H	-1.39996	3.99846	0.56112
C	-2.04472	3.459	-1.33058
C	-1.94676	2.678	-2.53198
N	-1.97063	2.05212	-3.50767
C	-3.29423	4.30687	-1.25706
H	-4.20117	3.68549	-1.35764
H	-3.34171	4.8424	-0.29548
H	-3.30853	5.05332	-2.07058
C	0.15389	2.68881	-0.17844
C	1.04666	3.38007	0.83058
C	1.4391	4.69293	0.54794
C	1.5014	2.78539	2.00717
C	2.27226	5.39051	1.41908

H	1.09926	5.16776	-0.37828
C	2.34046	3.47646	2.8789
H	1.19903	1.76066	2.22987
C	2.72984	4.7827	2.58814
H	2.57255	6.41455	1.17858
H	2.69243	2.98806	3.7926
H	3.38872	5.32696	3.27086
O	2.45233	1.56711	-2.79034
C	3.80503	1.44041	-3.16884
H	4.31301	0.6665	-2.56864
H	3.79779	1.14632	-4.2283
H	4.34361	2.39458	-3.04336
C	3.69269	-1.40518	1.18471
C	4.01349	-0.1085	0.77197
C	4.54219	-2.45646	0.81772
C	5.1631	0.13363	0.02185
H	3.35772	0.73436	1.0098
C	5.69082	-2.21932	0.06402
H	4.29767	-3.47916	1.1239
C	6.00644	-0.91977	-0.33367
H	5.37469	1.1576	-0.29378
H	6.34142	-3.0541	-0.21363
H	6.90772	-0.72897	-0.92401

$^{L3}TS_{A-B}^{S}$

C	3.29682	-0.61124	1.74031
C	0.94892	-0.6371	3.15012
C	2.20676	-1.02941	3.93062
C	3.36354	-0.30136	3.24694
H	3.71438	-1.62168	1.5761
H	0.77263	0.44248	3.30388
H	2.11908	-0.7589	4.99687
H	2.37944	-2.1195	3.88343
H	3.23493	0.78345	3.40792
H	4.3435	-0.58013	3.67023
C	1.25754	-2.54487	0.91136
C	-0.15845	-2.88506	0.42752
H	1.98708	-2.73834	0.1029
H	1.53225	-3.19579	1.76043
H	-0.18291	-3.90333	0.0038
H	-0.86208	-2.87373	1.27825
C	-0.41093	-2.28455	-2.49475
C	-2.6392	-2.22364	-0.89798
C	-1.57996	-3.18639	-2.91255
H	-0.44636	-1.37903	-3.12318
C	-2.84958	-2.51016	-2.39869
H	-2.67543	-3.19206	-0.36851
H	-1.49386	-4.18783	-2.45442
H	-1.59367	-3.32959	-4.00659
H	-3.75103	-3.12742	-2.55126
H	-3.01397	-1.55753	-2.93392
P	-0.85458	-1.65646	-0.77476
P	1.45486	-0.74765	1.34495
Cu	0.01109	0.34931	-0.13053
H	-0.80508	1.29674	0.80258
C	0.99012	-2.83327	-2.58457
C	3.6568	-3.73917	-2.61513
C	2.03804	-1.96721	-2.93016
C	1.29953	-4.16668	-2.28542
C	2.62014	-4.61516	-2.29485
C	3.3576	-2.41729	-2.94475
H	1.80443	-0.93213	-3.20547
H	0.50477	-4.87623	-2.03904
H	2.8386	-5.65971	-2.05378
H	4.16138	-1.72689	-3.21718
H	4.69287	-4.08959	-2.62343
C	-3.68609	-1.31908	-0.29573
C	-5.73874	0.2961	0.74351
C	-3.67472	0.06619	-0.50195
C	-4.73963	-1.87947	0.43547
C	-5.7597	-1.08219	0.95303
C	-4.69472	0.86474	0.013
H	-2.85215	0.53529	-1.05228
H	-4.75769	-2.96073	0.60912
H	-6.57183	-1.54033	1.52539
H	-4.66731	1.94388	-0.16008
H	-6.5347	0.92745	1.14887
C	4.02262	0.34719	0.81855
C	5.29707	2.11432	-0.97221
C	3.8672	0.21077	-0.56783
C	4.85066	1.37086	1.28858
C	5.48024	2.24851	0.40192
C	4.49145	1.0811	-1.45563
H	3.22401	-0.58099	-0.96217
H	5.01426	1.50107	2.36099
H	6.11875	3.0454	0.79485
H	4.33541	0.95987	-2.53189
H	5.78375	2.80624	-1.66526
C	-0.34516	-1.35789	3.42867
C	-2.79868	-2.71056	3.67942
C	-1.55381	-0.71794	3.11602
C	-0.38992	-2.67284	3.90681
C	-1.60752	-3.34513	4.0295
C	-2.76768	-1.38842	3.23218
H	-1.53783	0.31526	2.75967
H	0.53168	-3.19397	4.18013
H	-1.62163	-4.37425	4.40086

H	-3.69405	-0.87311	2.96488
H	-3.75228	-3.23959	3.76739
O	0.47425	0.83821	-3.87331
C	-0.02534	1.26801	-2.84918
C	0.71355	1.60506	-1.64512
O	-1.36472	1.42483	-2.73932
H	1.79258	1.48143	-1.75111
C	-2.13292	1.20662	-3.90091
H	-3.18143	1.3446	-3.59971
H	-1.8685	1.93008	-4.69033
H	-1.98277	0.18863	-4.29855
C	1.30896	2.8145	0.3877
H	2.3012	2.85699	-0.0799
C	1.24867	3.21021	1.67309
C	0.04334	3.11339	2.4514
N	-0.86424	3.0307	3.16738
C	2.44286	3.7482	2.42282
H	2.65874	3.1397	3.3191
H	3.33357	3.74813	1.77618
H	2.25433	4.78044	2.7667
C	0.2082	2.33966	-0.51498
C	-0.91737	3.33845	-0.70227
C	-2.11756	3.34157	0.00747
C	-0.68642	4.36673	-1.62764
C	-3.06202	4.34815	-0.19535
H	-2.31129	2.53867	0.72213
C	-1.62968	5.3689	-1.8377
H	0.24851	4.37148	-2.19703
C	-2.8257	5.36421	-1.11882
H	-3.99271	4.33496	0.38013
H	-1.42916	6.15824	-2.56832
H	-3.56949	6.15036	-1.27852

$^{L4}TS_{A\text{-}B}^{R}$

C	1.78337	0.10031	-0.76711
C	2.85337	0.57152	-1.59549
C	3.6489	-0.5395	-1.98137
C	3.09033	-1.70457	-1.3844
C	1.93846	-1.32709	-0.62671
H	3.0142	1.60917	-1.88575
H	4.54838	-0.50298	-2.5952
H	3.50007	-2.71019	-1.46617
C	5.27138	-1.16907	0.99094
C	5.53184	0.17804	0.60508
C	4.16617	-1.15945	1.89199
H	5.80866	-2.05119	0.64261
C	4.58406	1.01527	1.26175
H	6.30244	0.50658	-0.09214
C	3.73971	0.18662	2.05721
H	3.71809	-2.02881	2.37188
H	4.51396	2.09725	1.16547
Fe	3.62428	-0.36171	0.06431
H	2.90707	0.5198	2.67506
C	1.06916	-2.27959	0.16485
H	0.85488	-1.78232	1.12661
P	-0.67566	-2.39604	-0.57276
P	0.31594	1.06216	-0.24077
C	0.77265	1.77017	1.38278
C	1.77147	2.7403	1.54057
C	0.11621	1.27796	2.51425
C	2.09461	3.22135	2.80514
H	2.31094	3.11645	0.66689
C	0.4507	1.75008	3.7848
H	-0.67558	0.5305	2.41318
C	1.43422	2.72467	3.93196
H	2.87273	3.98203	2.91662
H	-0.07978	1.36194	4.65847
H	1.68802	3.10425	4.92589
C	0.34513	2.47648	-1.41432
C	0.26904	3.81606	-1.01795
C	0.33958	2.18018	-2.78602
C	0.2431	4.83623	-1.97165
H	0.21127	4.07885	0.0404
C	0.30661	3.19718	-3.73528
H	0.3675	1.138	-3.11675
C	0.26878	4.53301	-3.33062
H	0.19305	5.87754	-1.64022
H	0.31026	2.94401	-4.79936
H	0.24836	5.33399	-4.07526
C	1.82176	-3.57913	0.47852
H	2.84092	-3.33637	0.81849
H	1.91199	-4.24398	-0.3918
H	1.3359	-4.14627	1.2841
C	-0.50139	-2.95637	-2.38465
C	-1.56114	-3.71619	0.4704
C	-0.05856	-1.73728	-3.21705
H	0.93876	-1.36845	-2.93268
H	-0.78192	-0.91144	-3.11786
H	-0.01227	-2.0341	-4.28165
C	0.49763	-4.09483	-2.63044
H	0.44145	-4.39947	-3.69228

H	0.29536	-4.988	-2.01992
H	1.53263	-3.76701	-2.44481
C	-1.8906	-3.36579	-2.8993
H	-2.64651	-2.5936	-2.6716
H	-2.22727	-4.32999	-2.48631
H	-1.84653	-3.47832	-3.99808
C	-1.15348	-5.17658	0.22722
H	-1.47437	-5.52438	-0.76839
H	-1.66215	-5.81773	0.97107
H	-0.07347	-5.35818	0.31944
C	-3.07638	-3.59855	0.22939
H	-3.5942	-4.33787	0.86775
H	-3.36618	-3.80486	-0.81034
H	-3.4611	-2.61061	0.51696
C	-1.3195	-3.32511	1.94188
H	-2.00949	-3.90045	2.58495
H	-1.52525	-2.25307	2.11072
H	-0.29547	-3.5395	2.28601
Cu	-1.59866	-0.28582	-0.42765
H	-2.39205	0.08947	-1.77406
C	-3.02533	0.54411	0.92437
H	-2.47067	1.38083	1.34728
C	-3.54862	-0.35476	1.93714
O	-4.44127	-1.17476	1.8541
O	-2.88415	-0.17831	3.11529
C	-3.33986	-0.94526	4.20633
H	-4.39679	-0.72655	4.43341
H	-3.24535	-2.02572	4.00467
H	-2.70739	-0.6654	5.06162
C	-3.51156	0.65221	-0.41474
C	-4.6701	-0.21925	-0.87929
H	-5.59006	0.09368	-0.35493
H	-4.81871	-0.11315	-1.96615
H	-4.51131	-1.27832	-0.64975
C	-3.49696	1.95929	-1.12446
H	-3.86319	1.88264	-2.15455
C	-3.17521	3.21047	-0.737
C	-2.64619	3.55427	0.55456
N	-2.18774	3.93681	1.54868
C	-3.33082	4.39395	-1.66542
H	-3.75116	4.07354	-2.63179
H	-4.00294	5.15158	-1.22546
H	-2.35766	4.87648	-1.85637

$^{L4}TS_{A\text{-}B}{}^{S}$

C	-1.51218	-0.58235	1.00534
C	-2.30767	-0.70666	2.19116
C	-2.64597	-2.07505	2.36176
C	-2.07537	-2.80511	1.28045
C	-1.36899	-1.89923	0.43162
H	-2.58977	0.11445	2.84974
H	-3.26226	-2.48976	3.15887
H	-2.19315	-3.87486	1.11423
C	-4.98388	-2.39365	-0.18003
C	-5.40825	-1.39182	0.74123
C	-4.28758	-1.74468	-1.24207
H	-5.14566	-3.46685	-0.07927
C	-4.96829	-0.12764	0.25057
H	-5.94897	-1.56578	1.6714
C	-4.27743	-0.34804	-0.97623
H	-3.83618	-2.22796	-2.10799
H	-5.12282	0.83638	0.733
Fe	-3.36286	-1.37701	0.56789
H	-3.81393	0.4151	-1.59929
C	-0.63373	-2.27894	-0.83676
H	-0.88616	-1.50845	-1.58551
P	1.23793	-1.99835	-0.67808
P	-0.59311	0.88944	0.45088
C	-1.75597	1.87539	-0.5596
C	-2.87944	2.52996	-0.03811
C	-1.50132	1.94116	-1.93226
C	-3.73175	3.23984	-0.87977
H	-3.09196	2.48037	1.03422
C	-2.35796	2.6475	-2.77884
H	-0.61402	1.44314	-2.33726
C	-3.47305	3.29771	-2.25259
H	-4.60792	3.74768	-0.46615
H	-2.13666	2.69727	-3.84851
H	-4.1449	3.85537	-2.91152
C	-0.36464	1.86027	1.98689
C	-0.48579	3.25533	2.006
C	0.15164	1.21557	3.12069
C	-0.1217	3.98534	3.13844
H	-0.86046	3.78604	1.12661
C	0.51177	1.94403	4.25087
H	0.29052	0.13103	3.1152

C	0.37509	3.33292	4.26495
H	-0.22605	5.0743	3.13513
H	0.916	1.42206	5.12252
H	0.66381	3.90574	5.15069
C	-1.13153	-3.62287	-1.38374
H	-2.23344	-3.63638	-1.36889
H	-0.78144	-4.48323	-0.79729
H	-0.81635	-3.77847	-2.42444
C	1.86897	-3.06625	0.76113
C	1.97079	-2.56147	-2.33922
C	1.55719	-2.28303	2.0515
H	0.47472	-2.13421	2.19751
H	2.05097	-1.29708	2.05541
H	1.93381	-2.85648	2.91946
C	1.24068	-4.46137	0.87009
H	1.73611	-5.015	1.68964
H	1.36204	-5.0558	-0.04946
H	0.1693	-4.40692	1.11911
C	3.39643	-3.20967	0.66273
H	3.90607	-2.24297	0.51786
H	3.70257	-3.88841	-0.1489
H	3.76848	-3.64897	1.60733
C	2.07987	-4.07893	-2.54681
H	2.79345	-4.54184	-1.8472
H	2.45507	-4.27556	-3.56832
H	1.11684	-4.60116	-2.44416
C	3.3707	-1.9298	-2.46254
H	3.79821	-2.19702	-3.44666
H	4.07412	-2.26635	-1.688
H	3.30832	-0.83214	-2.40202
C	1.11177	-1.93814	-3.45686
H	1.6286	-2.08086	-4.42305
H	0.98925	-0.85161	-3.3042
H	0.11584	-2.39931	-3.5508
Cu	1.45471	0.27237	-0.47896
H	1.66502	0.95407	-1.89396
C	2.92072	1.33962	0.6074
H	2.39307	1.79775	1.44809
C	3.99498	0.45222	1.00395
O	4.81024	-0.12992	0.31122
O	4.00141	0.30267	2.35756
C	5.00608	-0.52586	2.89473
H	4.86065	-1.5786	2.59866
H	6.0082	-0.20696	2.56298
H	4.92398	-0.43513	3.98799
C	2.74043	1.90945	-0.70198
C	3.89267	1.92138	-1.69344
H	4.41246	0.95533	-1.69021
H	3.5566	2.15561	-2.71378
H	4.61842	2.69104	-1.37001
C	1.88051	3.13278	-0.66607
H	1.73232	3.55136	0.33689
C	1.26453	3.79582	-1.66335
C	1.29151	3.32725	-3.02244
N	1.2451	2.99448	-4.13164
C	0.42145	5.0261	-1.43
H	0.49509	5.34914	-0.37936
H	0.74622	5.86205	-2.07332
H	-0.63937	4.81844	-1.66183

B$^{R\text{-}anti}$

C	2.9856	-0.35395	2.04436
C	0.38567	-0.46209	2.90594
C	1.46313	-0.49829	3.99456
C	2.66337	0.25974	3.4246
H	3.45083	-1.34061	2.21549
H	0.02202	0.57678	2.8377
H	1.09295	-0.04761	4.93155
H	1.76489	-1.53614	4.22478
H	2.40301	1.32545	3.29853
H	3.5467	0.21202	4.08377
C	1.39198	-2.54292	1.02165
C	0.17231	-3.13	0.29619
H	2.30352	-2.69439	0.41566
H	1.54936	-3.06404	1.98276
H	0.43241	-4.10143	-0.16069
H	-0.64252	-3.31703	1.01856
C	0.33776	-2.35014	-2.57049
C	-2.11044	-2.92067	-1.4847
C	-0.41813	-3.52537	-3.19734
H	0.14002	-1.45484	-3.18685
C	-1.90316	-3.22192	-2.98674
H	-2.07576	-3.88147	-0.942
H	-0.16487	-4.47425	-2.69078
H	-0.16307	-3.64574	-4.26442
H	-2.55513	-4.05193	-3.30749
H	-2.19196	-2.33637	-3.58129
P	-0.5699	-1.98585	-0.9654
P	1.30671	-0.70641	1.29078
Cu	0.05205	0.18786	-0.39675

H	-2.38923	2.24078	-0.48679
C	1.82554	-2.43253	-2.34365
C	4.55122	-2.4548	-1.64807
C	2.53715	-1.23411	-2.17667
C	2.50983	-3.6417	-2.18701
C	3.86358	-3.65197	-1.84063
C	3.88389	-1.24117	-1.82729
H	2.01591	-0.28019	-2.31714
H	1.989	-4.59328	-2.3249
H	4.38175	-4.60779	-1.71818
H	4.41371	-0.29454	-1.68687
H	5.60813	-2.4626	-1.36679
C	-3.41981	-2.24106	-1.15517
C	-5.88062	-1.03031	-0.5365
C	-3.5896	-0.85613	-1.28348
C	-4.50806	-3.00937	-0.72722
C	-5.73044	-2.41193	-0.42002
C	-4.80754	-0.25122	-0.974
H	-2.74904	-0.23293	-1.60665
H	-4.39199	-4.09306	-0.61836
H	-6.56728	-3.02974	-0.08074
H	-4.92208	0.83326	-1.07204
H	-6.8344	-0.55543	-0.28991
C	3.91921	0.47039	1.19103
C	5.68723	2.01279	-0.35864
C	3.46584	1.59655	0.49538
C	5.27355	0.13018	1.10058
C	6.15279	0.89301	0.33165
C	4.33884	2.36412	-0.27283
H	2.40778	1.86963	0.52515
H	5.64474	-0.75247	1.63236
H	7.20736	0.60821	0.26924
H	3.93973	3.2275	-0.8117
H	6.37498	2.61004	-0.96454
C	-0.81531	-1.36703	3.00386
C	-3.04696	-3.08309	2.9413
C	-1.96675	-1.03126	2.27582
C	-0.81	-2.5706	3.71628
C	-1.9158	-3.42382	3.68147
C	-3.07281	-1.87422	2.243
H	-1.98858	-0.09129	1.71588
H	0.0652	-2.85985	4.30434
H	-1.89008	-4.36295	4.24226
H	-3.9555	-1.5868	1.66537
H	-3.91211	-3.75171	2.91339
C	-1.60325	4.13071	-1.02572
H	-0.69552	4.73783	-1.12189
C	-2.7691	4.6647	-1.43386
C	-3.97674	3.88647	-1.31908
N	-4.95085	3.26453	-1.23203
C	-2.91477	6.04798	-2.01782
H	-3.59588	6.66747	-1.40846
H	-1.93385	6.5472	-2.05944
H	-3.32896	6.00741	-3.04049
C	-1.03823	2.84254	1.01171
C	0.22139	3.28398	1.44137
C	-1.99601	2.5254	1.98282
C	0.51548	3.38177	2.80053
H	0.96312	3.56032	0.68763
C	-1.70198	2.61472	3.34475
H	-2.99351	2.2006	1.66755
C	-0.4419	3.0428	3.7596
H	1.50396	3.7334	3.1126
H	-2.46408	2.34711	4.08237
H	-0.2077	3.11748	4.82571
C	-1.41036	2.74547	-0.46517
C	-0.5014	1.88214	-1.36318
H	-1.08238	1.49926	-2.2171
C	0.74272	2.44535	-1.8639
O	1.1754	1.78403	-2.9792
O	1.40824	3.36496	-1.40932
C	2.41102	2.19225	-3.52069
H	2.5373	1.62516	-4.4549
H	2.41824	3.27456	-3.73221
H	3.24476	1.96297	-2.83409

B$^{S\text{-}anti}$

C	-1.18257	2.25618	2.47332
C	0.70336	0.42851	3.26582
C	0.20983	1.40985	4.33511
C	-1.21812	1.77647	3.93439
H	-0.77808	3.28464	2.45514
H	0.10999	-0.49794	3.35574
H	0.25784	0.96083	5.34223
H	0.83153	2.32298	4.3533
H	-1.84219	0.87026	4.01703
H	-1.66045	2.54157	4.59443
C	1.46143	2.3569	1.15512
C	2.59278	1.67784	0.36352
H	0.97229	3.12448	0.52615
H	1.87582	2.87612	2.03764
H	3.25781	2.44239	-0.07361
H	3.21121	1.06426	1.03981
C	2.02563	1.52103	-2.52945
C	3.47195	-0.50238	-1.39089
C	3.47795	1.40398	-3.00116
H	1.39409	0.93749	-3.21954

C	3.84842	-0.06885	-2.82923
H	4.27358	-0.17836	-0.70518
H	4.14809	2.02815	-2.38275
H	3.58985	1.74207	-4.04602
H	4.91632	-0.26385	-3.02454
H	3.27379	-0.67774	-3.55004
P	1.95694	0.51794	-0.94756
P	0.1126	1.16846	1.64543
Cu	-0.18849	0.0033	-0.29943
H	-3.44477	-0.48886	0.02192
C	1.39852	2.88216	-2.37931
C	0.12109	5.3555	-1.9671
C	0.00508	2.99763	-2.49954
C	2.14042	4.02793	-2.06893
C	1.50696	5.25373	-1.85879
C	-0.6253	4.22366	-2.29876
H	-0.58124	2.10851	-2.75806
H	3.22991	3.97537	-1.99121
H	2.10478	6.13692	-1.61511
H	-1.71169	4.29723	-2.40302
H	-0.37604	6.31618	-1.8057
C	3.28579	-1.99615	-1.24568
C	2.98511	-4.7854	-1.02478
C	4.30618	-2.78599	-0.70498
C	2.12093	-2.62982	-1.6945
C	1.96858	-4.01011	-1.58411
C	4.15881	-4.16888	-0.59067
H	5.2304	-2.30878	-0.36188
H	1.29681	-2.05478	-2.12741
H	1.04134	-4.46886	-1.9389
H	4.96689	-4.76758	-0.15961
H	2.86631	-5.86895	-0.93318
C	-2.4882	2.24193	1.70609
C	-4.8293	2.13008	0.14576
C	-2.49758	2.70916	0.38522
C	-3.68138	1.74502	2.23883
C	-4.84181	1.68599	1.46537
C	-3.6521	2.65426	-0.3891
H	-1.57601	3.10391	-0.05536
H	-3.72189	1.38504	3.26954
H	-5.7556	1.25942	1.88486
H	-3.62867	3.00576	-1.42507
H	-5.73386	2.05597	-0.46343
C	2.16024	0.04798	3.24418
C	4.87359	-0.67139	3.02523
C	2.54243	-1.12642	2.57921
C	3.16033	0.84845	3.8075
C	4.50633	0.49566	3.6929
C	3.88264	-1.48719	2.47584
H	1.77255	-1.75971	2.12754
H	2.89791	1.76465	4.34259
H	5.27255	1.13804	4.13691
H	4.14891	-2.41287	1.96016
H	5.92793	-0.95097	2.943
O	-1.00985	-2.23951	-2.72379
C	-1.27257	-1.05312	-2.54885
C	-1.86616	-0.41796	-1.39785
O	-0.93545	-0.14831	-3.5262
H	-2.30745	0.55133	-1.66248
C	-0.28633	-0.6697	-4.66036
H	-0.13101	0.18071	-5.34157
H	0.68881	-1.12189	-4.40215
H	-0.89654	-1.4428	-5.15686
C	-3.63334	-2.23835	-1.18014
H	-3.09398	-2.88739	-1.8796
C	-4.95835	-2.42606	-1.0467
C	-5.72235	-1.63064	-0.11928
N	-6.37863	-1.02723	0.62127
C	-5.74015	-3.46735	-1.81121
H	-6.53889	-3.00216	-2.41523
H	-5.07148	-4.02347	-2.4871
H	-6.22082	-4.18682	-1.12518
C	-2.78039	-1.22617	-0.45729
C	-2.03338	-1.92451	0.67702
C	-2.46423	-1.78122	2.00147
C	-0.90307	-2.71446	0.42062
C	-1.77469	-2.39764	3.04666
H	-3.35166	-1.17552	2.21151
C	-0.21254	-3.32973	1.4652
H	-0.58174	-2.85707	-0.61573
C	-0.64382	-3.17256	2.78337
H	-2.12529	-2.27242	4.0761
H	0.66886	-3.93855	1.23861
H	-0.101	-3.65254	3.60307

B$^{R\text{-}syn}$

C	-1.79334	-3.07111	-1.02985
C	0.4253	-2.25805	-2.40846
C	-0.28574	-3.5036	-2.94483
C	-1.75486	-3.33946	-2.54938
H	-1.54613	-4.01803	-0.51826
H	0.07216	-1.39724	-2.99936
H	-0.15311	-3.60001	-4.03636
H	0.11681	-4.42334	-2.48298
H	-2.19497	-2.47815	-3.08443
H	-2.3638	-4.22474	-2.79857
C	0.66816	-2.93133	0.49697

C	1.90041	-2.19877	1.04872
H	-0.03718	-3.15415	1.31828
H	0.96874	-3.89855	0.05642
H	2.21608	-2.65107	2.00578
H	2.74635	-2.29894	0.3446
C	0.93931	-0.09128	2.94338
C	3.38948	0.18838	1.74995
C	2.1402	0.0471	3.8844
H	0.46957	0.90366	2.8602
C	3.17953	0.86977	3.12021
H	3.96752	-0.73669	1.92089
H	2.56969	-0.94033	4.13238
H	1.84637	0.52597	4.83433
H	4.13704	0.95248	3.66195
H	2.79947	1.89554	2.96508
P	1.67334	-0.36605	1.24008
P	-0.33187	-1.93784	-0.71633
Cu	0.16993	0.2981	-0.33578
C	-0.1472	-1.09103	3.24342
C	-2.19539	-2.99644	3.55095
C	-1.40629	-0.90136	2.65483
C	0.06236	-2.24525	4.00458
C	-0.95405	-3.19237	4.15415
C	-2.42252	-1.83862	2.80434
H	-1.58659	-0.00295	2.05679
H	1.02793	-2.42191	4.48643
H	-0.7699	-4.09116	4.75034
H	-3.39178	-1.66654	2.3291
H	-2.98972	-3.73973	3.66407
C	4.12957	1.02792	0.73614
C	5.54482	2.59431	-1.12359
C	3.48782	2.0484	0.02398
C	5.49209	0.81027	0.50583
C	6.19712	1.58587	-0.41477
C	4.186	2.82288	-0.89974
H	2.41643	2.21205	0.16911
H	6.00892	0.01192	1.04874
H	7.26118	1.39605	-0.58361
H	3.66084	3.60399	-1.45712
H	6.0927	3.19866	-1.85217
C	-3.12126	-2.58183	-0.50411
C	-5.60838	-1.72614	0.47868
C	-3.94818	-3.45792	0.20764
C	-3.56369	-1.27195	-0.73373
C	-4.79738	-0.84578	-0.24149
C	-5.18551	-3.0372	0.69548
H	-3.61371	-4.48451	0.39281
H	-2.95337	-0.56469	-1.3091
H	-5.12491	0.18154	-0.42885
H	-5.81723	-3.73592	1.25245
H	-6.57496	-1.38798	0.86345
C	1.92879	-2.19112	-2.36653
C	4.70717	-1.92817	-2.01848
C	2.53921	-0.92682	-2.38041
C	2.73729	-3.32187	-2.2109
C	4.11704	-3.19115	-2.03747
C	3.91314	-0.79429	-2.20118
H	1.92026	-0.0345	-2.53425
H	2.29292	-4.32115	-2.21239
H	4.73312	-4.08678	-1.91303
H	4.36596	0.20115	-2.1997
H	5.78611	-1.82373	-1.87218
O	-2.17939	1.03701	-2.76291
C	-1.03556	1.38674	-2.53073
C	-0.50864	1.94325	-1.29381
O	-0.06372	1.22237	-3.48208
H	0.36271	2.58666	-1.49595
C	-0.48849	0.70259	-4.72076
H	0.41554	0.61062	-5.34092
H	-0.96781	-0.28543	-4.60802
H	-1.21397	1.37584	-5.20869
C	-2.06111	3.88343	-1.06467
H	-1.28879	4.63152	-1.28904
C	-3.32296	4.16346	-1.43385
C	-4.37751	3.23197	-1.12428
N	-5.25404	2.52416	-0.85618
C	-3.74116	5.42433	-2.14824
H	-4.49279	5.98343	-1.56401
H	-2.87013	6.07838	-2.31312
H	-4.19087	5.18988	-3.12889
C	-1.57356	2.61338	-0.41736
C	-1.09376	2.90653	0.99576
C	-1.90356	2.59809	2.09422
C	0.14943	3.50577	1.23885
C	-1.4742	2.84849	3.39884
H	-2.88587	2.14574	1.9225
C	0.58077	3.76647	2.53815
H	0.79112	3.77715	0.39584
C	-0.22824	3.43139	3.62634
H	-2.11855	2.58179	4.24144
H	1.55604	4.23535	2.70176
H	0.11086	3.62853	4.64748
H	-2.42779	1.92255	-0.34014

B*^S-syn^*

C	1.57909	-2.39291	1.95601
C	-0.48277	-0.90444	2.96333

C	0.20662	-1.84895	3.95101
C	1.6451	-1.98817	3.44642
H	1.27451	-3.45344	1.91179
H	-0.03363	0.09501	3.09647
H	0.16319	-1.45179	4.97991
H	-0.27713	-2.84276	3.96006
H	2.16497	-1.01761	3.53859
H	2.22657	-2.72877	4.02139
C	-0.99919	-2.79378	0.70184
C	-2.23678	-2.32363	-0.0809
H	-0.36183	-3.42177	0.05326
H	-1.30586	-3.41942	1.55901
H	-2.6421	-3.1571	-0.68195
H	-3.03049	-2.01015	0.62187
C	-1.28125	-1.46107	-2.77893
C	-3.61368	-0.40052	-1.81328
C	-2.53991	-1.75387	-3.60115
H	-0.79913	-0.56573	-3.21186
C	-3.48166	-0.57669	-3.34401
H	-4.30622	-1.17068	-1.43146
H	-3.02385	-2.69101	-3.27062
H	-2.30072	-1.8677	-4.67261
H	-4.47479	-0.72041	-3.80201
H	-3.0486	0.34098	-3.77933
P	-1.91889	-0.82654	-1.13414
P	0.12765	-1.42496	1.26991
Cu	-0.19661	0.3333	-0.14854
C	-0.23429	-2.52922	-2.60068
C	1.73942	-4.45752	-2.04017
C	1.0702	-2.14691	-2.25017
C	-0.52487	-3.89522	-2.68551
C	0.45288	-4.85189	-2.40364
C	2.04839	-3.09783	-1.97357
H	1.32472	-1.08312	-2.1783
H	-1.52706	-4.22801	-2.96877
H	0.20333	-5.91504	-2.47193
H	3.05604	-2.77368	-1.70045
H	2.50594	-5.2049	-1.81649
C	-4.11111	0.95883	-1.38334
C	-5.03583	3.48234	-0.56477
C	-3.29754	2.09309	-1.52109
C	-5.39503	1.11101	-0.85194
C	-5.85889	2.36358	-0.44651
C	-3.75636	3.34197	-1.10798
H	-2.29277	2.00287	-1.95418
H	-6.04009	0.23283	-0.74134
H	-6.86597	2.46273	-0.03041
H	-3.10669	4.21546	-1.21725
H	-5.39274	4.46479	-0.24193
C	2.88567	-2.24871	1.20941
C	5.34023	-2.01969	-0.14336
C	3.27139	-1.03985	0.62025
C	3.75388	-3.34192	1.11416
C	4.97231	-3.23066	0.44416
C	4.4854	-0.91952	-0.05422
H	2.59015	-0.1866	0.66019
H	3.46651	-4.2997	1.56133
H	5.63526	-4.09838	0.37547
H	4.75889	0.03217	-0.52155
H	6.29135	-1.93139	-0.67598
C	-1.97991	-0.74436	2.964
C	-4.76288	-0.44607	2.66698
C	-2.52459	0.3979	2.35736
C	-2.85619	-1.72322	3.44239
C	-4.2376	-1.57531	3.29332
C	-3.89857	0.54885	2.20498
H	-1.85207	1.17771	1.98526
H	-2.46588	-2.6202	3.93117
H	-4.90725	-2.35399	3.67055
H	-4.29407	1.44287	1.71589
H	-5.84413	-0.33479	2.5445
C	2.26394	3.88242	-0.33317
H	1.56613	4.70856	-0.14155
C	3.42571	4.1773	-0.94478
C	4.3786	3.12715	-1.20246
N	5.17025	2.30609	-1.40686
C	3.82573	5.56134	-1.39237
H	3.98587	5.59356	-2.48434
H	3.03907	6.28863	-1.13611
H	4.76631	5.88018	-0.91019
C	1.78674	2.51159	0.06494
C	0.54594	2.11396	-0.74071
H	-0.2973	2.81306	-0.62315
C	0.72071	1.83053	-2.15839
O	1.9894	1.45592	-2.47321
O	-0.14963	1.84394	-3.01885
C	2.25914	1.14892	-3.82221
H	3.34462	0.98345	-3.88106
H	1.72313	0.23795	-4.1421
H	1.96257	1.97853	-4.48498
C	1.5661	2.44631	1.56953
C	2.58417	1.9708	2.40476
C	0.36384	2.85453	2.16183
C	2.3982	1.87265	3.78404
H	3.53828	1.66289	1.96533
C	0.17555	2.76772	3.54122
H	-0.44788	3.23571	1.53565
C	1.18973	2.26716	4.35842

H	3.20568	1.48584	4.41295
H	-0.77668	3.08154	3.97907
H	1.03948	2.18926	5.43908
H	2.58797	1.79874	-0.17775

$^{L3}TS_{B\text{-}C}^{R\text{-}E}$

C	3.70848	1.58077	-0.5789
C	1.6842	3.0968	-1.61785
C	3.07199	3.74067	-1.65903
C	4.05999	2.57194	-1.71696
H	4.14301	1.96217	0.36142
H	1.52374	2.57672	-2.57952
H	3.18105	4.41895	-2.52308
H	3.2586	4.33783	-0.74795
H	3.95999	2.05511	-2.68814
H	5.10966	2.90099	-1.63457
C	1.6259	2.4618	1.2842
C	0.16441	2.48471	1.76699
H	2.23583	1.84002	1.96345
H	2.04694	3.48265	1.31008
H	0.13444	2.58843	2.86639
H	-0.36009	3.35978	1.34231
C	-0.55165	-0.3116	2.51415
C	-2.55511	1.45601	1.89834
C	-1.588	-0.05326	3.61086
H	-0.84407	-1.24079	1.99479
C	-2.88234	0.30928	2.88065
H	-2.42911	2.3809	2.48807
H	-1.28615	0.79123	4.25712
H	-1.70695	-0.94056	4.2555
H	-3.68949	0.61031	3.56963
H	-3.24514	-0.56899	2.31641
P	-0.85631	1.02506	1.23313
P	1.8472	1.69153	-0.3897
C	0.90765	-0.43001	2.86784
C	3.68092	-0.55128	3.33424
C	1.76526	-1.06861	1.95848
C	1.46146	0.12998	4.0229
C	2.83899	0.07414	4.25225
C	3.13559	-1.13282	2.18703
H	1.34555	-1.51342	1.04962
H	0.81995	0.62339	4.75815
H	3.25362	0.52189	5.16047
H	3.78041	-1.63761	1.46246
H	4.75901	-0.596	3.51438
C	-3.59475	1.70535	0.83235
C	-5.53132	2.19476	-1.14634
C	-3.77399	0.8129	-0.23237
C	-4.41066	2.84006	0.89551
C	-5.37326	3.08523	-0.08431
C	-4.7299	1.0535	-1.21654
H	-3.15053	-0.0826	-0.32249
H	-4.28264	3.55045	1.71931
H	-5.99876	3.98075	-0.01937
H	-4.82955	0.33356	-2.03318
H	-6.28071	2.38854	-1.91942
C	4.21617	0.17259	-0.80406
C	5.16684	-2.45093	-1.16062
C	5.4549	-0.21661	-0.28303
C	3.47296	-0.76729	-1.52978
C	3.93592	-2.07095	-1.69743
C	5.93073	-1.51572	-0.46151
H	6.04839	0.50386	0.28998
H	2.49929	-0.49141	-1.94709
H	3.31412	-2.78945	-2.23945
H	6.89846	-1.80268	-0.03948
H	5.53045	-3.47494	-1.28599
C	0.45625	3.91866	-1.32365
C	-1.92153	5.21162	-0.5595
C	-0.79711	3.38679	-1.66808
C	0.49781	5.12705	-0.6218
C	-0.68399	5.76968	-0.24326
C	-1.9753	4.01877	-1.284
H	-0.85021	2.45125	-2.23689
H	1.45847	5.57541	-0.35295
H	-0.63244	6.71298	0.30871
H	-2.93959	3.57228	-1.54316
H	-2.84705	5.70604	-0.25083
Cu	0.02614	0.34505	-0.72774
O	-0.08553	-0.4226	-2.59549
C	-0.03297	-1.64516	-2.23929
C	-0.55512	-2.15828	-1.06584
O	0.63167	-2.5194	-3.04312
H	-1.3021	-1.53768	-0.56281
C	1.13083	-2.01985	-4.26142
H	1.88794	-1.23206	-4.1047
H	0.32296	-1.60556	-4.88758
H	1.59394	-2.87726	-4.77307
C	-0.50088	-3.61178	-0.67896
H	0.52745	-3.98685	-0.82666
C	-0.82017	-3.74728	0.80695
C	-2.12636	-3.53957	1.27218
C	0.1878	-3.98834	1.74391
C	-2.41103	-3.55771	2.63625
H	-2.9314	-3.34942	0.55497
C	-0.09026	-3.99984	3.11145
H	1.21498	-4.1443	1.39975

C	-1.39099	-3.78401	3.56289
H	-3.43692	-3.39064	2.97851
H	0.71905	-4.1707	3.82705
H	-1.61128	-3.79431	4.63446
C	-1.38364	-4.54816	-1.48135
H	-1.0841	-5.6041	-1.452
C	-2.48758	-4.27077	-2.19479
C	-3.27515	-5.3234	-2.94168
H	-2.807	-6.3136	-2.82441
H	-4.31302	-5.38069	-2.56937
H	-3.32508	-5.086	-4.01879
C	-3.0602	-2.94772	-2.28537
N	-3.66933	-1.9694	-2.40693

$^{L3}TS_{B-C}{}^{S-E}$

C	1.85507	-3.50719	-0.62814
C	3.18603	-1.42118	-1.79242
C	3.9071	-2.76956	-1.85609
C	2.799	-3.82561	-1.8167
H	2.29953	-3.91741	0.29506
H	2.58766	-1.31045	-2.71491
H	4.52945	-2.85664	-2.76364
H	4.57635	-2.90165	-0.98664
H	2.21907	-3.78127	-2.75576
H	3.19554	-4.85147	-1.73309
C	2.78567	-1.35376	1.14338
C	2.7517	0.11262	1.61255
H	2.27125	-1.99562	1.88008
H	3.83133	-1.70345	1.08071
H	2.9594	0.16514	2.69614
H	3.54068	0.69238	1.09957
C	-0.02133	0.62138	2.60589
C	1.51796	2.75006	1.82918
C	0.17954	1.73069	3.64366
H	-1.00216	0.79087	2.13365
C	0.38662	3.01999	2.84556
H	2.46996	2.69917	2.38646
H	1.07181	1.53743	4.26659
H	-0.68962	1.80124	4.31988
H	0.64508	3.87704	3.48968
H	-0.54885	3.27845	2.31717
P	1.17571	1.01484	1.2173
P	1.88991	-1.64034	-0.45888
C	0.04504	-0.82596	3.0188
C	0.2433	-3.57286	3.6021
C	-0.56179	-1.78507	2.19293
C	0.7428	-1.2669	4.14764
C	0.84566	-2.63053	4.4341
C	-0.47054	-3.14286	2.48115
H	-1.10689	-1.45709	1.30142
H	1.21931	-0.5463	4.81768
H	1.39872	-2.95538	5.32043
H	-0.95596	-3.86696	1.8214
H	0.32077	-4.63985	3.83038
C	1.66047	3.7769	0.73222
C	1.95266	5.69544	-1.30289
C	0.70672	3.88764	-0.28831
C	2.75409	4.64868	0.72127
C	2.90123	5.60343	-0.28532
C	0.85343	4.83434	-1.29932
H	-0.15604	3.2142	-0.30744
H	3.50972	4.5734	1.51029
H	3.7651	6.27472	-0.27637
H	0.10281	4.89618	-2.09209
H	2.06744	6.43787	-2.09787
C	0.46044	-4.07456	-0.78727
C	-2.12973	-5.13894	-1.05768
C	0.13392	-5.31307	-0.2241
C	-0.52638	-3.39001	-1.50747
C	-1.81287	-3.90921	-1.63627
C	-1.14926	-5.84399	-0.35832
H	0.89159	-5.86273	0.34471
H	-0.29938	-2.41888	-1.95672
H	-2.56302	-3.33246	-2.18518
H	-1.38694	-6.81099	0.09496
H	-3.13937	-5.54903	-1.15262
C	3.95365	-0.14256	-1.57896
C	5.1594	2.31821	-0.948
C	3.32166	1.0714	-1.894
C	5.21447	-0.10227	-0.97631
C	5.81378	1.12083	-0.66379
C	3.91166	2.29007	-1.57487
H	2.34269	1.05859	-2.38669
H	5.73967	-1.03048	-0.7342
H	6.79949	1.13336	-0.18916
H	3.39005	3.22266	-1.80824
H	5.62087	3.27584	-0.69053
Cu	0.41181	0.09584	-0.68331
O	-0.3743	0.295	-2.52095
C	-1.56405	-0.04714	-2.20672
C	-2.17992	0.2266	-0.99945
O	-2.26612	-0.79049	-3.10465
H	-1.71812	1.01407	-0.38998
C	-1.63706	-1.09048	-4.32735
H	-1.38342	-0.17463	-4.887
H	-2.36201	-1.68636	-4.90206
H	-0.7122	-1.67558	-4.1813

C	-3.60256	-0.13954	-0.66503
H	-3.88481	-0.96948	-1.33351
C	-4.64143	0.96552	-0.90013
C	-5.96869	0.77534	-0.49298
C	-4.31389	2.15432	-1.553
C	-6.93883	1.74726	-0.72454
H	-6.24801	-0.15049	0.02168
C	-5.28134	3.13265	-1.78688
H	-3.28193	2.31316	-1.87599
C	-6.59689	2.93502	-1.37322
H	-7.96874	1.57784	-0.39554
H	-5.00044	4.06069	-2.29407
H	-7.35455	3.70357	-1.55272
C	-3.6497	-0.68917	0.74535
H	-3.68701	-1.7813	0.84062
C	-3.58891	0.00752	1.89559
C	-3.6003	-0.62274	3.26699
H	-2.68676	-0.3673	3.83271
H	-4.46697	-0.27218	3.855
H	-3.65168	-1.71991	3.18504
C	-3.41596	1.43809	1.88077
N	-3.18051	2.57165	1.94068

$^{L3}TS_{B-C}^{R-Z}$

C	-3.56653	-1.89979	-0.45477
C	-1.35804	-3.30569	-1.25874
C	-2.68595	-4.06825	-1.27606
C	-3.76693	-3.00902	-1.51164
H	-3.91769	-2.29779	0.51321
H	-1.173	-2.94124	-2.28439
H	-2.69195	-4.85261	-2.0525
H	-2.8702	-4.56587	-0.30685
H	-3.65613	-2.57784	-2.52343
H	-4.78708	-3.42382	-1.44777
C	-1.4712	-2.20053	1.50042
C	-0.03931	-2.02339	2.01908
H	-2.15787	-1.53389	2.05265
H	-1.81184	-3.23659	1.67614
H	-0.03654	-2.018	3.12354
H	0.58883	-2.87253	1.69549
C	0.44508	0.86258	2.56847
C	2.56158	-0.78801	2.02826
C	1.50877	0.75629	3.66478
H	0.66135	1.78482	2.00629
C	2.82172	0.4332	2.94013
H	2.48516	-1.67257	2.68513
H	1.27487	-0.05848	4.37433
H	1.5679	1.69524	4.24035
H	3.64713	0.22158	3.64087
H	3.13363	1.29286	2.32199
P	0.83363	-0.51739	1.37144
P	-1.70595	-1.73916	-0.28372
C	-1.02493	0.87039	2.8953
C	-3.80812	0.67535	3.26611
C	-1.91894	1.35171	1.92526
C	-1.55033	0.318	4.0667
C	-2.93205	0.21998	4.25025
C	-3.29499	1.25244	2.10239
H	-1.53362	1.79553	1.00151
H	-0.88085	-0.05043	4.84892
H	-3.32396	-0.21816	5.17307
H	-3.96834	1.62248	1.32409
H	-4.8897	0.59194	3.4069
C	3.62577	-1.07833	0.99231
C	5.63624	-1.69806	-0.87657
C	3.60668	-0.51693	-0.28971
C	4.67329	-1.94777	1.324
C	5.67216	-2.25621	0.40231
C	4.60132	-0.82759	-1.21645
H	2.79812	0.15169	-0.59683
H	4.69884	-2.40261	2.32032
H	6.47774	-2.94195	0.68149
H	4.55106	-0.38101	-2.21291
H	6.41282	-1.94441	-1.60665
C	-4.308	-0.61223	-0.72705
C	-5.72896	1.76729	-1.20373
C	-5.53149	-0.3687	-0.09207
C	-3.81237	0.35011	-1.61407
C	-4.51439	1.53084	-1.84834
C	-6.23915	0.8097	-0.32662
H	-5.93031	-1.10861	0.61016
H	-2.85147	0.20695	-2.11579
H	-4.09645	2.26896	-2.5386
H	-7.19031	0.98398	0.18506
H	-6.27711	2.69668	-1.38331
C	-0.09214	-3.96379	-0.77392
C	2.32184	-4.93502	0.29223
C	1.13732	-3.38753	-1.13353
C	-0.08901	-5.05175	0.1044
C	1.11029	-5.53331	0.63479
C	2.33302	-3.86369	-0.60336
H	1.15618	-2.53762	-1.82612
H	-1.02915	-5.53018	0.39268
H	1.09316	-6.38271	1.32419
H	3.27743	-3.38985	-0.88526
H	3.26055	-5.30471	0.71449
Cu	-0.0502	-0.20475	-0.67919

O	0.73404	0.24457	-2.48838
C	-0.20799	1.10112	-2.61472
C	-0.45903	2.2098	-1.8294
O	-1.12832	0.89033	-3.6119
H	-1.38374	2.74541	-2.06067
C	-1.06404	-0.34127	-4.2818
H	-1.21597	-1.1899	-3.58829
H	-0.09673	-0.48483	-4.79144
H	-1.87761	-0.32877	-5.0231
C	0.49434	2.91022	-0.88039
H	0.36613	2.56512	0.16112
C	1.99405	2.80292	-1.1595
C	2.52184	2.66821	-2.44596
C	2.88167	2.92041	-0.08256
C	3.90199	2.6348	-2.64476
H	1.84515	2.58375	-3.29906
C	4.26048	2.88521	-0.27646
H	2.48217	3.05258	0.92892
C	4.7766	2.73694	-1.56306
H	4.29745	2.52028	-3.65863
H	4.9336	2.96512	0.58208
H	5.85814	2.69713	-1.72176
C	0.16318	4.38316	-0.88304
H	0.09044	4.84513	-1.87587
C	-0.02833	5.17009	0.19081
C	-0.33715	6.64502	0.11867
H	-1.29202	6.8757	0.62222
H	0.45099	7.23961	0.61281
H	-0.4098	6.96891	-0.93154
C	0.10555	4.61442	1.51376
N	0.2404	4.19461	2.58584

$^{L3}TS_{B-C}^{S-Z}$

C	4.01252	-0.5326	-0.52566
C	2.94387	1.7129	-1.67265
C	4.46647	1.57623	-1.7565
C	4.74882	0.0711	-1.74333
H	4.58396	-0.25676	0.37777
H	2.51836	1.33991	-2.61992
H	4.867	2.06799	-2.65981
H	4.95578	2.04651	-0.88439
H	4.36477	-0.39066	-2.67098
H	5.82714	-0.15655	-1.69397
C	2.59306	1.25177	1.23951
C	1.35084	2.01802	1.71054
H	2.81742	0.4281	1.9404
H	3.47009	1.92338	1.2362
H	1.40668	2.19158	2.80012
H	1.30943	3.00904	1.22404
C	-0.63881	0.03518	2.68546
C	-1.47256	2.52371	1.89638
C	-1.36256	0.87404	3.74334
H	-1.37697	-0.65739	2.25168
C	-2.30811	1.80128	2.97532
H	-0.85009	3.27924	2.40661
H	-0.65082	1.48447	4.32852
H	-1.90501	0.22616	4.45296
H	-2.80544	2.53468	3.63244
H	-3.09839	1.20265	2.49057
P	-0.2715	1.2196	1.28617
P	2.40394	0.42715	-0.41352
C	0.58347	-0.76581	3.05458
C	2.96644	-2.18994	3.52316
C	0.99949	-1.78599	2.18437
C	1.37317	-0.48559	4.17388
C	2.55796	-1.19	4.40413
C	2.17541	-2.49307	2.41286
H	0.39572	-2.01495	1.3013
H	1.07288	0.29394	4.87931
H	3.16387	-0.95314	5.28385
H	2.47815	-3.28012	1.71672
H	3.89463	-2.74015	3.70246
C	-2.27059	3.22278	0.82354
C	-3.75146	4.55224	-1.16439
C	-2.86335	2.51067	-0.22527
C	-2.44224	4.61046	0.86619
C	-3.17785	5.27248	-0.11686
C	-3.59264	3.16635	-1.21412
H	-2.72869	1.42737	-0.28277
H	-1.98167	5.18497	1.67678
H	-3.29673	6.35889	-0.06769
H	-4.03147	2.58956	-2.03309
H	-4.32039	5.0696	-1.94213
C	3.87333	-2.03733	-0.54798
C	3.66218	-4.84274	-0.55158
C	4.81349	-2.82806	0.12319
C	2.83266	-2.67545	-1.23265
C	2.72514	-4.06505	-1.23266
C	4.71216	-4.21881	0.12311
H	5.6311	-2.3453	0.66924
H	2.07045	-2.09493	-1.75873
H	1.89427	-4.53354	-1.76752
H	5.45324	-4.81803	0.66034
H	3.57456	-5.93298	-0.54575
C	2.30142	3.04554	-1.38672
C	0.9045	5.36891	-0.64136
C	0.95127	3.21629	-1.73523

C	2.94915	4.07307	-0.69428
C	2.25484	5.22796	-0.3249
C	0.25579	4.3612	-1.3585
H	0.43731	2.43088	-2.30137
H	4.00464	3.97688	-0.42449
H	2.7764	6.02065	0.21981
H	-0.80108	4.46353	-1.62121
H	0.35677	6.2666	-0.34038
Cu	0.1022	0.22524	-0.6987
O	-0.46084	0.12231	-2.62489
C	-0.48229	-1.16116	-2.63987
C	-1.29374	-1.97926	-1.88268
O	0.40809	-1.80425	-3.45529
H	-1.20266	-3.05749	-2.04057
C	1.30372	-1.01218	-4.18868
H	2.02709	-0.49554	-3.53084
H	0.78299	-0.2518	-4.79401
H	1.85351	-1.70217	-4.84669
C	-2.57063	-1.45416	-1.26823
H	-2.52204	-0.35366	-1.35078
C	-2.81828	-1.78869	0.1927
C	-2.11523	-2.79652	0.85549
C	-3.80676	-1.09123	0.89971
C	-2.39496	-3.1087	2.18583
H	-1.34299	-3.34888	0.31475
C	-4.09124	-1.39844	2.2282
H	-4.37852	-0.30708	0.39136
C	-3.38361	-2.41137	2.8775
H	-1.83453	-3.90413	2.68492
H	-4.87341	-0.8477	2.75935
H	-3.60811	-2.66067	3.91846
C	-3.69605	-1.86282	-2.19401
H	-3.65844	-1.33898	-3.15728
C	-4.65421	-2.80145	-2.07337
C	-5.63043	-3.11354	-3.18645
H	-5.53263	-4.16355	-3.51402
H	-6.67272	-2.96999	-2.85143
H	-5.44918	-2.46094	-4.0554
C	-4.83826	-3.61418	-0.89667
N	-5.0752	-4.3265	-0.01388

C*R-Z*

C	0.45091	-3.2269	1.60197
C	-1.436	-1.68385	2.85737
C	-1.09813	-3.01768	3.53119
C	0.34992	-3.3266	3.14142
H	-0.01953	-4.13097	1.17651
H	-0.84608	-0.89447	3.35578
H	-1.23601	-2.96641	4.62532
H	-1.75236	-3.82537	3.15631
H	1.02843	-2.58509	3.60118
H	0.67748	-4.32116	3.48907
C	-1.95874	-2.62207	0.09035
C	-2.9132	-1.64359	-0.61612
H	-1.38462	-3.19473	-0.65928
H	-2.53587	-3.35238	0.6849
H	-3.42834	-2.15731	-1.44721
H	-3.68995	-1.29656	0.08861
C	-1.42114	-0.51444	-2.9219
C	-3.45324	0.97953	-1.83969
C	-2.59729	-0.20797	-3.85427
H	-0.64053	0.25058	-3.07947
C	-3.19167	1.11198	-3.35921
H	-4.39488	0.42366	-1.69002
H	-3.36772	-0.99856	-3.7974
H	-2.27098	-0.14585	-4.90711
H	-4.1187	1.38598	-3.89091
H	-2.46513	1.92594	-3.53145
P	-2.05848	-0.10378	-1.20877
P	-0.66911	-1.79538	1.14558
Cu	-0.31685	0.25495	0.20752
H	2.75462	1.34366	0.70378
C	-0.76625	-1.87274	-2.96367
C	0.45364	-4.40062	-2.76841
C	0.52921	-2.00568	-2.43702
C	-1.42648	-3.02042	-3.41676
C	-0.82218	-4.27606	-3.31687
C	1.13107	-3.25747	-2.33809
H	1.05347	-1.11338	-2.07314
H	-2.4295	-2.94846	-3.84682
H	-1.35652	-5.16269	-3.67188
H	2.13583	-3.33772	-1.91277
H	0.9248	-5.38459	-2.68494
C	-3.53571	2.2959	-1.10296
C	-3.67283	4.73844	0.28472
C	-2.39097	3.08903	-0.93401
C	-4.74691	2.75078	-0.57424
C	-4.81869	3.96347	0.11422
C	-2.45824	4.29634	-0.24427
H	-1.42869	2.74538	-1.33073
H	-5.64896	2.14136	-0.69433
H	-5.77629	4.30141	0.52181
H	-1.55023	4.89261	-0.11357
H	-3.72522	5.68604	0.82884
C	-2.86557	-1.21276	2.77316
C	-5.48238	-0.27038	2.33222
C	-3.11006	0.14546	2.51495

C	-3.95844	-2.08394	2.83261
C	-5.25622	-1.61694	2.6127
C	-4.40178	0.61314	2.29186
H	-2.26945	0.84399	2.46415
H	-3.80553	-3.14569	3.04431
H	-6.09649	-2.31604	2.65991
H	-4.56004	1.67335	2.07571
H	-6.49887	0.09244	2.15392
O	1.30971	0.6637	-0.92016
C	1.54343	1.8362	-1.35776
C	2.48706	2.69538	-0.856
H	2.68911	3.67753	-1.28539
C	3.04659	3.30108	1.47223
H	3.85646	4.01903	1.66001
C	1.92126	3.43561	2.20548
C	0.85613	2.52351	1.90301
N	0.04557	1.75758	1.58839
C	1.68014	4.48462	3.25995
H	1.44601	4.02705	4.23695
H	2.5771	5.11253	3.3796
H	0.83358	5.13931	2.98764
C	3.24737	2.27816	0.37853
C	4.71545	1.90742	0.1911
C	5.22901	1.62396	-1.07727
C	5.55446	1.73957	1.30078
C	6.5449	1.1882	-1.23471
H	4.57127	1.7394	-1.9432
C	6.87106	1.30668	1.14876
H	5.16759	1.93258	2.3078
C	7.37222	1.02831	-0.1232
H	6.92639	0.96872	-2.23664
H	7.50708	1.17873	2.02997
H	8.4038	0.68543	-0.24654
O	0.72903	2.18501	-2.41561
C	0.86216	3.44573	-3.00354
H	1.86687	3.58574	-3.44637
H	0.10722	3.50565	-3.80347
H	0.68006	4.25893	-2.27453
C	1.86799	-3.14402	1.07174
C	2.52776	-1.92535	0.87471
C	2.55688	-4.3275	0.77751
C	3.831	-1.88602	0.38021
H	2.00635	-0.98359	1.06217
C	3.86334	-4.29547	0.29205
H	2.05436	-5.29114	0.9159
C	4.50329	-3.07206	0.08699
H	4.31388	-0.91912	0.20863
H	4.38046	-5.23184	0.06167
H	5.52319	-3.04071	-0.30692

C$^{S\text{-}Z}$

C	-2.22333	-1.12889	-1.76745
C	-0.42764	0.84472	-2.42271
C	-1.4459	0.47682	-3.50543
C	-2.68185	-0.0556	-2.78112
H	-2.0044	-2.05527	-2.33071
H	-0.79858	1.73224	-1.87538
H	-1.68526	1.34642	-4.14163
H	-1.04638	-0.31216	-4.16798
H	-3.18267	0.76328	-2.23955
H	-3.41637	-0.47021	-3.49158
C	0.55567	-1.88233	-1.82616
C	2.01323	-1.80317	-1.35754
H	0.09892	-2.82452	-1.47612
H	0.51676	-1.89499	-2.92999
H	2.52127	-2.76614	-1.54092
H	2.55795	-1.0321	-1.93187
C	2.078	-2.86556	1.44848
C	4.0833	-1.2187	0.56173
C	3.51946	-3.3519	1.65642
H	1.69699	-2.49665	2.4158
C	4.3952	-2.10566	1.78319
H	4.47634	-1.73745	-0.33036
H	3.86648	-3.93949	0.78879
H	3.59103	-4.00754	2.54101
H	5.47069	-2.34812	1.82255
H	4.15133	-1.5596	2.71306
P	2.21636	-1.29227	0.41381
P	-0.54207	-0.53688	-1.1589
Cu	0.50777	0.17312	0.80149
H	-1.89603	1.20686	0.68297
C	1.06174	-3.8296	0.8878
C	-0.93351	-5.42419	-0.30052
C	-0.29164	-3.64814	1.20758
C	1.3984	-4.842	-0.0193
C	0.41031	-5.63098	-0.60947
C	-1.28084	-4.43237	0.61756
H	-0.56806	-2.8625	1.91779
H	2.4434	-5.02077	-0.28634
H	0.69623	-6.4129	-1.31928
H	-2.33019	-4.25848	0.87144
H	-1.7084	-6.03852	-0.76775
C	4.69394	0.15927	0.61041
C	5.93845	2.67726	0.74455
C	4.22201	1.14154	1.48957
C	5.79063	0.46248	-0.20468
C	6.40786	1.71123	-0.14454

C	4.84642	2.38521	1.56256
H	3.3417	0.94469	2.10976
H	6.16479	-0.29223	-0.90432
H	7.26038	1.92917	-0.79454
H	4.46852	3.13714	2.26037
H	6.42075	3.65739	0.79959
C	-3.26621	-1.47484	-0.71968
C	-5.25164	-2.17283	1.15945
C	-2.99281	-1.48285	0.64925
C	-4.5595	-1.8261	-1.1321
C	-5.54103	-2.17097	-0.20628
C	-3.97049	-1.82739	1.58247
H	-1.99594	-1.21969	1.00163
H	-4.80866	-1.83095	-2.19738
H	-6.54276	-2.43923	-0.55503
H	-3.71895	-1.82015	2.64739
H	-6.02257	-2.44284	1.88702
C	1.01013	1.0916	-2.81201
C	3.767	1.37447	-3.3205
C	1.8502	1.7493	-1.90023
C	1.57233	0.60684	-3.99788
C	2.9397	0.74594	-4.24942
C	3.21307	1.88471	-2.14491
H	1.43252	2.13399	-0.96687
H	0.94766	0.10447	-4.74109
H	3.35853	0.35553	-5.18184
H	3.84899	2.38398	-1.40769
H	4.83931	1.47487	-3.51197
O	0.56092	2.12465	1.264
C	-0.05697	3.15509	0.84439
C	-1.40062	3.28688	0.5742
O	0.68345	4.28323	0.63019
H	-1.77835	4.28261	0.33349
C	2.03467	4.24584	0.99491
H	2.44206	5.24267	0.76407
H	2.16208	4.03744	2.07329
H	2.60199	3.48326	0.434
C	-2.58643	2.30981	2.36888
H	-3.19521	3.17026	2.6745
C	-2.02147	1.57459	3.35806
C	-1.18989	0.46429	3.02094
N	-0.49656	-0.43177	2.7537
C	-2.14177	1.89982	4.82855
H	-1.16331	2.18052	5.25735
H	-2.83223	2.74629	4.97083
H	-2.52655	1.04098	5.40581
C	-2.37188	2.18329	0.88955
C	-3.66975	2.21996	0.09889
C	-4.77052	1.46801	0.52775
C	-3.78557	2.94334	-1.09267
C	-5.94421	1.42017	-0.22011
H	-4.69932	0.89193	1.45511
C	-4.962	2.90272	-1.84318
H	-2.93521	3.53293	-1.44419
C	-6.04444	2.13722	-1.41316
H	-6.78328	0.81209	0.13016
H	-5.03	3.47478	-2.77362
H	-6.96555	2.10268	-2.00256

C$^{R\text{-}E}$

C	-0.84494	-3.55917	-0.91668
C	1.47967	-2.71044	-2.07957
C	1.07841	-4.159	-2.36989
C	-0.44839	-4.18906	-2.27122
H	-0.64297	-4.3042	-0.12769
H	1.11074	-2.08787	-2.91449
H	1.44012	-4.4881	-3.35945
H	1.50707	-4.84716	-1.619
H	-0.88681	-3.59547	-3.09361
H	-0.85875	-5.20948	-2.35633
C	1.26109	-2.78919	0.89083
C	2.29415	-1.81691	1.47743
H	0.44352	-2.95142	1.61664
H	1.72924	-3.77253	0.70452
H	2.48859	-2.06575	2.53617
H	3.25374	-1.91678	0.93883
C	0.83004	0.39989	2.81556
C	3.39403	0.8102	1.96027
C	1.8558	0.70787	3.90901
H	0.36518	1.35635	2.52541
C	2.92444	1.56695	3.22452
H	4.05609	-0.011	2.28609
H	2.31759	-0.21608	4.30398
H	1.38794	1.23076	4.76099
H	3.7817	1.78259	3.88455
H	2.48297	2.53341	2.92485
P	1.84721	-0.02215	1.30575
P	0.39786	-2.17884	-0.63754
Cu	0.61493	0.12944	-0.58122
H	-3.45759	2.97912	-2.22466
C	-0.26528	-0.60853	3.03139
C	-2.27354	-2.57471	3.15524
C	-1.38707	-0.55136	2.18842
C	-0.18209	-1.6514	3.95809
C	-1.18038	-2.62808	4.01866
C	-2.38007	-1.52395	2.2405
H	-1.47007	0.26935	1.46709

H	0.67212	-1.71585	4.63826
H	-1.09781	-3.43951	4.74812
H	-3.23569	-1.46348	1.56076
H	-3.04875	-3.34521	3.19473
C	4.14561	1.64778	0.95307
C	5.5825	3.1772	-0.91723
C	3.47672	2.51408	0.07826
C	5.5411	1.57168	0.88632
C	6.25802	2.32981	-0.0394
C	4.19088	3.26771	-0.85142
H	2.3851	2.58974	0.11307
H	6.07618	0.89577	1.56199
H	7.34865	2.25207	-0.07932
H	3.65013	3.92968	-1.53401
H	6.13941	3.76735	-1.65089
C	2.92246	-2.34971	-1.83814
C	5.51868	-1.50941	-1.1542
C	3.30975	-1.00809	-1.98801
C	3.86817	-3.26759	-1.37101
C	5.15764	-2.85	-1.03166
C	4.5906	-0.58797	-1.64385
H	2.59024	-0.27581	-2.37331
H	3.60283	-4.3222	-1.25662
H	5.8829	-3.58228	-0.6644
H	4.86275	0.4659	-1.75032
H	6.52363	-1.17854	-0.87704
O	0.19438	1.32088	-2.04301
C	-0.88816	1.99936	-1.99975
C	-2.00156	1.74731	-1.23852
H	-2.01646	0.83065	-0.64332
C	-3.00709	3.9569	-0.43623
H	-3.8349	4.67137	-0.53899
C	-1.99109	4.34185	0.35229
C	-0.82518	3.53327	0.61965
N	0.17831	3.08798	0.99757
C	-1.95679	5.67763	1.06242
H	-1.07697	6.26751	0.75072
H	-2.86443	6.25952	0.83683
H	-1.89179	5.54348	2.15648
C	-3.19368	2.6612	-1.19768
C	-4.41481	1.95734	-0.61963
C	-4.38583	1.46937	0.69337
C	-5.57635	1.76907	-1.37309
C	-5.47878	0.79556	1.23182
H	-3.48776	1.61939	1.30119
C	-6.67856	1.09943	-0.83629
H	-5.61591	2.14284	-2.40144
C	-6.63237	0.60879	0.46742
H	-5.42978	0.4138	2.25603
H	-7.57788	0.96013	-1.44394
H	-7.49371	0.0824	0.88943
O	-0.93873	3.11933	-2.77717
C	0.23433	3.48278	-3.45694
H	0.52392	2.72931	-4.21019
H	0.01199	4.43687	-3.95984
H	1.08137	3.61873	-2.76147
C	-2.29296	-3.14522	-0.81559
C	-2.75735	-1.96042	-1.4003
C	-3.20556	-3.96029	-0.13781
C	-4.09786	-1.5961	-1.29951
H	-2.06233	-1.28875	-1.91576
C	-4.54988	-3.60128	-0.03851
H	-2.85528	-4.88586	0.33156
C	-4.99886	-2.41517	-0.61754
H	-4.4354	-0.65412	-1.73882
H	-5.24733	-4.24922	0.50098
H	-6.04743	-2.11772	-0.53305

C$^{S\text{-}Z}$

C	2.00328	-1.81341	1.75006
C	0.62253	0.48045	2.32864
C	1.59831	-0.00081	3.40533
C	2.68916	-0.763	2.65165
H	1.67967	-2.65827	2.38281
H	1.16116	1.22772	1.71973
H	2.01208	0.84795	3.97635
H	1.10058	-0.67621	4.12518
H	3.25707	-0.05821	2.02082
H	3.41378	-1.24663	3.32831
C	-0.88024	-2.0585	1.90835
C	-2.31302	-1.69806	1.49096
H	-0.62947	-3.0748	1.55364
H	-0.80065	-2.0689	3.01009
H	-2.9868	-2.55567	1.66769
H	-2.68522	-0.86089	2.10907
C	-2.76648	-2.6178	-1.31159
C	-4.30551	-0.60857	-0.27574
C	-4.26543	-2.91116	-1.21025
H	-2.56908	-2.25717	-2.3368
C	-4.95462	-1.54734	-1.31885
H	-4.6903	-0.90321	0.71627
H	-4.51548	-3.37213	-0.23744
H	-4.59552	-3.60991	-1.99823
H	-6.04372	-1.60943	-1.15476
H	-4.80385	-1.13115	-2.33125
P	-2.494	-1.08957	-0.25675
P	0.43098	-0.97366	1.1639

Cu	-0.45324	-0.31061	-0.84747
H	3.73924	2.28005	-1.98265
C	-1.74158	-3.67424	-0.98924
C	0.3206	-5.42706	-0.22319
C	-0.42867	-3.4913	-1.45428
C	-2.00848	-4.76675	-0.15911
C	-0.98371	-5.63759	0.22091
C	0.59461	-4.35236	-1.07199
H	-0.19603	-2.64748	-2.11482
H	-3.02294	-4.94343	0.20917
H	-1.21069	-6.4856	0.87392
H	1.6125	-4.17708	-1.43134
H	1.12549	-6.10117	0.08343
C	-4.60384	0.86054	-0.46901
C	-5.22276	3.58481	-0.77717
C	-3.83685	1.67629	-1.30779
C	-5.68919	1.43015	0.20866
C	-6.00067	2.77998	0.0563
C	-4.13948	3.02914	-1.45723
H	-2.95759	1.27383	-1.82049
H	-6.29262	0.80744	0.8781
H	-6.84895	3.20741	0.59936
H	-3.49227	3.64907	-2.0812
H	-5.45461	4.64812	-0.88771
C	2.87934	-2.34732	0.64246
C	4.51004	-3.34251	-1.41905
C	3.16149	-1.56565	-0.48415
C	3.44007	-3.62525	0.73036
C	4.25144	-4.12163	-0.29121
C	3.96385	-2.06027	-1.50944
H	2.73844	-0.55753	-0.57735
H	3.22847	-4.24868	1.60575
H	4.67778	-5.12588	-0.20773
H	4.15487	-1.4315	-2.38432
H	5.13802	-3.73326	-2.22517
C	-0.7189	1.05402	2.70318
C	-3.33596	1.96066	3.20147
C	-1.40011	1.84349	1.76228
C	-1.36082	0.75252	3.90883
C	-2.6608	1.20068	4.15537
C	-2.69602	2.2903	2.00495
H	-0.91947	2.10765	0.81266
H	-0.85113	0.15239	4.66763
H	-3.1476	0.9505	5.10288
H	-3.20762	2.89472	1.25094
H	-4.35678	2.30575	3.3888
O	0.48275	0.14047	-2.47473
C	1.51134	0.89221	-2.35984
C	1.84843	1.65155	-1.26869
O	2.36984	0.92115	-3.42027
H	1.14046	1.66637	-0.43711
C	2.07337	0.08391	-4.50867
H	2.8732	0.25121	-5.24679
H	2.06108	-0.9805	-4.21326
H	1.09715	0.32987	-4.96007
C	2.72834	4.03203	-1.4225
H	3.60724	4.69086	-1.4311
C	1.54074	4.63074	-1.61399
C	0.27281	3.93481	-1.59564
N	-0.83057	3.57759	-1.57907
C	1.39754	6.11822	-1.85033
H	0.76776	6.58329	-1.07177
H	2.3832	6.60999	-1.84264
H	0.91558	6.3168	-2.82367
C	3.02862	2.56791	-1.18811
C	3.74816	2.39597	0.14643
C	4.88767	1.59021	0.25028
C	3.24793	2.99213	1.31113
C	5.5174	1.39325	1.47875
H	5.28301	1.10311	-0.64674
C	3.8719	2.79474	2.54344
H	2.35659	3.62588	1.24973
C	5.01364	1.99783	2.63123
H	6.40664	0.75861	1.53607
H	3.46586	3.27347	3.43995
H	5.50727	1.8448	3.59532

$^{L3}TS_{C\text{-}D}^{R\text{-}Z}$

C	-0.15945	3.39412	-1.55428
C	-1.75079	1.50706	-2.74293
C	-1.687	2.87172	-3.43636
C	-0.31088	3.44984	-3.09145
H	-0.80108	4.18765	-1.13164
H	-1.04029	0.83484	-3.25505
H	-1.84146	2.78018	-4.52551
H	-2.46986	3.54936	-3.05049
H	0.48188	2.8397	-3.56136
H	-0.18331	4.4828	-3.45788
C	-2.33014	2.35865	0.05222
C	-3.06742	1.22993	0.78882
H	-1.83318	3.02131	0.783
H	-3.04936	2.97809	-0.51248
H	-3.60914	1.63847	1.66008
H	-3.81754	0.76741	0.12168
C	-1.23436	0.2817	2.96685
C	-3.15515	-1.42073	1.99418
C	-2.19211	-0.33257	3.99489

H	-0.29573	-0.29761	2.98455
C	-2.62476	-1.68402	3.42006
H	-4.12999	-0.91041	2.08826
H	-3.0872	0.29979	4.13525
H	-1.70694	-0.4351	4.98092
H	-3.39312	-2.181	4.03657
H	-1.75438	-2.36316	3.36779
P	-1.9556	-0.16746	1.29246
P	-0.95918	1.77471	-1.06057
Cu	-0.27472	-0.24892	-0.24387
H	3.58925	-0.46952	-0.74646
C	-0.88315	1.74675	3.04461
C	-0.24899	4.48586	2.90652
C	0.32177	2.17778	2.46618
C	-1.75114	2.70879	3.574
C	-1.43789	4.06788	3.50347
C	0.63463	3.53327	2.3954
H	1.00688	1.43477	2.04409
H	-2.69272	2.40679	4.04143
H	-2.13213	4.80453	3.9192
H	1.57427	3.84482	1.92939
H	-0.00609	5.55086	2.84661
C	-3.34259	-2.6529	1.14035
C	-3.72546	-4.9432	-0.4486
C	-2.25543	-3.28597	0.5233
C	-4.62045	-3.19182	0.95518
C	-4.81379	-4.32902	0.17048
C	-2.44534	-4.41625	-0.26837
H	-1.25135	-2.86543	0.63137
H	-5.48218	-2.70556	1.42466
H	-5.82212	-4.73146	0.03512
H	-1.58419	-4.87917	-0.75893
H	-3.87472	-5.82809	-1.07413
C	-3.06085	0.77387	-2.60166
C	-5.42086	-0.64801	-2.02837
C	-3.02769	-0.60827	-2.35445
C	-4.29953	1.42358	-2.58825
C	-5.47096	0.71775	-2.30354
C	-4.19284	-1.31186	-2.06378
H	-2.06809	-1.13555	-2.36487
H	-4.36197	2.49651	-2.79069
H	-6.42972	1.24491	-2.29344
H	-4.1378	-2.3841	-1.85396
H	-6.33659	-1.19847	-1.79377
O	1.58643	-0.33416	0.85477
C	1.86308	-1.54676	0.92912
C	2.72644	-2.22874	0.05825
H	2.88152	-3.30123	0.20289
C	3.72551	-2.07758	-2.13894
H	4.61022	-2.14208	-2.78078
C	2.51103	-2.41872	-2.58513
C	1.42142	-2.10878	-1.64994
N	0.3257	-1.65703	-1.55685
C	2.14573	-2.88997	-3.96253
H	1.46618	-2.16928	-4.45005
H	3.04448	-3.00922	-4.58882
H	1.61621	-3.85792	-3.92078
C	3.82617	-1.55166	-0.71374
C	5.2387	-1.66731	-0.14951
C	5.52463	-2.36029	1.02879
C	6.29682	-1.03174	-0.81536
C	6.82934	-2.432	1.5211
H	4.7112	-2.84075	1.57834
C	7.60002	-1.09806	-0.32837
H	6.09438	-0.46351	-1.7302
C	7.87285	-1.80469	0.84419
H	7.02868	-2.98077	2.44655
H	8.40779	-0.59083	-0.86461
H	8.89466	-1.85905	1.23094
O	1.07274	-2.24567	1.79721
C	1.20218	-3.63426	1.94899
H	2.21728	-3.91735	2.28417
H	0.47519	-3.93149	2.72011
H	0.96567	-4.17095	1.01219
C	1.24875	3.61933	-1.04394
C	2.13237	2.56898	-0.76923
C	1.69101	4.93238	-0.83632
C	3.41535	2.82459	-0.28588
H	1.8087	1.5305	-0.87787
C	2.97558	5.19247	-0.36238
H	1.0094	5.76663	-1.0355
C	3.84301	4.13587	-0.081
H	4.07544	1.98464	-0.05001
H	3.29704	6.22574	-0.20082
H	4.84689	4.33471	0.30527

$^{L3}TS_{C-D}^{S-Z}$

C	-1.25798	2.0804	2.25712
C	0.04168	-0.26521	2.7975
C	-0.6887	0.3947	3.97072
C	-1.85172	1.17591	3.35828
H	-0.68878	2.88147	2.76074
H	-0.60175	-1.06568	2.39337
H	-1.03026	-0.35811	4.70177
H	-0.02789	1.09771	4.50921
H	-2.58114	0.47669	2.90977
H	-2.39674	1.78056	4.10314

C	1.55399	2.03247	1.63926
C	2.79867	1.47751	0.93572
H	1.29038	3.0189	1.21807
H	1.76354	2.18213	2.71339
H	3.5367	2.28529	0.78385
H	3.28101	0.71076	1.56942
C	2.39798	1.95708	-1.9887
C	4.23547	0.11239	-1.13708
C	3.83223	2.11788	-2.50816
H	1.79471	1.48981	-2.78604
C	4.44871	0.72112	-2.53611
H	4.88418	0.66608	-0.43636
H	4.42937	2.75422	-1.83188
H	3.84157	2.60141	-3.50005
H	5.52219	0.73821	-2.79013
H	3.94518	0.10119	-3.29998
P	2.47441	0.60286	-0.67248
P	0.02845	1.00443	1.41028
Cu	0.32208	-0.23121	-0.53443
H	-3.15832	-1.07241	0.02121
C	1.66387	3.18994	-1.5195
C	0.20276	5.36356	-0.48208
C	0.2622	3.21257	-1.58176
C	2.31946	4.28866	-0.95073
C	1.59581	5.36482	-0.43356
C	-0.46158	4.28519	-1.06796
H	-0.26635	2.36307	-2.02574
H	3.41092	4.3138	-0.89406
H	2.12952	6.21011	0.01118
H	-1.55395	4.27509	-1.11924
H	-0.36701	6.20238	-0.07201
C	4.5734	-1.35119	-1.02316
C	5.26692	-4.07168	-0.83519
C	3.88609	-2.31763	-1.76713
C	5.61391	-1.77109	-0.18712
C	5.96	-3.1184	-0.09058
C	4.2303	-3.66483	-1.67652
H	3.06188	-2.01374	-2.42118
H	6.15903	-1.02922	0.40537
H	6.77507	-3.42436	0.57192
H	3.68221	-4.40428	-2.26717
H	5.53475	-5.12965	-0.7637
C	-2.28896	2.73019	1.36754
C	-4.26986	3.96685	-0.20267
C	-2.89469	2.04235	0.30946
C	-2.69138	4.0461	1.62309
C	-3.67114	4.66353	0.84639
C	-3.87836	2.65343	-0.4661
H	-2.57336	1.02406	0.07008
H	-2.22192	4.60114	2.44212
H	-3.96614	5.69486	1.06123
H	-4.33588	2.09607	-1.2874
H	-5.03823	4.44618	-0.81596
C	1.42041	-0.83986	3.00171
C	4.06069	-1.80785	3.10487
C	1.85519	-1.84288	2.12135
C	2.32067	-0.35459	3.9561
C	3.63159	-0.83566	4.00713
C	3.16235	-2.31678	2.16438
H	1.14623	-2.23141	1.38401
H	2.01099	0.41538	4.66838
H	4.32232	-0.44176	4.75882
H	3.48548	-3.08341	1.45352
H	5.0904	-2.17601	3.13684
O	-0.68645	-1.97294	0.09898
C	-1.21027	-2.6492	-0.82676
C	-2.43372	-2.41065	-1.4447
O	-0.47778	-3.63514	-1.40745
H	-2.6873	-3.08329	-2.27056
C	0.79523	-3.88903	-0.87367
H	1.25396	-4.64956	-1.52332
H	1.42784	-2.98569	-0.8636
H	0.73273	-4.2825	0.1573
C	-4.07117	-0.65856	-1.86088
H	-5.12939	-0.37829	-1.90292
C	-3.18534	-0.10769	-2.7
C	-1.78702	-0.43549	-2.43083
N	-0.6692	-0.06697	-2.3256
C	-3.45553	0.94326	-3.73942
H	-3.08837	0.62334	-4.73009
H	-4.53587	1.14683	-3.81779
H	-2.93538	1.88408	-3.48478
C	-3.57253	-1.656	-0.82603
C	-4.71603	-2.48332	-0.24185
C	-5.78536	-1.83002	0.38738
C	-4.72644	-3.87895	-0.28435
C	-6.84107	-2.54764	0.94483
H	-5.78581	-0.73565	0.45318
C	-5.78355	-4.60315	0.27078
H	-3.8878	-4.40477	-0.74768
C	-6.84581	-3.94236	0.88431
H	-7.66287	-2.01596	1.43425
H	-5.77211	-5.69645	0.22527
H	-7.67258	-4.51067	1.3206

$^{L3}TS_{C\text{-}D}^{R\text{-}E}$

C	-0.97905	-3.30394	-1.33518

C	1.05992	-1.97935	-2.5895
C	0.65198	-3.31542	-3.21521
C	-0.82218	-3.50726	-2.85999
H	-0.65037	-4.22657	-0.82649
H	0.50991	-1.17402	-3.10921
H	0.82163	-3.31833	-4.30606
H	1.24209	-4.14778	-2.79109
H	-1.43028	-2.75372	-3.39161
H	-1.20519	-4.49781	-3.15862
C	1.44586	-2.94547	0.19677
C	2.58202	-2.12078	0.82249
H	0.80136	-3.36091	0.9925
H	1.86739	-3.8028	-0.35834
H	2.98114	-2.6407	1.71198
H	3.41416	-2.02271	0.10264
C	1.41988	-0.42839	2.97208
C	3.77301	0.33201	1.80566
C	2.63954	-0.41094	3.89604
H	0.91575	0.54959	3.05481
C	3.57774	0.64719	3.30813
H	4.5005	-0.49373	1.72087
H	3.14935	-1.39182	3.90759
H	2.35448	-0.17859	4.93681
H	4.55172	0.6841	3.82476
H	3.11606	1.64514	3.41185
P	2.12689	-0.37208	1.24311
P	0.27733	-1.97705	-0.87812
Cu	0.56974	0.27691	-0.23813
H	-3.33861	3.57774	-1.5984
C	0.37416	-1.50497	3.09371
C	-1.57743	-3.52974	3.0221
C	-0.88333	-1.27596	2.51151
C	0.62438	-2.7548	3.66775
C	-0.34569	-3.76042	3.6322
C	-1.84818	-2.27664	2.46718
H	-1.09844	-0.29601	2.07182
H	1.58845	-2.95955	4.14192
H	-0.13113	-4.73378	4.08366
H	-2.81259	-2.08142	1.98916
H	-2.33253	-4.32006	2.98229
C	4.27328	1.4933	0.97962
C	5.23055	3.63924	-0.56465
C	3.41864	2.53806	0.60279
C	5.61343	1.54874	0.58334
C	6.09255	2.6124	-0.18163
C	3.89299	3.59863	-0.16638
H	2.36301	2.51424	0.89411
H	6.29198	0.73713	0.86652
H	7.1432	2.63399	-0.48612
H	3.20702	4.3981	-0.46128
H	5.59976	4.47068	-1.17206
C	2.51542	-1.58886	-2.54793
C	5.18621	-0.77466	-2.19965
C	2.8432	-0.22865	-2.42429
C	3.55174	-2.52848	-2.53288
C	4.87793	-2.1244	-2.3608
C	4.16278	0.17436	-2.24156
H	2.04652	0.52328	-2.46217
H	3.33231	-3.5939	-2.64431
H	5.67403	-2.87499	-2.34864
H	4.3924	1.23726	-2.12256
H	6.22231	-0.45705	-2.05069
O	0.01992	1.4767	-1.67554
C	-1.01971	2.21299	-1.5881
C	-2.15911	1.95706	-0.86639
H	-2.22601	1.00578	-0.33246
C	-3.00691	3.95118	0.43624
H	-3.74083	4.76236	0.53311
C	-2.00086	3.93896	1.32531
C	-0.97647	2.91802	1.34402
N	-0.07072	2.23814	1.61065
C	-1.83258	4.97505	2.41398
H	-0.86389	5.4959	2.31639
H	-2.63785	5.72478	2.36273
H	-1.85434	4.50671	3.4136
C	-3.25691	2.96418	-0.68279
C	-4.60485	2.28689	-0.47236
C	-4.96021	1.76975	0.77898
C	-5.49206	2.11246	-1.54042
C	-6.16908	1.09771	0.96028
H	-4.27477	1.88921	1.62421
C	-6.70174	1.44118	-1.36479
H	-5.22232	2.50036	-2.52787
C	-7.04614	0.93204	-0.11141
H	-6.42497	0.69754	1.94597
H	-7.38057	1.31372	-2.2133
H	-7.99574	0.40695	0.028
O	-0.98244	3.40398	-2.25566
C	0.20869	3.72676	-2.92437
H	0.42274	3.02166	-3.74703
H	0.06177	4.7365	-3.33849
H	1.07516	3.72865	-2.23959
C	-2.38959	-2.98889	-0.90164
C	-2.95423	-1.73396	-1.16285
C	-3.16601	-3.94884	-0.24571
C	-4.25632	-1.44143	-0.76669
H	-2.3658	-0.9577	-1.66487
C	-4.47195	-3.66055	0.15421

H	-2.73735	-4.93363	-0.03144
C	-5.01862	-2.40347	-0.10112
H	-4.67289	-0.45272	-0.97315
H	-5.062	-4.42245	0.67269
H	-6.03846	-2.16583	0.21499

$^{L3}TS_{C\text{-}D}^{S\text{-}E}$

C	-1.97014	1.49648	1.82161
C	-0.39835	-0.7008	2.3115
C	-1.5501	-0.43791	3.29242
C	-2.64122	0.28927	2.50437
H	-1.66936	2.20431	2.61487
H	-0.70596	-1.53514	1.65712
H	-1.91786	-1.38177	3.72975
H	-1.21986	0.20494	4.12762
H	-3.07154	-0.38219	1.7399
H	-3.47516	0.61864	3.14638
C	0.88415	1.9828	1.79562
C	2.3174	1.70604	1.32366
H	0.5416	2.96399	1.41838
H	0.85293	2.027	2.89836
H	2.93453	2.61609	1.42851
H	2.77687	0.92682	1.9579
C	2.60903	2.53818	-1.52914
C	4.28788	0.6469	-0.48227
C	4.10692	2.85695	-1.57823
H	2.32015	2.14743	-2.52048
C	4.81971	1.50377	-1.65151
H	4.70963	1.06967	0.44684
H	4.43076	3.38526	-0.66356
H	4.35129	3.50755	-2.43552
H	5.91709	1.60089	-1.59123
H	4.59052	1.00508	-2.6108
P	2.45645	1.03761	-0.40641
P	-0.37412	0.78364	1.14989
Cu	0.44166	0.09702	-0.89335
H	-3.86469	-2.48201	-2.20507
C	1.61127	3.59976	-1.14154
C	-0.37391	5.38042	-0.24497
C	0.26486	3.41616	-1.49658
C	1.9474	4.7037	-0.35161
C	0.96171	5.58749	0.0943
C	-0.71878	4.29349	-1.05045
H	-0.02469	2.55861	-2.11539
H	2.98814	4.88202	-0.06744
H	1.24473	6.44318	0.71463
H	-1.76275	4.12148	-1.32568
H	-1.1493	6.06432	0.11169
C	4.66603	-0.8159	-0.53289
C	5.43308	-3.52109	-0.56665
C	3.85859	-1.77853	-1.14613
C	5.86918	-1.22805	0.05507
C	6.2544	-2.56715	0.03695
C	4.23437	-3.12124	-1.15594
H	2.89502	-1.49941	-1.57983
H	6.50839	-0.48795	0.54886
H	7.19501	-2.86934	0.50702
H	3.56416	-3.85478	-1.61175
H	5.72494	-4.57537	-0.56951
C	-2.83573	2.23702	0.8319
C	-4.50393	3.61875	-0.96273
C	-3.18214	1.66987	-0.40048
C	-3.33758	3.50375	1.15033
C	-4.16615	4.19188	0.26304
C	-4.0073	2.35518	-1.28921
H	-2.79936	0.6832	-0.68358
H	-3.07223	3.96378	2.10827
H	-4.54707	5.18193	0.53121
H	-4.25764	1.89313	-2.24899
H	-5.15014	4.15653	-1.66277
C	0.98256	-1.01386	2.83352
C	3.65731	-1.4438	3.60357
C	1.85628	-1.75522	2.02286
C	1.46838	-0.508	4.04494
C	2.79516	-0.71855	4.42551
C	3.17955	-1.9689	2.40186
H	1.50441	-2.15346	1.06489
H	0.81381	0.06843	4.70461
H	3.15549	-0.3106	5.37473
H	3.8405	-2.54399	1.74696
H	4.6976	-1.60602	3.90027
O	-0.5751	-0.23391	-2.5237
C	-1.58853	-1.00839	-2.47327
C	-2.15915	-1.53635	-1.34047
O	-2.15452	-1.36579	-3.65966
H	-1.70753	-1.26923	-0.38476
C	-1.56635	-0.8619	-4.83135
H	-2.15465	-1.27161	-5.66708
H	-1.59874	0.24133	-4.86422
H	-0.51393	-1.17991	-4.92924
C	-2.68524	-3.9951	-1.34512
H	-3.42894	-4.80031	-1.41574
C	-1.39536	-4.3677	-1.29718
C	-0.30487	-3.42344	-1.16444
N	0.71672	-2.88654	-1.02352
C	-0.93882	-5.80788	-1.35363
H	-0.36921	-6.07809	-0.44721

H	-1.80225	-6.48643	-1.43939
H	-0.27542	-5.97616	-2.22008
C	-3.22648	-2.58349	-1.30807
C	-4.11202	-2.35767	-0.08703
C	-5.09787	-1.36277	-0.12734
C	-3.90122	-3.03401	1.11864
C	-5.85518	-1.05708	0.99953
H	-5.25421	-0.80696	-1.05722
C	-4.66206	-2.73361	2.25219
H	-3.12633	-3.80493	1.17787
C	-5.64185	-1.74542	2.19682
H	-6.61357	-0.27053	0.94588
H	-4.48178	-3.27647	3.18521
H	-6.23593	-1.50619	3.08367

$^{L3}TS_{B-D}^{R\text{-}anti}$

H	-2.8647	0.69124	-0.03801
O	-1.15815	2.17509	1.5008
C	-0.87922	2.54167	0.3678
C	-1.4651	2.07831	-0.88861
O	0.04962	3.4928	0.14318
C	-2.84381	1.45087	-0.84281
H	-1.4324	2.92012	-1.59452
C	0.70378	4.03988	1.26278
C	-3.9509	2.44703	-0.5523
H	1.17982	3.25341	1.86949
H	-0.00354	4.59697	1.90114
H	1.48012	4.71056	0.86708
C	-4.44627	2.59156	0.74858
C	-4.46242	3.27369	-1.55987
C	-5.43179	3.53593	1.0346
H	-4.03937	1.96433	1.54723
C	-5.44796	4.21822	-1.27732
H	-4.07873	3.17765	-2.58143
C	-5.9376	4.35161	0.02249
H	-5.80644	3.6359	2.05778
H	-5.83681	4.85472	-2.07796
H	-6.71177	5.09161	0.24601
C	-3.00994	0.75188	-2.17119
H	-4.00128	0.40986	-2.48805
C	-1.92246	0.55496	-2.92422
C	-0.62978	1.00298	-2.35096
N	0.54863	0.93498	-2.61055
C	-1.86312	-0.15716	-4.24164
H	-1.37905	0.47754	-5.00382
H	-2.86967	-0.43708	-4.59237
H	-1.24983	-1.07247	-4.16082
P	0.24231	-2.08547	-0.64089
P	1.7692	0.1812	1.18191
C	1.3737	-2.61468	0.72532
C	1.13939	-2.49177	-2.25501
C	-0.9142	-3.55813	-0.77026
C	3.34135	1.16102	1.51349
C	1.14019	0.3327	2.935
C	2.41769	-1.56096	1.11787
H	1.881	-3.55846	0.45989
H	0.70587	-2.83195	1.57748
C	0.60489	-3.86463	-2.68874
H	0.75725	-1.72877	-2.95505
C	2.63276	-2.29462	-2.14258
C	-0.86252	-3.93133	-2.26481
H	-0.43047	-4.3748	-0.20572
C	-2.30259	-3.3539	-0.21694
C	3.28096	1.53655	3.01783
H	4.16083	0.43333	1.36638
C	3.60219	2.34293	0.58956
C	2.41053	0.52716	3.76621
H	0.55174	1.26706	2.9323
C	0.20877	-0.8021	3.28538
H	3.23497	-1.54618	0.37699
H	2.86952	-1.83137	2.08833
H	1.15397	-4.67917	-2.1851
H	0.73375	-4.0116	-3.77468
C	3.15792	-1.00732	-2.33589
C	3.50557	-3.3174	-1.75134
H	-1.30502	-4.92805	-2.43165
H	-1.4632	-3.20877	-2.84792
C	-3.04878	-2.21846	-0.54908
C	-2.8848	-4.3126	0.61807
H	2.83649	2.54127	3.12011
H	4.29256	1.59134	3.45304
C	3.08231	2.40797	-0.70734
C	4.40194	3.4079	1.03081
H	2.17286	0.87405	4.78669
H	2.96056	-0.42439	3.87187
C	-1.07487	-0.81598	2.7159
C	0.59573	-1.8783	4.09014
C	4.50516	-0.74221	-2.09904
H	2.48609	-0.20452	-2.65775
C	4.85714	-3.05456	-1.52219
H	3.13662	-4.3367	-1.60691
C	-4.3375	-2.03617	-0.05392
H	-2.60544	-1.45379	-1.19266
C	-4.17807	-4.13957	1.11285
H	-2.31419	-5.20532	0.89463
C	3.32886	3.50721	-1.52857
H	2.43557	1.61845	-1.09496

C	4.65805	4.50484	0.20988
H	4.83556	3.39066	2.03451
C	-1.92895	-1.89585	2.91484
H	-1.39457	0.04214	2.11406
C	-0.26387	-2.96105	4.29354
H	1.58017	-1.88935	4.5651
C	5.36039	-1.76387	-1.68218
H	4.88508	0.27431	-2.23838
H	5.52039	-3.8675	-1.21184
C	-4.90821	-2.99854	0.78145
H	-4.89571	-1.1316	-0.3149
H	-4.61494	-4.8993	1.7677
C	4.1161	4.56288	-1.07462
H	2.87929	3.52999	-2.52495
H	5.28271	5.323	0.58086
C	-1.52256	-2.9792	3.69719
H	-2.91926	-1.89628	2.45101
H	0.05847	-3.79568	4.92339
H	6.41794	-1.55742	-1.49369
H	-5.91859	-2.8569	1.1754
H	4.30612	5.42889	-1.71535
H	-2.19406	-3.82985	3.84532
Cu	0.23933	0.27099	-0.52688

$^{L3}TS_{B-D}^{R-syn}$

H	2.93514	0.17062	-0.73622
O	1.94782	-3.64094	-1.40865
C	1.30739	-2.83893	-0.76831
C	1.54172	-1.40513	-0.66621
O	0.18769	-3.22568	-0.07724
C	2.91887	-0.90089	-1.02309
H	1.15474	-0.99868	0.28103
C	-0.1878	-4.57668	-0.24403
C	4.10933	-1.51283	-0.28789
H	0.59646	-5.25661	0.12889
H	-0.37155	-4.80669	-1.30656
H	-1.11572	-4.71606	0.33019
C	5.41854	-1.21427	-0.68848
C	3.94393	-2.29713	0.85813
C	6.52055	-1.68697	0.02161
H	5.58762	-0.58986	-1.57037
C	5.04199	-2.77209	1.57553
H	2.93672	-2.54923	1.19983
C	6.33753	-2.47098	1.16061
H	7.53109	-1.43949	-0.31787
H	4.87956	-3.3877	2.46537
H	7.19992	-2.84526	1.71994
C	3.01809	-0.92414	-2.53182
H	3.98959	-0.91096	-3.03358
C	1.88509	-0.91682	-3.23811
C	0.62571	-0.89247	-2.47607
N	-0.55472	-0.7016	-2.61947
C	1.76155	-0.87076	-4.73259
H	1.19338	-1.7426	-5.10115
H	2.75377	-0.86978	-5.21249
H	1.20885	0.03042	-5.0517
P	-1.59721	-0.48986	1.23663
P	-0.46697	2.19485	-0.20271
C	-2.20232	1.16611	1.80939
C	-0.56684	-1.18987	2.6509
C	-3.06481	-1.59801	1.5835
C	0.78318	3.59821	-0.17424
C	-1.66072	3.12546	-1.30483
C	-1.23337	2.31368	1.48942
H	-2.42868	1.15306	2.8897
H	-3.15965	1.31659	1.27962
C	-1.55519	-2.00019	3.50154
H	0.09486	-1.90929	2.13816
C	0.28762	-0.1445	3.32438
C	-2.50542	-2.688	2.52124
H	-3.77676	-0.98825	2.16768
C	-3.78235	-2.12235	0.36088
C	0.17204	4.72658	-1.04531
H	0.83404	3.93982	0.87305
C	2.16213	3.14813	-0.59879
C	-1.35366	4.60055	-1.03442
H	-1.32305	2.88257	-2.32911
C	-3.06551	2.6035	-1.13553
H	-0.38289	2.29322	2.19481
H	-1.74206	3.28468	1.62379
H	-2.14053	-1.34052	4.16652
H	-1.02357	-2.72214	4.14494
C	1.55829	0.14854	2.80719
C	-0.16658	0.612	4.41288
H	-3.32617	-3.21992	3.03151
H	-1.95093	-3.43282	1.92491
C	-3.0865	-2.53101	-0.78338
C	-5.17838	-2.22386	0.36829
H	0.53262	4.62436	-2.08287
H	0.51079	5.71427	-0.69074
C	3.22132	3.13424	0.31519
C	2.40962	2.71669	-1.90899
H	-1.82419	5.25798	-1.78603
H	-1.74455	4.90765	-0.04817
C	-3.37519	1.33534	-1.65396
C	-4.04027	3.28277	-0.39792
C	2.33409	1.17888	3.33261

H	1.95871	-0.44299	1.97975
C	0.60531	1.64759	4.9404
H	-1.14185	0.40292	4.86042
C	-3.77281	-3.0123	-1.89676
H	-1.99717	-2.44281	-0.82315
C	-5.8674	-2.71465	-0.74029
H	-5.73726	-1.9016	1.25354
C	4.49601	2.71706	-0.06904
H	3.04378	3.45714	1.34602
C	3.68498	2.3196	-2.30242
H	1.59268	2.67463	-2.63723
C	-4.61305	0.74915	-1.40779
H	-2.62919	0.78651	-2.24003
C	-5.28482	2.69615	-0.15443
H	-3.83462	4.27835	0.00466
C	1.85543	1.943	4.39717
H	3.32232	1.37325	2.90672
H	0.22509	2.22632	5.78734
C	-5.16495	-3.10656	-1.88031
H	-3.20824	-3.30048	-2.7877
H	-6.95915	-2.7822	-0.71653
C	4.73468	2.32028	-1.38364
H	5.30915	2.70692	0.66241
H	3.8525	1.98734	-3.33036
C	-5.57031	1.42419	-0.64727
H	-4.8262	-0.24706	-1.80534
H	-6.03389	3.24084	0.42815
H	2.46047	2.75347	4.81371
H	-5.70261	-3.48012	-2.75666
H	5.73585	2.0036	-1.68831
H	-6.53978	0.95828	-0.44836
Cu	-0.39235	-0.04174	-0.65232

$^{L3}TS_{B-D}^{S-syn}$

H	-2.72644	-0.30907	0.66352
O	-2.73232	-2.24695	-2.87848
C	-1.77099	-2.00235	-2.18225
C	-1.72055	-1.14564	-1.00309
O	-0.52914	-2.44998	-2.5211
C	-3.01548	-0.8356	-0.26881
H	-0.886	-1.45227	-0.34003
C	-0.42546	-3.09954	-3.76906
C	-3.83146	-2.04199	0.16019
H	-1.02754	-4.02351	-3.78914
H	-0.76717	-2.44175	-4.58553
H	0.64048	-3.33904	-3.90128
C	-3.62565	-2.60144	1.42706
C	-4.77961	-2.63643	-0.68121
C	-4.33626	-3.72462	1.84738
H	-2.89317	-2.1456	2.10347
C	-5.49619	-3.75751	-0.26367
H	-4.93334	-2.22818	-1.68222
C	-5.27812	-4.30736	0.99974
H	-4.15721	-4.14266	2.84264
H	-6.2301	-4.20993	-0.93749
H	-5.84144	-5.18763	1.32356
C	-3.76937	0.16554	-1.11524
H	-4.8421	0.31895	-0.96375
C	-3.07446	0.8515	-2.02401
C	-1.61551	0.61344	-2.03526
N	-0.60509	1.14105	-2.43768
C	-3.60754	1.84626	-3.00847
H	-3.56649	1.42005	-4.02734
H	-4.65235	2.11226	-2.78087
H	-2.99439	2.7614	-3.01332
P	2.37225	0.38712	-0.50948
P	-0.08224	0.99906	1.55029
C	2.7434	1.0024	1.20412
C	2.94735	1.72068	-1.70727
C	3.84151	-0.72663	-0.85727
C	-1.30408	2.14981	2.40687
C	-0.26009	-0.33877	2.85816
C	1.58277	1.76518	1.8673
H	3.65067	1.63069	1.20355
H	2.98059	0.09484	1.78583
C	4.38422	1.34192	-2.09083
H	2.30059	1.54708	-2.58401
C	2.68075	3.12059	-1.21328
C	4.42118	-0.18247	-2.17952
H	4.57612	-0.51235	-0.06019
C	3.58373	-2.21548	-0.87261
C	-1.91632	1.31235	3.54395
H	-0.69578	2.9542	2.86064
C	-2.28234	2.79046	1.44296
C	-0.8282	0.38721	4.08135
H	-1.05818	-0.98724	2.45492
C	0.97325	-1.185	3.02825
H	1.51858	2.78635	1.44667
H	1.77306	1.87132	2.95028
H	5.1025	1.67245	-1.31981
H	4.67878	1.82494	-3.03815
C	1.4417	3.71719	-1.49181
C	3.60552	3.83477	-0.44038
H	5.4401	-0.5726	-2.34369
H	3.7994	-0.53093	-3.0243
C	2.37719	-2.7591	-1.32529
C	4.59903	-3.08895	-0.46342

H	-2.7404	0.69157	3.14962
H	-2.3436	1.95911	4.32856
C	-1.78862	3.45827	0.31482
C	-3.66568	2.76218	1.64022
H	-1.21799	-0.32807	4.82583
H	-0.04762	0.98785	4.5812
C	1.21326	-2.22774	2.12286
C	1.92508	-0.93492	4.02358
C	1.1409	4.98845	-1.00473
H	0.704	3.16362	-2.08542
C	3.299	5.10211	0.05579
H	4.5866	3.4063	-0.21714
C	2.18361	-4.14011	-1.3465
H	1.55599	-2.11126	-1.64583
C	4.41533	-4.47054	-0.49888
H	5.54867	-2.67893	-0.10285
C	-2.64367	4.09045	-0.57944
H	-0.71127	3.4781	0.12736
C	-4.52945	3.38652	0.73707
H	-4.0932	2.25535	2.5085
C	2.37879	-2.98494	2.19361
H	0.47651	-2.44964	1.34324
C	3.09777	-1.68854	4.09346
H	1.76303	-0.13848	4.75452
C	2.06302	5.68377	-0.22207
H	0.17268	5.44169	-1.2368
H	4.03658	5.64035	0.65845
C	3.20127	-5.00195	-0.93638
H	1.21947	-4.53576	-1.67808
H	5.22116	-5.13516	-0.17352
C	-4.02334	4.05692	-0.37217
H	-2.22988	4.5997	-1.45476
H	-5.60888	3.34735	0.91002
C	3.33199	-2.71069	3.17498
H	2.54411	-3.7902	1.47373
H	3.83172	-1.47408	4.87591
H	1.82241	6.67948	0.16123
H	3.04787	-6.08484	-0.95312
H	-4.69982	4.5432	-1.08057
H	4.25105	-3.30127	3.22603
Cu	0.09103	0.33377	-0.63851

D$^{R\text{-}anti}$

C	4.21567	0.80907	-0.7369
C	2.34334	2.57435	-1.67658
C	3.81195	2.96201	-1.88231
C	4.58692	1.64451	-1.98144
H	4.69749	1.29006	0.13277
H	1.97152	2.14669	-2.62344
H	3.93962	3.5915	-2.77978
H	4.19543	3.53821	-1.02125
H	4.29153	1.09882	-2.89615
H	5.67815	1.79965	-2.03429
C	2.32413	1.78984	1.20506
C	0.90596	1.97054	1.76112
H	2.87857	1.05832	1.81993
H	2.87774	2.74428	1.2528
H	0.93552	2.03675	2.8628
H	0.46728	2.91364	1.3871
C	-0.11952	-0.7843	2.41575
C	-1.89342	1.25534	1.95788
C	-1.23287	-0.58686	3.454
H	-0.3881	-1.65858	1.79964
C	-2.425	0.0174	2.70876
H	-1.61734	2.00725	2.71914
H	-0.91593	0.11383	4.24664
H	-1.49005	-1.54108	3.945
H	-3.24904	0.29605	3.38717
H	-2.84154	-0.71234	1.9893
P	-0.28329	0.65734	1.20911
P	2.37825	1.09293	-0.51794
C	1.30699	-0.97203	2.87083
C	4.04367	-1.17275	3.50903
C	2.19259	-1.67556	2.03897
C	1.81486	-0.38842	4.03743
C	3.1716	-0.48539	4.35256
C	3.54591	-1.77515	2.35249
H	1.81576	-2.12878	1.11745
H	1.15372	0.16161	4.71288
H	3.54789	-0.01729	5.26719
H	4.21545	-2.32044	1.68074
H	5.10728	-1.24474	3.75429
C	-2.88003	1.89181	1.00716
C	-4.7923	3.06266	-0.69648
C	-3.07047	1.41407	-0.29296
C	-3.65881	2.97316	1.43705
C	-4.60658	3.55577	0.59586
C	-4.01694	1.99022	-1.13749
H	-2.44263	0.60979	-0.67753
H	-3.51761	3.36998	2.44818
H	-5.20162	4.40219	0.95163
H	-4.13443	1.59803	-2.15216
H	-5.53227	3.51997	-1.35971
C	4.66872	-0.63339	-0.77224
C	5.59519	-3.29154	-0.79684
C	3.87876	-1.65998	-1.30337
C	5.93186	-0.96092	-0.26288

C	6.39563	-2.27517	-0.27471
C	4.33736	-2.97676	-1.31051
H	2.87542	-1.44097	-1.68033
H	6.5592	-0.17089	0.16384
H	7.38335	-2.50779	0.13409
H	3.69674	-3.76535	-1.71498
H	5.94998	-4.32623	-0.79988
C	1.3407	3.59883	-1.20694
C	-0.62226	5.30777	-0.13718
C	-0.02417	3.36009	-1.43983
C	1.70328	4.72008	-0.45267
C	0.7297	5.56789	0.07971
C	-0.99547	4.20438	-0.90675
H	-0.33028	2.48579	-2.02741
H	2.7576	4.9404	-0.26366
H	1.03457	6.43698	0.67046
H	-2.05329	3.99305	-1.08625
H	-1.38729	5.96503	0.286
Cu	0.45636	-0.03426	-0.88448
N	-0.75311	-0.19719	-2.43073
C	-2.84872	-1.30925	-3.17174
C	-3.66104	-2.22463	-2.62728
H	-4.61139	-2.55125	-3.06182
C	-1.65379	-1.04961	-2.31174
C	-3.02296	-0.54825	-4.4438
H	-2.95266	0.53567	-4.24674
H	-2.20075	-0.78305	-5.14255
H	-3.9852	-0.7715	-4.93339
C	-3.16663	-2.79778	-1.31491
C	-1.82721	-2.0779	-1.08631
C	-0.57121	-2.85601	-1.03003
O	0.47887	-2.37011	-0.63615
H	-1.85595	-1.45387	-0.17998
O	-0.62964	-4.08597	-1.53326
C	0.59335	-4.79278	-1.63774
H	1.27487	-4.27874	-2.33533
H	1.08461	-4.87884	-0.65495
H	0.33583	-5.78782	-2.02538
H	-3.00854	-3.88451	-1.41846
C	-4.08184	-2.55856	-0.12499
C	-4.92562	-1.44537	-0.06707
C	-4.00075	-3.39244	0.99755
C	-5.66111	-1.16271	1.08441
H	-4.99296	-0.77758	-0.92995
C	-4.73605	-3.11673	2.14939
H	-3.34168	-4.26717	0.96845
C	-5.56884	-1.99689	2.19727
H	-6.30121	-0.27595	1.11077
H	-4.65876	-3.78027	3.01609
H	-6.14288	-1.77599	3.10193

D$^{S\text{-}anti}$

C	-0.84923	2.97599	1.81126
C	0.9402	1.25734	2.96924
C	0.55462	2.47547	3.81228
C	-0.85214	2.8661	3.35585
H	-0.4307	3.95791	1.53122
H	0.27446	0.42128	3.24849
H	0.59273	2.24955	4.89226
H	1.24662	3.31771	3.63092
H	-1.56722	2.08115	3.66066
H	-1.19885	3.80961	3.81048
C	1.76379	2.72998	0.53816
C	2.85899	1.91313	-0.16727
H	1.27599	3.40806	-0.18453
H	2.21205	3.36158	1.32573
H	3.43776	2.56144	-0.84902
H	3.56624	1.50575	0.5775
C	1.75457	1.01055	-2.78743
C	3.78558	-0.42391	-1.63349
C	3.03423	0.85923	-3.61622
H	1.03252	0.24629	-3.12196
C	3.66803	-0.4615	-3.17341
H	4.6173	0.2555	-1.37691
H	3.74109	1.68296	-3.40996
H	2.81854	0.87787	-4.69873
H	4.65496	-0.63418	-3.63538
H	3.01963	-1.30604	-3.47104
P	2.21217	0.42204	-1.06483
P	0.3859	1.68467	1.22603
C	1.03631	2.33521	-2.7421
C	-0.33988	4.76547	-2.3898
C	-0.31722	2.3491	-2.36896
C	1.6801	3.55839	-2.95852
C	0.99829	4.76441	-2.78069
C	-0.99877	3.54991	-2.19347
H	-0.83262	1.39952	-2.19618
H	2.73045	3.58376	-3.26157
H	1.52124	5.71043	-2.95035
H	-2.04989	3.53274	-1.89306
H	-0.87228	5.71059	-2.24771
C	4.05335	-1.76556	-0.98998
C	4.6004	-4.25313	0.2054
C	3.02644	-2.68933	-0.75679
C	5.35821	-2.11381	-0.62314
C	5.6338	-3.34708	-0.03311
C	3.29543	-3.91857	-0.15869
H	1.99125	-2.42905	-0.99748

H	6.17205	-1.40018	-0.79042
H	6.66035	-3.59671	0.25127
H	2.47126	-4.60974	0.03858
H	4.81054	-5.21569	0.68063
C	-2.2195	2.84052	1.18839
C	-4.78109	2.60273	0.04987
C	-2.8477	1.59143	1.08795
C	-2.90293	3.96773	0.72199
C	-4.17429	3.8532	0.15691
C	-4.11446	1.47034	0.52101
H	-2.32842	0.69422	1.44353
H	-2.42639	4.95152	0.79012
H	-4.68957	4.74765	-0.20611
H	-4.58617	0.48665	0.44254
H	-5.77408	2.50351	-0.39771
C	2.35828	0.74472	2.98689
C	4.9871	-0.21891	2.72337
C	2.59989	-0.57282	2.563
C	3.45098	1.55614	3.31074
C	4.75706	1.07815	3.17931
C	3.902	-1.04608	2.42574
H	1.75559	-1.22969	2.31818
H	3.29364	2.58014	3.66106
H	5.59909	1.7292	3.43335
H	4.0687	-2.06896	2.07586
H	6.00922	-0.59169	2.60854
Cu	0.40724	-0.31057	0.09032
N	-0.22152	-1.89978	1.0075
C	-1.9217	-3.67137	1.31746
C	-3.09228	-3.96333	0.7376
H	-3.73057	-4.80982	1.01132
C	-1.2978	-2.454	0.71699
C	-1.2222	-4.39267	2.42089
H	-0.21956	-4.71272	2.08693
H	-1.05627	-3.71146	3.27356
H	-1.78859	-5.27358	2.76527
C	-3.49288	-3.01178	-0.36973
H	-3.54766	-3.56222	-1.32435
C	-2.33886	-1.9907	-0.41588
H	-2.66886	-0.97981	-0.1381
C	-4.82545	-2.31532	-0.15489
C	-5.52587	-1.79689	-1.25126
C	-5.3406	-2.0966	1.12715
C	-6.70372	-1.0734	-1.07384
H	-5.13278	-1.95715	-2.26106
C	-6.52287	-1.37737	1.30981
H	-4.80052	-2.48726	1.9949
C	-7.20763	-0.86052	0.21078
H	-7.23343	-0.67428	-1.94408
H	-6.90763	-1.2142	2.32086
H	-8.13244	-0.29407	0.35365
C	-1.55082	-1.85573	-1.66084
O	-0.82848	-0.89746	-1.8834
O	-1.57443	-2.91583	-2.46921
C	-0.69116	-2.89379	-3.57505
H	0.35579	-2.83131	-3.23395
H	-0.8562	-3.83678	-4.11399
H	-0.90508	-2.03591	-4.23341

D$^{R\text{-}syn}$

C	3.84609	0.95573	-1.14901
C	1.75493	2.40482	-2.16303
C	3.15609	2.83072	-2.61322
C	4.01481	1.56395	-2.55934
H	4.41415	1.58984	-0.4458
H	1.32986	1.75645	-2.94867
H	3.13704	3.28005	-3.62129
H	3.58245	3.58468	-1.9271
H	3.6639	0.84023	-3.31741
H	5.07899	1.76456	-2.77171
C	2.15934	2.27923	0.78754
C	0.81092	2.53497	1.4756
H	2.82863	1.72008	1.46589
H	2.65661	3.23754	0.55399
H	0.97055	2.83363	2.52673
H	0.28193	3.36609	0.97453
C	0.01679	-0.01937	2.82466
C	-1.91485	1.80252	2.14049
C	-0.89201	0.47179	3.95743
H	-0.35956	-1.00011	2.48718
C	-2.2261	0.83422	3.30201
H	-1.63265	2.77516	2.581
H	-0.47331	1.37132	4.44356
H	-1.00715	-0.29932	4.73879
H	-2.93678	1.2924	4.01115
H	-2.70269	-0.07819	2.90026
P	-0.36153	1.10017	1.36327
P	2.03679	1.21638	-0.73266
C	1.50177	-0.14525	3.05578
C	4.30898	-0.27425	3.23346
C	2.23427	-1.03389	2.25252
C	2.19846	0.65642	3.96742
C	3.5909	0.5951	4.05362
C	3.62267	-1.0969	2.33833
H	1.70149	-1.67123	1.53954
H	1.6582	1.35045	4.6173
H	4.11641	1.23492	4.76909

H	4.17155	-1.79115	1.69548
H	5.40021	-0.31893	3.29648
C	-3.06406	2.02832	1.18594
C	-5.24261	2.4623	-0.54067
C	-3.36044	1.1161	0.166
C	-3.87544	3.16011	1.32538
C	-4.95622	3.37886	0.47116
C	-4.43952	1.33047	-0.68891
H	-2.71812	0.24565	0.00766
H	-3.65122	3.88902	2.11159
H	-5.57457	4.27308	0.59402
H	-4.64219	0.61018	-1.48715
H	-6.08525	2.63326	-1.21689
C	4.34949	-0.4628	-1.00279
C	5.35015	-3.07571	-0.6942
C	3.54049	-1.57506	-1.26549
C	5.67068	-0.68375	-0.59514
C	6.17129	-1.97605	-0.44433
C	4.03322	-2.86888	-1.1049
H	2.49669	-1.42804	-1.56015
H	6.31513	0.17457	-0.37698
H	7.20492	-2.1246	-0.11809
H	3.37125	-3.72012	-1.28309
H	5.73399	-4.09139	-0.56194
C	0.7159	3.44003	-1.80985
C	-1.2796	5.20263	-0.90155
C	-0.63825	3.06498	-1.83399
C	1.04819	4.72046	-1.35441
C	0.05781	5.59587	-0.90269
C	-1.62429	3.93608	-1.37786
H	-0.92375	2.06556	-2.18739
H	2.09201	5.04667	-1.3369
H	0.33845	6.59154	-0.5459
H	-2.67053	3.61659	-1.38658
H	-2.05559	5.88242	-0.53752
Cu	0.09248	0.03467	-0.61107
N	-1.07664	-0.40459	-2.0839
C	-2.9041	-1.78767	-2.98687
C	-3.43705	-2.93384	-2.54259
H	-4.42789	-3.31885	-2.80278
C	-1.60003	-1.51833	-2.2804
C	-3.48189	-0.78071	-3.92256
H	-3.34526	0.22784	-3.49658
H	-2.93355	-0.78842	-4.88217
H	-4.55017	-0.96107	-4.12793
C	-1.18744	-2.95388	-1.77749
H	-0.75121	-3.45568	-2.6574
C	-0.20394	-2.9886	-0.6473
O	-0.13377	-2.14039	0.21283
O	0.68766	-3.99371	-0.58201
C	0.67889	-5.08058	-1.48167
H	1.02752	-4.77982	-2.4849
H	1.38006	-5.82006	-1.06834
H	-0.32011	-5.54135	-1.56056
C	-3.0317	-3.46541	-0.08065
C	-3.79433	-2.36422	0.31624
C	-2.62807	-4.38232	0.89741
C	-4.13353	-2.17302	1.65527
H	-4.12536	-1.64478	-0.43595
C	-2.95873	-4.19474	2.23793
H	-2.02902	-5.25297	0.60718
C	-3.71201	-3.08448	2.62258
H	-4.72975	-1.30021	1.93834
H	-2.62625	-4.91947	2.98687
H	-3.97388	-2.9343	3.67416
C	-2.56388	-3.63649	-1.51957
H	-2.50409	-4.71917	-1.72858

D$^{S\text{-}syn}$

C	2.99312	2.31467	1.63985
C	2.59638	-0.23028	2.56811
C	3.62668	0.61925	3.32008
C	3.18009	2.07386	3.15309
H	4.00013	2.32652	1.1869
H	1.65243	-0.20402	3.13915
H	3.69398	0.32105	4.38054
H	4.63467	0.50197	2.8834
H	2.22059	2.23826	3.67681
H	3.90622	2.79068	3.57341
C	3.41006	0.21357	-0.27439
C	2.97144	-1.00524	-1.09494
H	3.52085	1.09316	-0.93406
H	4.39504	0.0256	0.18862
H	3.53646	-1.05132	-2.0428
H	3.18776	-1.9348	-0.53737
C	0.83775	-0.09393	-2.98861
C	0.97918	-2.73628	-2.26628
C	0.94957	-1.11184	-4.12959
H	-0.22215	0.20045	-2.89729
C	0.30755	-2.40294	-3.61541
H	2.00884	-3.07047	-2.48475
H	2.00686	-1.31461	-4.37653
H	0.46552	-0.73971	-5.04907
H	0.41874	-3.24144	-4.32381
H	-0.77688	-2.2513	-3.46119
P	1.14489	-1.0731	-1.41323
P	2.17787	0.74645	1.0126

C	1.66275	1.16819	-3.0199
C	3.24723	3.48611	-2.81996
C	1.23268	2.27089	-2.26421
C	2.88895	1.25583	-3.68837
C	3.67689	2.40426	-3.58674
C	2.01614	3.41792	-2.16537
H	0.28005	2.21687	-1.72662
H	3.24933	0.41924	-4.29321
H	4.63477	2.45016	-4.1136
H	1.66514	4.26245	-1.56571
H	3.86503	4.38484	-2.73588
C	0.29463	-3.81811	-1.46325
C	-0.97172	-5.86303	-0.00197
C	-0.8247	-3.54608	-0.66927
C	0.76752	-5.1345	-1.51545
C	0.14104	-6.15029	-0.79342
C	-1.4525	-4.55441	0.05826
H	-1.18259	-2.51979	-0.57125
H	1.64866	-5.36697	-2.12307
H	0.52986	-7.17171	-0.84381
H	-2.31434	-4.30433	0.68431
H	-1.45844	-6.65676	0.57214
C	2.31355	3.61505	1.28087
C	1.12896	6.08425	0.63125
C	0.92056	3.74383	1.21988
C	3.09908	4.74293	1.01279
C	2.5173	5.96797	0.69088
C	0.3359	4.96787	0.8972
H	0.28602	2.86653	1.38185
H	4.19031	4.65578	1.04625
H	3.15184	6.83365	0.47945
H	-0.75263	5.04925	0.83881
H	0.66548	7.04047	0.37228
C	2.89497	-1.67312	2.24382
C	3.33615	-4.30776	1.36249
C	1.81641	-2.54514	2.02107
C	4.19533	-2.15247	2.0498
C	4.41484	-3.46026	1.61136
C	2.0359	-3.84729	1.57871
H	0.78967	-2.18858	2.17389
H	5.05681	-1.50291	2.22937
H	5.43899	-3.81521	1.46119
H	1.18113	-4.50454	1.39439
H	3.50548	-5.32904	1.00873
Cu	0.13992	-0.08552	0.3868
N	-1.14755	-0.88814	1.61814
C	-3.47163	-1.61933	2.05388
C	-4.62849	-1.52073	1.38813
H	-5.58194	-1.93608	1.73067
C	-2.35095	-0.97202	1.31013
C	-3.20202	-2.28362	3.36279
H	-2.42461	-3.05823	3.23972
H	-2.79241	-1.55251	4.08129
H	-4.10897	-2.74241	3.78985
C	-4.52545	-0.81217	0.05369
H	-4.68199	-1.56515	-0.74135
C	-3.03562	-0.38991	-0.02794
C	-5.57054	0.26338	-0.19087
C	-5.84898	0.6683	-1.5009
C	-6.25881	0.88257	0.85617
C	-6.7791	1.67347	-1.76118
H	-5.32355	0.18824	-2.33411
C	-7.19536	1.88367	0.6024
H	-6.04056	0.5897	1.88716
C	-7.45737	2.28622	-0.70739
H	-6.97999	1.9758	-2.79346
H	-7.7233	2.35657	1.43624
H	-8.19195	3.07203	-0.90693
C	-2.61046	1.03097	-0.03941
O	-1.51953	1.38064	-0.46653
H	-2.53039	-0.86928	-0.87903
O	-3.42	1.86488	0.59605
C	-2.99625	3.20133	0.76451
H	-2.16712	3.24889	1.49015
H	-2.66684	3.63561	-0.193
H	-3.86799	3.74638	1.15214

E^R

C	4.22387	0.13974	-1.01022
C	2.6815	2.30049	-1.67778
C	4.17955	2.41629	-1.97715
C	4.66916	0.98591	-2.2229
H	4.86123	0.43244	-0.15715
H	2.17488	2.01333	-2.61577
H	4.37079	3.07922	-2.83881
H	4.72389	2.83775	-1.11299
H	4.21125	0.58242	-3.14447
H	5.76328	0.93185	-2.35524
C	2.75443	1.36486	1.14264
C	1.45877	1.7776	1.85256
H	3.21362	0.50823	1.66749
H	3.48601	2.19218	1.16647
H	1.60965	1.77608	2.94653
H	1.17573	2.80557	1.56151
C	0.00748	-0.74907	2.54406
C	-1.39396	1.59254	2.30202
C	-0.885	-0.35409	3.72681

H	-0.5248	-1.51652	1.95778
C	-2.03527	0.4691	3.14263
H	-0.92168	2.30518	3.00119
H	-0.33154	0.27055	4.45053
H	-1.2431	-1.24489	4.27125
H	-2.69085	0.89178	3.92302
H	-2.66837	-0.16689	2.496
P	-0.00328	0.73357	1.38215
P	2.50621	0.76779	-0.60031
C	1.40579	-1.25557	2.79723
C	4.09095	-2.06366	3.05918
C	2.01037	-2.07306	1.82835
C	2.16412	-0.86873	3.90835
C	3.49659	-1.26639	4.03666
C	3.33812	-2.47297	1.95709
H	1.43114	-2.3728	0.94884
H	1.72281	-0.2405	4.68708
H	4.07299	-0.94848	4.91078
H	3.78831	-3.10205	1.18355
H	5.13588	-2.37197	3.15812
C	-2.35233	2.35386	1.41956
C	-4.16897	3.77524	-0.19134
C	-2.84663	1.79377	0.23506
C	-2.78463	3.63367	1.78393
C	-3.68503	4.34196	0.98739
C	-3.74752	2.49664	-0.56206
H	-2.50995	0.80037	-0.07717
H	-2.40339	4.08791	2.70464
H	-4.00783	5.34284	1.2891
H	-4.12657	2.03532	-1.47919
H	-4.87452	4.32683	-0.81938
C	4.35647	-1.3569	-1.18933
C	4.68742	-4.13697	-1.48128
C	3.32112	-2.15085	-1.6966
C	5.56254	-1.97866	-0.84137
C	5.73149	-3.35451	-0.98651
C	3.48349	-3.52878	-1.83608
H	2.35674	-1.70085	-1.94891
H	6.38031	-1.37389	-0.43463
H	6.68012	-3.8194	-0.70204
H	2.65214	-4.12906	-2.21661
H	4.81151	-5.2187	-1.58628
C	1.92944	3.46445	-1.08268
C	0.40734	5.45634	0.20234
C	0.52765	3.47093	-1.1642
C	2.55415	4.48796	-0.36281
C	1.79906	5.4748	0.27591
C	-0.22804	4.45075	-0.52874
H	0.01439	2.66772	-1.70017
H	3.64437	4.52159	-0.28617
H	2.30817	6.26396	0.83726
H	-1.3193	4.4197	-0.59533
H	-0.18379	6.22458	0.70903
Cu	0.4075	-0.03863	-0.77941
C	-3.1577	-0.23071	-3.18203
C	-4.09568	-1.15925	-2.9312
H	-5.09775	-1.18027	-3.36941
C	-1.95307	-0.52194	-2.35285
C	-3.23167	0.93762	-4.11338
H	-3.01725	1.88532	-3.58581
H	-2.48752	0.84425	-4.92591
H	-4.22884	1.02029	-4.5748
C	-3.62471	-2.19434	-1.94139
C	-1.28135	-2.42999	-0.87623
O	-0.13035	-2.06331	-0.55825
O	-1.70264	-3.65092	-0.47918
C	-0.84379	-4.39142	0.35545
H	0.12595	-4.58751	-0.13249
H	-0.65475	-3.86711	1.3084
H	-1.36139	-5.34083	0.55647
H	-3.60666	-3.18483	-2.43549
C	-4.51323	-2.30559	-0.70967
C	-4.95044	-1.15495	-0.04286
C	-4.8639	-3.55123	-0.18337
C	-5.7046	-1.24353	1.12472
H	-4.69035	-0.17227	-0.44477
C	-5.62344	-3.64754	0.98404
H	-4.52045	-4.45978	-0.687
C	-6.04376	-2.49442	1.64506
H	-6.0304	-0.32864	1.6291
H	-5.88477	-4.63311	1.38161
H	-6.63693	-2.56882	2.56146
C	-2.22365	-1.71224	-1.63205
N	-0.87057	0.22156	-2.30796
H	-0.91082	1.00183	-2.96033

E^S

C	1.06424	-1.99544	2.3363
C	-0.74488	-0.06643	3.04354
C	-0.12973	-0.86859	4.19382
C	1.25229	-1.30613	3.70561
H	0.63861	-2.99751	2.51917
H	-0.18028	0.87825	2.948
H	-0.07769	-0.27246	5.12165
H	-0.73434	-1.76636	4.41574
H	1.90392	-0.42162	3.5841
H	1.75755	-1.98321	4.41522

C	-1.61147	-2.28793	1.28187
C	-2.80915	-1.81044	0.45014
H	-1.11473	-3.13432	0.7748
H	-1.95382	-2.6536	2.26648
H	-3.39147	-2.67615	0.08757
H	-3.48552	-1.19933	1.0747
C	-2.02615	-1.8421	-2.42664
C	-4.02305	-0.16312	-1.5876
C	-3.38503	-1.99561	-3.11858
H	-1.37643	-1.23765	-3.08313
C	-4.04838	-0.61657	-3.06307
H	-4.76314	-0.77557	-1.043
H	-4.02281	-2.72049	-2.58151
H	-3.27166	-2.36432	-4.15277
H	-5.08171	-0.6288	-3.4498
H	-3.47903	0.10138	-3.68146
P	-2.33831	-0.70193	-0.96376
P	-0.28062	-1.00277	1.47677
C	-1.24512	-3.06236	-2.00658
C	0.27403	-5.20153	-0.98798
C	0.1318	-2.91932	-1.77115
C	-1.84266	-4.29929	-1.74274
C	-1.08875	-5.36039	-1.23511
C	0.88355	-3.97609	-1.26586
H	0.61145	-1.95171	-1.95495
H	-2.91074	-4.44831	-1.92429
H	-1.57519	-6.31925	-1.03191
H	1.95149	-3.83431	-1.07573
H	0.86223	-6.03157	-0.58557
C	-4.36251	1.29162	-1.35692
C	-5.05676	3.98463	-0.91657
C	-3.41034	2.30927	-1.50238
C	-5.6679	1.64893	-0.999
C	-6.01662	2.98169	-0.78192
C	-3.75345	3.64193	-1.27972
H	-2.37558	2.05993	-1.75598
H	-6.42365	0.86591	-0.87539
H	-7.04199	3.23657	-0.49807
H	-2.98973	4.41798	-1.38818
H	-5.32357	5.03053	-0.73921
C	2.33978	-2.15832	1.54333
C	4.74482	-2.48503	0.12472
C	2.91887	-1.08255	0.85918
C	2.98944	-3.39753	1.51085
C	4.18244	-3.56359	0.80717
C	4.11079	-1.24246	0.15545
H	2.42738	-0.10372	0.85272
H	2.54698	-4.25053	2.03665
H	4.67261	-4.54181	0.79119
H	4.53874	-0.38932	-0.37751
H	5.67976	-2.60819	-0.42964
C	-2.21312	0.27126	3.06407
C	-4.96262	0.83287	2.82067
C	-2.66901	1.3248	2.2571
C	-3.1605	-0.4782	3.76964
C	-4.52409	-0.20064	3.64707
C	-4.02576	1.6039	2.12985
H	-1.94	1.91473	1.69615
H	-2.84231	-1.29828	4.41895
H	-5.24876	-0.80237	4.20377
H	-4.35254	2.42083	1.48019
H	-6.03092	1.04433	2.7186
Cu	-0.46157	0.4055	-0.36209
C	1.69274	4.14597	-0.04901
C	2.96017	4.25547	-0.47834
H	3.60649	5.12524	-0.32924
C	1.17569	2.79255	-0.40223
C	0.87183	5.15486	0.6895
H	-0.03965	5.42068	0.12248
H	0.54261	4.76047	1.66859
H	1.44128	6.08074	0.86926
C	3.43737	3.00258	-1.16788
H	3.71705	3.23663	-2.21236
C	4.65325	2.39103	-0.48406
C	5.7646	1.97597	-1.22148
C	4.65636	2.18384	0.90122
C	6.8524	1.36411	-0.59433
H	5.76811	2.11622	-2.30663
C	5.73894	1.57701	1.53177
H	3.78432	2.49253	1.48714
C	6.84374	1.16274	0.78465
H	7.71102	1.04014	-1.19053
H	5.71833	1.41594	2.6137
H	7.69228	0.67942	1.27753
N	-0.01914	2.34169	-0.09125
H	-0.59369	3.04786	0.36448
C	2.20695	2.12449	-1.10816
C	2.0652	0.89939	-1.78365
O	3.1379	0.55274	-2.53079
O	1.07783	0.13738	-1.76453
C	3.09982	-0.70526	-3.16479
H	4.05368	-0.80045	-3.70436
H	3.00968	-1.52014	-2.42601
H	2.25881	-0.7729	-3.87546

3A

C	0.94453	-1.79056	-0.96373

C	2.11784	-1.61688	-0.32663
C	2.17738	-0.21444	0.13529
C	1.02255	0.43501	-0.23797
H	0.59508	-2.72572	-1.40808
C	0.12447	-0.52494	-0.97771
H	-0.0117	-0.19762	-2.02619
C	-1.25407	-0.71564	-0.36227
C	-2.4137	-0.4579	-1.09475
C	-1.3783	-1.15202	0.96191
C	-3.67461	-0.63451	-0.52153
H	-2.33069	-0.10264	-2.12679
C	-2.6331	-1.32976	1.53809
H	-0.47329	-1.3466	1.54702
C	-3.78838	-1.07181	0.79631
H	-4.57292	-0.42426	-1.10976
H	-2.71228	-1.67034	2.57487
H	-4.77506	-1.2095	1.24825
C	0.78303	1.82725	0.04514
O	-0.37814	2.27629	-0.46285
O	1.53847	2.552	0.67529
N	3.21809	0.30607	0.81324
H	3.1558	1.28632	1.07966
H	4.04503	-0.23715	1.01231
C	3.21025	-2.61173	-0.08973
H	4.15843	-2.28517	-0.55569
H	3.40107	-2.74731	0.9909
H	2.94887	-3.59472	-0.51195
C	-0.70817	3.6216	-0.19052
H	-0.77108	3.80104	0.89564
H	0.04288	4.30837	-0.61559
H	-1.68783	3.79443	-0.65829

[(**L3**)CuOH]

C	2.89852	-1.69565	-0.51625
C	0.41134	-2.83282	-0.37495
C	1.48852	-3.65156	-1.09284
C	2.81887	-3.23771	-0.45826
H	3.11426	-1.41321	-1.5614
H	0.35202	-3.19634	0.66594
H	1.30067	-4.73535	-1.0012
H	1.51368	-3.41435	-2.17163
H	2.85097	-3.56949	0.59556
H	3.68849	-3.68978	-0.96489
C	0.75745	-0.21298	-1.76032
C	-0.59409	0.51448	-1.7447
H	1.57955	0.5164	-1.87742
H	0.80878	-0.90368	-2.62099
H	-0.61756	1.2939	-2.52701
H	-1.40704	-0.19969	-1.9698
C	-0.34827	2.96177	-0.06406
C	-2.80148	1.79114	-0.37833
C	-1.42503	3.82903	-0.72278
H	-0.32727	3.21821	1.01001
C	-2.75853	3.32174	-0.16377
H	-2.99833	1.61523	-1.45094
H	-1.41875	3.7014	-1.82054
H	-1.26533	4.9016	-0.51621
H	-3.62707	3.80561	-0.64199
H	-2.82051	3.54231	0.91718
P	-1.04383	1.22013	-0.08502
P	1.14661	-1.11244	-0.17923
Cu	-0.11465	-0.1958	1.45295
C	1.07065	2.96779	-0.56952
C	3.71519	2.679	-1.49036
C	2.07915	2.46986	0.27101
C	1.4169	3.3415	-1.87169
C	2.72913	3.19622	-2.32898
C	3.38686	2.32303	-0.18046
H	1.82656	2.1689	1.29349
H	0.65819	3.74394	-2.54852
H	2.97921	3.49057	-3.35262
H	4.15125	1.92181	0.49113
H	4.74099	2.55933	-1.85057
C	-3.87128	1.06729	0.41385
C	-5.93547	-0.25865	1.79282
C	-3.6354	0.4991	1.67233
C	-5.15548	0.96309	-0.13824
C	-6.18133	0.30896	0.54133
C	-4.6613	-0.16079	2.35007
H	-2.6415	0.4853	2.13337
H	-5.35171	1.39185	-1.12738
H	-7.17436	0.23467	0.08778
H	-4.43533	-0.61934	3.31672
H	-6.73422	-0.78314	2.32575
C	3.96259	-1.08244	0.36292
C	5.97923	0.05542	1.96158
C	5.19629	-0.71872	-0.18871
C	3.75836	-0.87637	1.7332
C	4.75565	-0.30986	2.52515
C	6.19842	-0.15494	0.60045
H	5.37055	-0.8679	-1.25957
H	2.79242	-1.13091	2.18165
H	4.57129	-0.14626	3.59082
H	7.15329	0.1277	0.14752
H	6.75955	0.50437	2.58273
C	-0.99351	-2.77293	-0.92197
C	-3.60046	-2.40091	-1.91017

C	-2.03721	-2.38419	-0.06452
C	-1.28334	-2.99489	-2.27248
C	-2.57707	-2.80808	-2.76428
C	-3.32692	-2.19658	-0.55639
H	-1.83333	-2.17853	0.99503
H	-0.49472	-3.31019	-2.96136
H	-2.78253	-2.98272	-3.82473
H	-4.12174	-1.87858	0.12414
H	-4.61312	-2.24695	-2.29383
O	-0.9741	-0.70662	3.01461
H	-0.59314	-1.47701	3.45107

H_2O

O	0	0	0.11785
H	0	-0.76632	-0.47139
H	0	0.76632	-0.47139

15^I^

C	-1.99655	1.55819	-0.2532
C	-2.70236	0.22682	-0.29981
C	-1.54865	-0.74653	-0.22552
C	-0.35713	-0.05399	-0.13844
H	-2.52213	2.513	-0.28881
C	-0.66227	1.39302	-0.15794
H	-3.1999	0.09169	-1.27938
C	0.90698	-0.75495	-0.04504
O	1.01925	-1.97244	-0.03464
O	1.97884	0.04993	0.03165
C	3.2514	-0.57749	0.12721
H	3.4041	-1.22899	-0.748
H	3.27158	-1.22902	1.01537
C	4.30007	0.50869	0.20601
H	5.30164	0.06127	0.28139
H	4.27207	1.14596	-0.68976
H	4.13839	1.14599	1.08749
N	-1.71165	-2.07588	-0.23194
H	-0.86948	-2.64333	-0.16621
H	-2.6266	-2.49226	-0.30387
C	-3.7328	0.02135	0.81513
H	-4.53604	0.76825	0.73833
H	-4.19889	-0.9756	0.76556
H	-3.25563	0.13015	1.80028
C	0.35981	2.48232	-0.08782
H	1.06796	2.4106	-0.92674
H	-0.12171	3.46982	-0.1118
H	0.9604	2.39922	0.83008

15^I^-15^II^

C	-1.93616	1.58277	-0.02188
C	-2.6233	0.24555	-0.05049
C	-1.59574	-0.71656	-0.02936
C	-0.34465	-0.03821	0.00989
H	-2.45589	2.52774	0.13624
C	-0.54782	1.36187	0.01982
H	-2.34076	1.03949	-1.09535
C	0.92493	-0.76958	0.00609
O	1.02448	-1.98117	0.02895
O	2.00227	0.02656	-0.00535
C	3.27533	-0.61004	0.01616
H	3.35954	-1.28267	-0.85201
H	3.35076	-1.24081	0.91609
C	4.33429	0.46836	-0.00402
H	5.33509	0.01336	0.0114
H	4.24842	1.08488	-0.91052
H	4.24009	1.12692	0.87156
N	-1.77992	-2.08001	-0.10798
H	-0.93015	-2.6135	0.05017
H	-2.60508	-2.4471	0.34715
C	-4.09352	0.01093	0.10791
H	-4.67721	0.86839	-0.25775
H	-4.41992	-0.87744	-0.45454
H	-4.36298	-0.13761	1.1672
C	0.47972	2.45195	0.03309
H	1.13398	2.40475	-0.8503
H	-0.00626	3.43791	0.05367
H	1.13731	2.37034	0.91081

15^II^

C	-1.99722	1.57335	0.02796
C	-2.6373	0.21978	-0.02076
C	-1.65434	-0.71859	-0.03132
C	-0.33694	-0.03674	0.00473
H	-2.27462	2.15923	0.92594
C	-0.5201	1.30984	0.03628
H	-2.26425	2.2113	-0.83689
C	0.94094	-0.77045	0.04897
O	1.04175	-1.9728	0.17144
O	2.01035	0.02614	-0.05083
C	3.28974	-0.60061	0.00198
H	3.36225	-1.34018	-0.81078
H	3.38118	-1.15582	0.94874
C	4.34085	0.4779	-0.12274
H	5.34408	0.0296	-0.0892

H	4.23584	1.01946	-1.07391
H	4.25915	1.20331	0.69967
N	-1.76755	-2.08822	-0.11951
H	-0.92568	-2.60242	0.11548
H	-2.63004	-2.49264	0.217
C	-4.11523	-0.00014	-0.06469
H	-4.59768	0.64469	-0.81835
H	-4.3611	-1.03919	-0.33231
H	-4.60664	0.21685	0.9007
C	0.48415	2.4164	0.08024
H	1.08828	2.43735	-0.83947
H	-0.01497	3.38947	0.19079
H	1.1929	2.28525	0.91037

15^{II}-15^{III}

C	2.0087	1.52419	0.04322
C	2.62925	0.25292	-0.00798
C	1.60191	-0.7038	-0.04331
C	0.334	-0.04542	-0.02628
H	2.49328	2.49376	-0.08964
C	0.55298	1.35673	0.01616
H	1.29029	1.64085	1.12512
C	-0.92661	-0.76877	-0.03336
O	-1.0296	-1.97825	-0.12565
O	-2.00831	0.02543	0.05423
C	-3.2785	-0.61444	0.02812
H	-3.34226	-1.3338	0.85991
H	-3.37506	-1.19567	-0.9027
C	-4.34064	0.45649	0.13152
H	-5.34047	-0.0004	0.10877
H	-4.23992	1.01956	1.07089
H	-4.2653	1.16506	-0.7062
N	1.7726	-2.05599	0.01887
H	0.94612	-2.61678	-0.15843
H	2.67176	-2.43826	-0.23294
C	4.09255	-0.04587	0.09699
H	4.33453	-0.61647	1.01103
H	4.45171	-0.64122	-0.76031
H	4.68755	0.87823	0.11751
C	-0.41259	2.48017	-0.22234
H	-1.31845	2.37465	0.38592
H	0.05834	3.45052	-0.00757
H	-0.72331	2.48121	-1.27845

15^{III}

C	-2.06162	1.46133	-0.34908
C	-2.60812	0.24425	-0.16487
C	-1.5059	-0.71998	0.01701
C	-0.30222	-0.0518	-0.0597
H	-2.62407	2.38549	-0.4994
C	-0.56001	1.41494	-0.2939
H	-0.15827	1.71854	-1.27987
C	0.9599	-0.73879	0.02862
O	1.0912	-1.93879	0.22647
O	2.02513	0.07045	-0.13665
C	3.30861	-0.53434	-0.05856
H	3.39305	-1.3146	-0.83204
H	3.41755	-1.04004	0.91425
C	4.3466	0.54931	-0.24442
H	5.35666	0.11706	-0.1989
H	4.22496	1.04421	-1.21889
H	4.26159	1.31277	0.54269
N	-1.68896	-2.03652	0.22258
H	-0.85265	-2.60807	0.30893
H	-2.60726	-2.45	0.20274
C	-4.05246	-0.14149	-0.13691
H	-4.28648	-0.87194	-0.92934
H	-4.32284	-0.60369	0.82695
H	-4.69998	0.7329	-0.2851
C	0.02196	2.35354	0.77056
H	1.11598	2.2613	0.79545
H	-0.2353	3.40283	0.55802
H	-0.37135	2.09378	1.76537

15^{III}-15^{IV}

C	-1.98989	1.54578	-0.02506
C	-2.6148	0.2845	0.0198
C	-1.62682	-0.70651	-0.00784
C	-0.34578	-0.06761	-0.10024
H	-2.51551	2.50203	-0.01286
C	-0.59901	1.42884	-0.07522
H	-0.34023	0.66181	-1.1495
C	0.92771	-0.78785	0.02739
O	1.0155	-1.98872	0.16815
O	1.99994	0.0084	-0.05637
C	3.27854	-0.61692	0.03376
H	3.37029	-1.36887	-0.76575
H	3.34867	-1.15635	0.99114
C	4.3313	0.46088	-0.08266
H	5.33373	0.01526	-0.01118
H	4.2537	0.98355	-1.04703
H	4.22361	1.20175	0.72262
N	-1.83426	-2.06499	0.02871
H	-1.00455	-2.63793	-0.07057
H	-2.67895	-2.41923	-0.39809

C	-4.08725	0.00817	0.02247
H	-4.43937	-0.38416	-0.94772
H	-4.34436	-0.7406	0.78947
H	-4.66356	0.91947	0.23479
C	0.41611	2.50712	0.1648
H	1.29584	2.40841	-0.48343
H	-0.04131	3.49024	-0.01606
H	0.77766	2.48375	1.20573

15^{IV}

C	1.82699	1.54227	-0.24233
C	2.54107	0.26265	-0.16705
C	1.67269	-0.67073	0.29898
C	0.34097	-0.01345	0.59091
H	2.29183	2.47398	-0.57507
C	0.54275	1.42987	0.15083
H	0.19067	-0.01834	1.68828
C	-0.85353	-0.75088	0.01624
O	-0.8223	-1.86018	-0.45783
O	-1.97878	-0.05045	0.13977
C	-3.18051	-0.65624	-0.3397
H	-3.34235	-1.60363	0.19794
H	-3.05479	-0.90677	-1.40433
C	-4.31249	0.31922	-0.11845
H	-5.25754	-0.11869	-0.47004
H	-4.41846	0.56107	0.94874
H	-4.1385	1.25447	-0.66971
N	1.87584	-2.00493	0.59799
H	1.08661	-2.58448	0.32046
H	2.75297	-2.37789	0.25304
C	3.97605	0.06746	-0.54446
H	4.66524	0.4475	0.22872
H	4.21786	-0.99544	-0.70169
H	4.21575	0.5968	-1.48014
C	-0.49177	2.50516	0.23124
H	-0.94545	2.55967	1.2342
H	-0.04053	3.4823	0.00652
H	-1.31897	2.33694	-0.47561

15^{IV}-15^{V}

C	1.9528	1.54611	-0.08736
C	2.62026	0.3068	-0.05838
C	1.65089	-0.70451	0.08036
C	0.32182	-0.00653	0.15871
H	2.45931	2.51324	-0.11689
C	0.57404	1.39227	0.04347
H	1.03642	-0.4174	1.20409
C	-0.94141	-0.76055	0.13276
O	-1.0218	-1.96664	0.23605
O	-2.00574	0.02485	-0.02946
C	-3.28084	-0.61381	-0.08038
H	-3.43623	-1.18105	0.85071
H	-3.28507	-1.34299	-0.90553
C	-4.32839	0.45836	-0.26953
H	-5.32778	0.00231	-0.31118
H	-4.30931	1.17594	0.56323
H	-4.16008	1.00987	-1.20566
N	1.87432	-2.08764	-0.03313
H	0.9969	-2.6038	-0.02142
H	2.39918	-2.30817	-0.87368
C	4.08833	0.04876	-0.18938
H	4.39372	-0.80047	0.44088
H	4.37059	-0.19861	-1.22768
H	4.67627	0.92961	0.10522
C	-0.42886	2.49847	0.1765
H	-0.9832	2.43638	1.12572
H	0.07558	3.47444	0.13913
H	-1.1782	2.46628	-0.62831

15^{V}

C	-2.06298	1.47342	0.01074
C	-2.66055	0.27236	-0.12121
C	-1.59542	-0.79264	-0.24029
C	-0.30721	0.00052	-0.10569
H	-2.57116	2.43631	0.09696
C	-0.59984	1.32372	0.02046
H	-1.63737	-1.18247	-1.277
C	0.97253	-0.71279	-0.15386
O	1.0588	-1.91455	-0.29604
O	2.05584	0.06663	-0.01926
C	3.32283	-0.58394	-0.0533
H	3.42654	-1.1259	-1.00652
H	3.3648	-1.33826	0.74827
C	4.39257	0.47092	0.11203
H	5.38754	0.00367	0.09052
H	4.34161	1.21175	-0.69909
H	4.2793	0.99792	1.07057
N	-1.81687	-1.91498	0.65429
H	-1.08197	-2.6019	0.49038
H	-1.70125	-1.60022	1.61725
C	-4.11088	-0.06133	-0.1486
H	-4.40077	-0.45503	-1.13638
H	-4.3218	-0.86174	0.57738
H	-4.73698	0.81335	0.07411
C	0.30064	2.50946	0.14247

H	0.13881	3.18962	-0.70934
H	0.0472	3.07756	1.0519
H	1.35628	2.22539	0.17672

15^{V}-15^{I}

C	-1.96153	1.56768	0.01806
C	-2.6264	0.32379	-0.0357
C	-1.5878	-0.71363	-0.07573
C	-0.32378	-0.02363	-0.04117
H	-2.45926	2.53663	-0.01147
C	-0.57628	1.36429	0.00726
H	-2.11568	-0.29169	-1.16008
C	0.93558	-0.75297	-0.07435
O	1.03206	-1.96275	-0.17044
O	2.01528	0.03724	0.02637
C	3.28574	-0.60365	0.01314
H	3.39106	-1.184	-0.91721
H	3.33892	-1.32404	0.84486
C	4.347	0.46648	0.12989
H	5.34648	0.00839	0.12121
H	4.28347	1.17438	-0.70922
H	4.23317	1.03086	1.06679
N	-1.84741	-2.07818	0.09123
H	-0.98481	-2.60786	-0.02595
H	-2.25946	-2.27987	0.99804
C	-4.08972	0.03391	0.0961
H	-4.69837	0.80413	-0.39704
H	-4.33573	-0.94707	-0.3342
H	-4.37362	0.01114	1.1603
C	0.43919	2.46632	-0.03501
H	1.05666	2.40476	-0.9431
H	-0.05274	3.44863	-0.00999
H	1.13098	2.39537	0.81618

16^{III}

C	0.0774	-0.91574	-0.33262
C	1.11547	-0.11318	-0.01875
C	0.6054	1.26566	0.1099
C	-0.71909	1.27987	-0.12449
H	0.12508	-1.99481	-0.49136
H	1.22665	2.12907	0.35735
H	-1.37744	2.14956	-0.09851
C	-1.19122	-0.1144	-0.43811
H	-1.53905	-0.13556	-1.49063
C	-2.33203	-0.60952	0.45733
H	-3.22284	0.02799	0.35191
H	-2.61988	-1.63852	0.1946
H	-2.02373	-0.59855	1.5137
C	2.54975	-0.49098	0.17601
H	3.1979	0.03555	-0.54303
H	2.89731	-0.21363	1.18425
H	2.70188	-1.57142	0.04615

16^{III}-16^{II}

C	0.03865	-0.92043	-0.03013
C	1.17113	-0.08281	0.04682
C	0.70606	1.24183	0.04799
C	-0.68961	1.27065	-0.03224
H	0.00248	-2.00658	0.06609
H	1.35199	2.12202	0.0421
H	-1.31879	2.15935	-0.07307
C	-1.17057	-0.05582	-0.08086
H	-0.61191	-0.59686	-1.13075
C	-2.57974	-0.53895	0.10447
H	-3.30046	0.1495	-0.35926
H	-2.72874	-1.53552	-0.33685
H	-2.82233	-0.61304	1.17623
C	2.60182	-0.53442	0.03198
H	3.00255	-0.6031	-0.99329
H	3.24108	0.16934	0.58503
H	2.71775	-1.52541	0.49561

16^{II}

C	0	-0.95149	0.00022
C	1.19038	-0.02747	-0.00003
C	0.73342	1.24277	0.00002
C	-0.73342	1.24277	0.00003
H	-0.00001	-1.61613	0.88368
H	1.35555	2.13992	0.0001
H	-1.35554	2.13993	0.00011
C	-1.19038	-0.02747	-0.00003
H	0	-1.61737	-0.88229
C	-2.60538	-0.50664	-0.00015
H	-3.31079	0.33632	0.00023
H	-2.81988	-1.12816	-0.88547
H	-2.8198	-1.12888	0.88469
C	2.60538	-0.50664	-0.00015
H	2.8198	-1.12838	-0.88533
H	3.31079	0.33633	-0.00004
H	2.81989	-1.12866	0.88482

17^{III}

C	-0.51626	-1.16529	-0.29051

C	0.72608	-0.70112	-0.06026
C	0.66951	0.78028	-0.04561
C	-0.60445	1.18083	-0.26724
H	-0.80375	-2.21694	-0.34958
H	-0.96364	2.20989	-0.30498
C	1.98582	-1.47682	0.16061
H	2.72787	-1.27493	-0.63094
H	2.45284	-1.20429	1.12089
H	1.79219	-2.55796	0.16593
C	-1.48652	-0.02584	-0.4415
H	-1.8895	-0.04507	-1.47422
C	-2.66711	-0.0966	0.53342
H	-3.33602	0.76751	0.40236
H	-3.26141	-1.01004	0.37521
H	-2.30537	-0.09432	1.57294
N	1.79428	1.53899	0.23642
H	2.67167	1.15461	-0.09257
H	1.71266	2.52596	0.02655

17^{III}-17^{II}

C	-0.5221	-1.15227	-0.0457
C	0.79729	-0.6649	0.045
C	0.72356	0.74388	0.04474
C	-0.61879	1.15147	-0.0432
H	-0.85711	-2.18228	0.08699
H	-0.97546	2.17981	-0.10327
C	2.06118	-1.46915	0.03588
H	2.65966	-1.30467	-0.87904
H	2.70585	-1.21466	0.89369
H	1.84852	-2.54619	0.0926
C	-1.43932	0.00518	-0.09592
H	-1.08998	-0.7118	-1.15918
C	-2.92291	-0.07091	0.11839
H	-3.4405	0.76929	-0.36652
H	-3.34251	-1.00578	-0.28215
H	-3.15621	-0.03626	1.19439
N	1.8302	1.58341	0.09762
H	2.68791	1.18753	-0.26739
H	1.67491	2.52144	-0.24857

17^{II}

C	-0.54604	-1.18786	-0.00195
C	0.84909	-0.62333	-0.00826
C	0.75475	0.73111	0.00676
C	-0.66478	1.1217	0.00859
H	-0.74292	-1.81659	0.88719
H	-1.01576	2.15627	0.02599
C	2.08211	-1.46845	-0.00451
H	2.02228	-2.27459	-0.75467
H	2.98494	-0.8809	-0.2353
H	2.25675	-1.95364	0.9722
C	-1.44289	0.02247	-0.00216
H	-0.74282	-1.83225	-0.87945
C	-2.93446	-0.05597	-0.0044
H	-3.38865	0.94495	0.00432
H	-3.30572	-0.59082	-0.89429
H	-3.3071	-0.60634	0.87528
N	1.76858	1.67837	0.08307
H	2.69498	1.32824	-0.13073
H	1.57729	2.53903	-0.41648

18^{III}

C	-2.71631	0.26374	-0.23206
C	-2.3333	-1.01952	-0.04722
C	-0.86751	-1.04659	-0.00001
C	-0.38266	0.20693	-0.15175
H	-3.74685	0.61777	-0.30074
C	-3.19681	-2.23391	0.08752
H	-2.97895	-2.96411	-0.70802
H	-3.01793	-2.74114	1.04889
H	-4.26285	-1.97465	0.03053
C	-1.52596	1.17358	-0.3125
H	-1.46818	1.61577	-1.32606
C	-1.54314	2.31606	0.71145
H	-0.6309	2.9199	0.61616
H	-2.41585	2.96652	0.55234
H	-1.59593	1.91328	1.73433
H	-0.26073	-1.94452	0.12777
C	1.02857	0.62055	-0.19453
O	1.40362	1.75179	-0.39875
O	1.87137	-0.39955	0.01853
C	3.26272	-0.0959	-0.01099
H	3.51713	0.34325	-0.98878
H	3.48199	0.67064	0.74925
C	4.02558	-1.37502	0.24562
H	5.10694	-1.17698	0.23087
H	3.76419	-1.79674	1.22687
H	3.80085	-2.12653	-0.52495

18^{III}-18^{II}

C	-2.68948	0.24501	-0.00543
C	-2.27038	-1.10689	0.03324
C	-0.87477	-1.09976	0.03537
C	-0.38905	0.21953	-0.01137

H	-3.70599	0.63103	0.08766
C	-3.17	-2.30638	-0.00607
H	-2.63935	-3.20174	0.34739
H	-4.05674	-2.17032	0.6313
H	-3.52897	-2.51774	-1.02682
C	-1.49611	1.11149	-0.0332
H	-2.17239	0.79024	-1.09871
C	-1.5111	2.59766	0.17727
H	-0.70172	3.08371	-0.37945
H	-2.47837	3.0288	-0.11874
H	-1.35559	2.81821	1.24431
H	-0.23992	-1.98574	0.00806
C	1.01567	0.64134	-0.05761
O	1.40523	1.78399	-0.14415
O	1.85633	-0.40331	0.01259
C	3.2477	-0.10685	-0.01728
H	3.48478	0.4342	-0.94732
H	3.49299	0.57115	0.81577
C	4.00495	-1.4114	0.08054
H	5.08765	-1.22085	0.06119
H	3.76194	-1.93552	1.01613
H	3.75469	-2.0734	-0.76108

18^{II}

C	-2.70466	0.31089	-0.00924
C	-2.24423	-1.12142	0.00249
C	-0.89632	-1.12818	0.00736
C	-0.39426	0.25353	0.00187
H	-3.33236	0.55684	0.86733
C	-3.17909	-2.28658	0.00518
H	-3.8321	-2.27979	-0.88319
H	-2.6281	-3.23731	0.01338
H	-3.84034	-2.26934	0.8873
C	-1.43977	1.12212	-0.00907
H	-3.32299	0.54427	-0.89581
C	-1.42178	2.61538	-0.0096
H	-0.75937	3.0044	-0.7957
H	-2.43249	3.02398	-0.15128
H	-1.01932	3.00222	0.93974
H	-0.25343	-2.00843	0.01485
C	1.02438	0.65608	0.00734
O	1.42766	1.79552	0.02029
O	1.84425	-0.40528	-0.00571
C	3.24247	-0.13491	-0.00124
H	3.49511	0.47832	-0.8809
H	3.49198	0.46592	0.88779
C	3.97413	-1.45731	-0.00939
H	5.0602	-1.28633	-0.0066
H	3.71501	-2.05339	0.87751
H	3.71762	-2.04108	-0.90518

3a^{III}

C	0.10733	2.17409	-0.44081
C	-0.72387	1.36399	0.24176
C	0.0384	0.16346	0.64266
C	1.33603	0.28896	0.19462
H	-0.16474	3.14821	-0.85347
C	1.49285	1.59945	-0.53369
H	1.73277	1.41304	-1.59825
C	2.31114	-0.75844	0.36263
O	2.11825	-1.80639	0.96151
O	3.49392	-0.48683	-0.22463
C	4.51018	-1.47307	-0.1053
H	4.15694	-2.41765	-0.54932
H	4.70067	-1.67634	0.96066
C	5.7464	-0.95598	-0.80566
H	6.55498	-1.6985	-0.74236
H	5.54027	-0.75782	-1.86754
H	6.09776	-0.02222	-0.34279
N	-0.48456	-0.85611	1.3513
H	0.12672	-1.65794	1.48673
H	-1.48703	-0.97475	1.39662
C	-2.16597	1.58834	0.58921
H	-2.2694	1.66456	1.68462
H	-2.48294	2.56124	0.18049
C	2.56076	2.53897	0.04059
H	3.54913	2.06403	-0.01923
H	2.59548	3.48852	-0.51576
H	2.34568	2.76211	1.09682
C	-3.09446	0.49912	0.08345
C	-2.9508	-0.0169	-1.21011
C	-4.11151	-0.01145	0.89733
C	-3.80486	-1.01213	-1.67868
H	-2.1506	0.36239	-1.85113
C	-4.96808	-1.00938	0.43126
H	-4.23506	0.37768	1.91211
C	-4.81699	-1.51259	-0.85914
H	-3.67647	-1.4042	-2.69004
H	-5.75528	-1.39623	1.08224
H	-5.48369	-2.29626	-1.22515

3a^{III}-3a^{II}

C	-0.1441	2.21743	0.09946
C	0.73759	1.30841	-0.53136
C	0.00467	0.13947	-0.80308

C	-1.33892	0.30732	-0.34494
H	0.03801	3.27776	0.2871
C	-1.47191	1.60842	0.20899
H	-0.58268	1.73229	1.23044
C	-2.31904	-0.76616	-0.38061
O	-2.11642	-1.87385	-0.8423
O	-3.50812	-0.43136	0.15078
C	-4.51884	-1.43265	0.14504
H	-4.16309	-2.31406	0.7019
H	-4.69915	-1.76235	-0.89037
C	-5.7626	-0.84108	0.76855
H	-6.57155	-1.58568	0.7757
H	-5.57185	-0.5293	1.80592
H	-6.10546	0.03634	0.20109
N	0.49853	-0.99458	-1.37373
H	-0.11665	-1.80138	-1.3496
H	1.49496	-1.15768	-1.31962
C	2.20076	1.5032	-0.7996
H	2.39086	1.48446	-1.88679
H	2.49664	2.5107	-0.46237
C	-2.71347	2.37216	0.56552
H	-3.38464	1.7836	1.20168
H	-2.45988	3.31284	1.07573
H	-3.26604	2.62304	-0.35289
C	3.11126	0.48045	-0.13914
C	2.87141	0.04189	1.16868
C	4.21234	-0.0453	-0.82363
C	3.71333	-0.88511	1.77816
H	2.00285	0.42829	1.70838
C	5.0575	-0.97591	-0.21817
H	4.40993	0.27787	-1.84981
C	4.81122	-1.39813	1.08651
H	3.50837	-1.21458	2.7994
H	5.91012	-1.37559	-0.772
H	5.46863	-2.12921	1.56203

Cyclopentadiene

C	0.000005	-1.21362	0.000195
H	0.000017	-1.87906	-0.88229
H	0	-1.87858	0.883062
C	1.176766	-0.28295	-0.00021
H	2.216492	-0.61126	-0.00032
C	0.734289	0.990239	0.000065
H	1.355175	1.887304	0.000144
C	-1.17676	-0.28296	-0.00025
H	-2.21649	-0.61128	-0.00037
C	-0.7343	0.990233	0.000122
H	-1.35519	1.887292	0.000232

Cyclopentadienyl anion

C	0.655872	1.006984	0.000119
H	1.253791	1.925006	0.000216
C	1.160405	-0.31258	-0.00011
H	2.218251	-0.59759	-0.00019
C	0.061281	-1.20016	0.000058
H	0.117151	-2.2943	0.000087
C	-0.75505	0.934941	-6E-06
H	-1.44338	1.787268	0.000002
C	-1.1225	-0.42918	-6.3E-05
H	-2.14583	-0.82041	-0.00012

19

C	1.961421	1.564182	-0.02772
C	2.625783	0.289972	-0.01209
C	1.640636	-0.68636	0.025705
C	0.352542	-0.04147	0.036359
H	2.465382	2.536612	-0.04668
C	0.589513	1.384557	0.008653
C	-0.874	-0.75856	0.025857
O	-1.03789	-1.98062	0.034665
O	-1.99453	0.047892	-0.00461
C	-3.23264	-0.6088	-0.01908
H	-3.33534	-1.26453	0.863885
H	-3.31052	-1.27318	-0.8983
C	-4.31885	0.44904	-0.04017
H	-5.31687	-0.01703	-0.05193
H	-4.24519	1.096568	0.846589
H	-4.22011	1.087512	-0.9311
N	1.858703	-2.08748	0.076998
H	0.949415	-2.54997	0.060675
H	2.386896	-2.40136	-0.73433
C	4.102764	0.032916	-0.01829
H	4.319287	-0.9566	0.420861
H	4.555021	0.039509	-1.03135
H	4.654015	0.786395	0.572268
C	-0.43365	2.482214	0.028552
H	-1.08356	2.435716	0.919986
H	0.063941	3.466965	0.022589
H	-1.11518	2.441415	-0.83931

20

C	-2.0441	1.425741	-0.36635
C	-2.62637	0.232643	-0.17709

C	-1.58049	-0.81699	0.043676
C	-0.31372	-0.12972	-0.02585
H	-2.57559	2.368582	-0.53843
C	-0.53696	1.345444	-0.27718
H	-0.09682	1.665719	-1.24543
C	0.922584	-0.78665	0.043957
O	1.15903	-1.98166	0.238622
O	2.010355	0.079214	-0.14612
C	3.27673	-0.51006	-0.0742
H	3.387793	-1.29688	-0.84268
H	3.418942	-1.01988	0.895974
C	4.313154	0.580833	-0.2685
H	5.332512	0.163947	-0.23501
H	4.171578	1.079521	-1.23967
H	4.224092	1.346141	0.517979
N	-1.91798	-2.05357	0.232402
H	-1.04925	-2.58454	0.355529
C	-4.07681	-0.11767	-0.16886
H	-4.28004	-0.89183	-0.92586
H	-4.34916	-0.57144	0.797458
H	-4.72019	0.756514	-0.35616
C	0.019679	2.283217	0.804621
H	1.111417	2.170426	0.870279
H	-0.21272	3.342532	0.589159
H	-0.41393	2.0249	1.784561

21

C	-1.97359	-2.98766	-1.18019
C	-0.66421	-2.79737	-1.41229
C	-0.23087	-1.52736	-0.75398
C	-1.38167	-0.98754	-0.10847
H	-2.55684	-3.84184	-1.53622
C	-2.56871	-1.90149	-0.32557
C	-1.39909	0.229782	0.596462
O	-0.42523	0.977002	0.823635
O	-2.62127	0.594761	1.056116
C	-2.70927	1.789479	1.81715
H	-2.37942	2.647319	1.207947
H	-2.03109	1.732716	2.684192
C	-4.1448	1.959488	2.257849
H	-4.25063	2.886418	2.840992
H	-4.81846	2.019474	1.389637
H	-4.46714	1.117325	2.888903
N	0.982803	-1.02754	-0.80293
C	0.275664	-3.65749	-2.19278
H	0.717774	-3.09989	-3.03569
H	1.11331	-4.00274	-1.56361
H	-0.23302	-4.54284	-2.59945
C	-3.2002	-2.47088	0.951653
H	-3.60678	-1.66051	1.572649
H	-4.02037	-3.16889	0.715996
H	-2.44642	-3.01452	1.544445
H	-3.3689	-1.39043	-0.89625
H	1.598713	-1.62783	-1.34988
Li	1.272271	0.734444	0.04281
O	1.519002	2.207404	-1.28101
C	0.706182	3.328378	-0.94511
C	1.146347	1.807417	-2.59227
C	-0.63378	3.125932	-1.67733
H	0.606373	3.352692	0.148197
H	1.210188	4.253647	-1.27596
C	-0.37343	1.917845	-2.5971
H	1.607169	2.486519	-3.33474
H	1.519264	0.787709	-2.75461
H	-0.90291	4.024986	-2.25021
H	-1.44788	2.918482	-0.97027
H	-0.78141	2.04788	-3.60908
H	-0.80735	1.003489	-2.16525
O	2.755586	0.634724	1.338062
C	3.962049	0.237563	0.679661
C	2.3931	-0.32202	2.337482
C	4.44948	-1.01246	1.408016
H	3.731024	0.024843	-0.37873
H	4.682815	1.068687	0.713519
C	3.148378	-1.58793	1.967335
H	2.694359	0.054398	3.331647
H	1.299108	-0.43272	2.320689
H	5.13026	-0.73827	2.229301
H	4.983364	-1.70178	0.739349
H	3.296893	-2.25917	2.824383
H	2.597363	-2.12893	1.182544

22

C	-3.69641	-1.41353	0.080824
C	-2.70212	-2.39149	-0.26679
C	-1.61257	-1.69945	-0.77226
C	-1.9037	-0.28663	-0.74993
H	-4.68099	-1.64538	0.49753
C	-3.2365	-0.13967	-0.21422
C	-0.96257	0.733874	-1.06423
O	0.231786	0.555706	-1.36559
O	-1.43503	1.991241	-0.97759
C	-0.51645	3.06048	-1.15593
H	-0.09537	3.024335	-2.17359
H	0.325053	2.947947	-0.45274
C	-1.25764	4.354508	-0.9146

H	-0.5761	5.207184	-1.05117
H	-2.09536	4.466031	-1.61926
H	-1.65886	4.393298	0.109522
N	-0.3556	-2.26588	-1.16141
H	-0.33901	-3.26753	-0.98228
C	-2.82082	-3.87759	-0.08366
H	-2.23293	-4.43605	-0.83189
H	-2.47717	-4.21778	0.910416
H	-3.86634	-4.20943	-0.18636
C	-4.00214	1.132402	0.007028
H	-4.12648	1.714759	-0.92028
H	-5.00547	0.909314	0.403316
H	-3.50141	1.805167	0.723392
H	-0.18486	-2.14441	-2.16089
Li	0.986259	-0.9312	-0.38192
O	0.840148	-0.43513	1.515142
C	1.488178	0.825196	1.723951
C	-0.3697	-0.50526	2.27571
C	0.600126	1.607555	2.69207
H	2.50154	0.653963	2.119818
H	1.575871	1.327246	0.746307
C	-0.76437	0.943464	2.50477
H	-1.10636	-1.08042	1.697835
H	-0.1716	-1.02436	3.230937
H	0.596221	2.684047	2.471555
H	0.950722	1.474745	3.727328
H	-1.27217	1.338717	1.611686
H	-1.43399	1.067293	3.36681
O	2.892708	-0.99006	-0.66361
C	3.789152	-1.48574	0.332637
C	3.527748	0.006179	-1.47329
C	4.917047	-0.46719	0.393188
H	3.231354	-1.59487	1.274256
H	4.160264	-2.47978	0.026989
C	4.995644	-0.00975	-1.06326
H	3.366727	-0.23989	-2.53313
H	3.052398	0.980338	-1.27125
H	5.853978	-0.89965	0.769631
H	4.637433	0.374722	1.046425
H	5.555418	-0.74406	-1.66361
H	5.473615	0.971088	-1.19051

10 NMR spectra

Diisopropyl (1-cyano-2-phenylethyl)phosphonate (P1)

^{1}H NMR

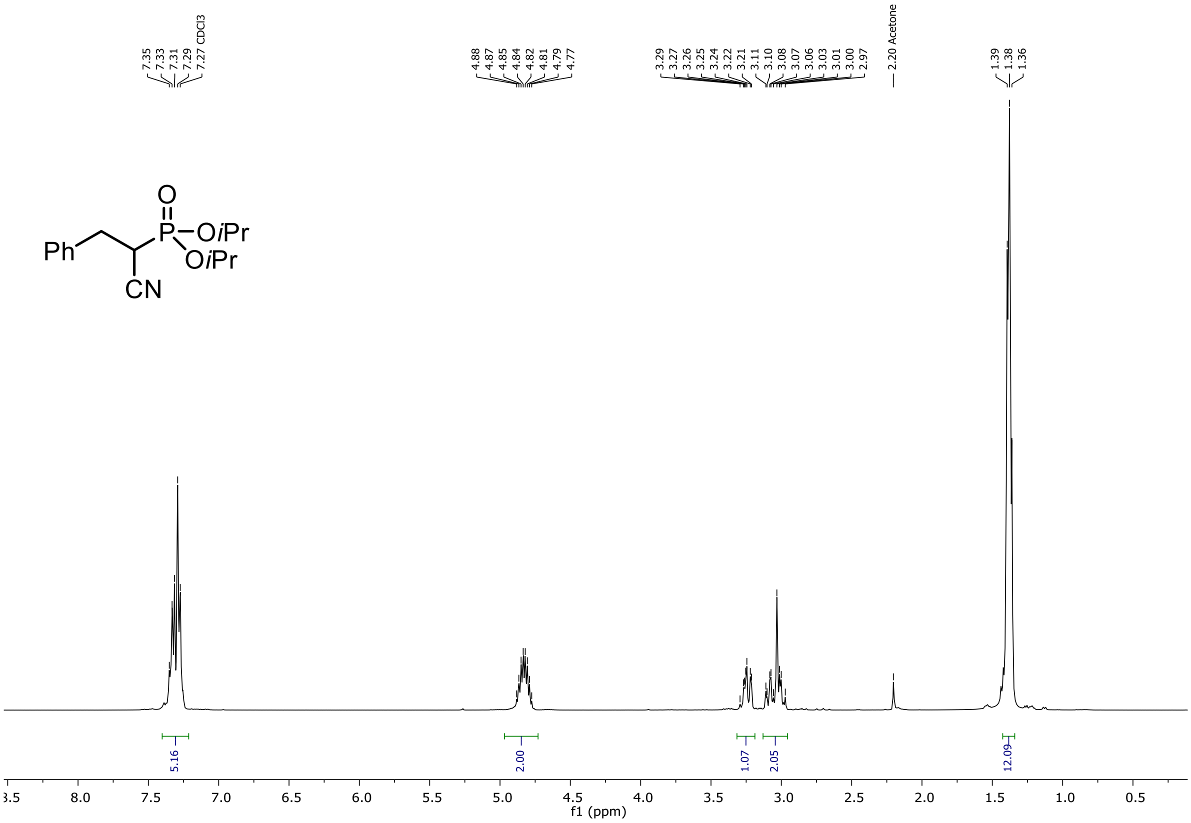

^{13}C NMR

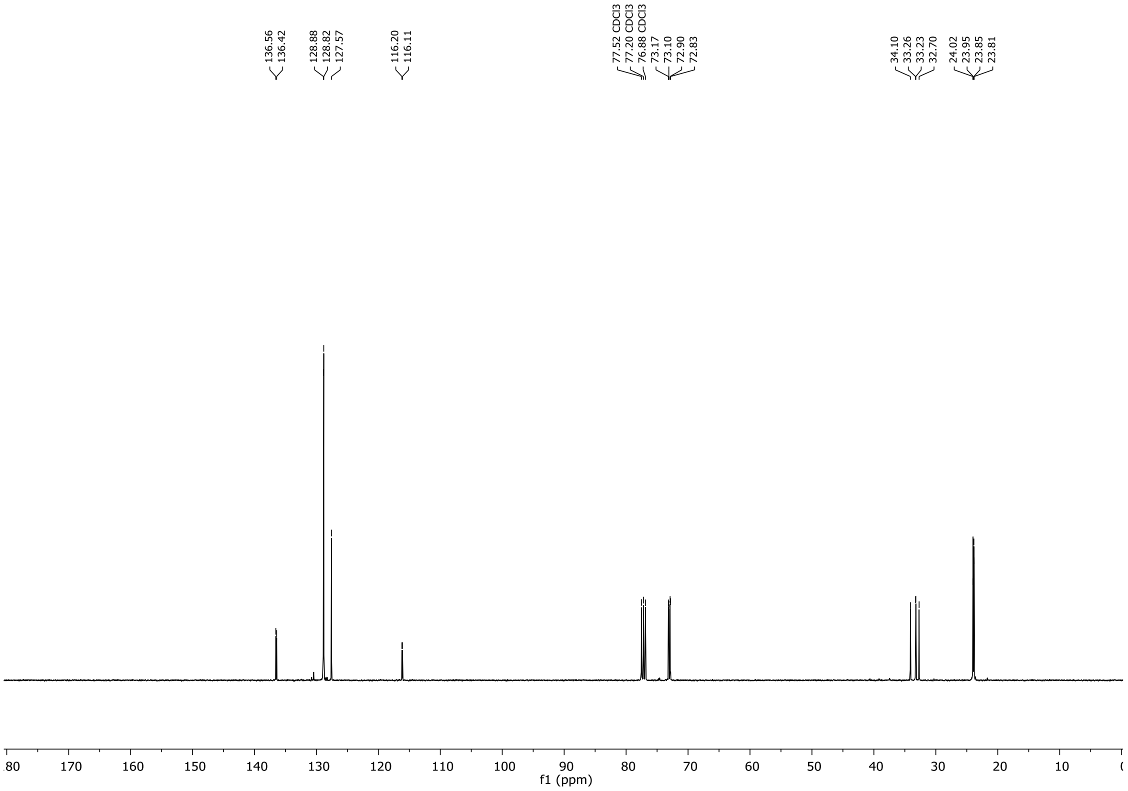

^{31}P NMR

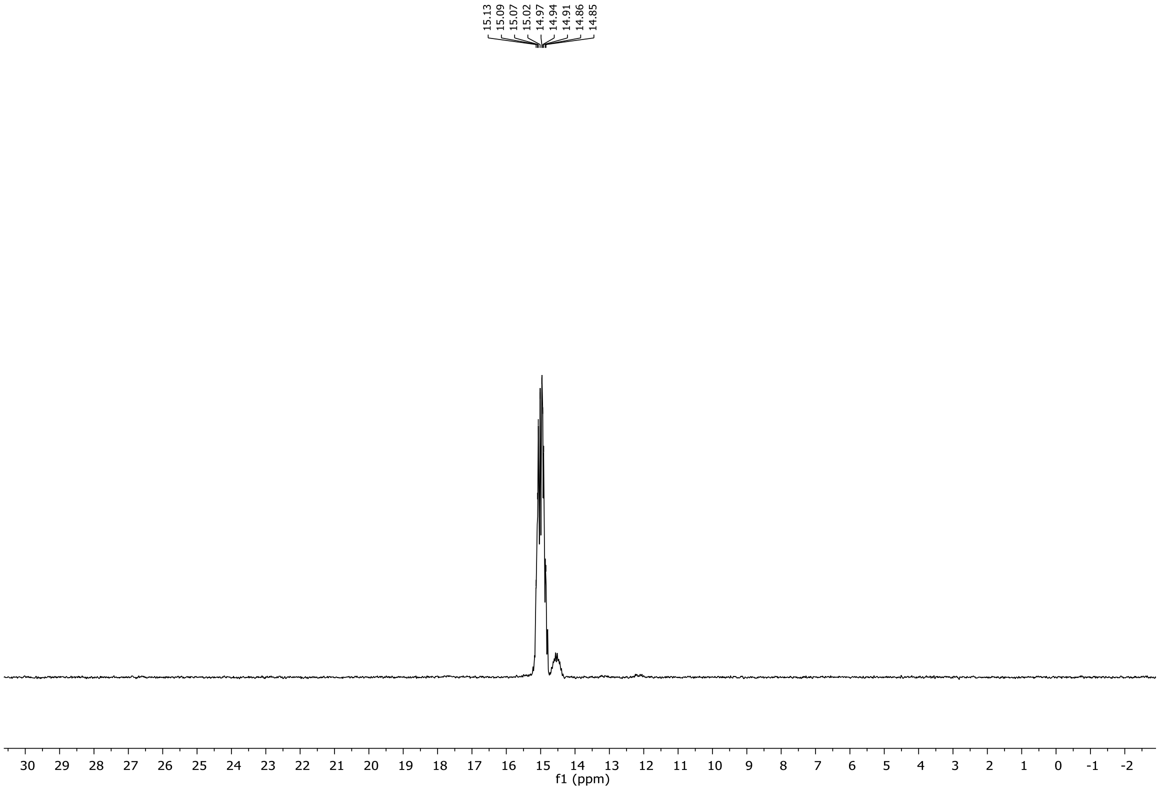

Diisopropyl (2-(2-bromophenyl)-1-cyanoethyl)phosphonate (P2)

^{1}H NMR

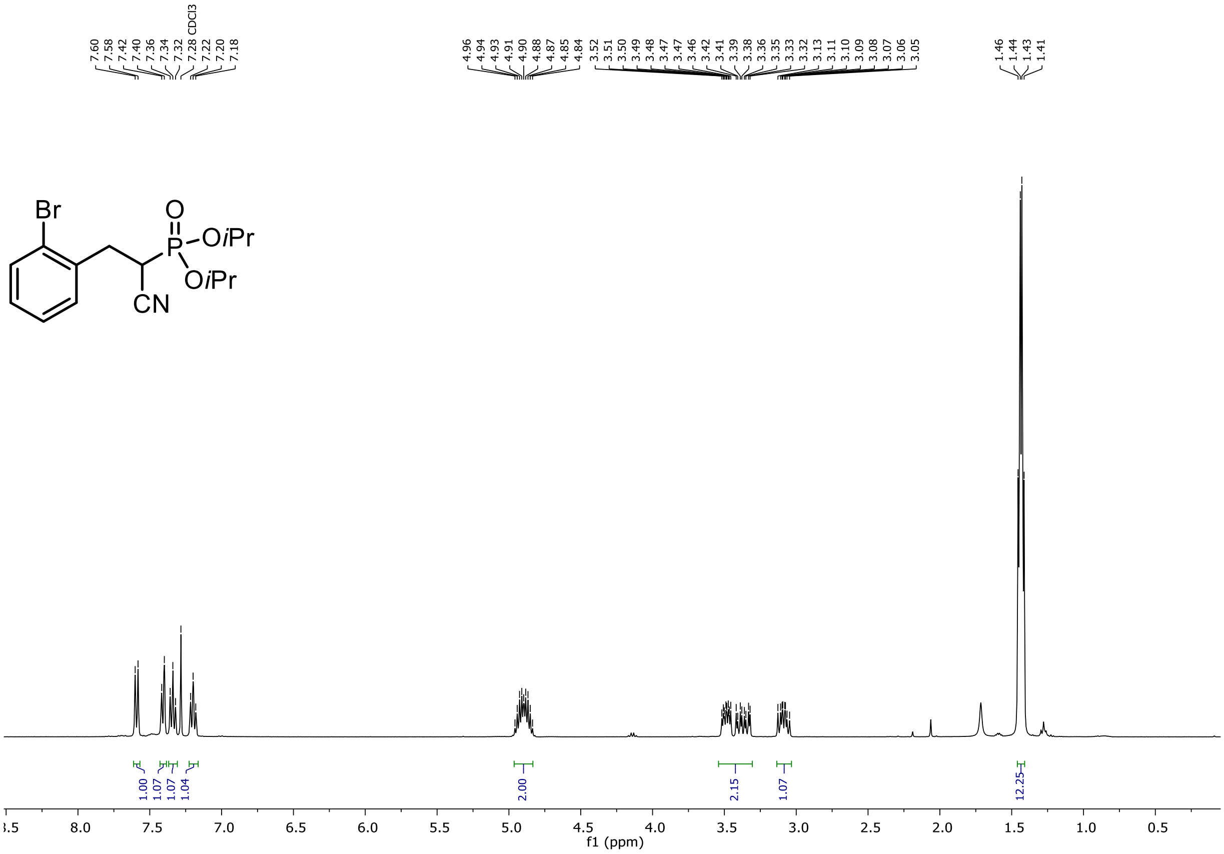

^{13}C NMR

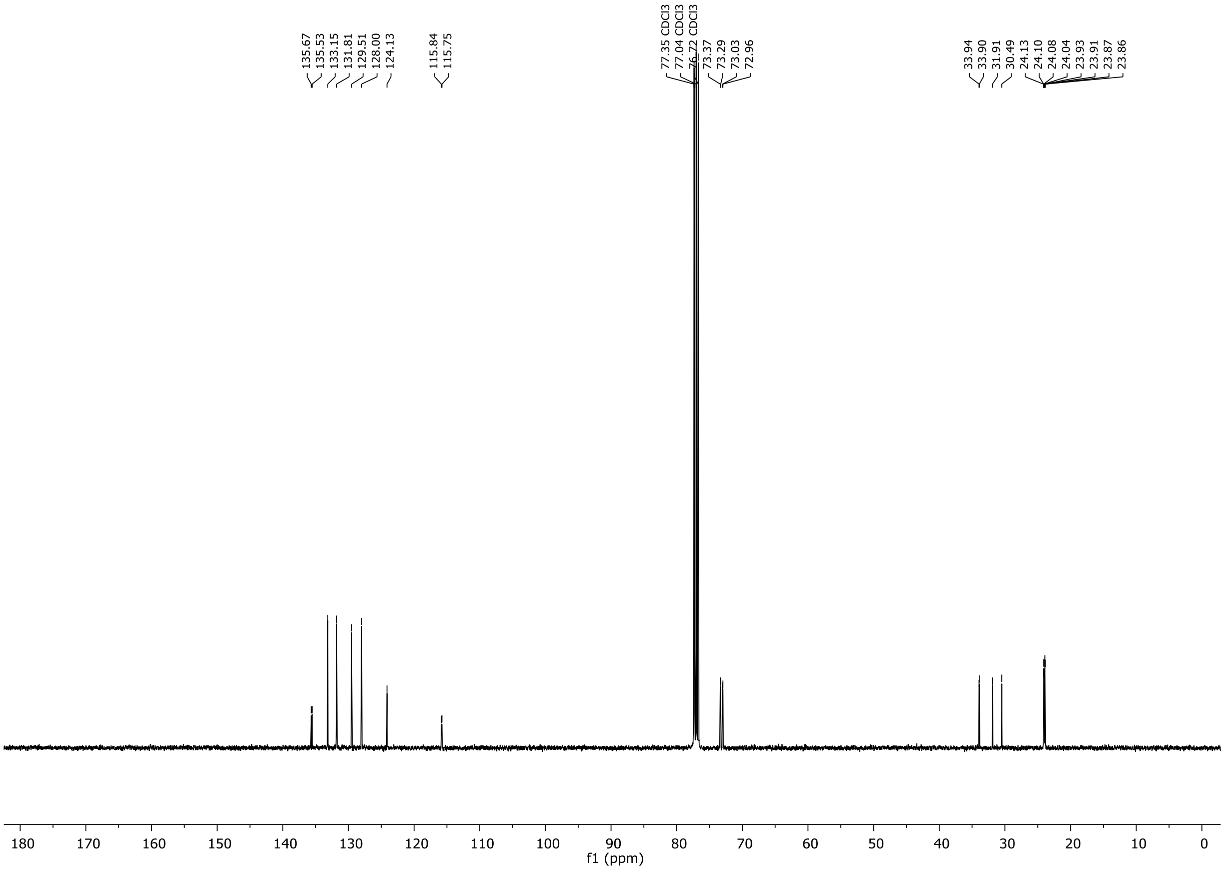

^{31}P NMR

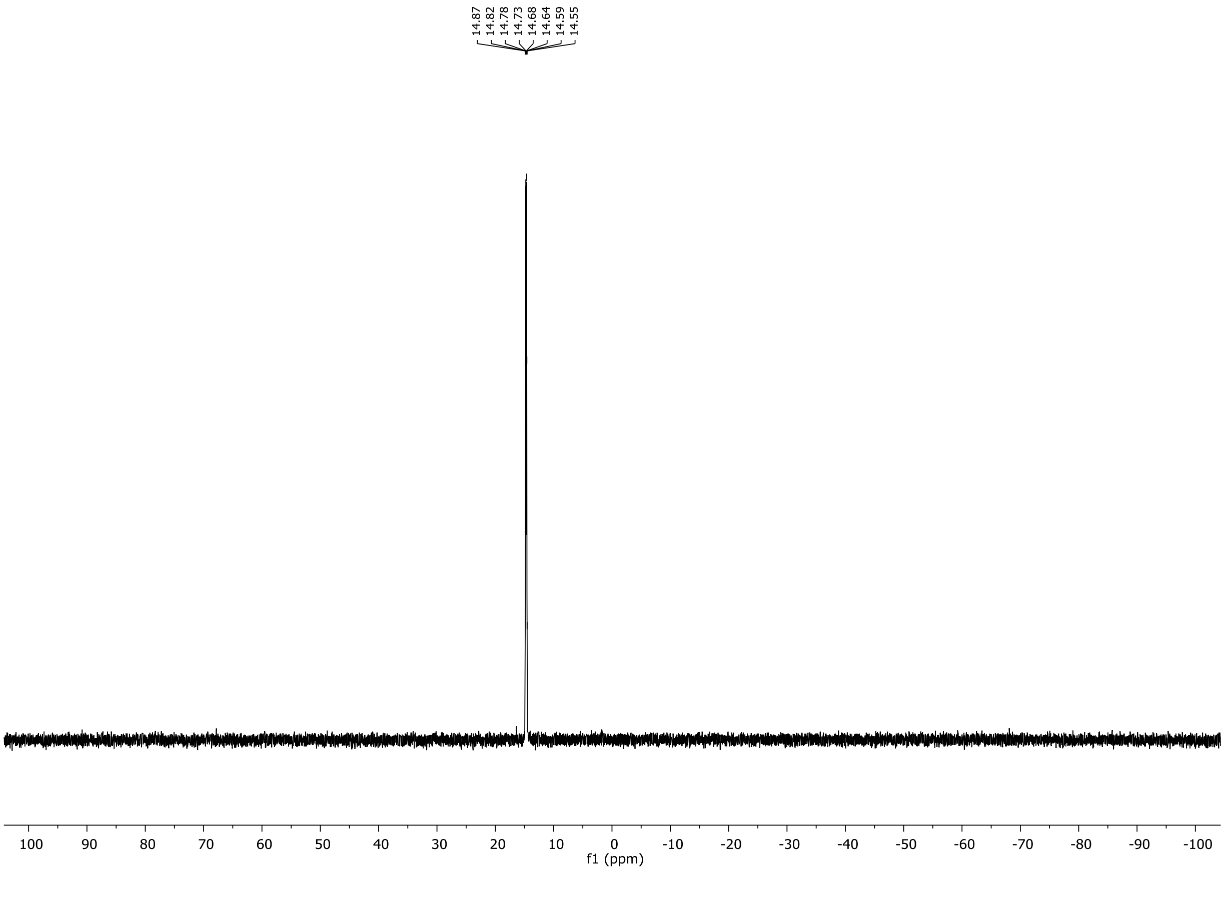

Diethyl (*E*)-(1-cyano-2-(6-(trifluoromethyl)pyridin-3-yl)vinyl)phosphonate (P3)

^{1}H NMR

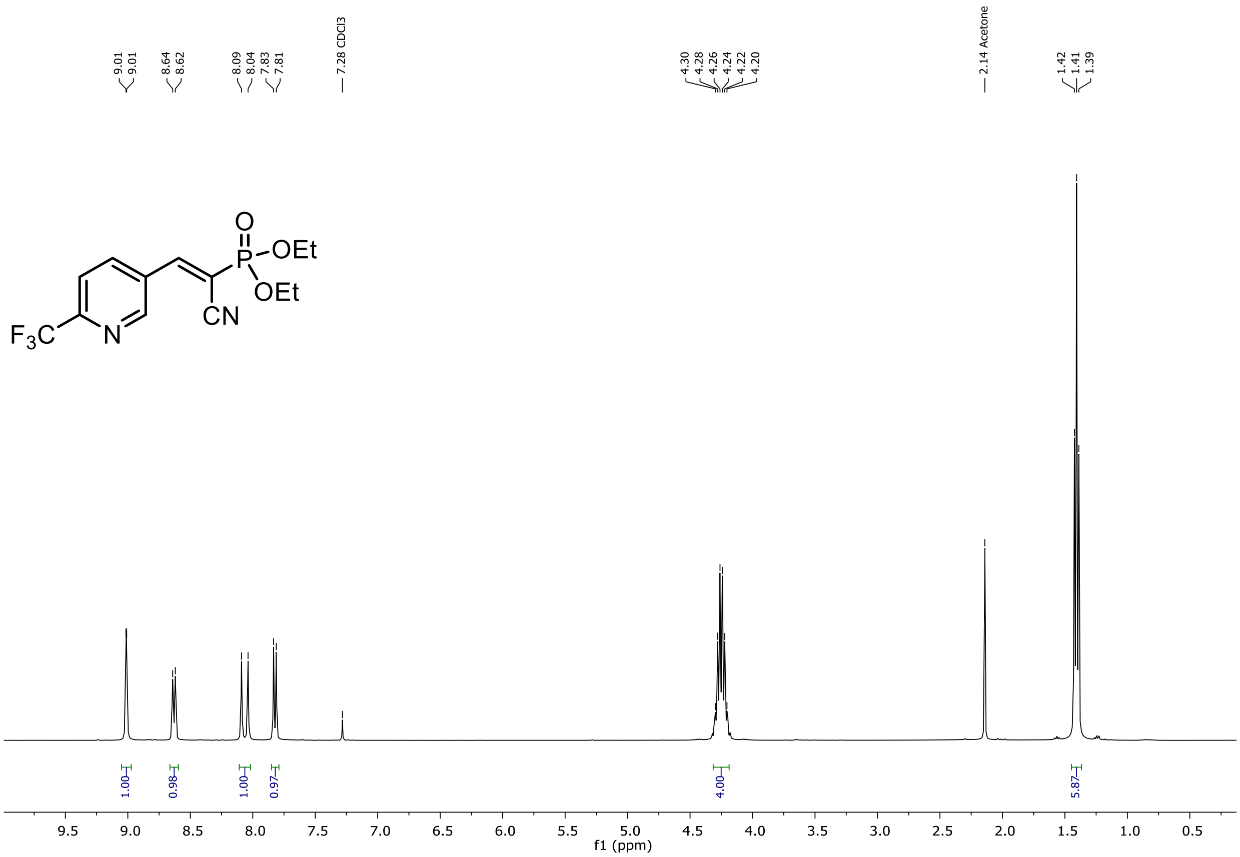

^{13}C NMR

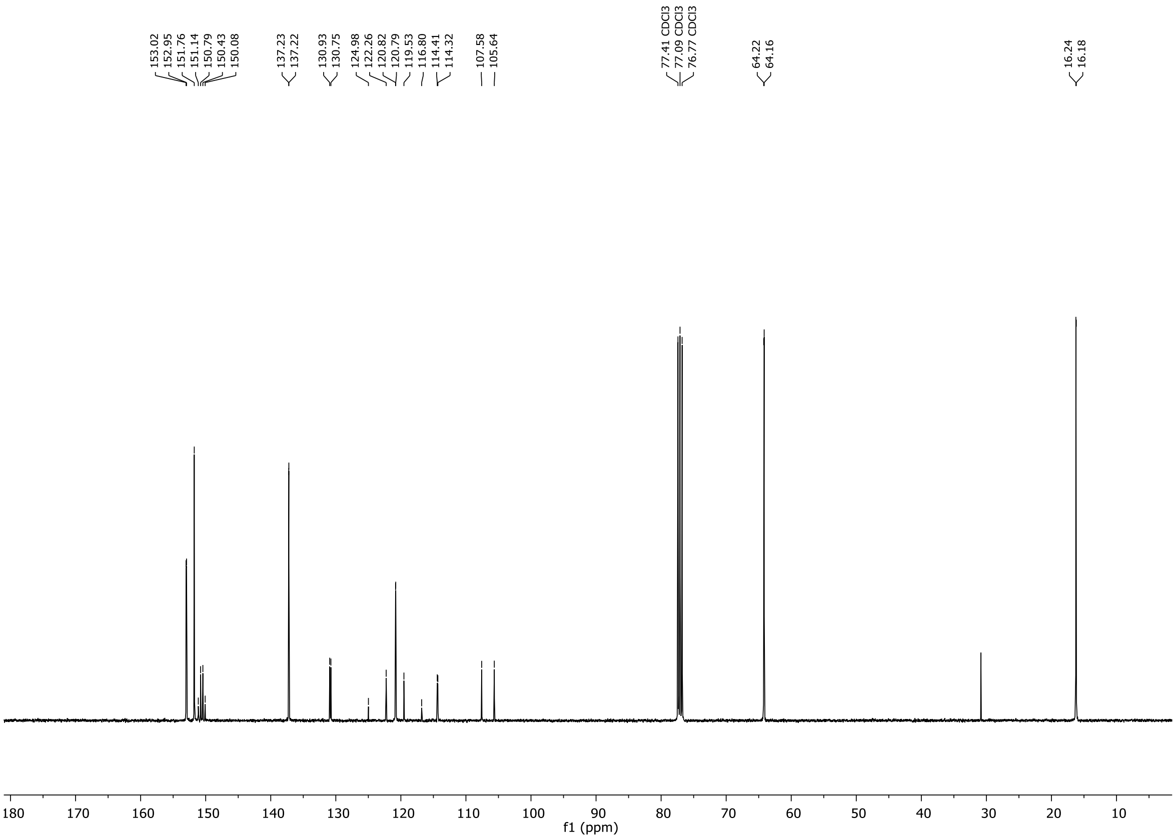

^{31}P NMR

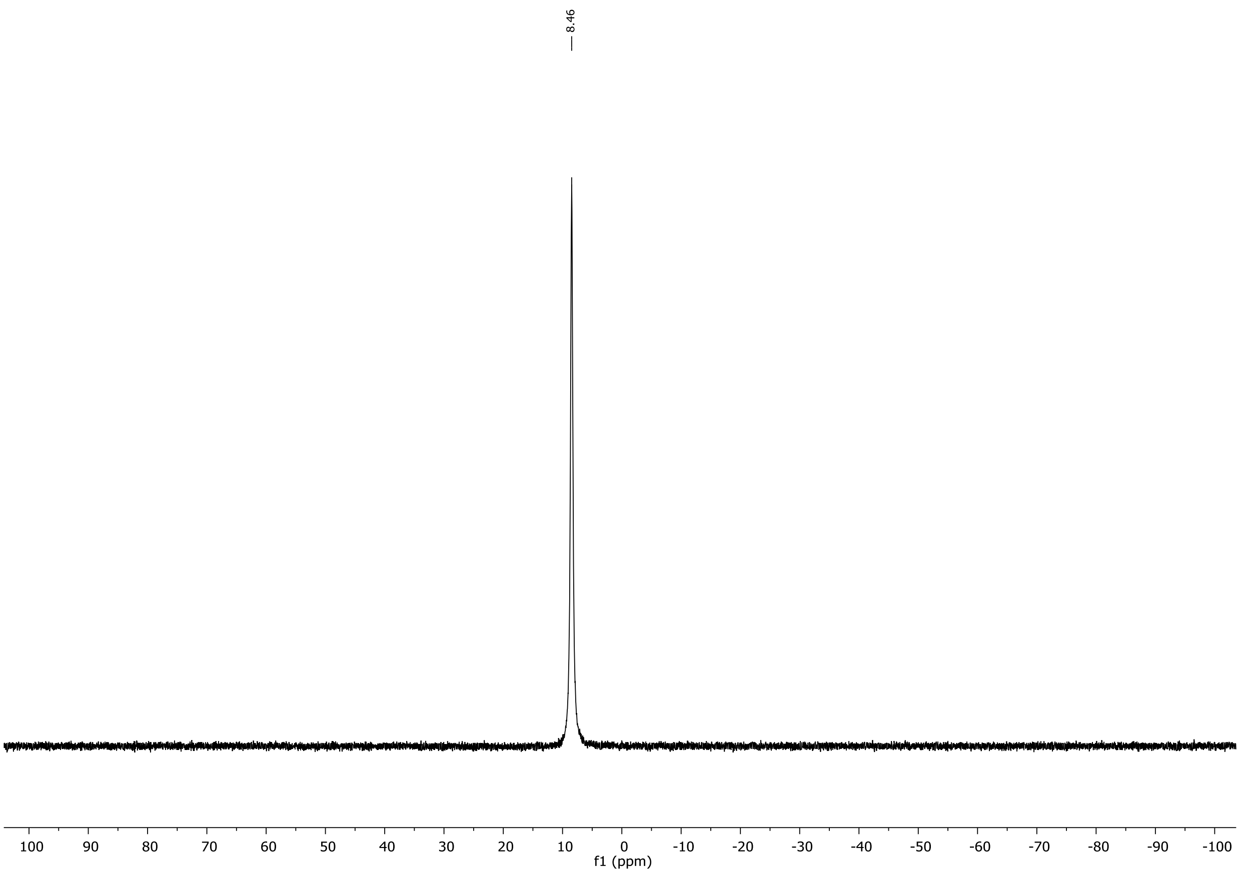

^{19}F NMR

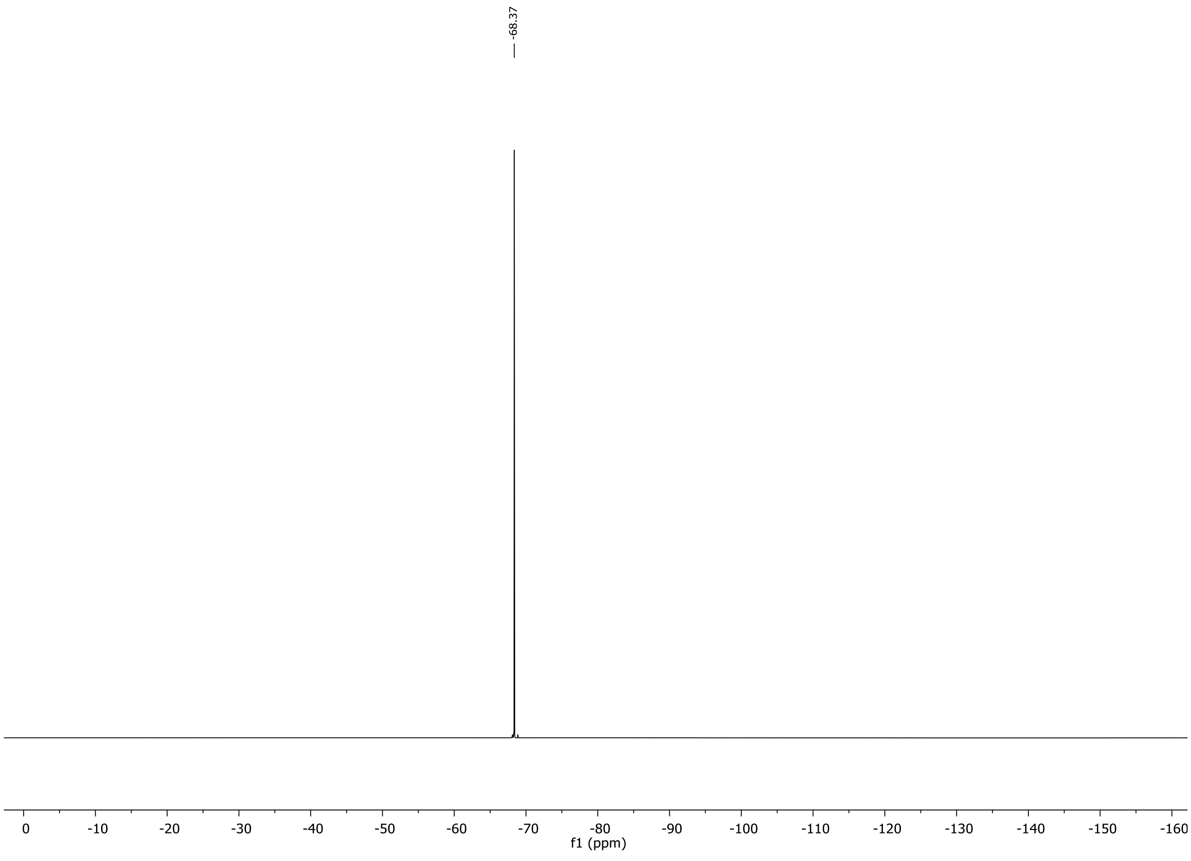

Diisopropyl (*E*)-(1-cyano-2-(thiophen-3-yl)vinyl)phosphonate (P4)

^{1}H NMR

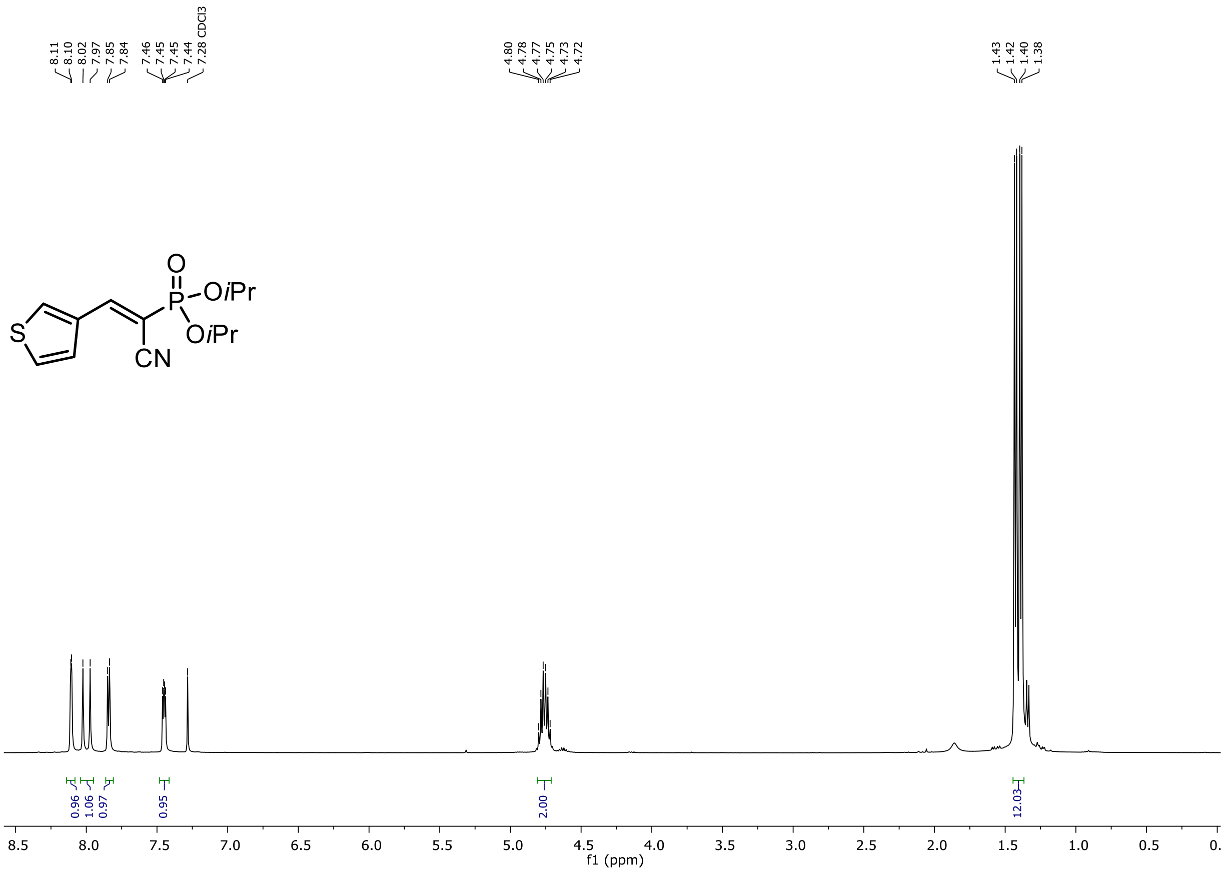

^{13}C NMR

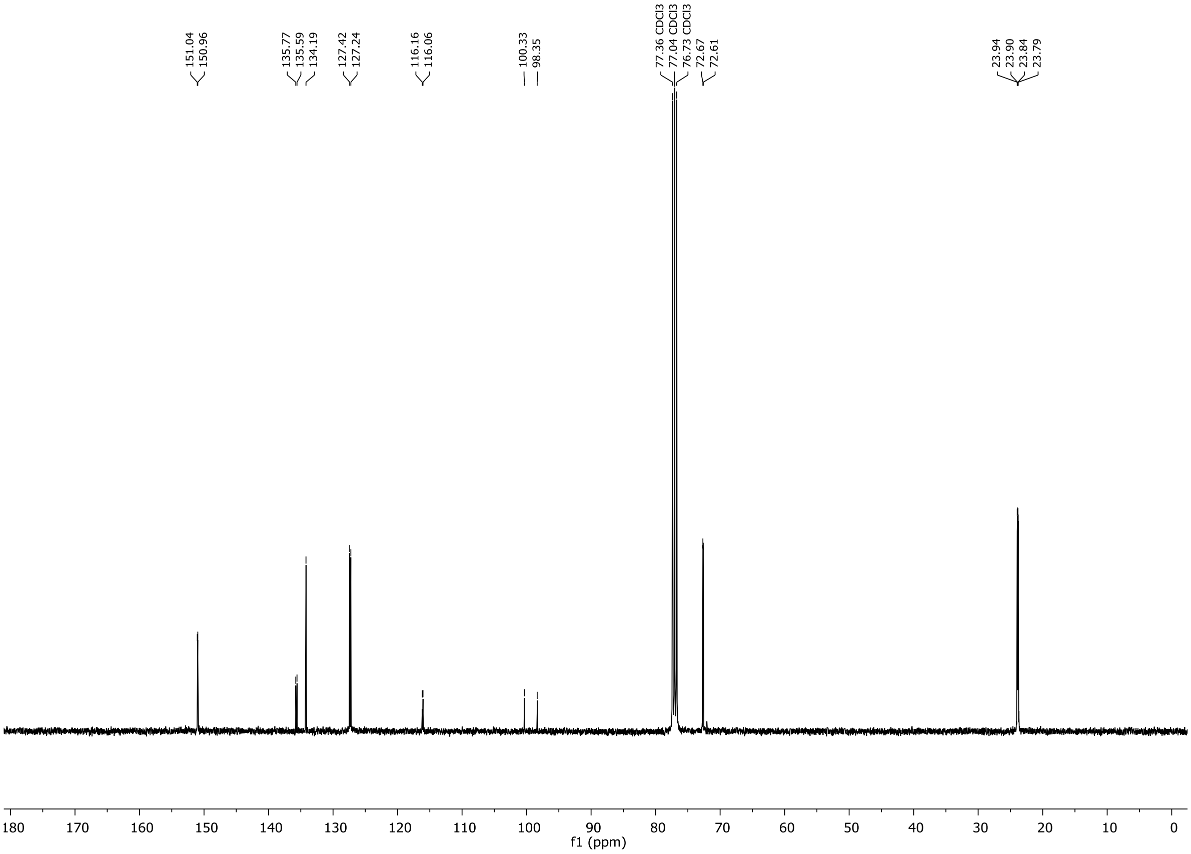

^{31}P NMR

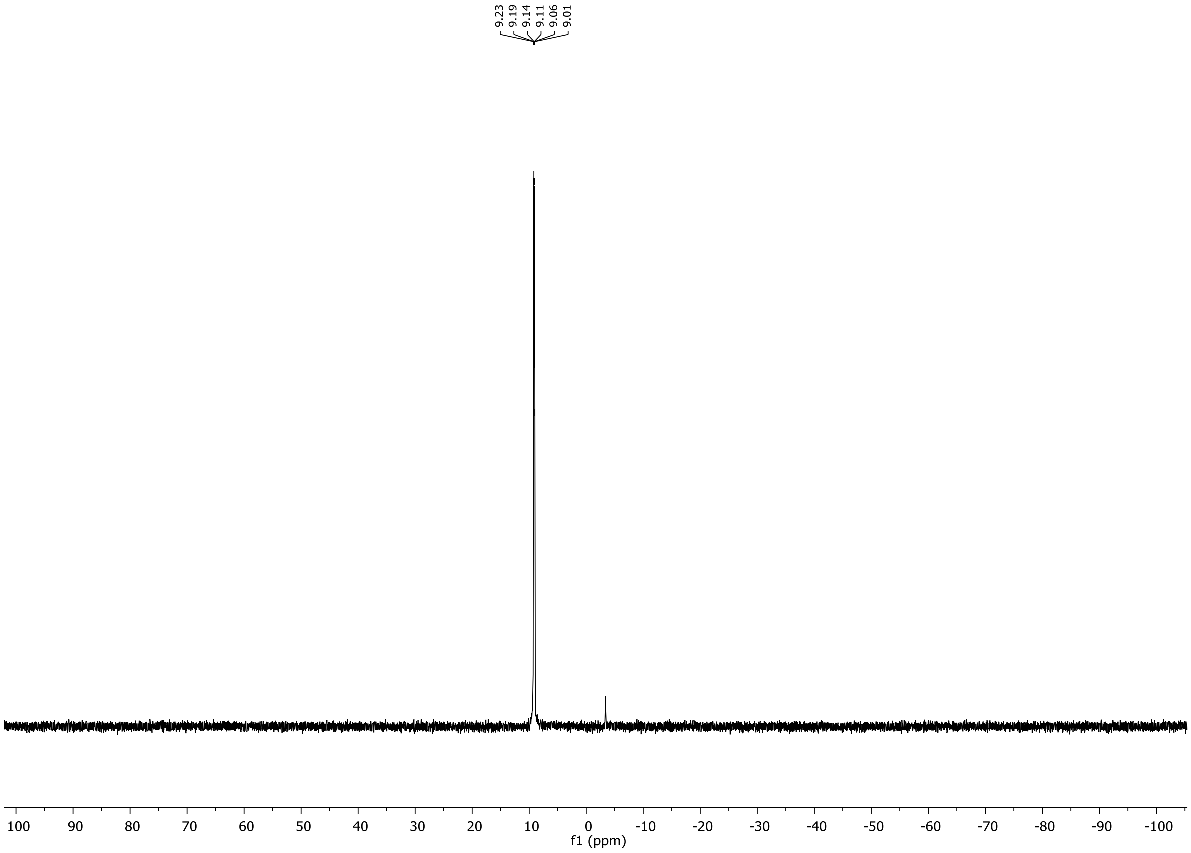

***Tert*-butyl 3-(2-cyano-2-(diisopropoxyphosphoryl)ethyl)-1H-indole-1-carboxylate (P5)**

^{1}H NMR

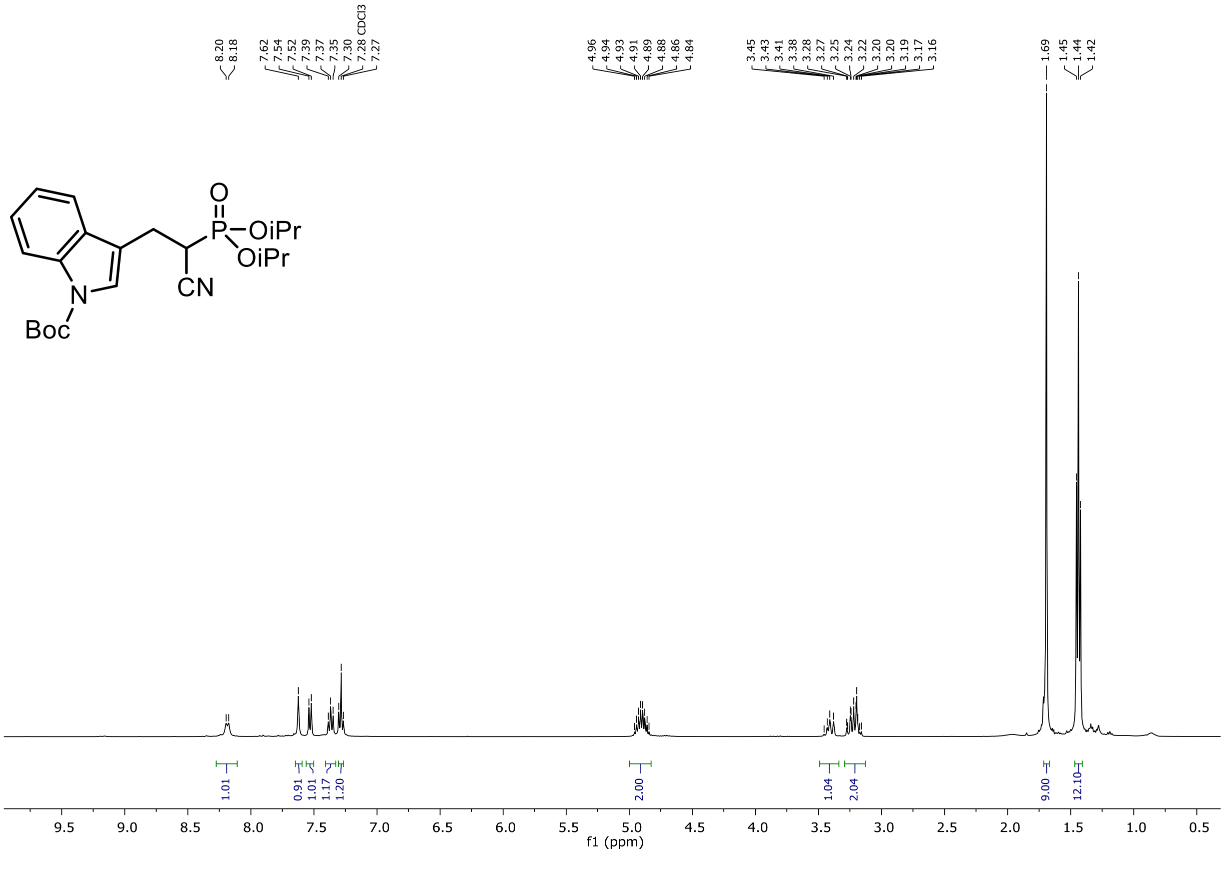

^{13}C NMR

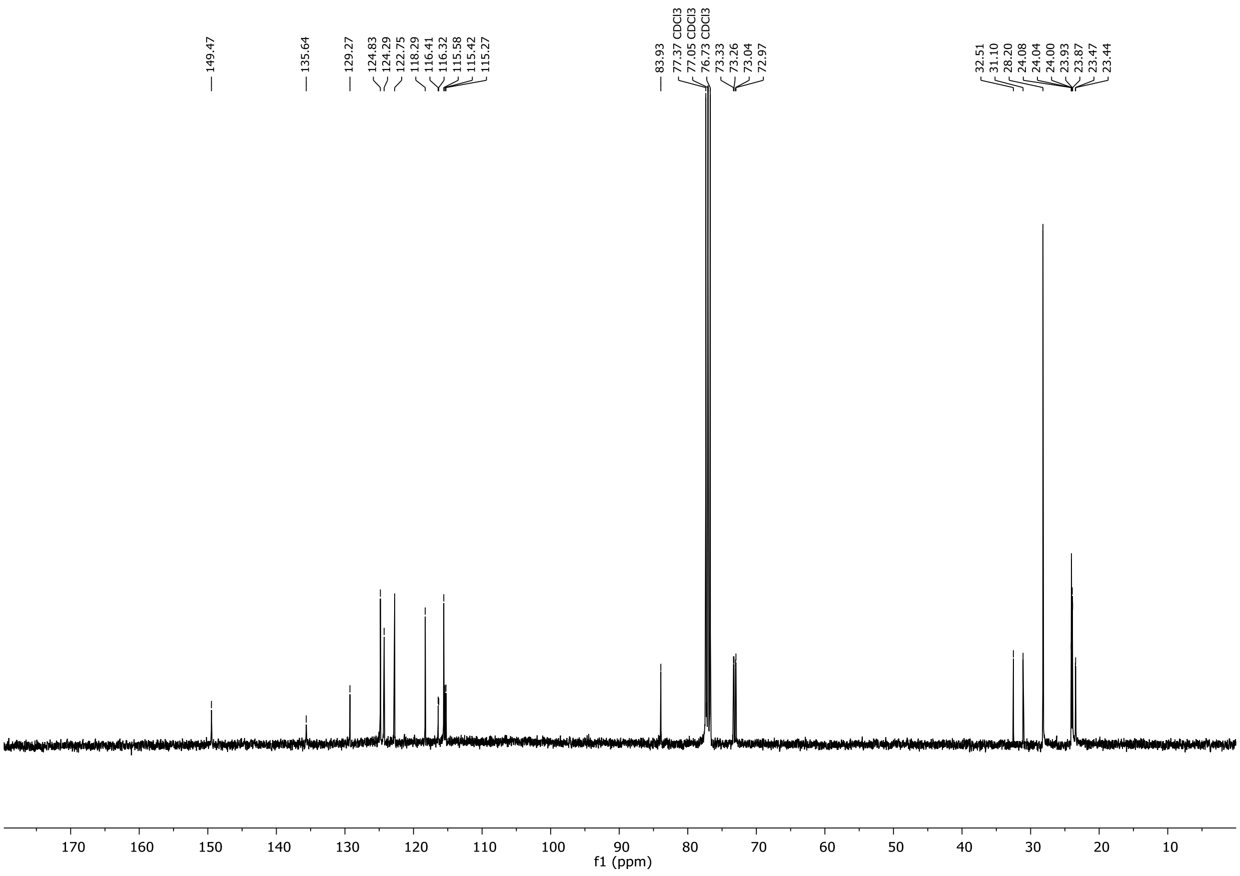

^{31}P NMR

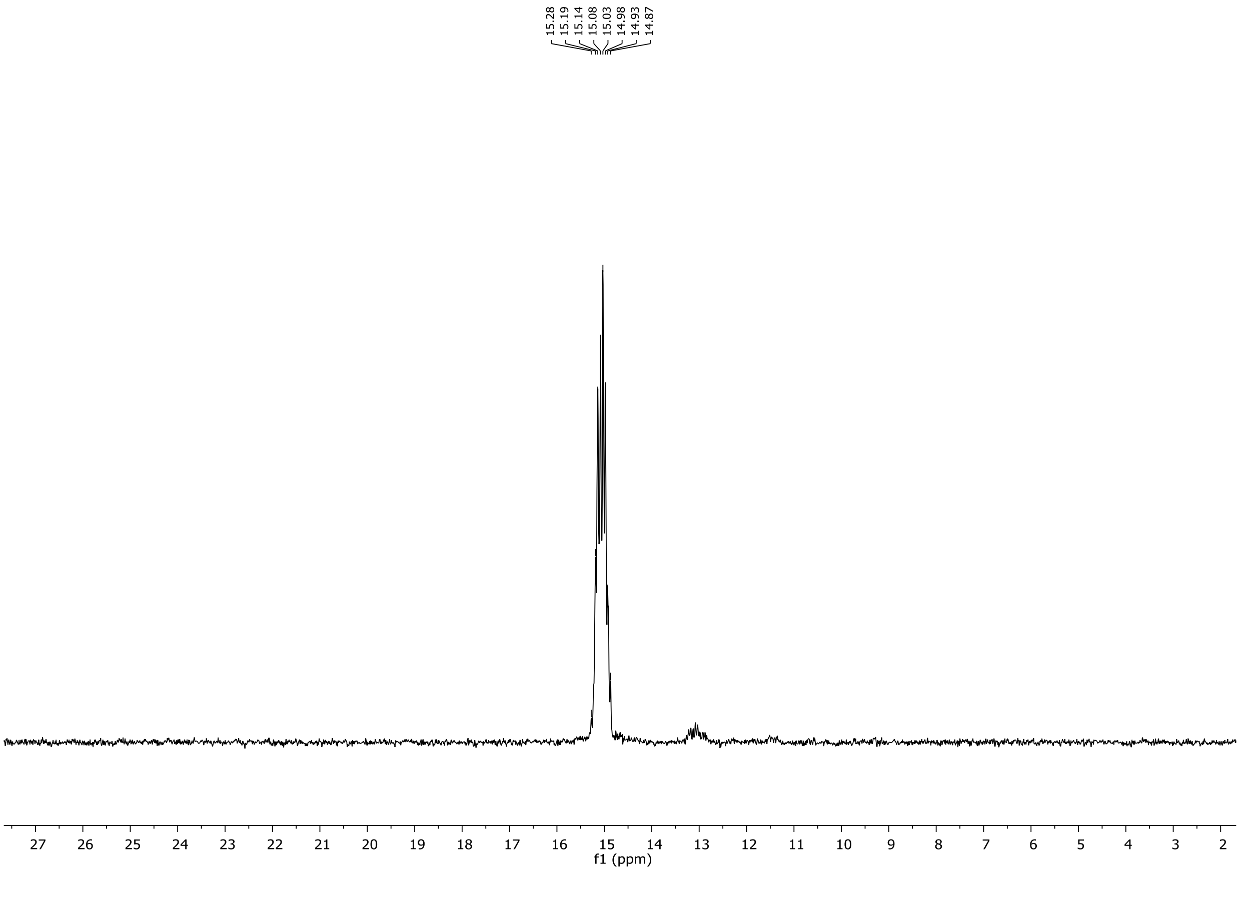

Diisopropyl (1-cyano-2-phenylethyl)phosphonate (P6)

^{1}H NMR

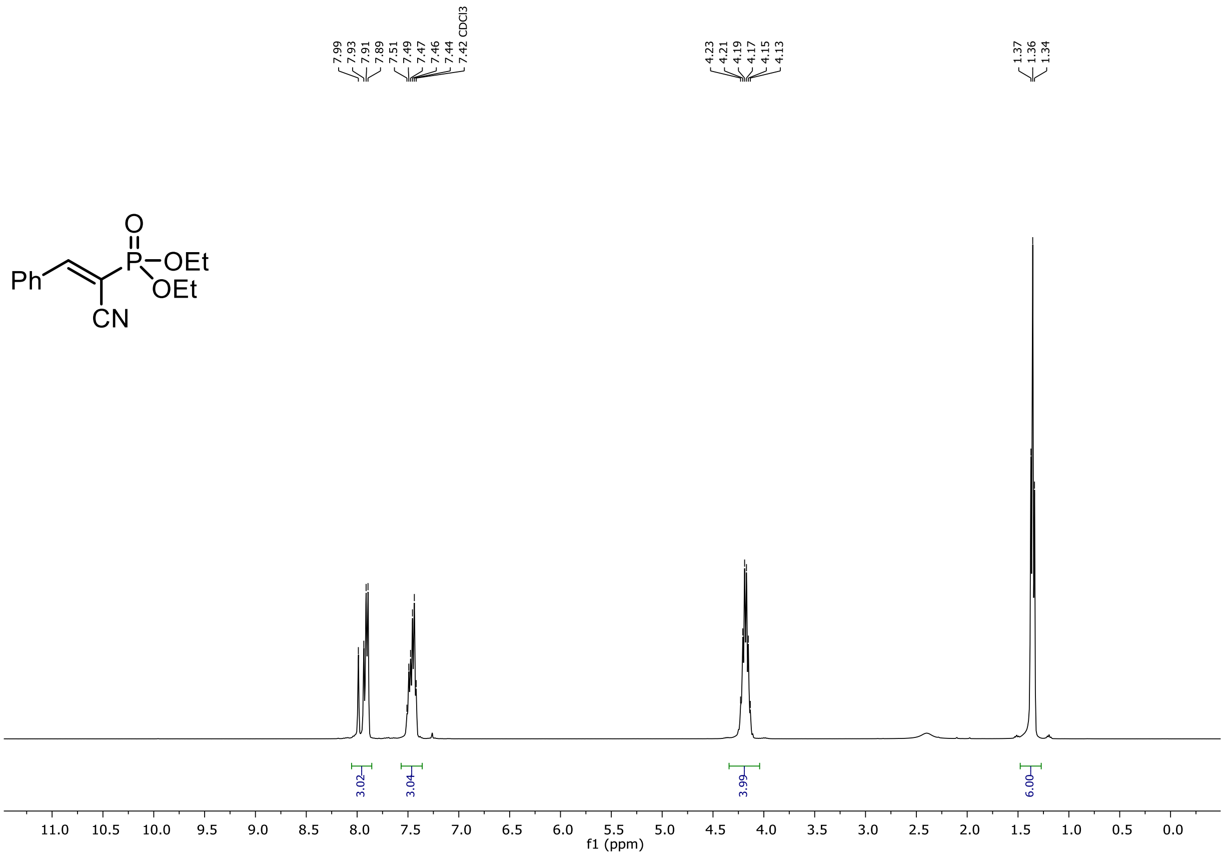

^{13}C NMR

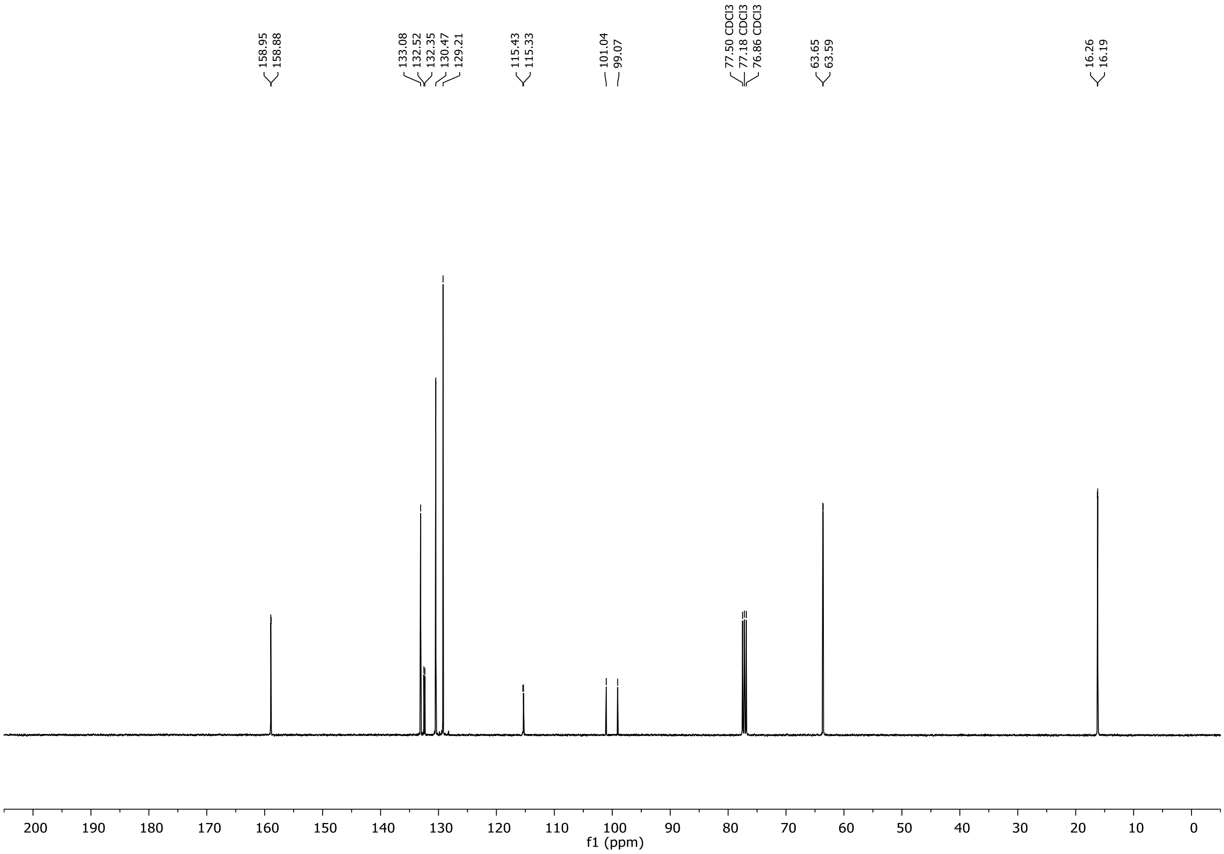

^{31}P NMR

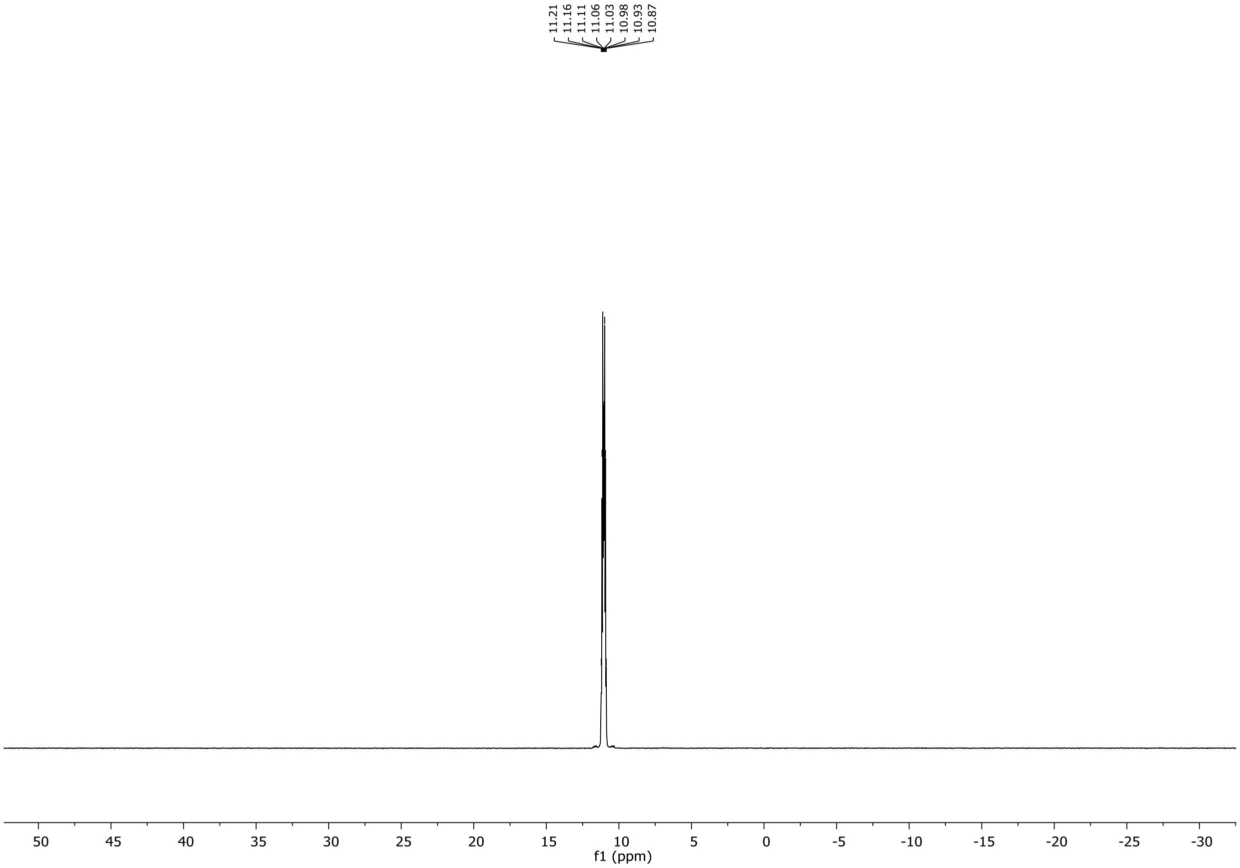

Diethyl (*E*)-(2-(4-bromophenyl)-1-cyanovinyl)phosphonate (P7)

^{1}H NMR

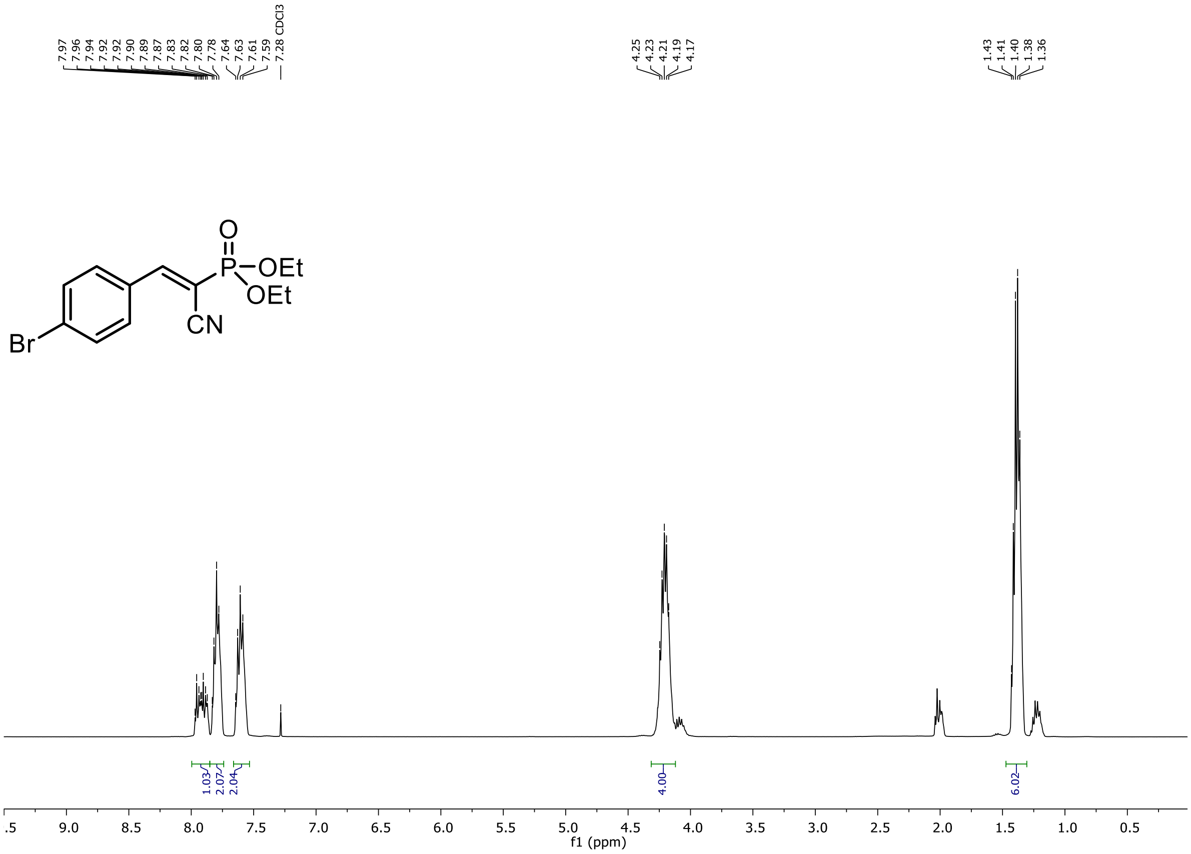

^{13}C NMR

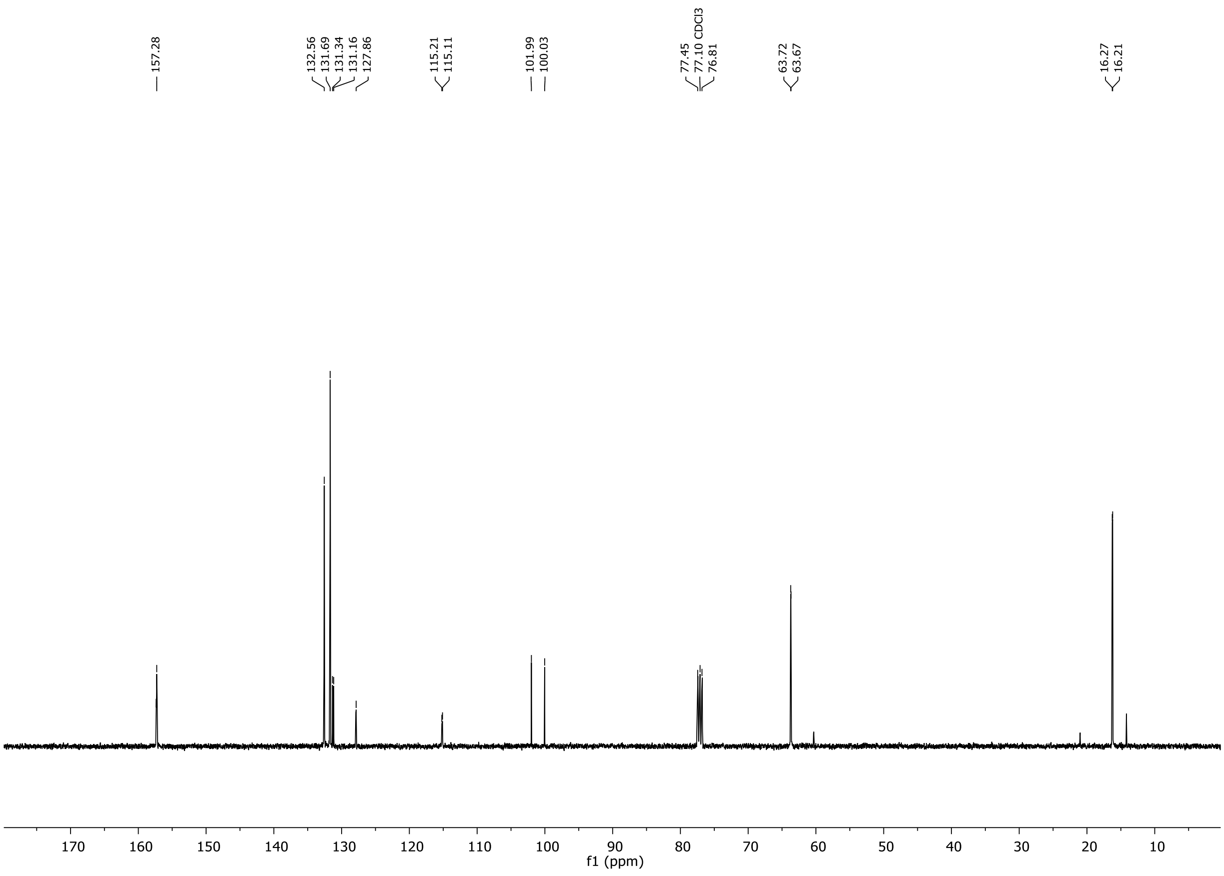

^{31}P NMR

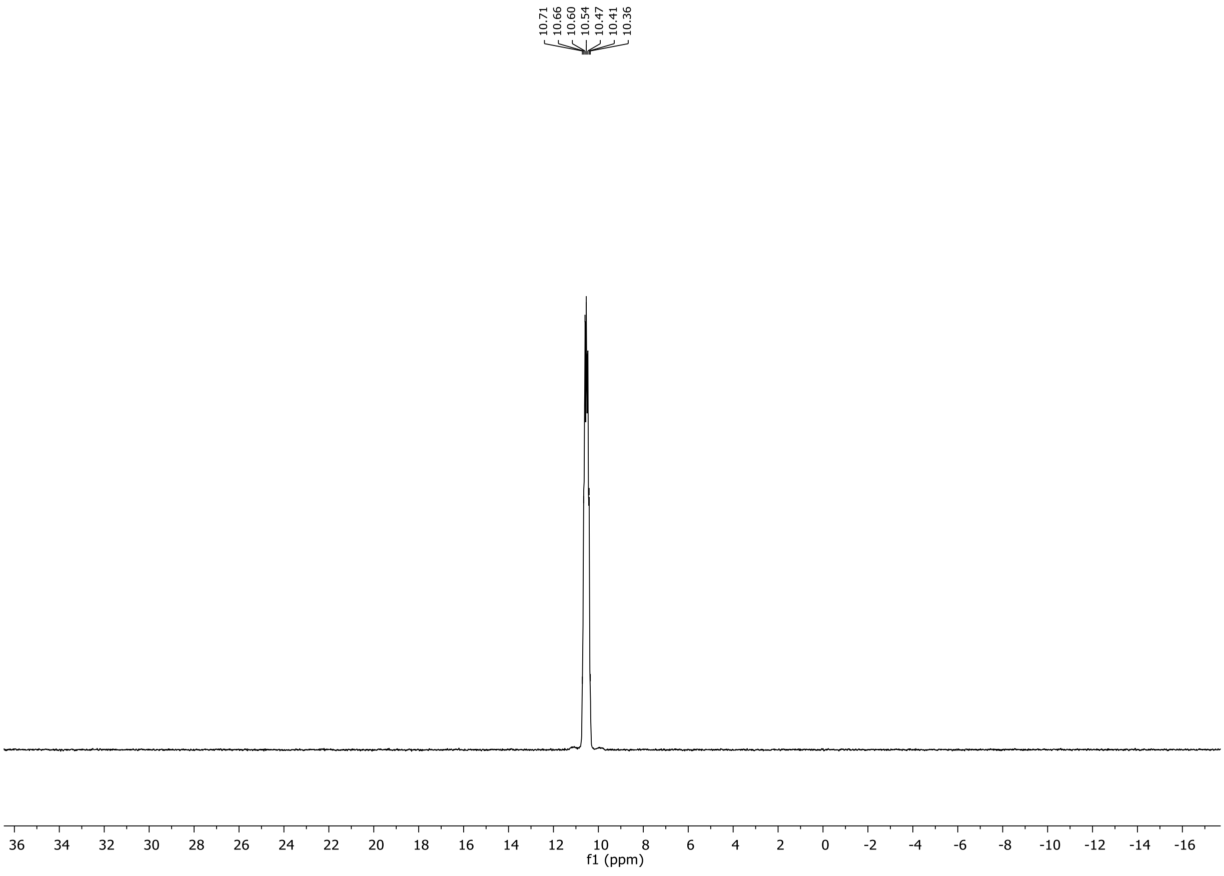

Diethyl (*E*)-(1-cyanoprop-1-en-1-yl)phosphonate (P8)

^{1}H NMR

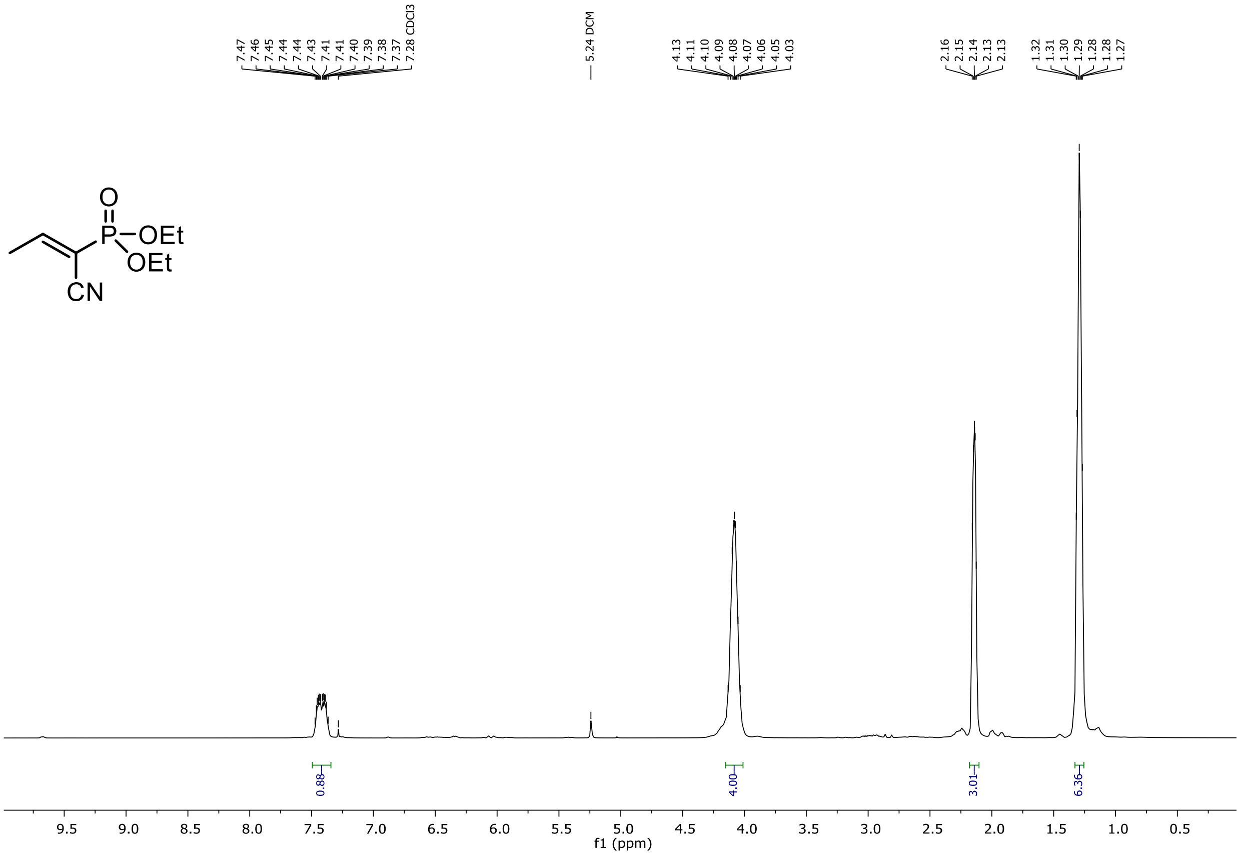

^{13}C NMR

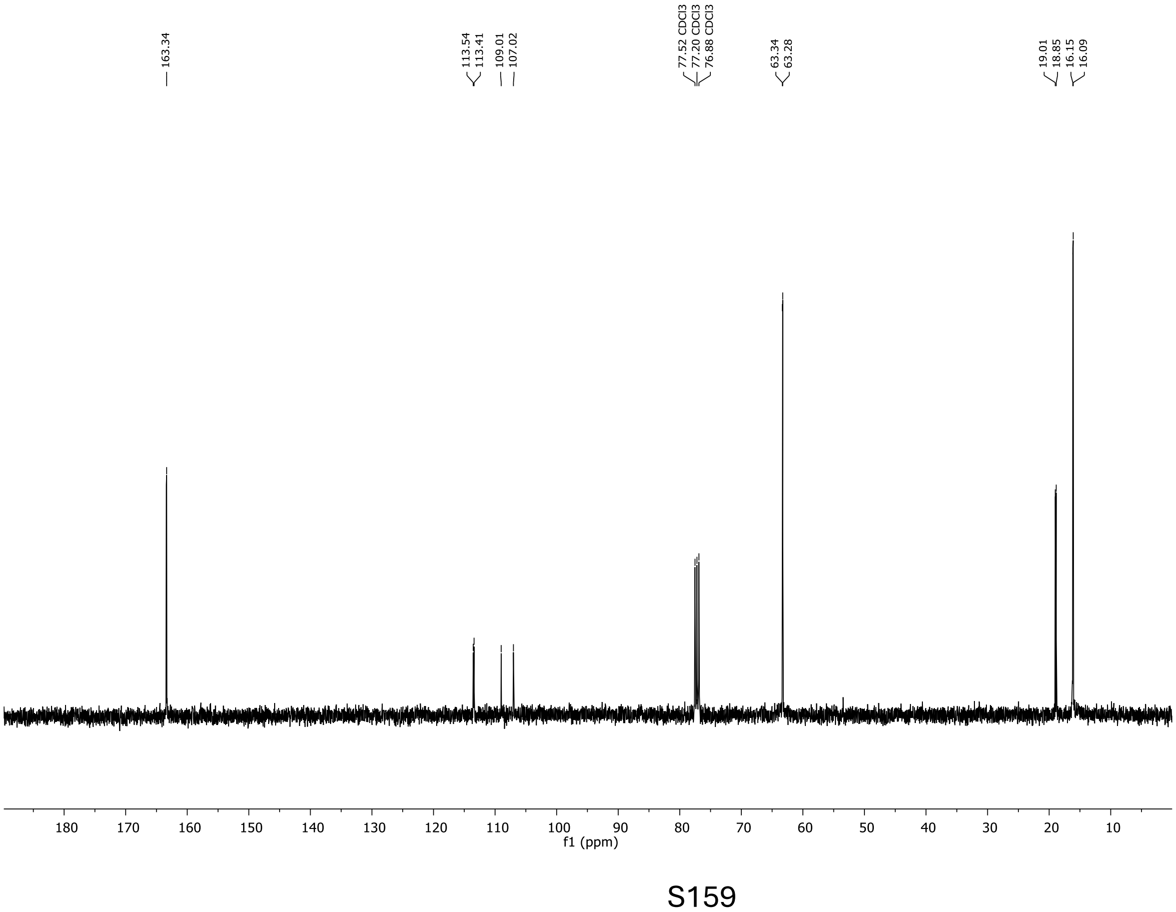

^{31}P NMR

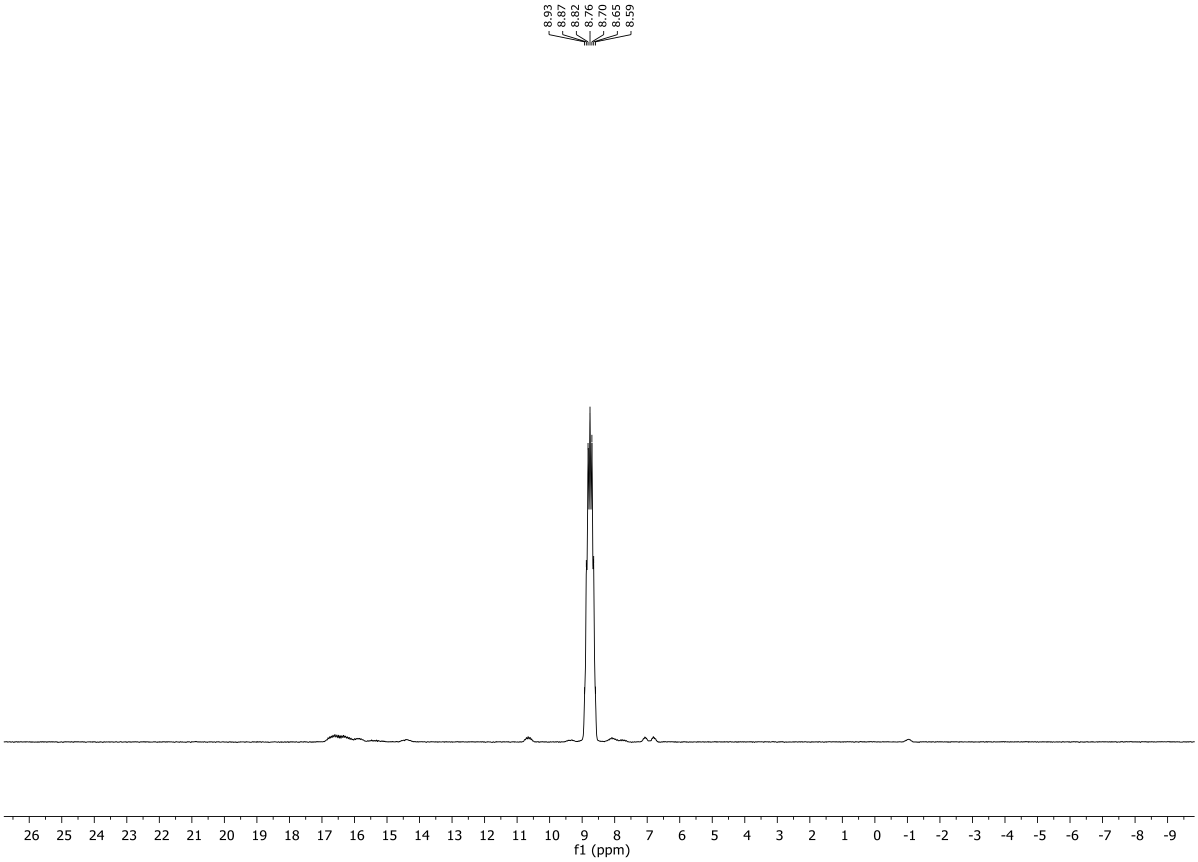

Diisopropyl (1-cyano-4-phenylbutyl)phosphonate (P9)

^{1}H NMR

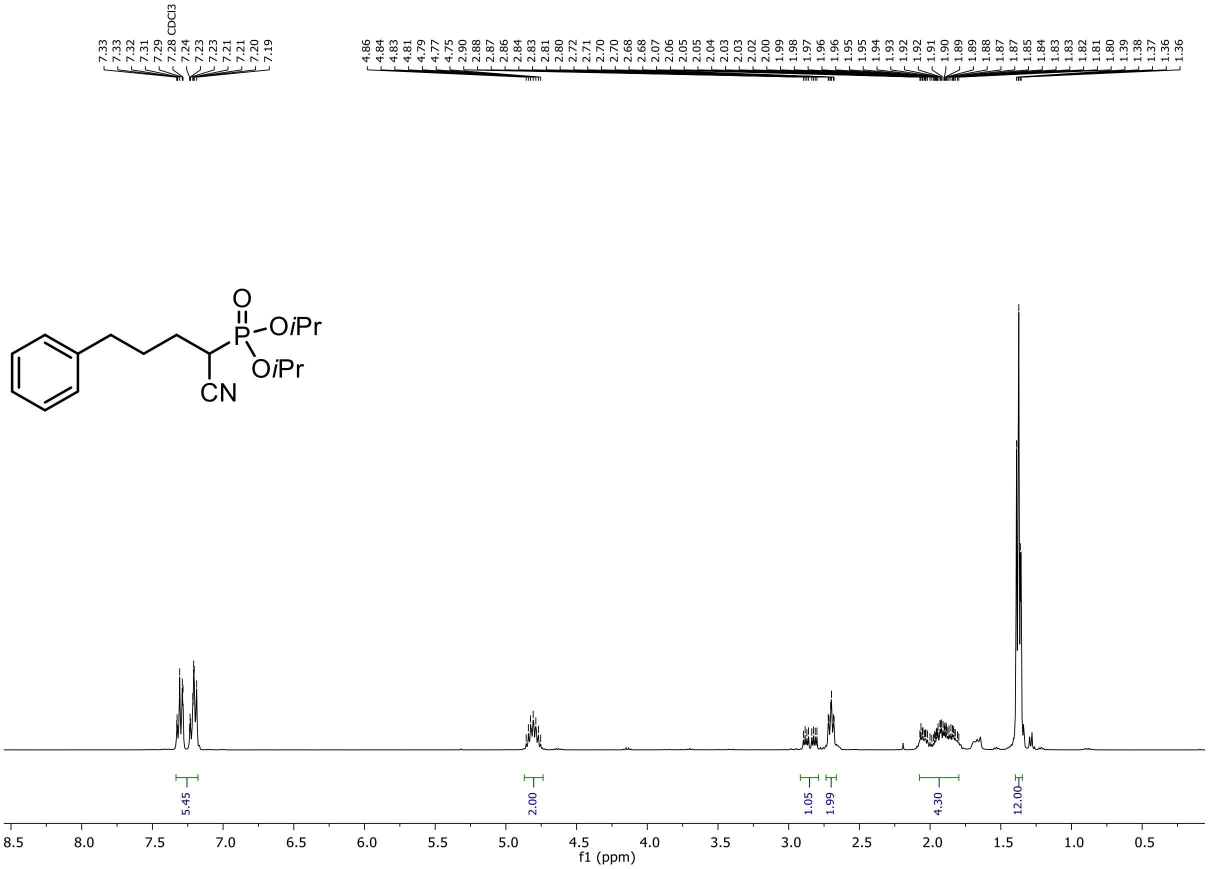

^{13}C NMR

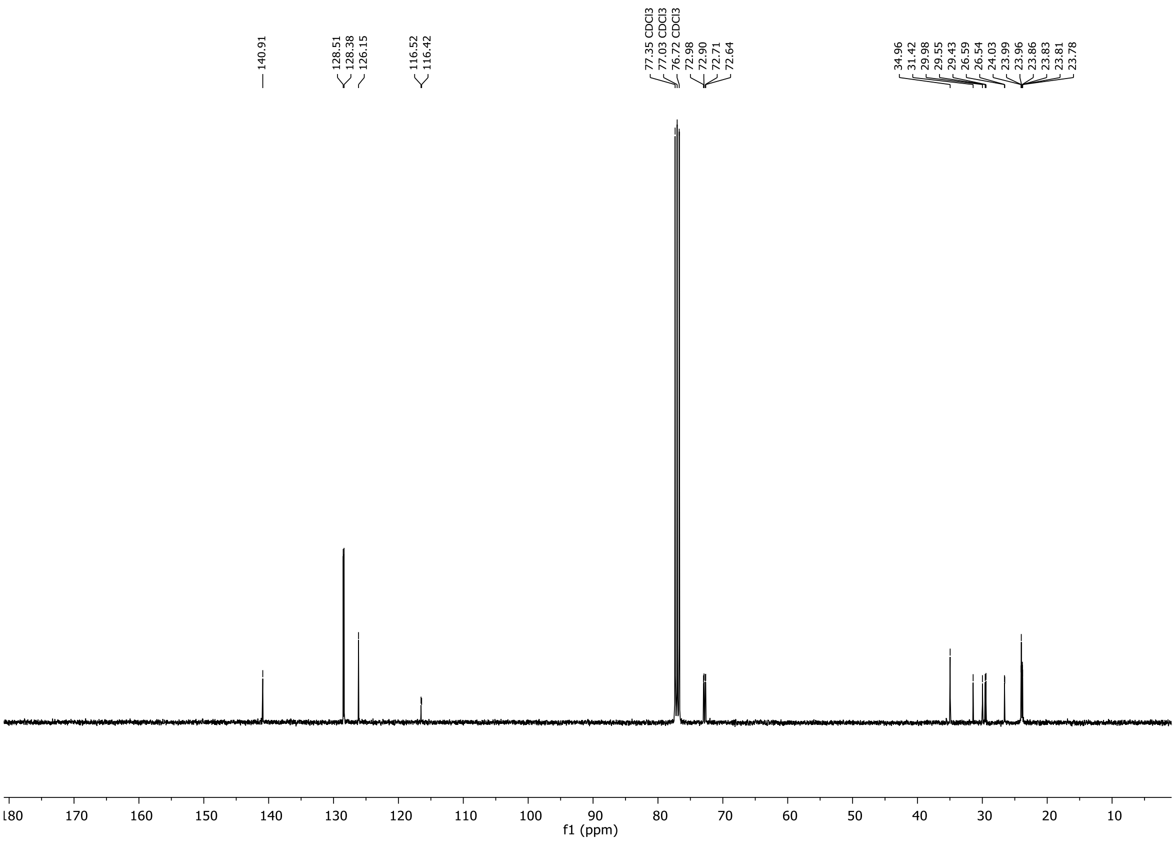

^{31}P NMR

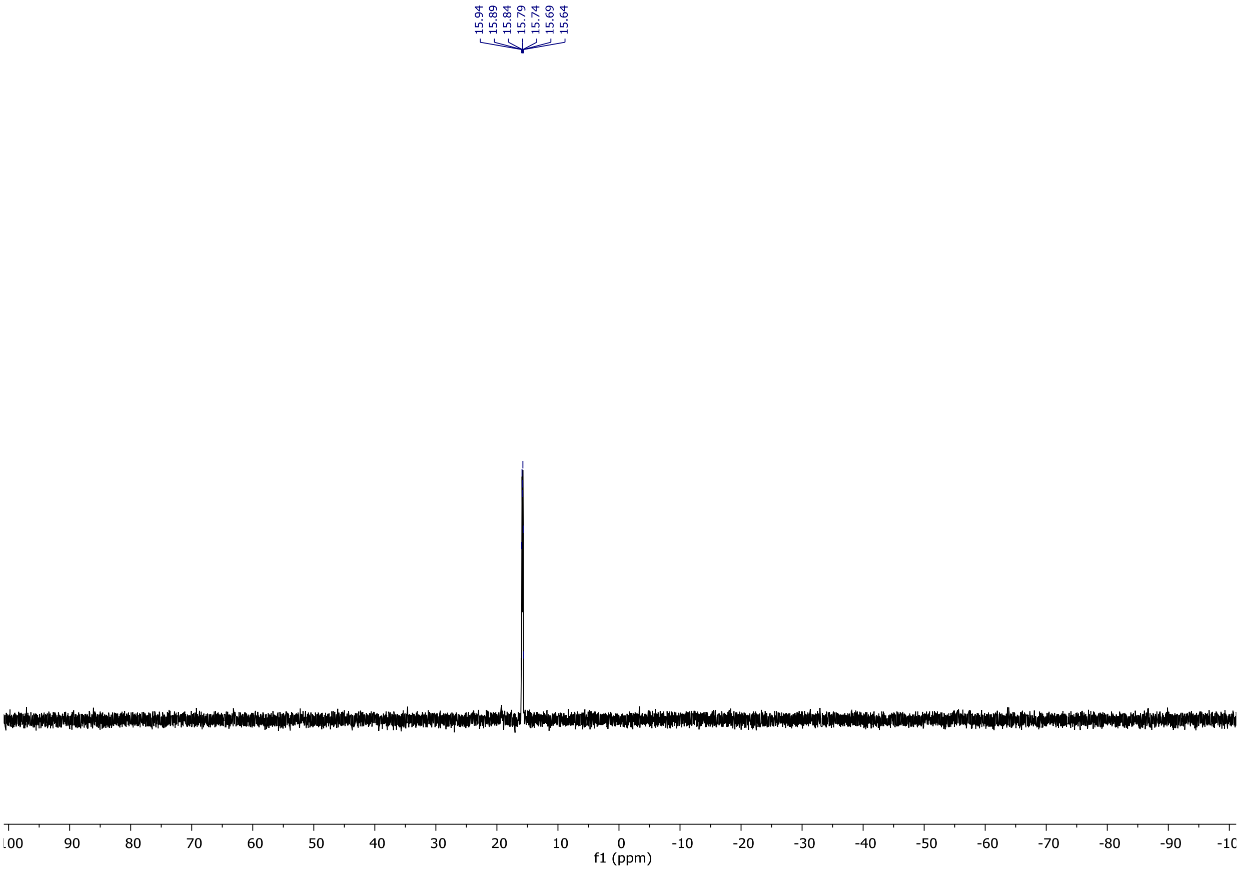

Diisopropyl (1-cyano-3-(1,3-dioxolan-2-yl)propyl)phosphonate (P10)

^{1}H NMR

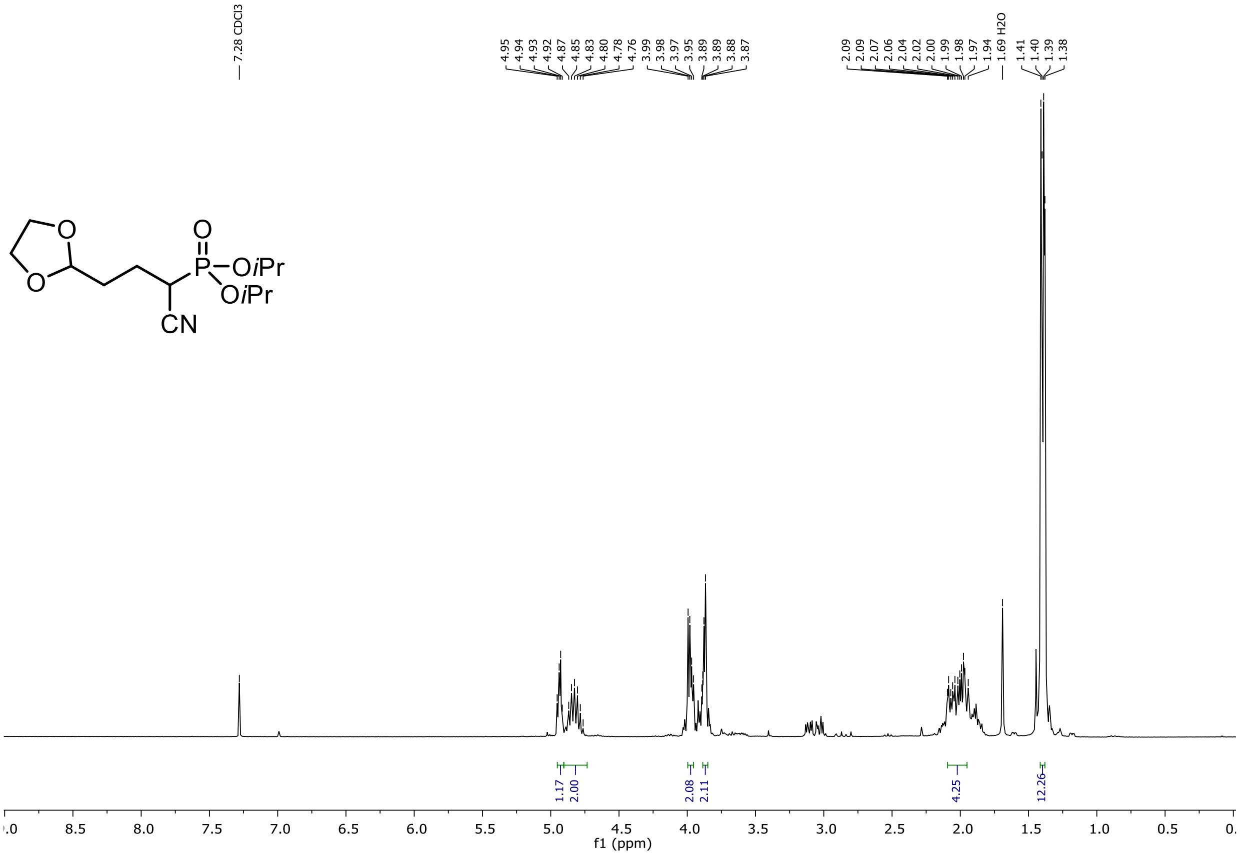

^{13}C NMR

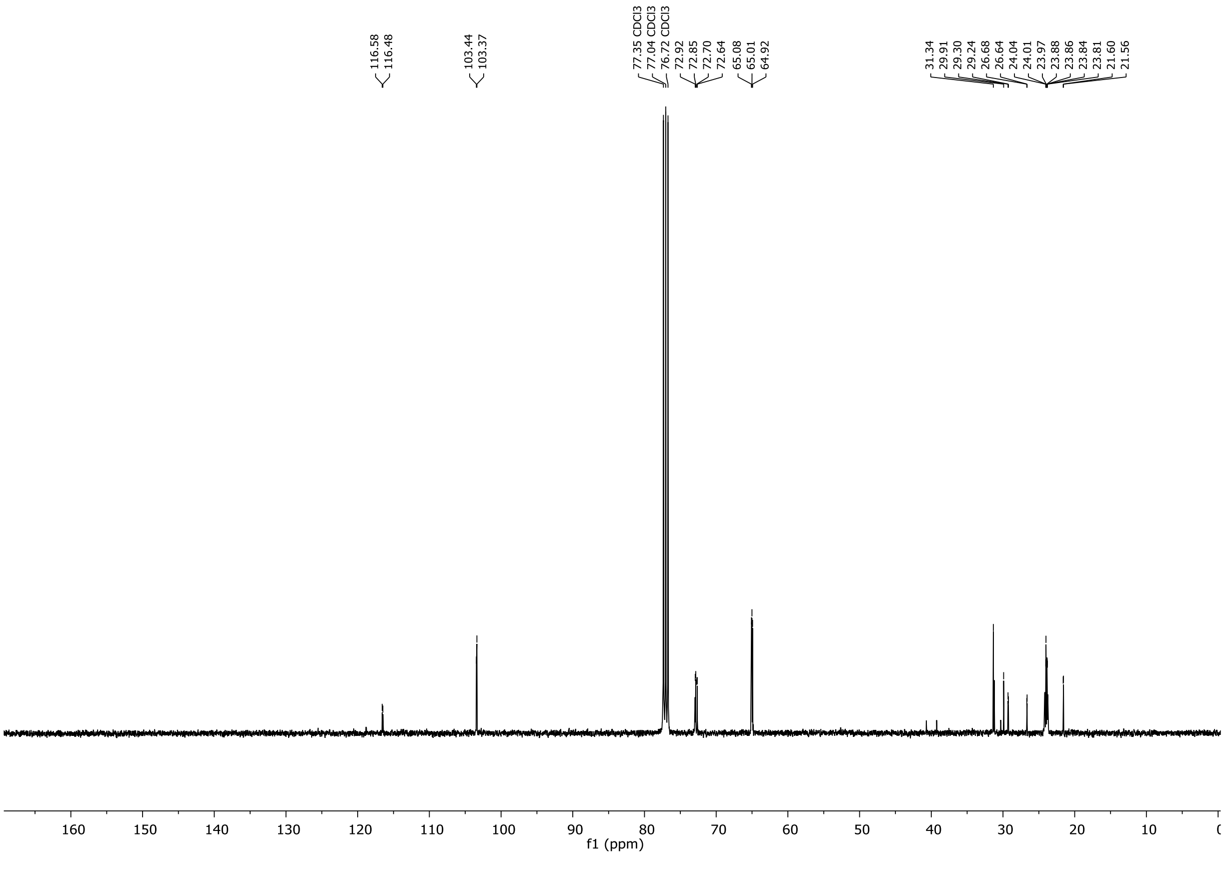

^{31}P NMR

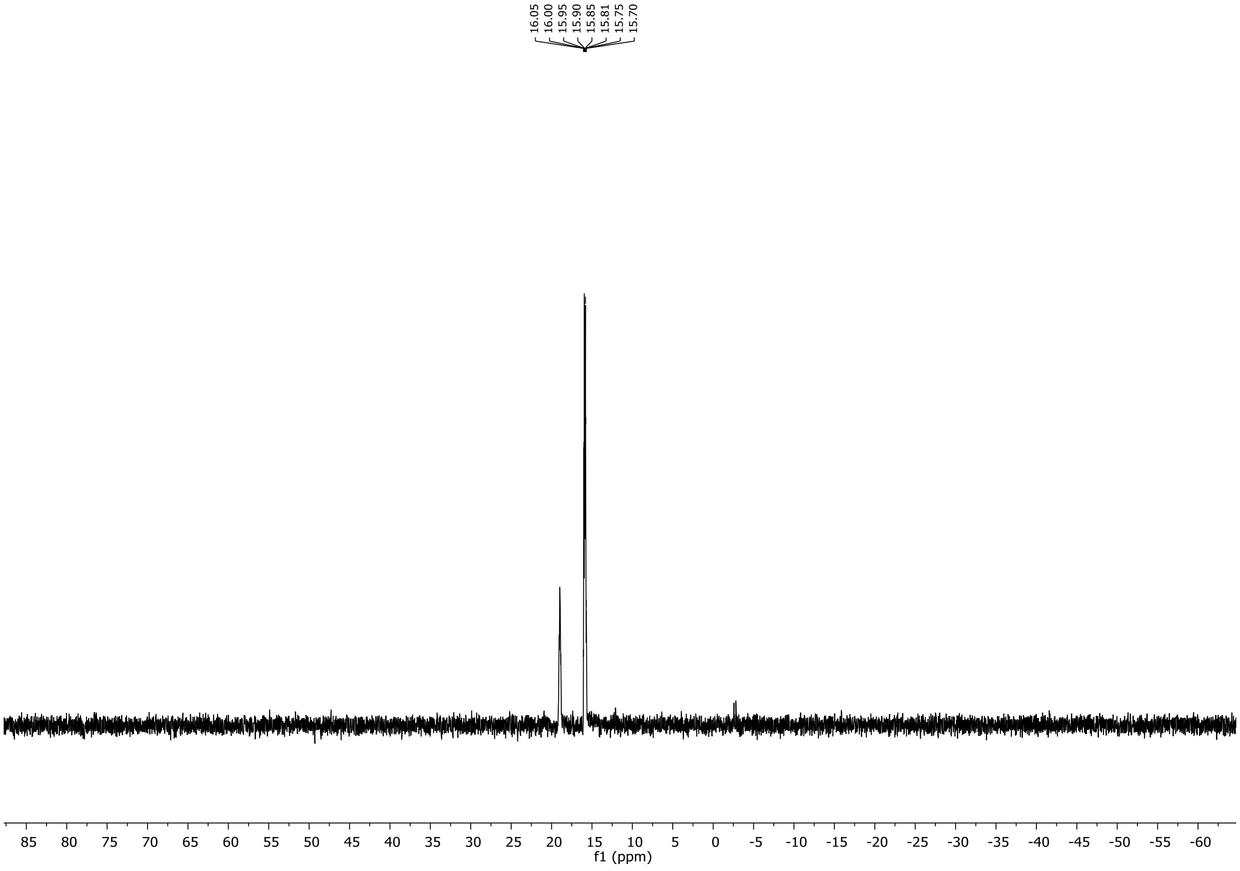

Diisopropyl (3-((tert-butyldimethylsilyl)oxy)-1-cyanopropyl)phosphonate (P11)

^{1}H NMR

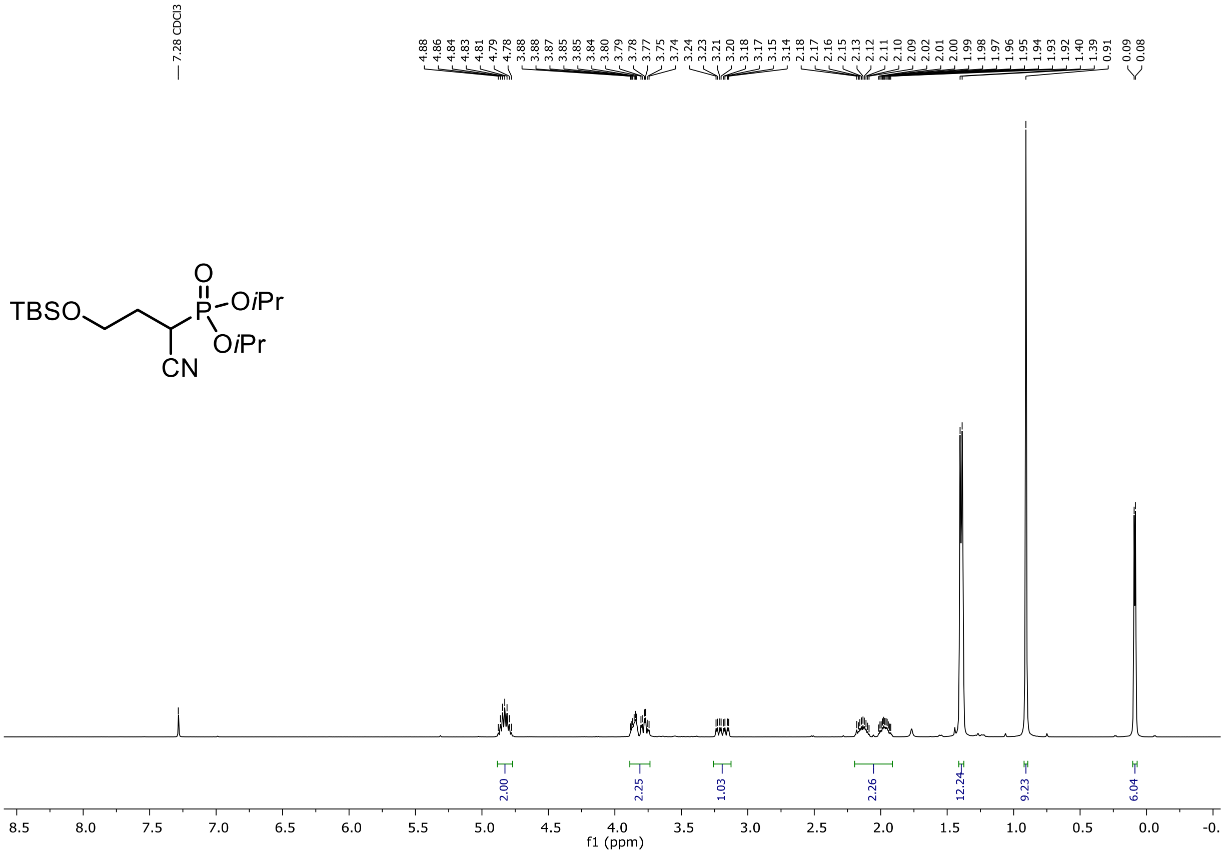

^{13}C NMR

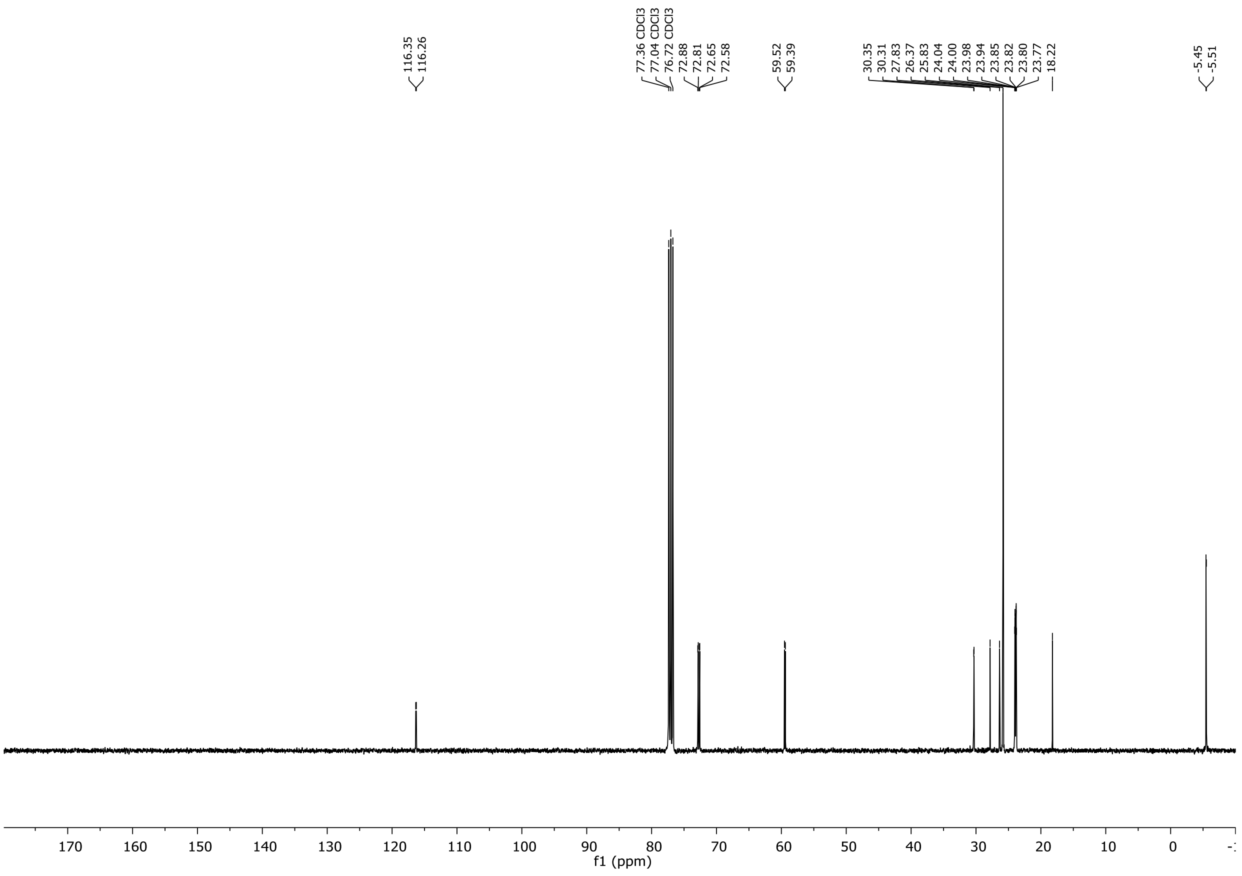

^{31}P NMR

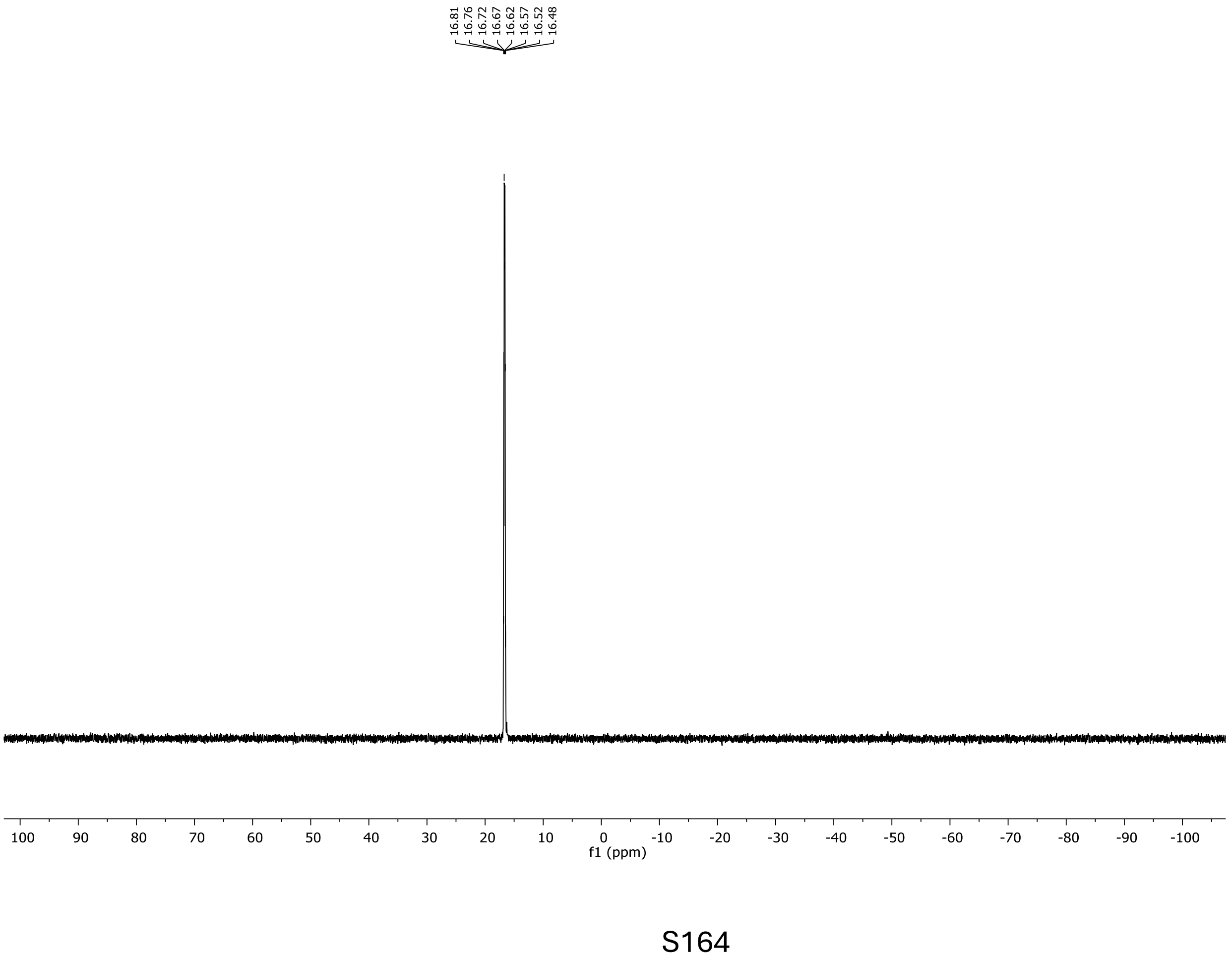

Diisopropyl (1-cyanobut-3-en-1-yl)phosphonate (P12)

^{1}H NMR

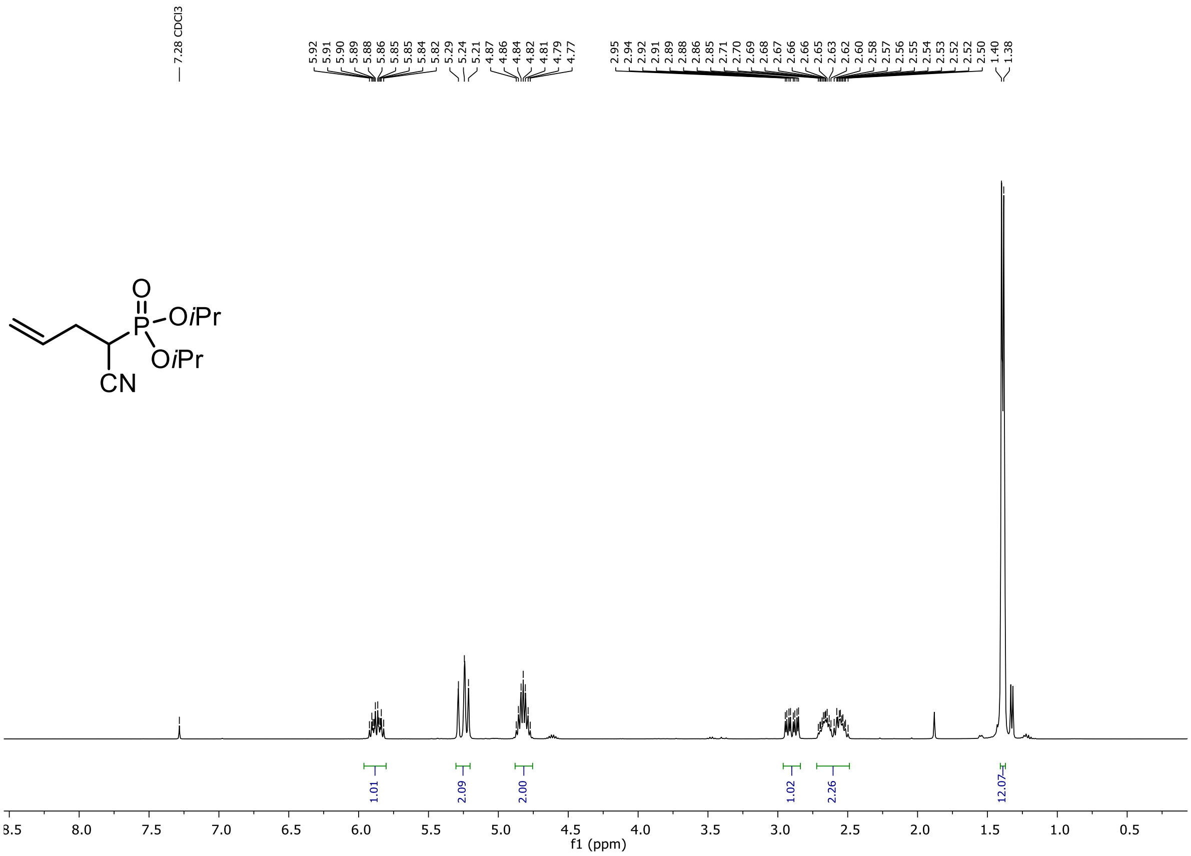

^{13}C NMR

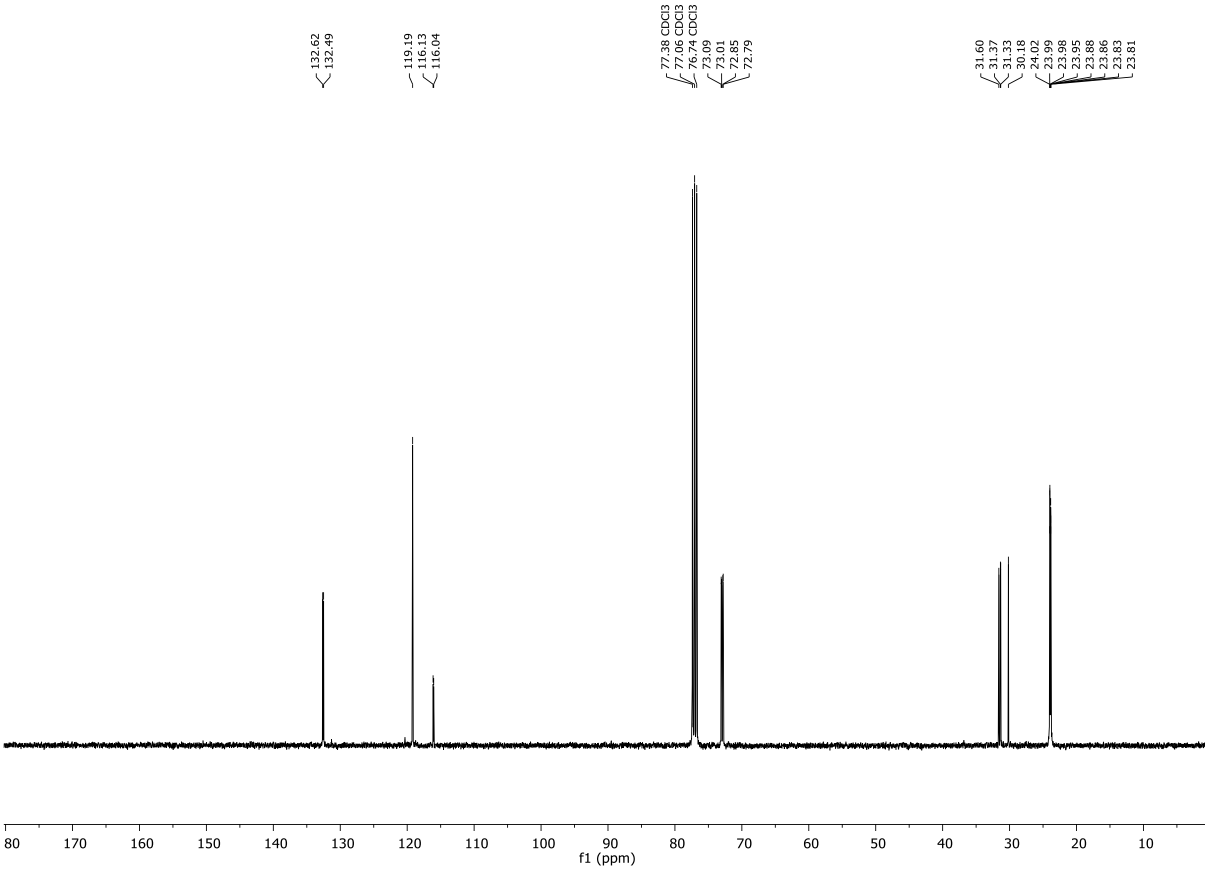

^{31}P NMR

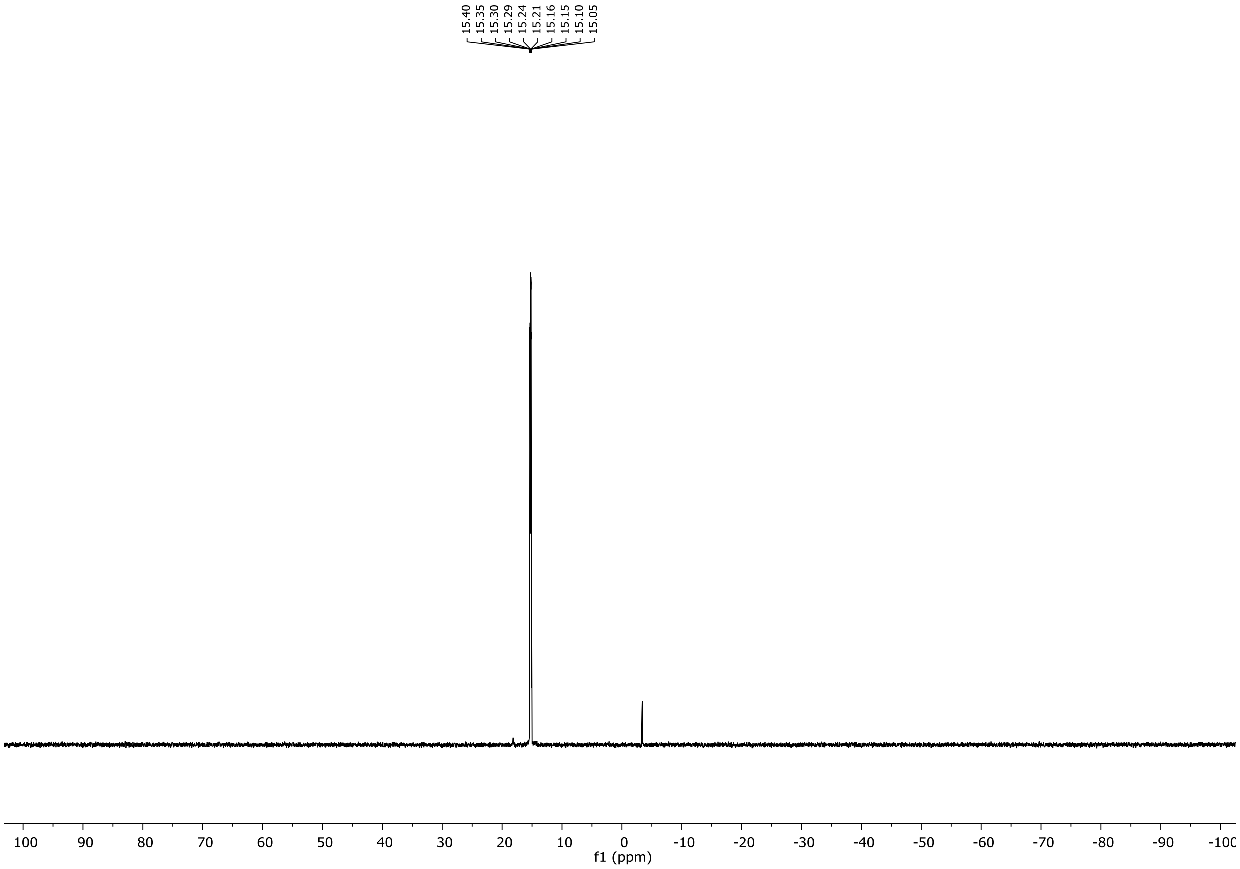

Diethyl (1-cyanoethyl)phosphonate (P13)

^{1}H NMR

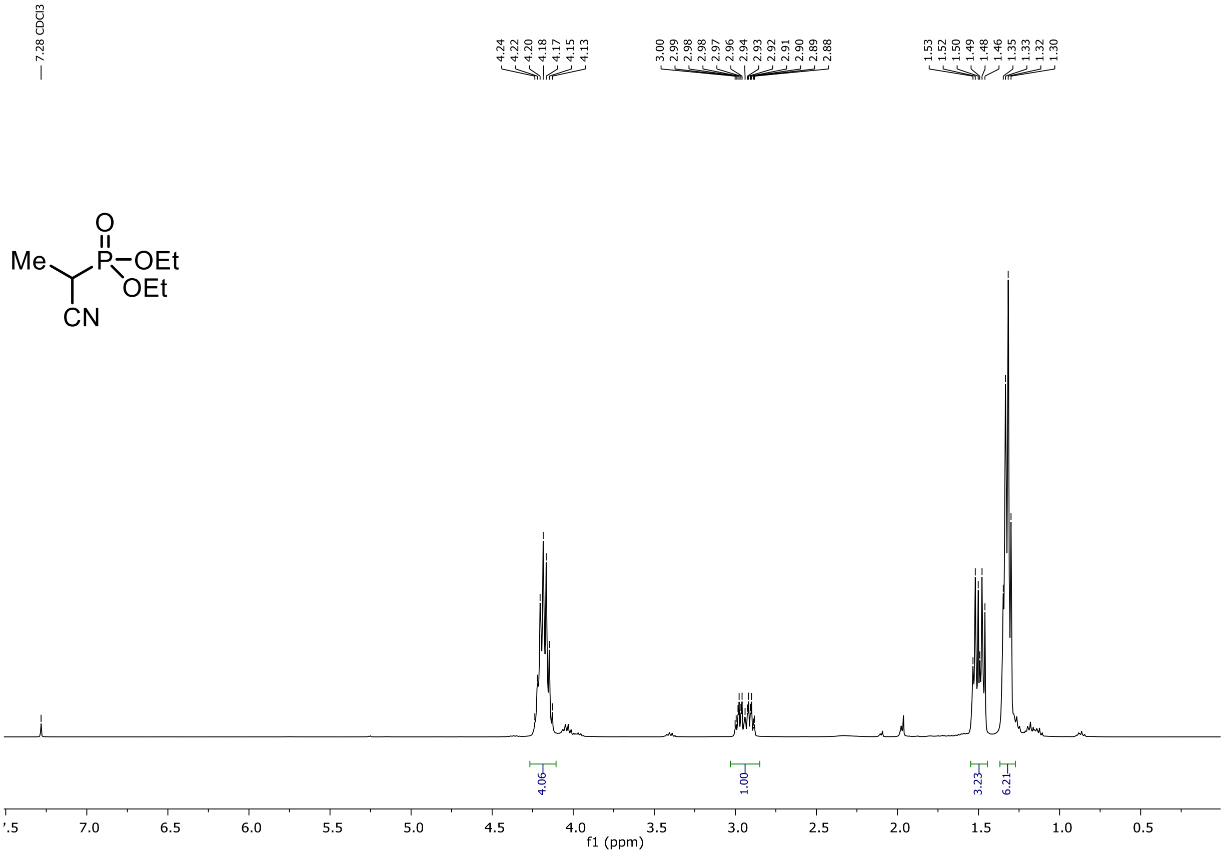

^{13}C NMR

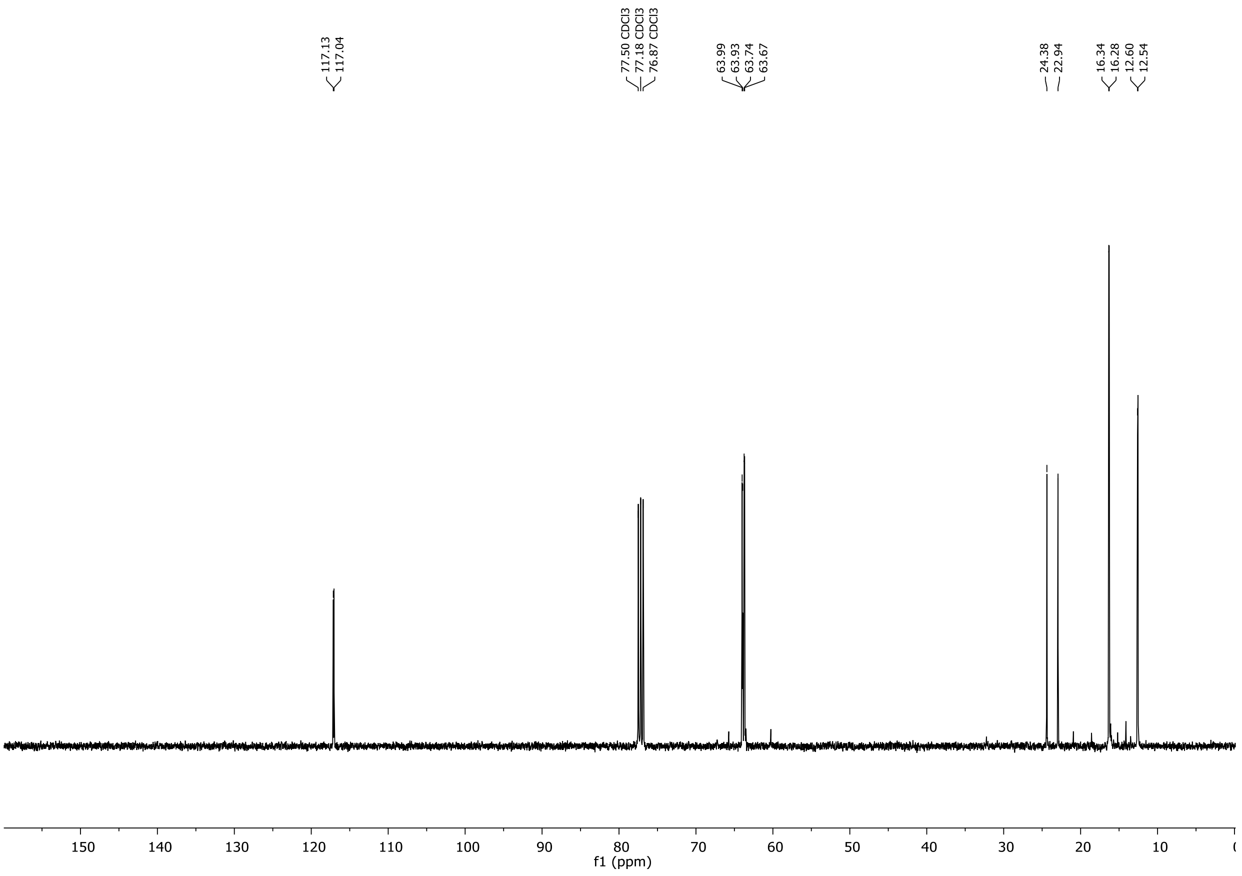

^{31}P NMR

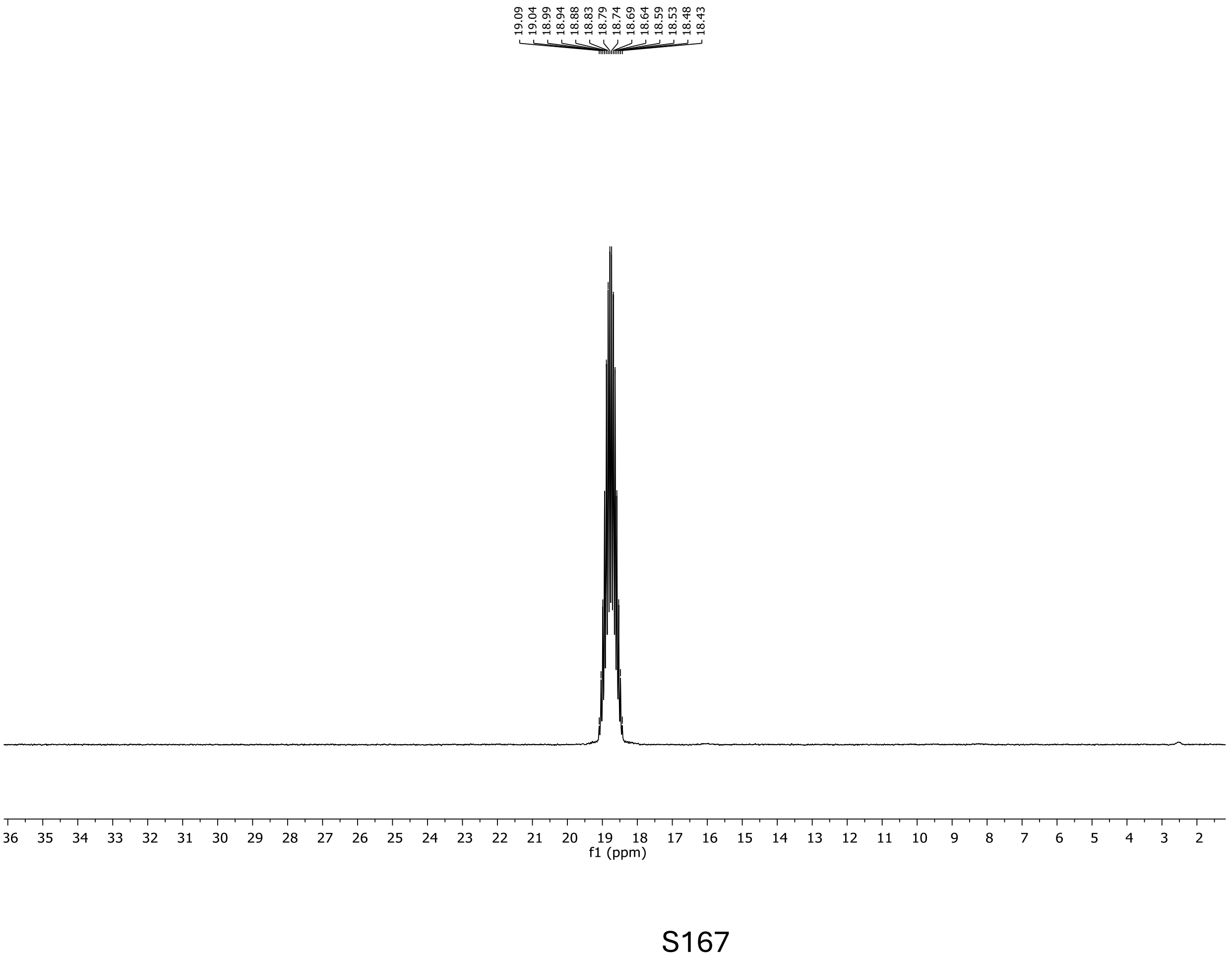

Diisopropyl (cyanofluoromethyl)phosphonate (P17)

^{1}H NMR

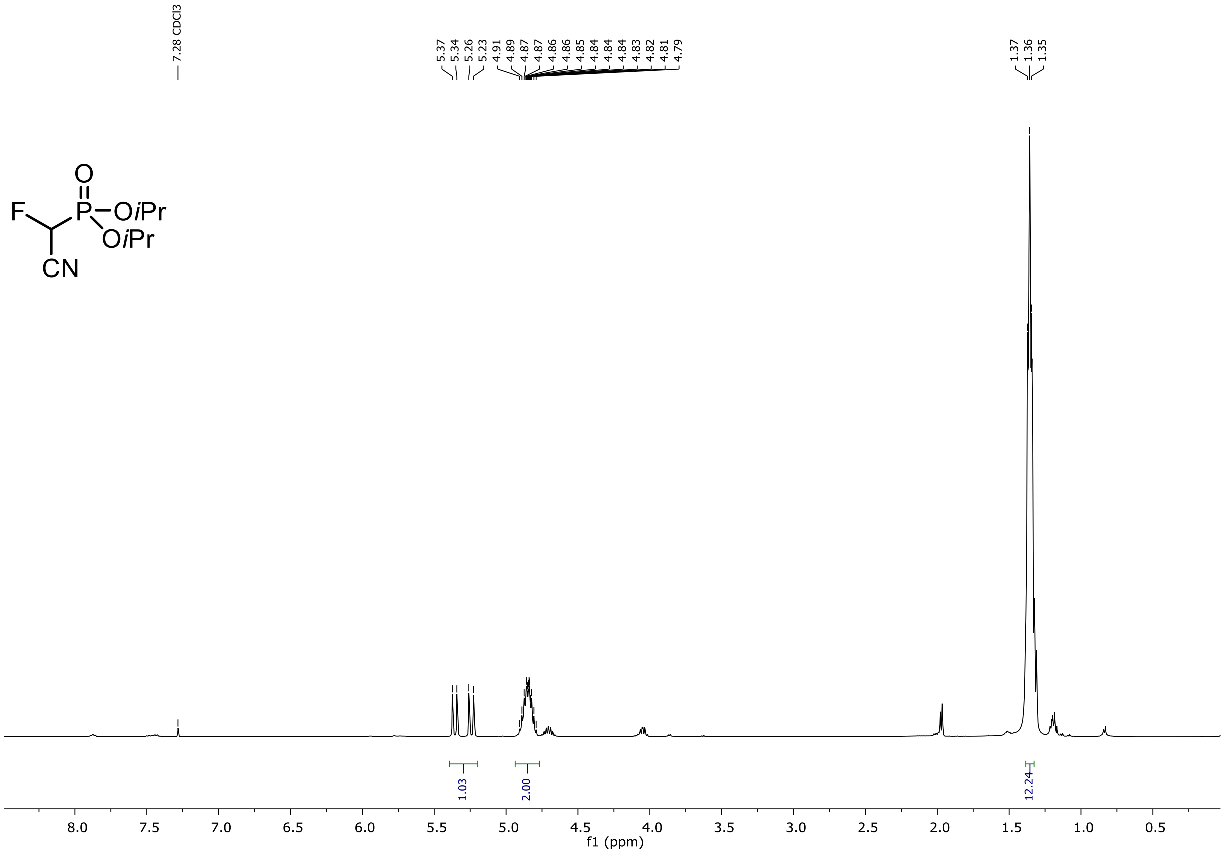

^{13}C NMR

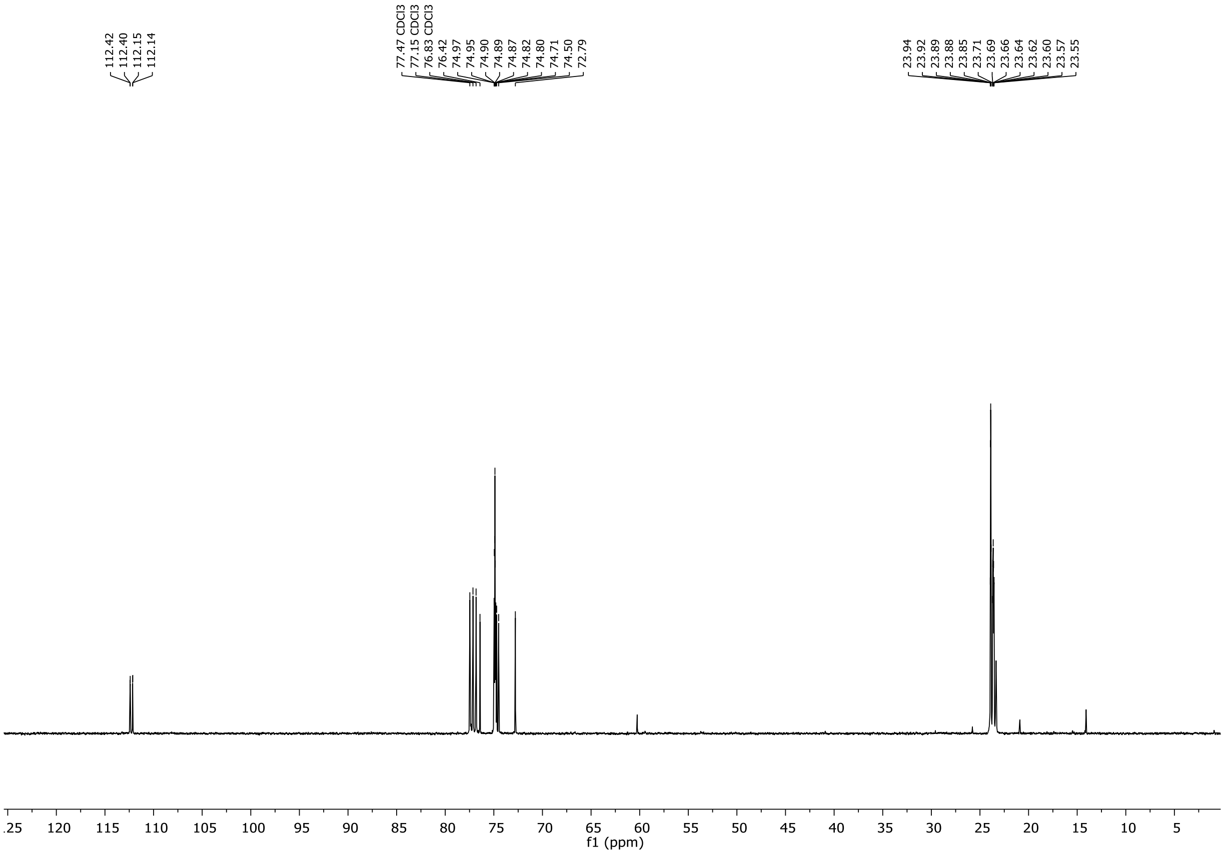

^{31}P NMR

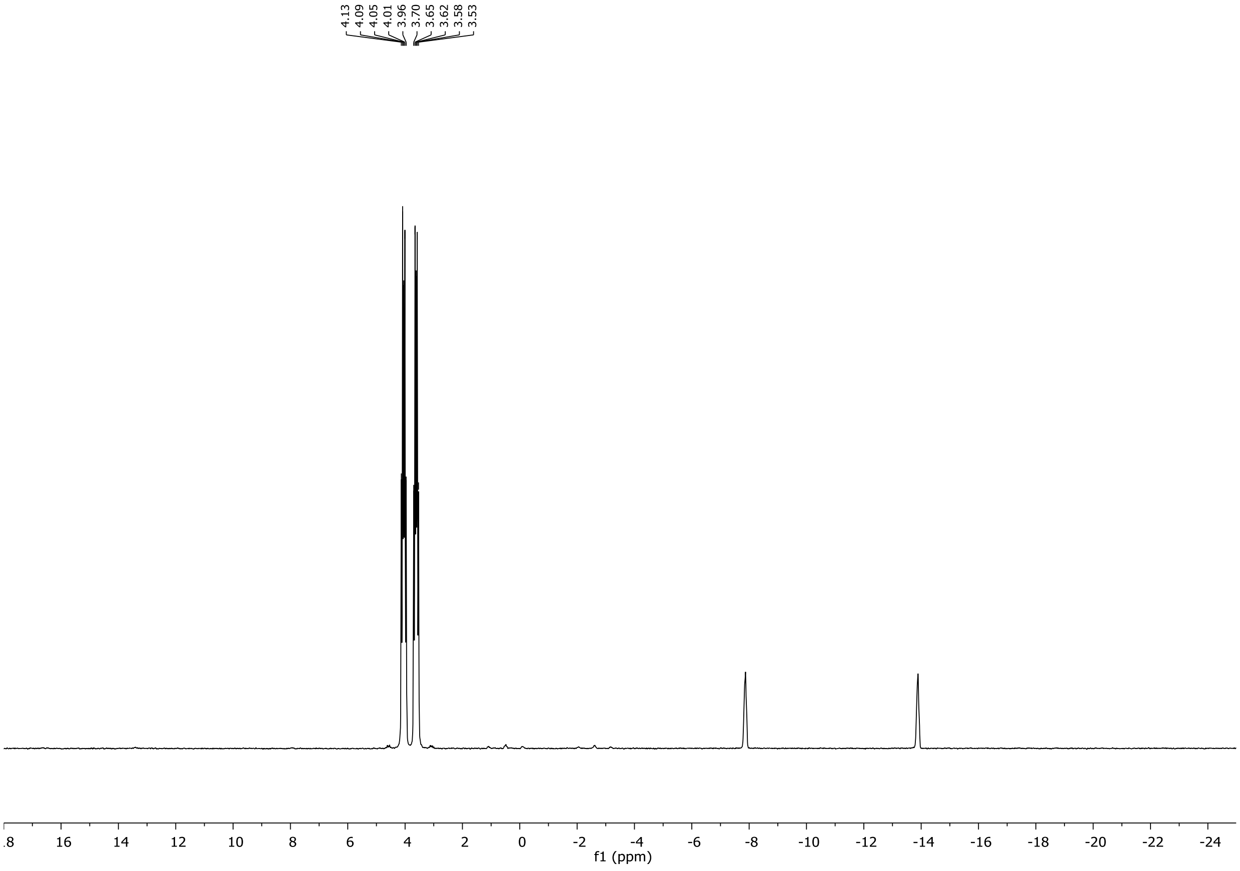

^{19}F NMR

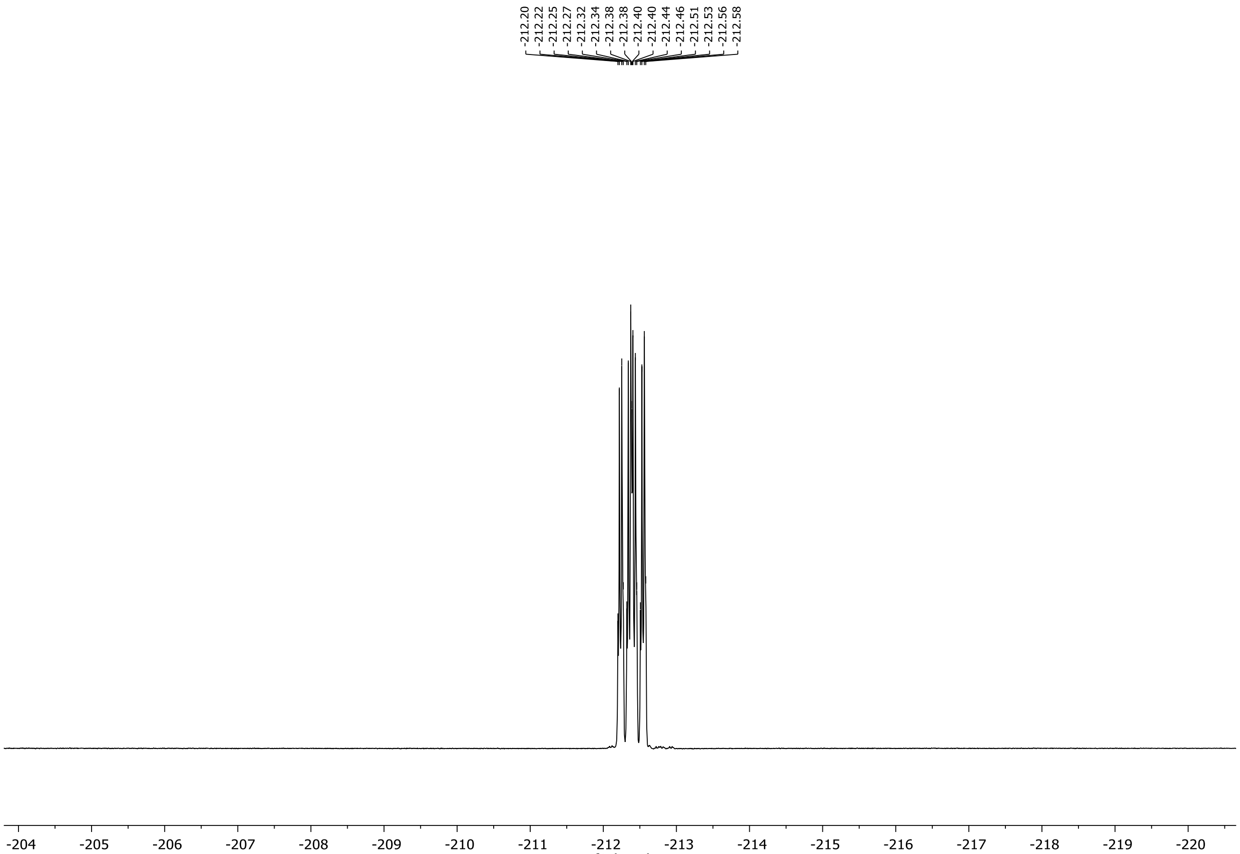

Diethyl (cyano(methylthio)methyl)phosphonate (P18)

^{1}H NMR

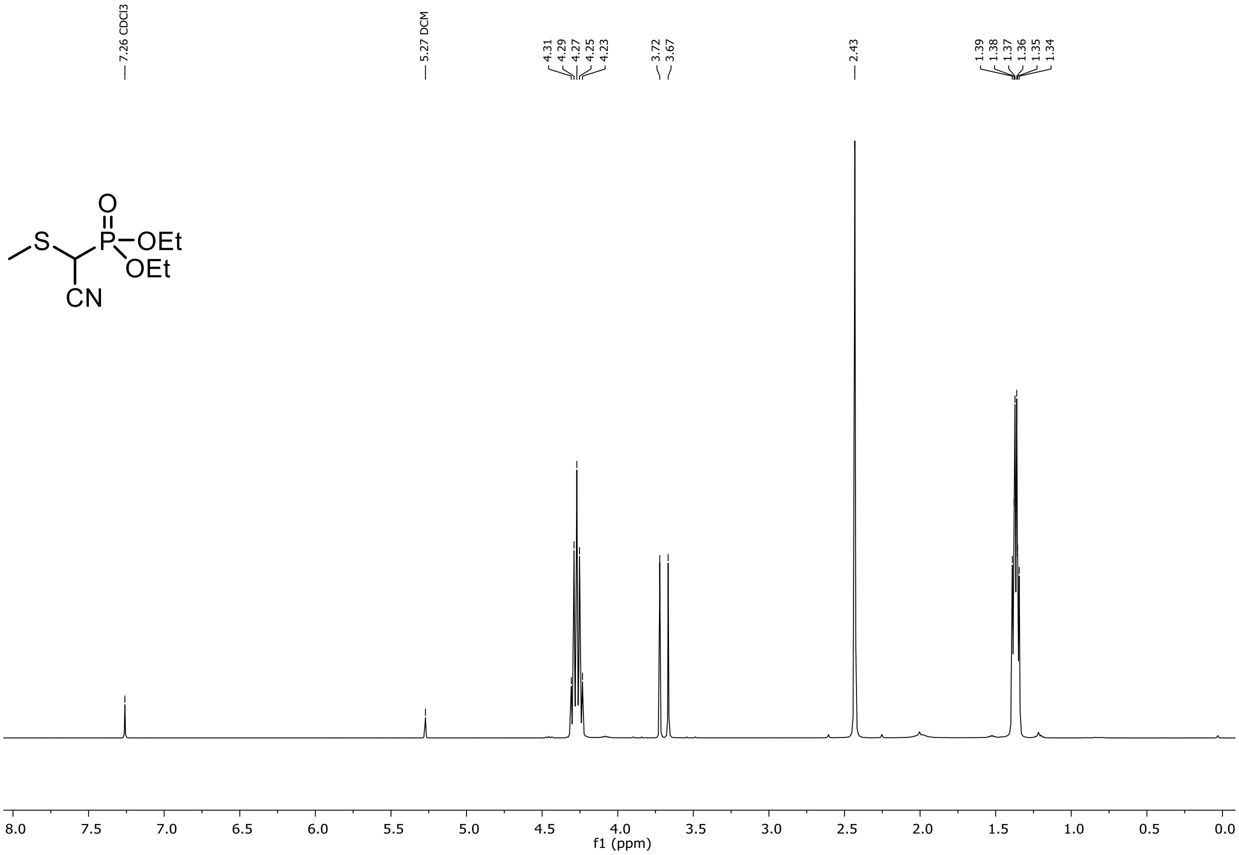

^{13}C NMR

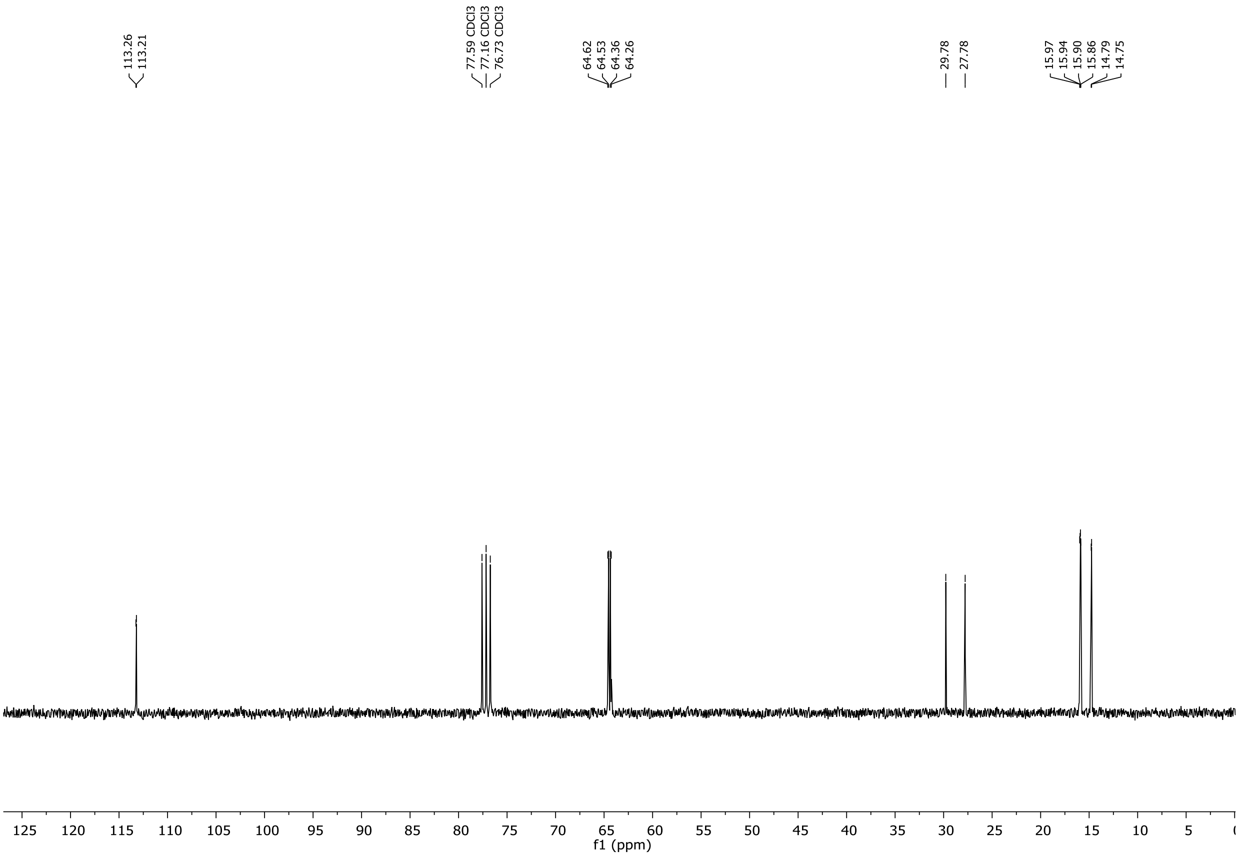

^{31}P NMR

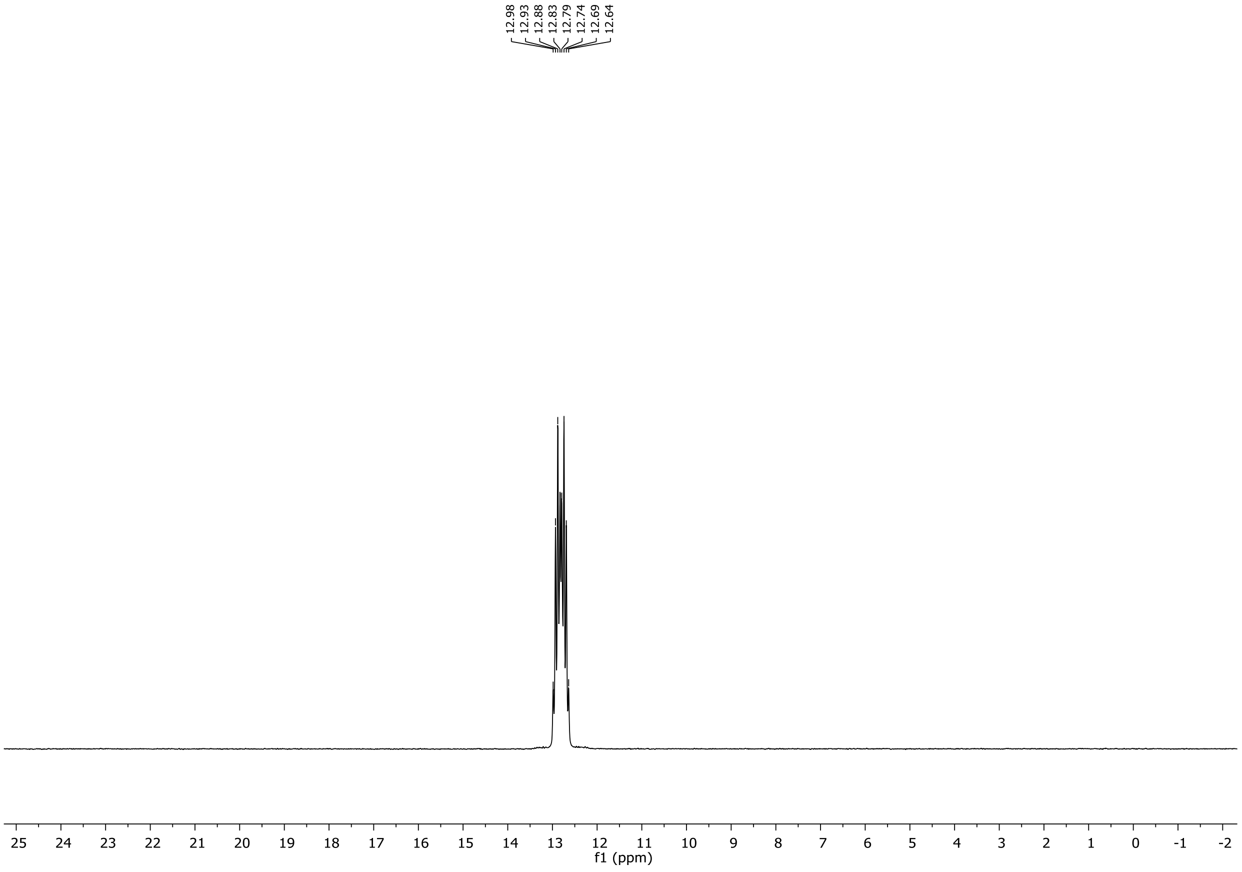
12.98
12.93
12.88
12.83
12.79
12.74
12.69
12.64
25 24 23 22 21 20 19 18 17 16 15 14 13 12 11 10 9 8 7 6 5 4 3 2 1 0 -1 -2
f1 (ppm)

4-ethyl-5-hydroxyfuran-2(5H)-one (A1)

^{1}H NMR

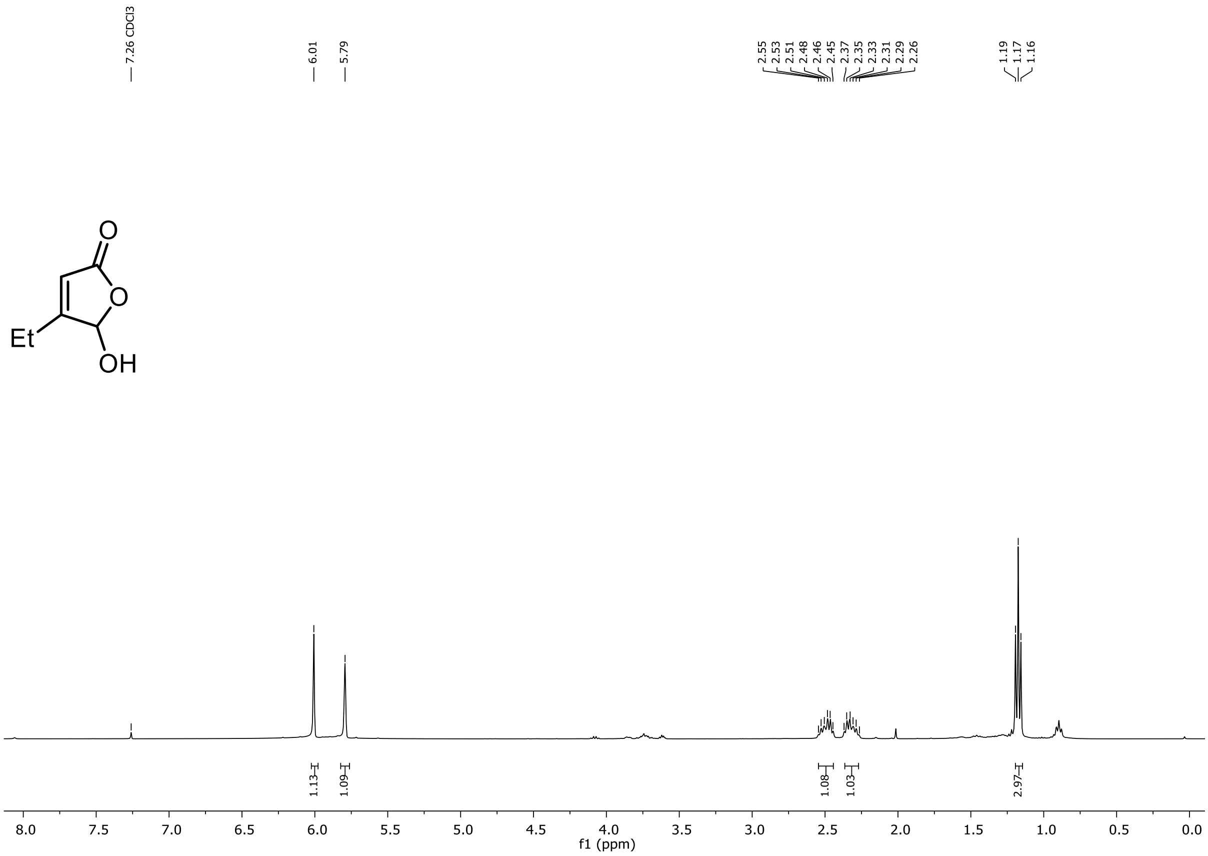

^{13}C NMR

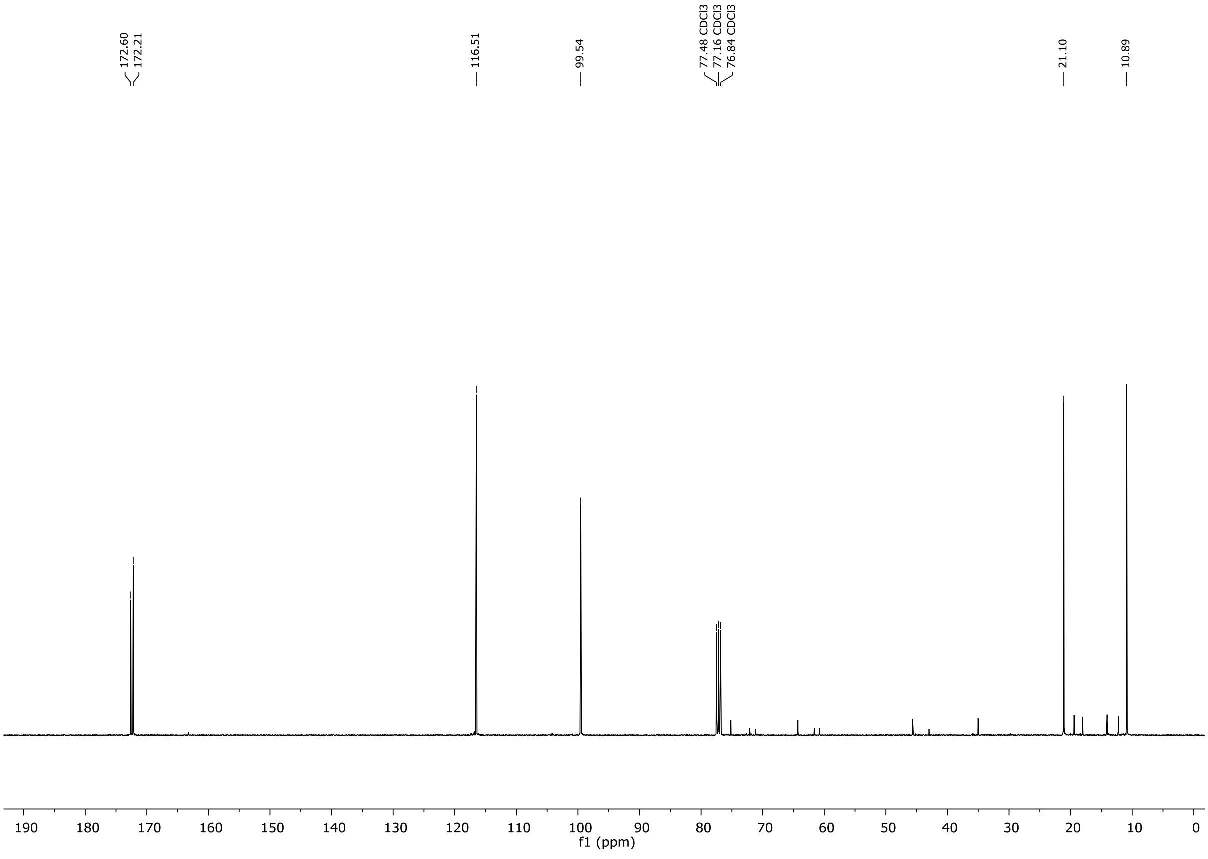

Methyl (*E*)-3-formylpent-2-enoate (A2)

^{1}H NMR (*dr*=70:30)

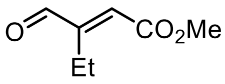

minor diast

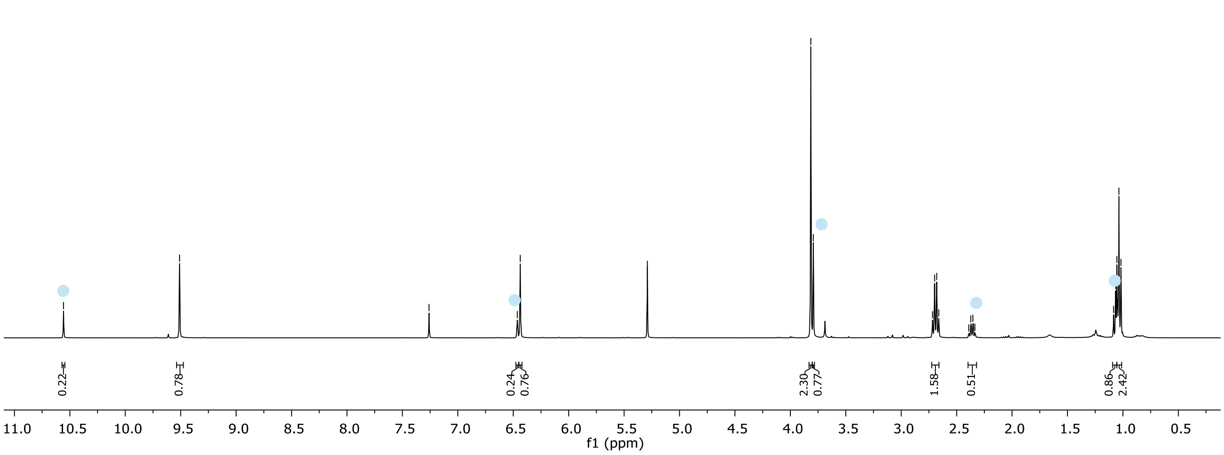

^{13}C NMR

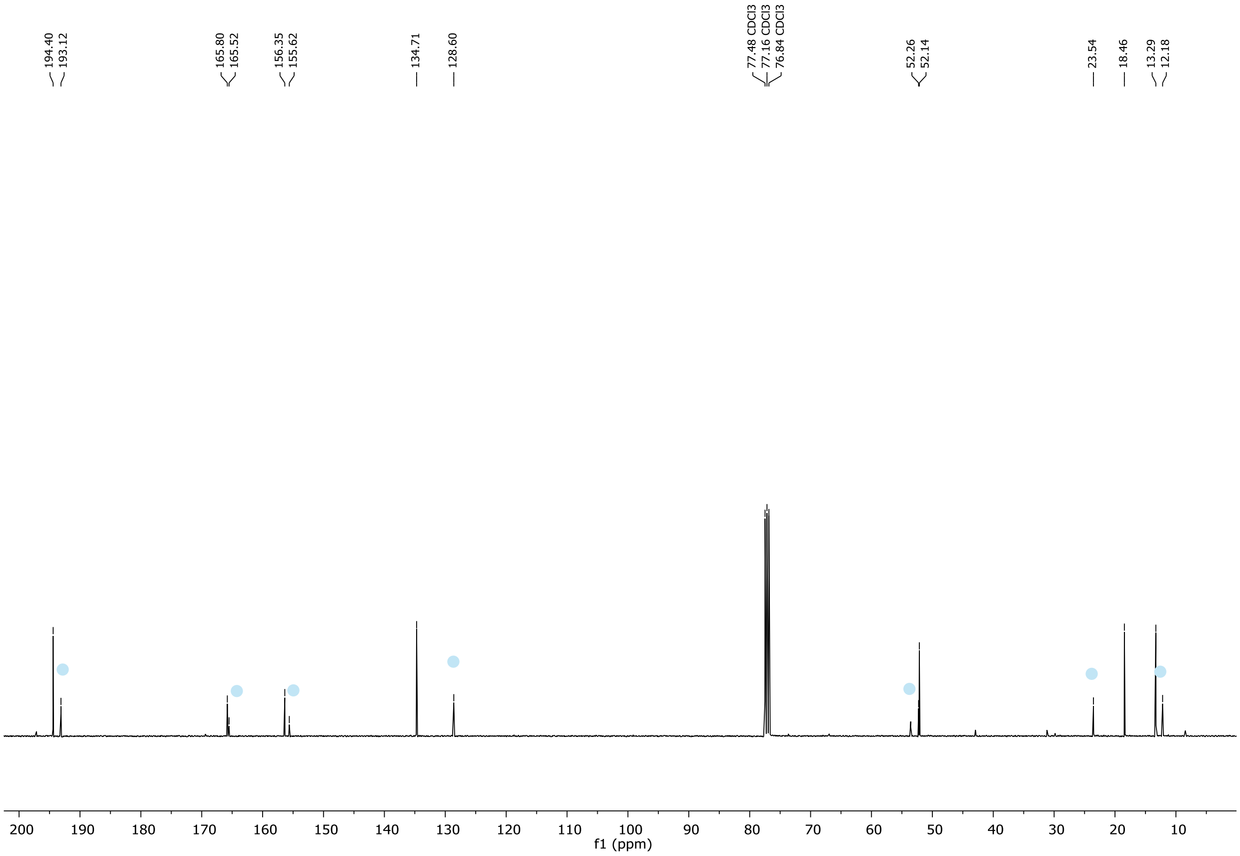

5-hydroxy-4-isopropylfuran-2(5H)-one (A3)

¹H NMR

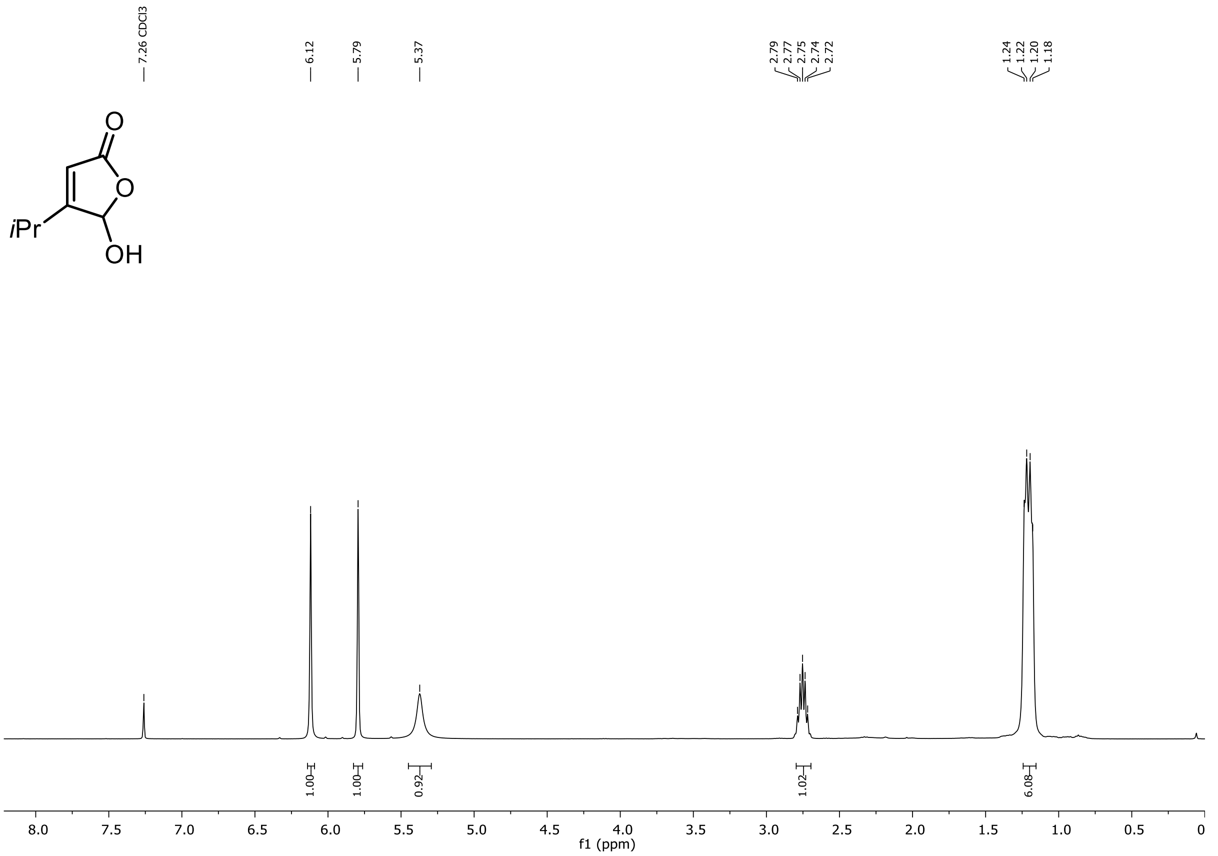

¹³C NMR

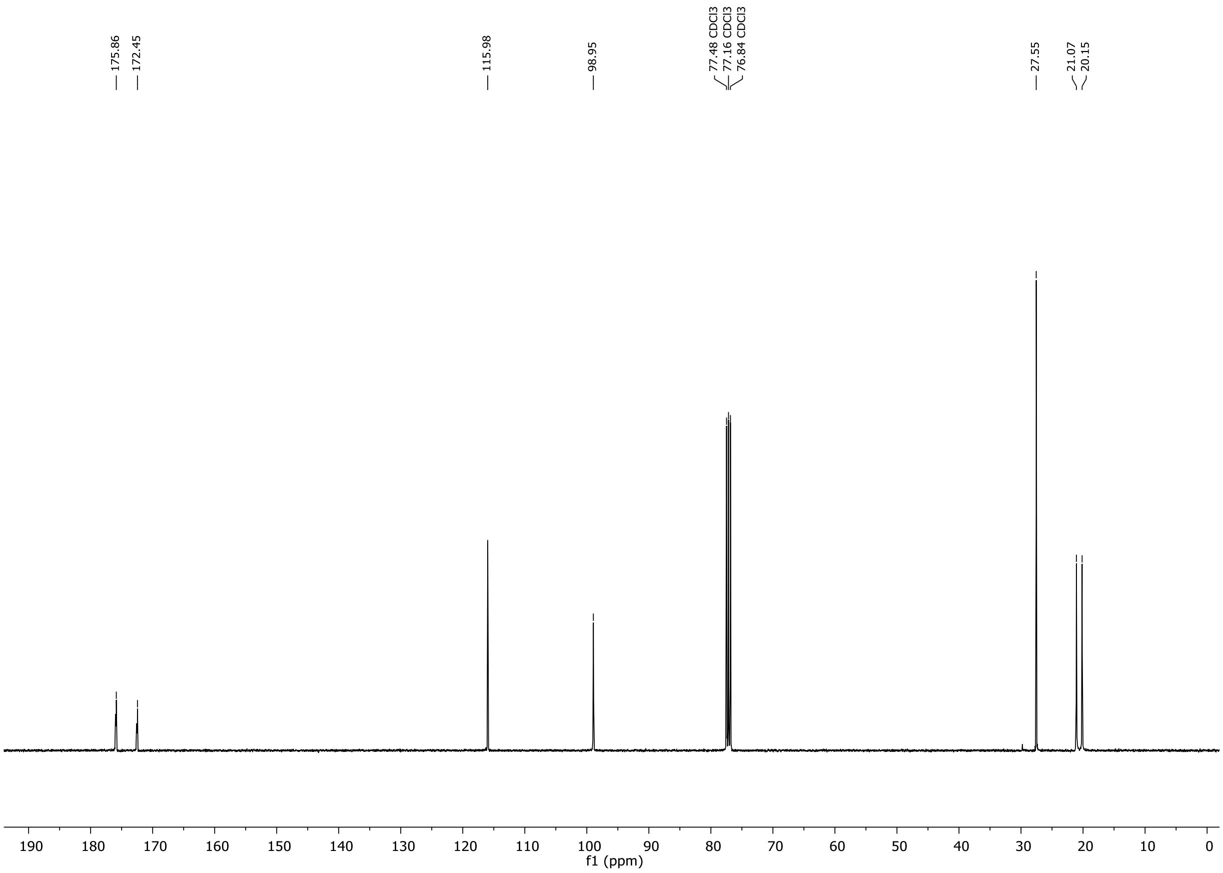

4-isopropyl-5-methoxyfuran-2(5H)-one (A4)

^{1}H NMR (*dr*=80:20)

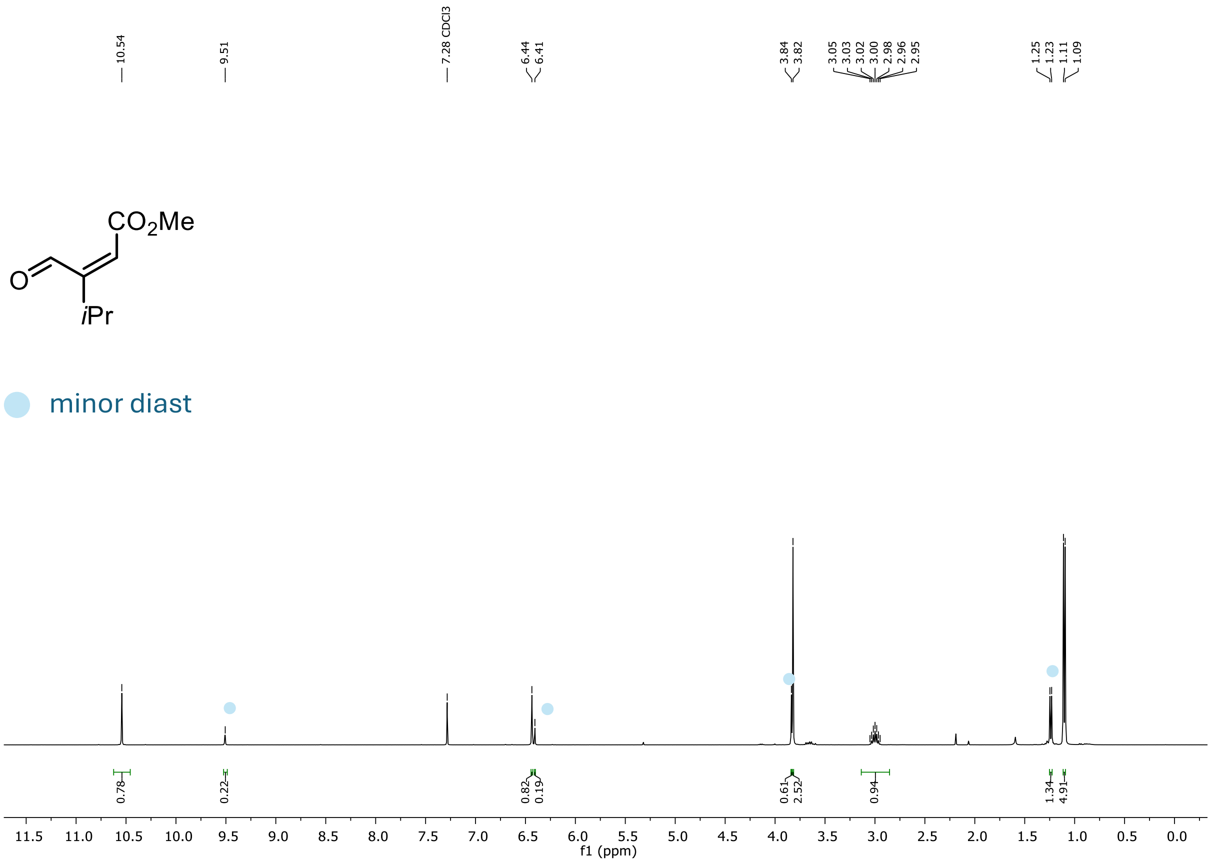

^{13}C NMR

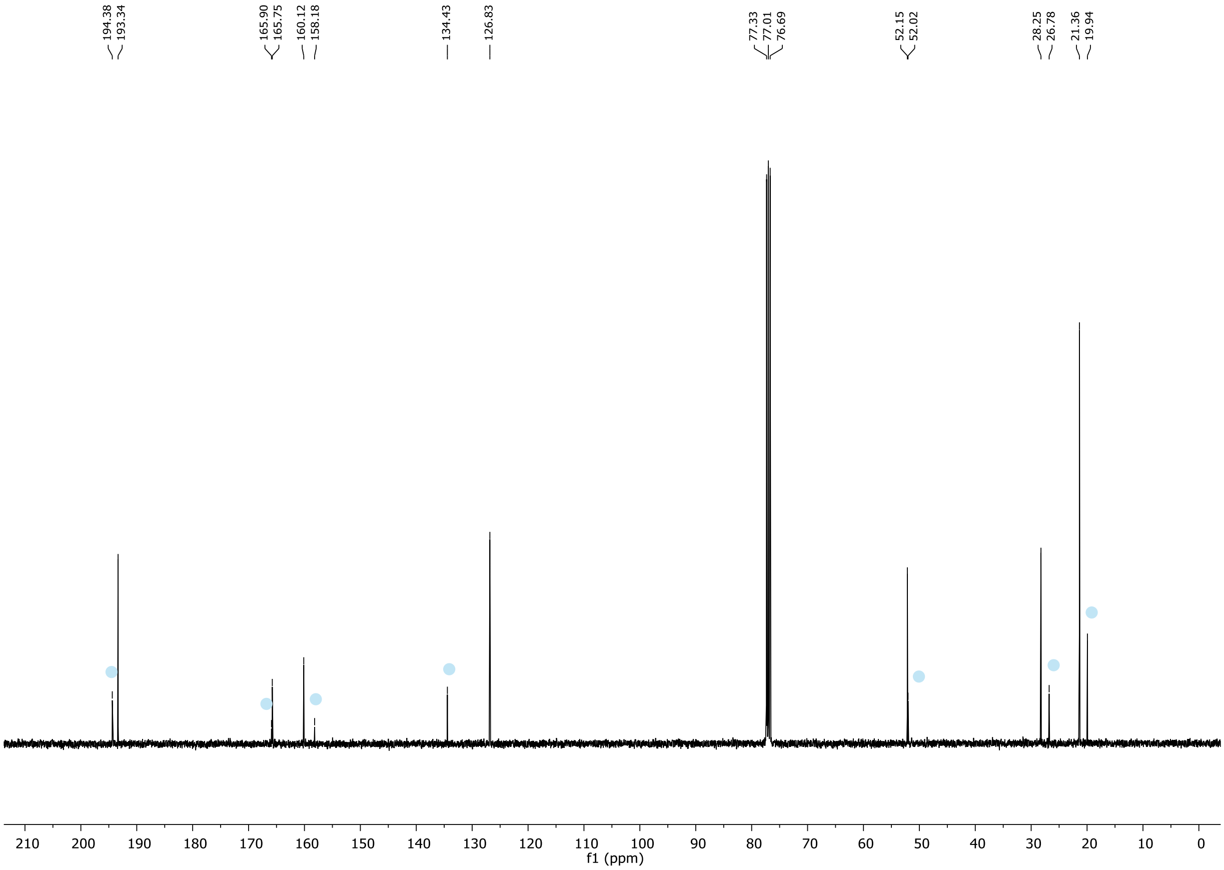

Ethyl (*E*)-3-formylhepta-2,6-dienoate (A6)

^{1}H NMR

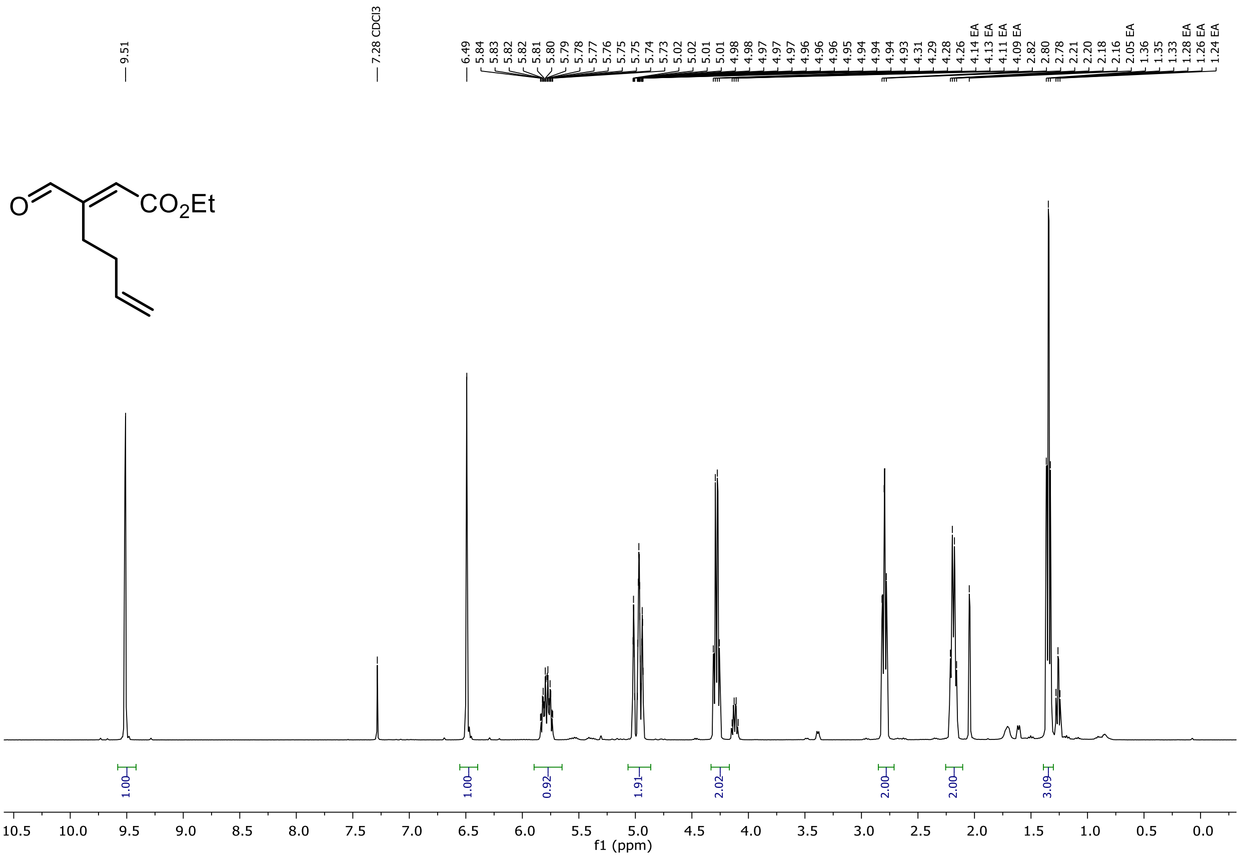

^{13}C NMR

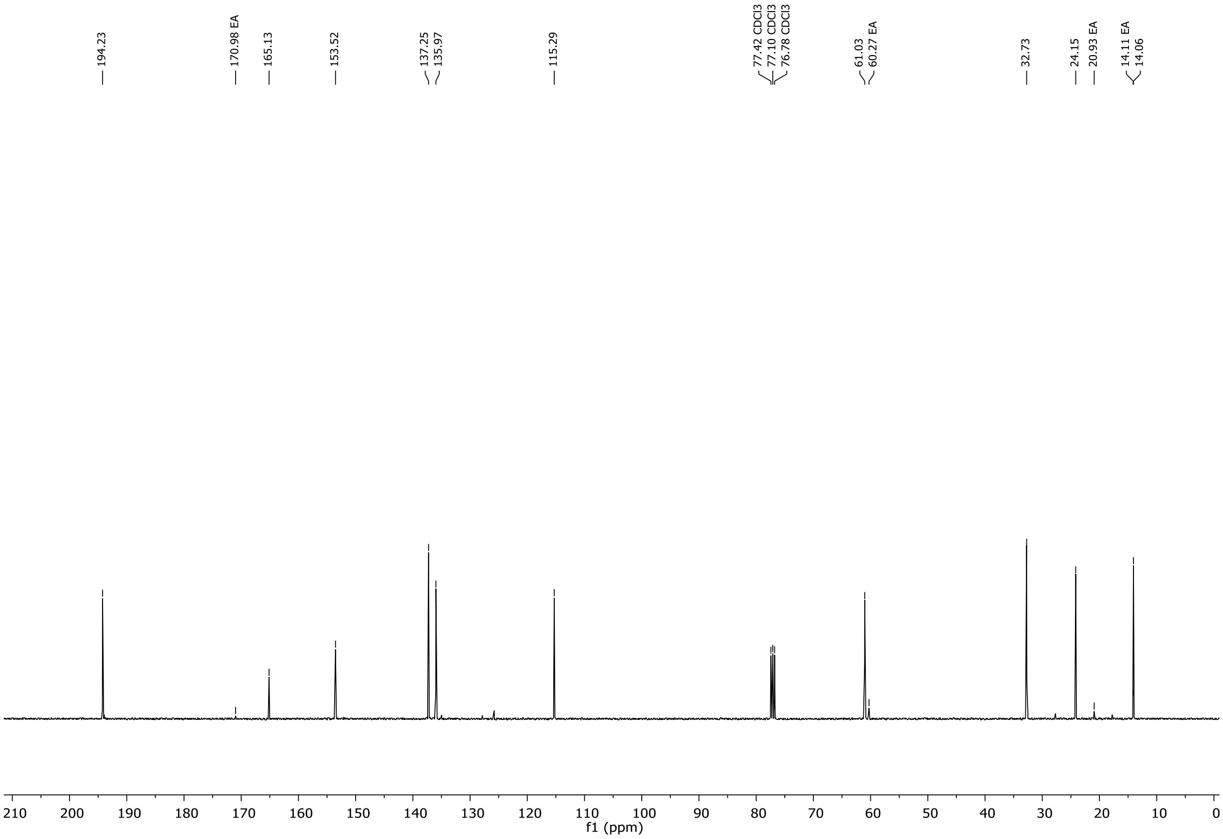

Ethyl (*E*)-5-(1,3-dioxolan-2-yl)-3-formylpent-2-enoate (A7)

^{1}H NMR

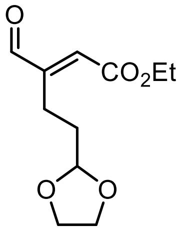

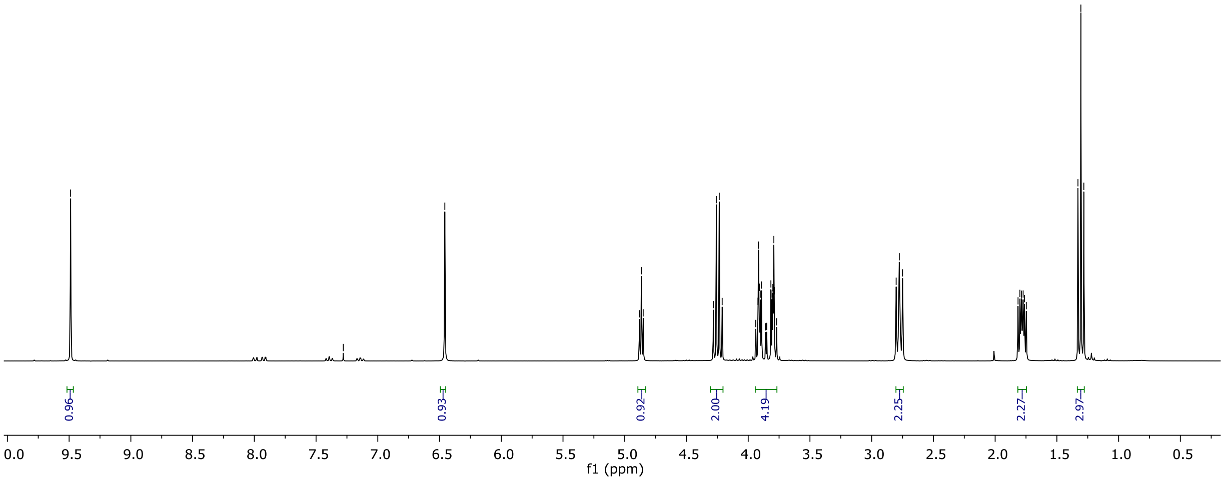

^{13}C NMR

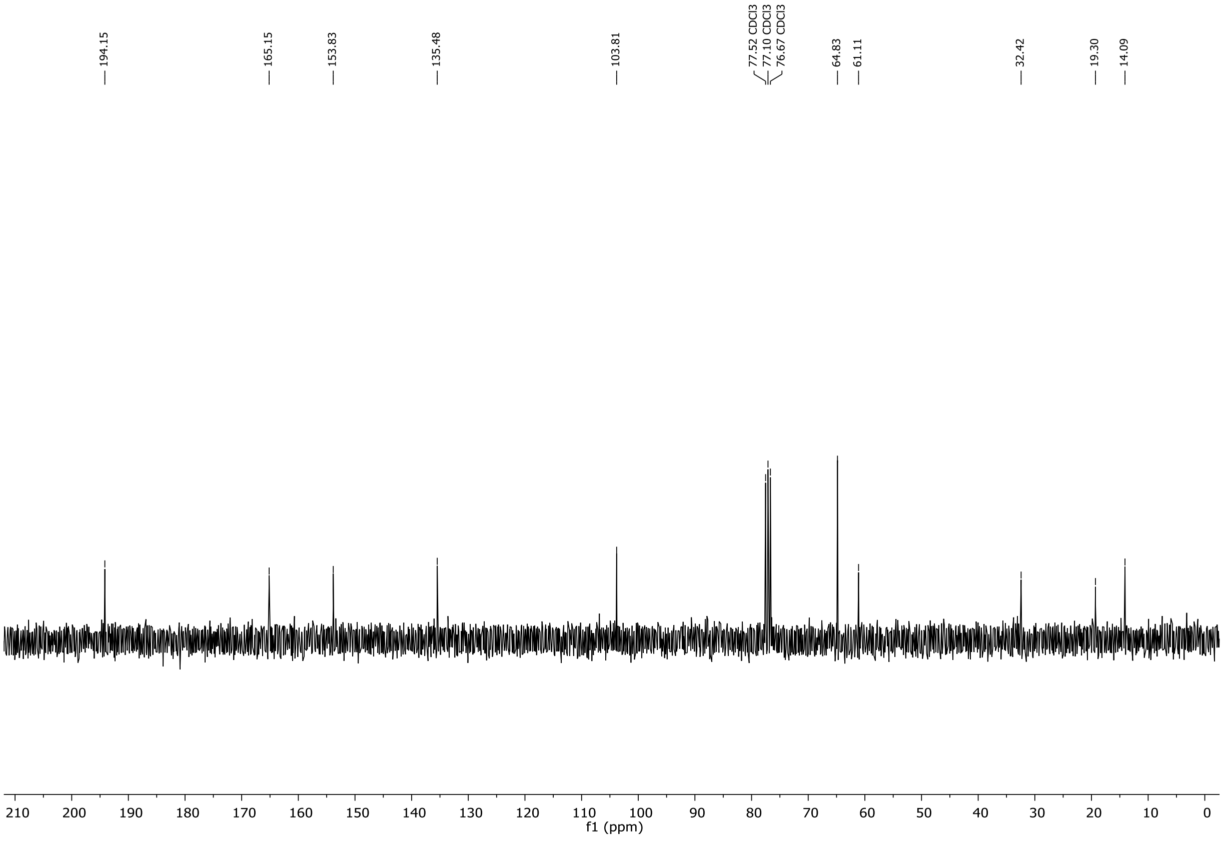

Ethyl (*E*)-4-oxo-3-phenylbut-2-enoate (A8)

^{1}H NMR

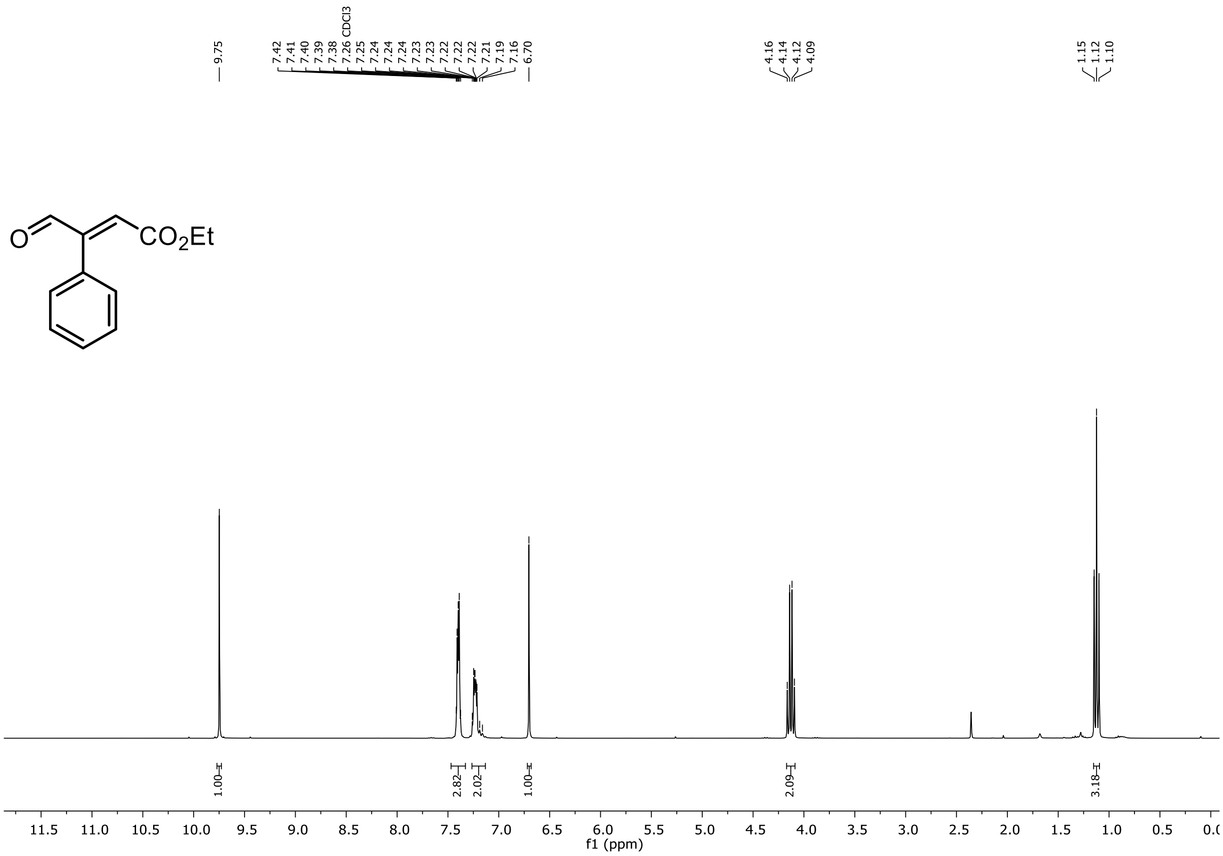

^{13}C NMR

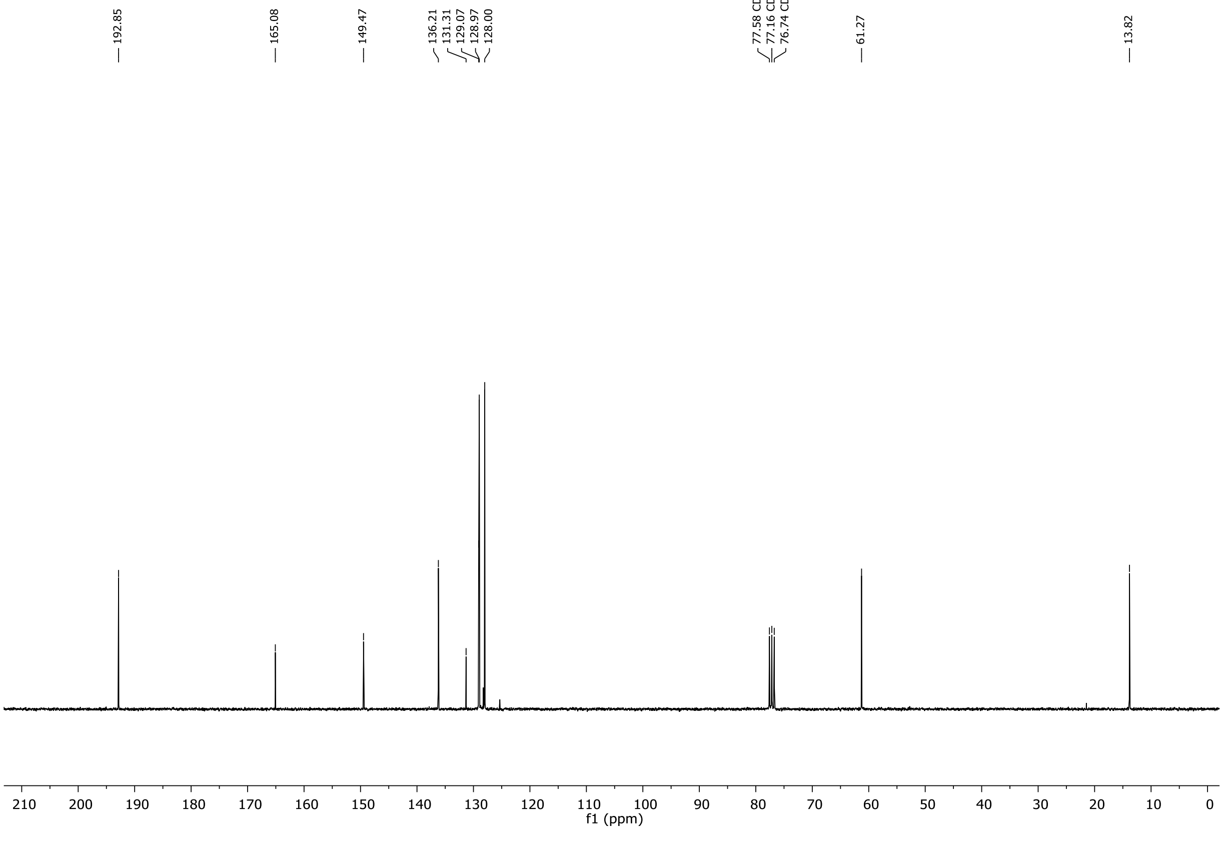

Ethyl (*E*)-3-(naphthalen-2-yl)-4-oxobut-2-enoate (A9)

^{1}H NMR

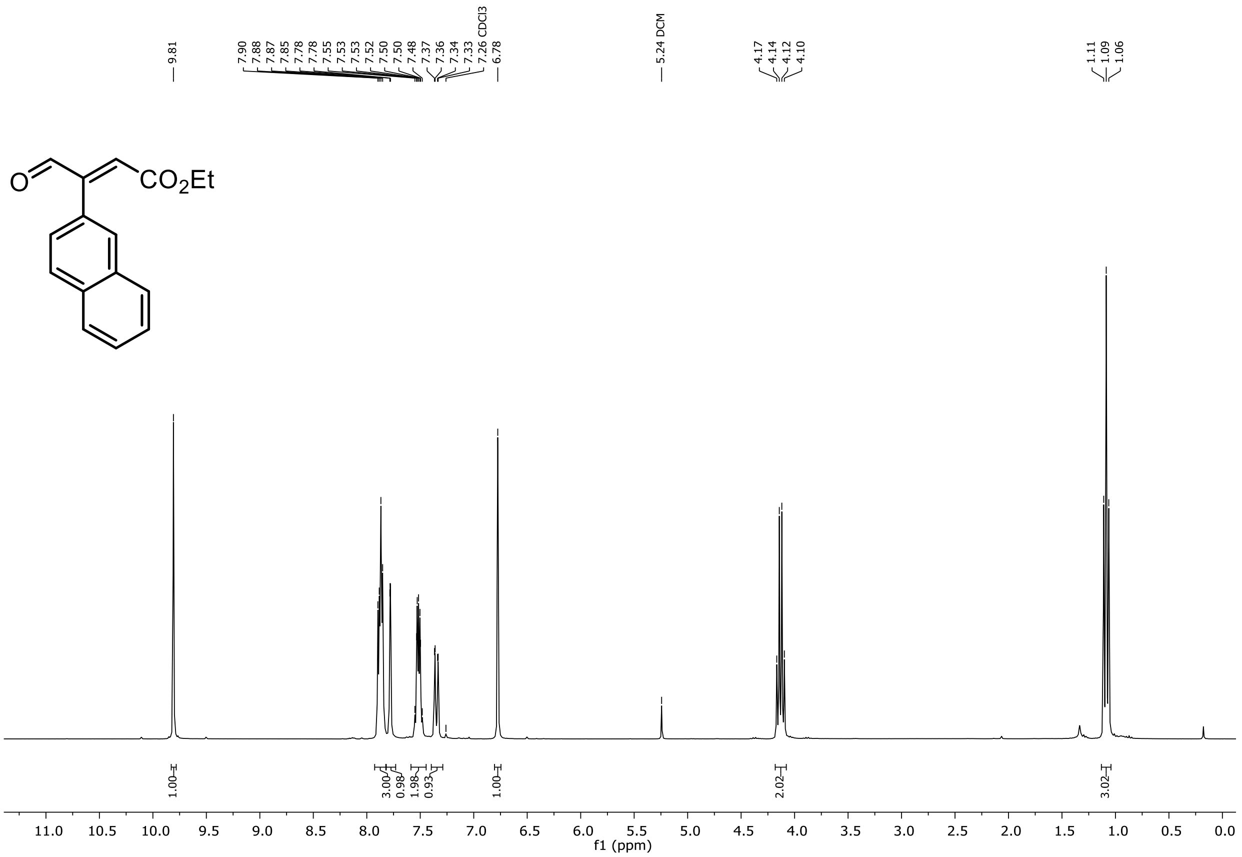

^{13}C NMR

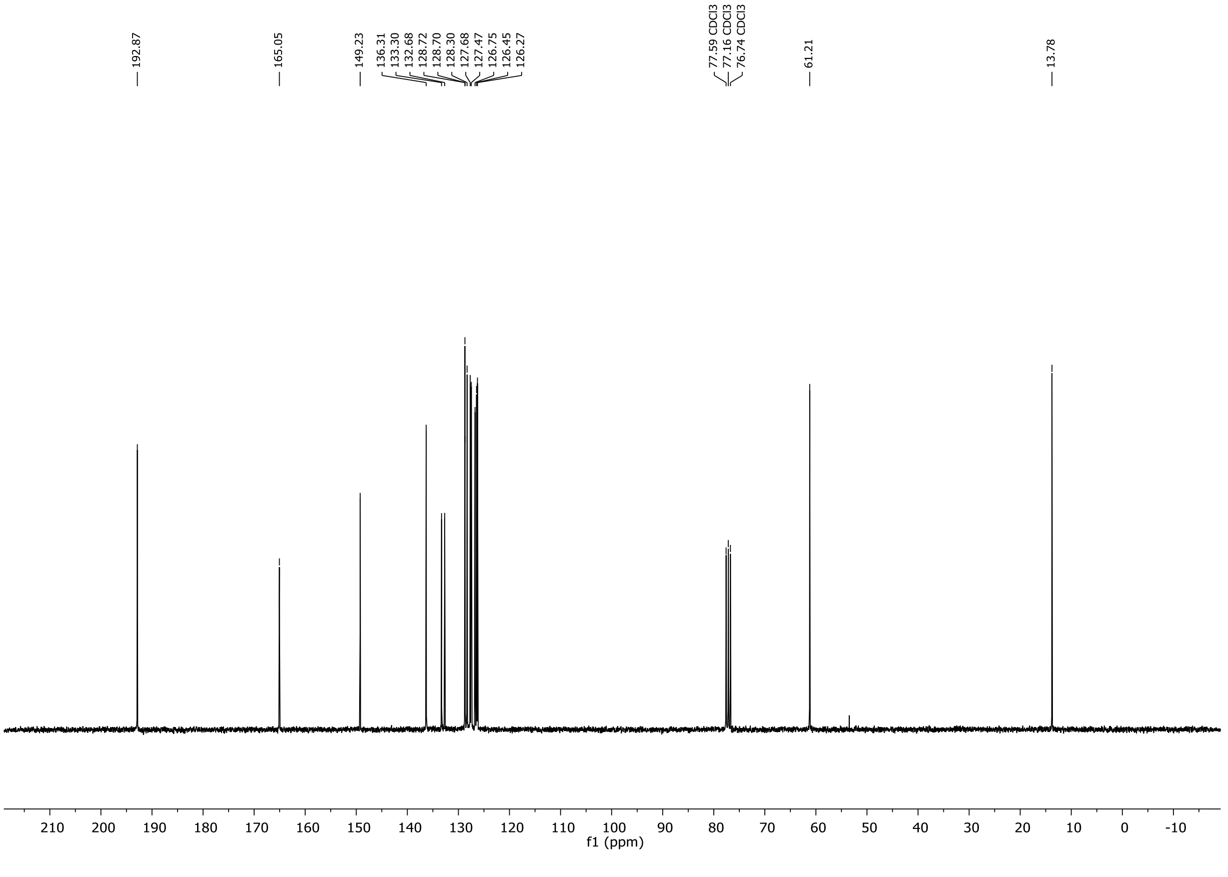

Ethyl (*E*)-4-oxo-3-(pyridin-4-yl)but-2-enoate (A10)

^{1}H NMR (*dr*=60:40)

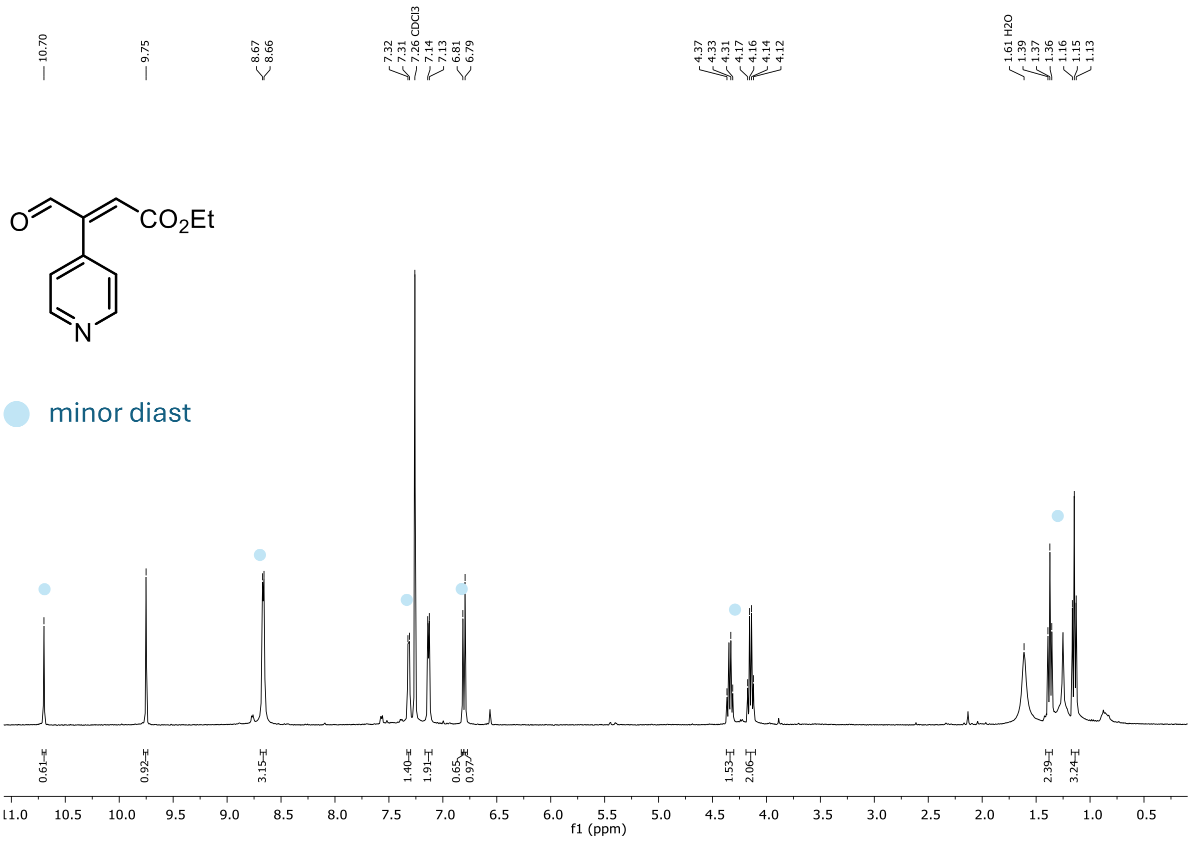

^{13}C NMR

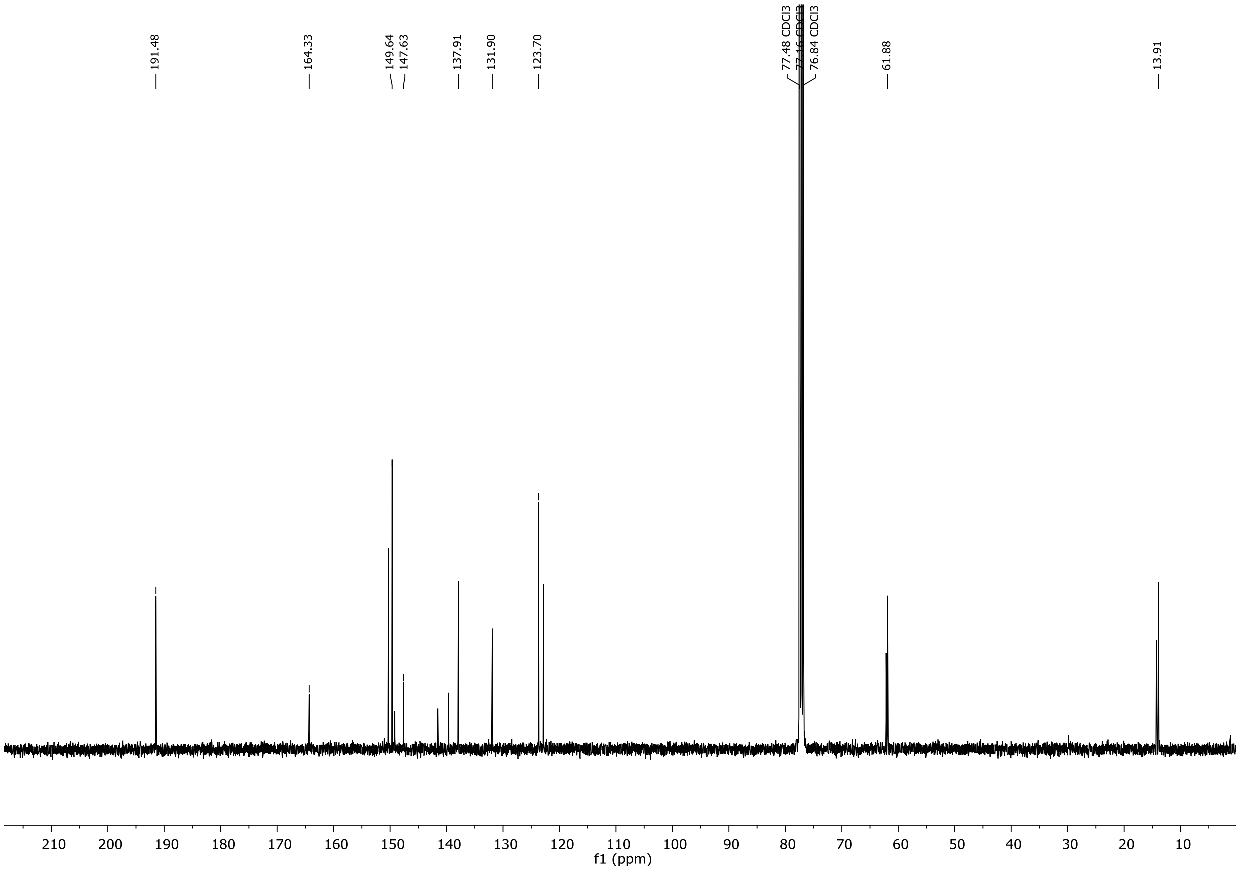

Ethyl (E)-3-methyl-4-oxopent-2-enoate (A11)

^{1}H NMR

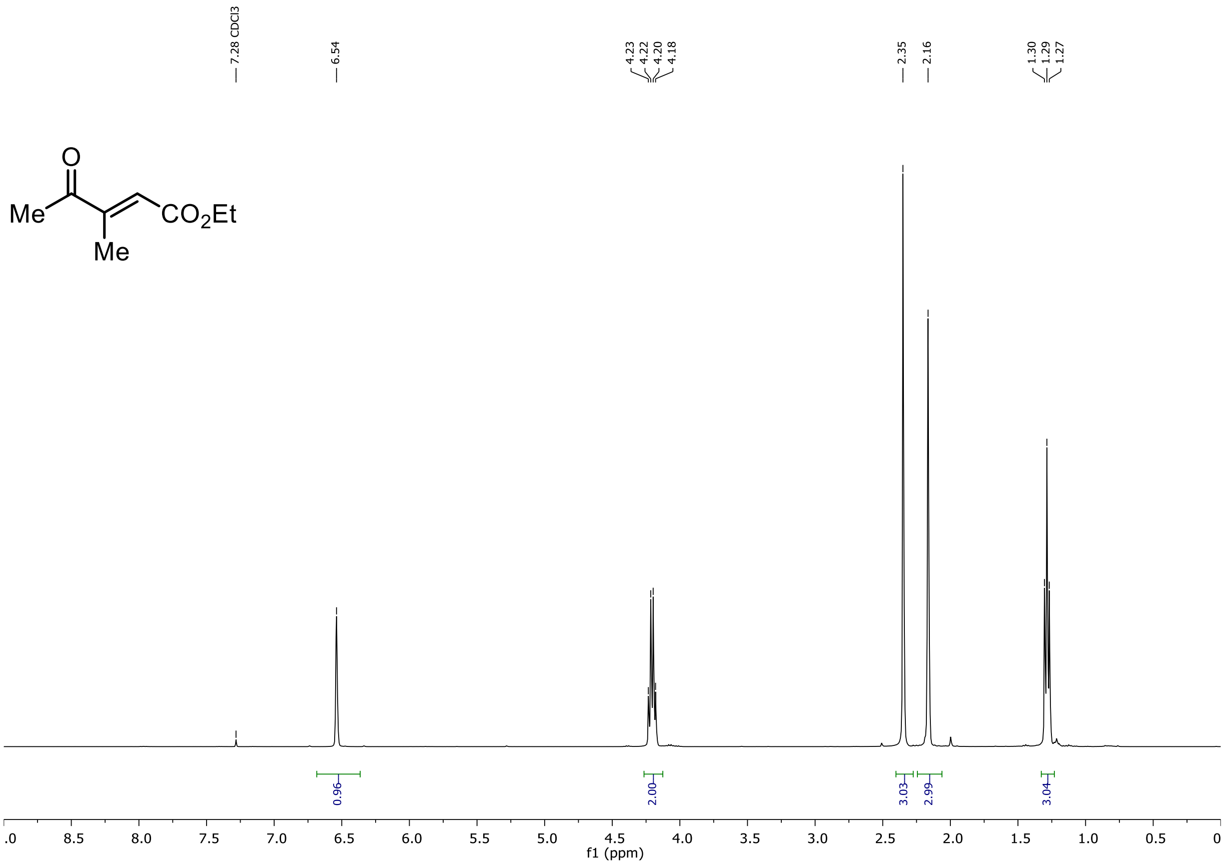

^{13}C NMR

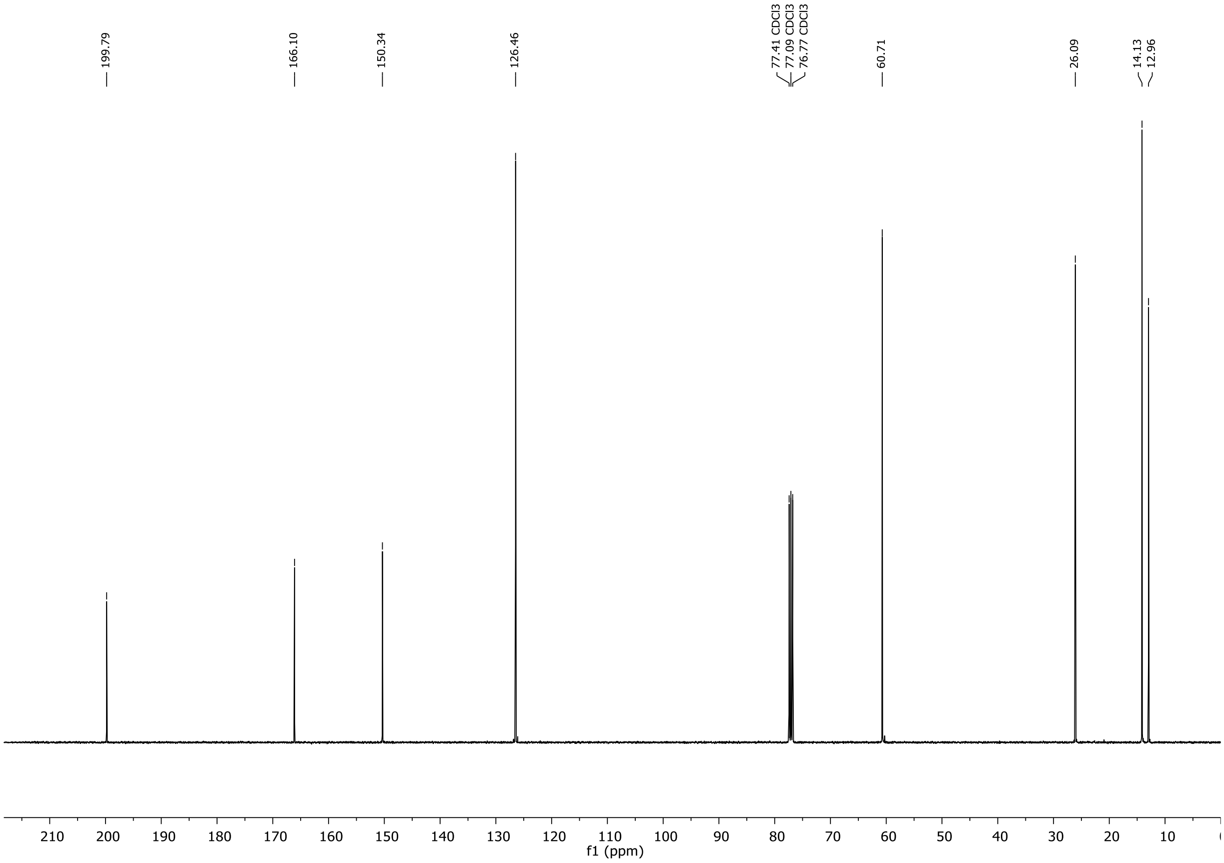

Ethyl (2*E*,4*Z*)-5-cyano-3-methyl-6-phenylhexa-2,4-dienoate (1a)

^{1}H NMR (*dr*=90:10)

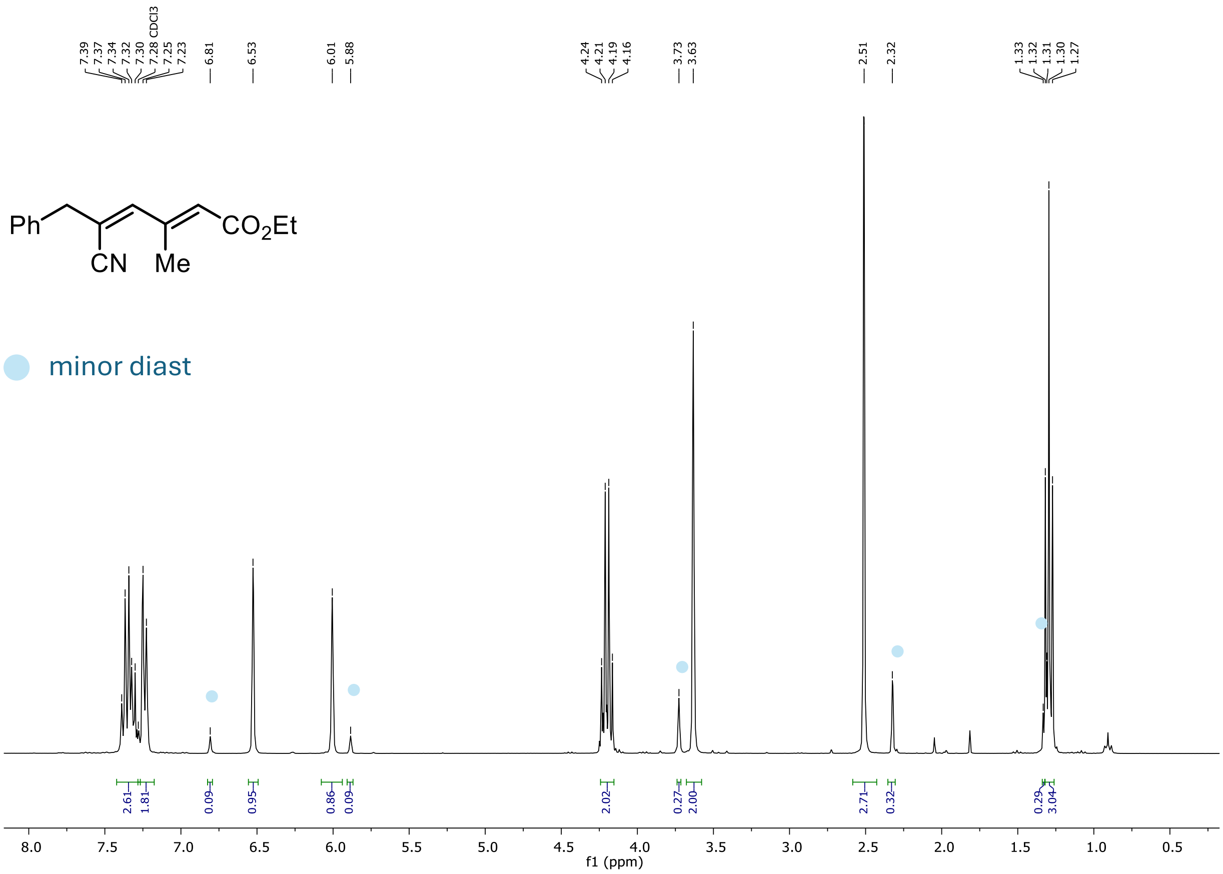

^{13}C NMR

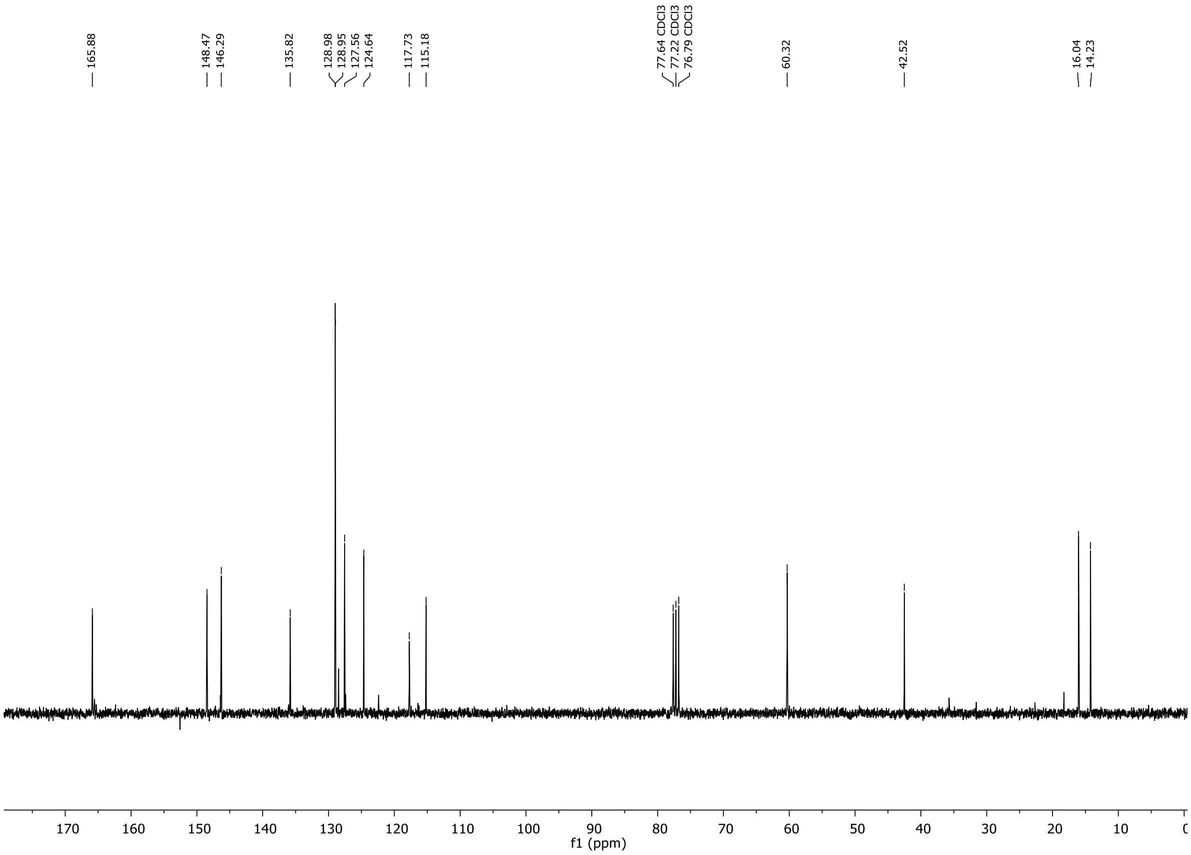

Ethyl (2*E*,4*Z*)-6-(2-bromophenyl)-5-cyano-3-methylhexa-2,4-dienoate (1b)

^{1}H NMR (*dr*>20:1)

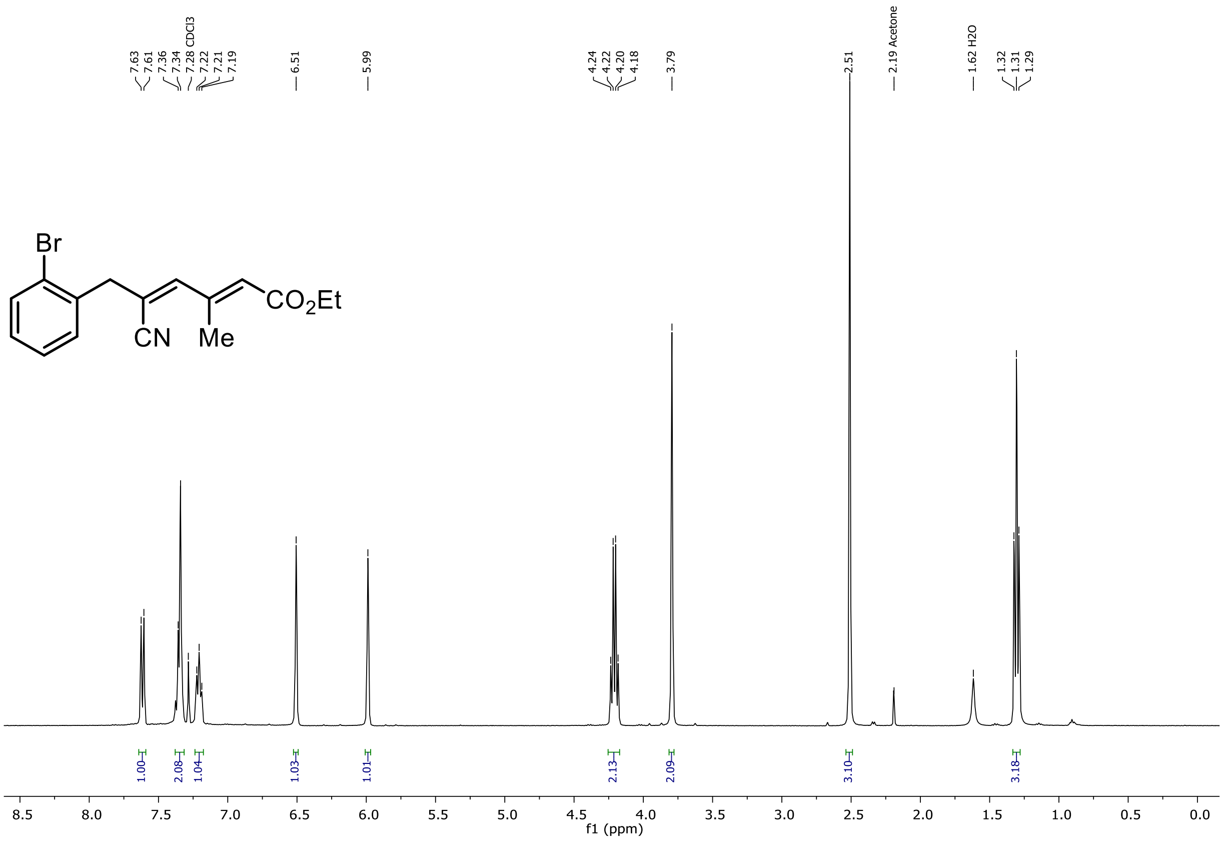

^{13}C NMR

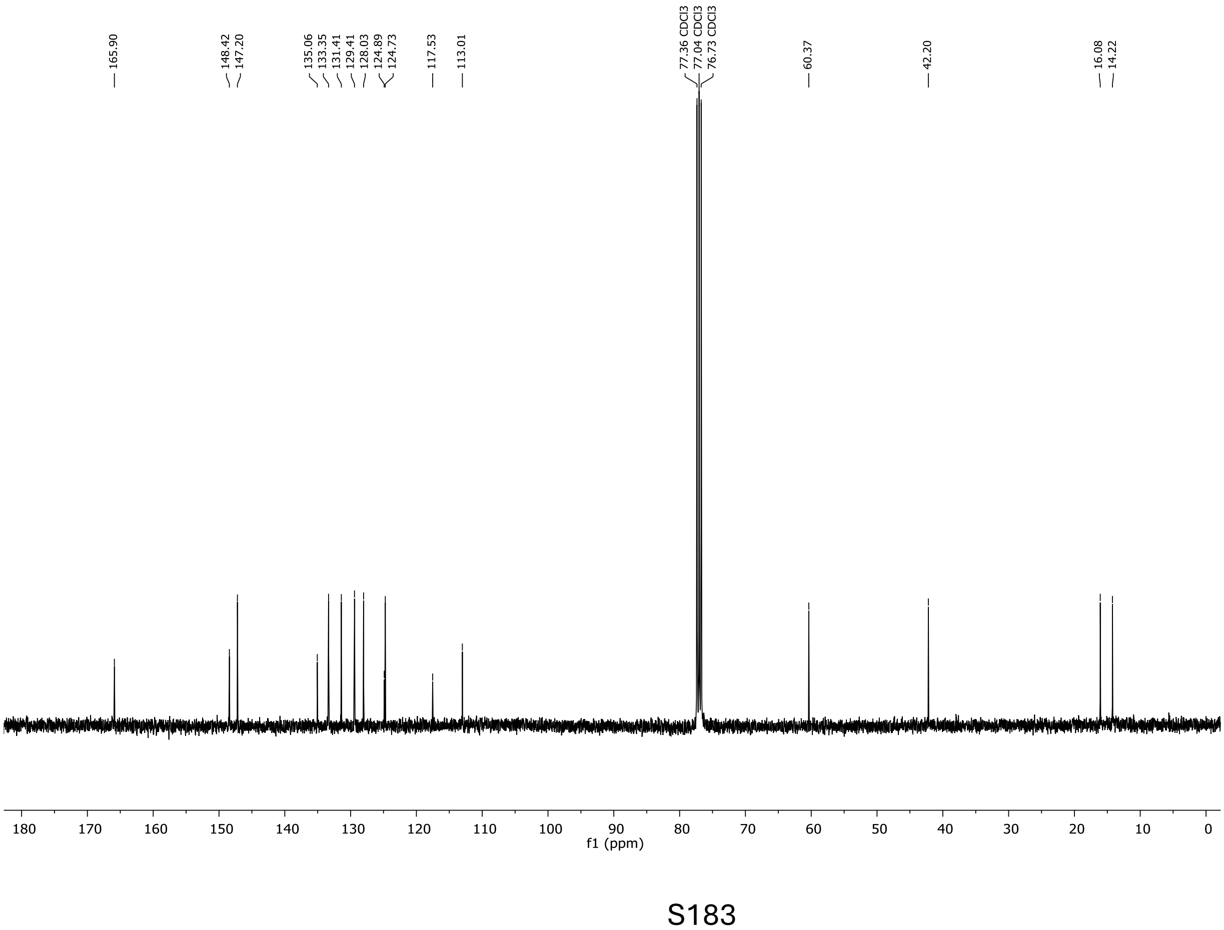

Ethyl (2*E*,4*Z*)-5-cyano-3-methyl-6-(6-(trifluoromethyl)pyridin-3-yl)hexa-2,4-dienoate (1c)

^{1}H NMR (*dr*>20:1)

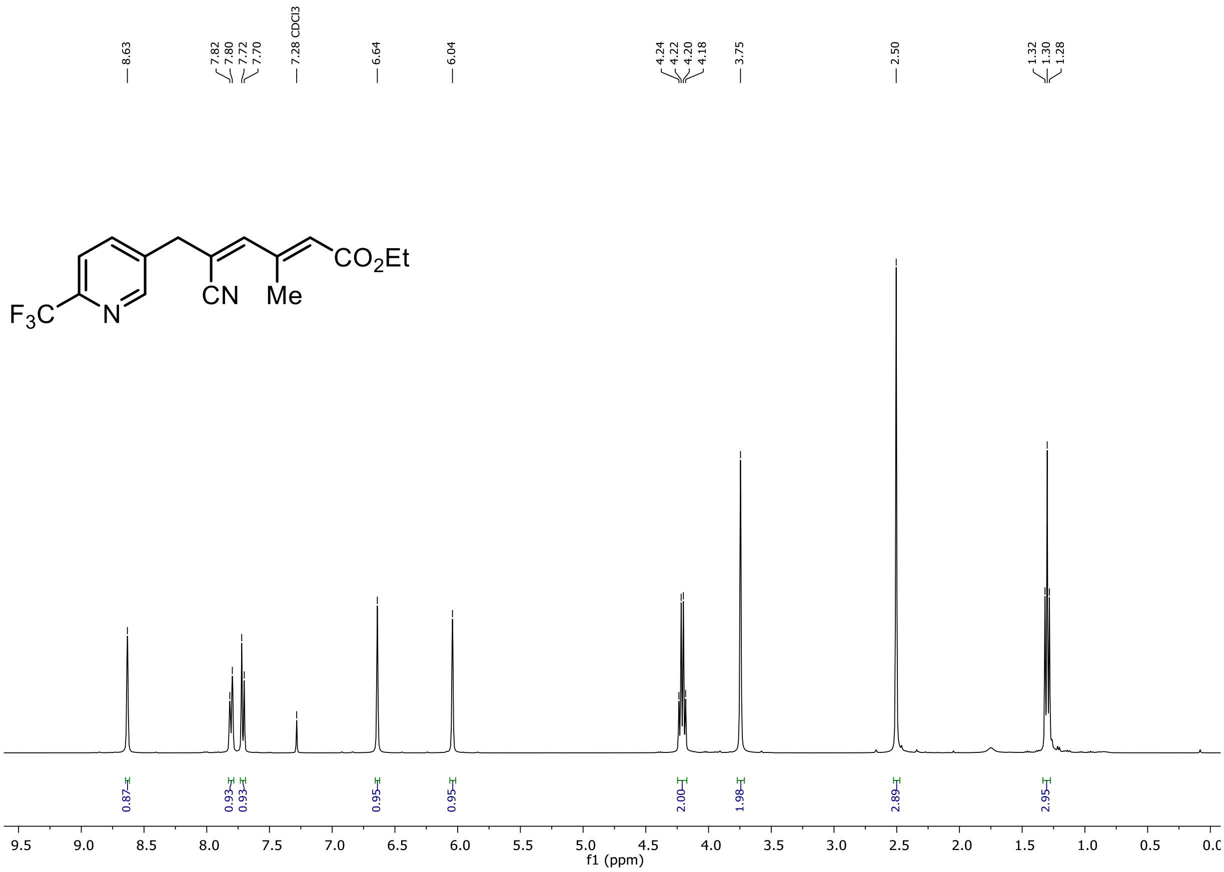

^{13}C NMR

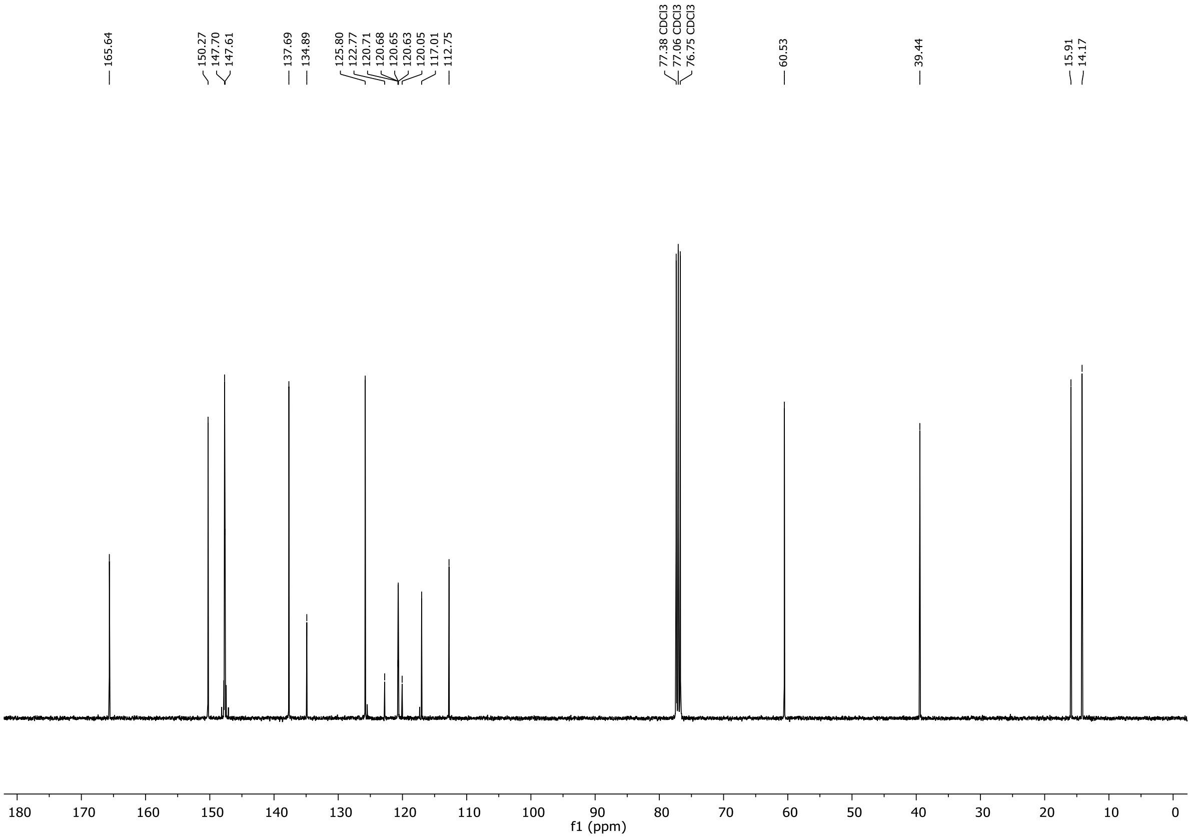

19F NMR

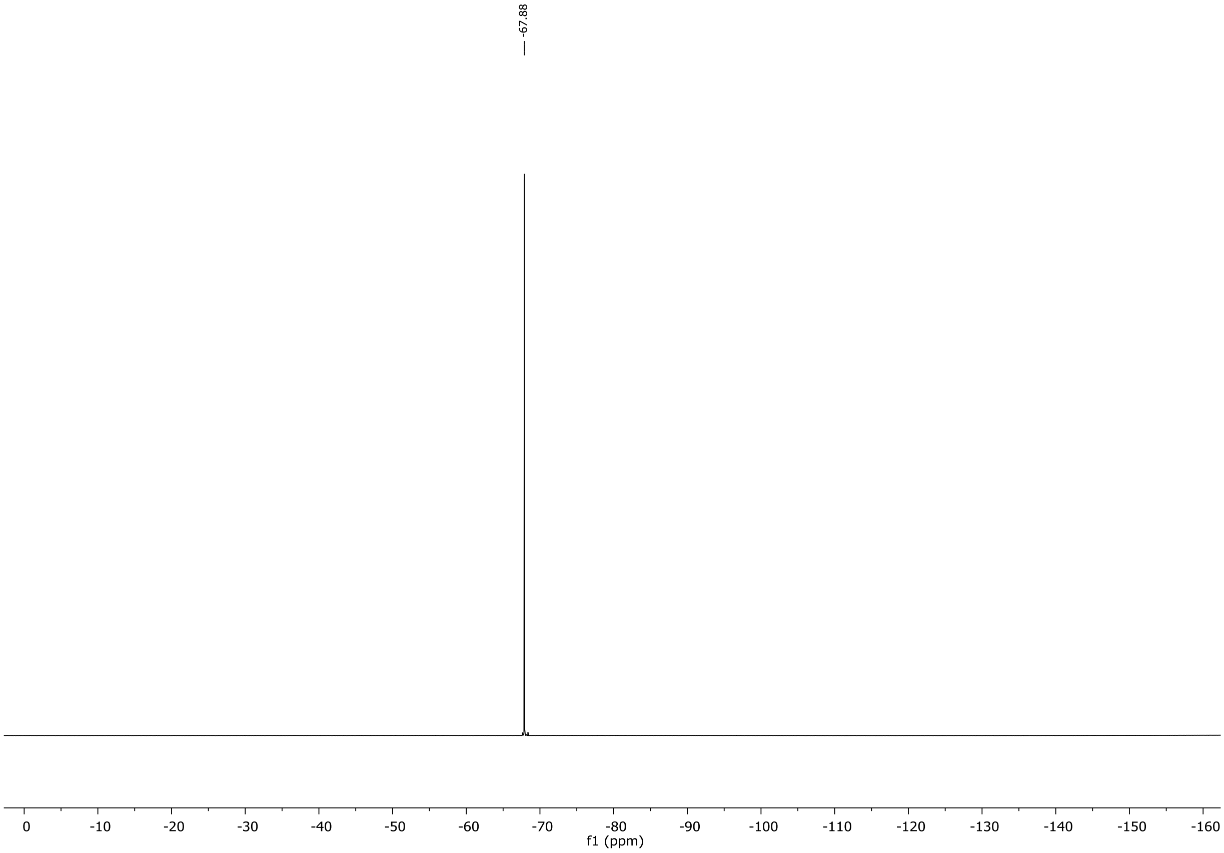

Ethyl (2*E*,4*Z*)-5-cyano-3-methyl-6-(thiophen-3-yl)hexa-2,4-dienoate (1d)

1H NMR (*dr*=69:31)

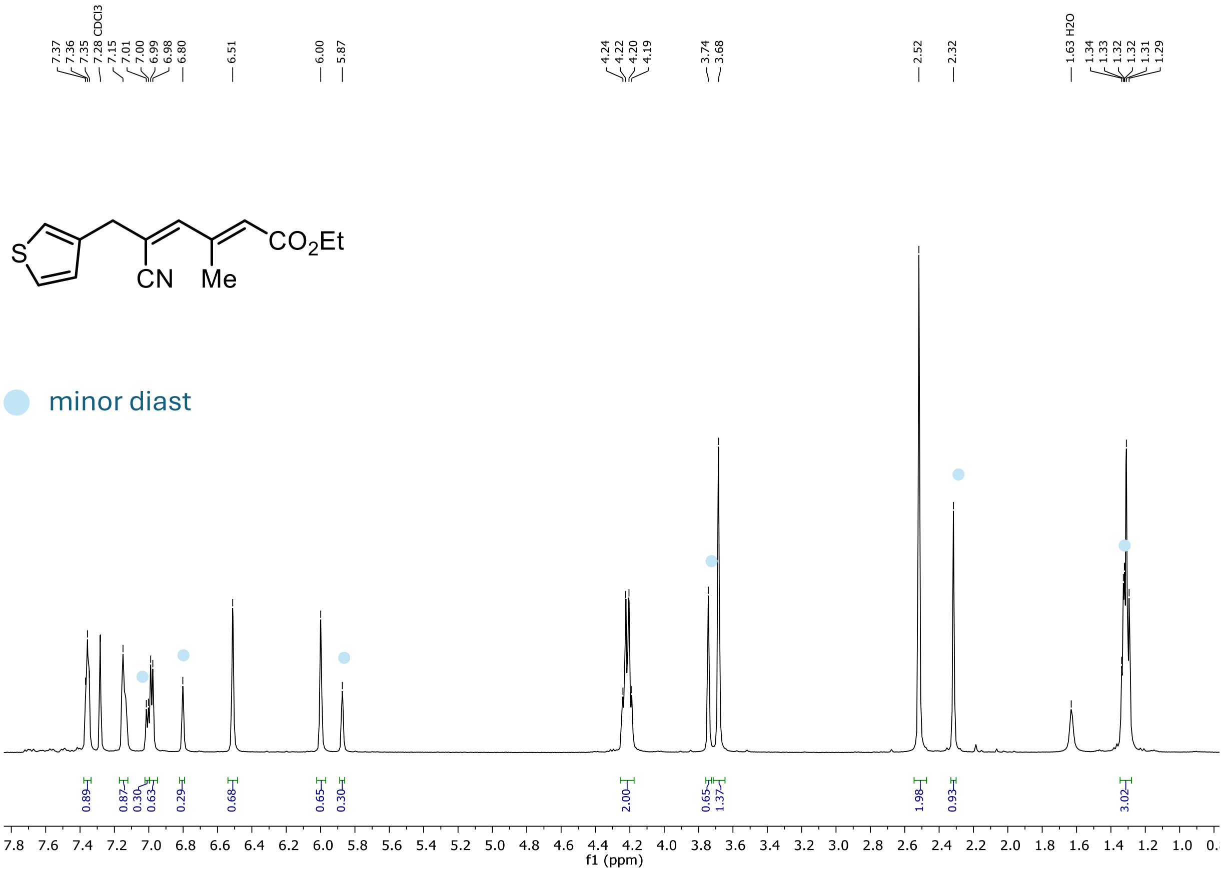

^{13}C NMR

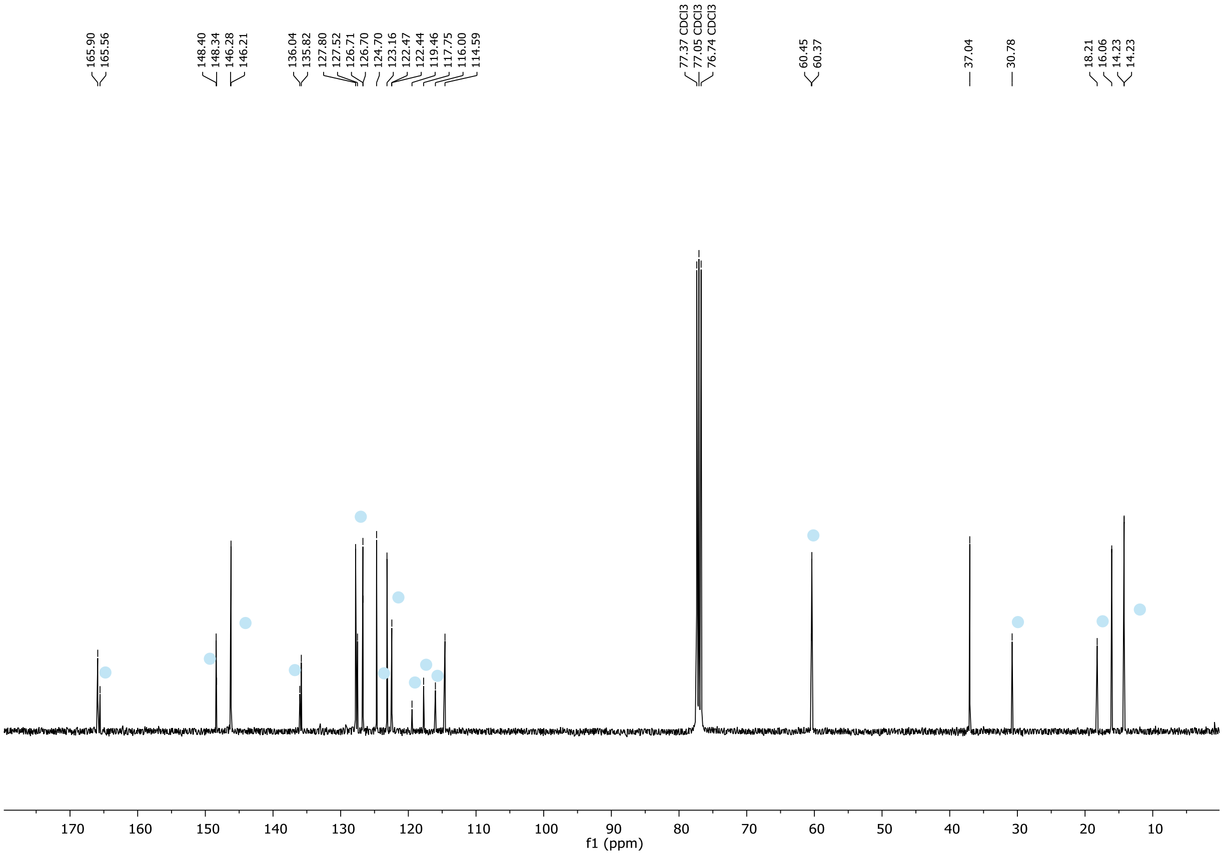

***Tert*-butyl 3-((2*Z*,4*E*)-2-cyano-6-ethoxy-4-methyl-6-oxohexa-2,4-dien-1-yl)-1H-indole-1-carboxylate (1e)**

^{1}H NMR (*dr*=83:17)

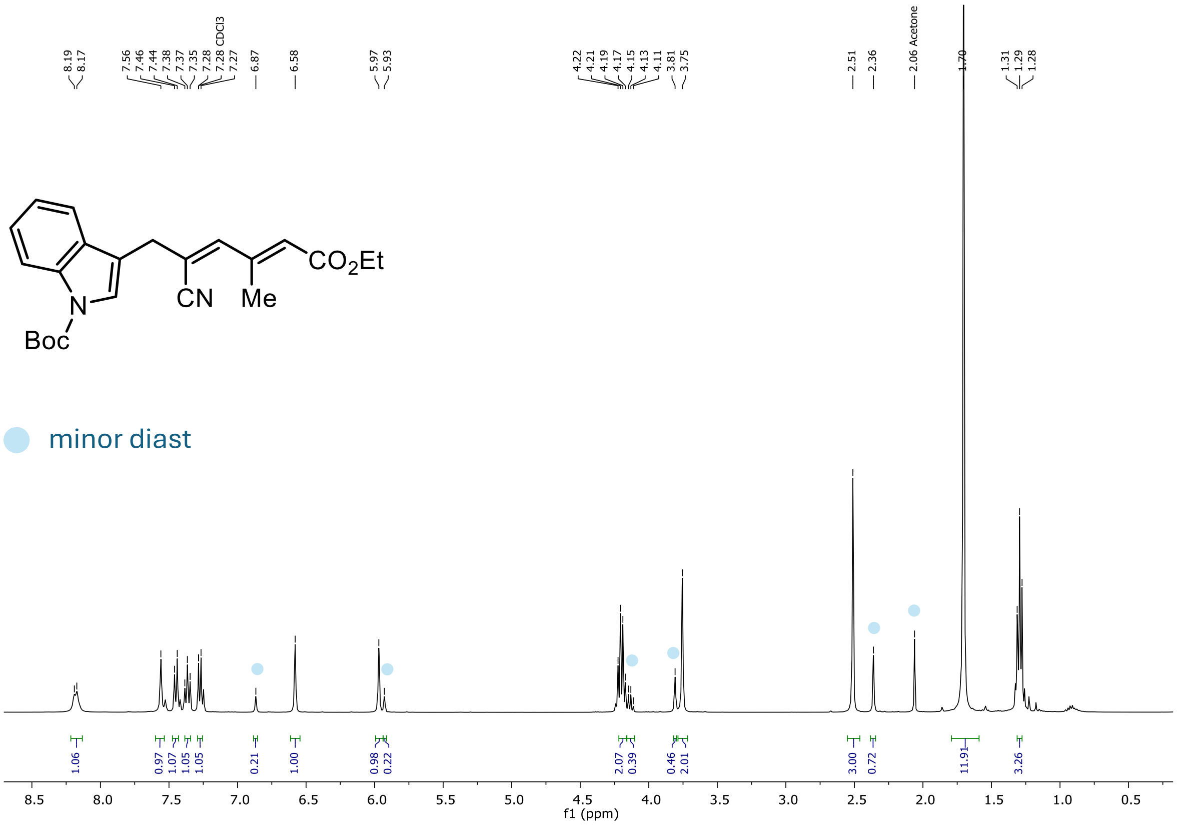

^{13}C NMR

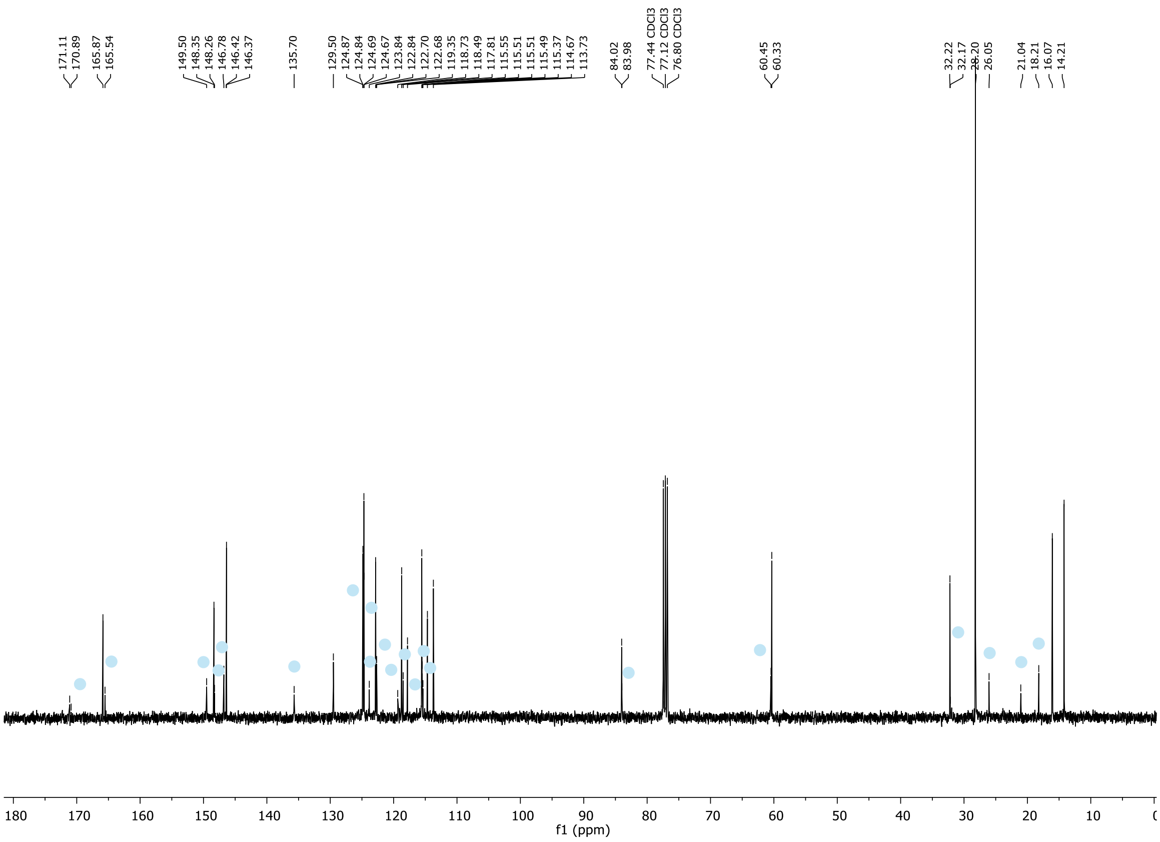

Ethyl (2*E*,4*Z*)-5-cyano-3-methyl-6,6-diphenylhexa-2,4-dienoate (1f)

^{1}H NMR (*dr*>20:1)

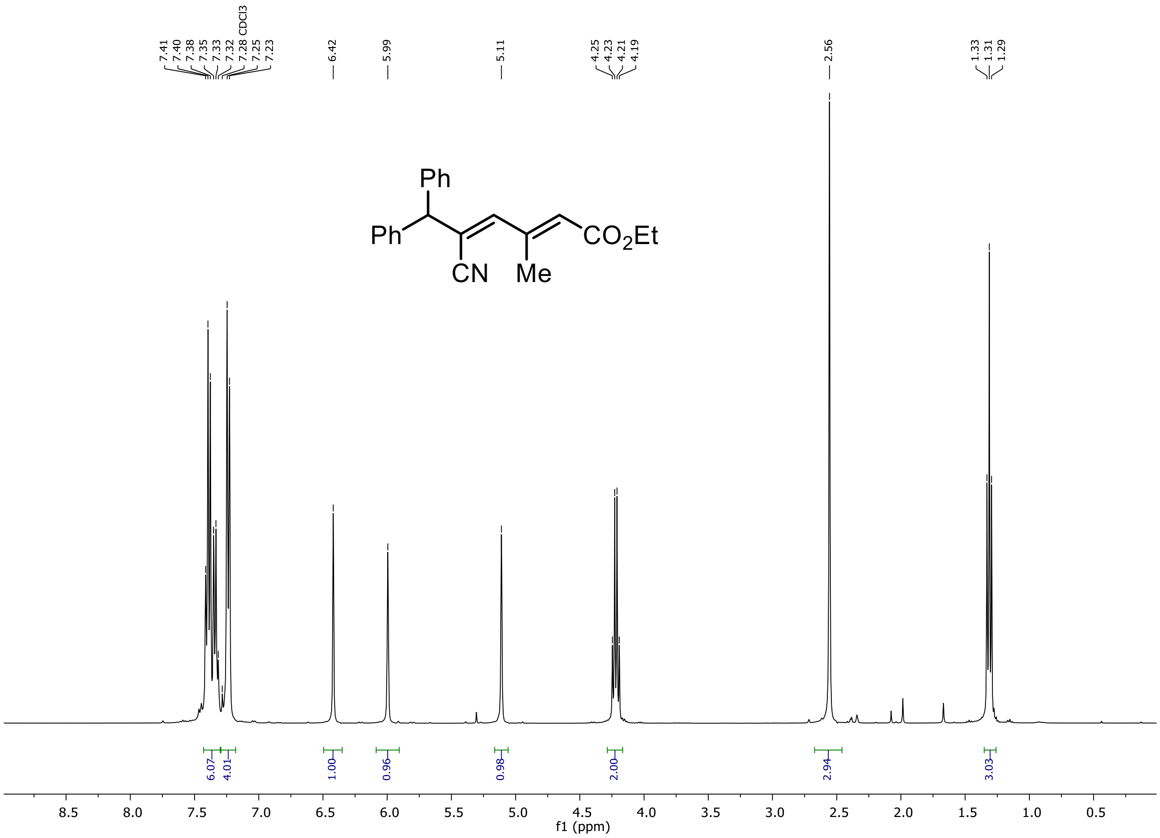

^{13}C NMR

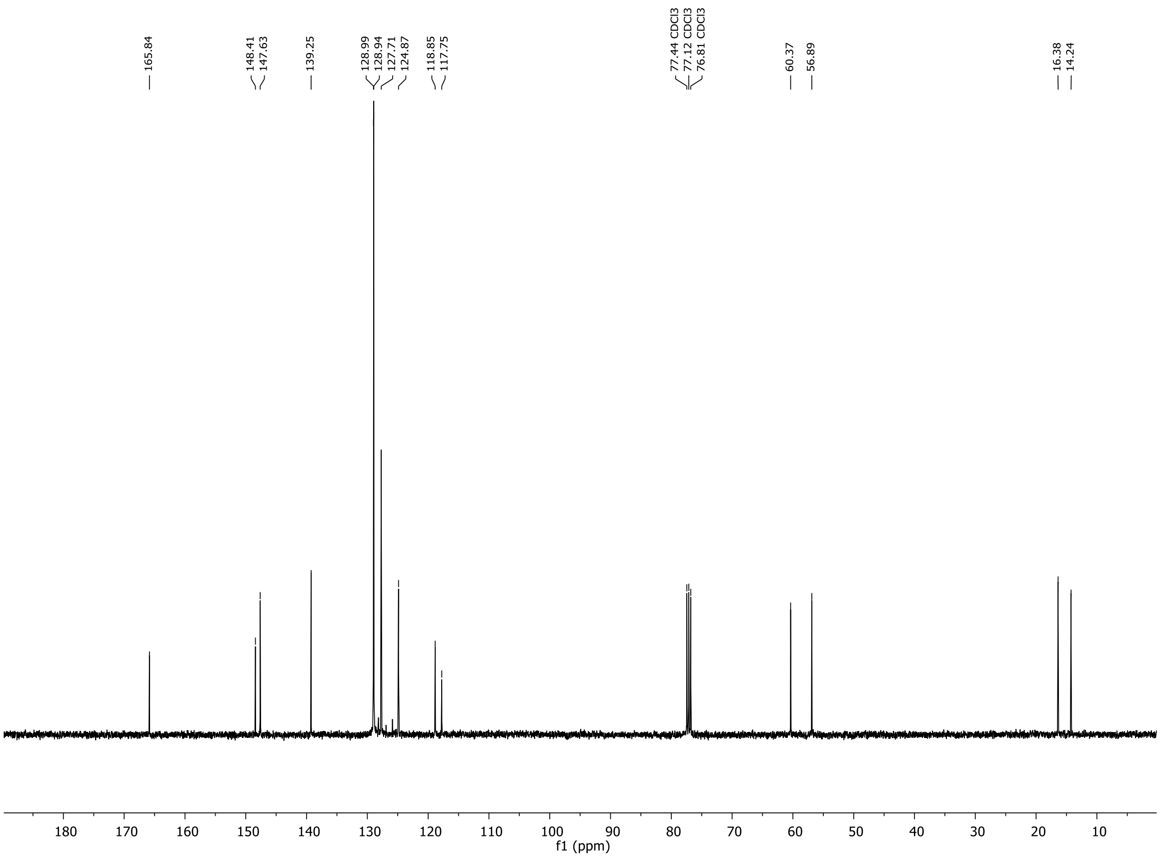

Ethyl (2*E*,4*Z*)-6,6-bis(4-bromophenyl)-5-cyano-3-methylhexa-2,4-dienoate (1g)

^{1}H NMR

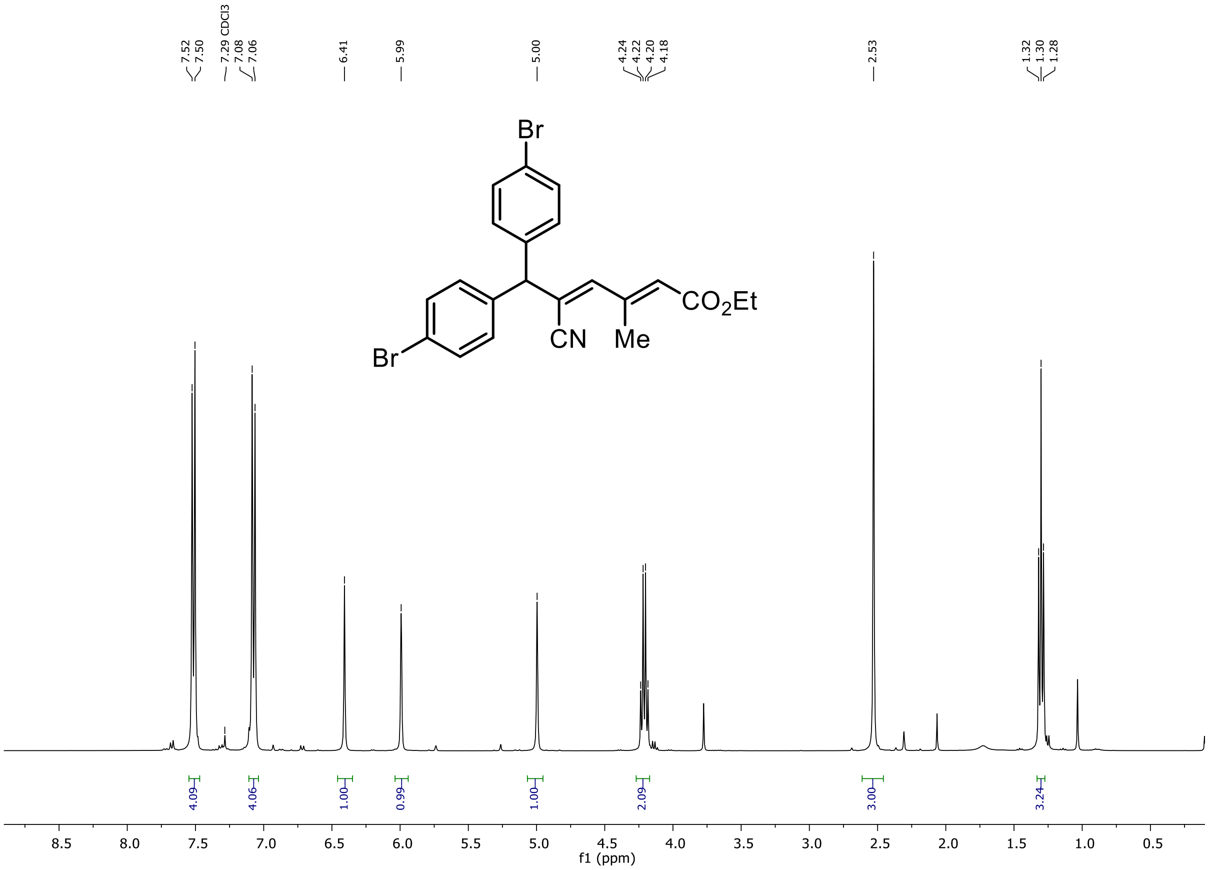

^{13}C NMR

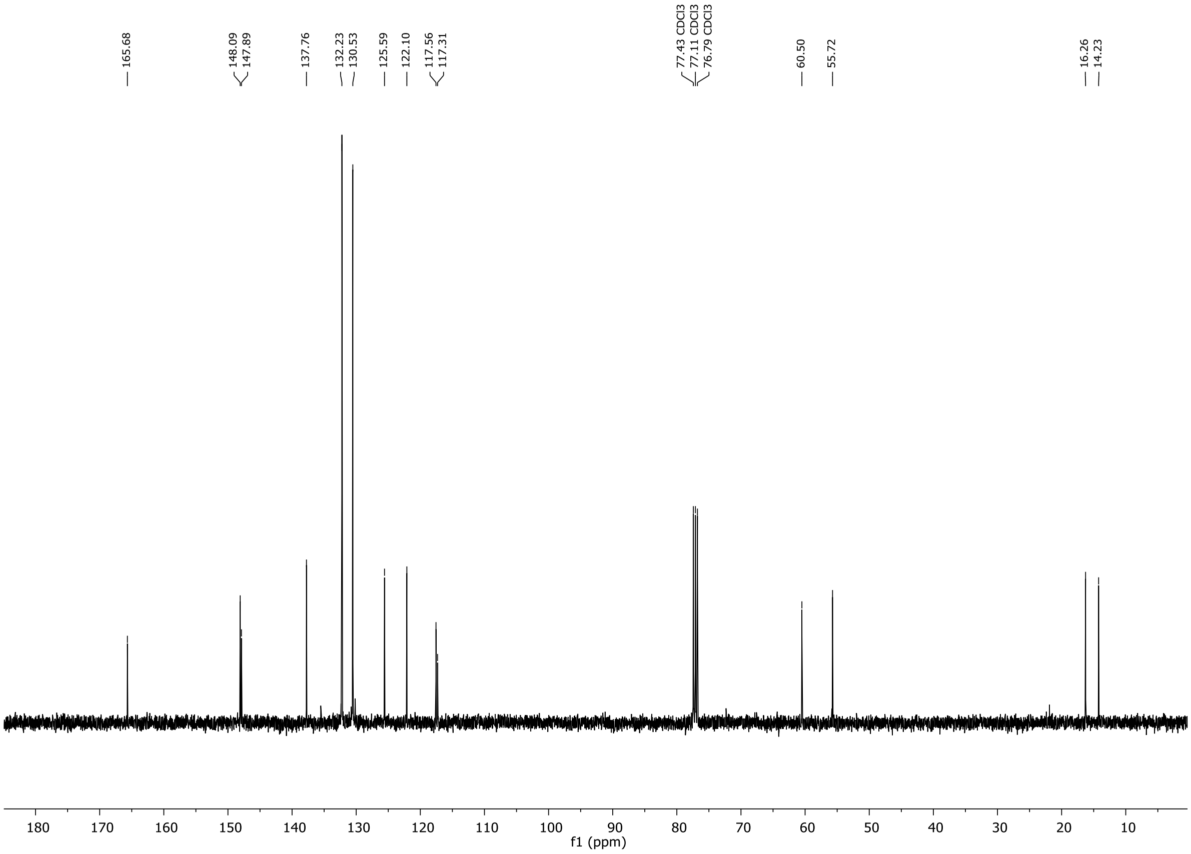

Ethyl (2*E*,4*Z*)-5-cyano-3,6-dimethylhepta-2,4-dienoate (1h)

^{1}H NMR (*dr*>20:1)

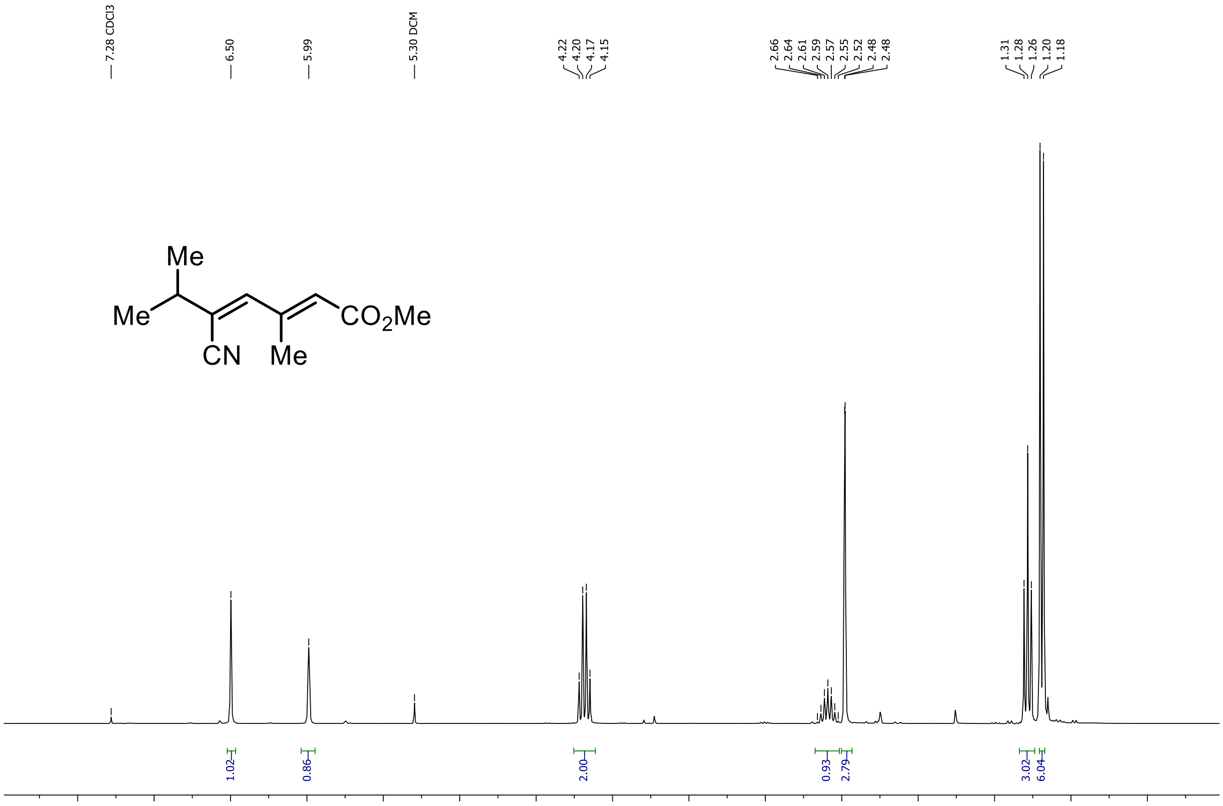

^{13}C NMR

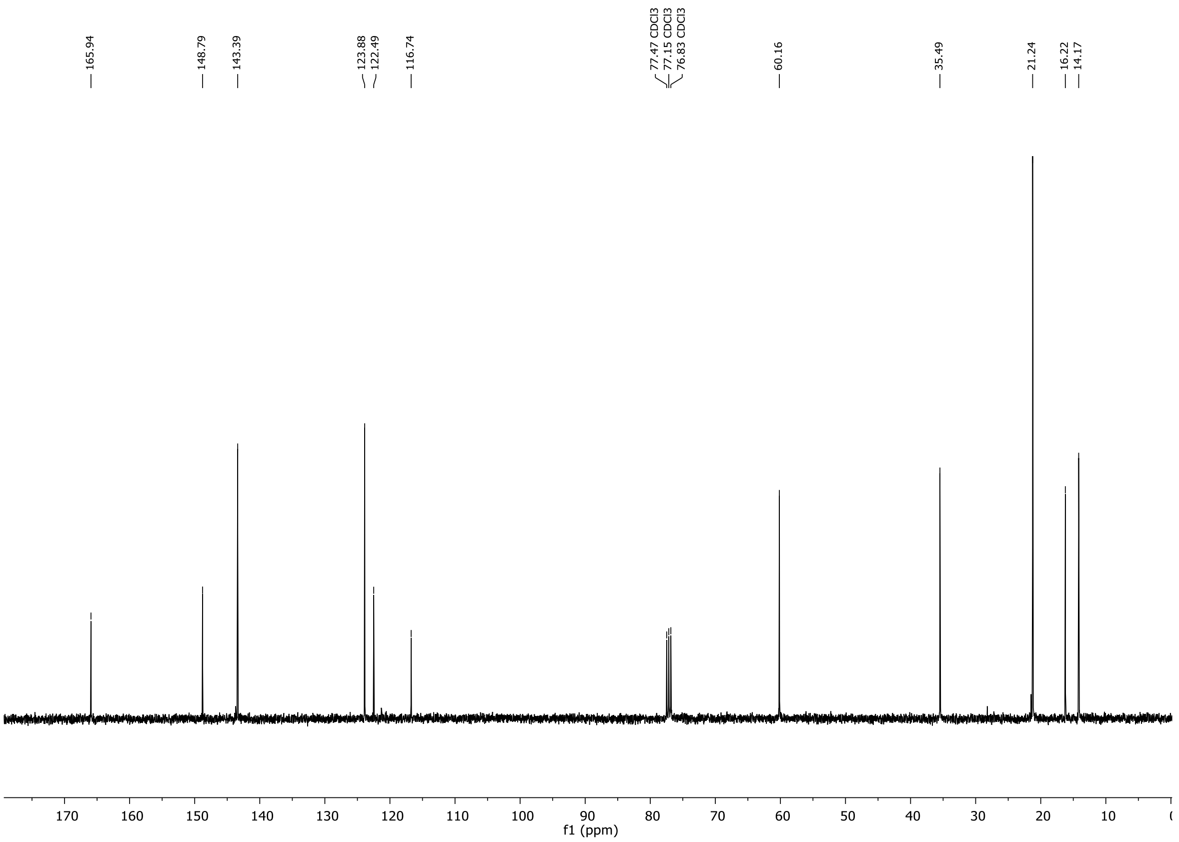

Ethyl (2*E*,4*Z*)-5-cyano-3-methyl-8-phenylocta-2,4-dienoate (1i)

^{1}H NMR (*dr*=78:22)

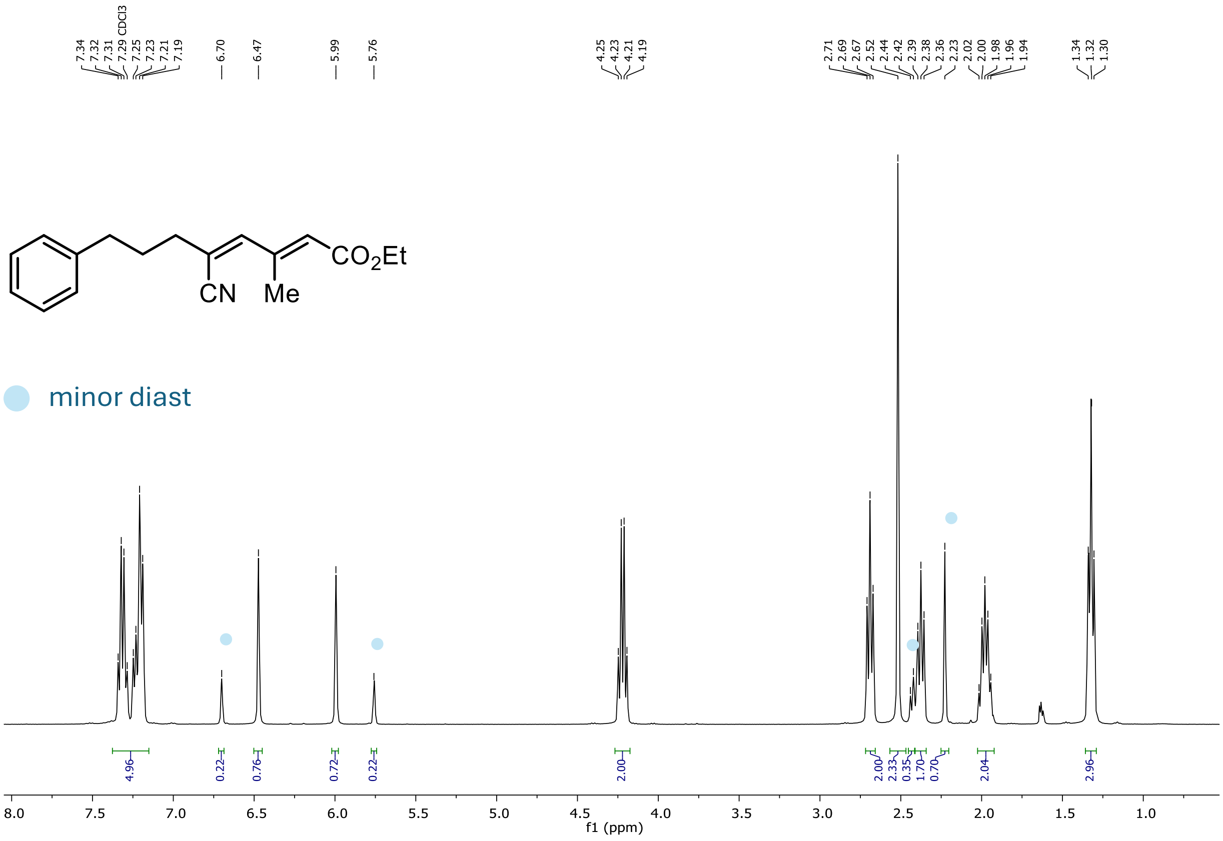

^{13}C NMR

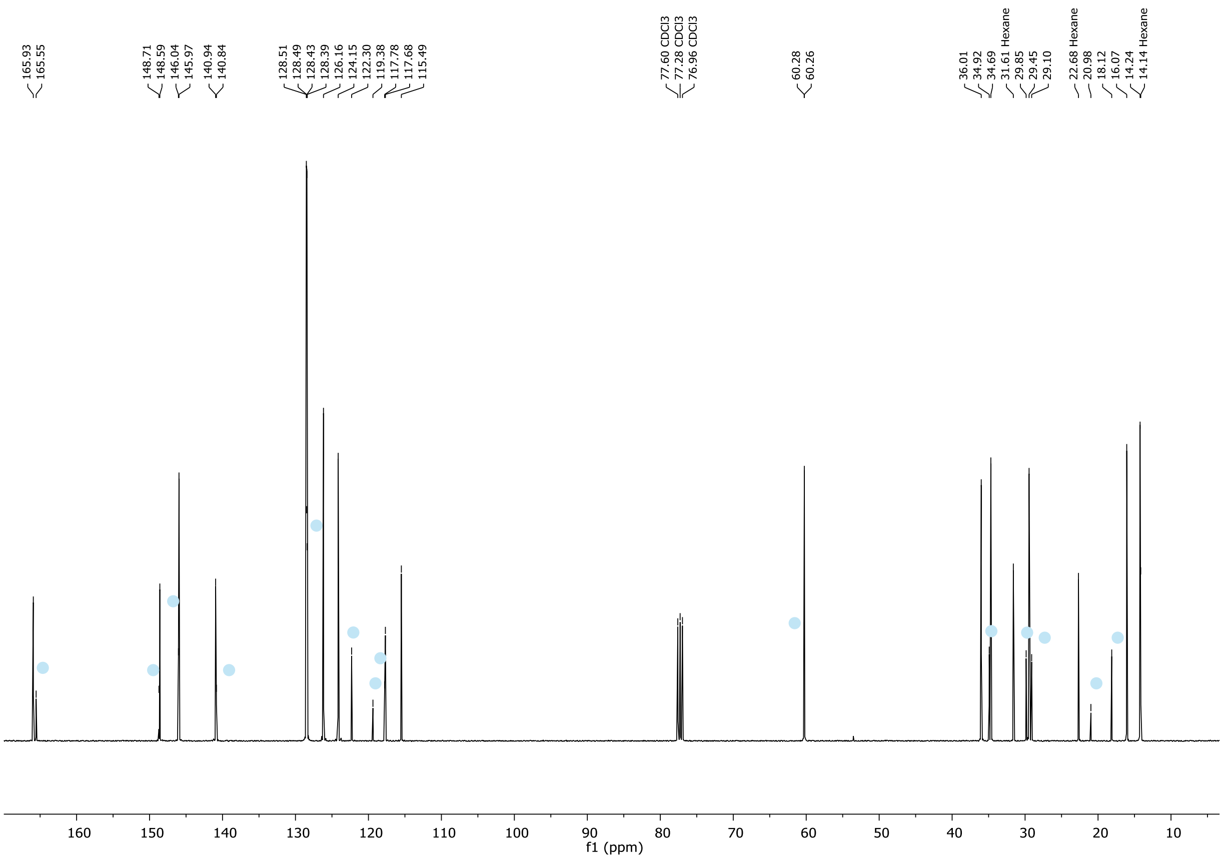

Ethyl (2*E*,4*Z*)-5-cyano-7-(1,3-dioxolan-2-yl)-3-methylhepta-2,4-dienoate (1j)

^{1}H NMR (*dr*>20:1)

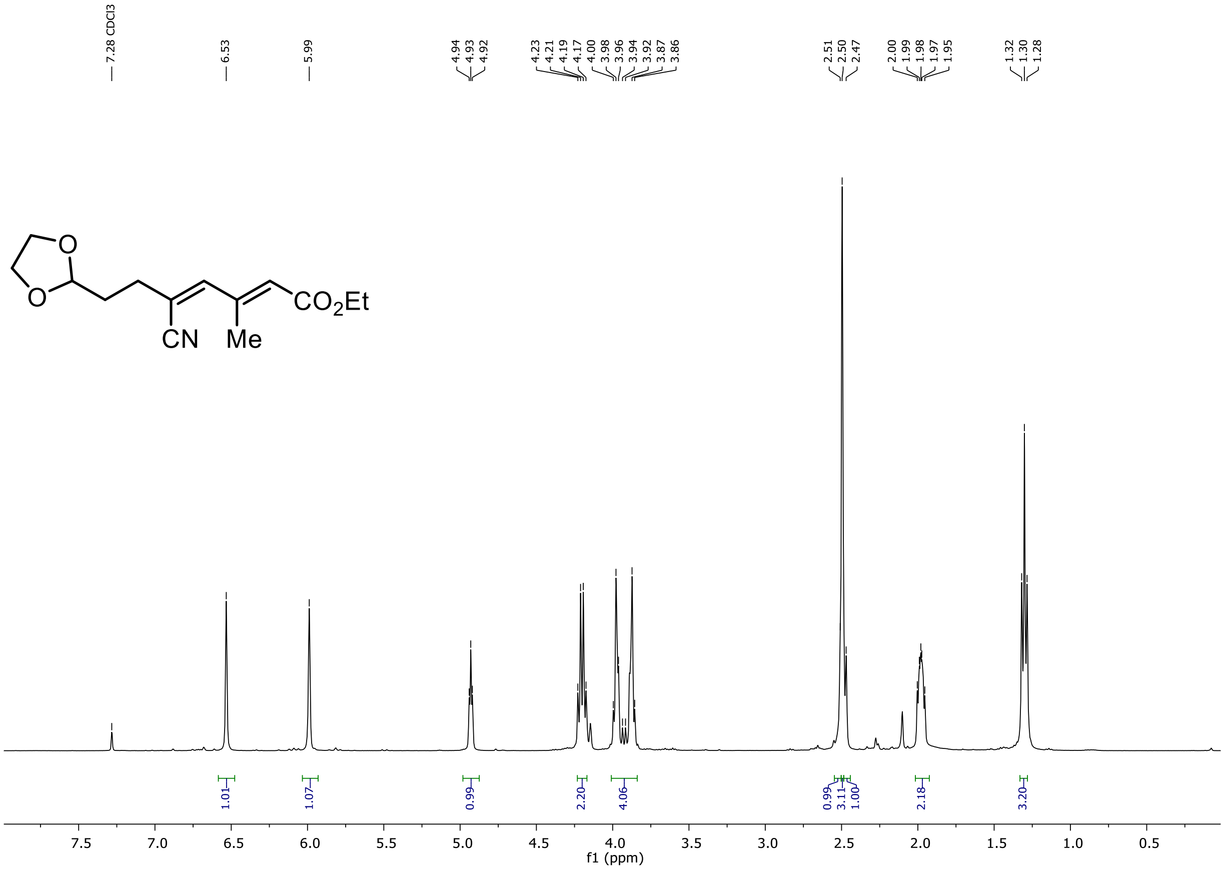

^{13}C NMR

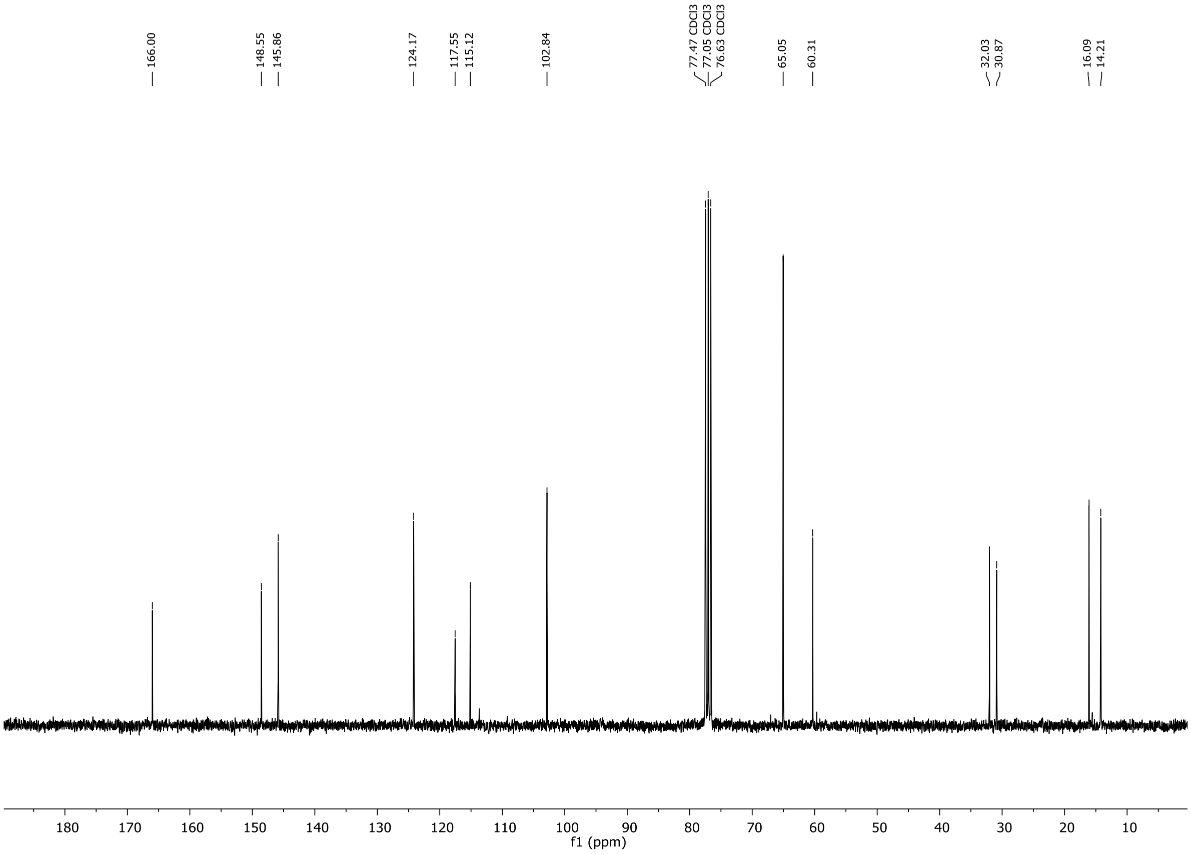

Ethyl (2*E*,4*Z*)-7-((tert-butyldimethylsilyl)oxy)-5-cyano-3-methylhepta-2,4-dienoate (1k)

^{1}H NMR (*dr*>20:1)

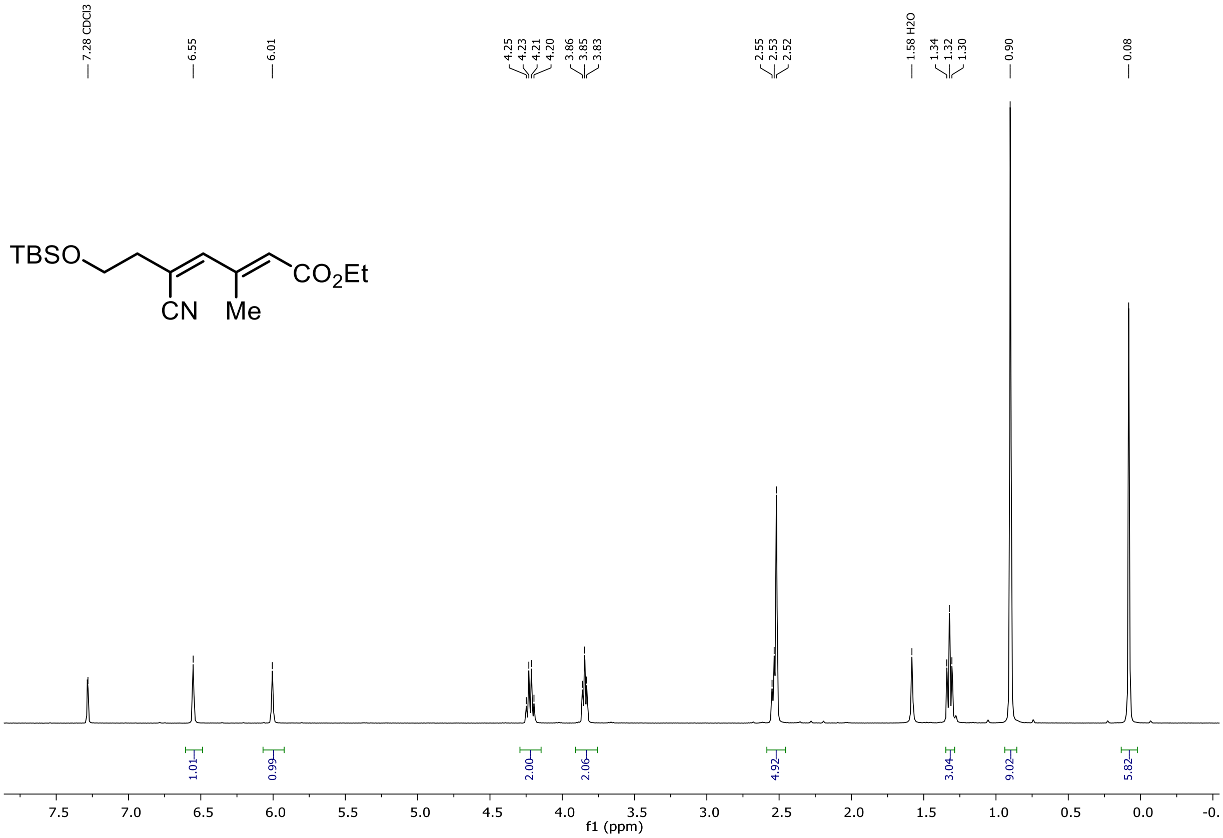

^{13}C NMR

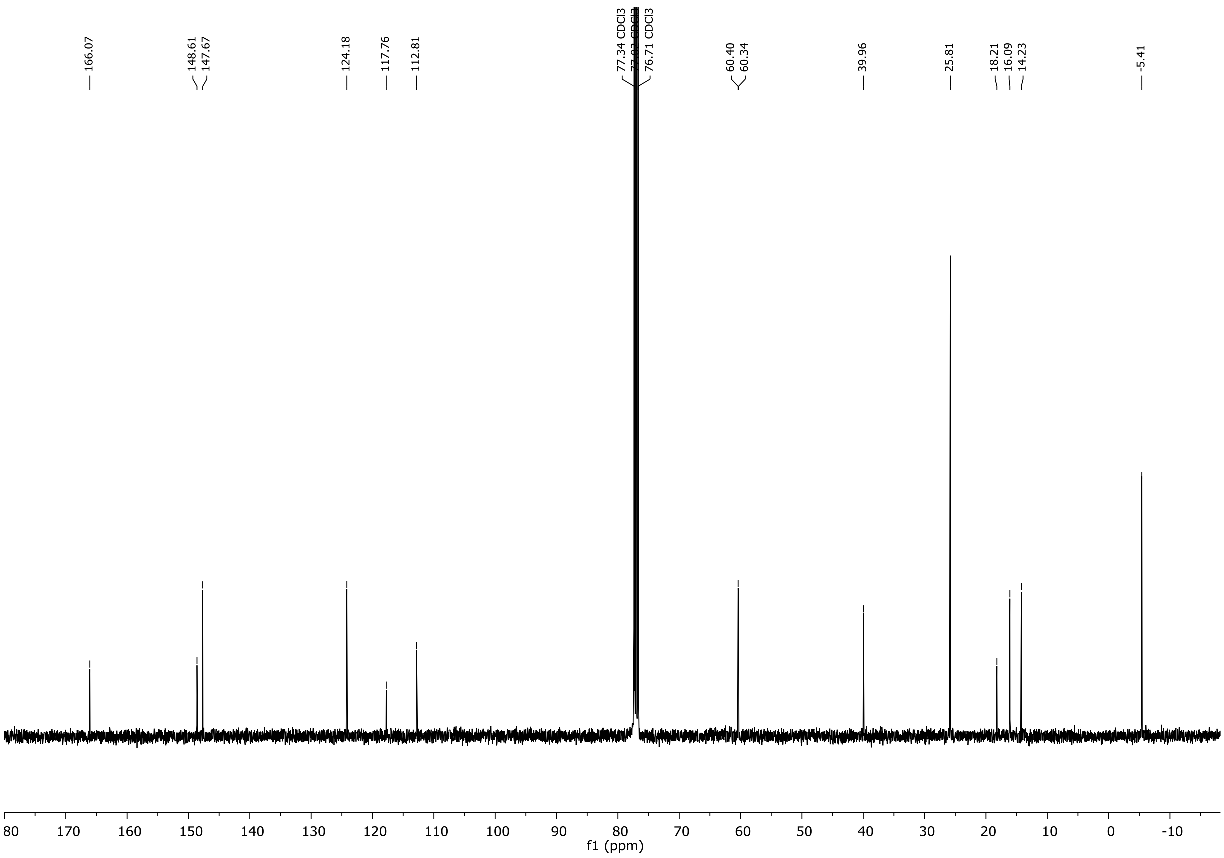

Ethyl (2*E*,4*Z*)-5-cyano-3-methylocta-2,4,7-trienoate (1l)

^1H NMR (*dr*=92:8)

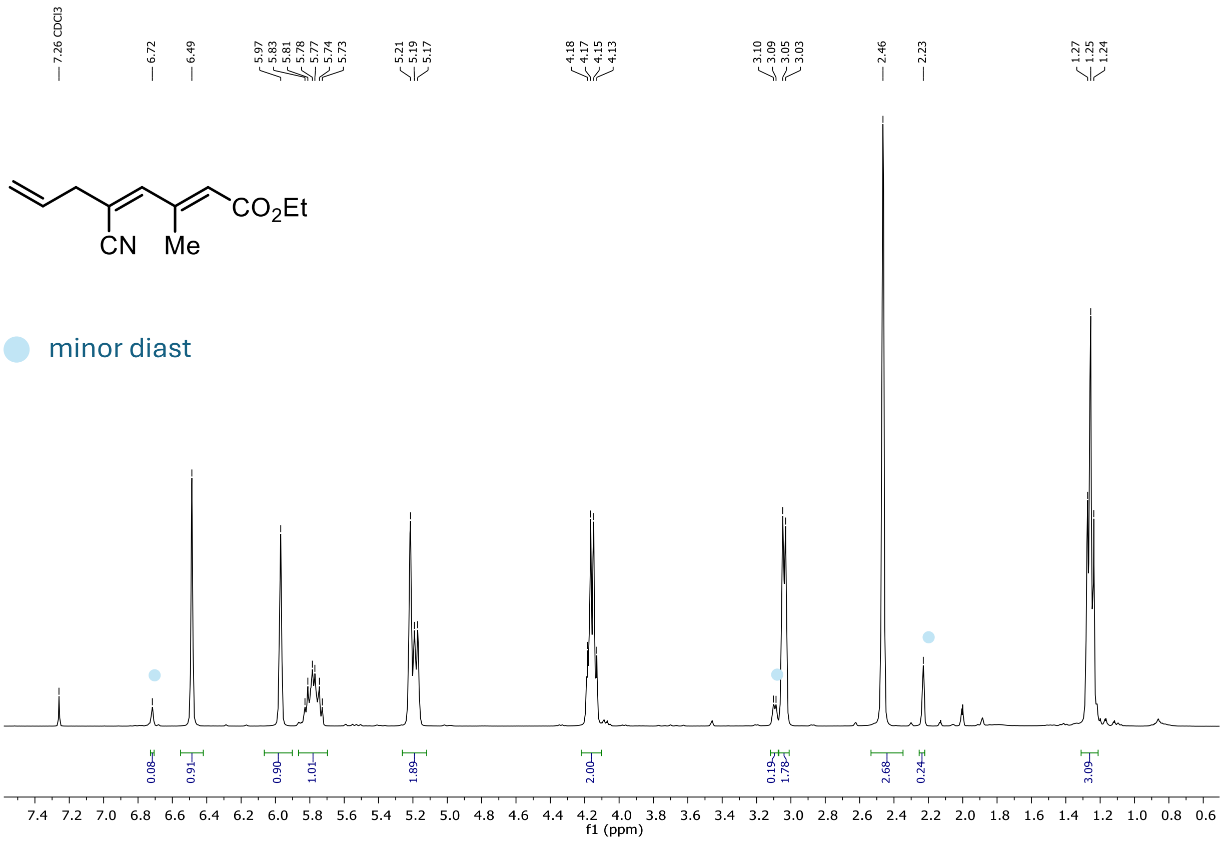

^{13}C NMR

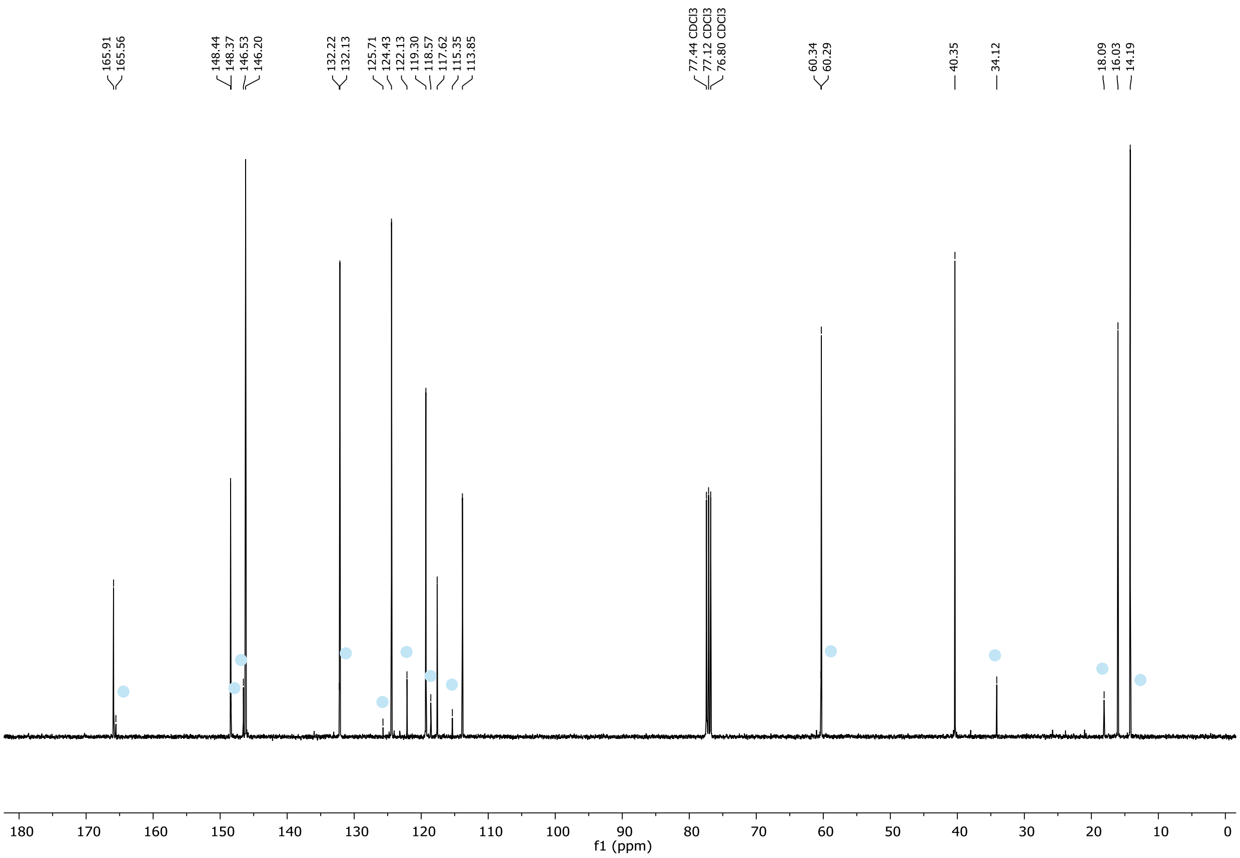

Ethyl (2*E*,4*Z*)-5-cyano-3-methylhexa-2,4-dienoate (1m)

^{1}H NMR (*dr*>20:1)

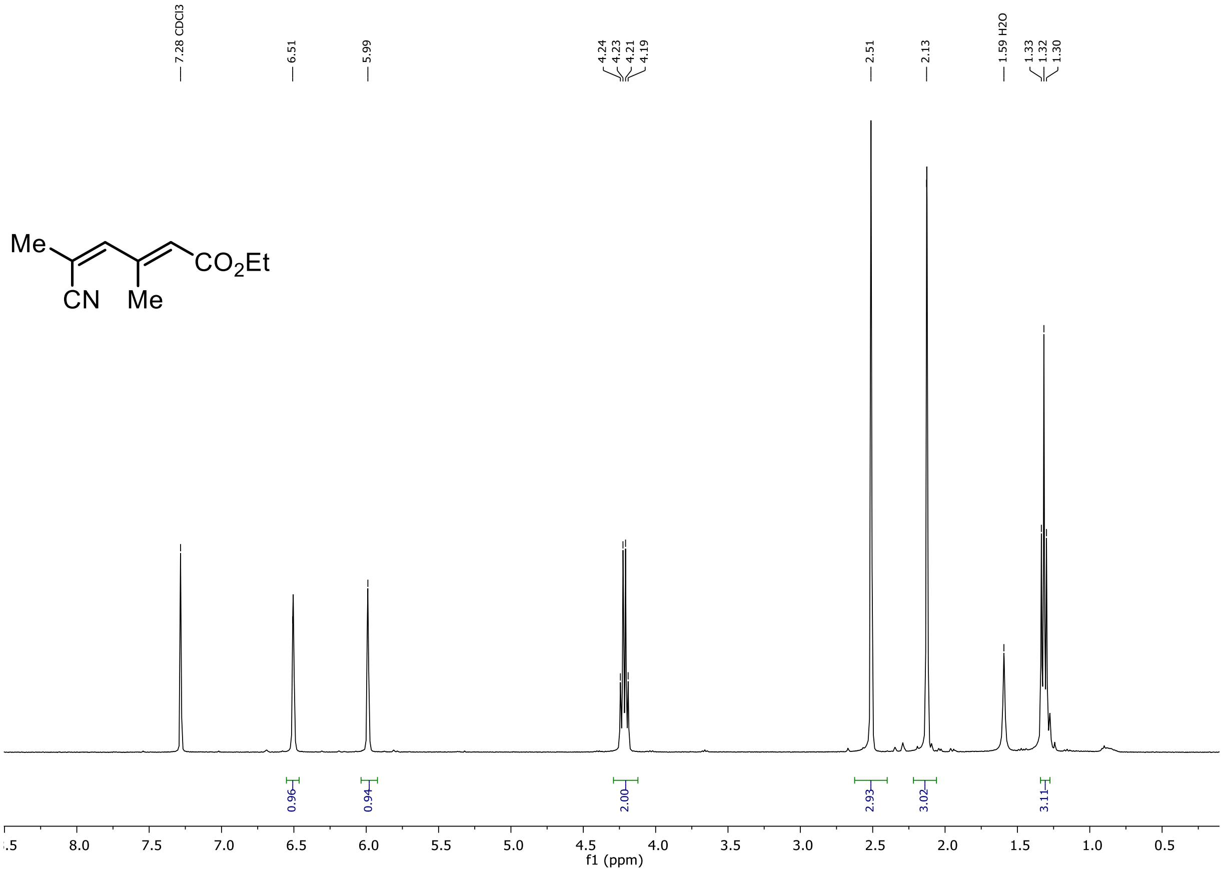

^{13}C NMR

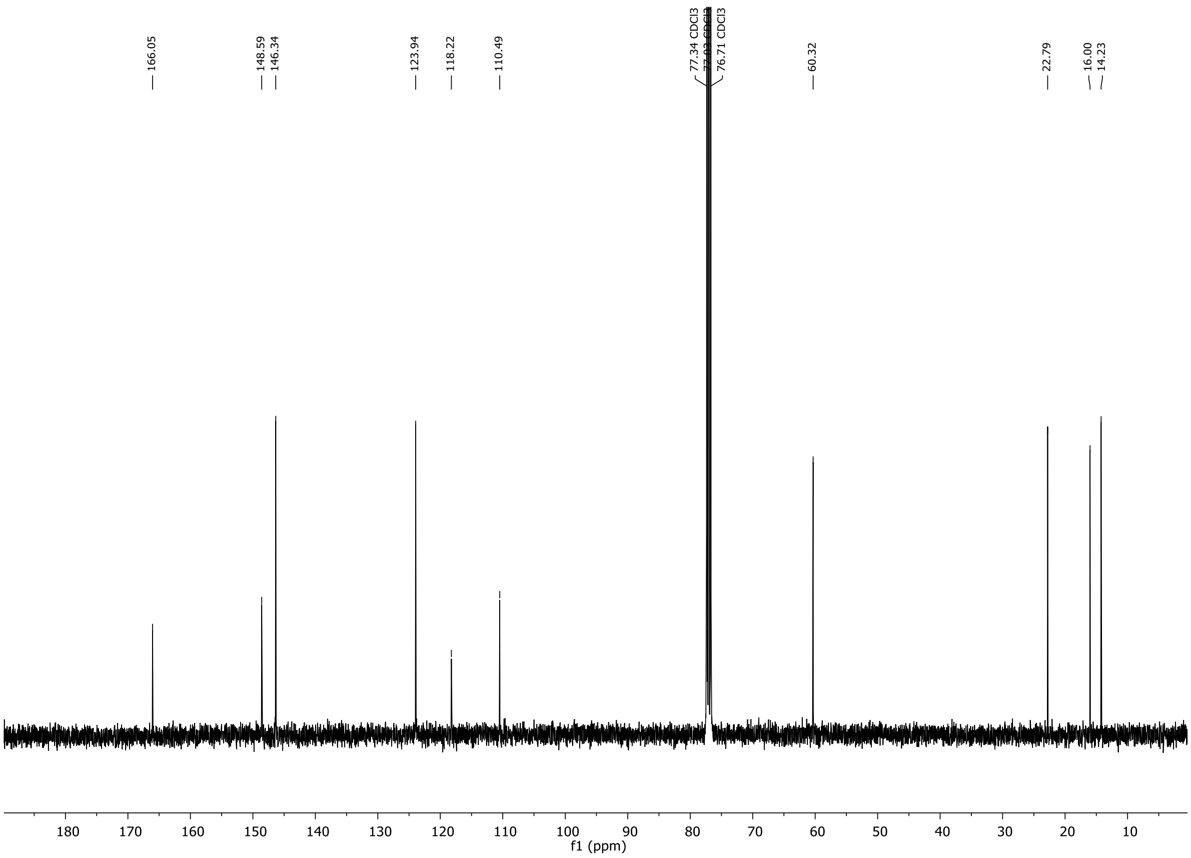

Ethyl (2*E*,4*Z*)-5-cyano-3-methyl-5-phenylpenta-2,4-dienoate (1n)

^{1}H NMR (*dr*=83:17)

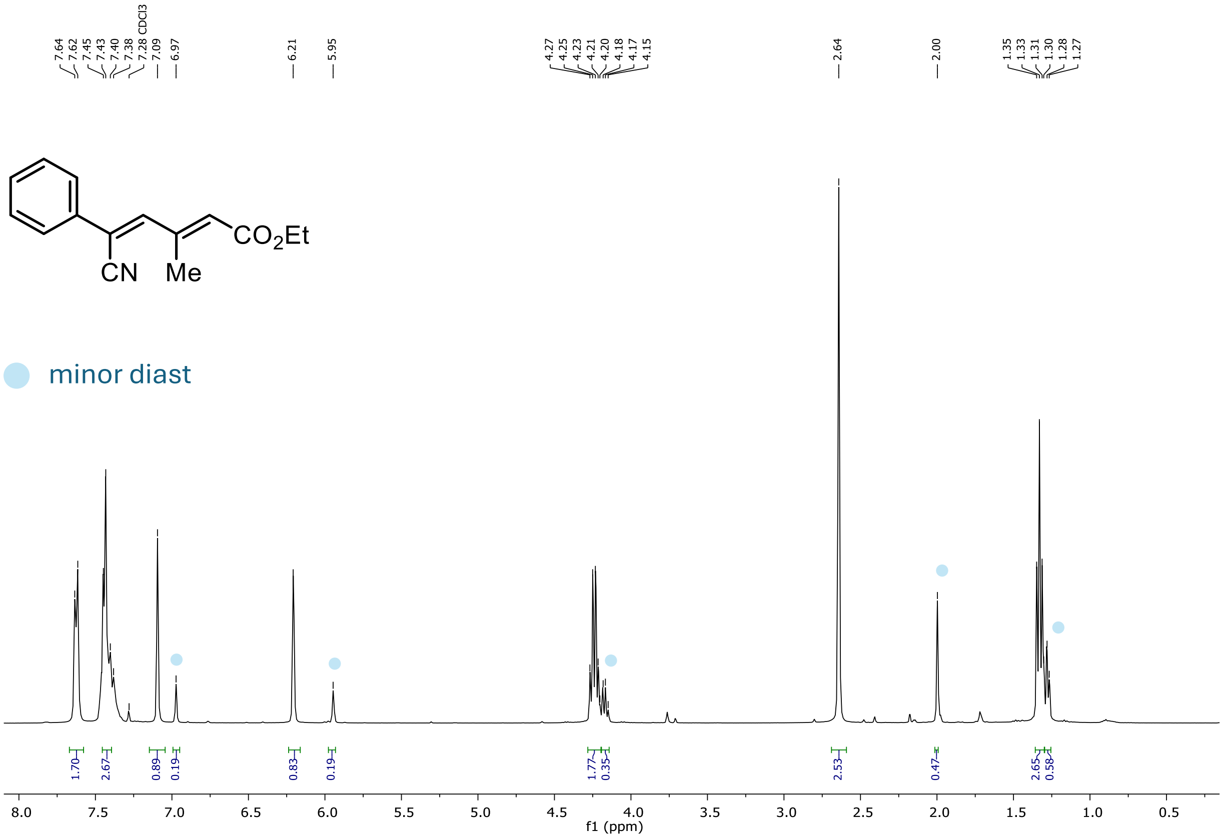

^{13}C NMR

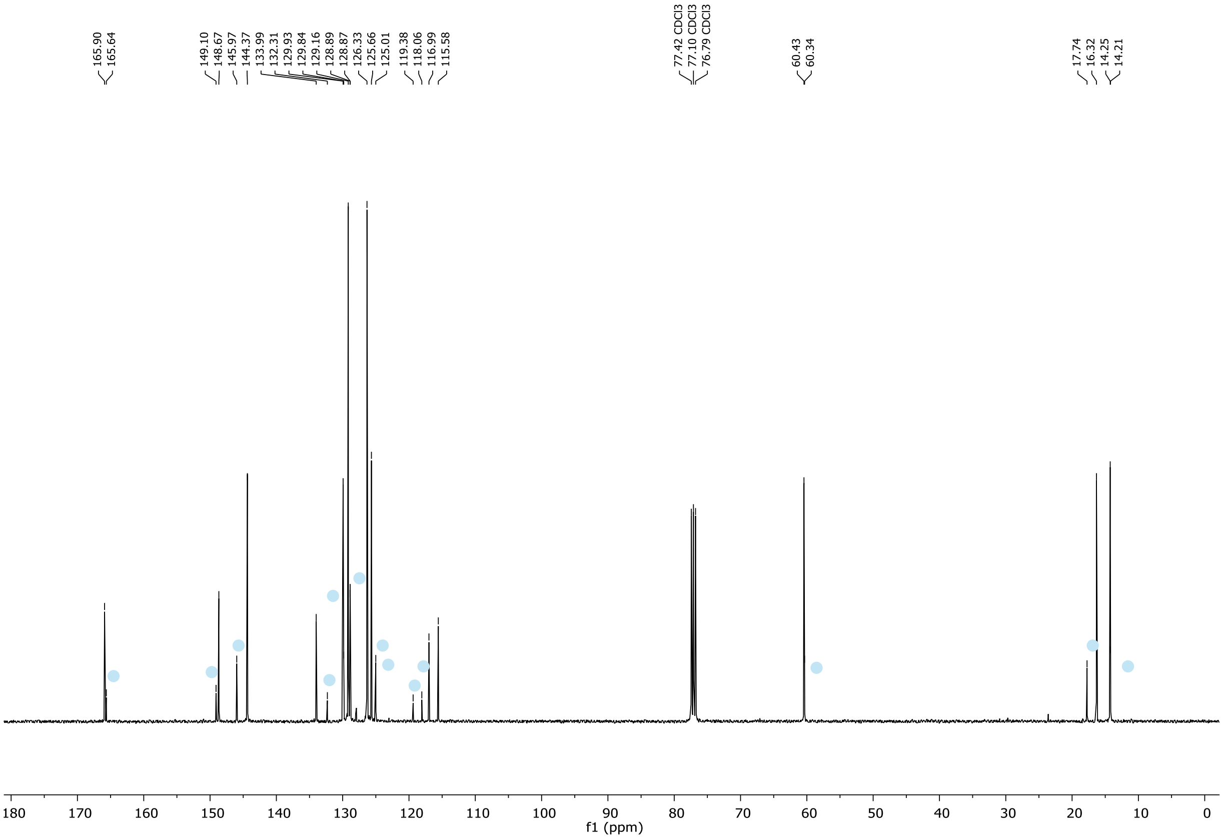

Ethyl (2*E*,4*Z*)-5-cyano-5-(4-methoxyphenyl)-3-methylpenta-2,4-dienoate (1o)

^{1}H NMR (*dr*>20:1)

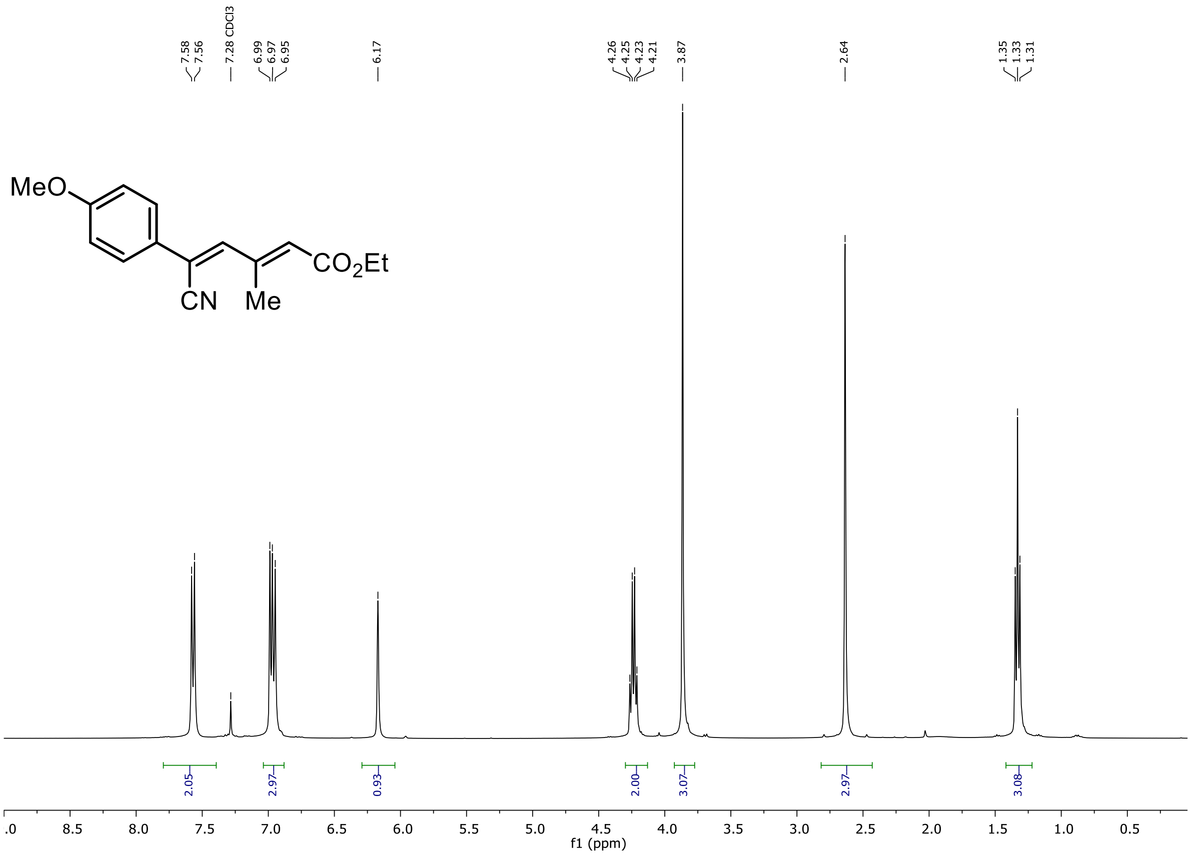

^{13}C NMR

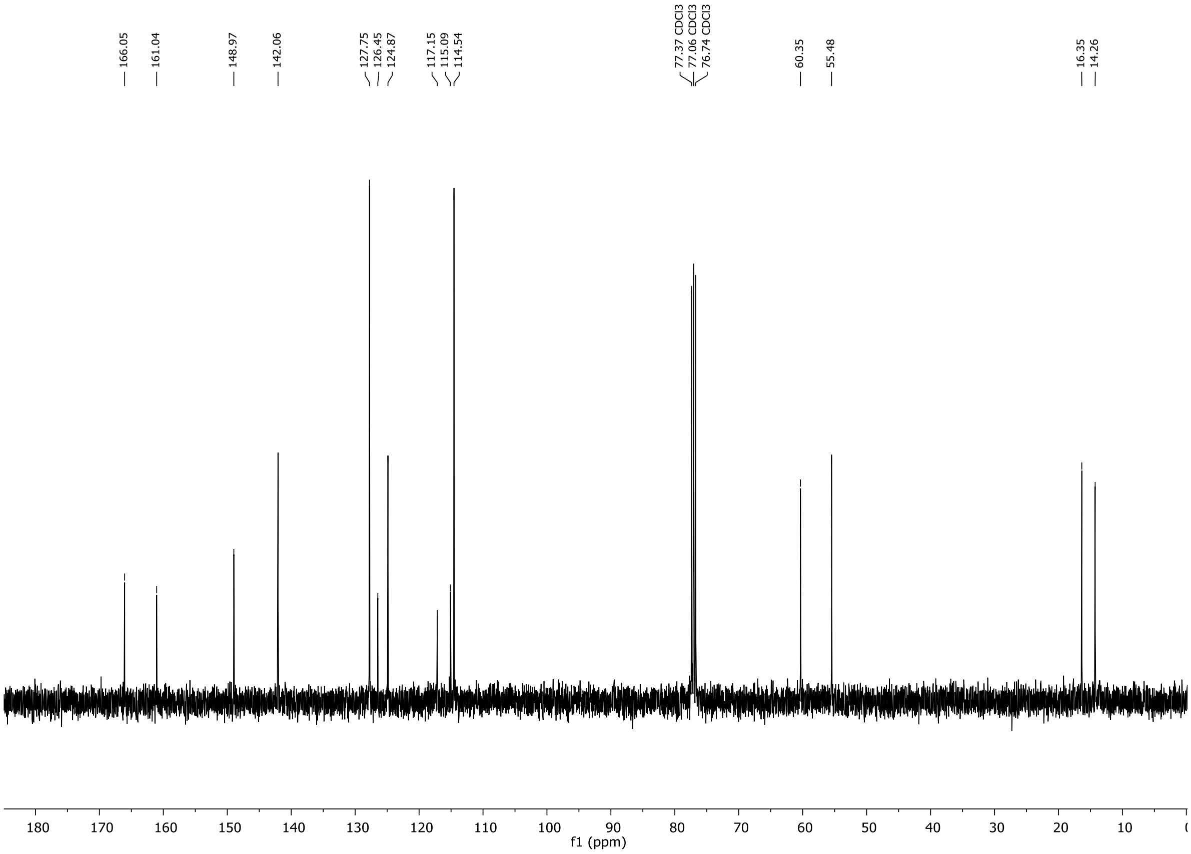

Ethyl (2*E*,4*Z*)-5-cyano-3-methyl-5-(m-tolyl)penta-2,4-dienoate (1p)

^{1}H NMR (*dr*>20:1)

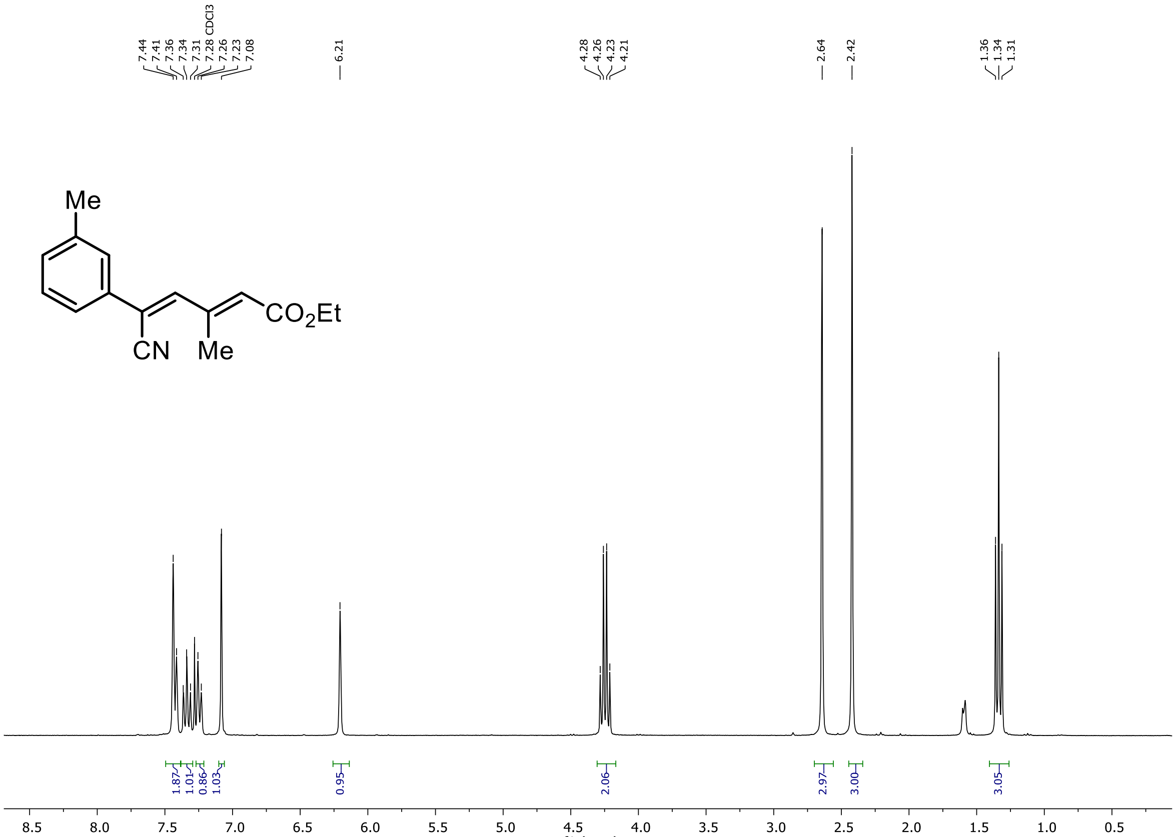

^{13}C NMR

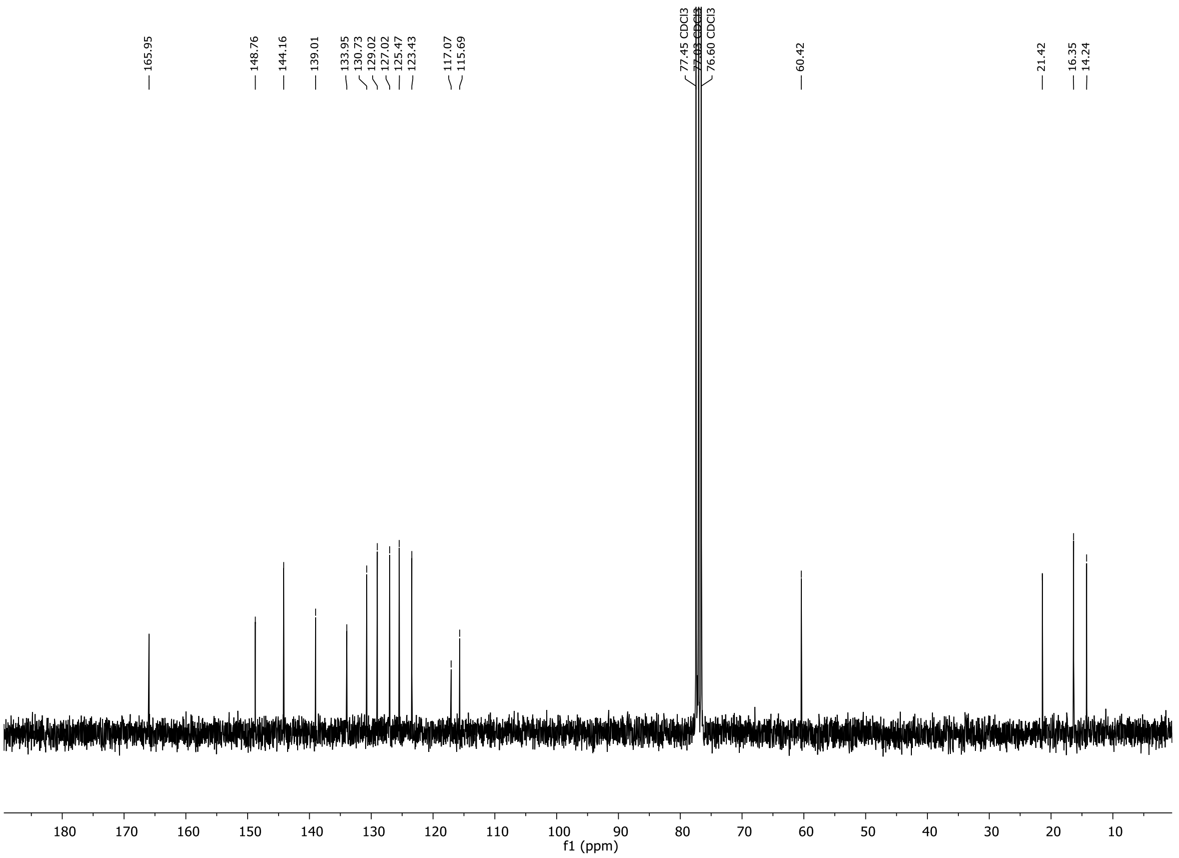

Ethyl (2*E*,4*Z*)-5-cyano-5-fluoro-3-methylpenta-2,4-dienoate (1q)

^{1}H NMR (*dr*=86:14)

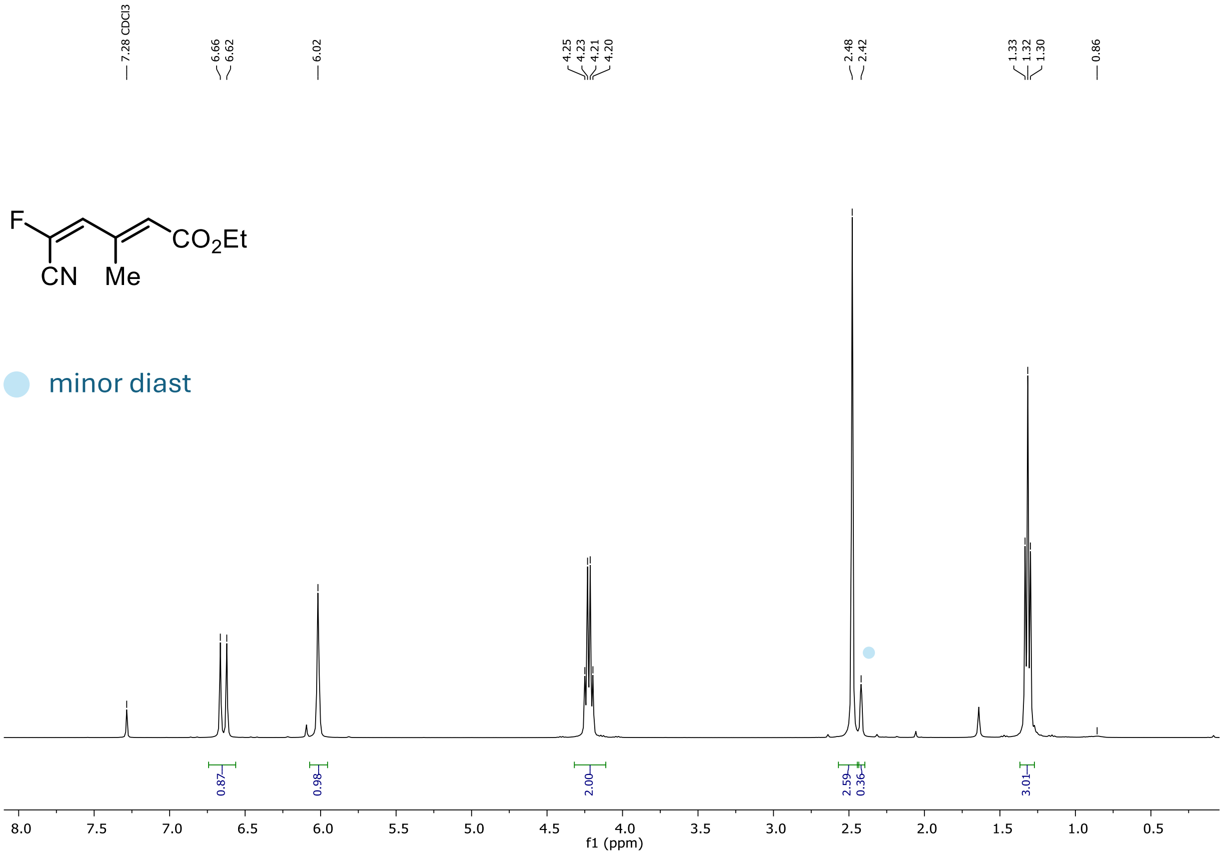

^{13}C NMR

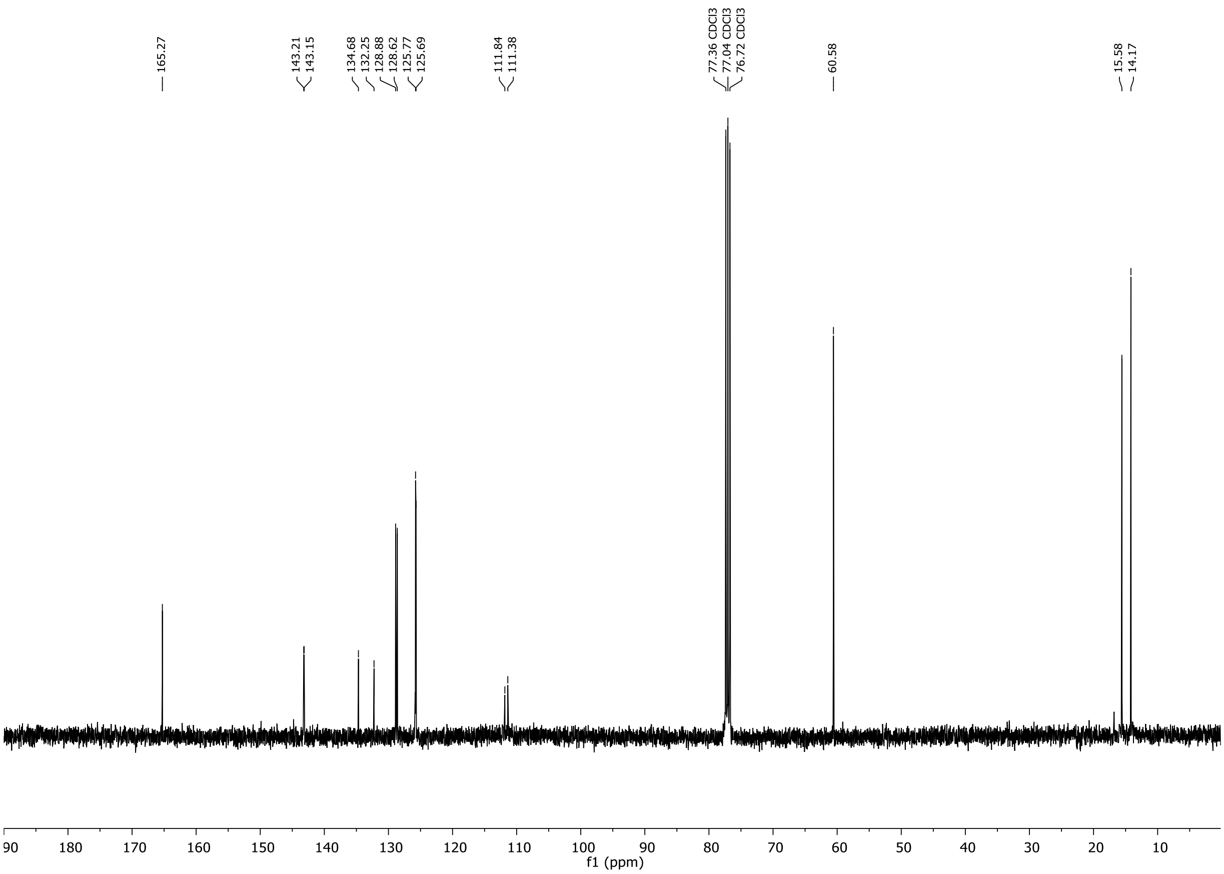

^{19}F NMR

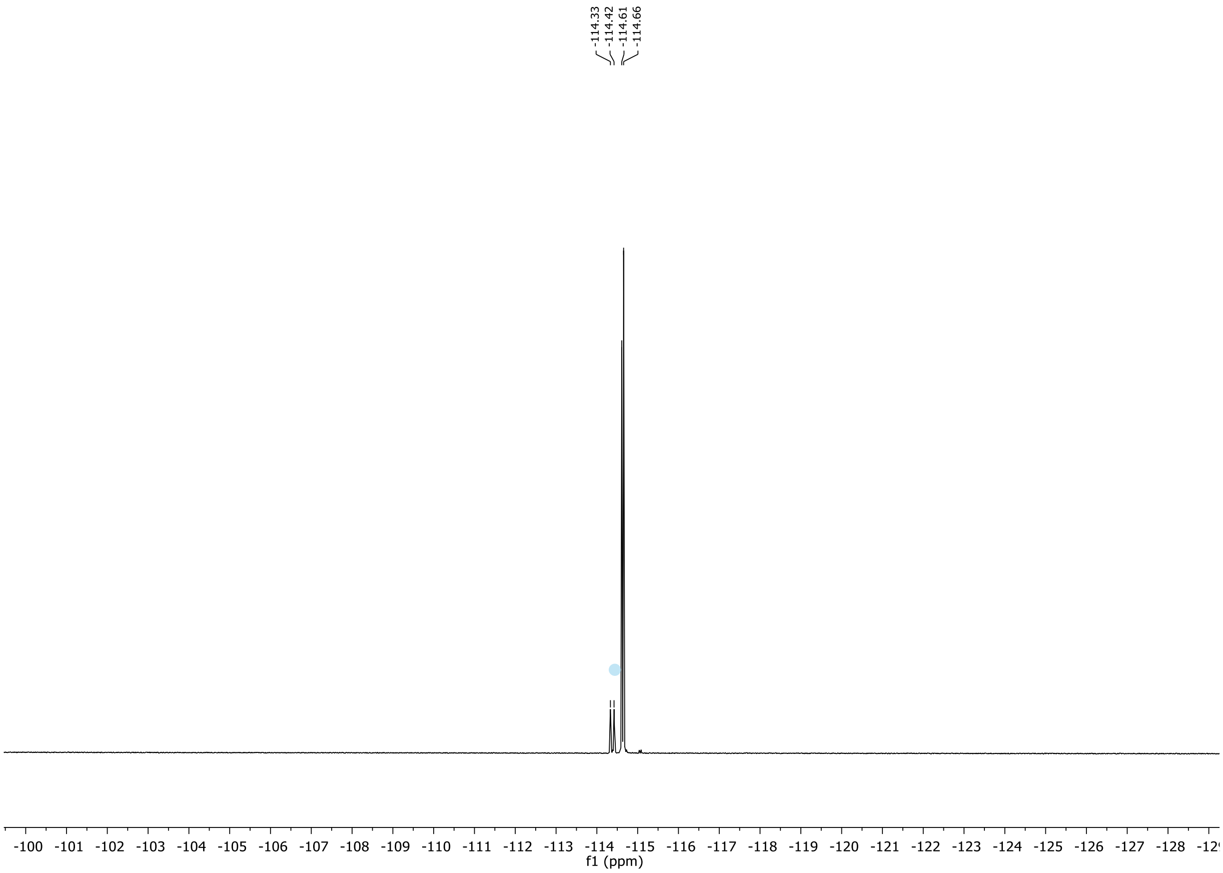

Ethyl (2*E*,4*E*)-5-cyano-3-methyl-5-(methylthio)penta-2,4-dienoate (1r)

^{1}H NMR (*dr*>20:1)

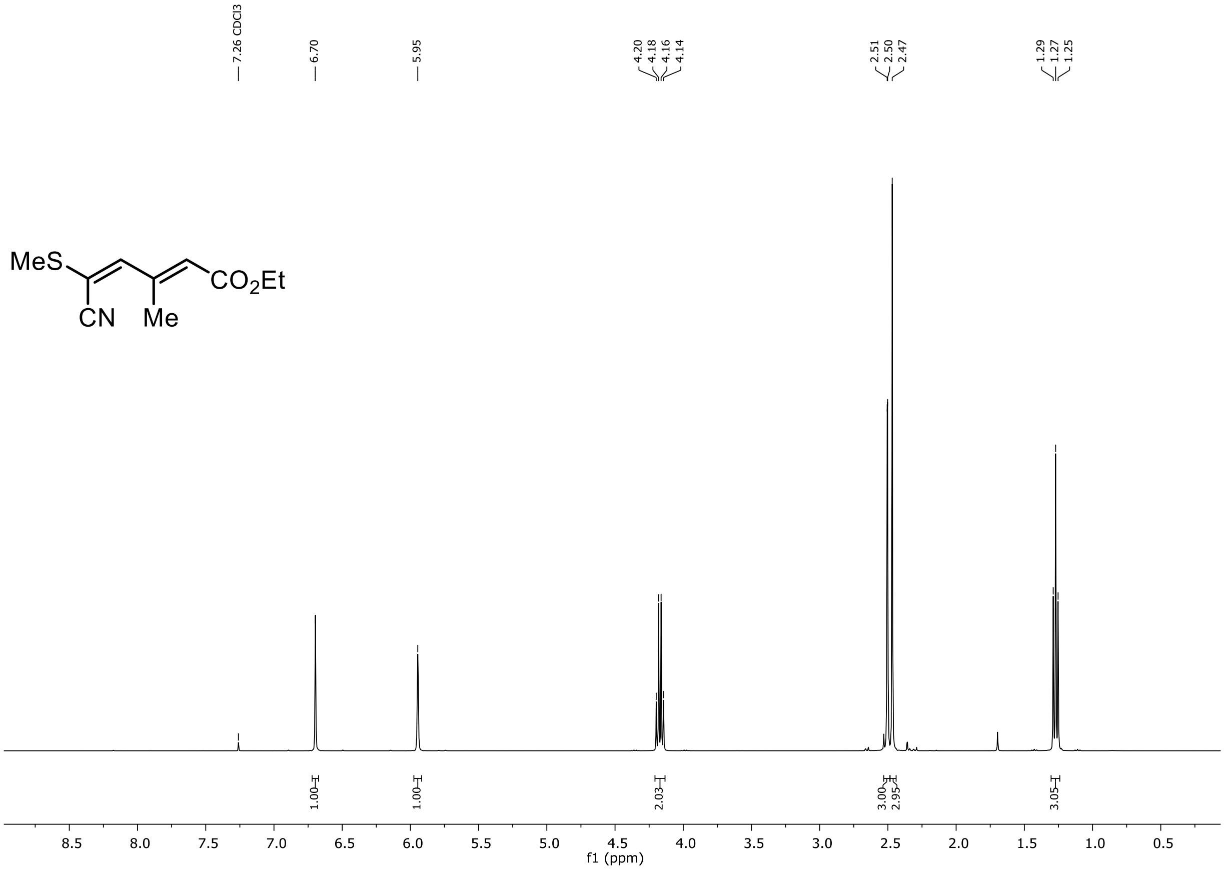

^{13}C NMR

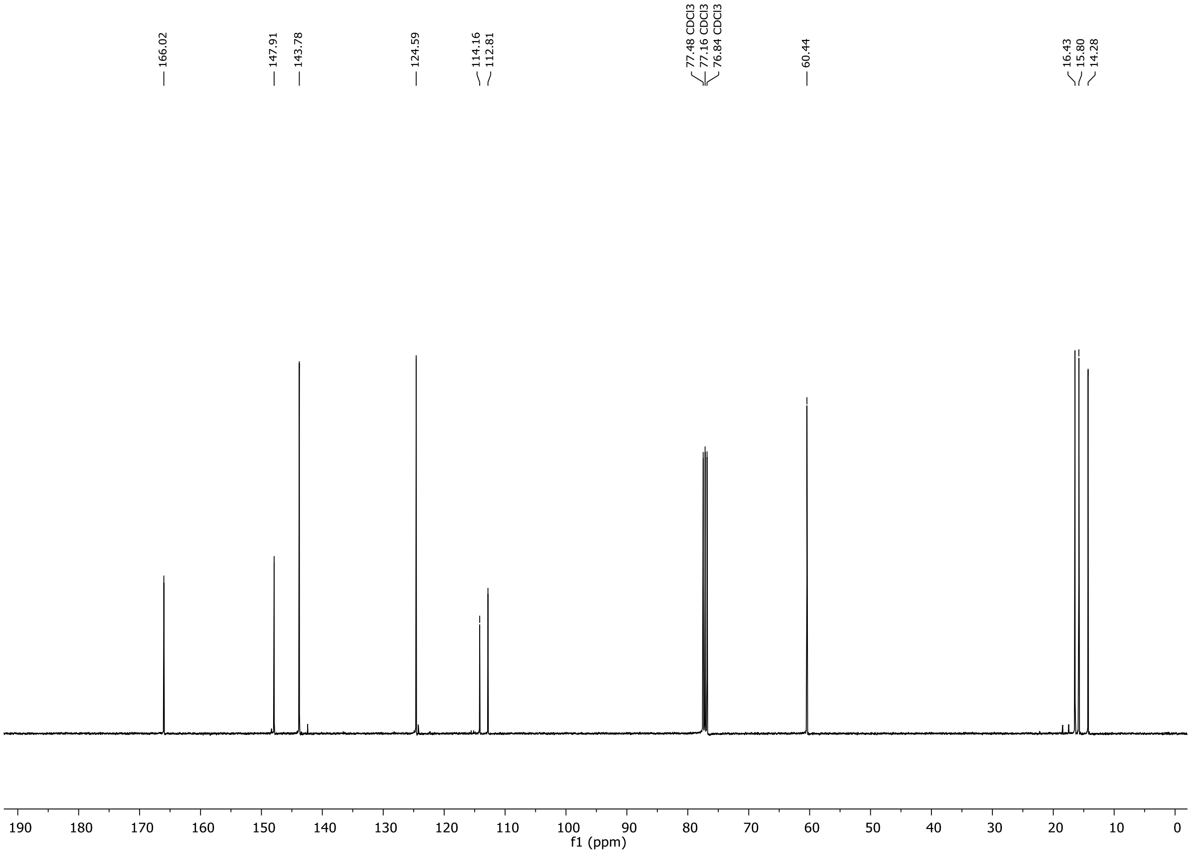

Methyl (2*E*,4*Z*)-5-cyano-3-ethyl-6-phenylhexa-2,4-dienoate (1s)

^{1}H NMR (*dr*>20:1)

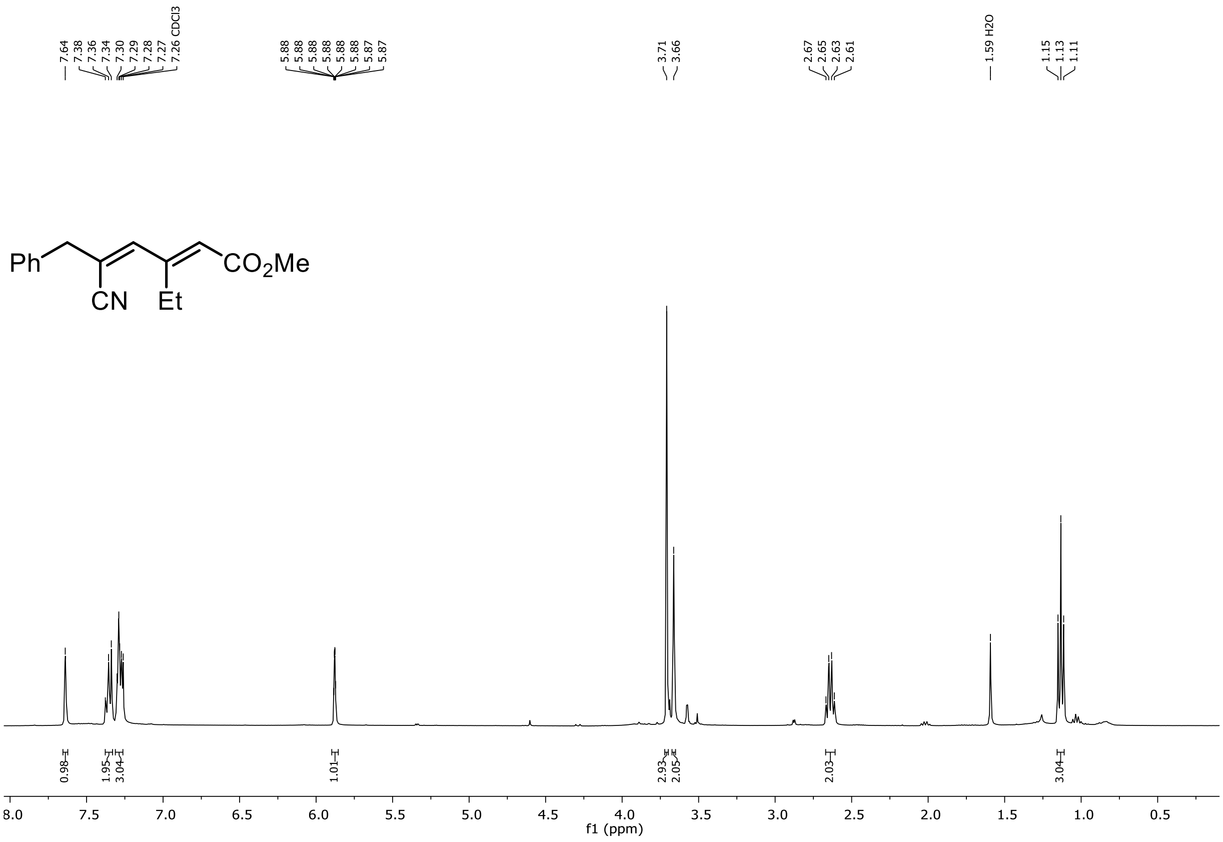

^{13}C NMR

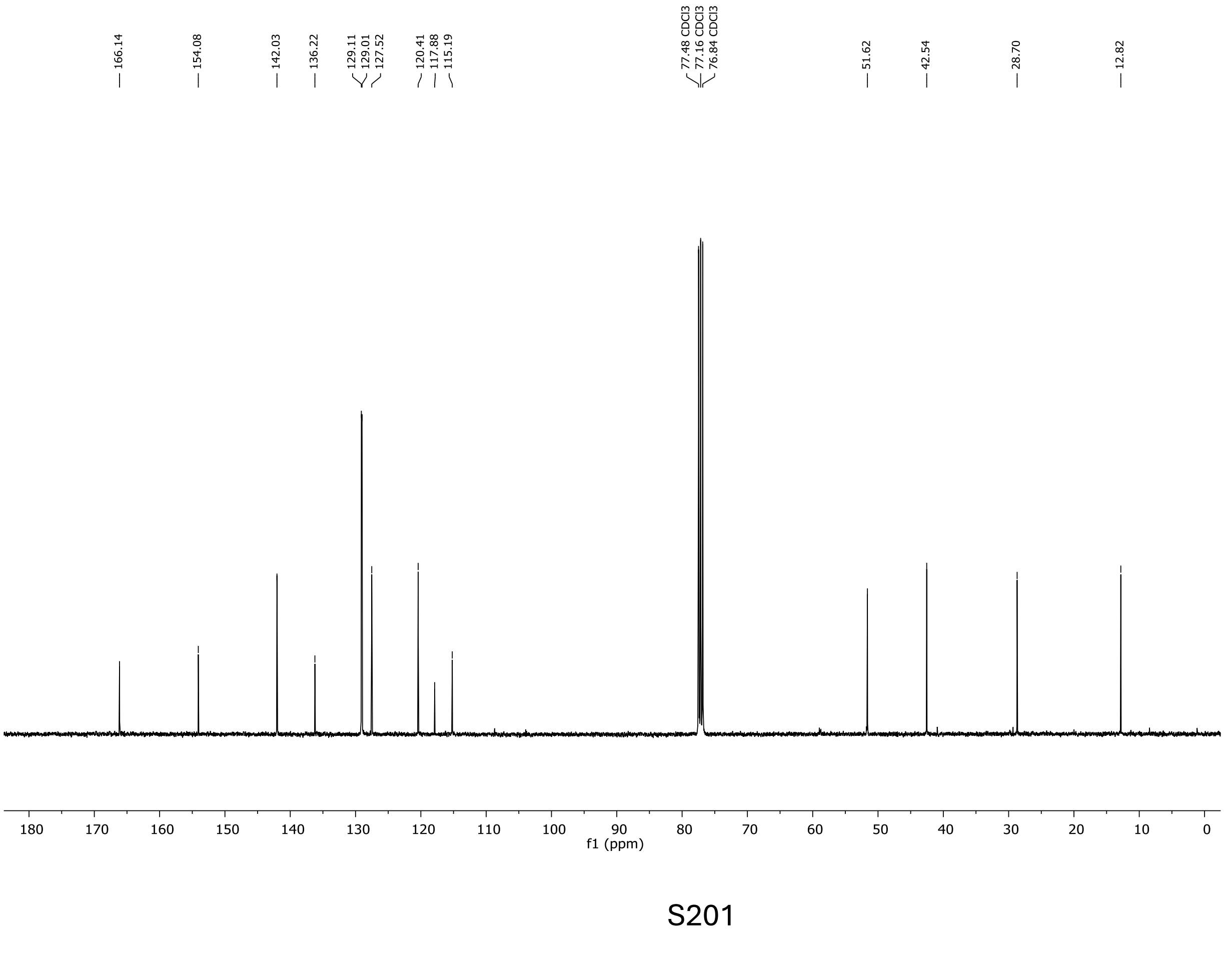

Methyl (2*E*,4*Z*)-5-cyano-3-isopropyl-6-phenylhexa-2,4-dienoate (1t)

^{1}H NMR (*dr*>20:1)

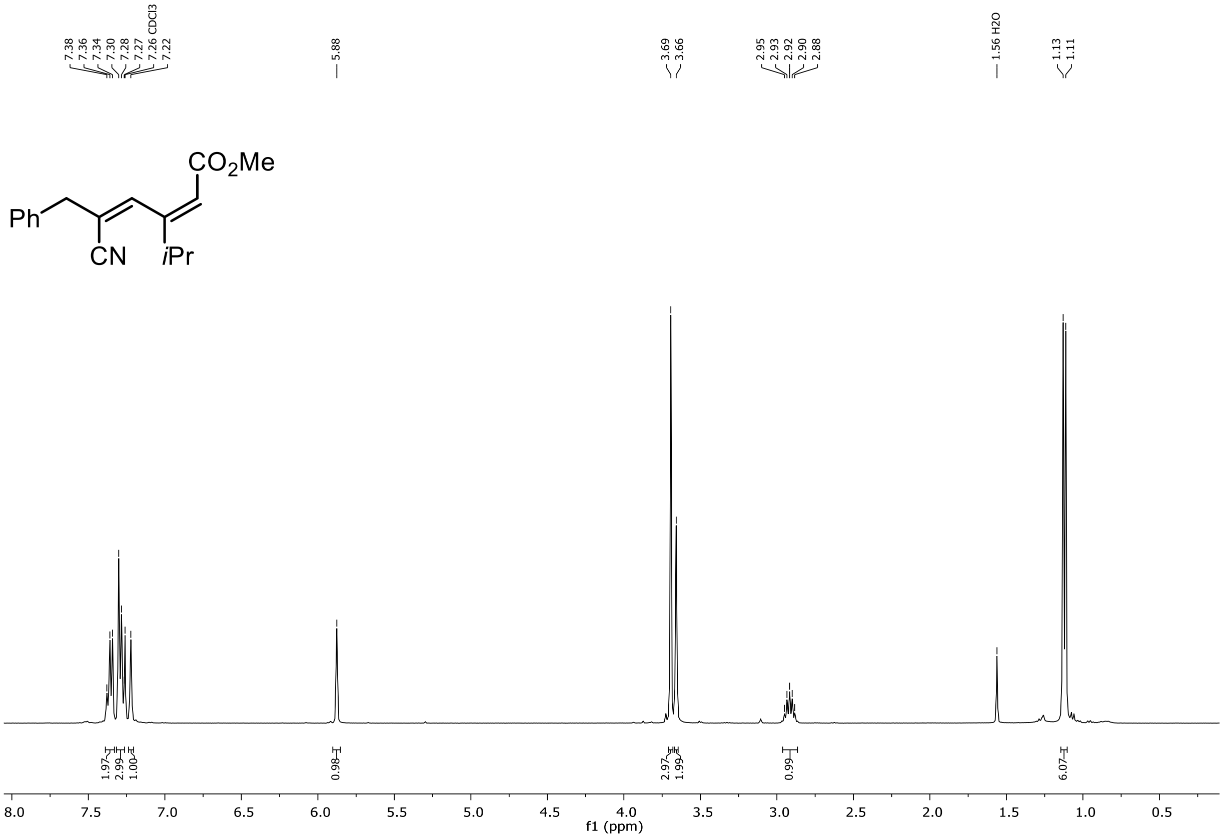

^{13}C NMR

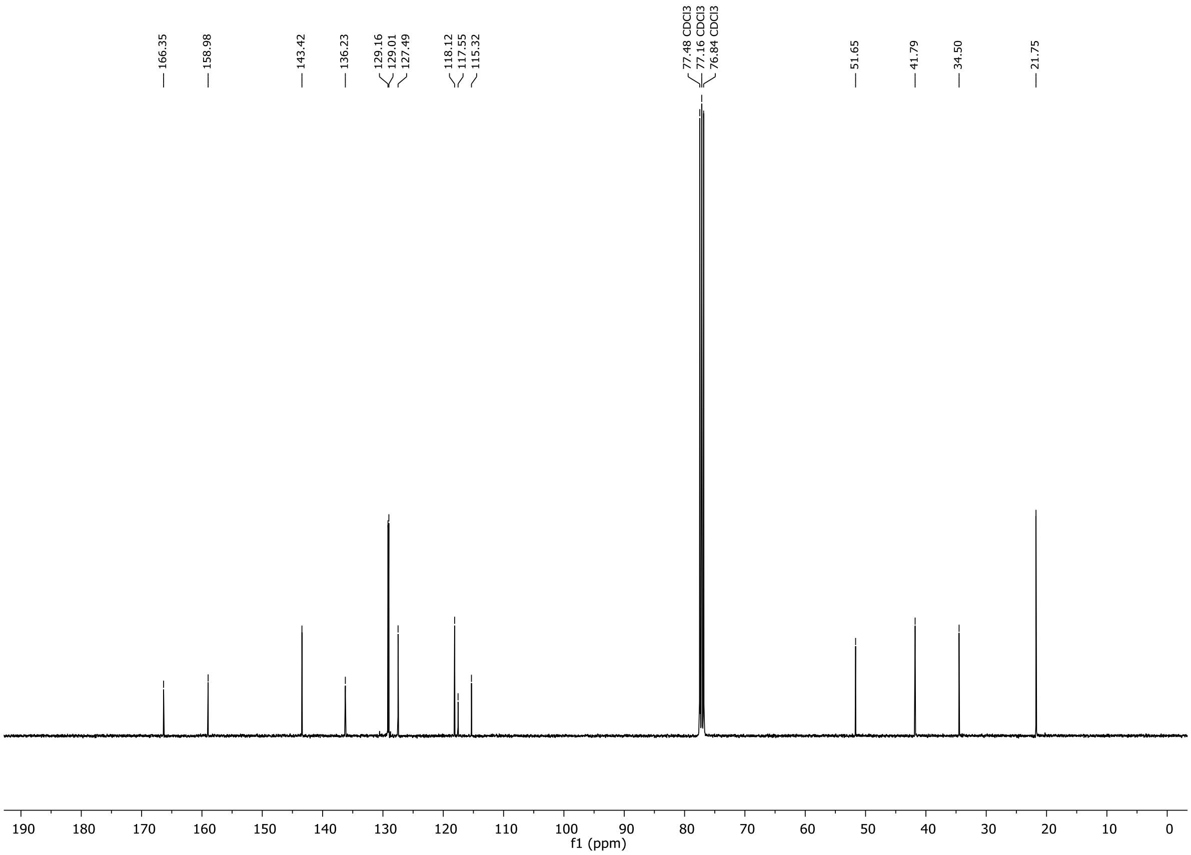

(*Z*)-2-benzyl-3-(5-oxo-2,5-dihydrofuran-3-yl)acrylonitrile (1u)

^{1}H NMR (*dr*>20:1)

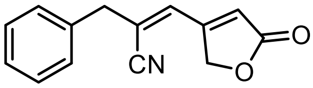

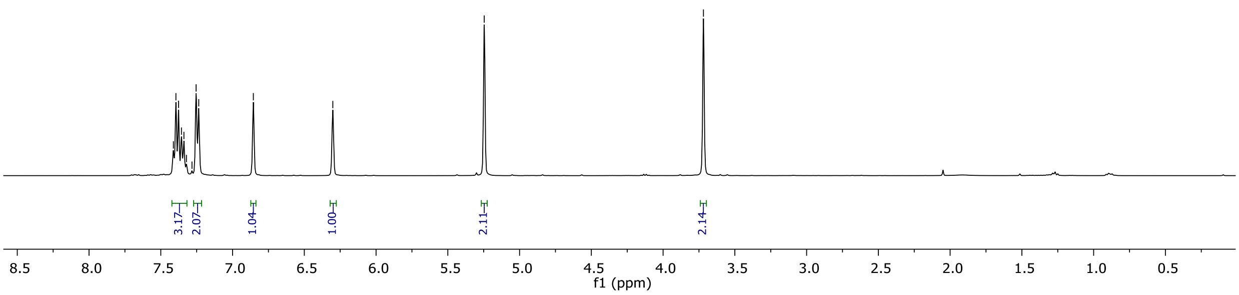

^{13}C NMR

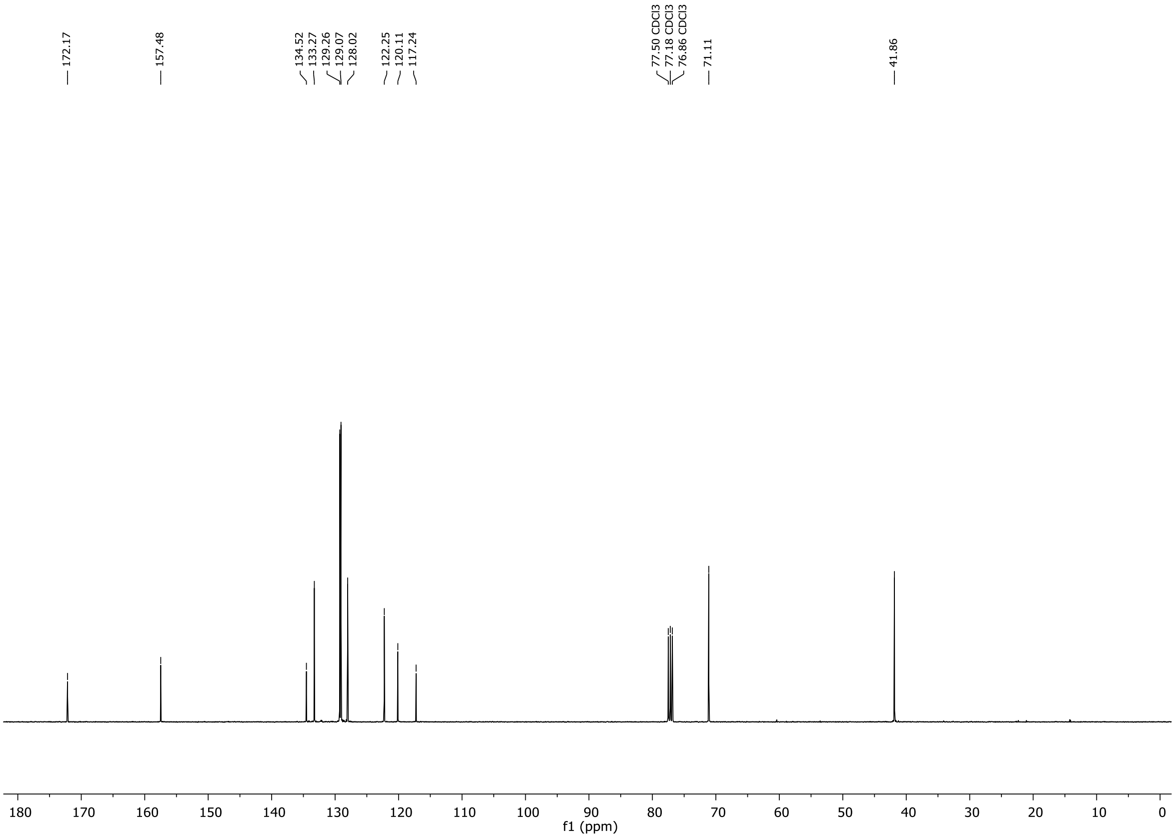

Ethyl (*E*)-3-((*Z*)-2-cyanoprop-1-en-1-yl)hepta-2,6-dienoate (1v)

^{1}H NMR (*dr*>20:1)

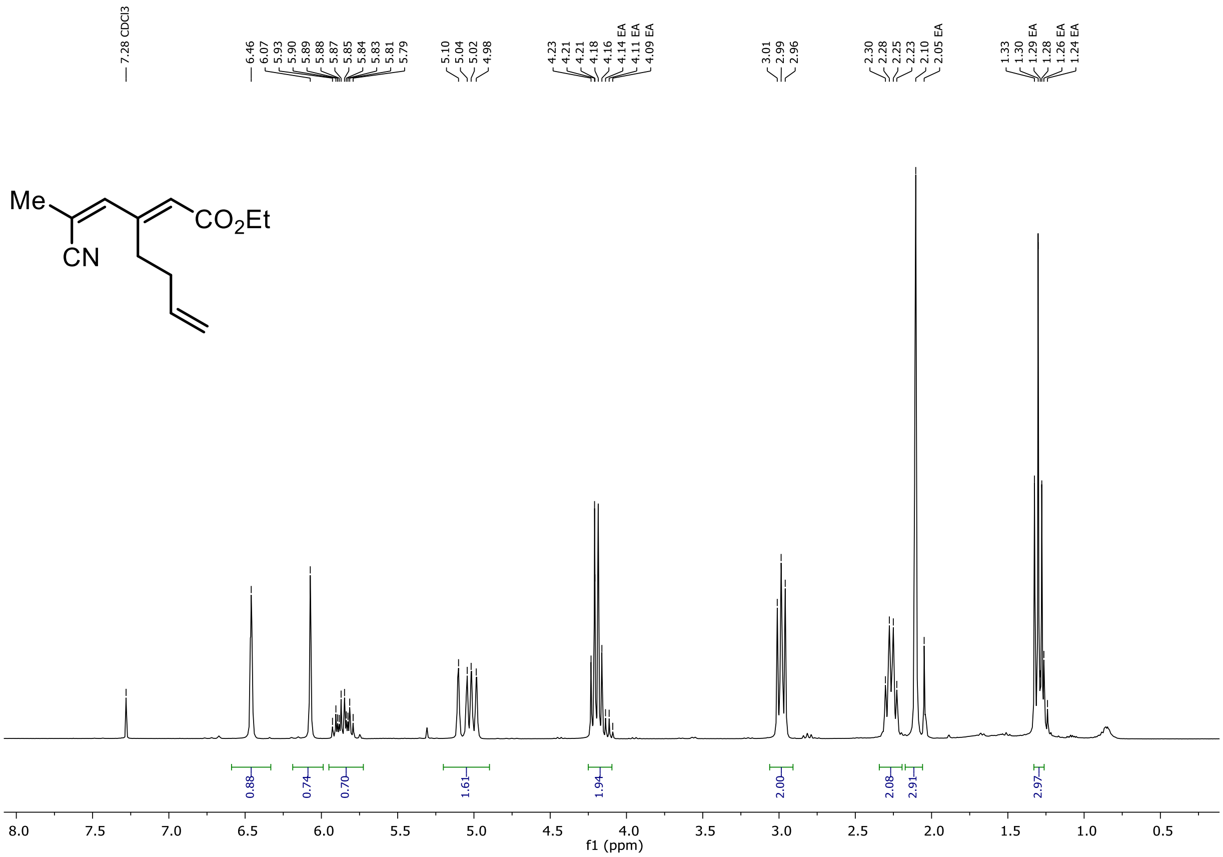

^{13}C NMR

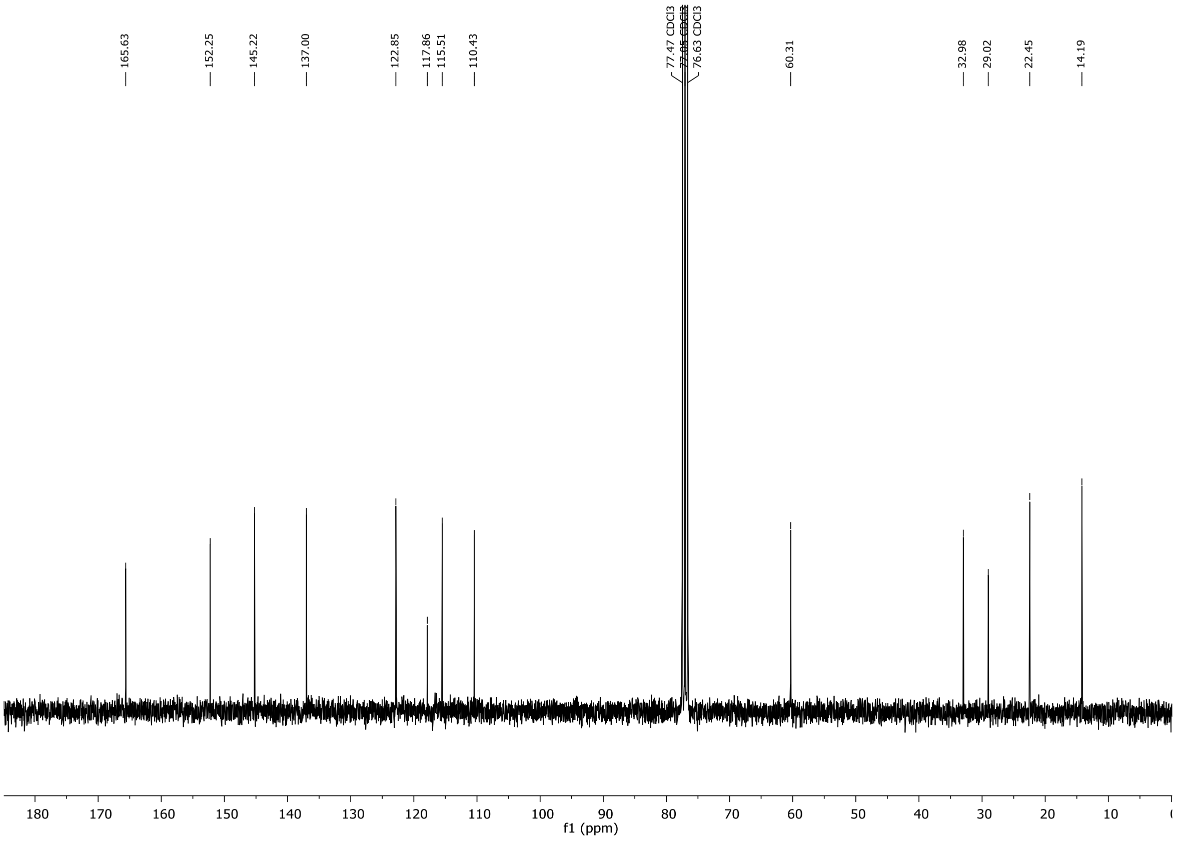

Ethyl (2*E*,4*Z*)-3-(2-(1,3-dioxolan-2-yl)ethyl)-5-cyano-6-phenylhexa-2,4-dienoate (1w)

^{1}H NMR (*dr*>20:1)

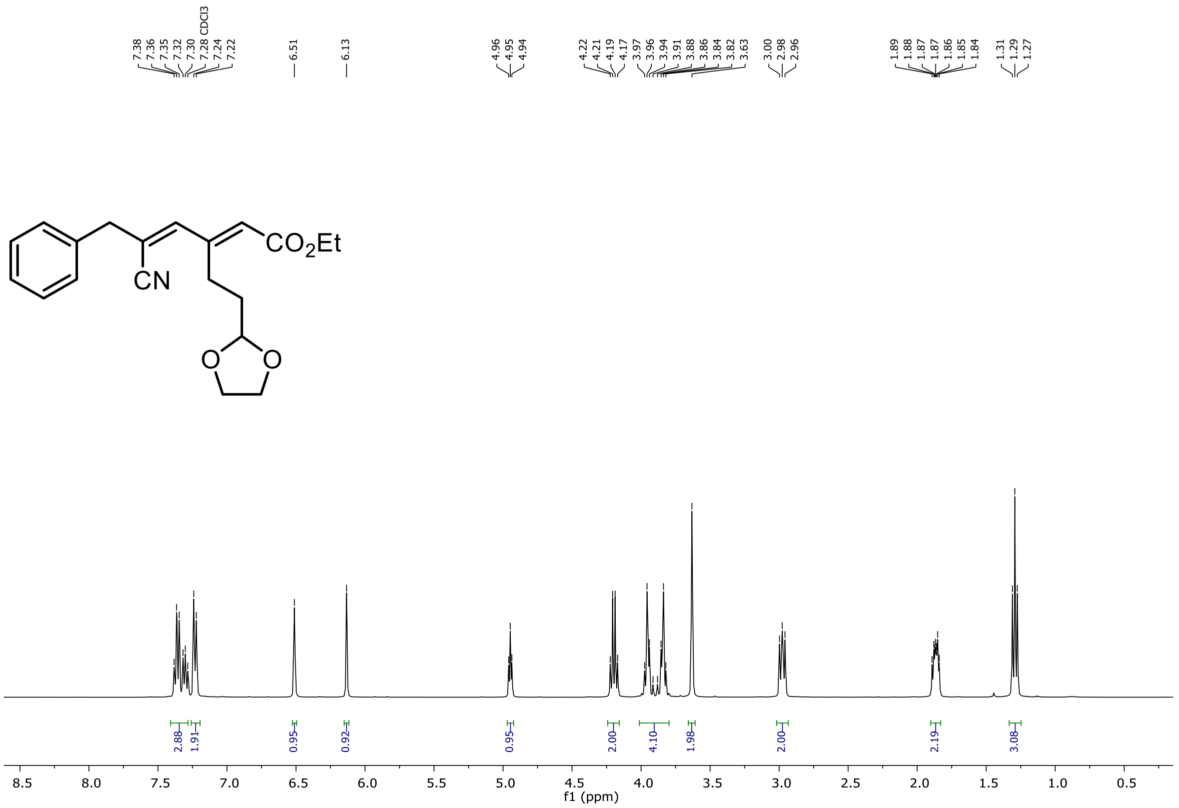

^{13}C NMR

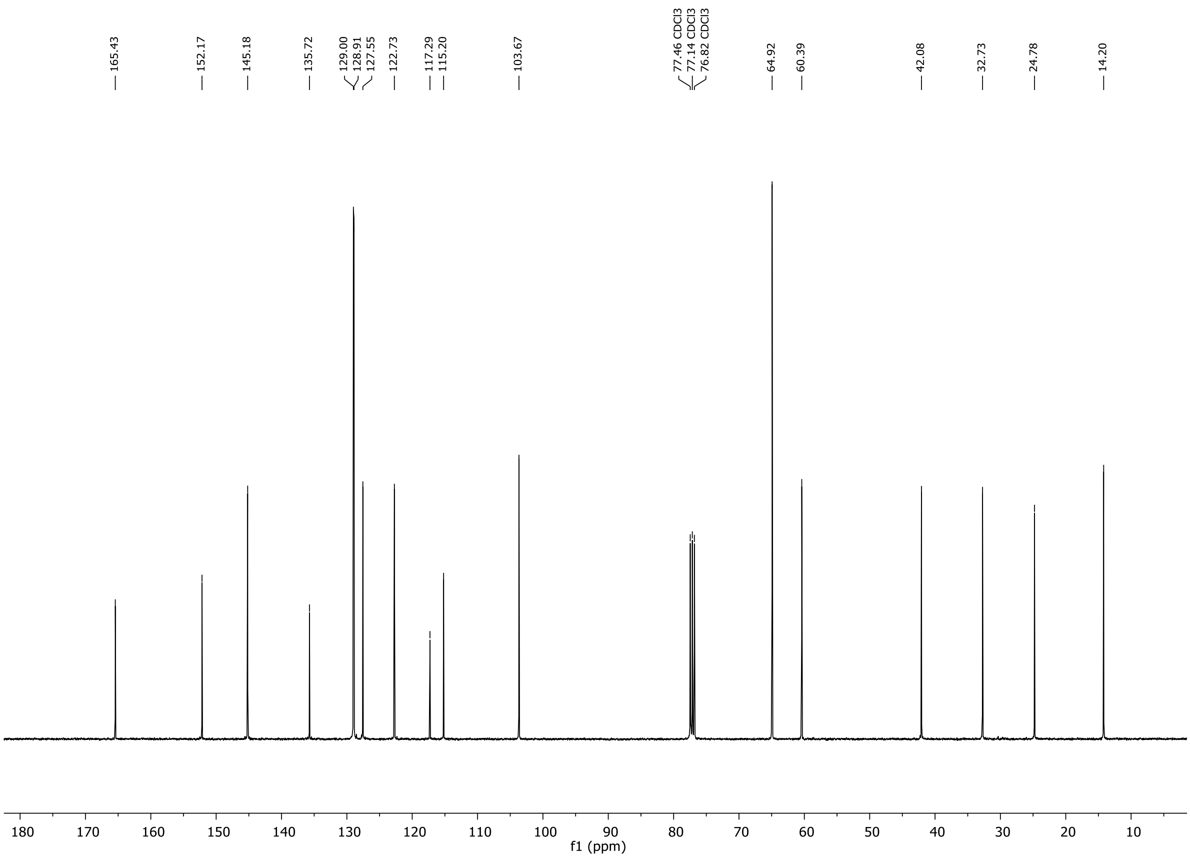

Ethyl (2*Z*,4*Z*)-5-cyano-3,6-diphenylhexa-2,4-dienoate (1x)

^{1}H NMR (*dr*>20:1)

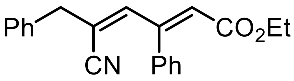

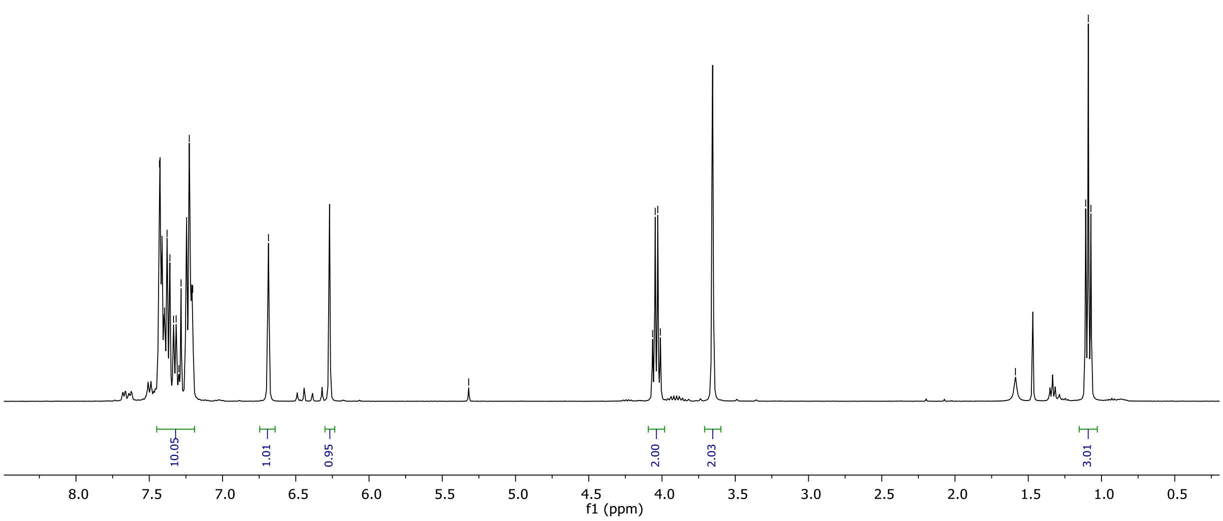

^{13}C NMR

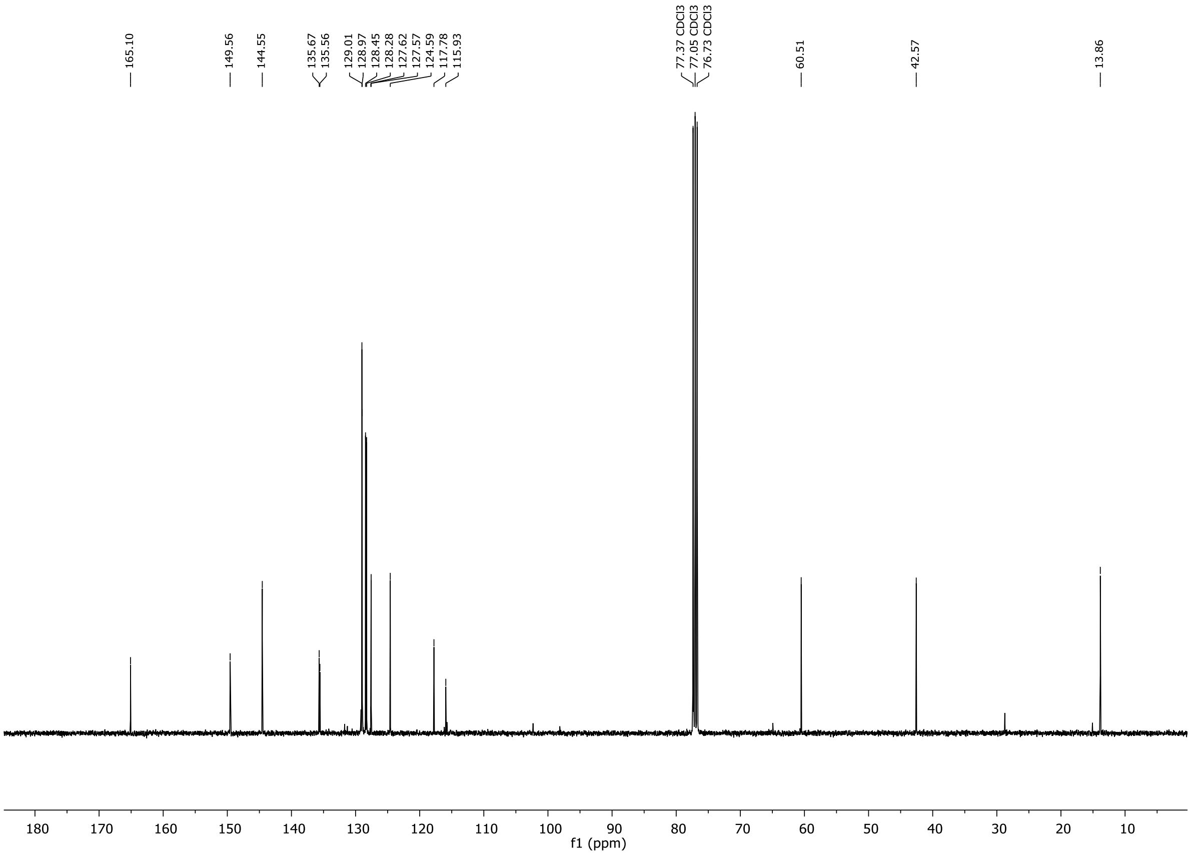

Ethyl (2*Z*,4*Z*)-5-cyano-3-(naphthalen-2-yl)-6-phenylhexa-2,4-dienoate (1y)

^{1}H NMR (*dr*>20:1)

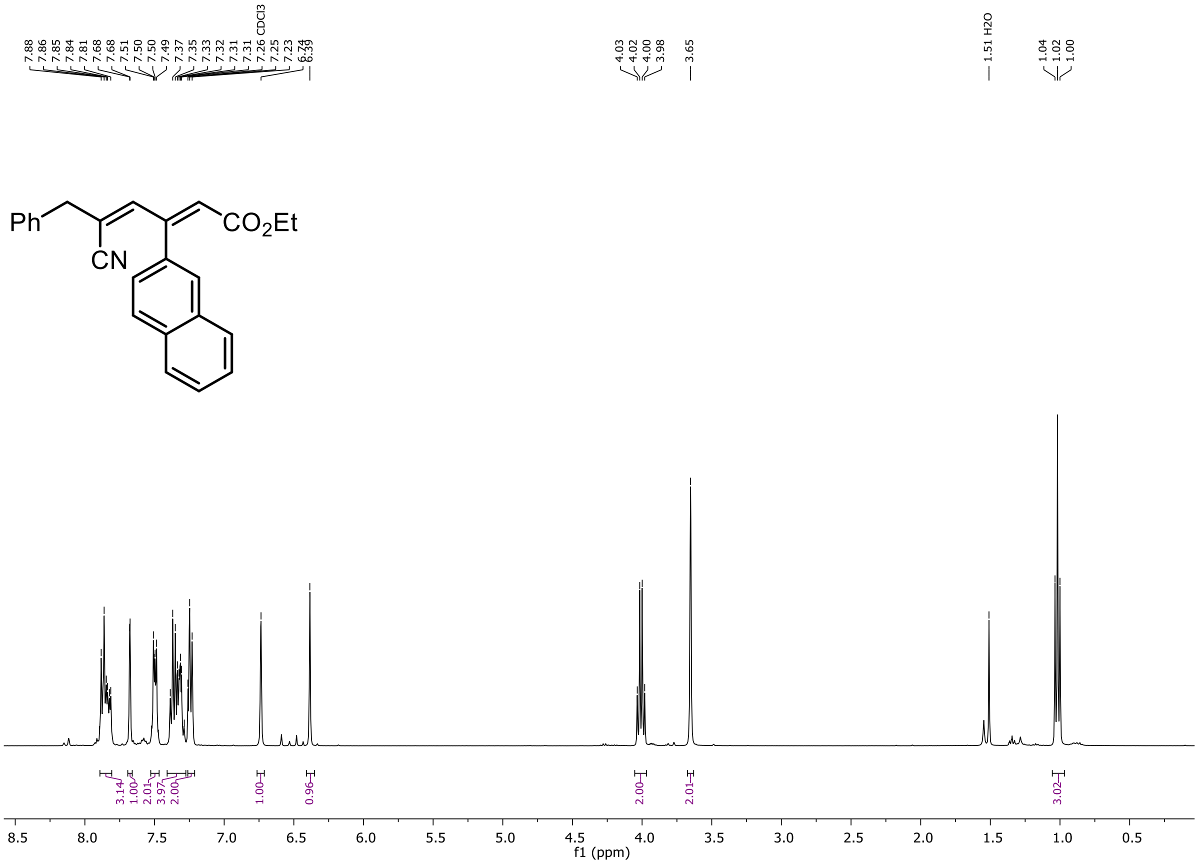

^{13}C NMR

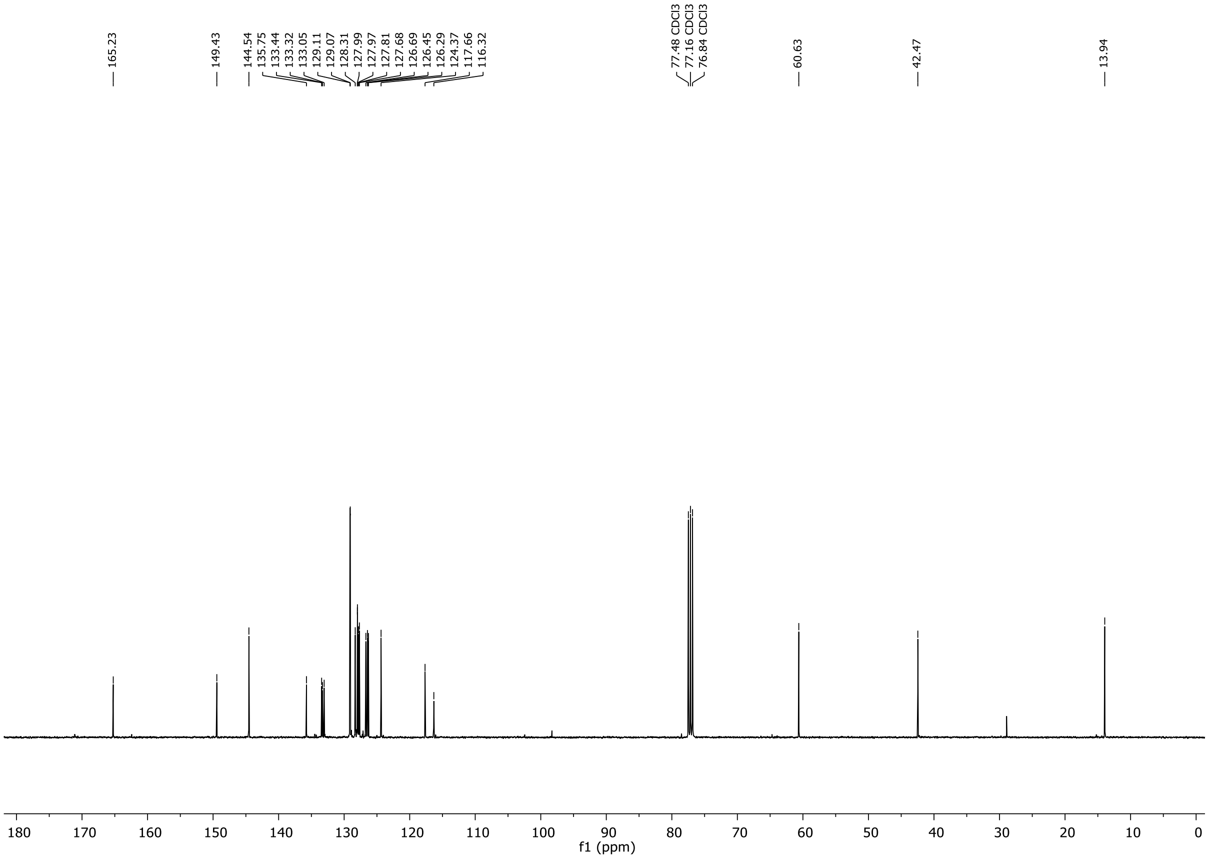

Ethyl (2*Z*,4*Z*)-5-cyano-6-phenyl-3-(pyridin-4-yl)hexa-2,4-dienoate (1z)

^{1}H NMR (*dr*=80:20)

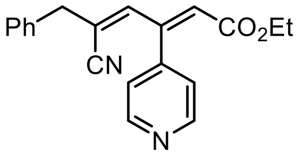

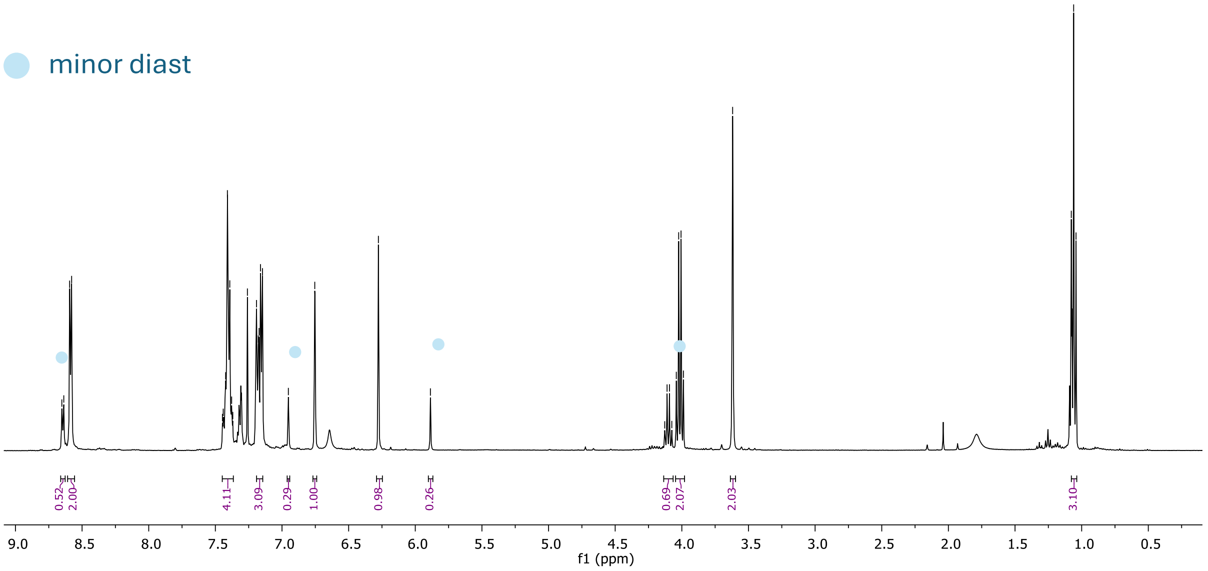

^{13}C NMR

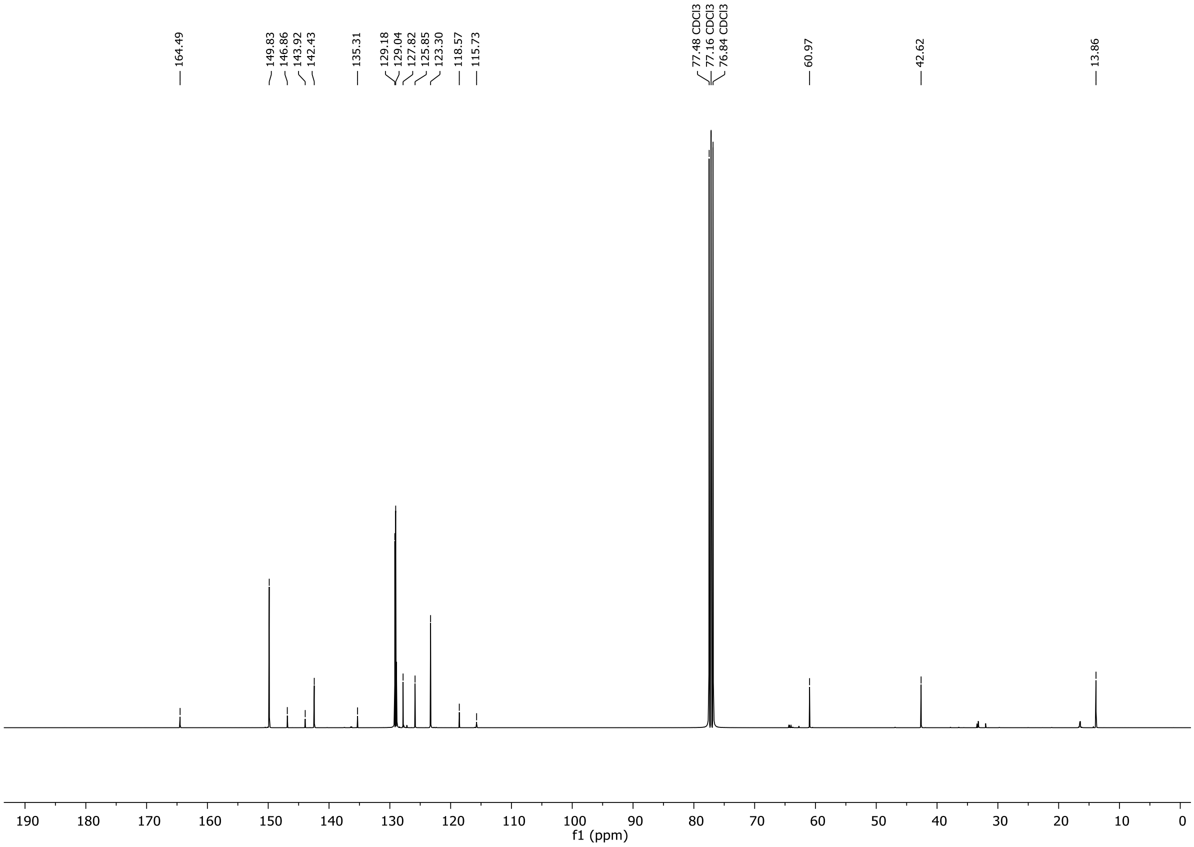

Ethyl (2E,4E)-5-cyano-5-fluoro-3,4-dimethylpenta-2,4-dienoate (1aa)

^{1}H NMR

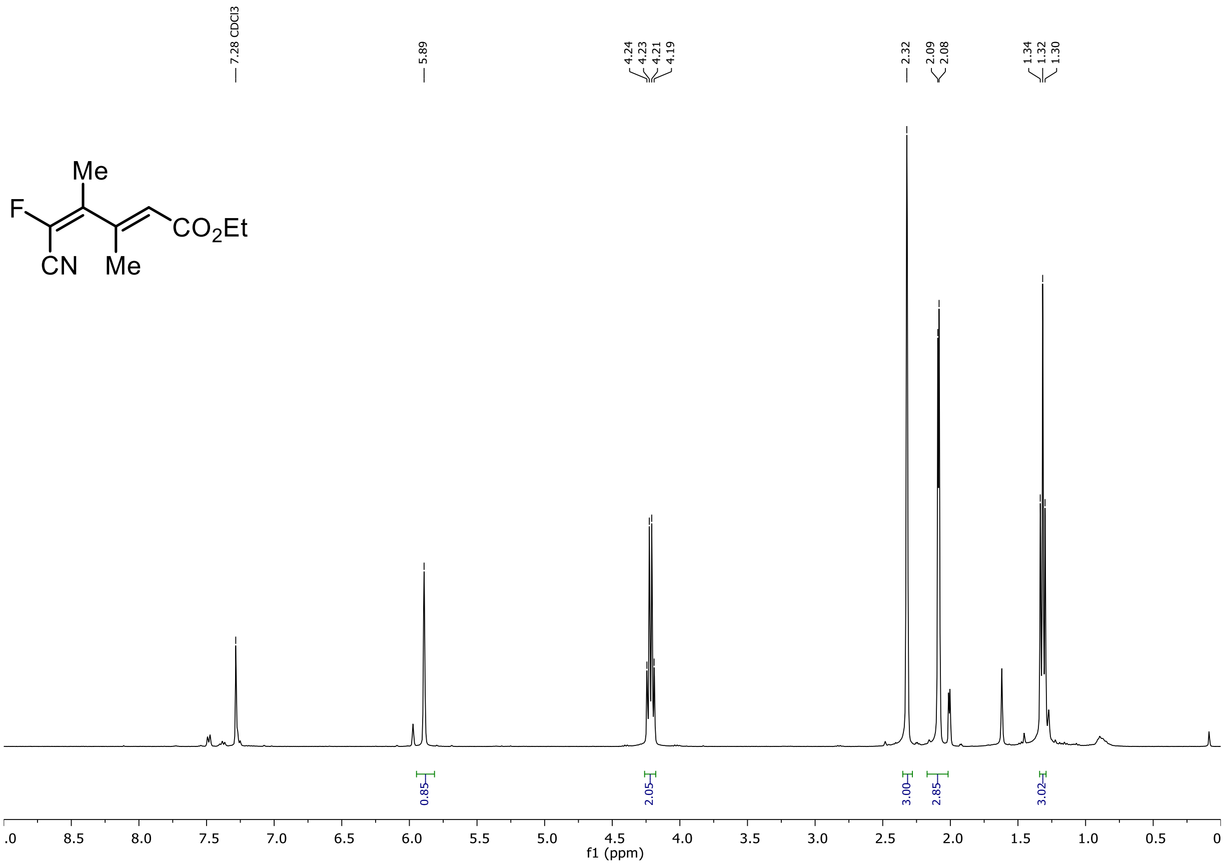

^{13}C NMR

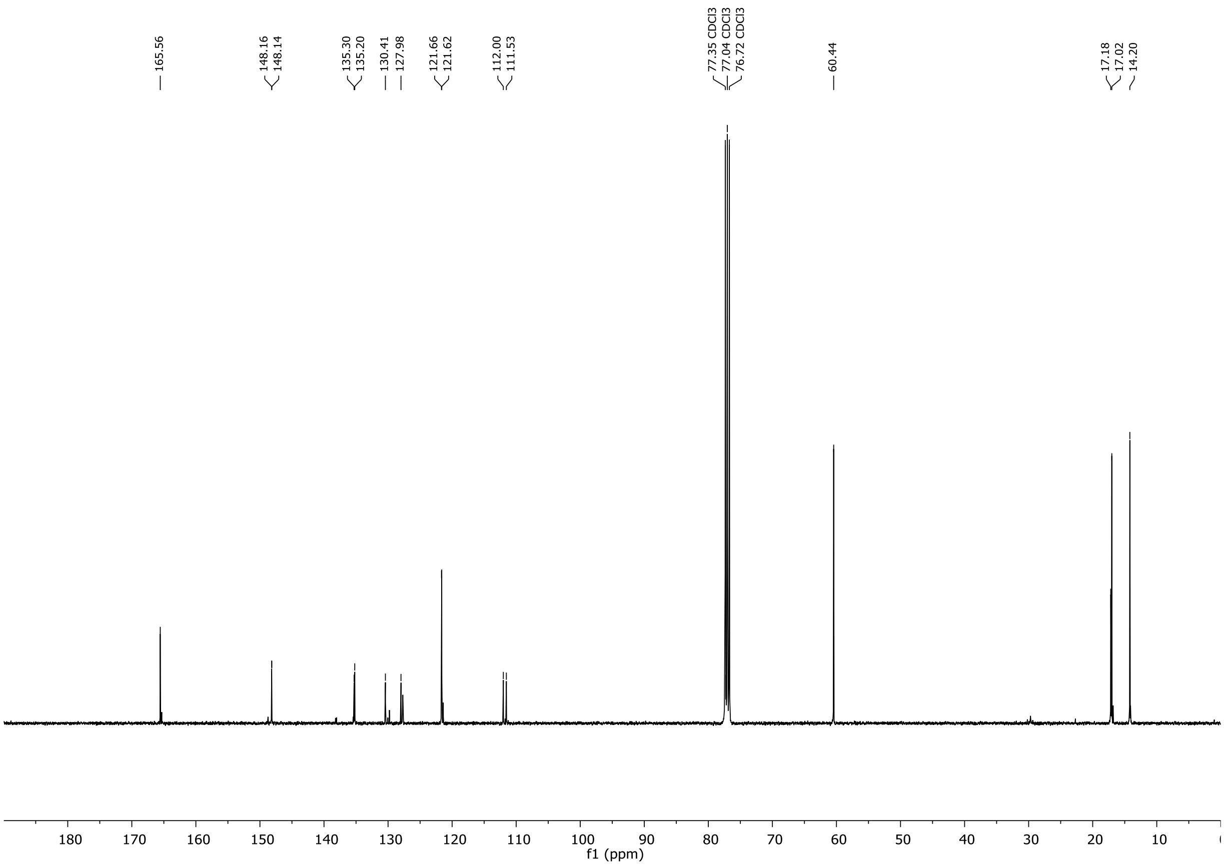

^{19}F-NMR

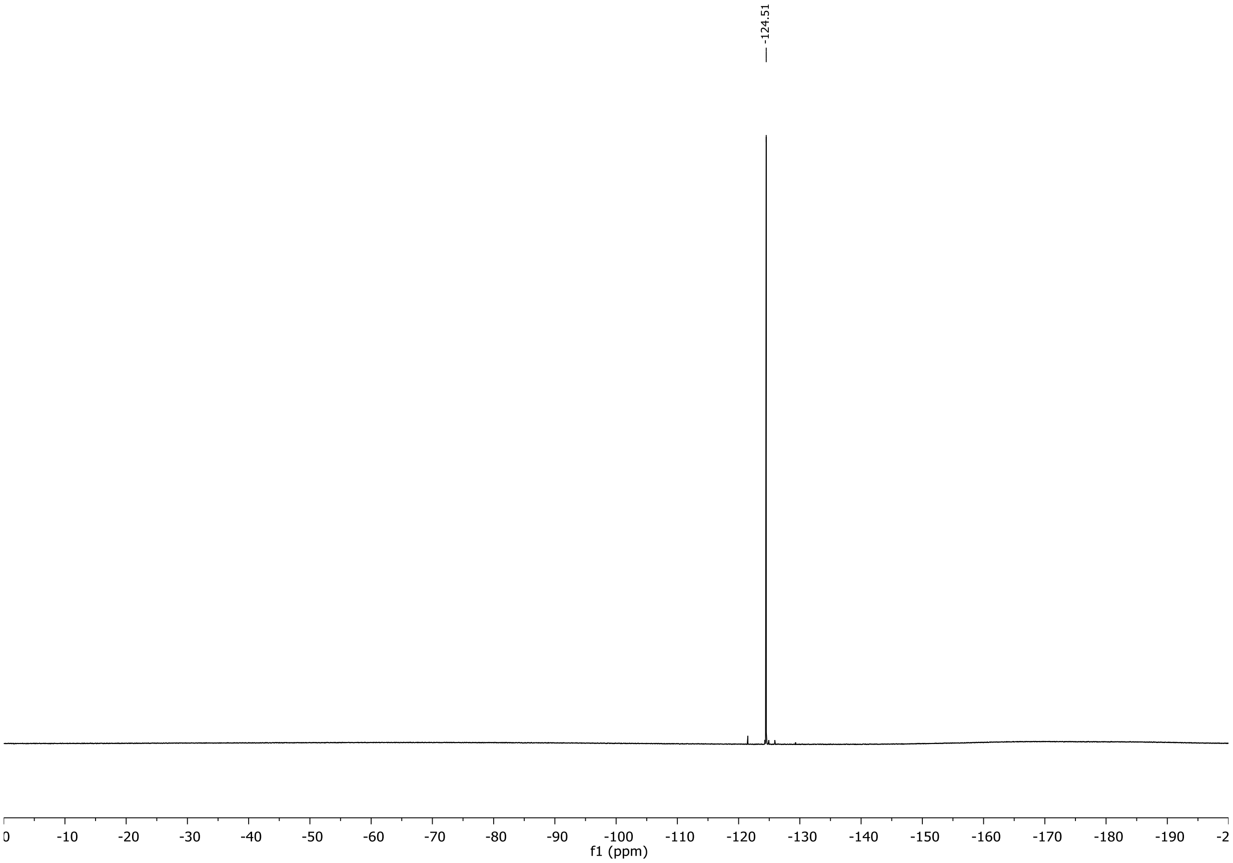

Ethyl (*R*)-2-amino-3-benzyl-5-methylcyclopenta-1,3-diene-1-carboxylate (3a)

^{1}H NMR

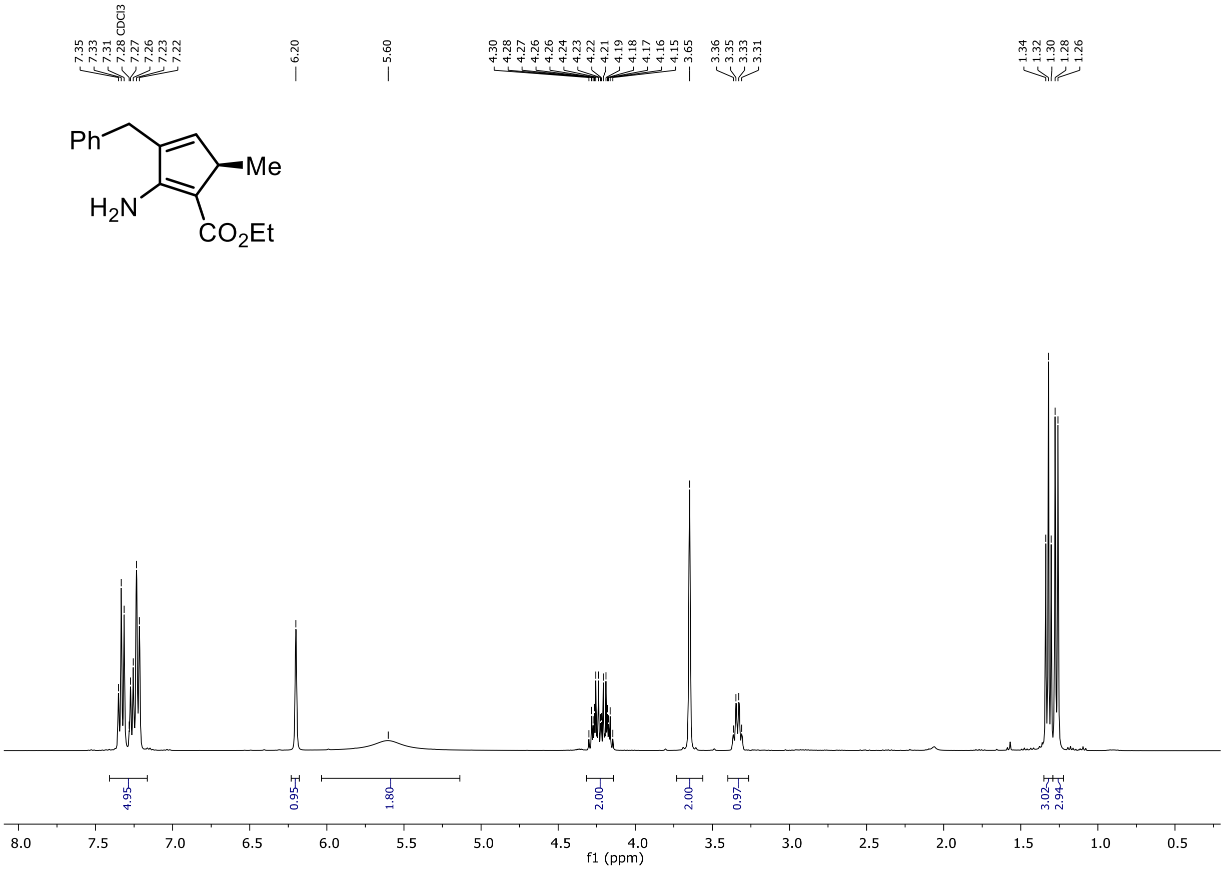

^{13}C NMR

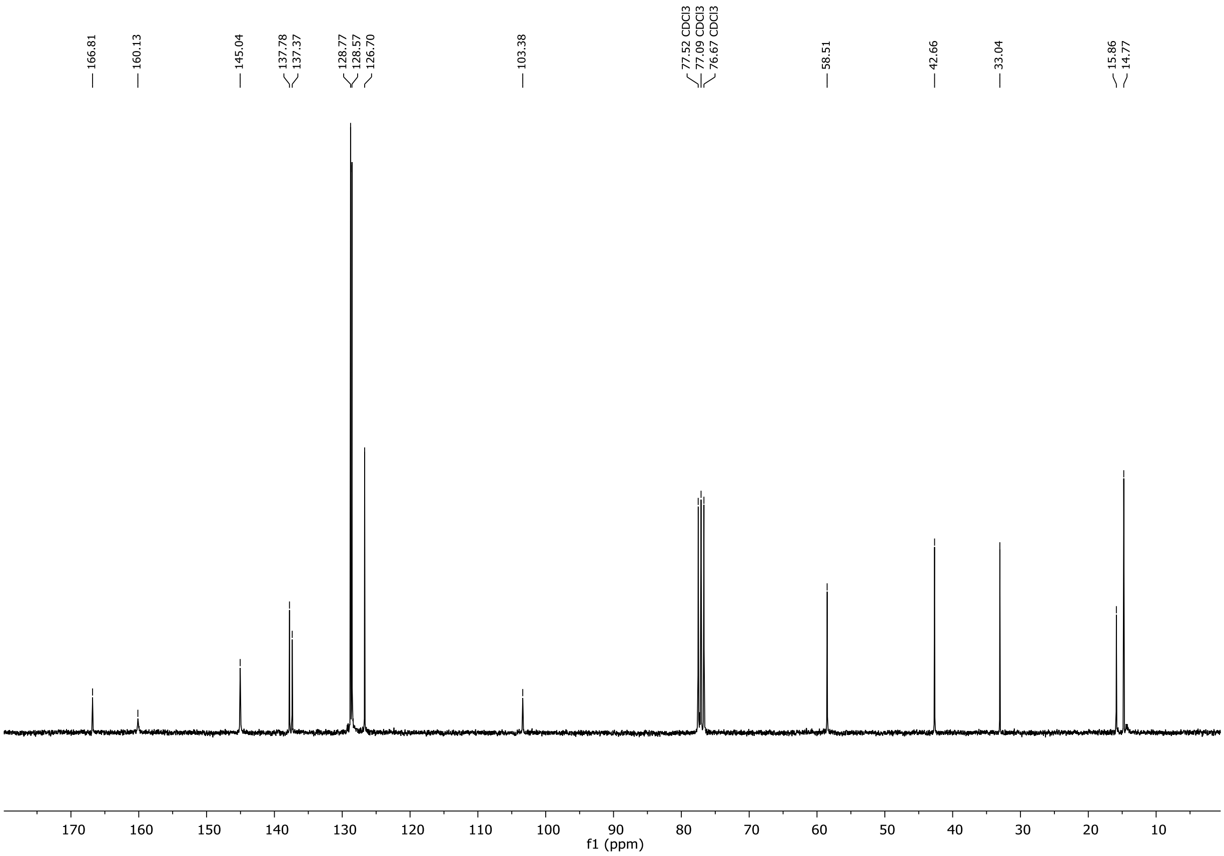

^{19}F NMR

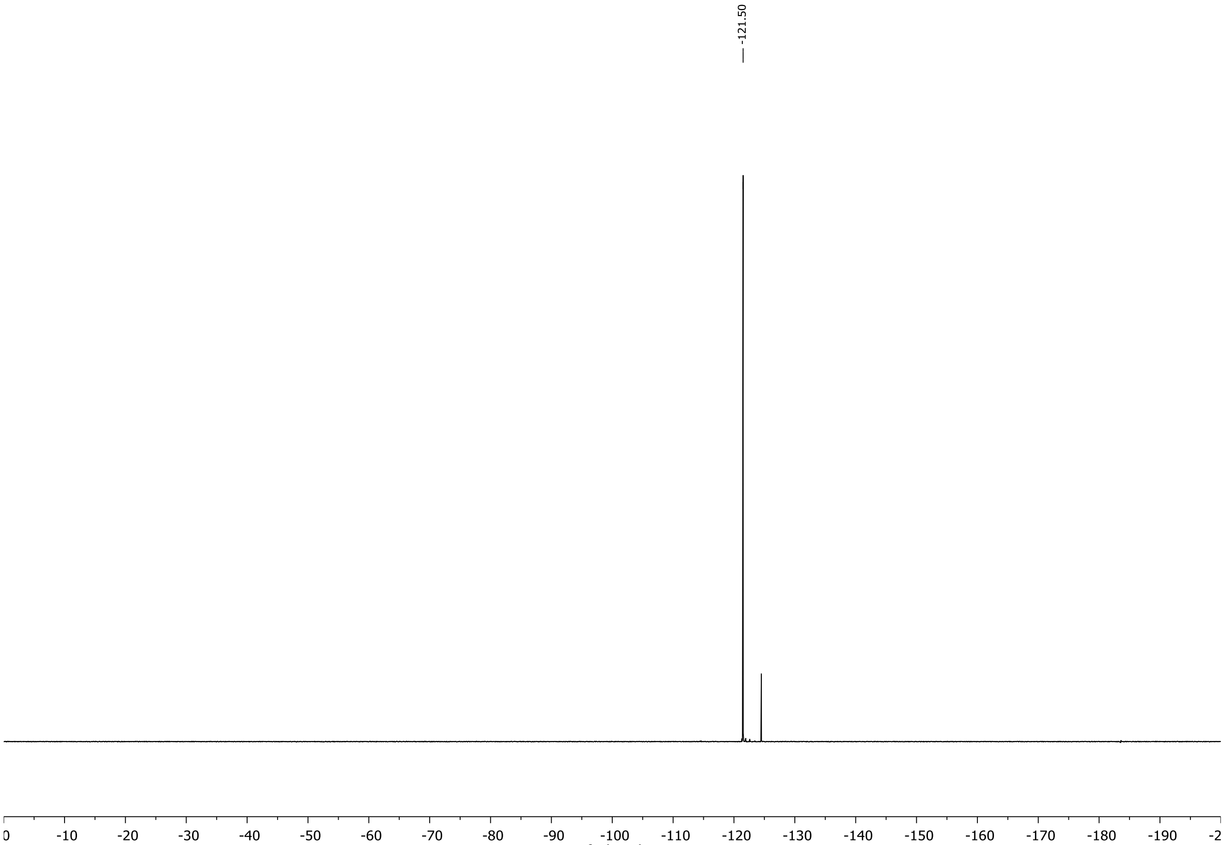

Ethyl (*R*)-2-amino-3-(2-bromobenzyl)-5-methylcyclopenta-1,3-diene-1-carboxylate (3b)

^{1}H NMR

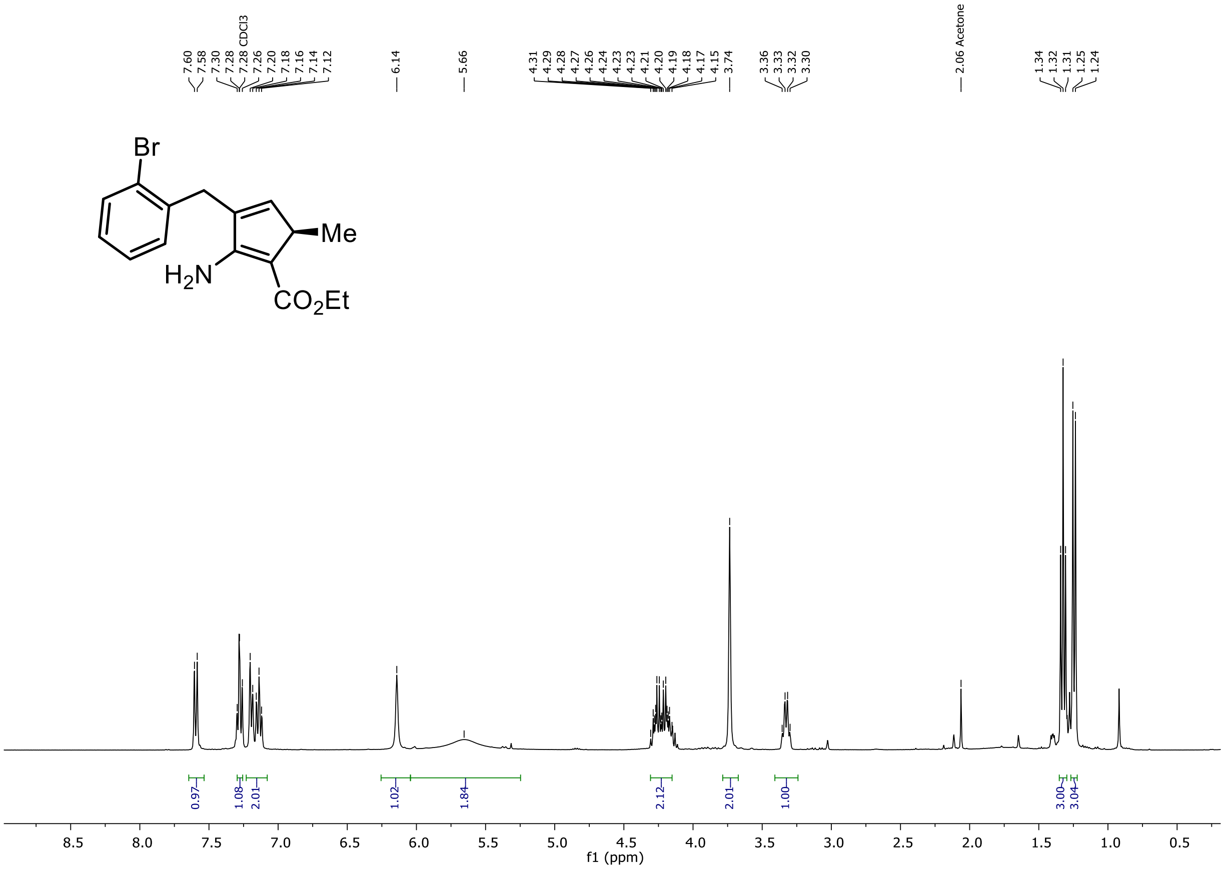

^{13}C NMR

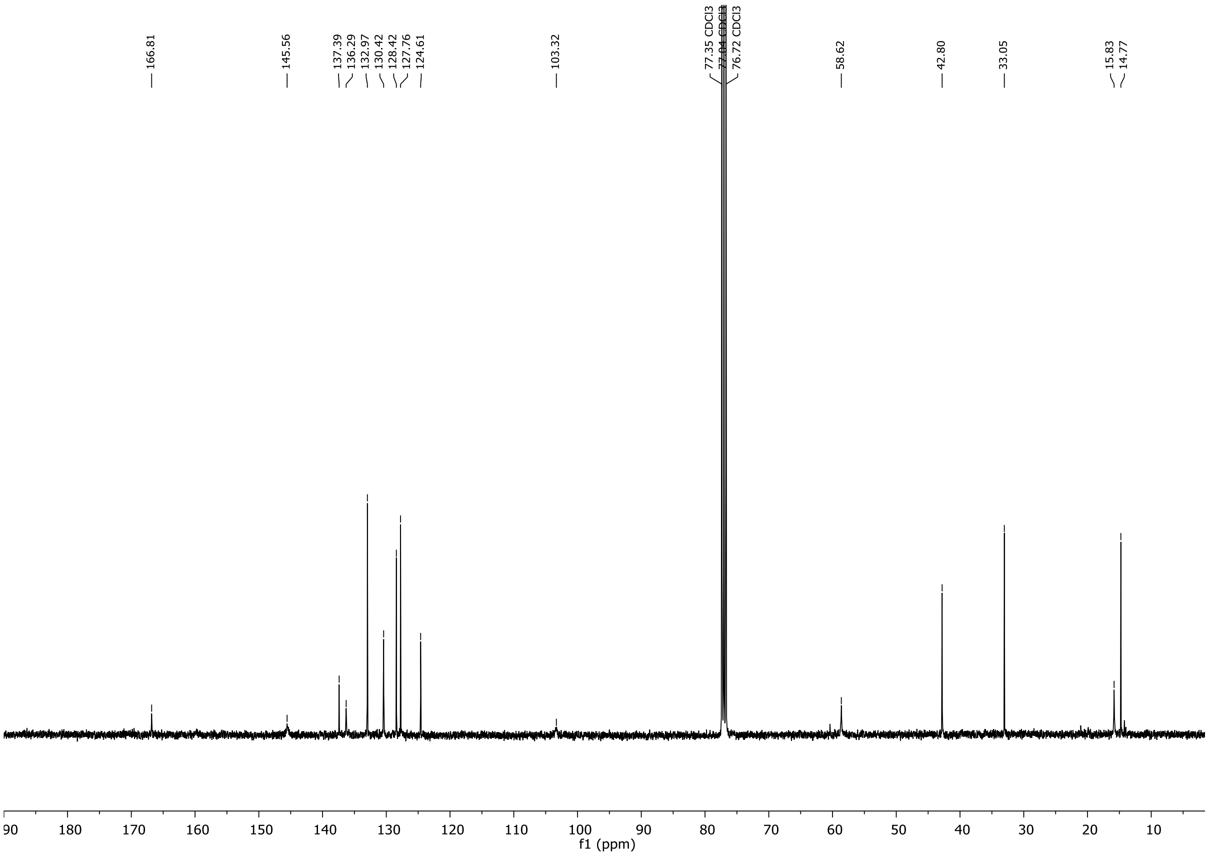

Ethyl (*R*)-2-amino-5-methyl-3-(thiophen-3-ylmethyl)cyclopenta-1,3-diene-1-carboxylate (3c)

^{1}H NMR

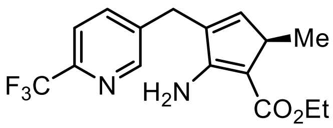

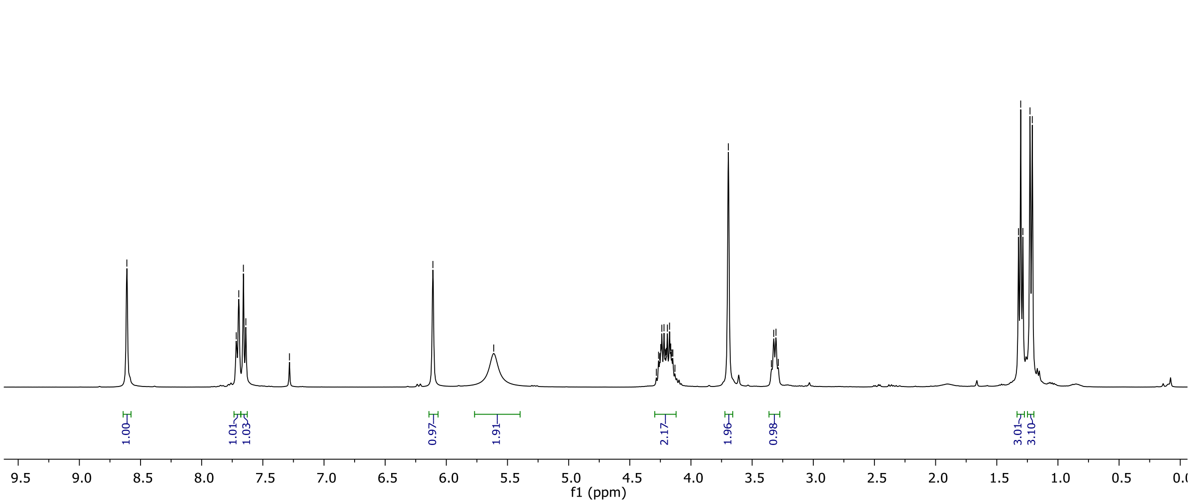

^{13}C NMR

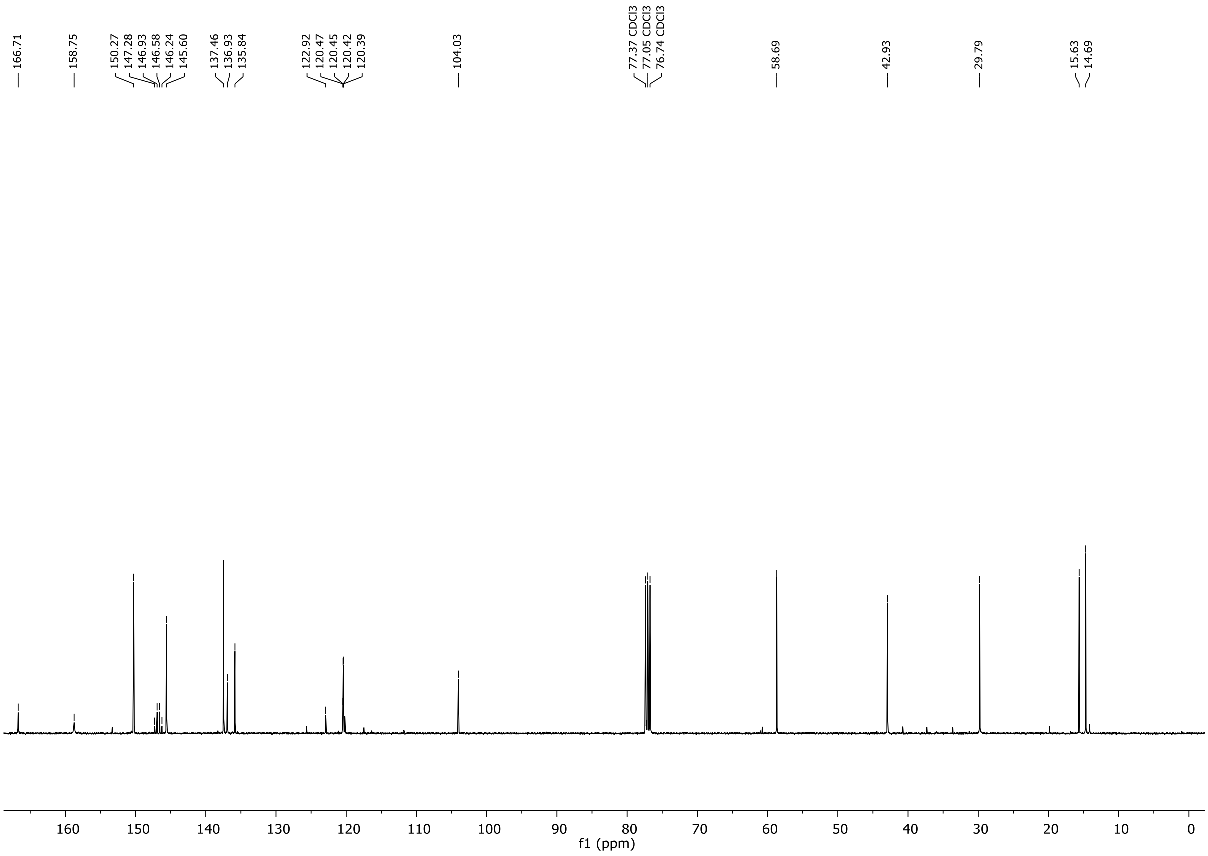

^{19}F NMR

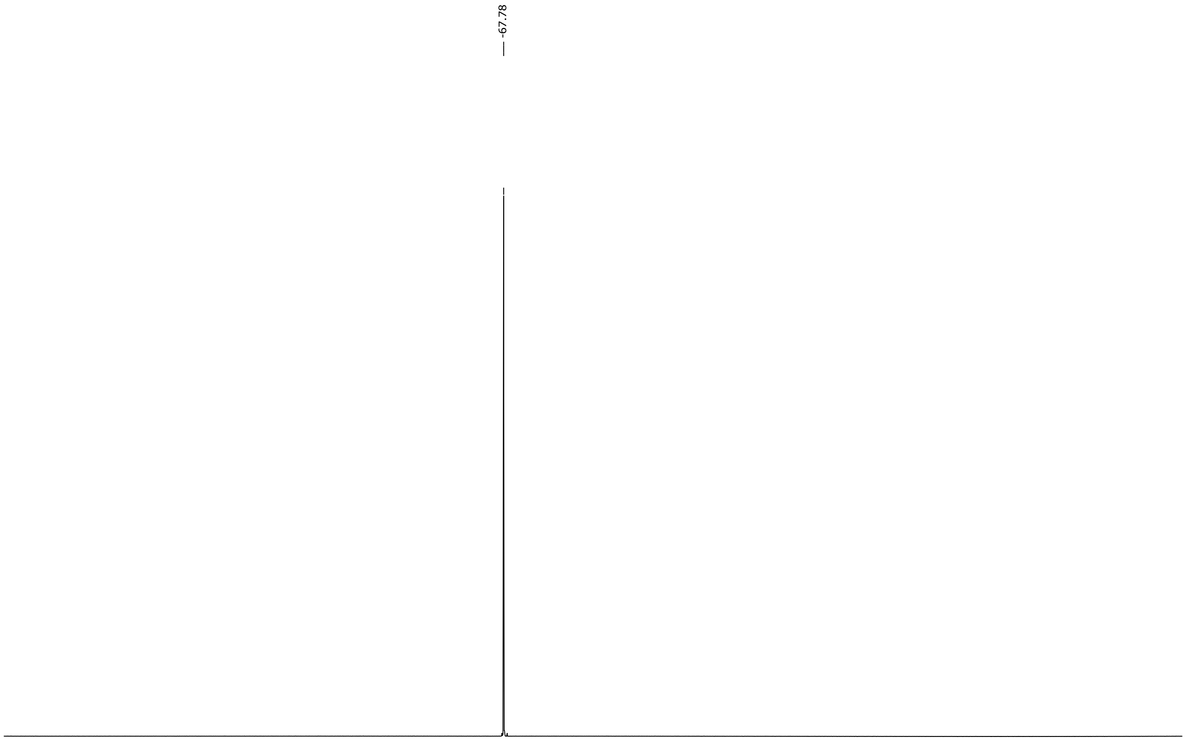

Ethyl (*R*)-2-amino-5-methyl-3-(thiophen-3-ylmethyl)cyclopenta-1,3-diene-1-carboxylate (3d)

^{1}H NMR

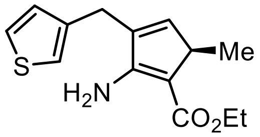

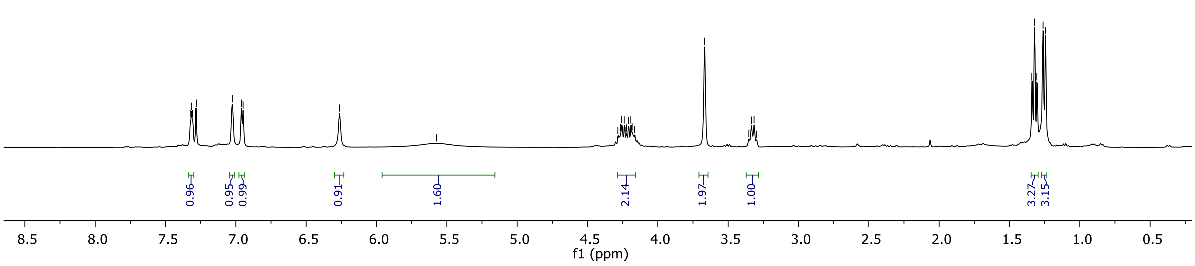

^{13}C NMR

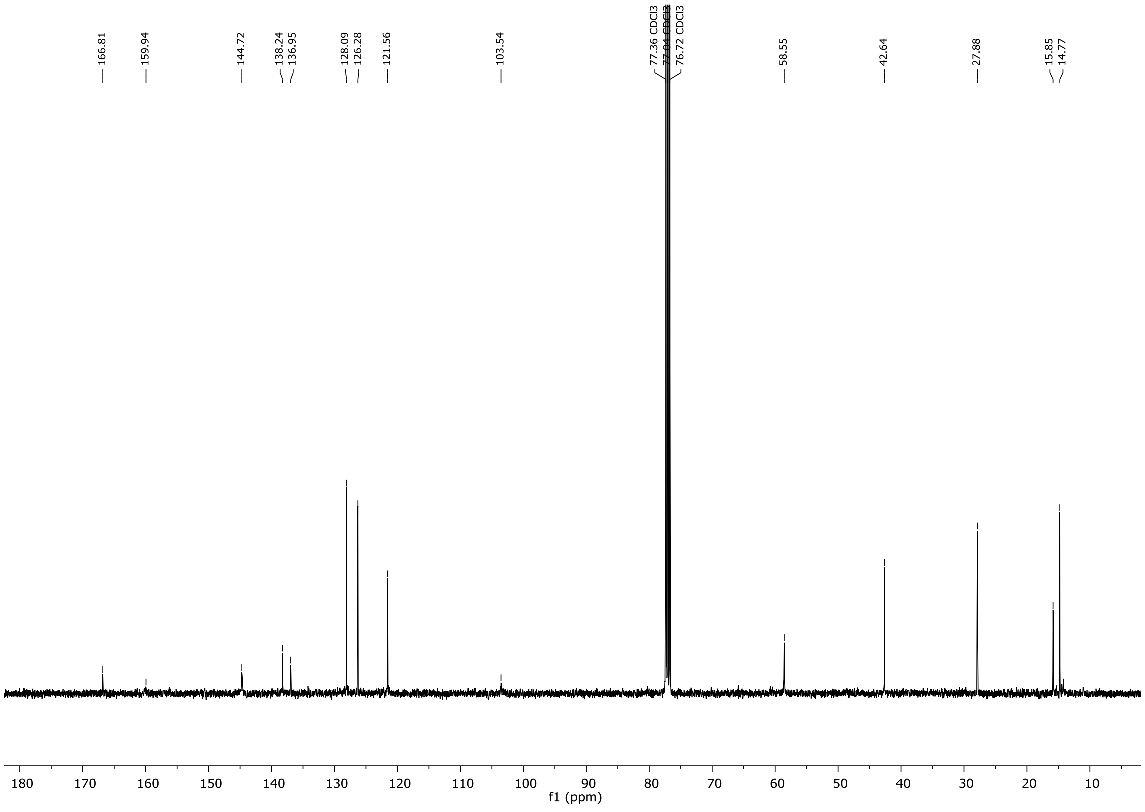

Tert-butyl (_R_)-3-((5-amino-4-(ethoxycarbonyl)-3-methylcyclopenta-1,4-dien-1-yl)methyl)-1H-indole-1-carboxylate (3e)

^{1}H NMR

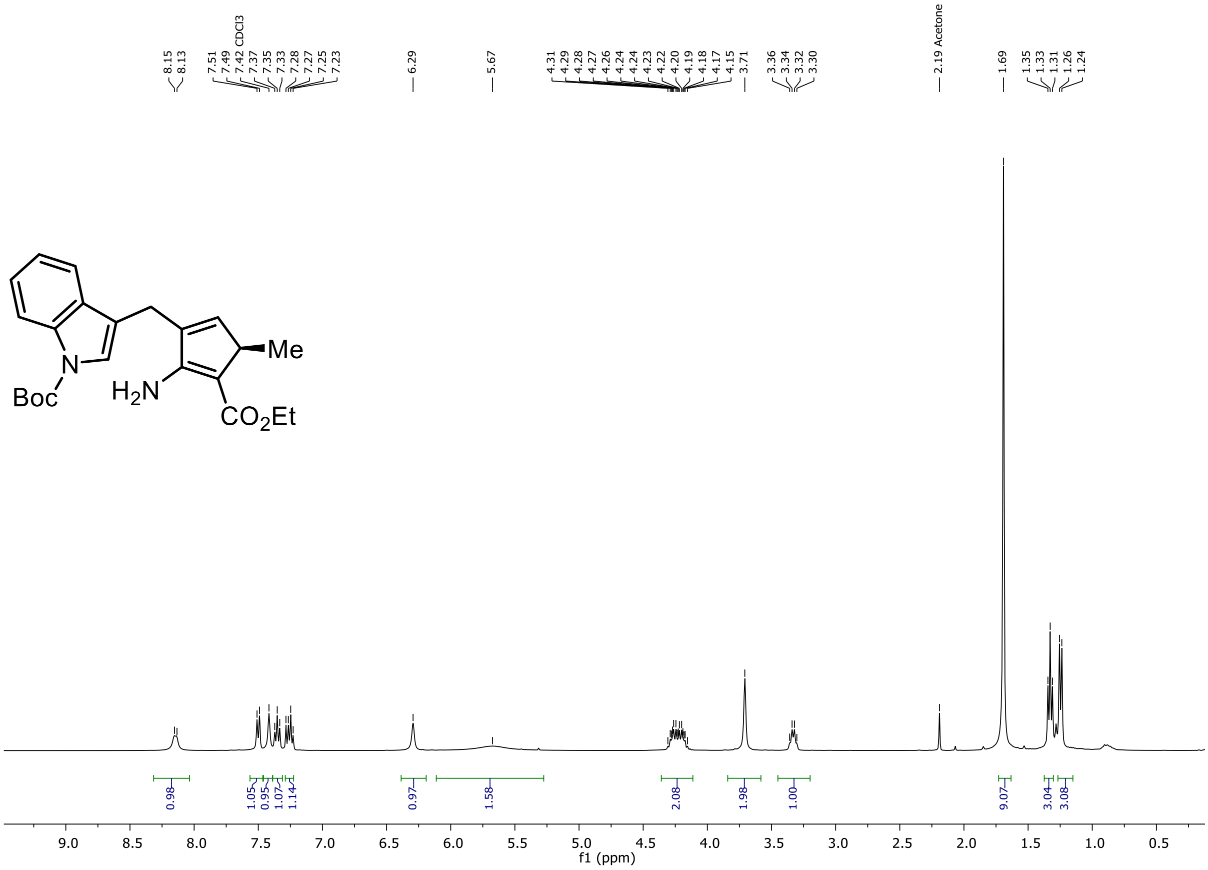

^{13}C NMR

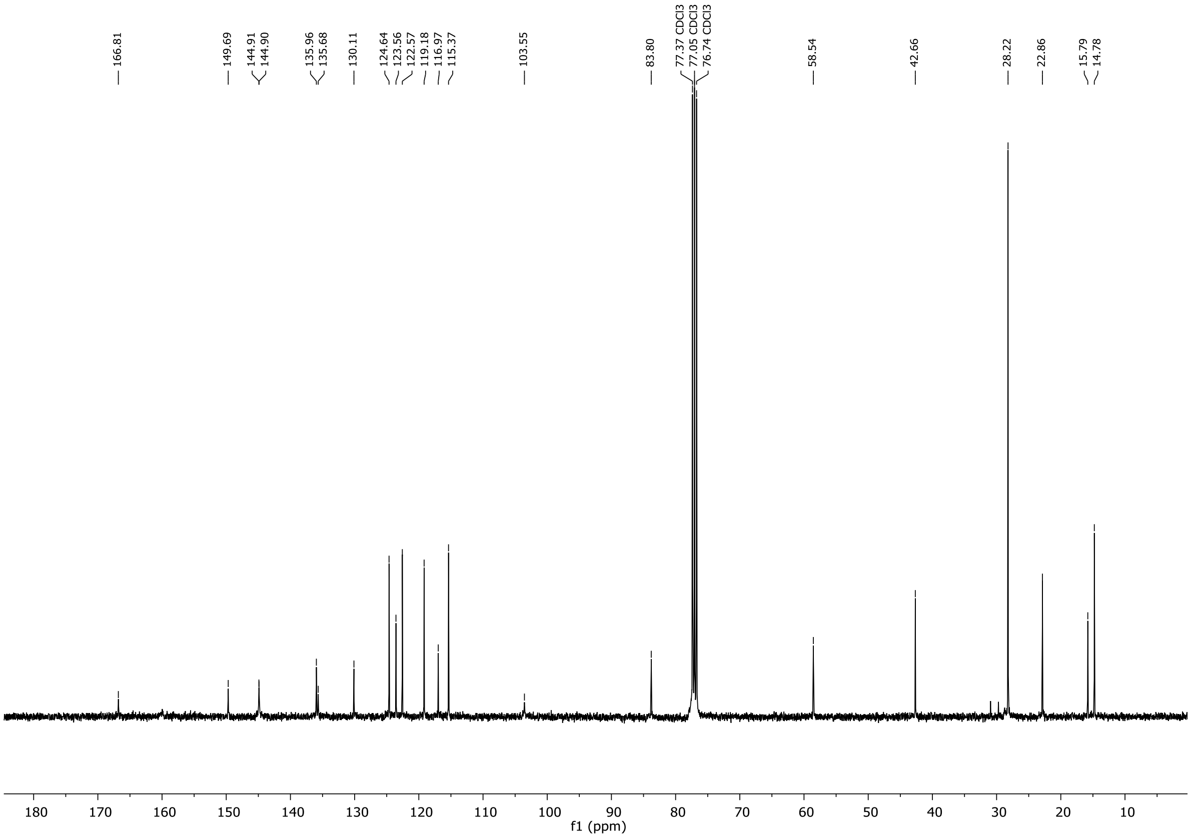

Ethyl (*R*)-2-amino-3-benzhydryl-5-methylcyclopenta-1,3-diene-1-carboxylate (3f)

^{1}H NMR

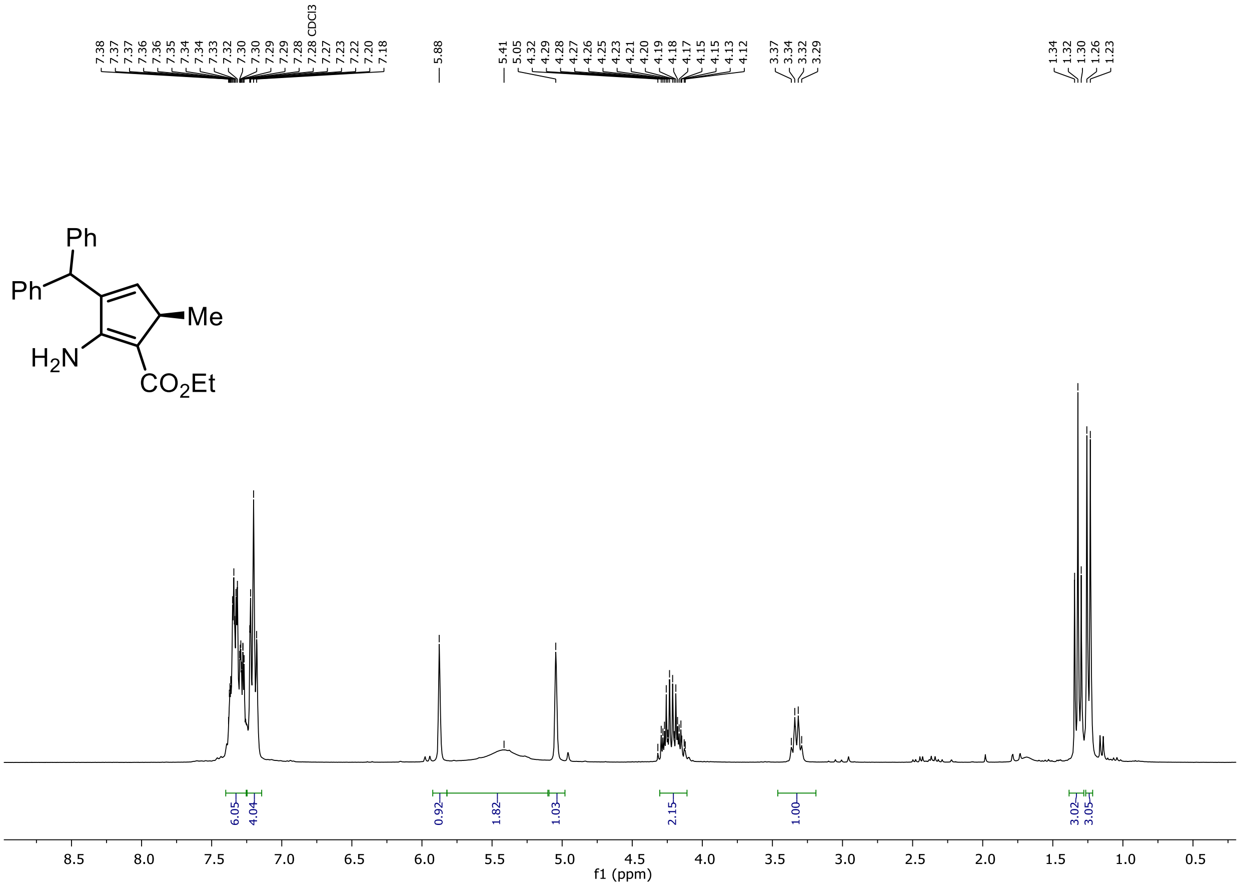

^{13}C NMR

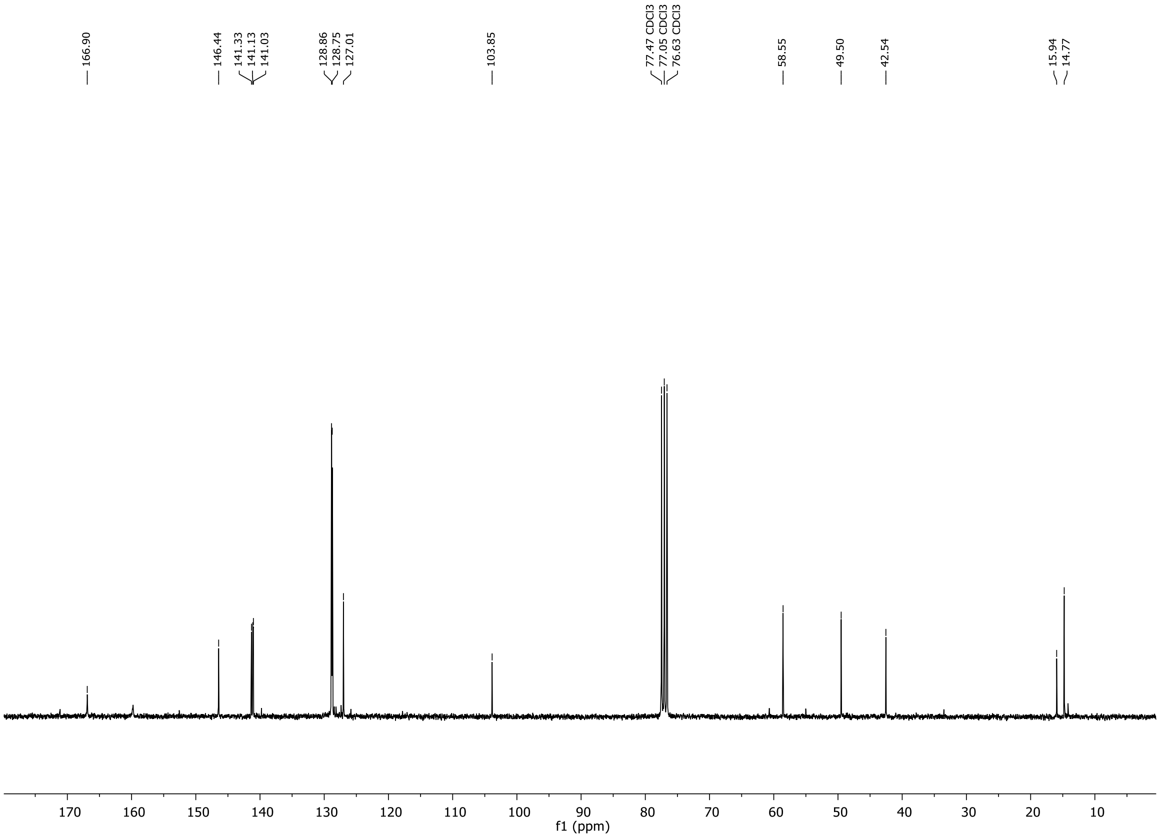

Ethyl (*R*)-2-amino-3-(bis(4-bromophenyl)methyl)-5-methylcyclopenta-1,3-diene-1-carboxylate (3g)

^{1}H NMR

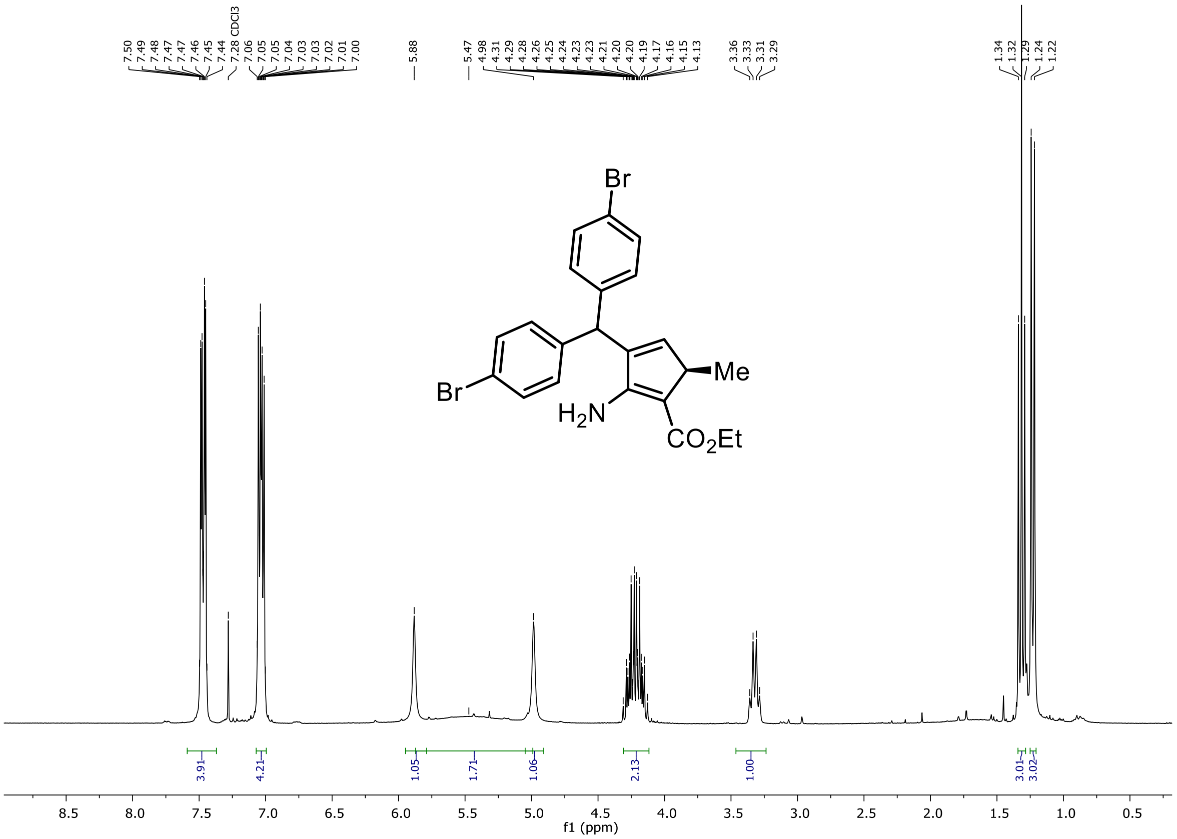

^{13}C NMR

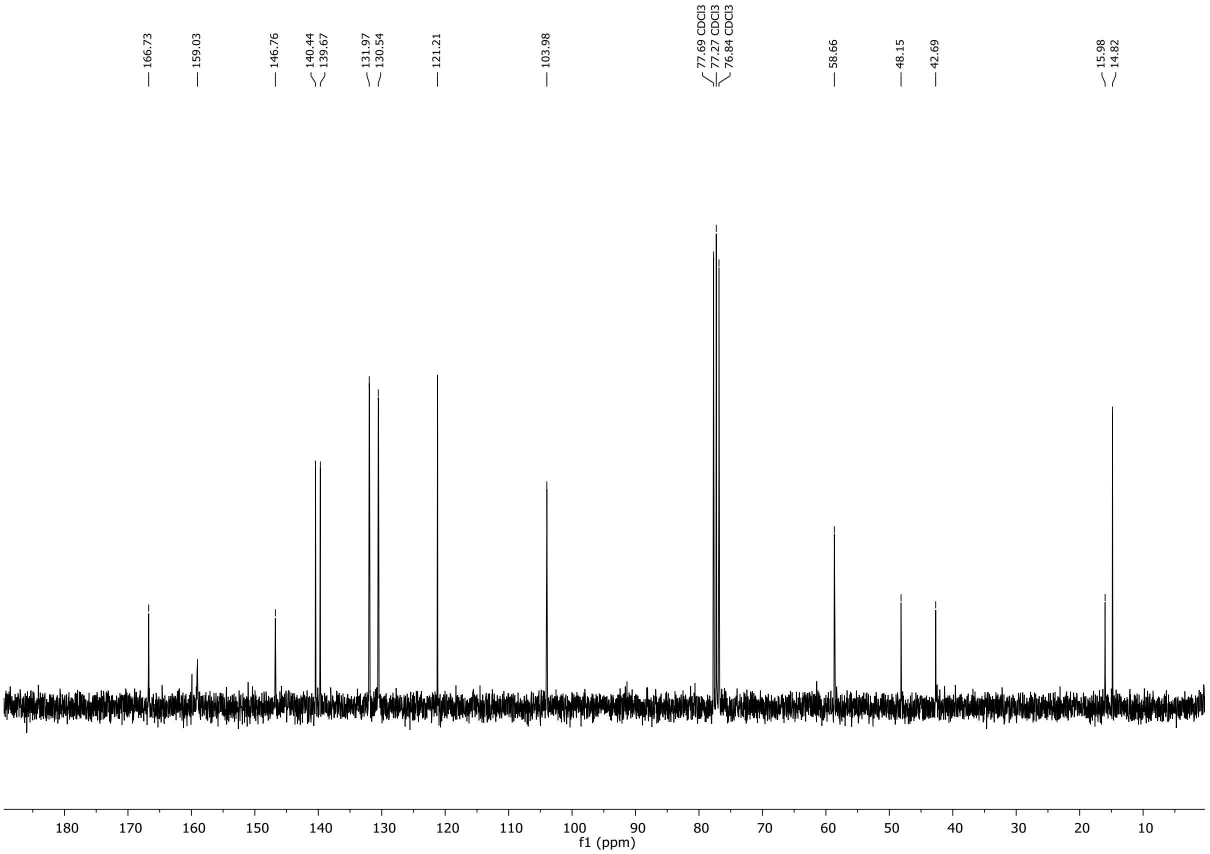

Ethyl (*R*)-2-amino-3-isopropyl-5-methylcyclopenta-1,3-diene-1-carboxylate (3h)

^{1}H NMR

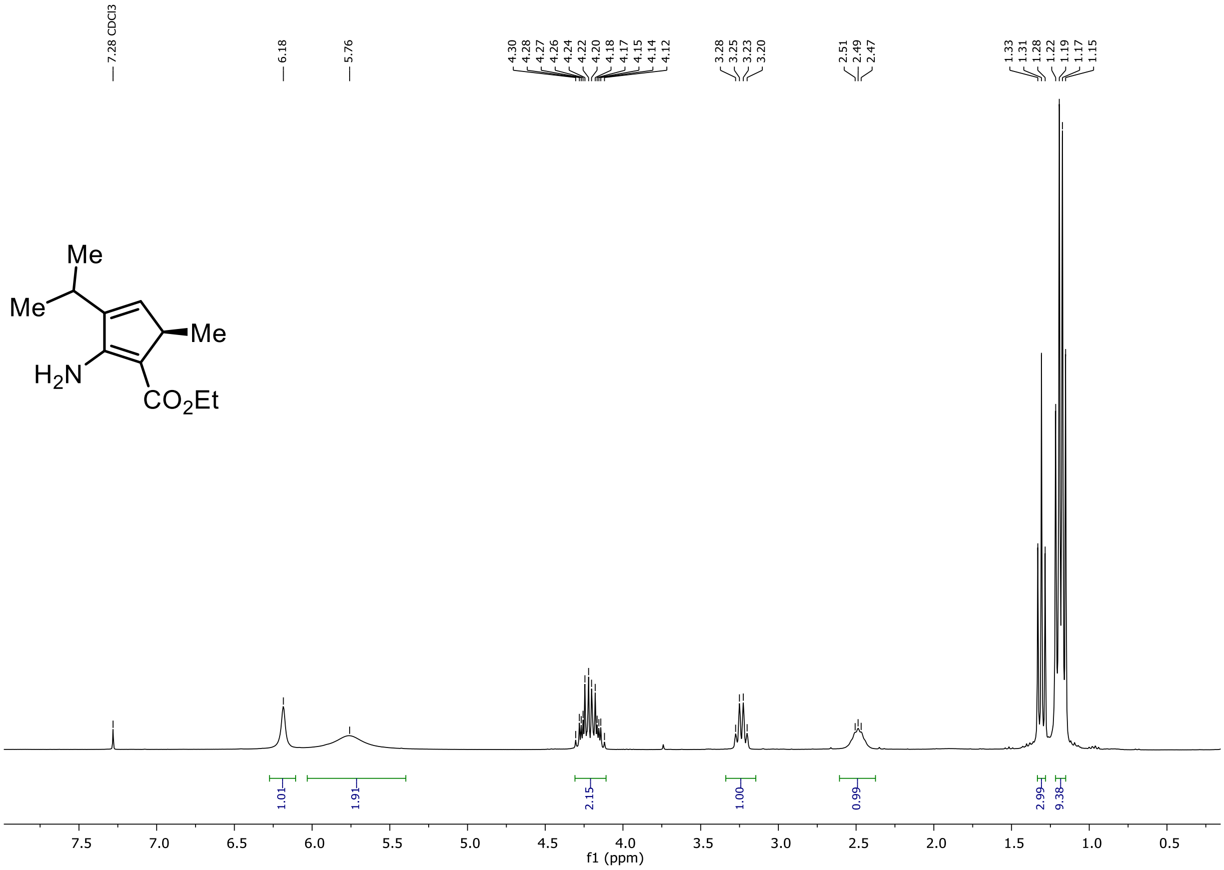

^{13}C NMR

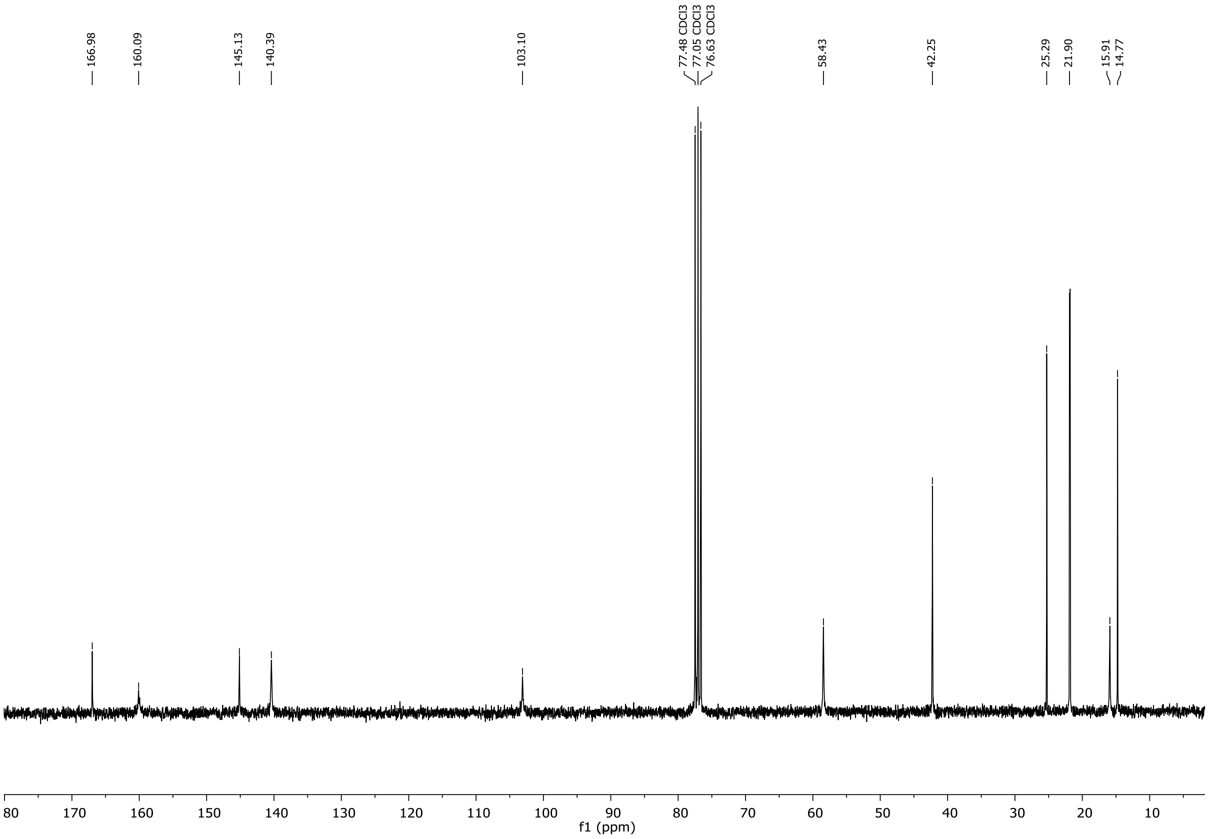

Ethyl (*R*)-2-amino-5-methyl-3-(3-phenylpropyl)cyclopenta-1,3-diene-1-carboxylate (3i)

^{1}H NMR

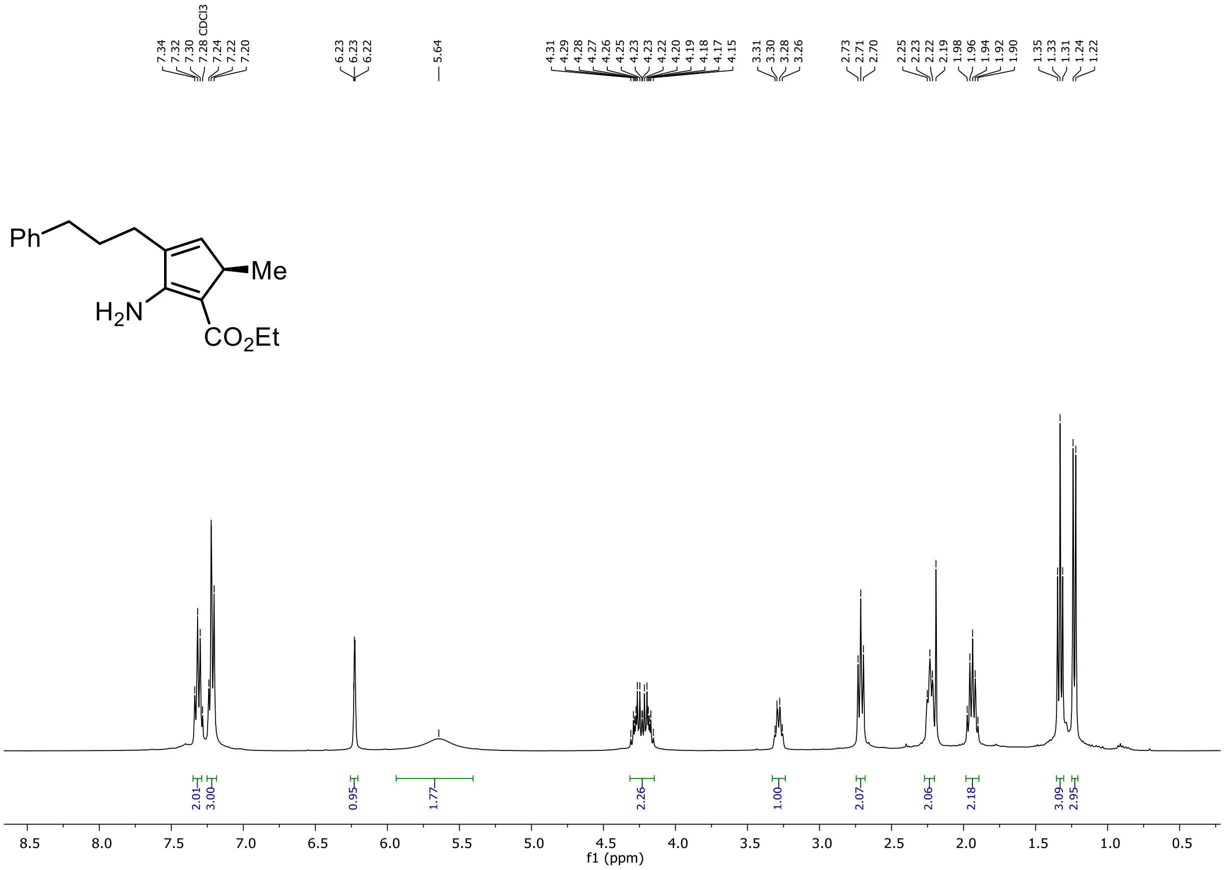

^{13}C NMR

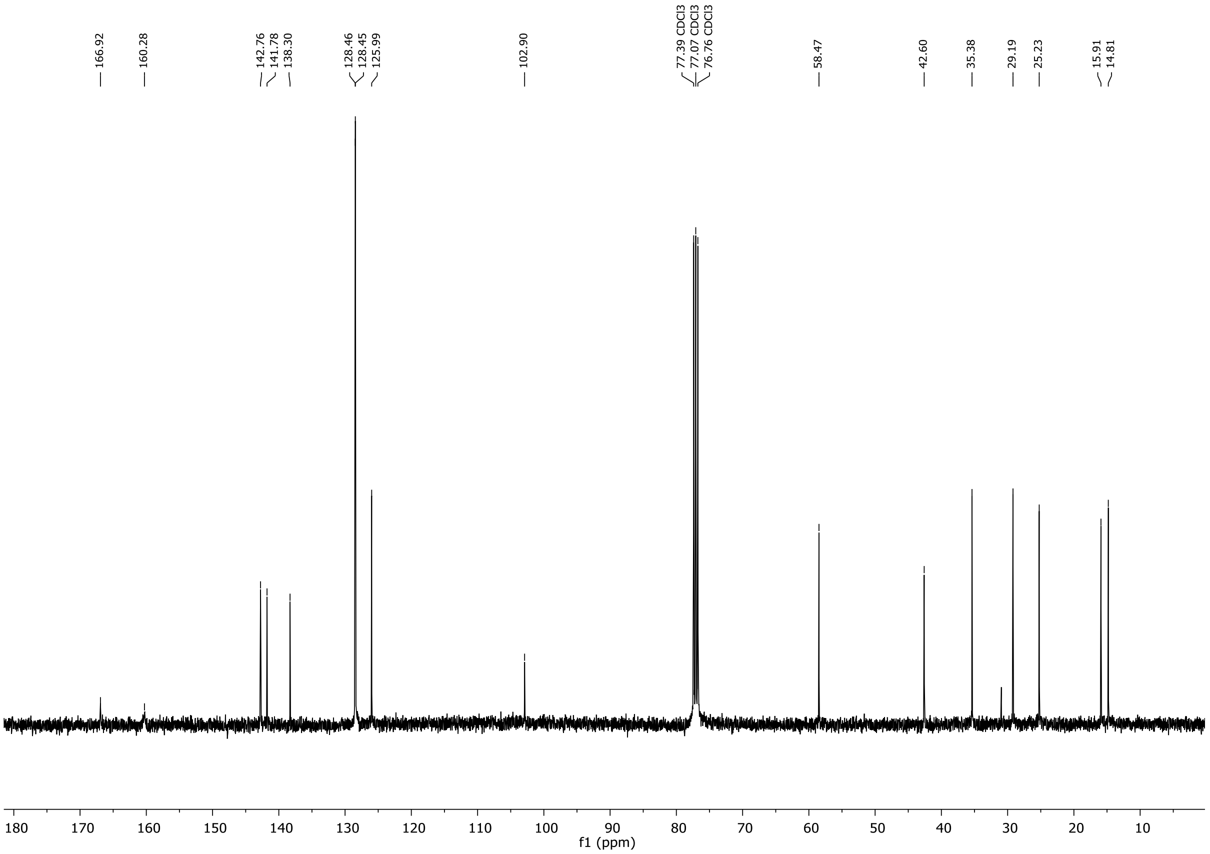

Ethyl (*R*)-3-(2-(1,3-dioxolan-2-yl)ethyl)-2-amino-5-methylcyclopenta-1,3-diene-1-carboxylate (3j)

^{1}H NMR

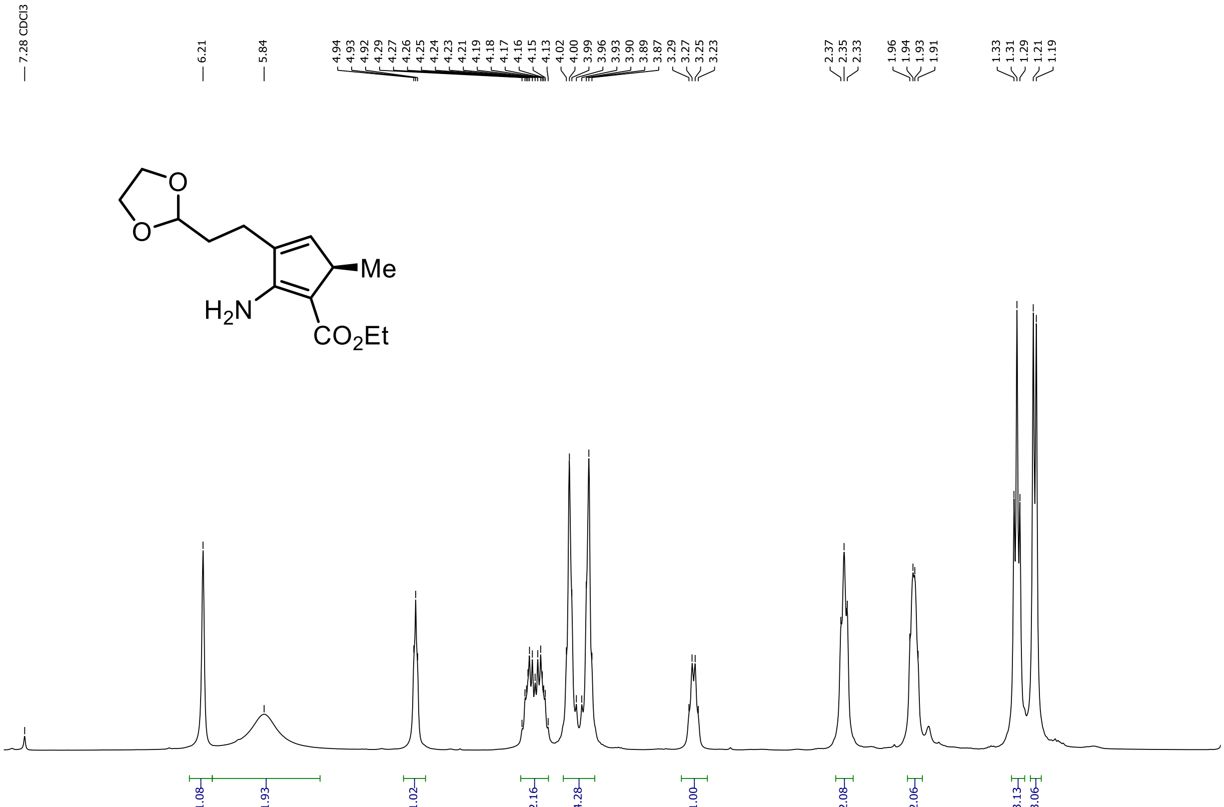

^{13}C NMR

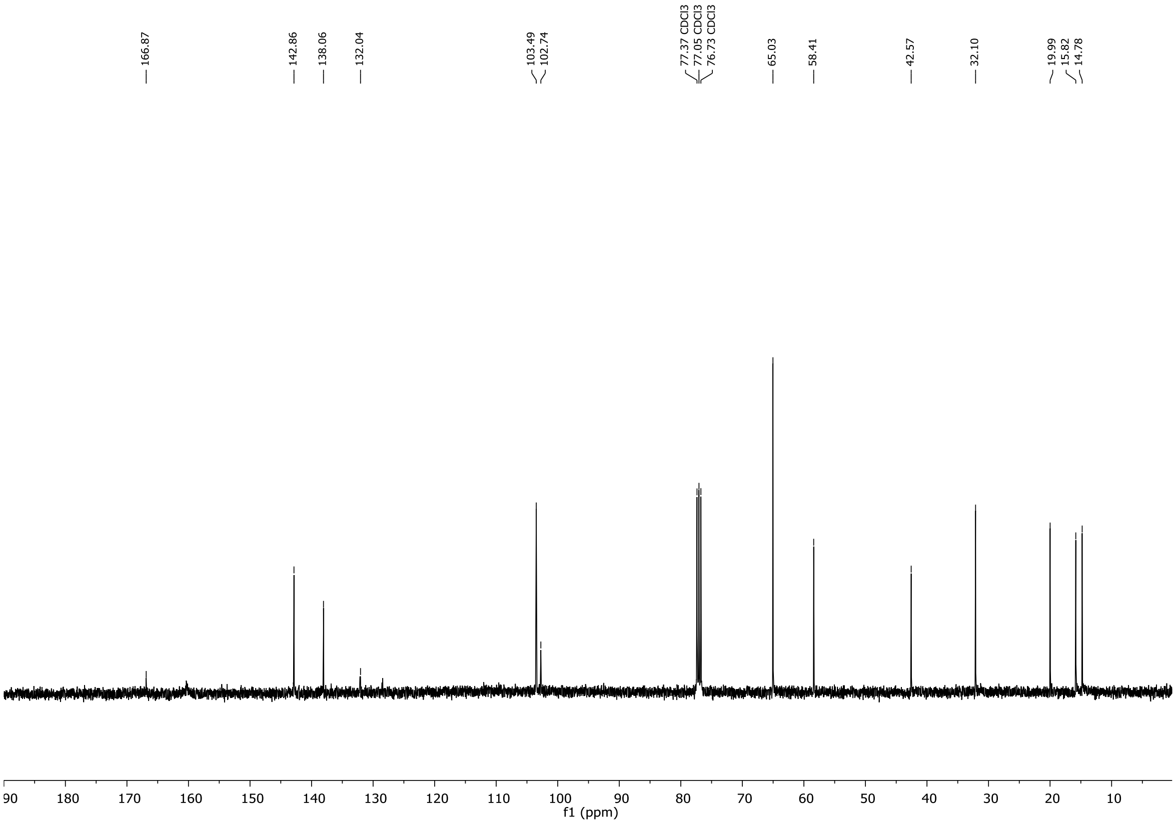

Ethyl (*R*)-2-amino-3-(2-((tert-butyldimethylsilyl)oxy)ethyl)-5-methylcyclopenta-1,3-diene-1-carboxylate (3k)

^{1}H NMR

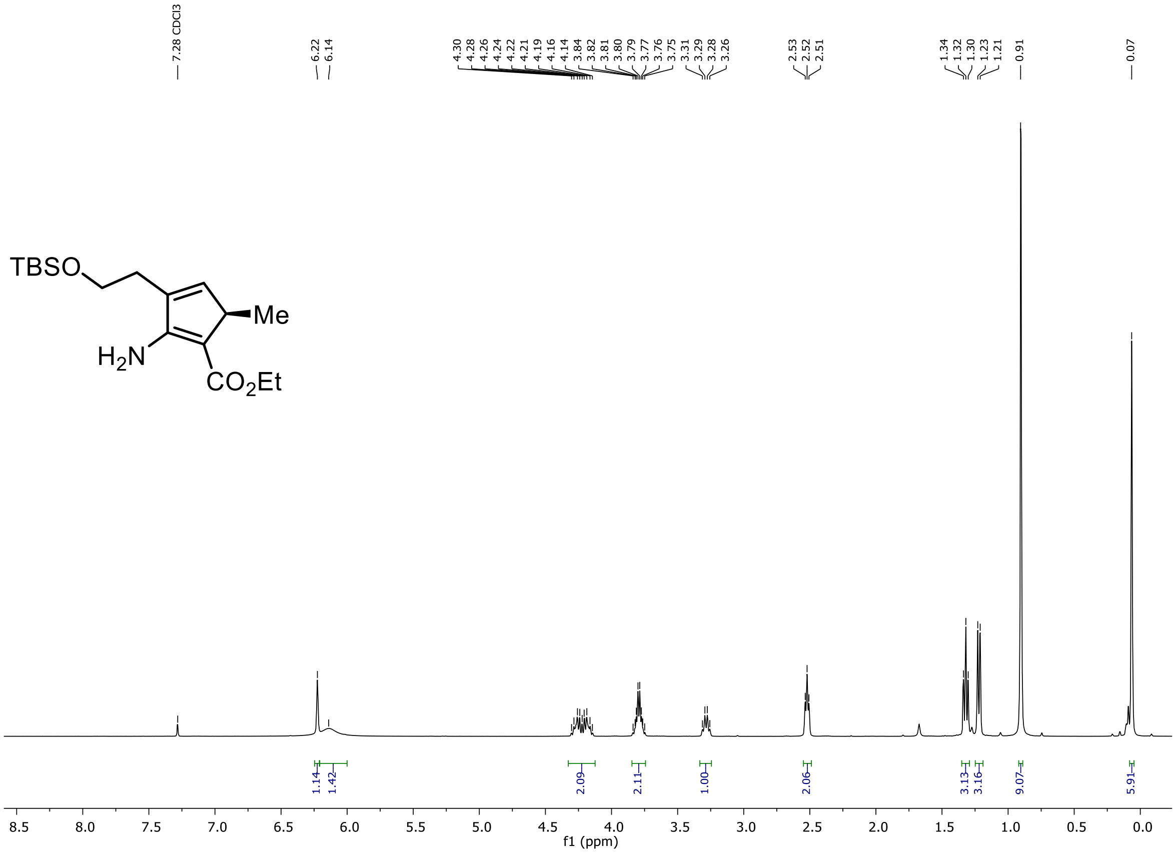

^{13}C NMR

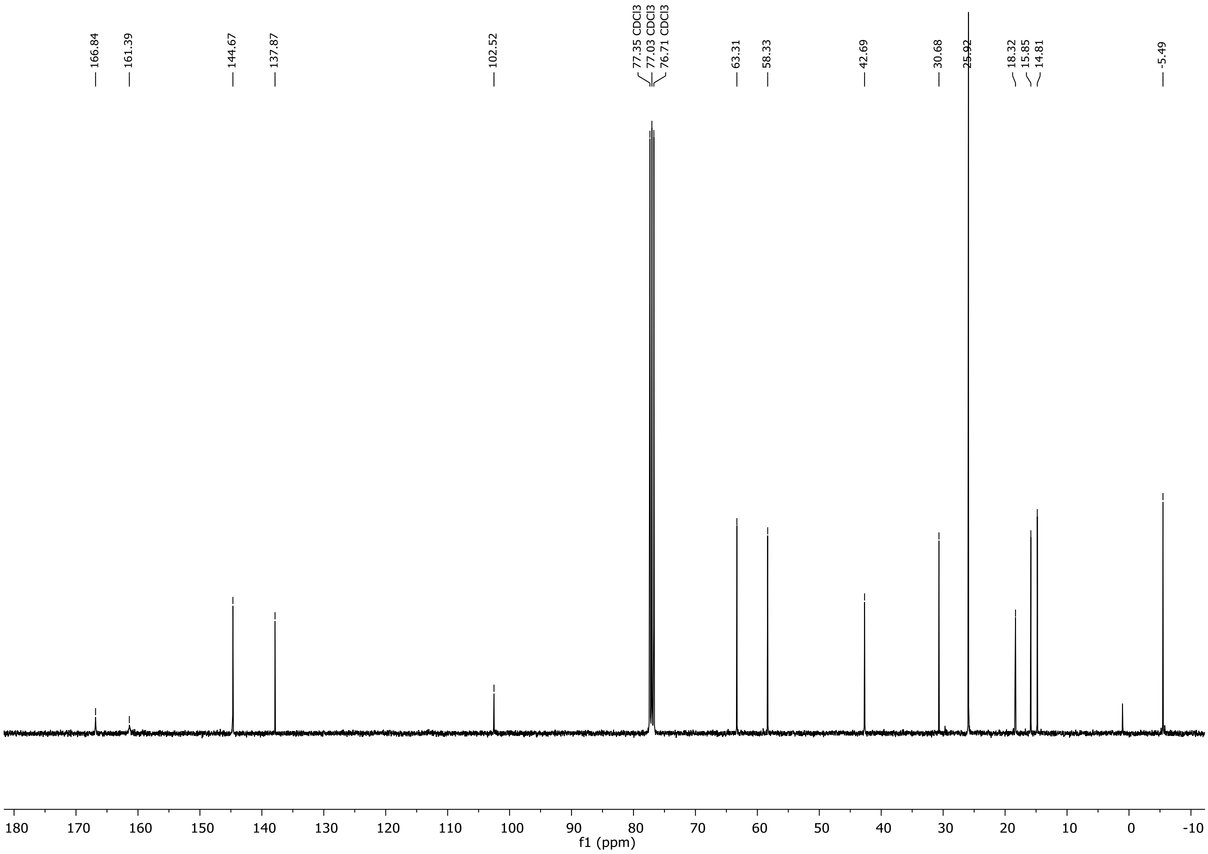

Ethyl (*R*)-3-allyl-2-amino-5-methylcyclopenta-1,3-diene-1-carboxylate (3l)

¹H NMR

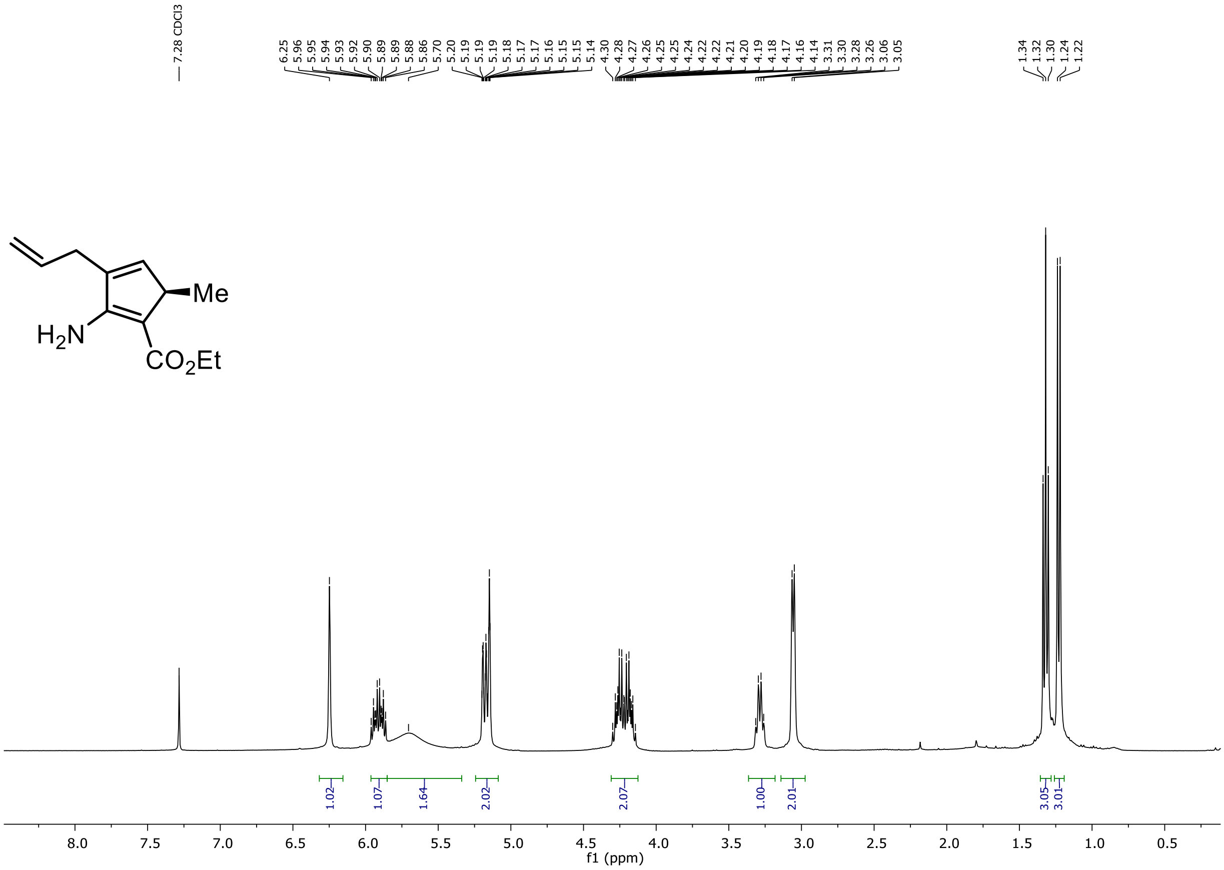

¹³C NMR

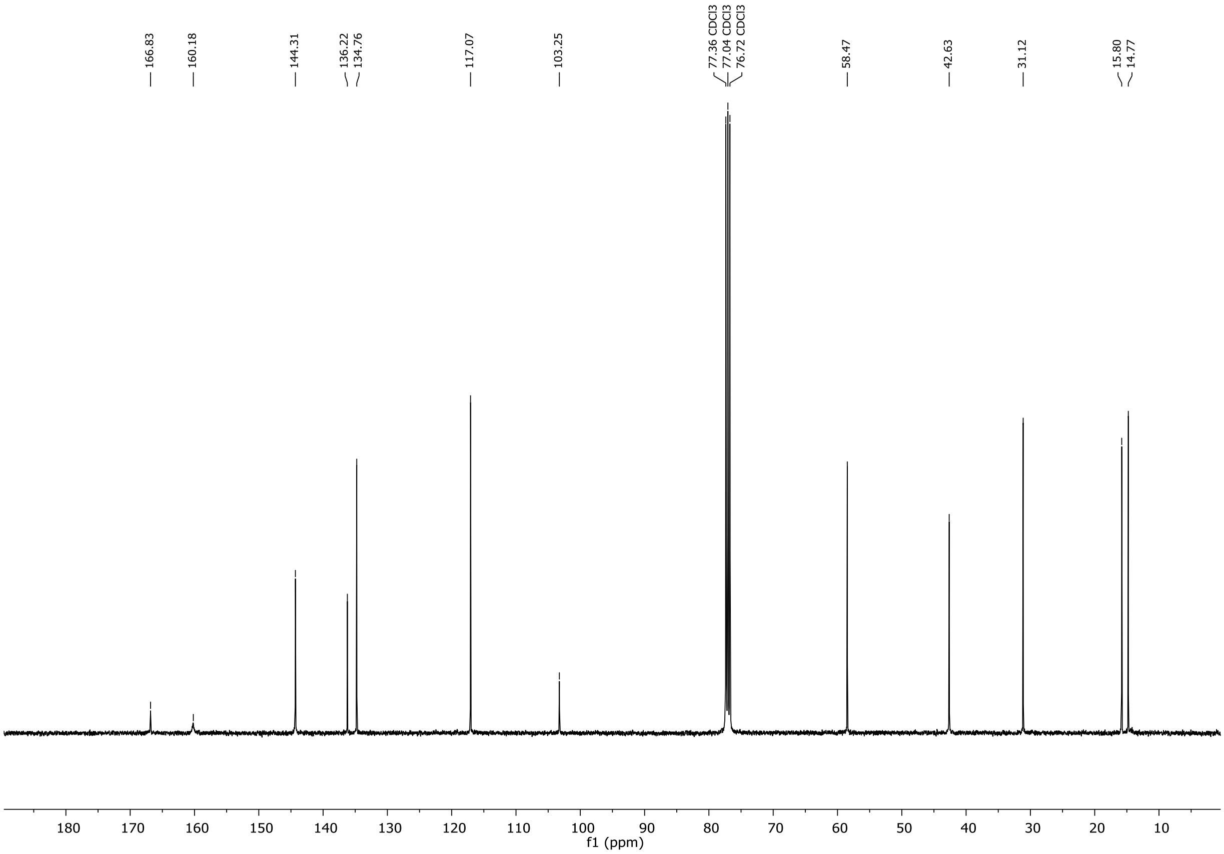

Ethyl (*R*)-2-amino-3,5-dimethylcyclopenta-1,3-diene-1-carboxylate (3m)

^{1}H NMR

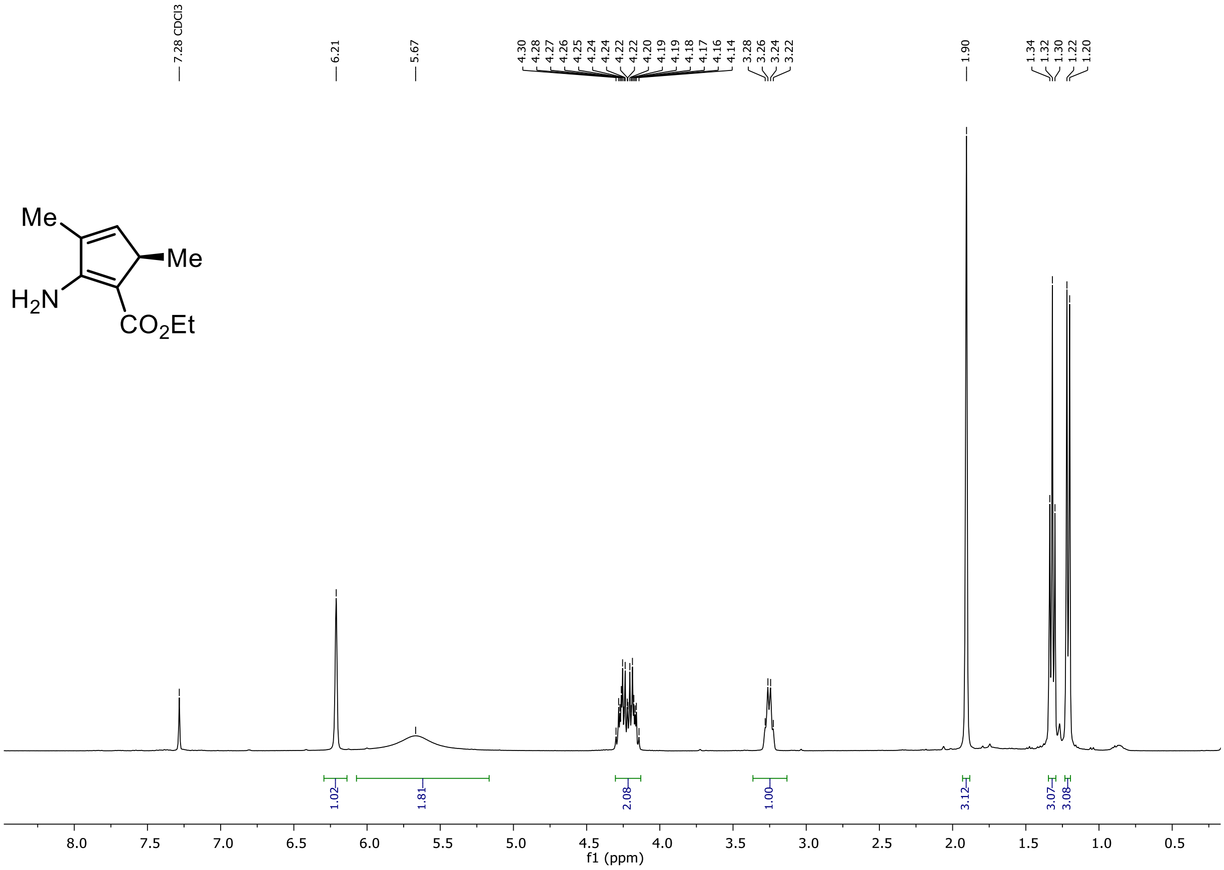

^{13}C NMR

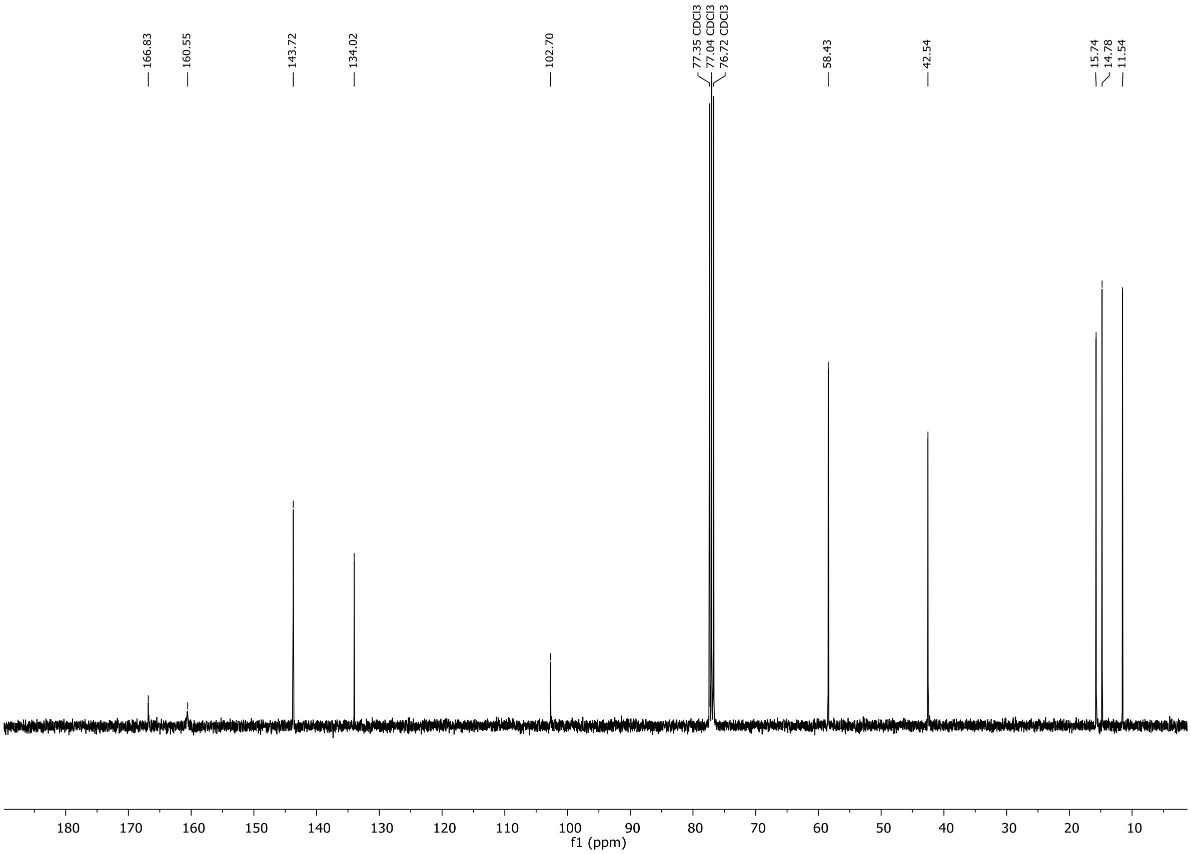

Ethyl (*R*)-2-amino-5-methyl-3-phenylcyclopenta-1,3-diene-1-carboxylate (3n)

^{1}H NMR

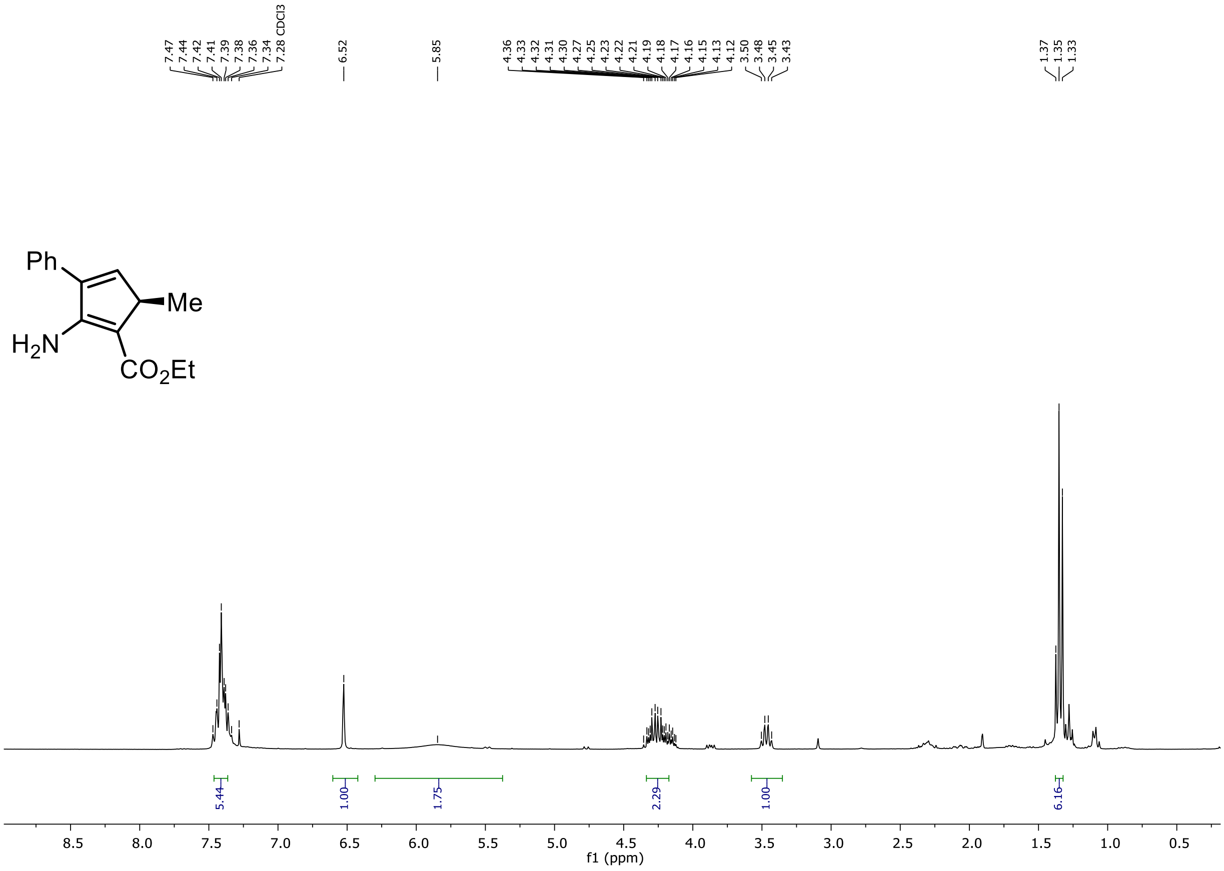

^{13}C NMR

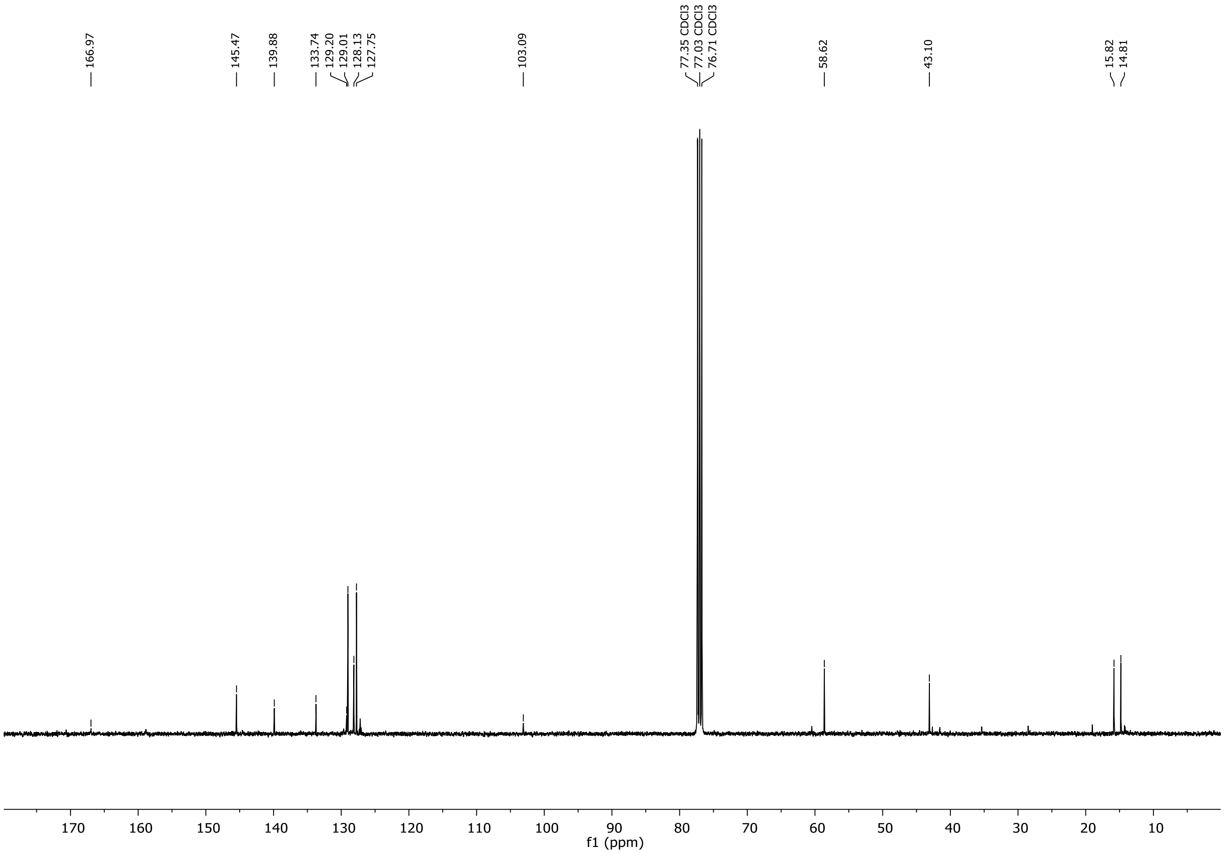

Ethyl (*R*)-2-amino-3-(4-methoxyphenyl)-5-methylcyclopenta-1,3-diene-1-carboxylate (3o)

^{1}H NMR

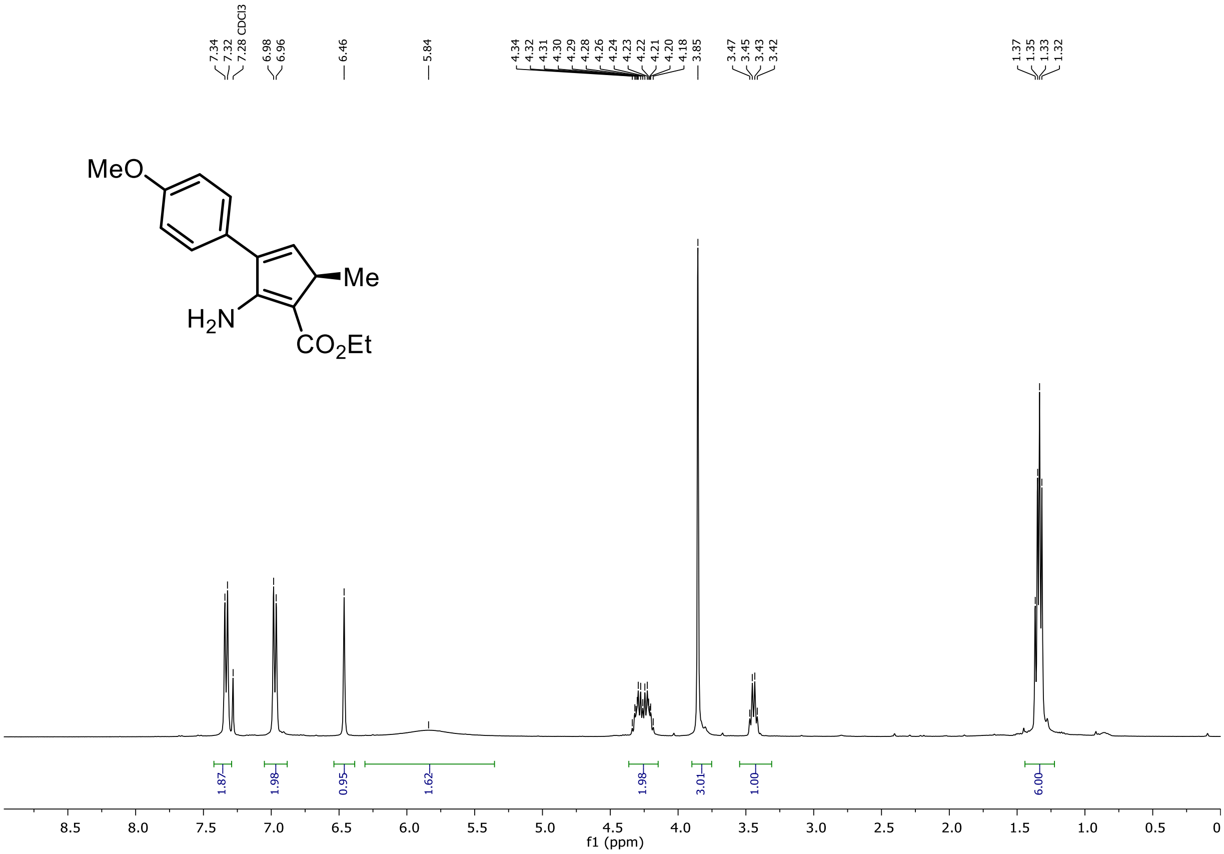

^{13}C NMR

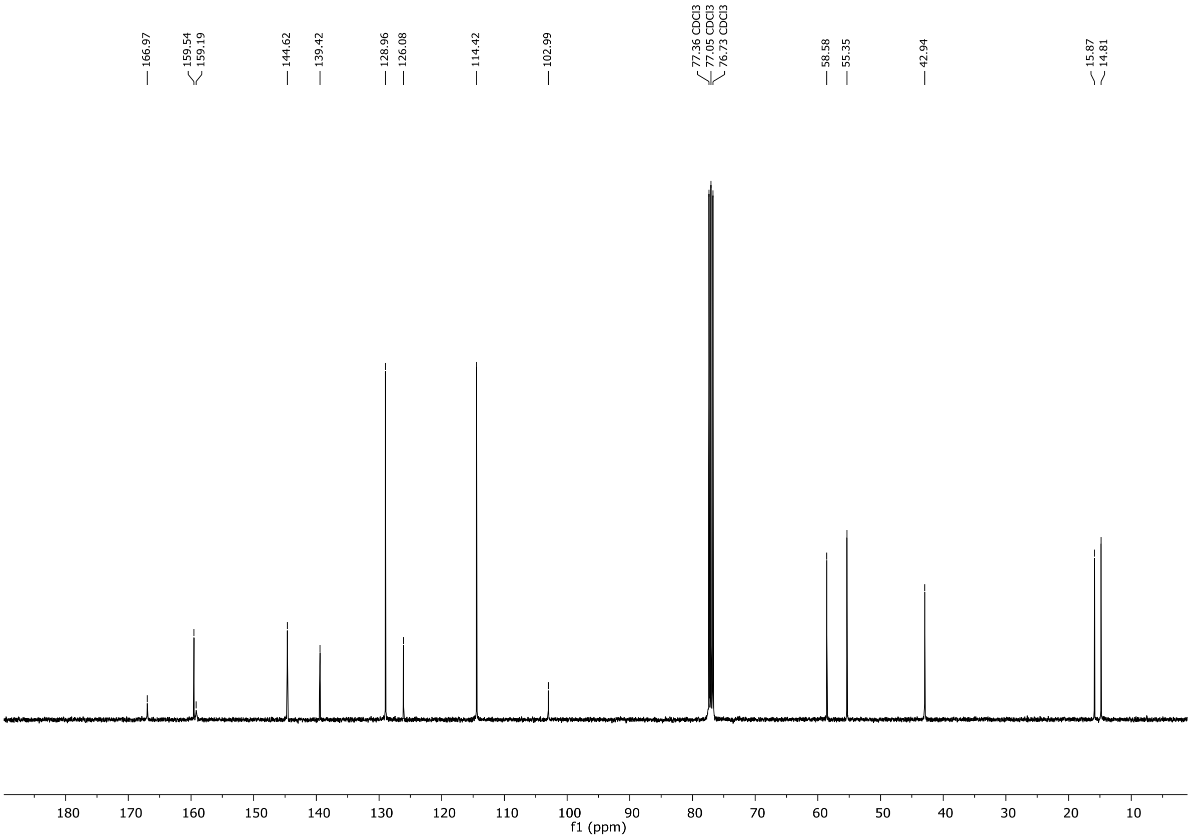

Ethyl (*R*)-2-amino-5-methyl-3-(m-tolyl)cyclopenta-1,3-diene-1-carboxylate (3p)

^{1}H NMR

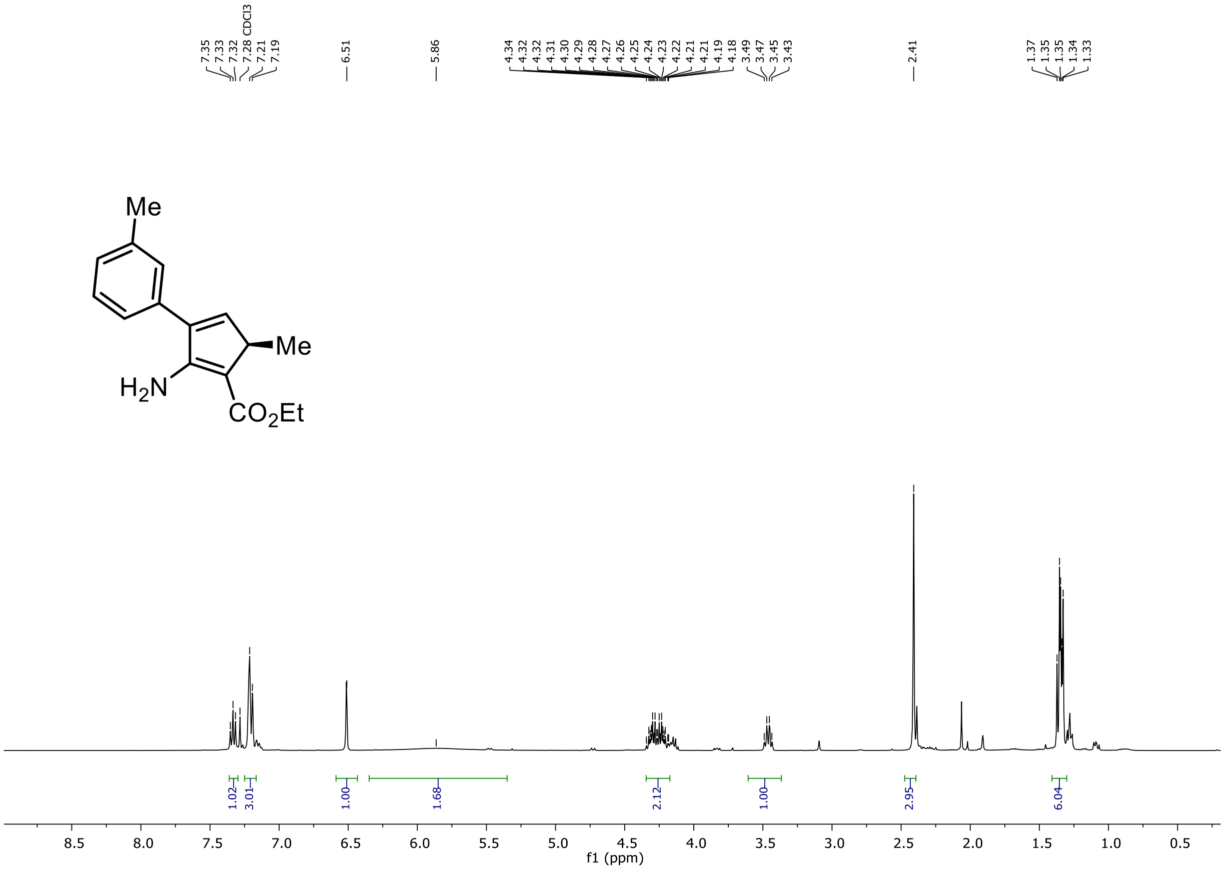

^{13}C NMR

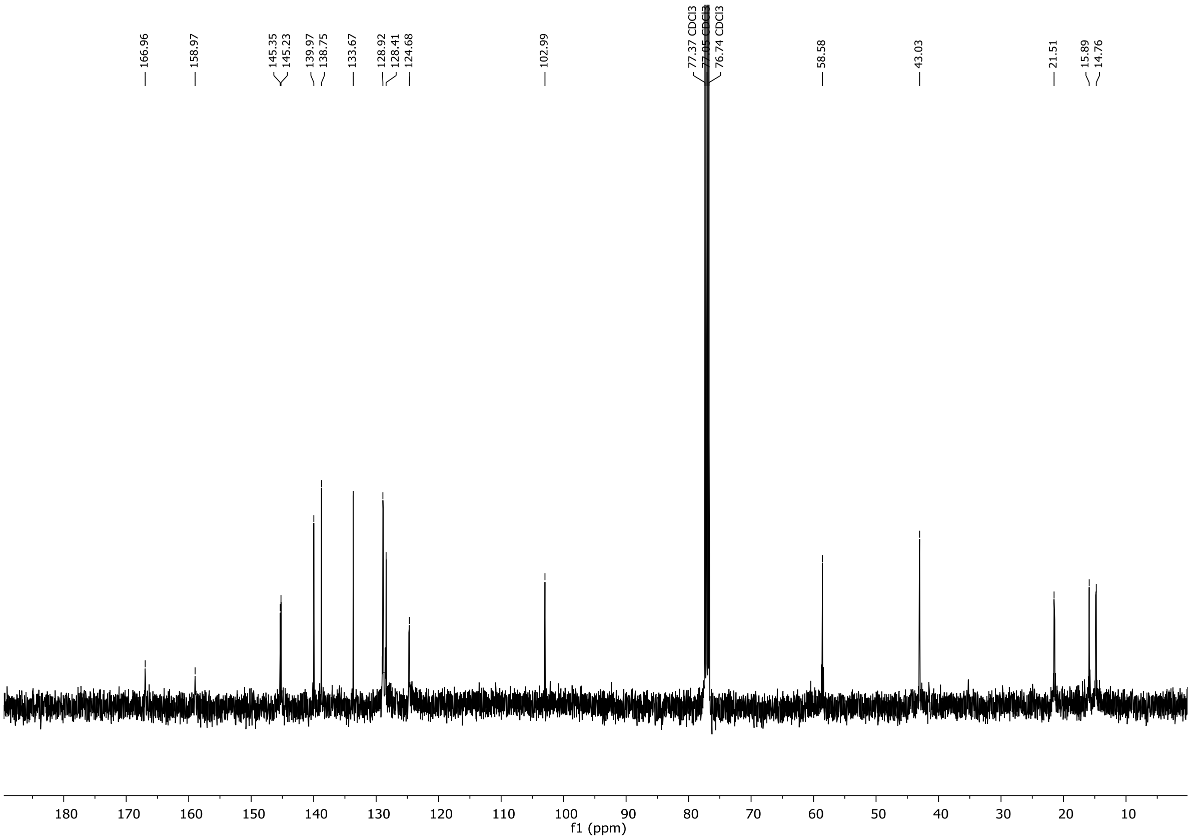

Ethyl (*R*)-2-amino-3-fluoro-5-methylcyclopenta-1,3-diene-1-carboxylate (3q)

^{1}H NMR

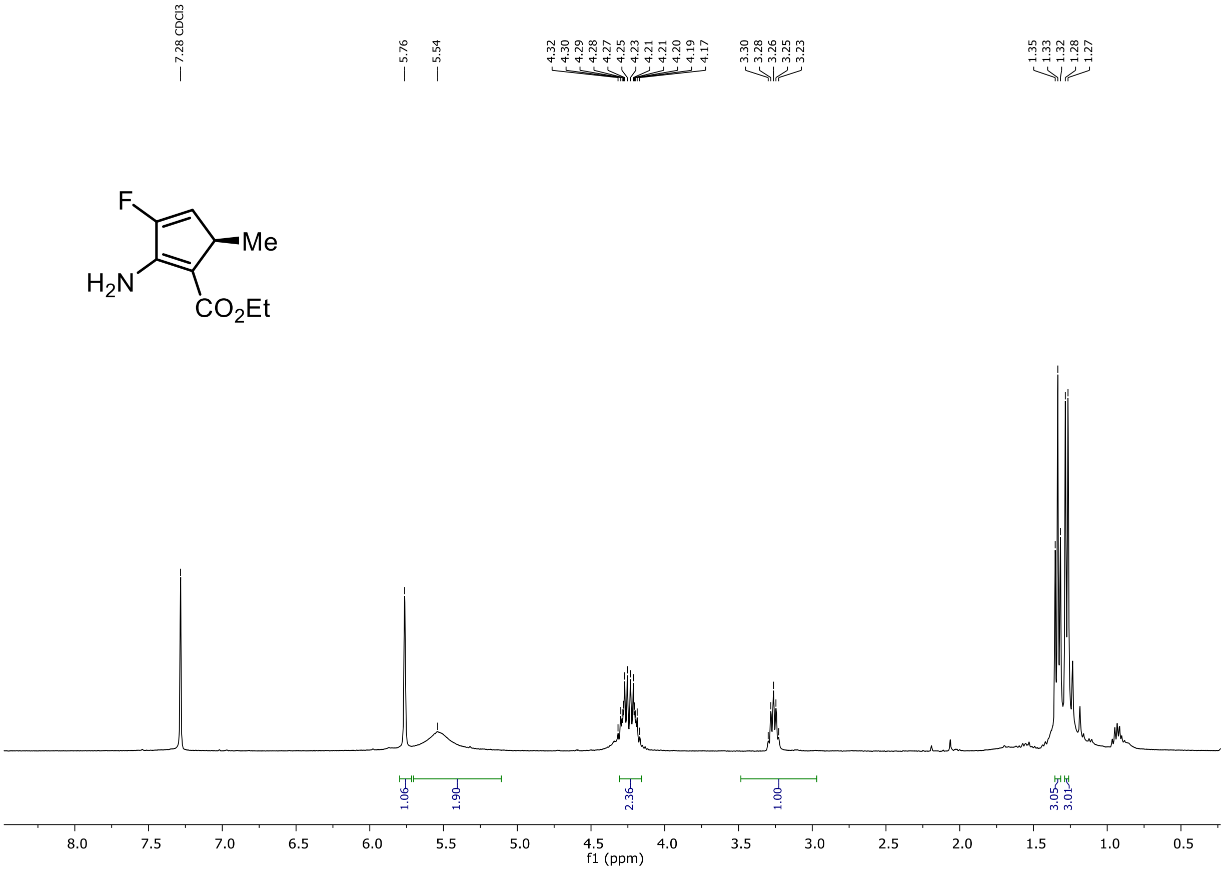

^{13}C NMR

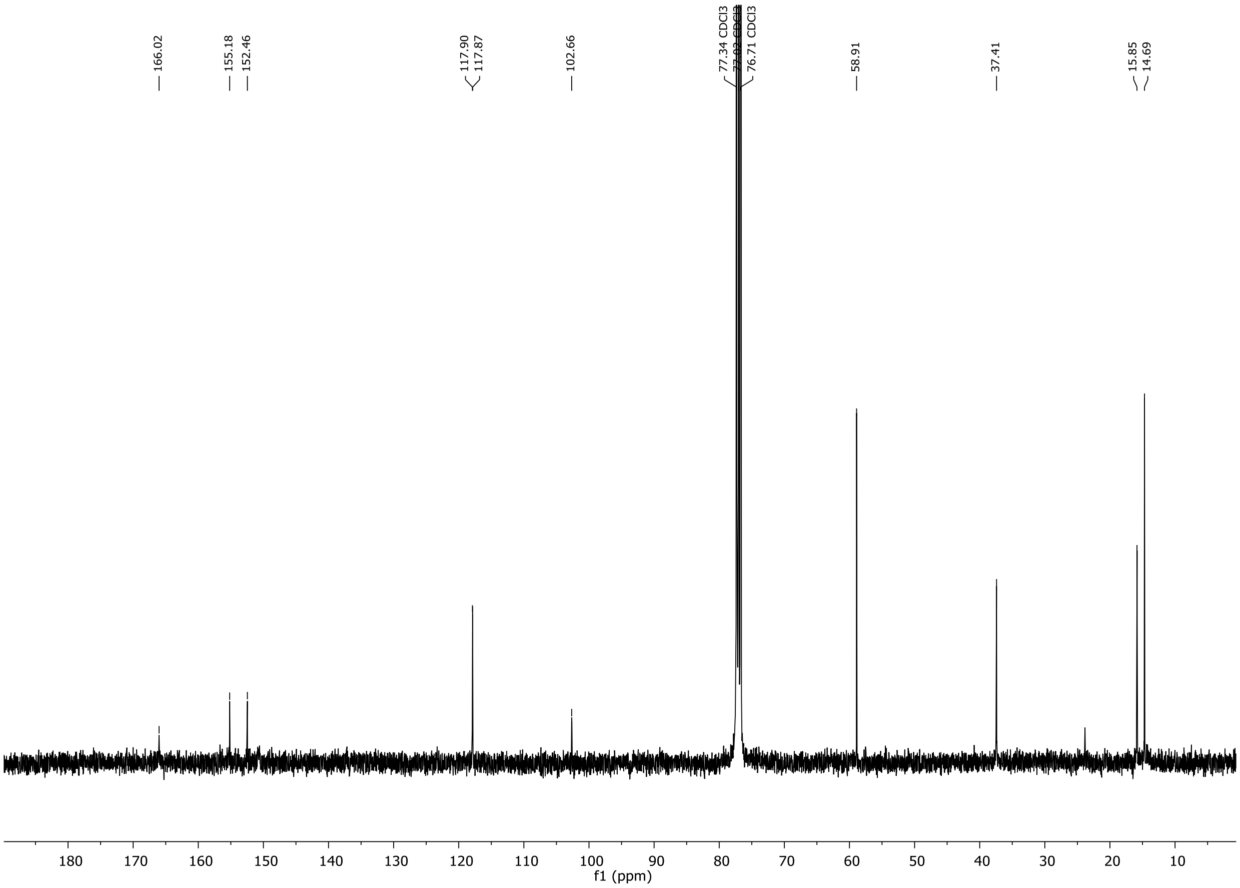

^{19}F NMR

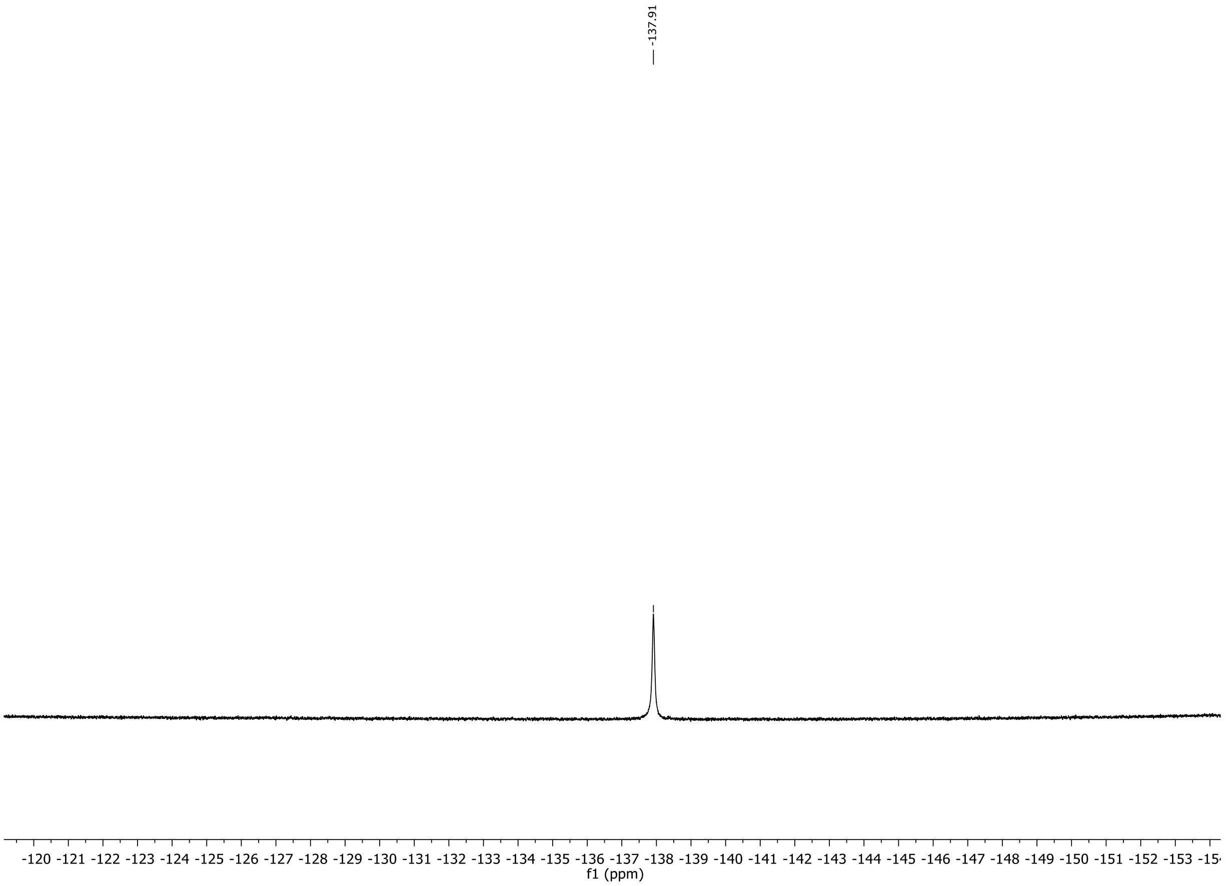

Ethyl (*S*)-2-amino-5-methyl-3-(methylthio)cyclopenta-1,3-diene-1-carboxylate (3r)

¹H NMR

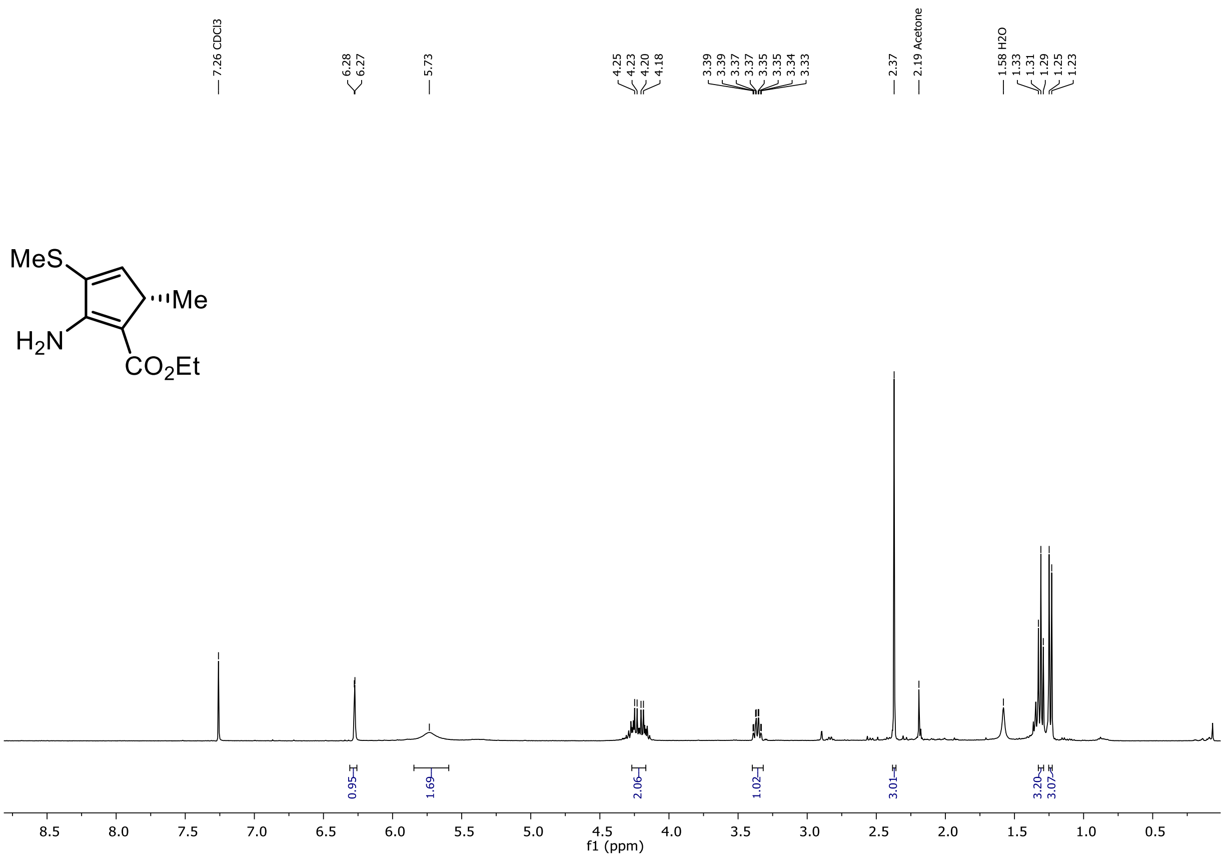

¹³C NMR

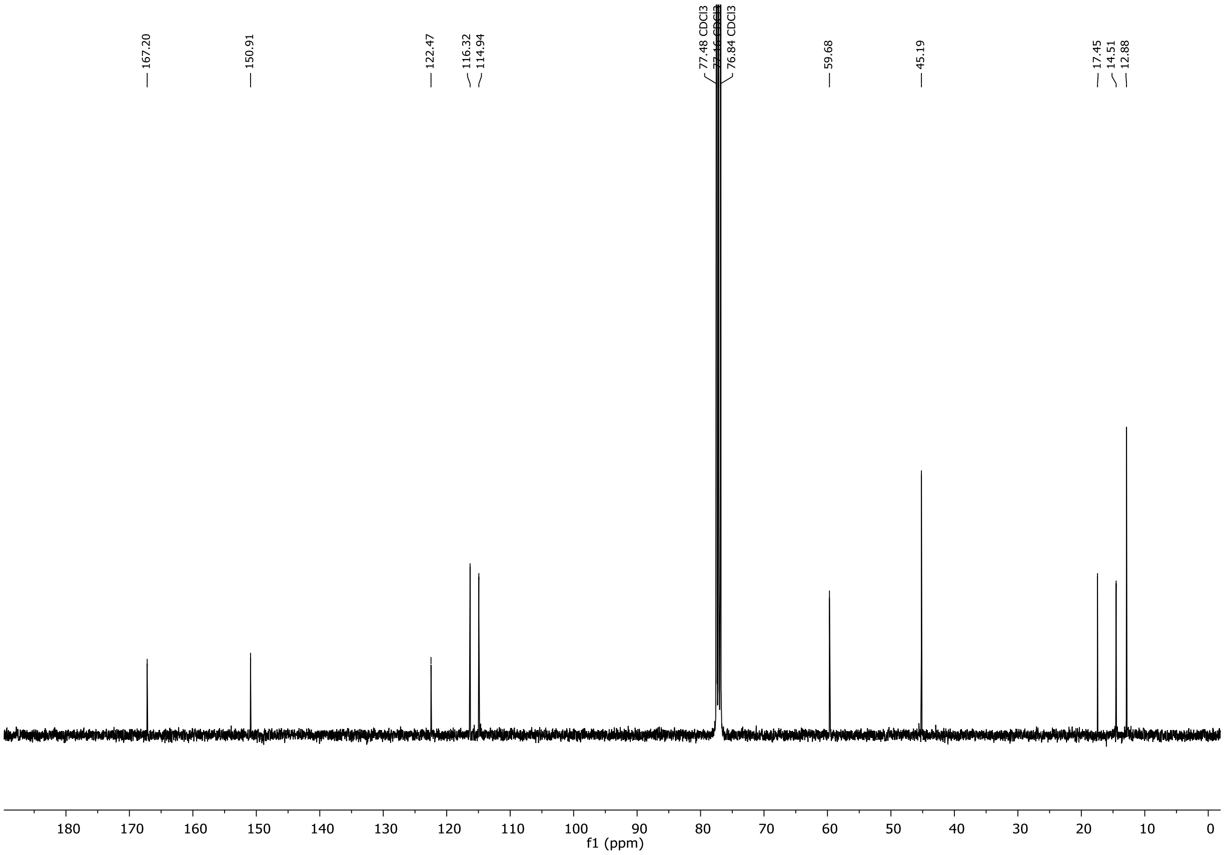

Methyl (*S*)-2-amino-3-benzyl-5-ethylcyclopenta-1,3-diene-1-carboxylate (3s)

^{1}H NMR

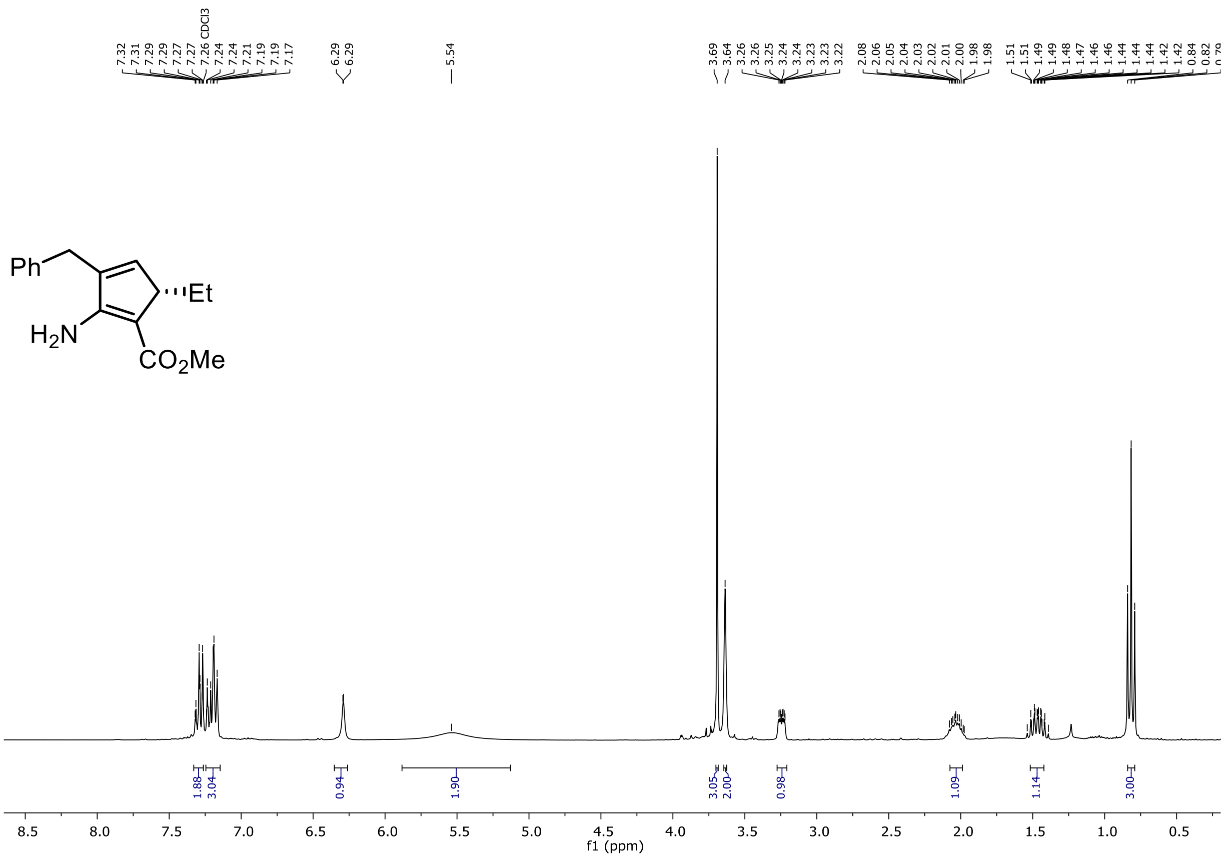

^{13}C NMR

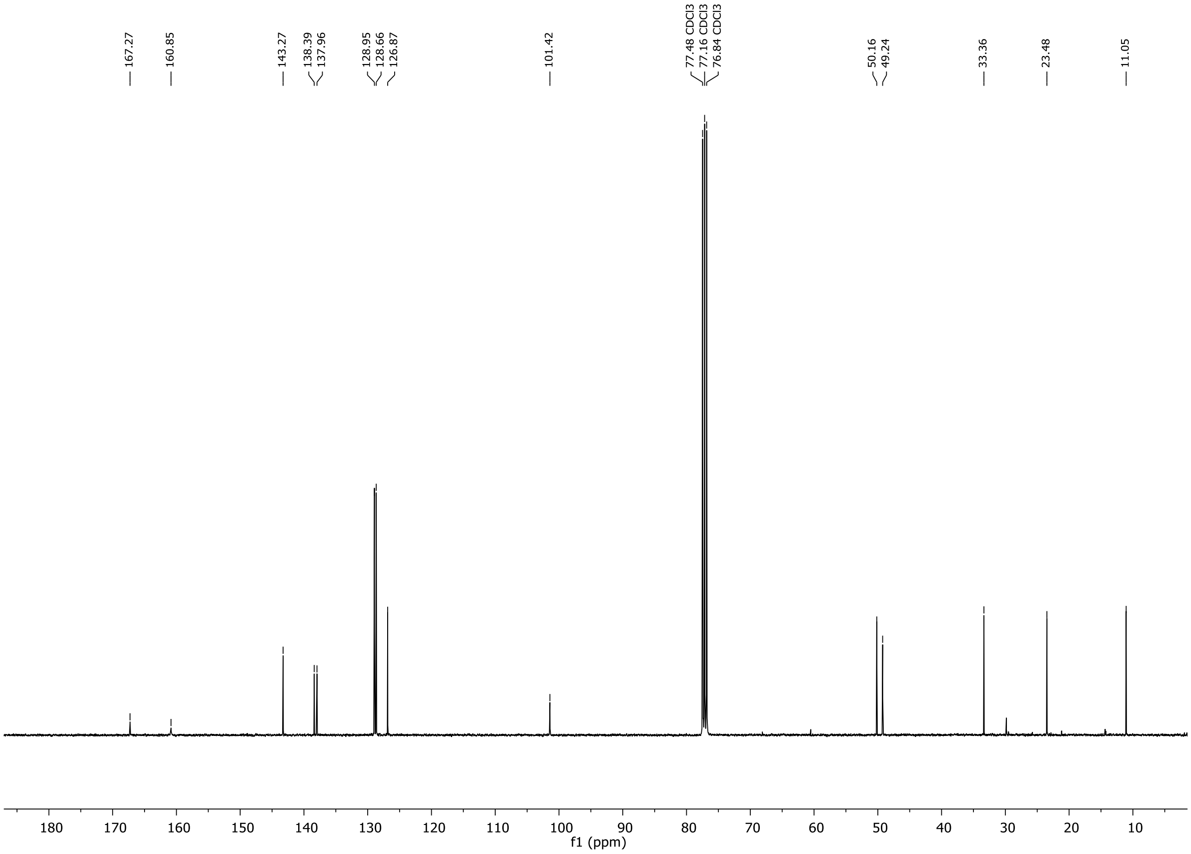

Methyl (*R*)-2-amino-3-benzyl-5-isopropylcyclopenta-1,3-diene-1-carboxylate (3t)

^{1}H NMR

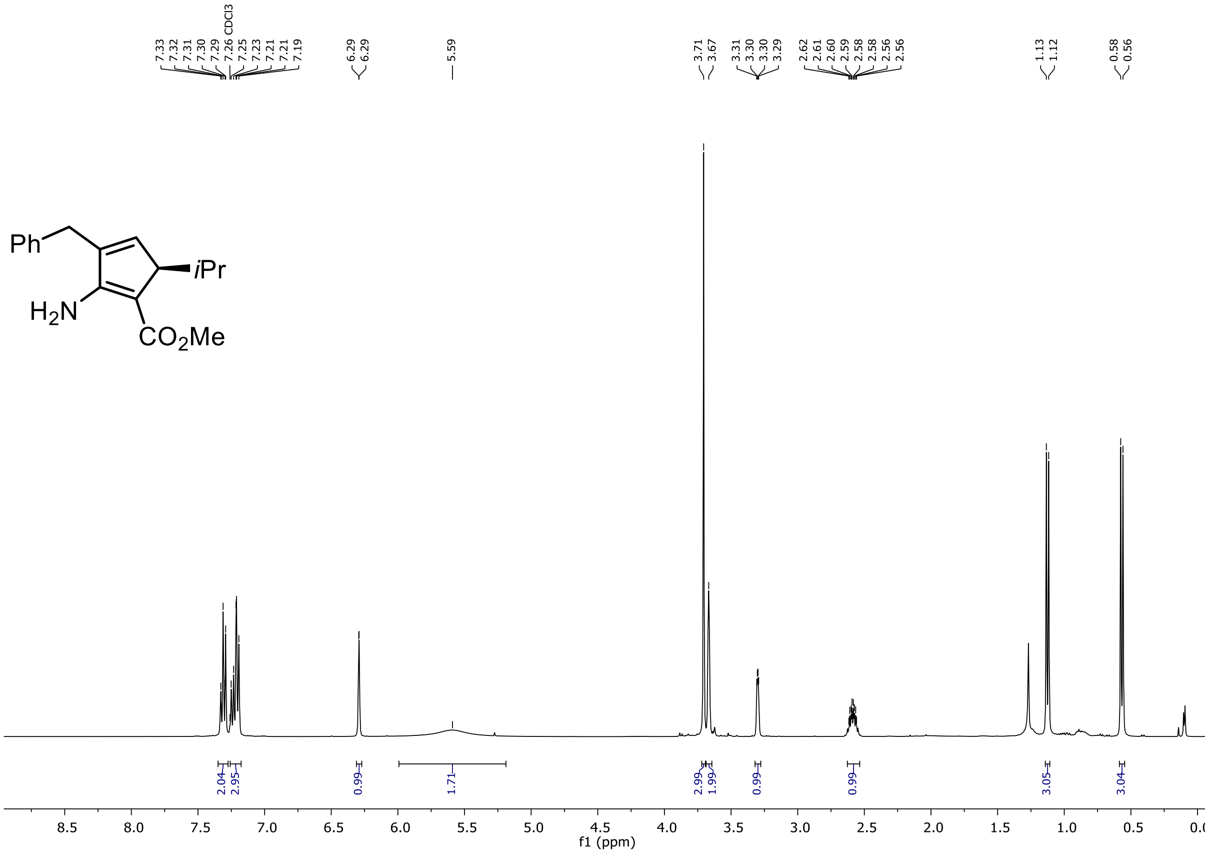

^{13}C NMR

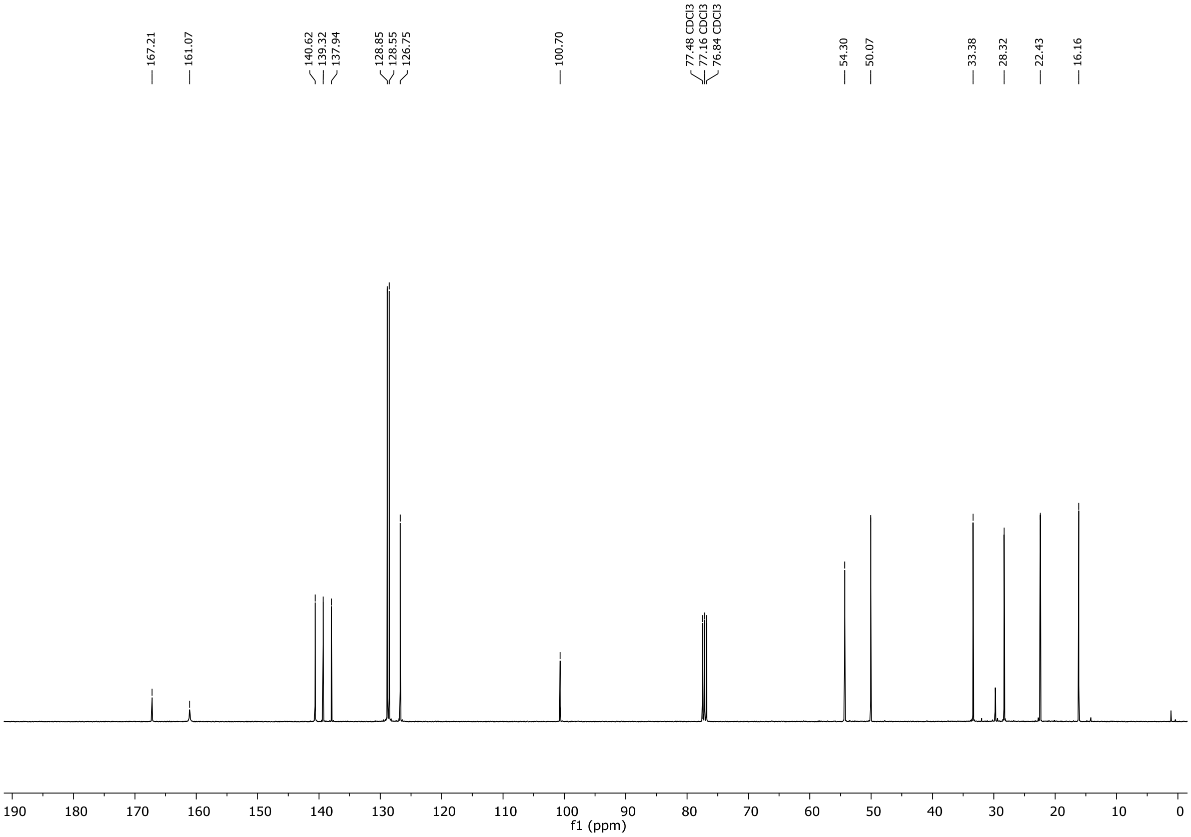

(*S*)-6-amino-5-benzyl-3,3a-dihydro-1H-cyclopenta[c]furan-1-one (3u)

^{1}H NMR

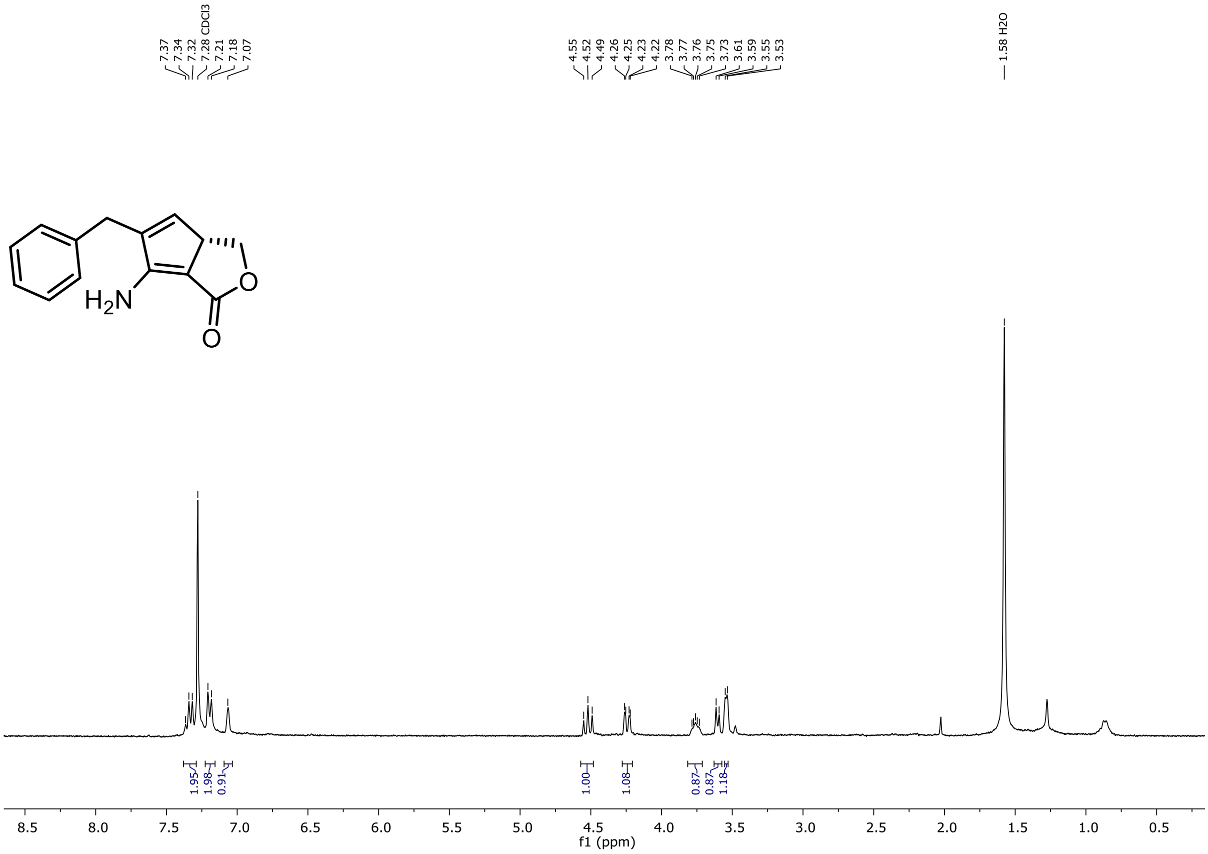

^{13}C NMR

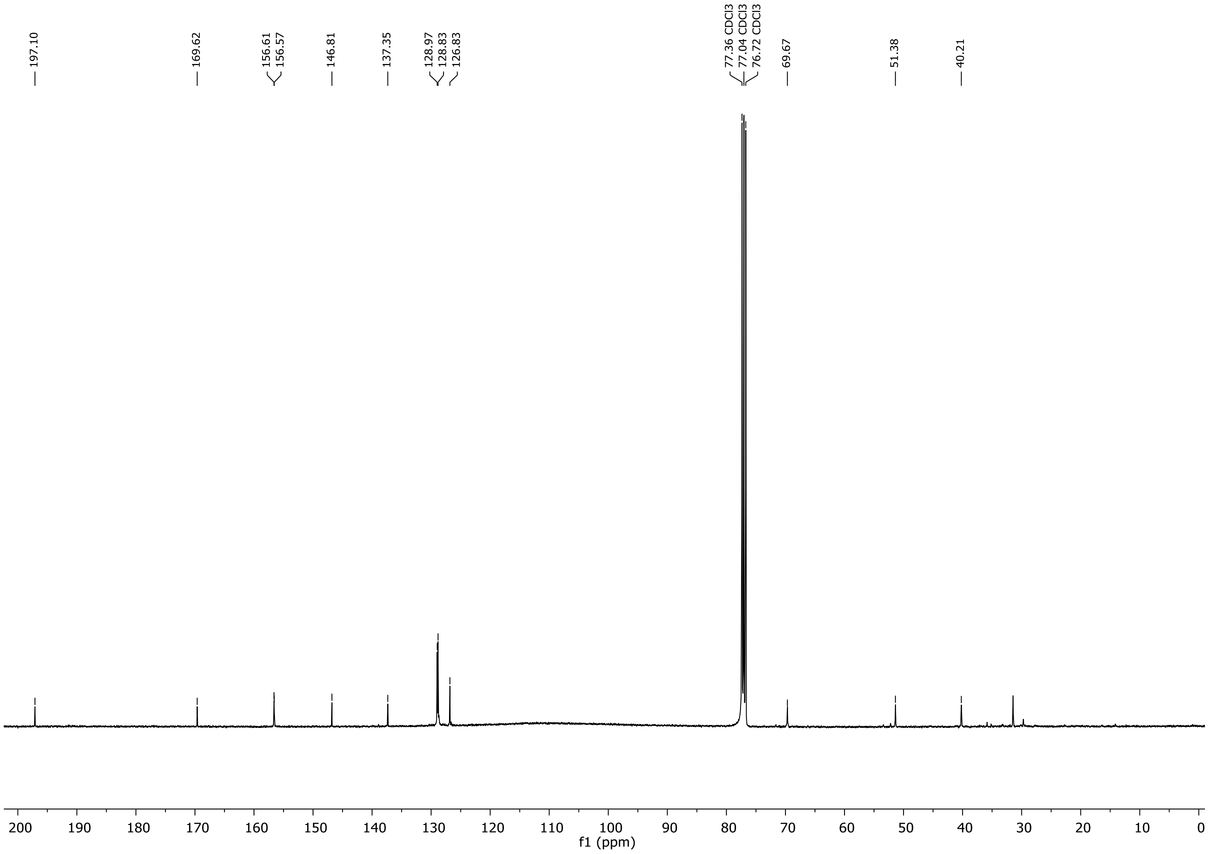

Ethyl (*R*)-2-amino-5-(but-3-en-1-yl)-3-methylcyclopenta-1,3-diene-1-carboxylate (3v)

^{1}H NMR

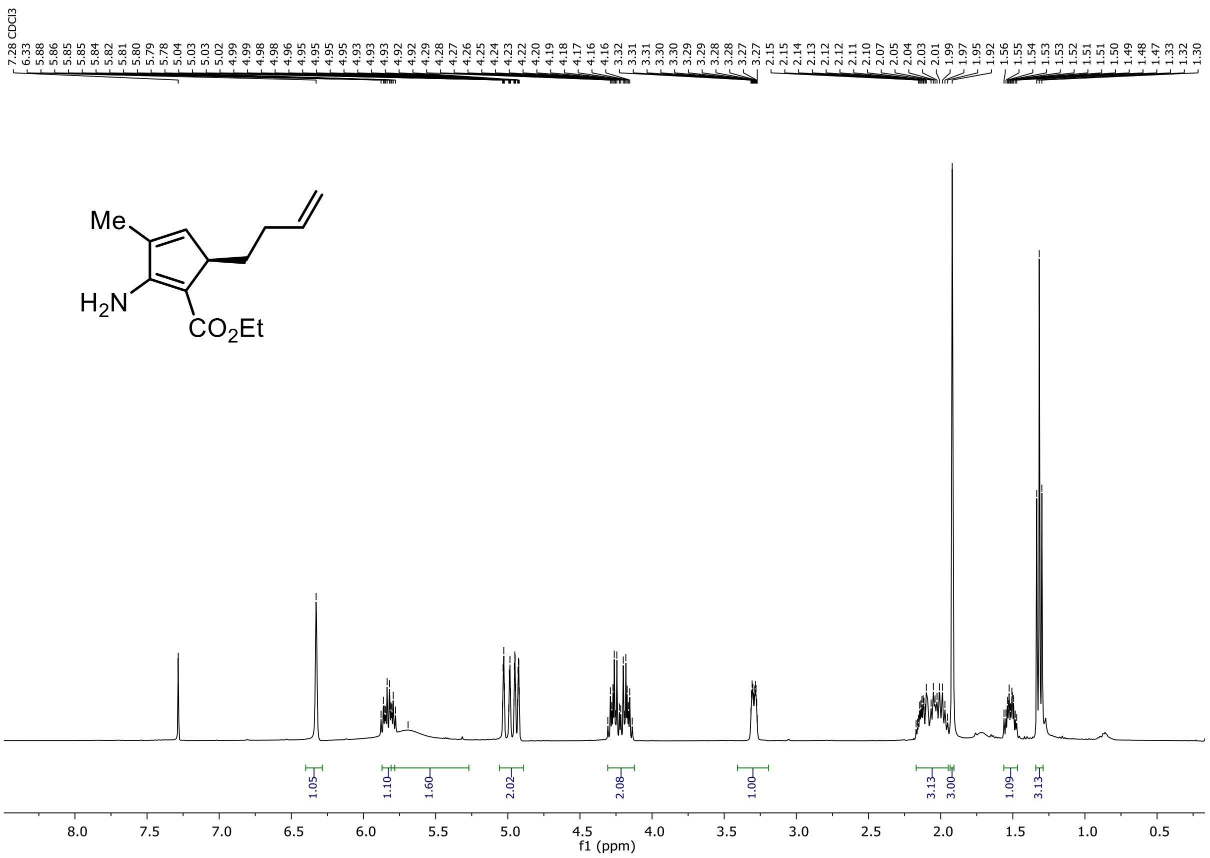

^{13}C NMR

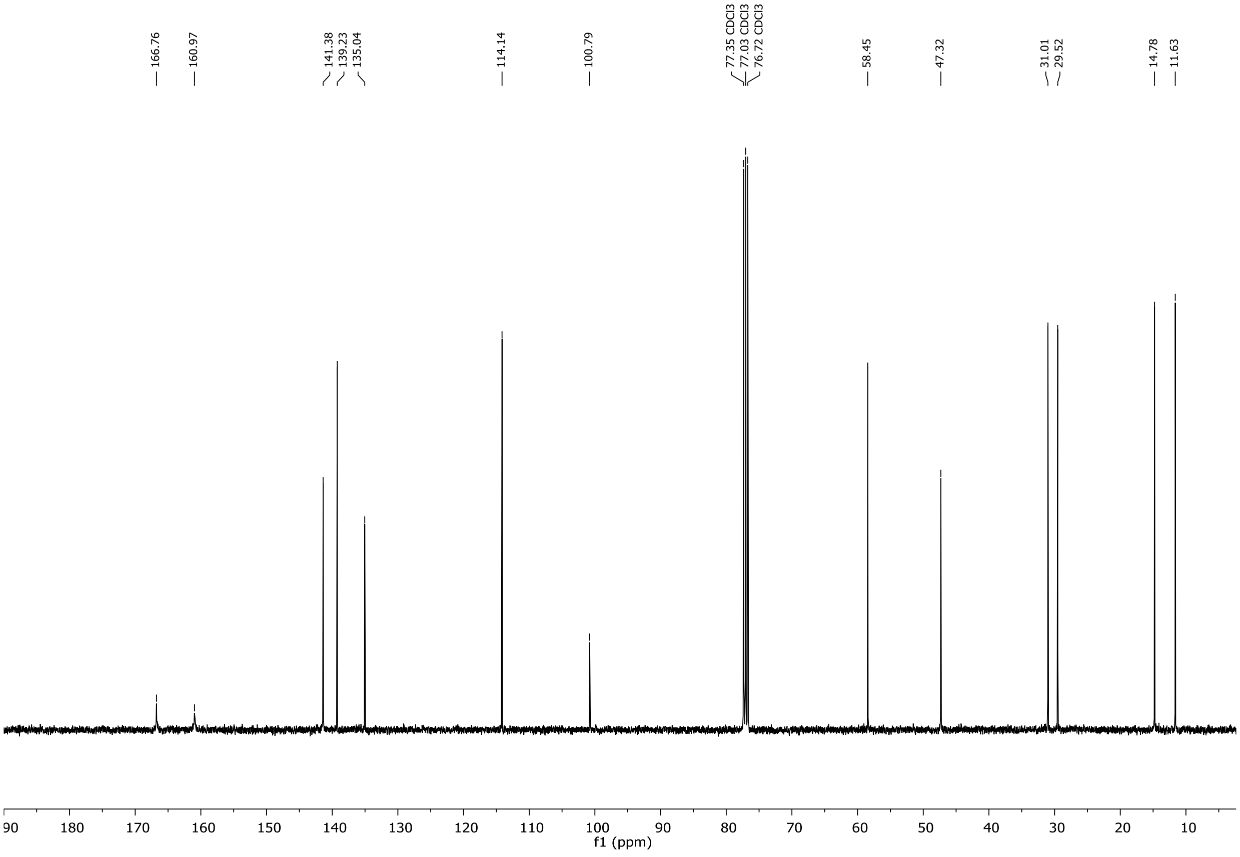

Ethyl (*R*)-5-(2-(1,3-dioxolan-2-yl)ethyl)-2-amino-3-benzylcyclopenta-1,3-diene-1-carboxylate (3w)

^{1}H NMR

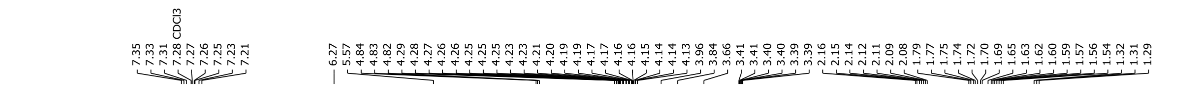

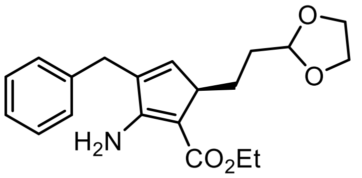

^{13}C NMR

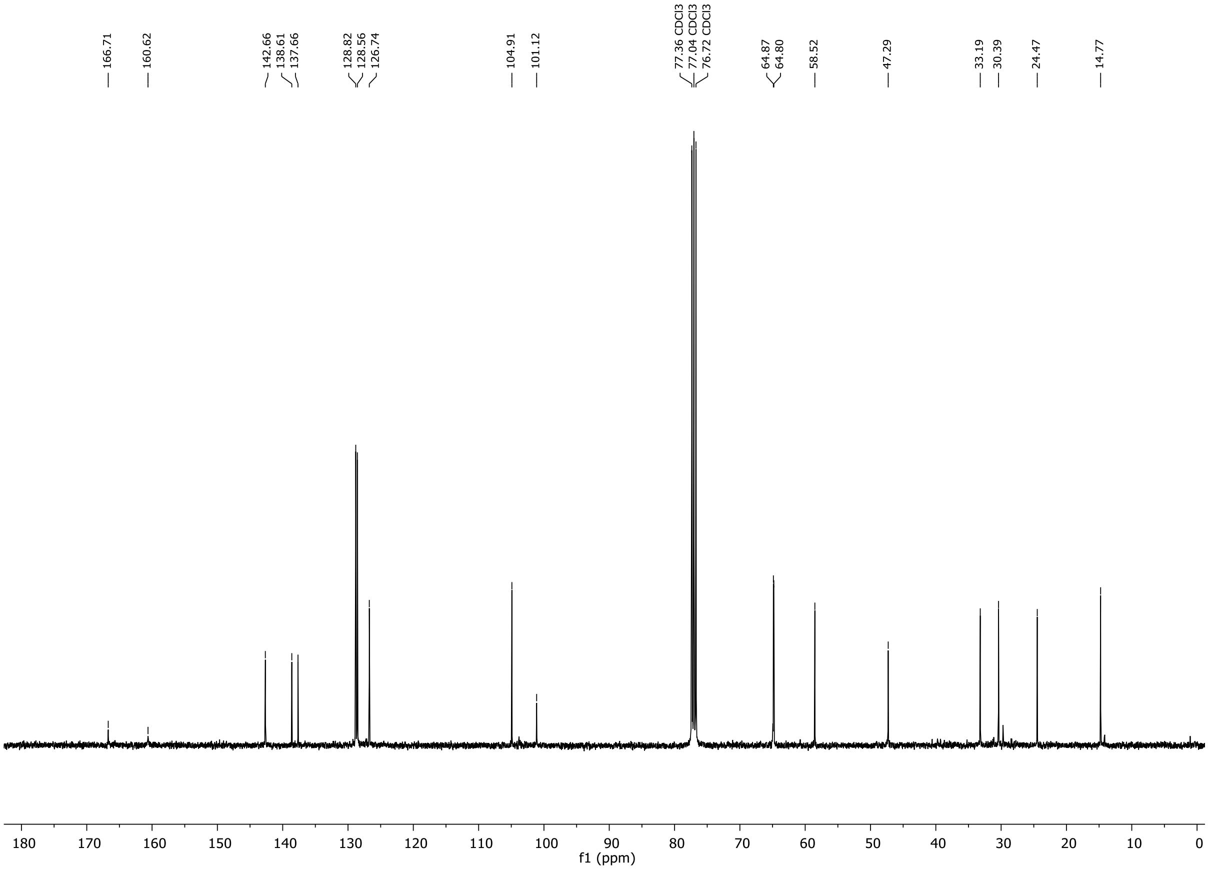

Ethyl (*S*)-2-amino-3-benzyl-5-phenylcyclopenta-1,3-diene-1-carboxylate (3x)

^{1}H NMR

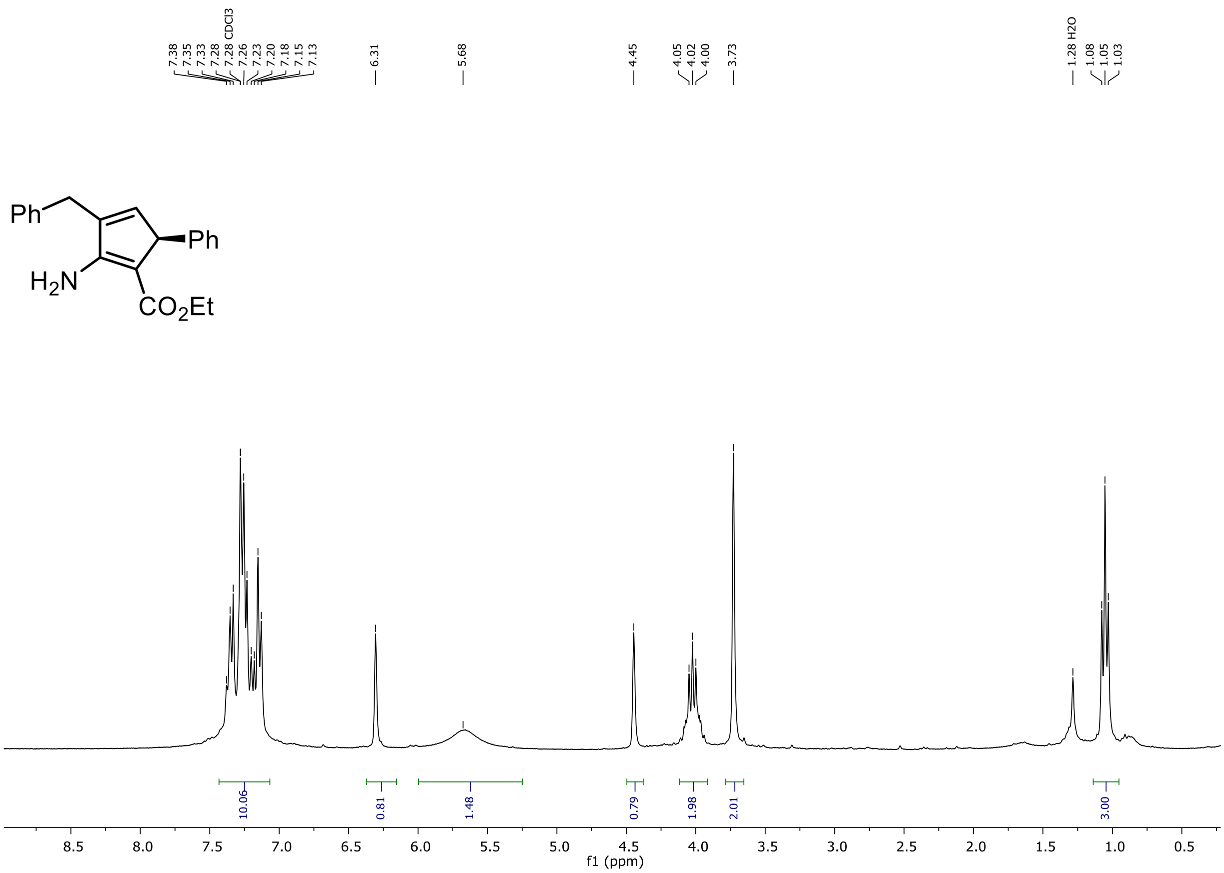

^{13}C NMR

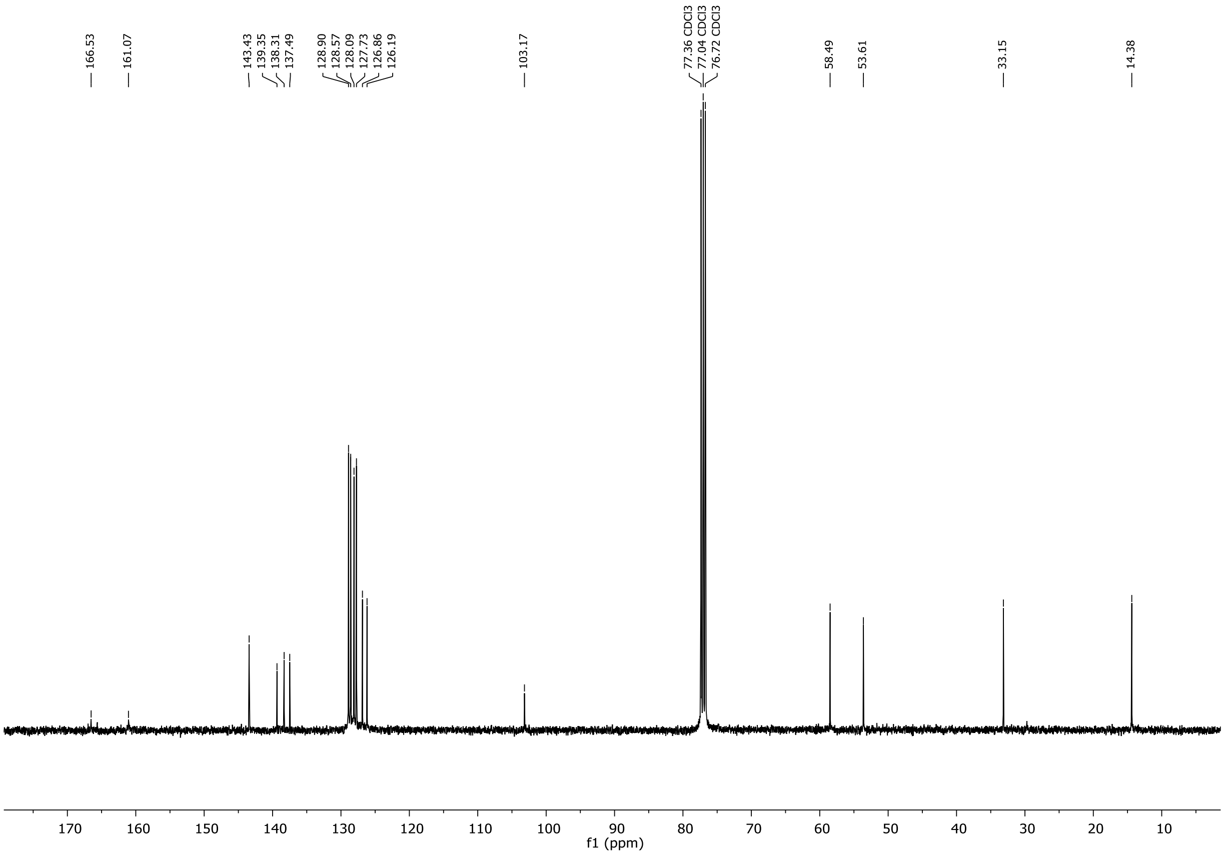

Ethyl (S)-2-amino-3-benzyl-5-(naphthalen-2-yl)cyclopenta-1,3-diene-1-carboxylate (3y)

^{1}H NMR

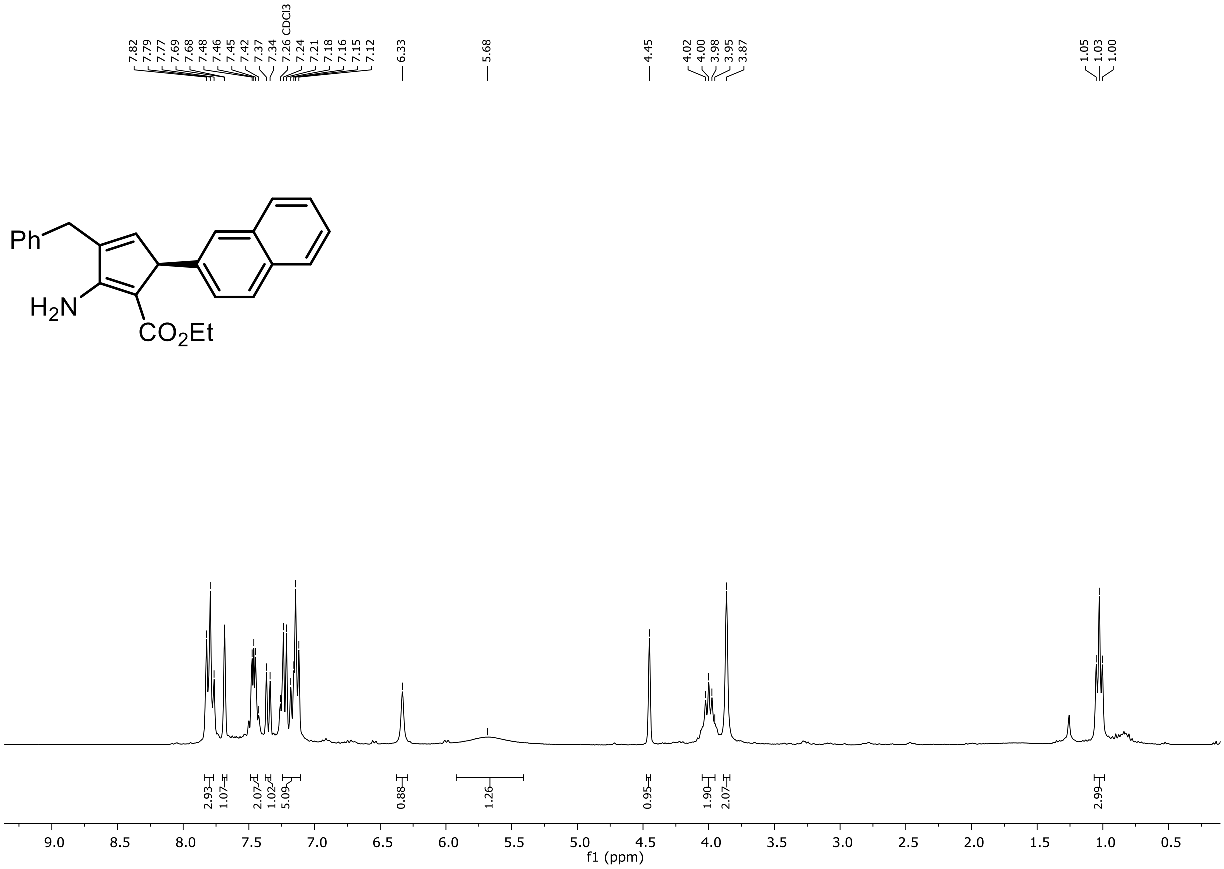

^{13}C NMR

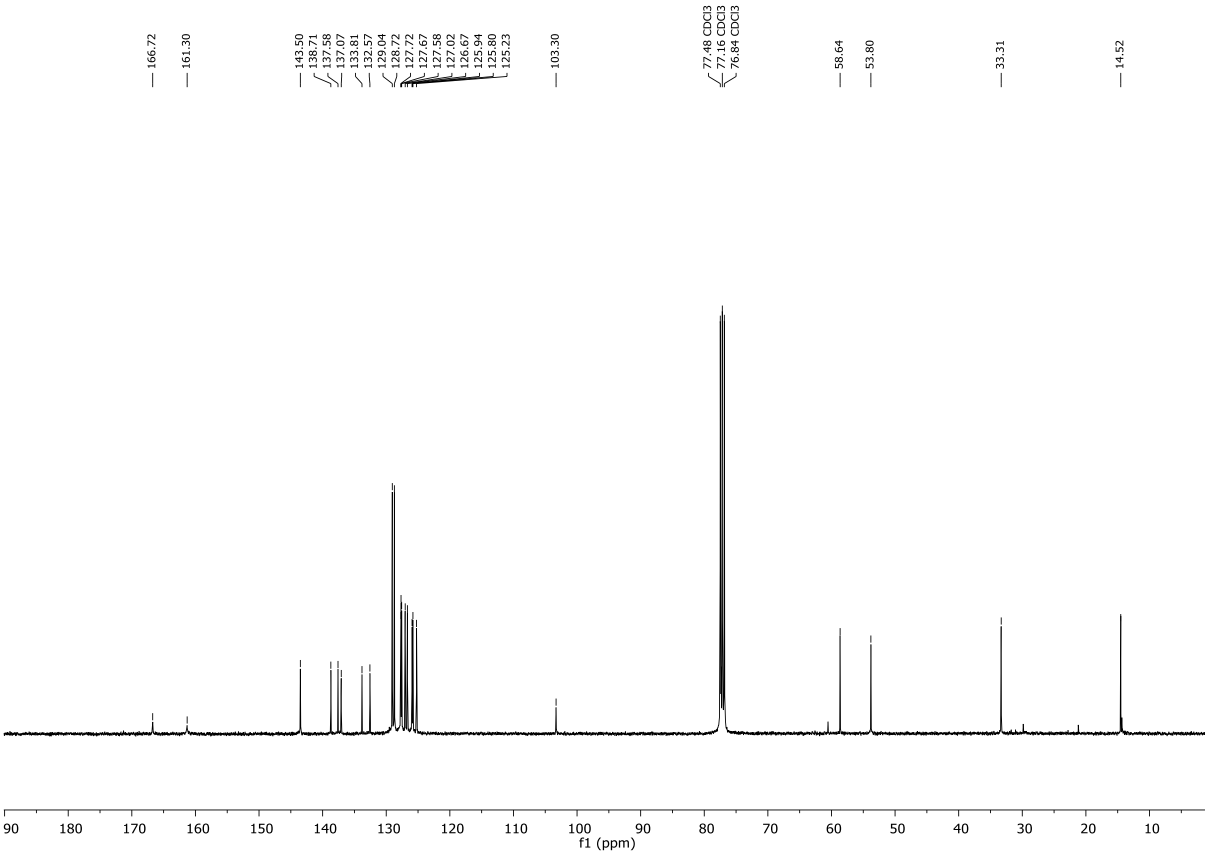

Ethyl (*S*)-2-amino-3-benzyl-5-(pyridin-4-yl)cyclopenta-1,3-diene-1-carboxylate (3z)

^{1}H NMR

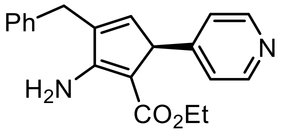

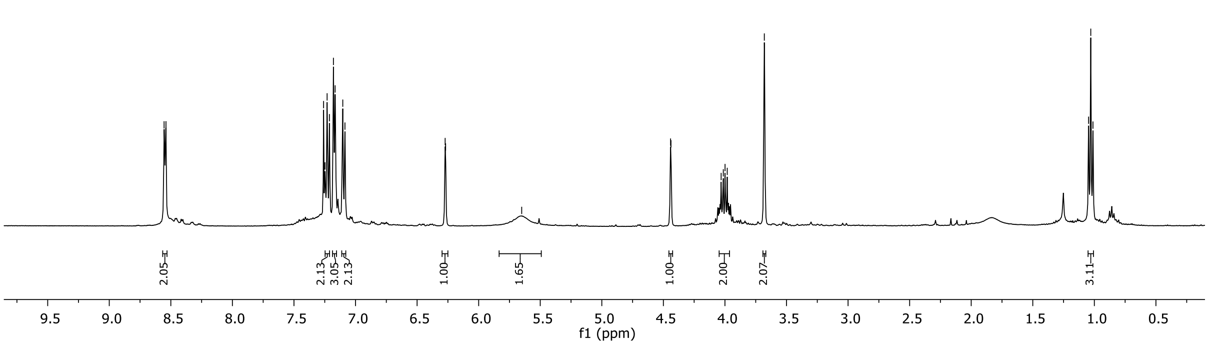

^{13}C NMR

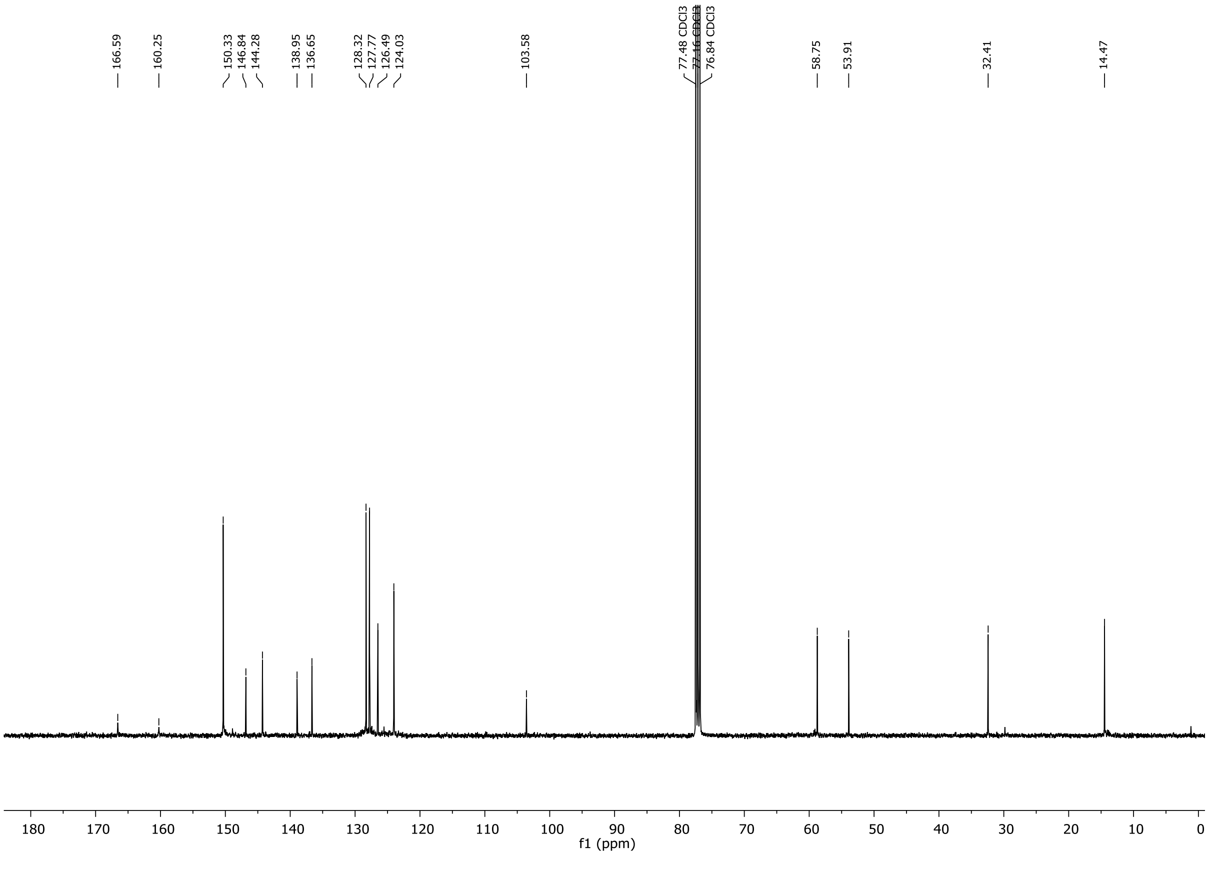

Ethyl (S)-2-amino-3-fluoro-4,5-dimethylcyclopenta-1,3-diene-1-carboxylate (3aa)

¹H NMR

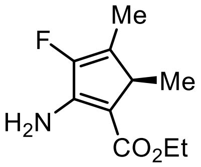

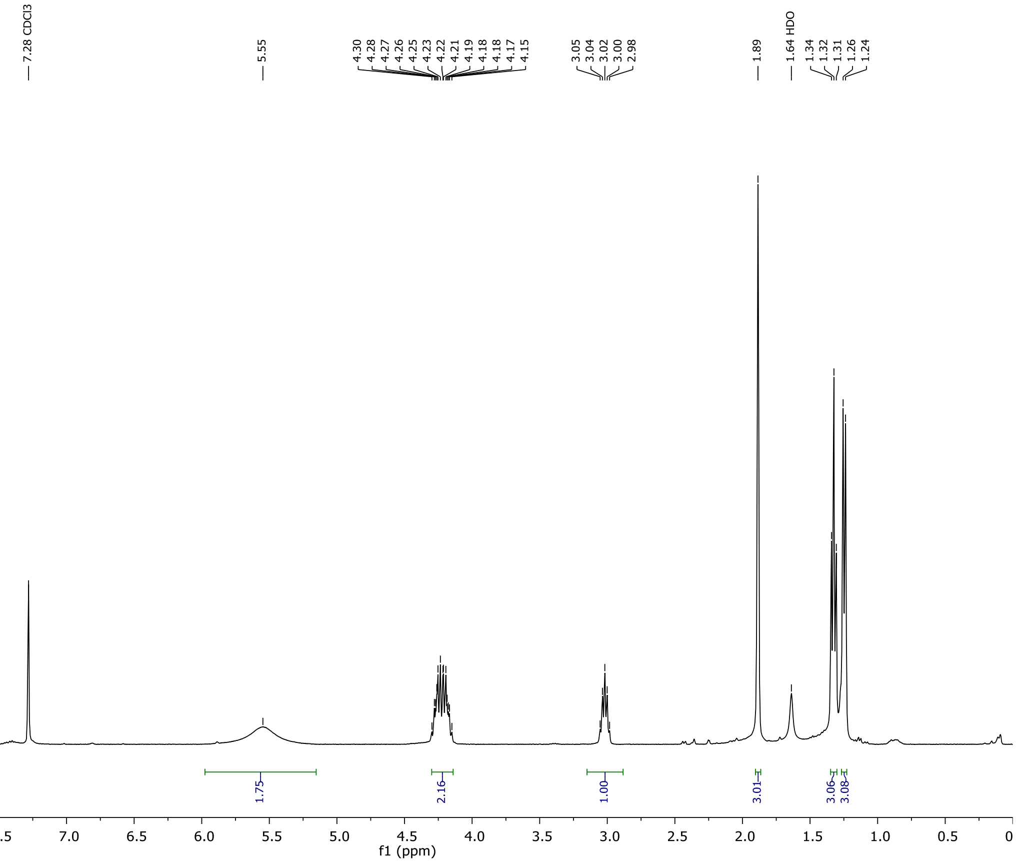

¹³C NMR

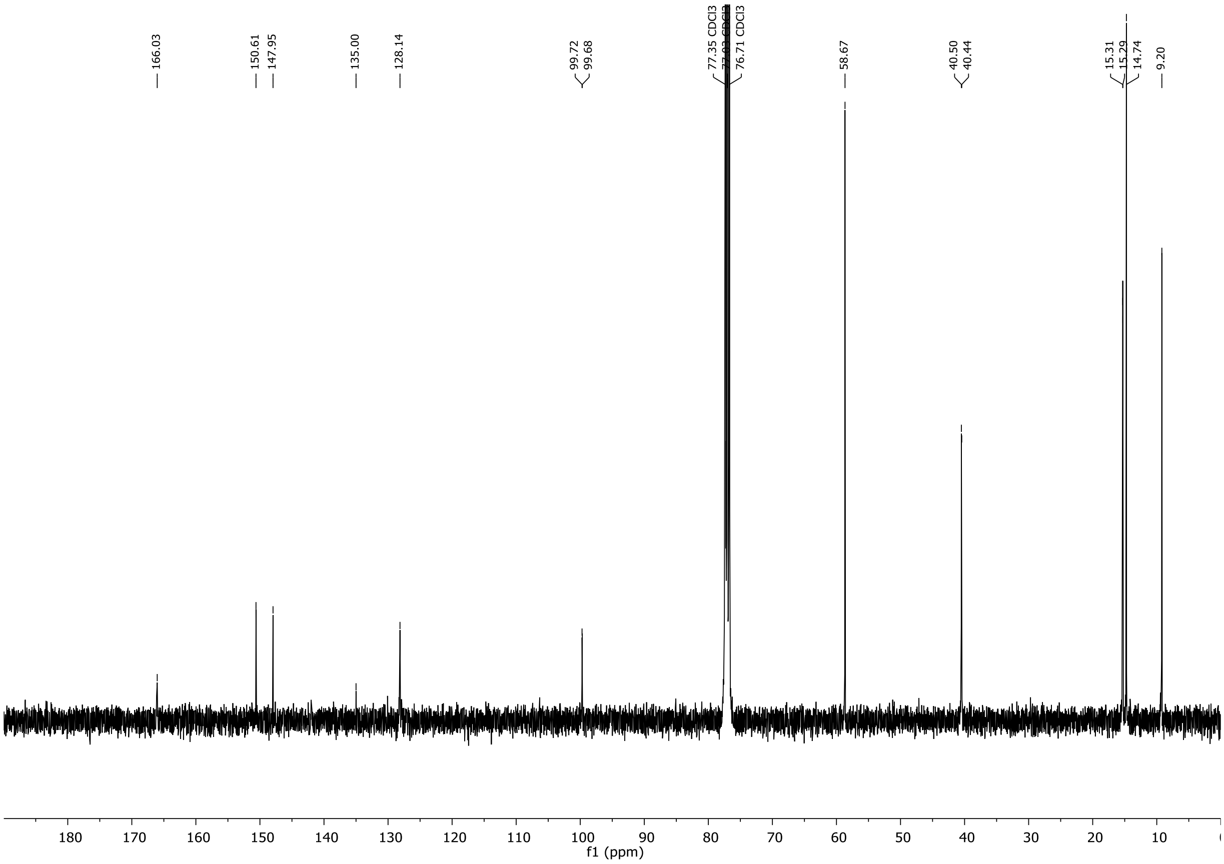

¹⁹F NMR

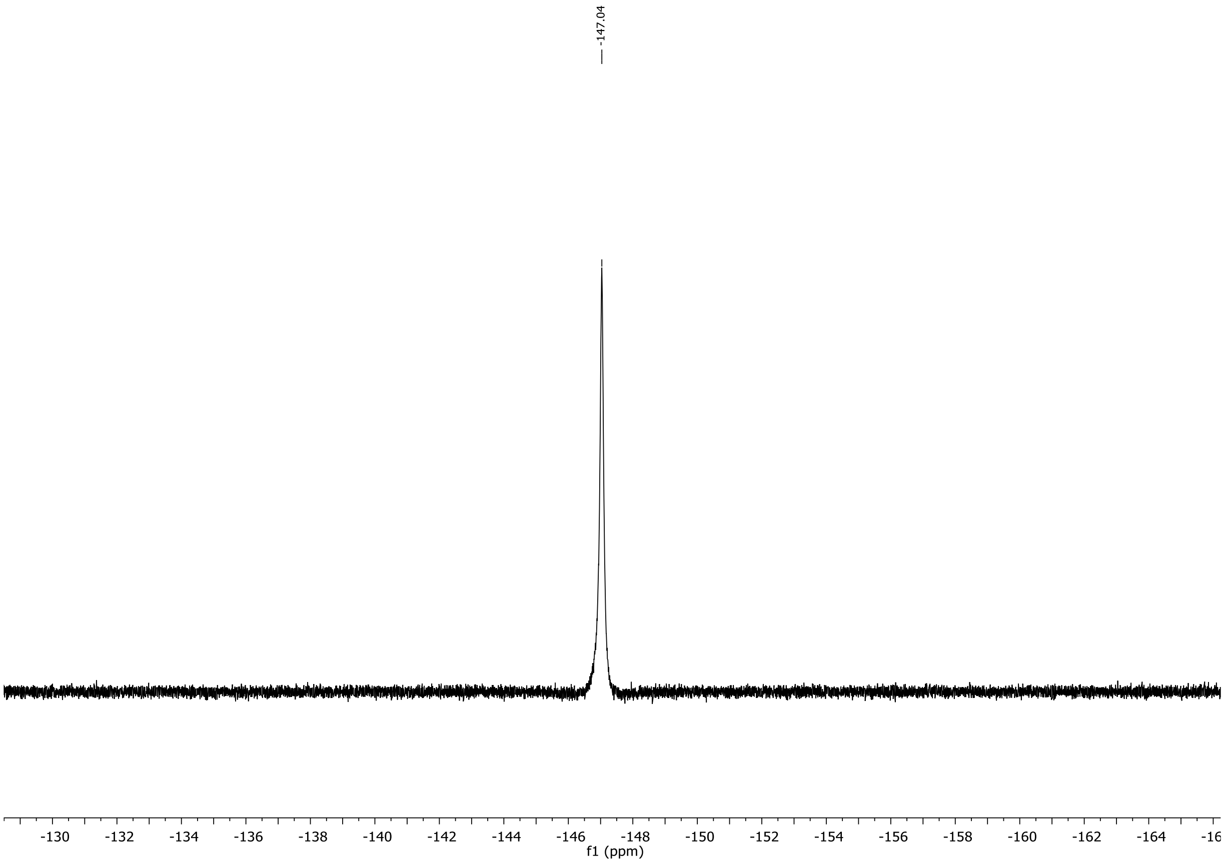

Ethyl (1*S*,5*R*)-3-benzyl-1-fluoro-5-methyl-2-oxocyclopent-3-ene-1-carboxylate (4)

^{1}H NMR (*dr*>20:1)

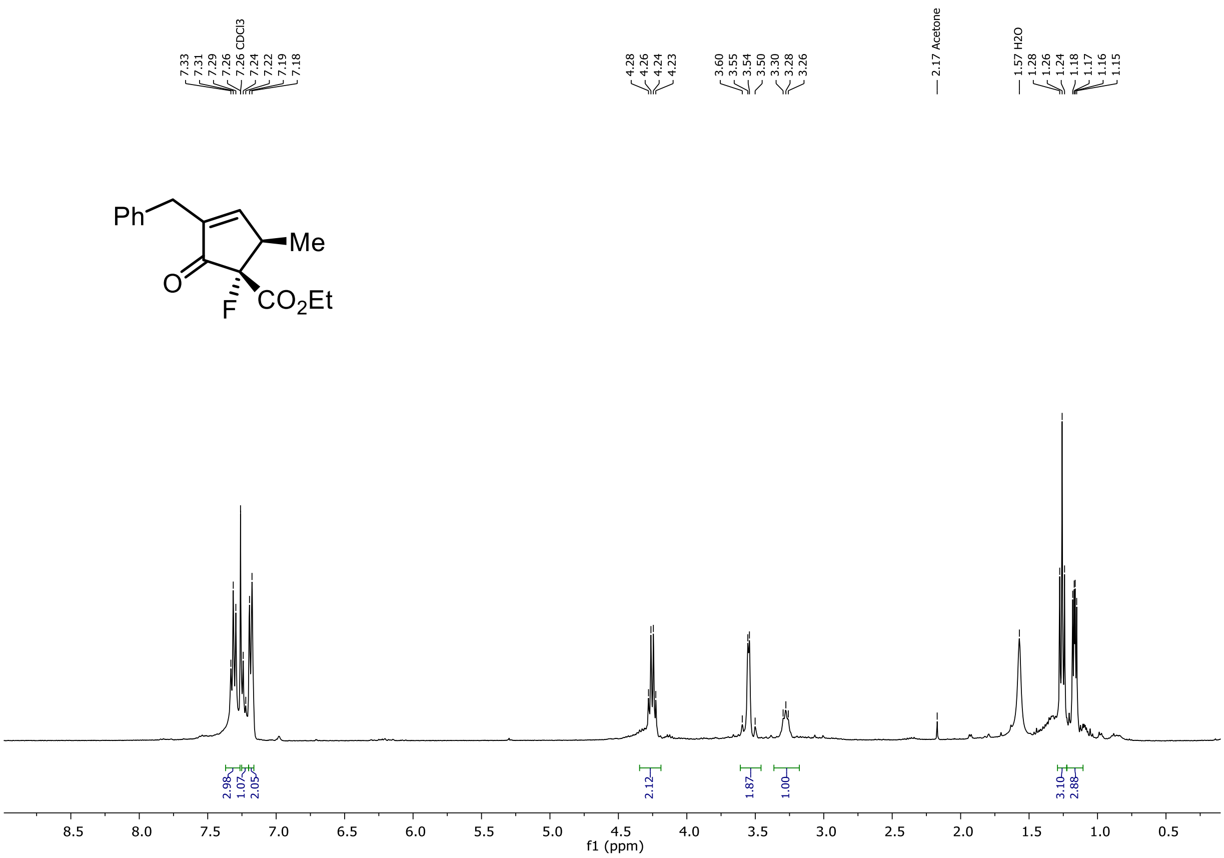

^{13}C NMR

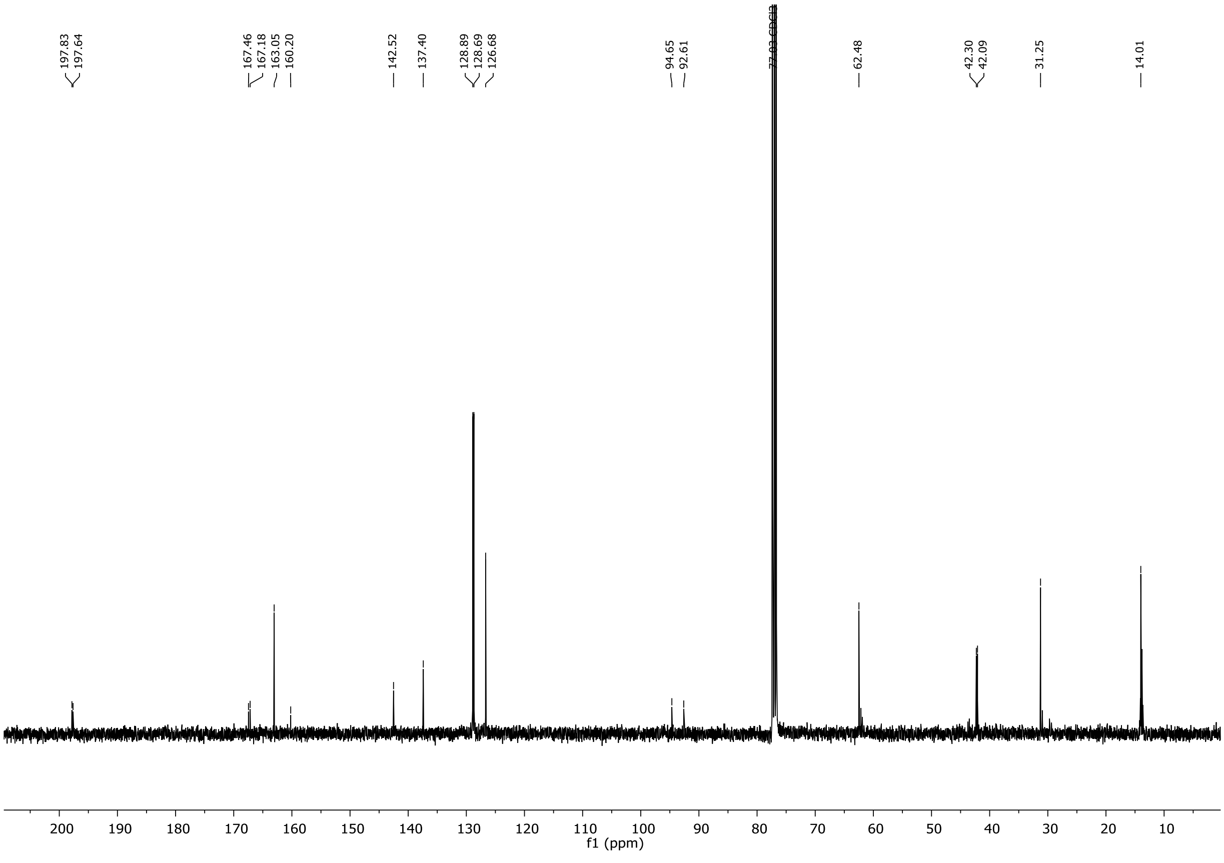

^{19}F NMR

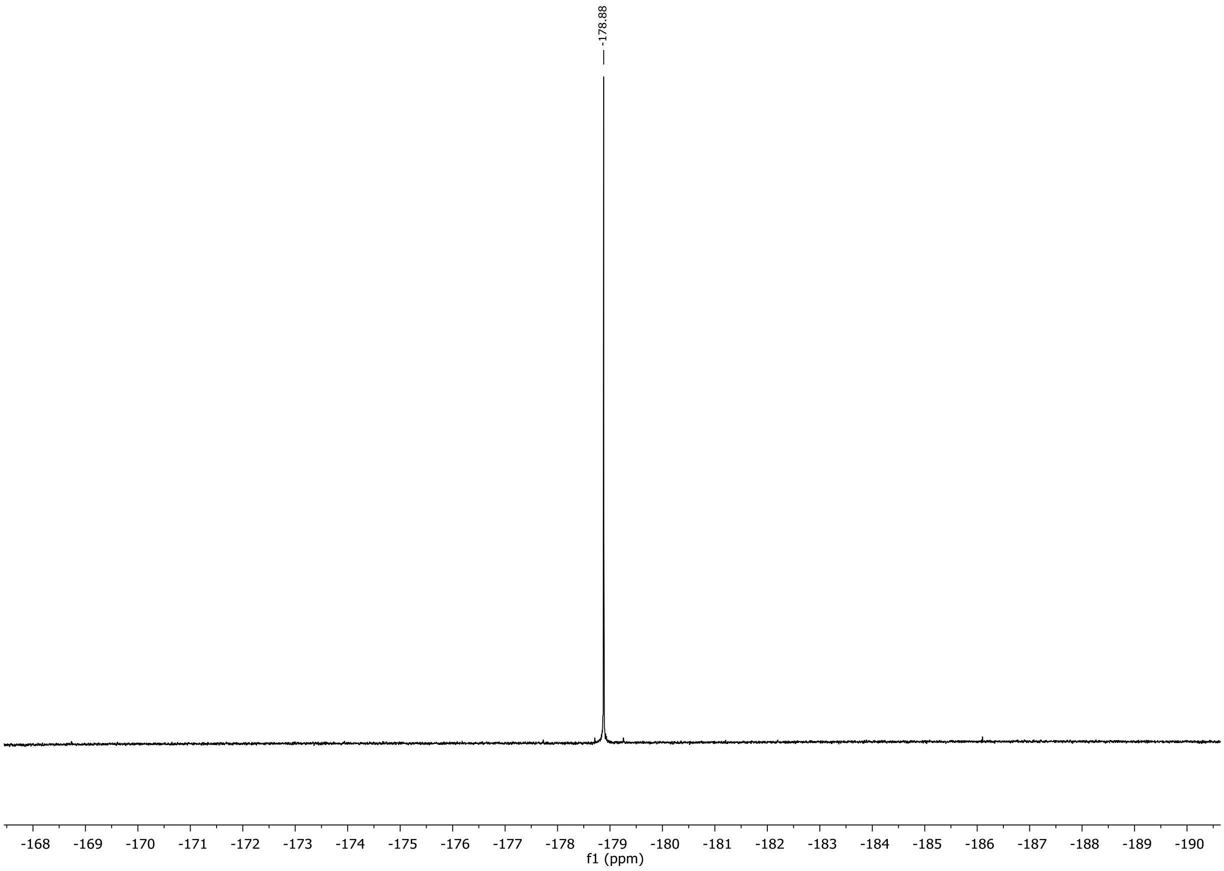

NOESY (400 MHz, Chloroform-*d*)

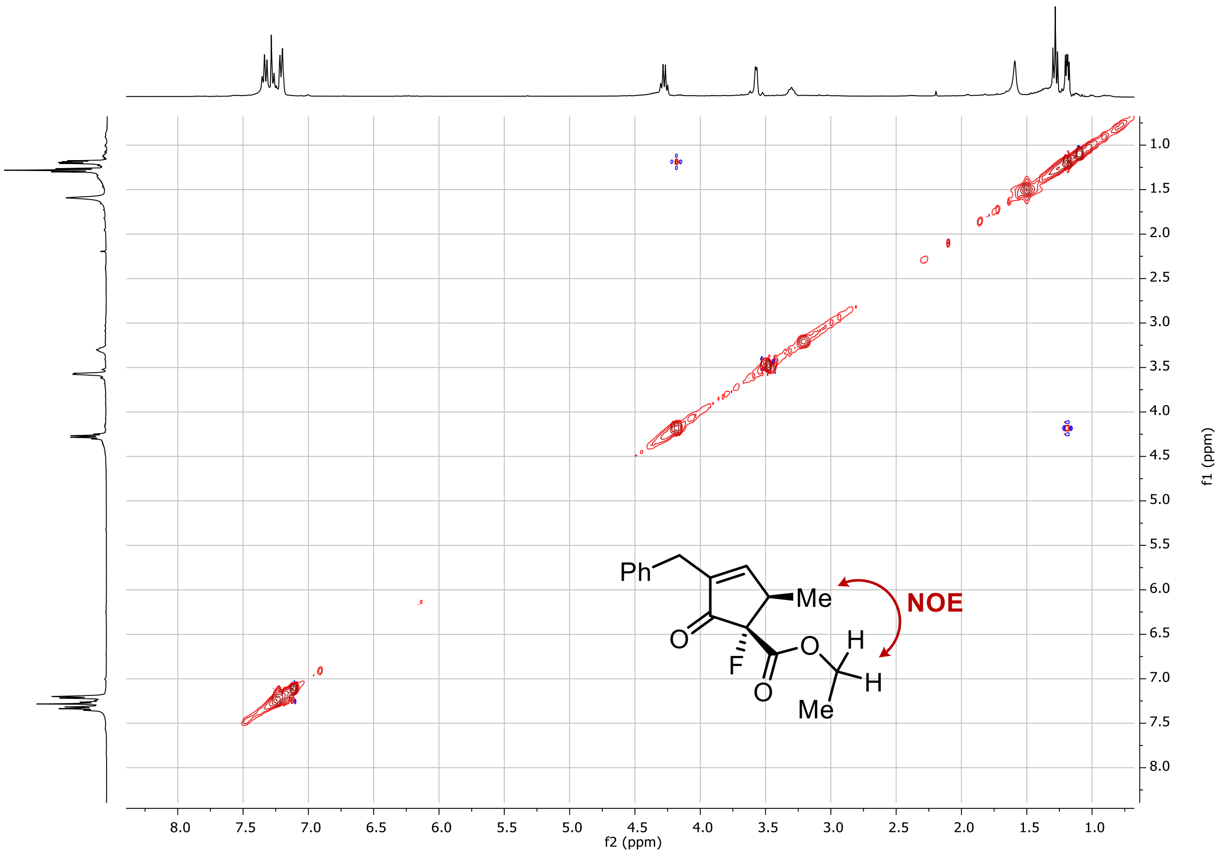

Ethyl (1*S*,5*R*)-3-benzyl-1-hydroxy-5-methyl-2-oxocyclopent-3-ene-1-carboxylate (5)

^{1}H NMR

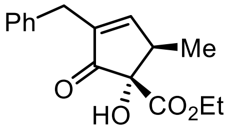

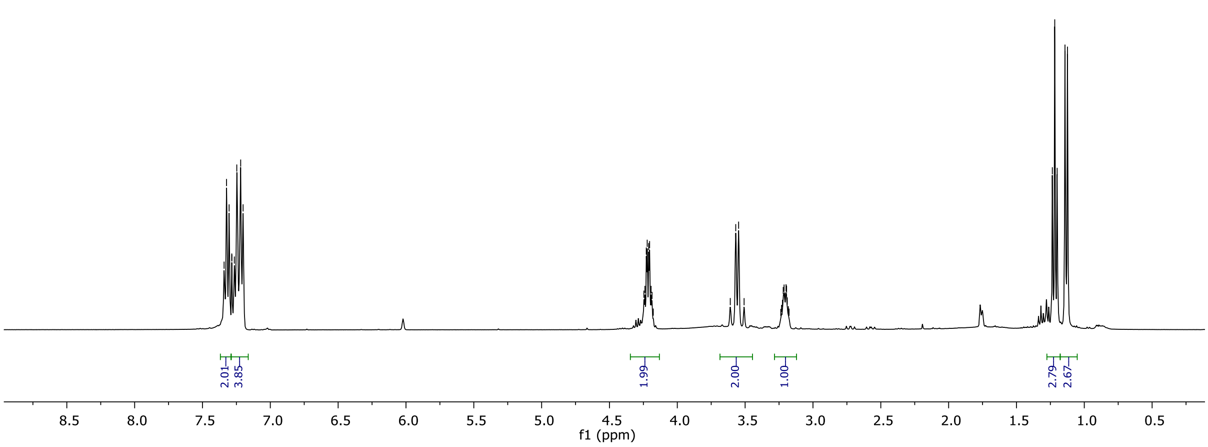

minor diast

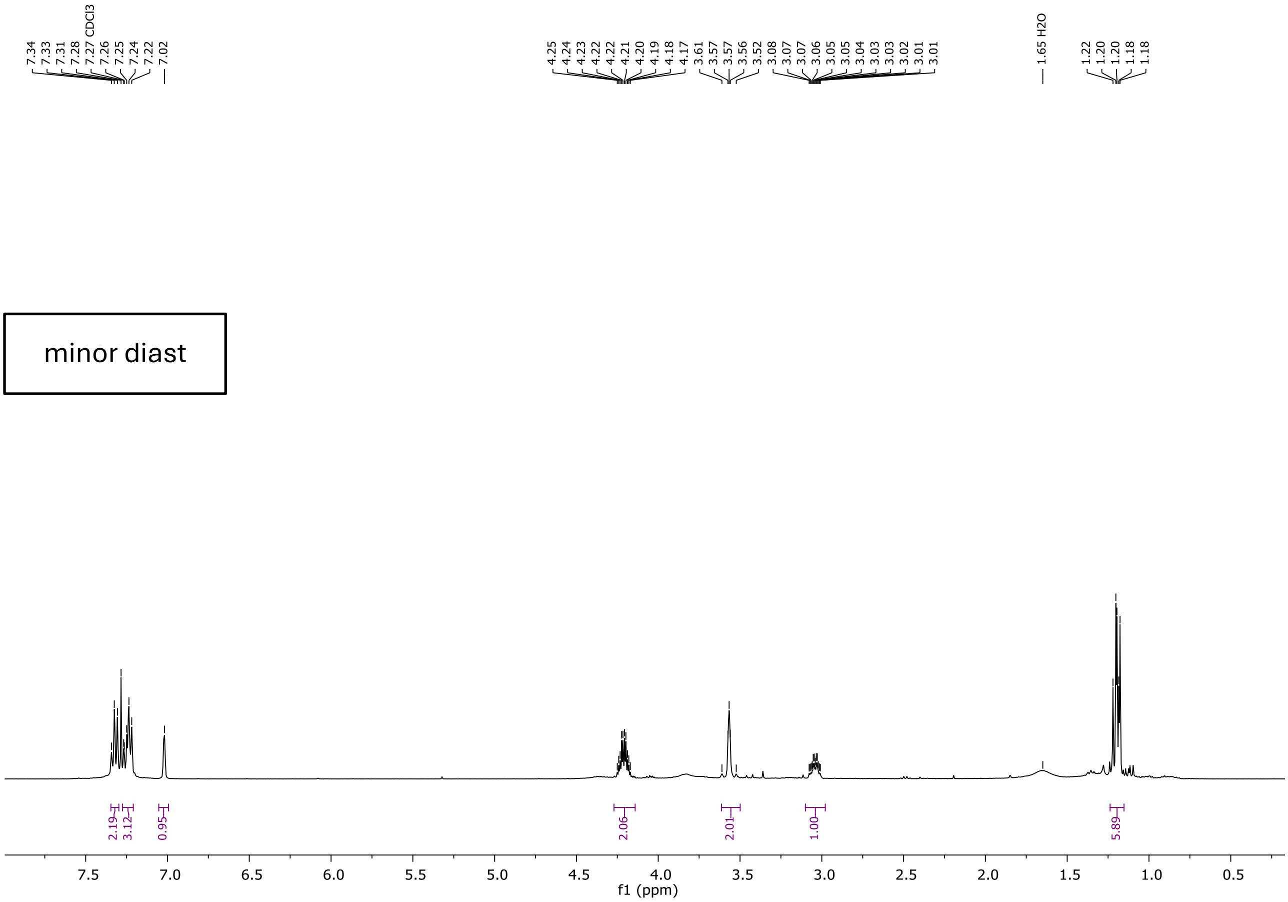

^{13}C NMR

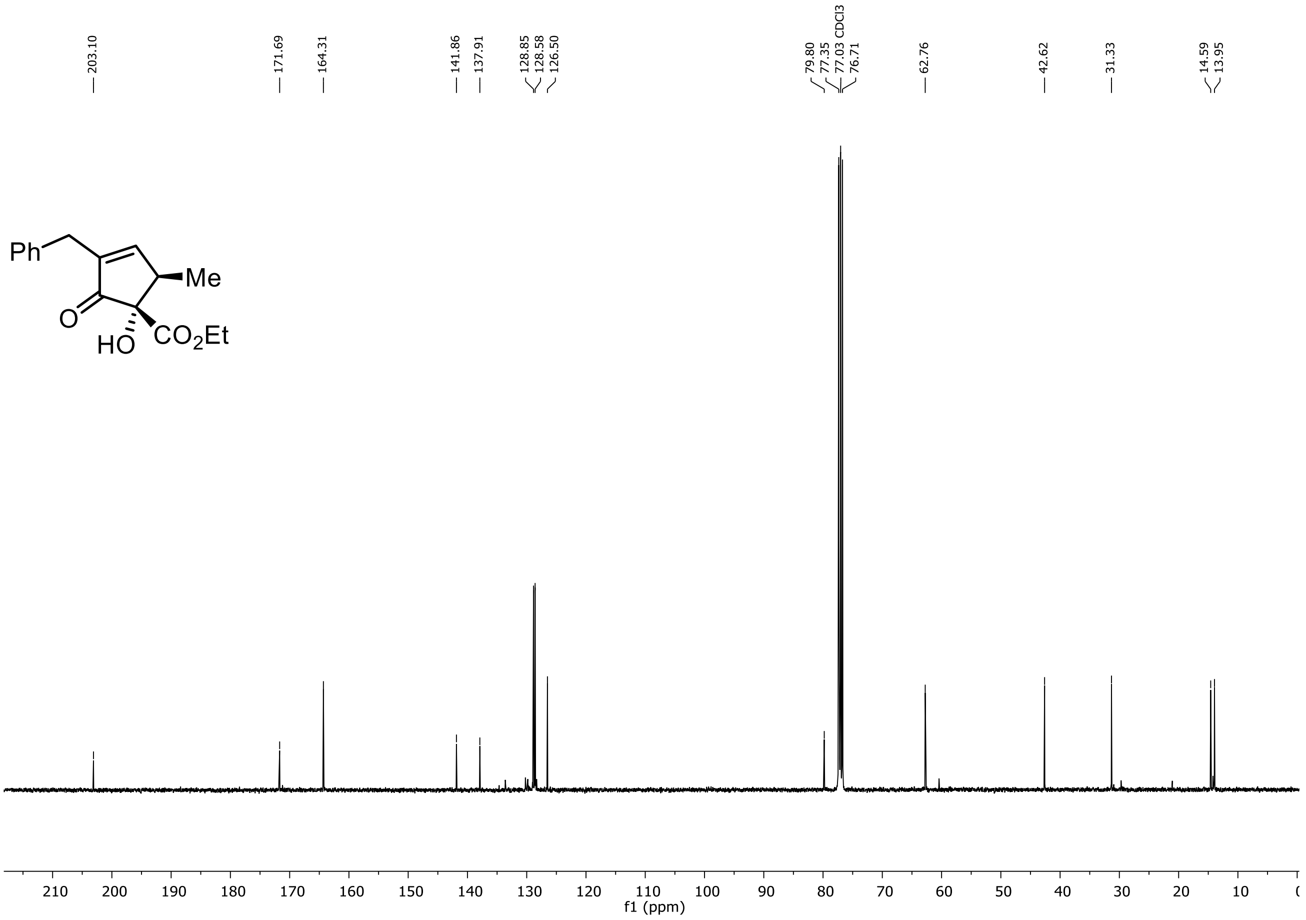

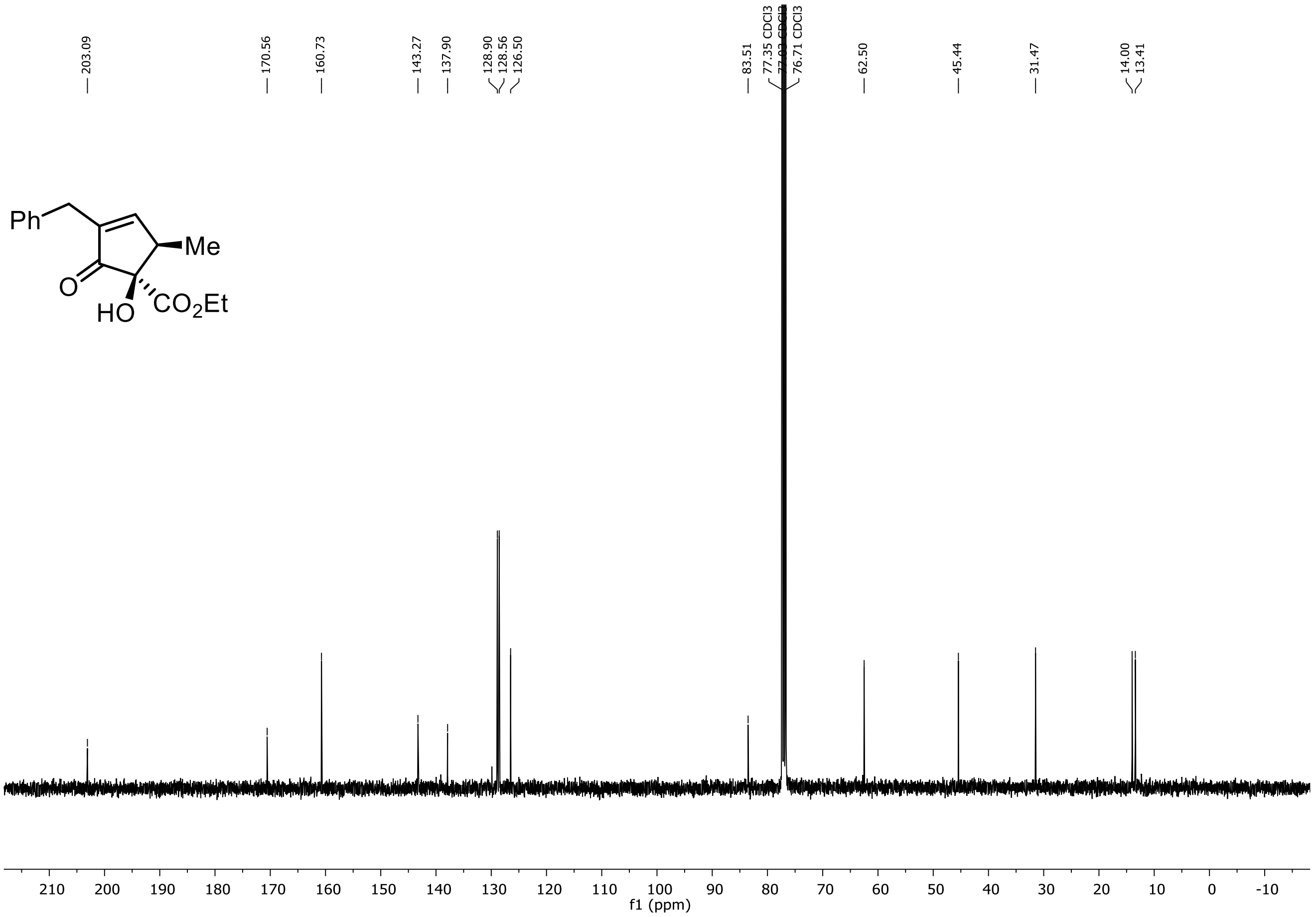

NOESY (400 MHz, Chloroform-*d*)

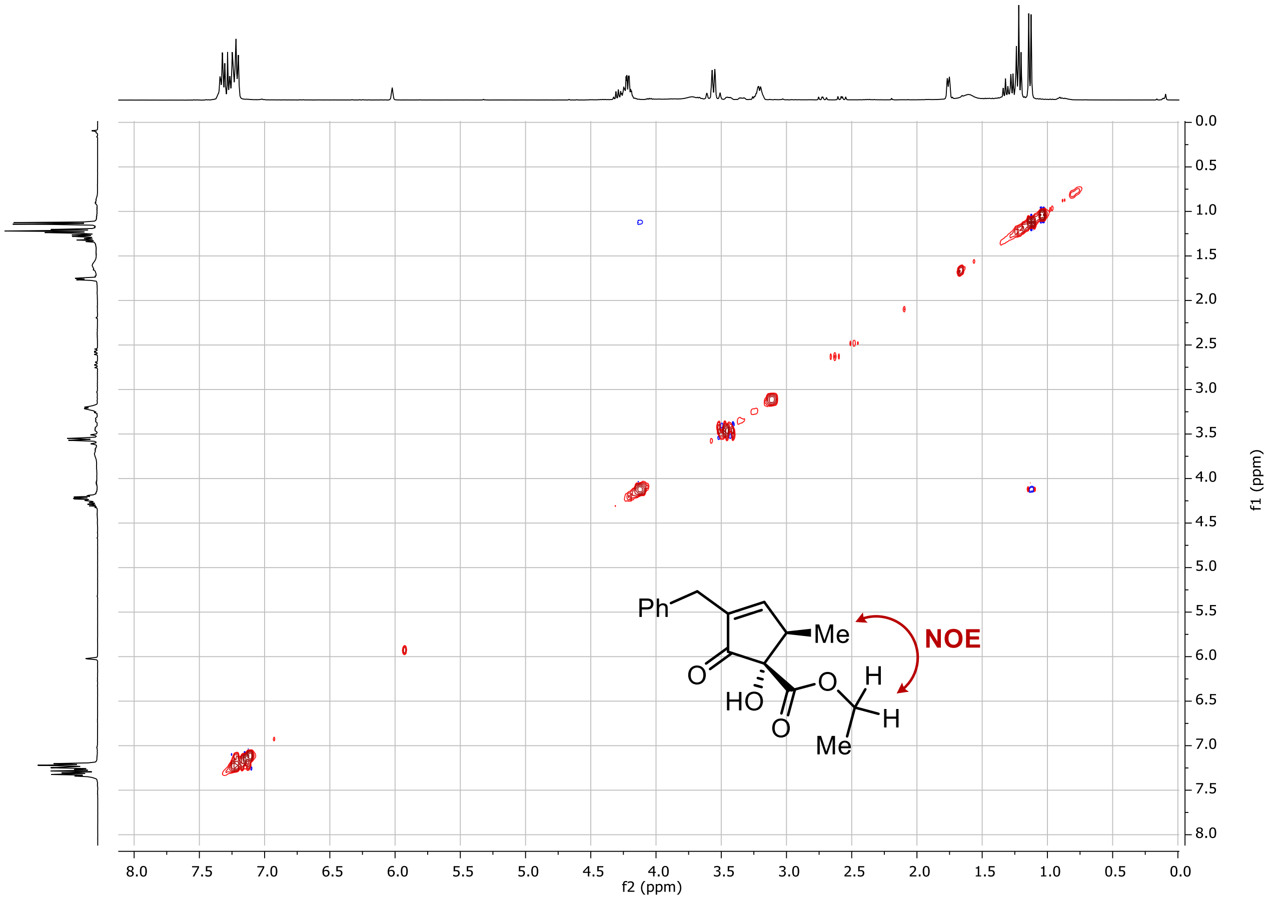

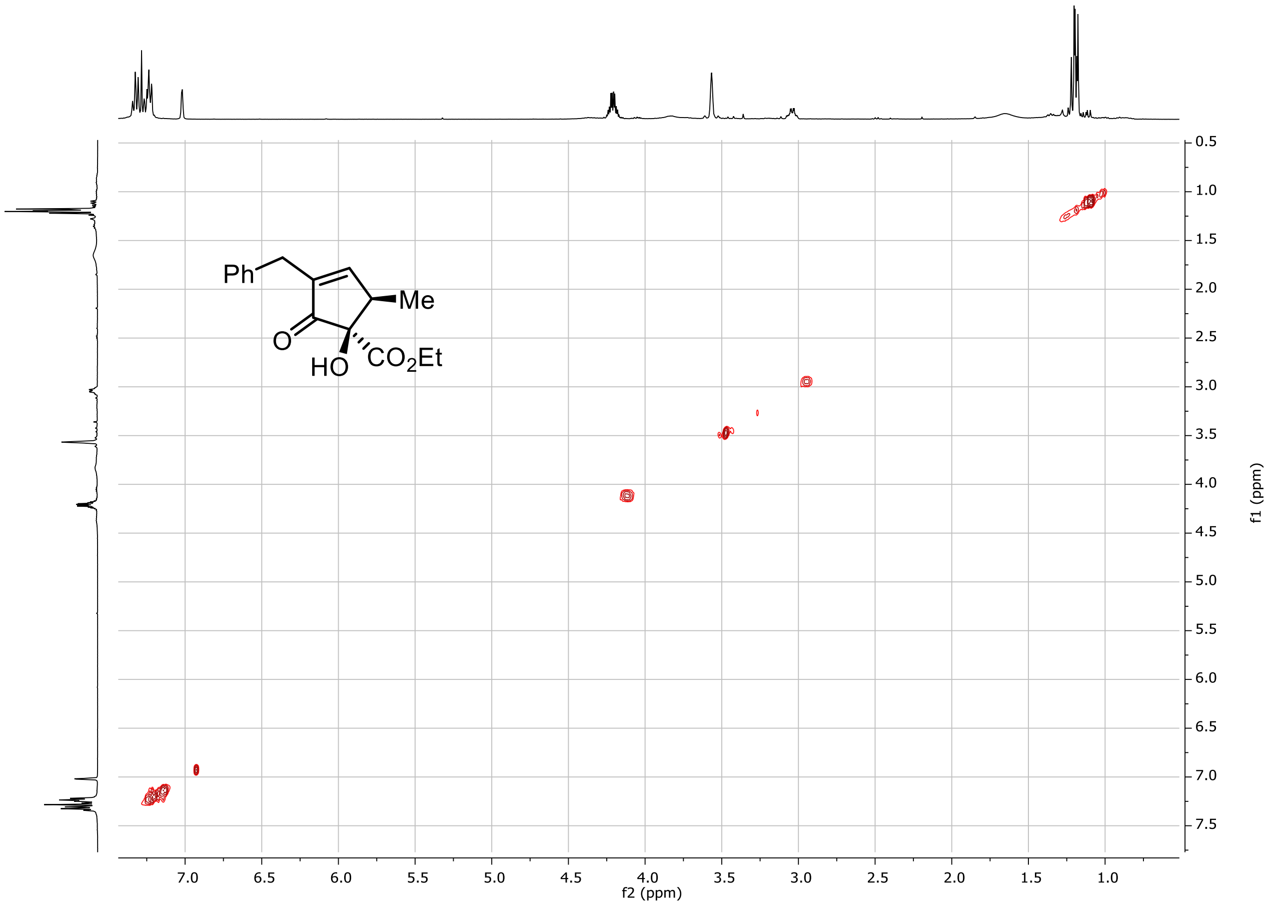

Ethyl (1*R*,5*R*)-1-allyl-3-benzyl-5-methyl-2-oxocyclopent-3-ene-1-carboxylate (6)

1H NMR

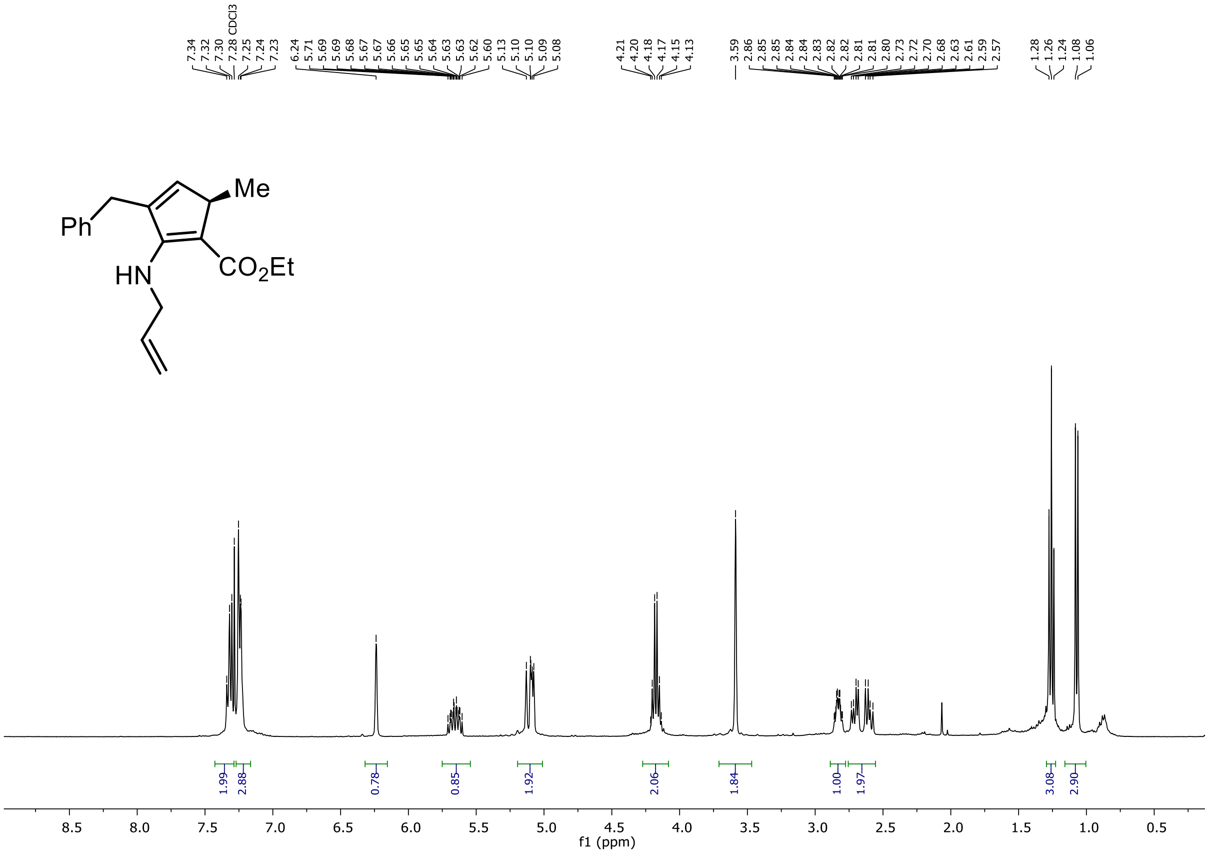

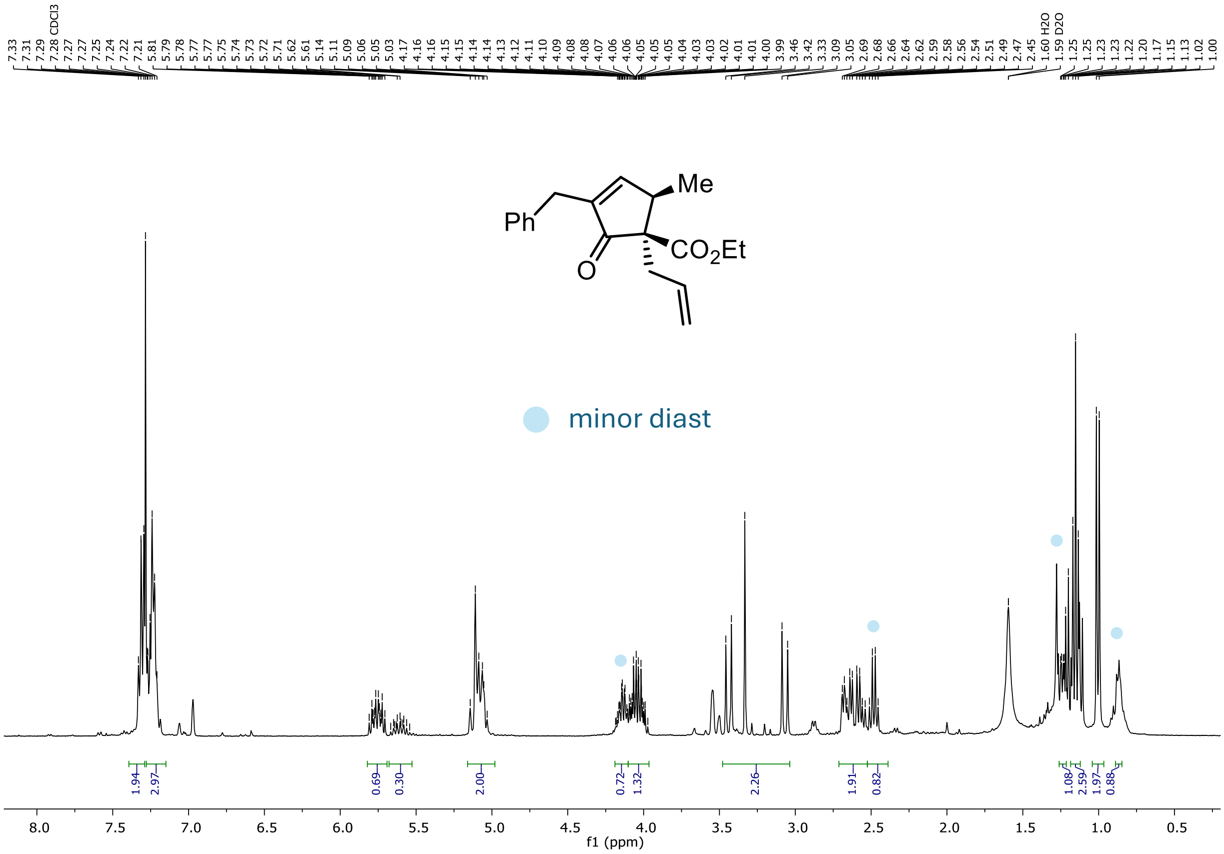

dr=7:3

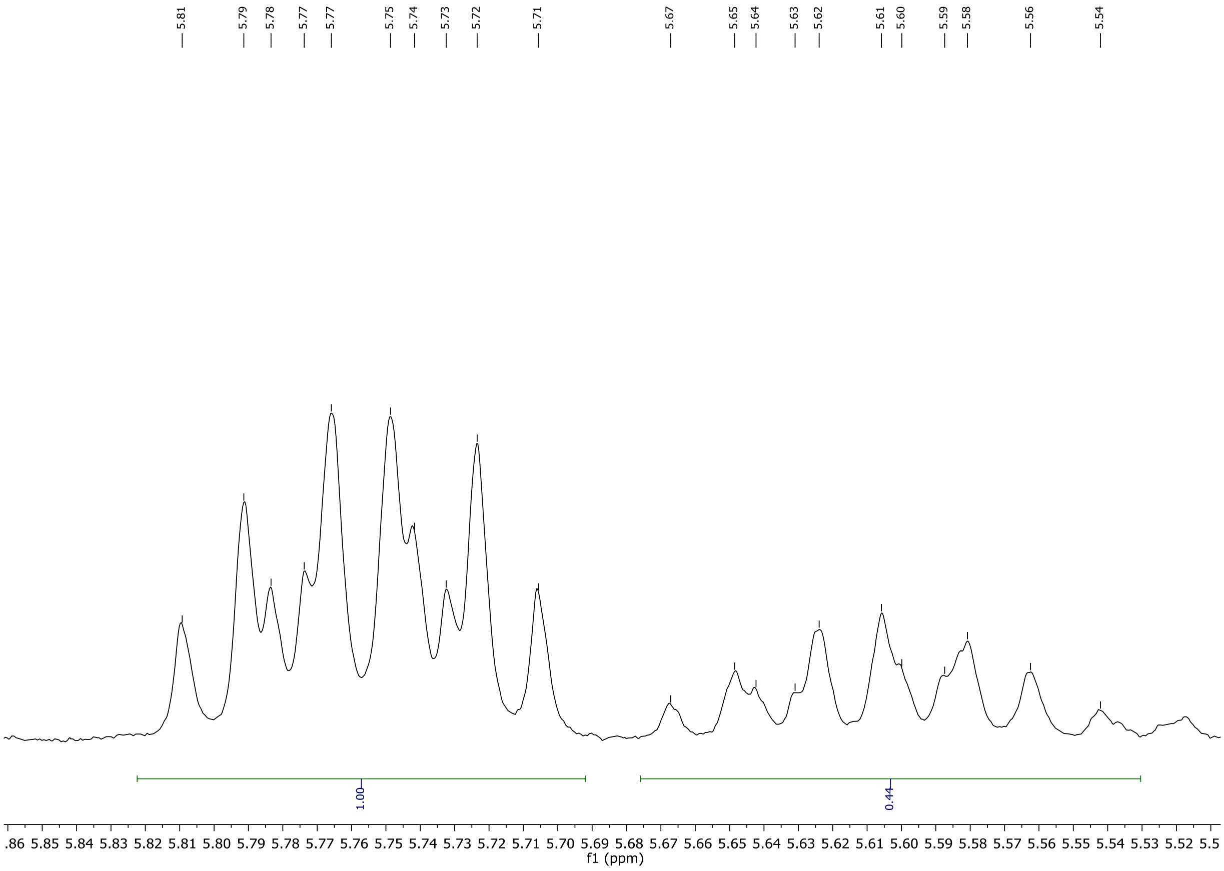

^{13}C NMR

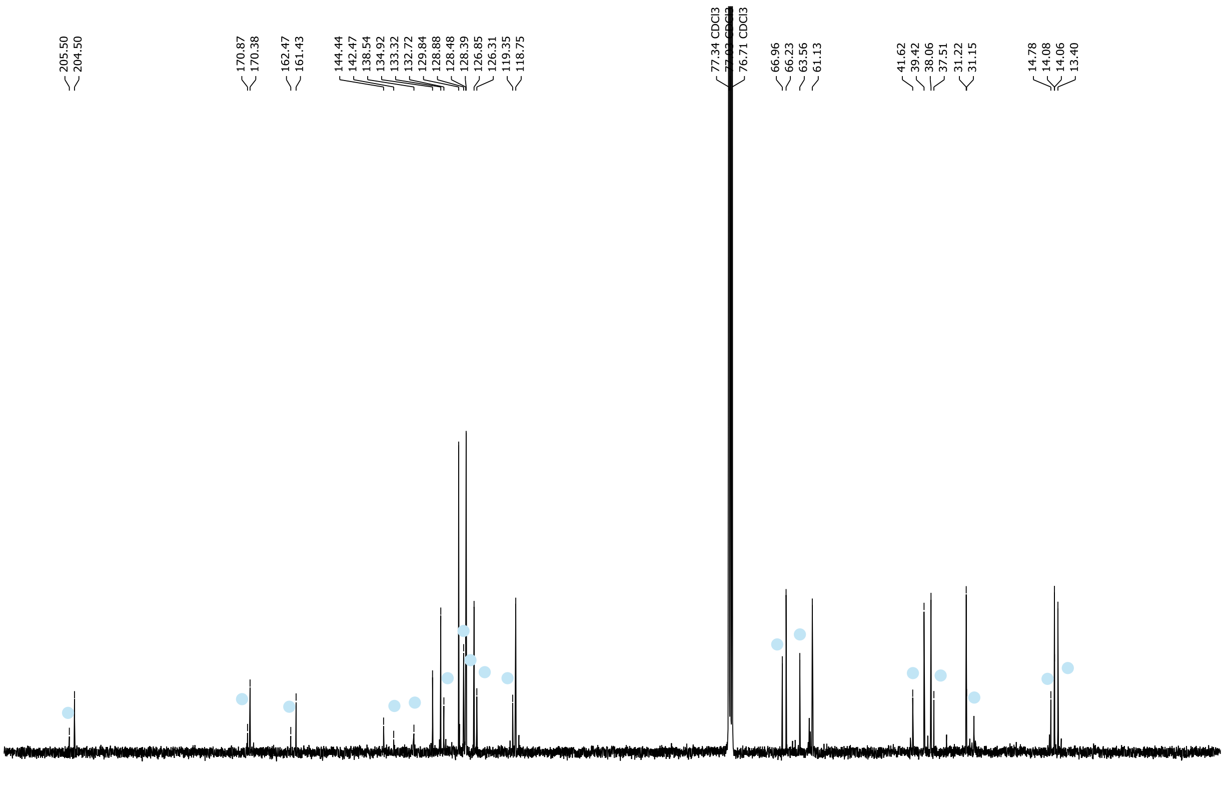

COSY (400 MHz, Chloroform-*d*)

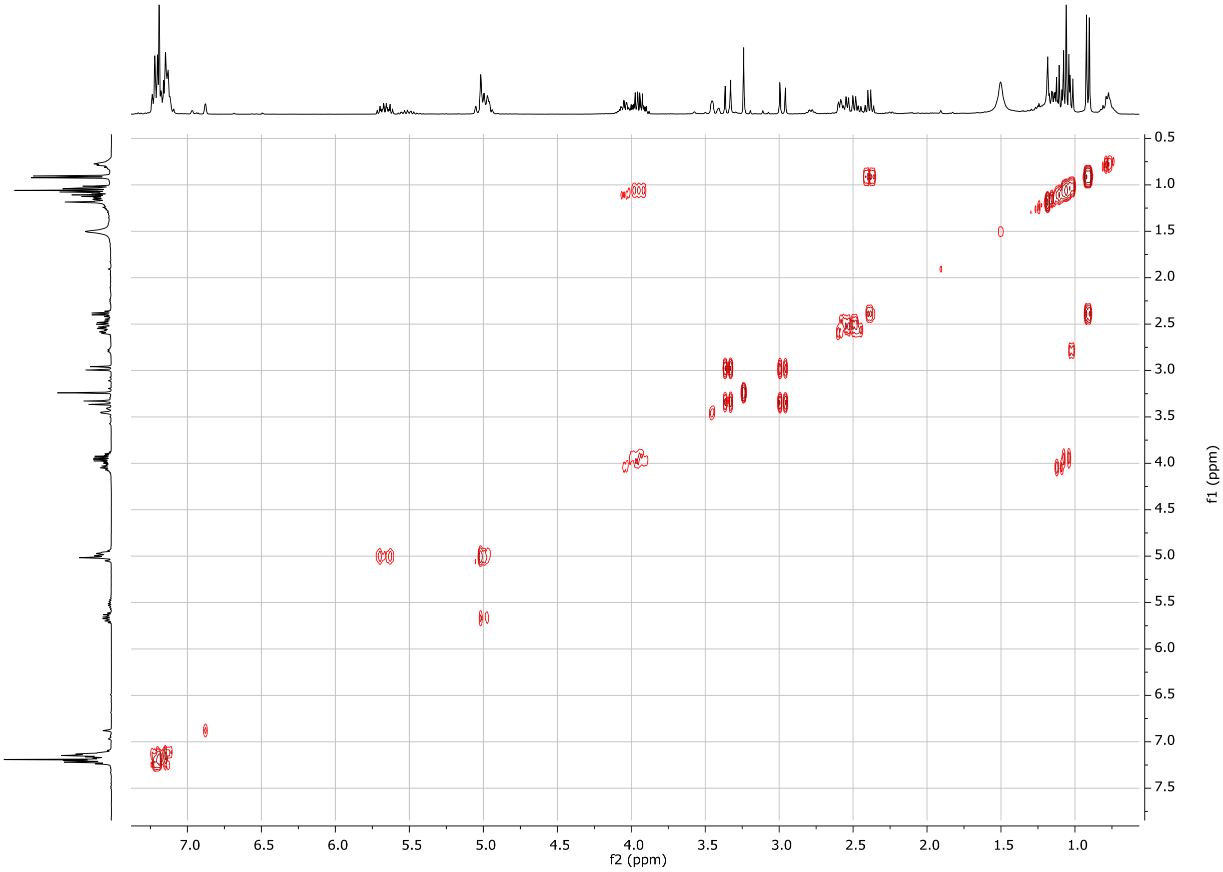

HSQC (400 MHz, Chloroform-*d*)

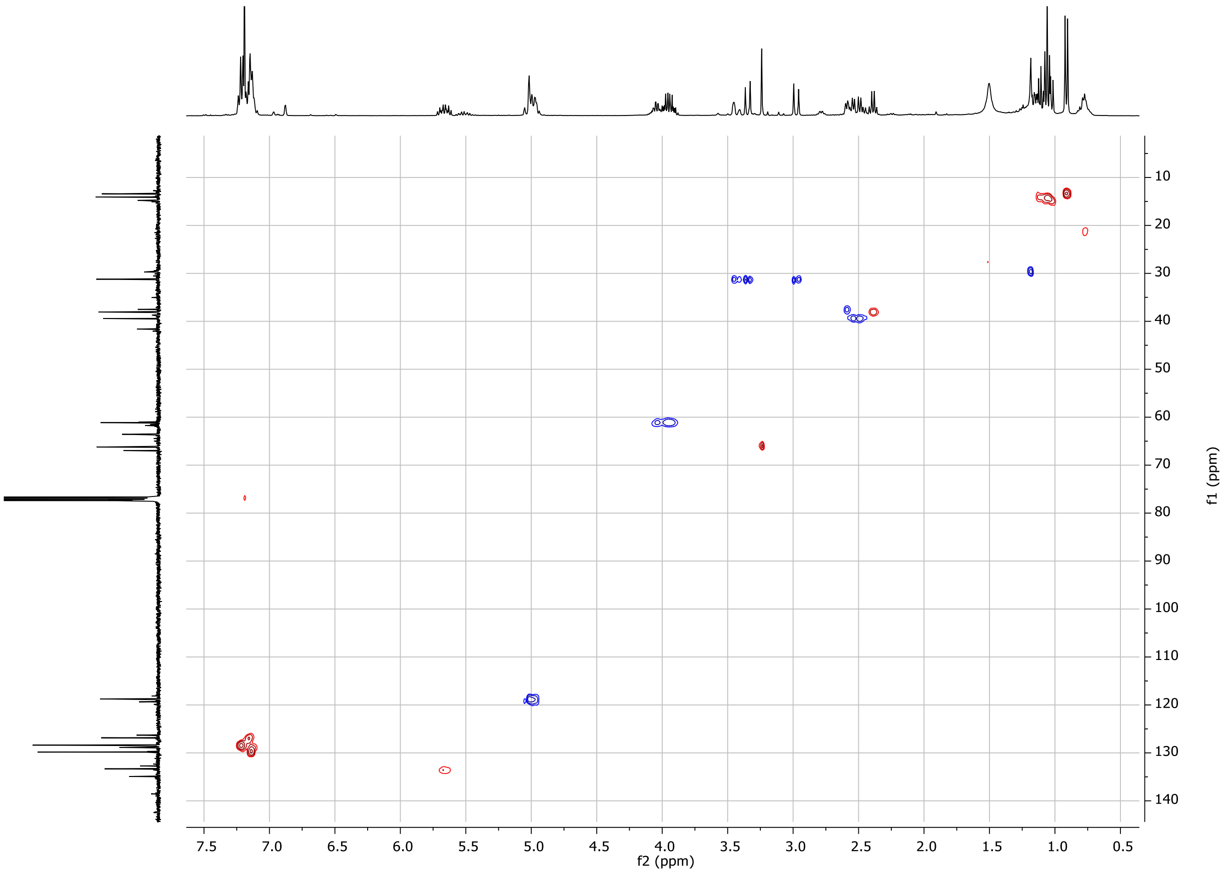

HMBC (400 MHz, Chloroform-*d*)

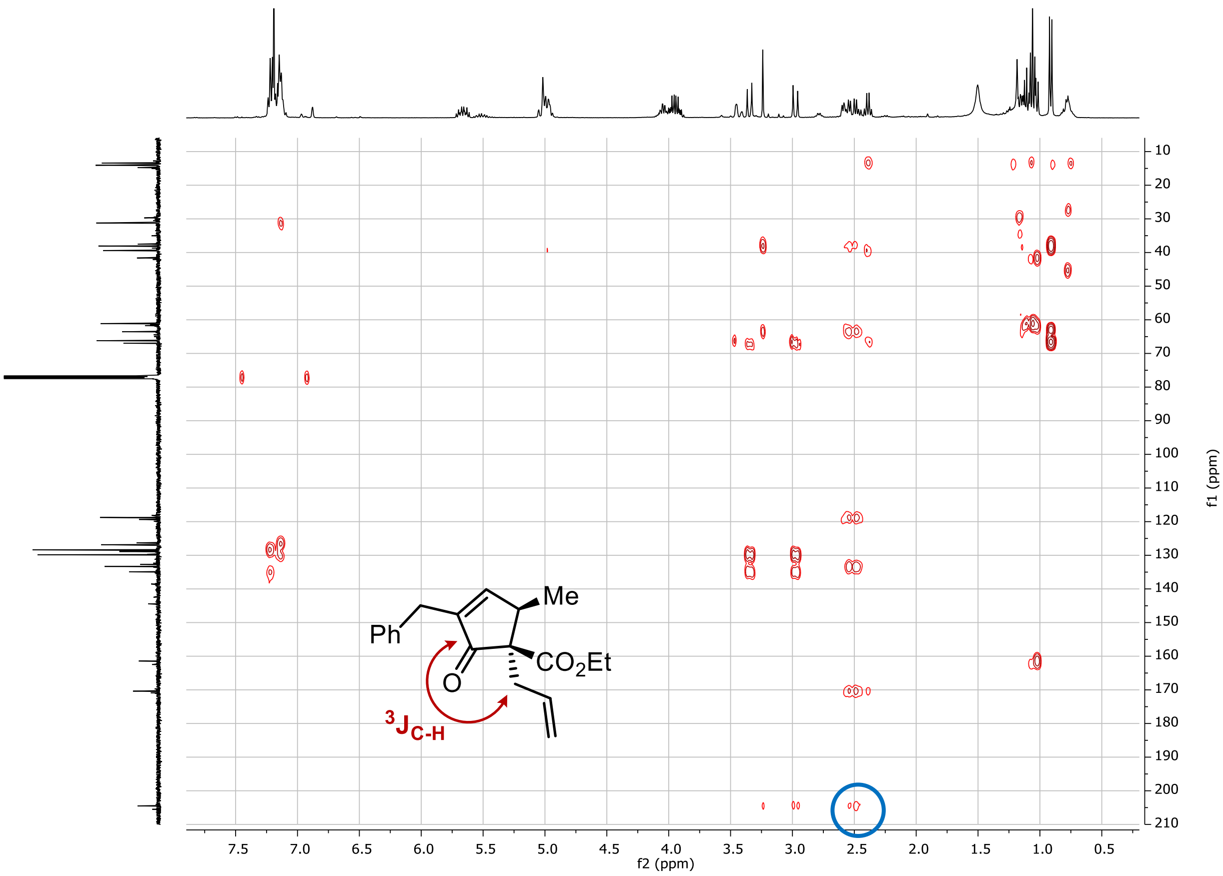

Ethyl (1*S*,5*R*)-3-benzyl-5-methyl-2-oxo-1-(p-tolyl)cyclopent-3-ene-1-carboxylate (7)

^{1}H NMR (*dr*>20:1)

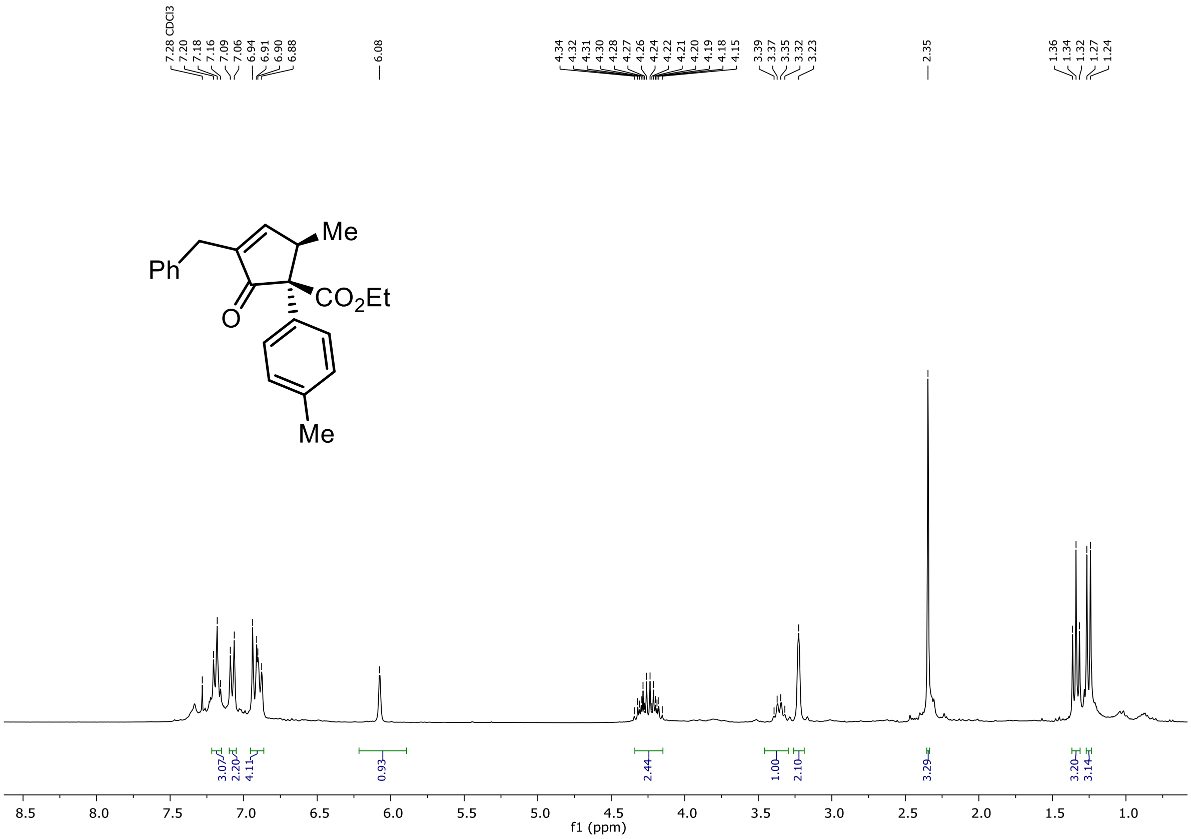

^{13}C NMR

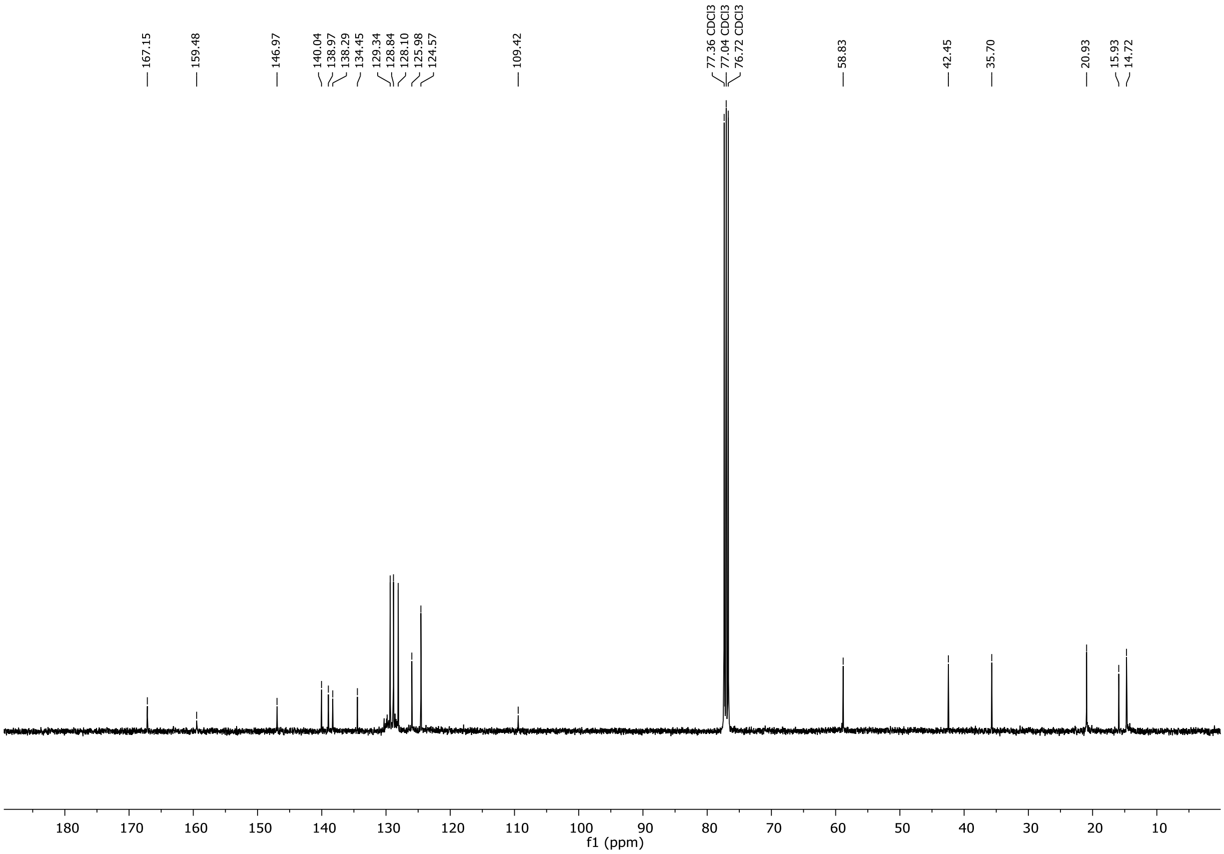

NOESY (400 MHz, Chloroform-*d*)

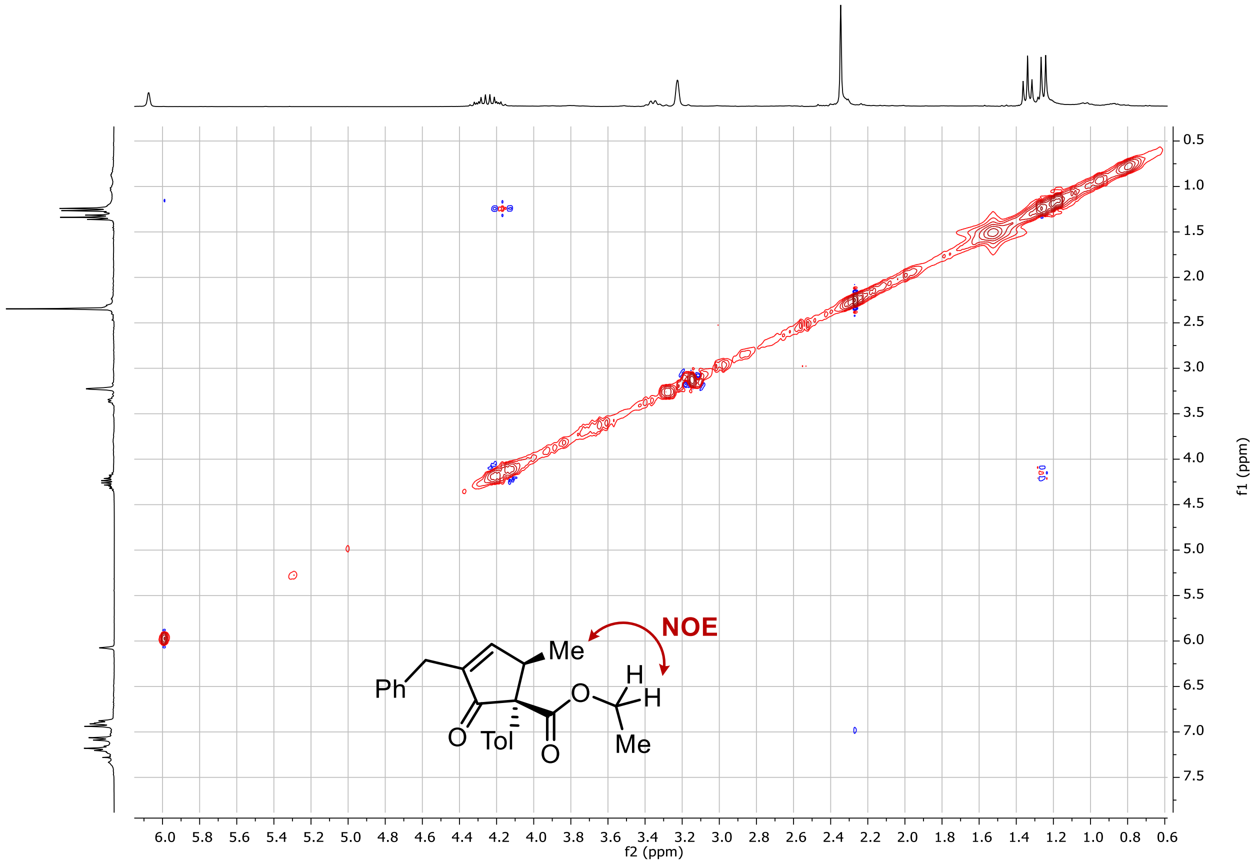

Ethyl (3a*S*,4*R*,7*S*,7a*S*,8*R*)-5-amino-6-benzyl-8-methyl-1,3-dioxo-2-phenyl-1,2,3,3a,7,7a-hexahydro-4H-4,7-methanoisoindole-4-carboxylate (8)

^{1}H NMR (*dr*>20:1)

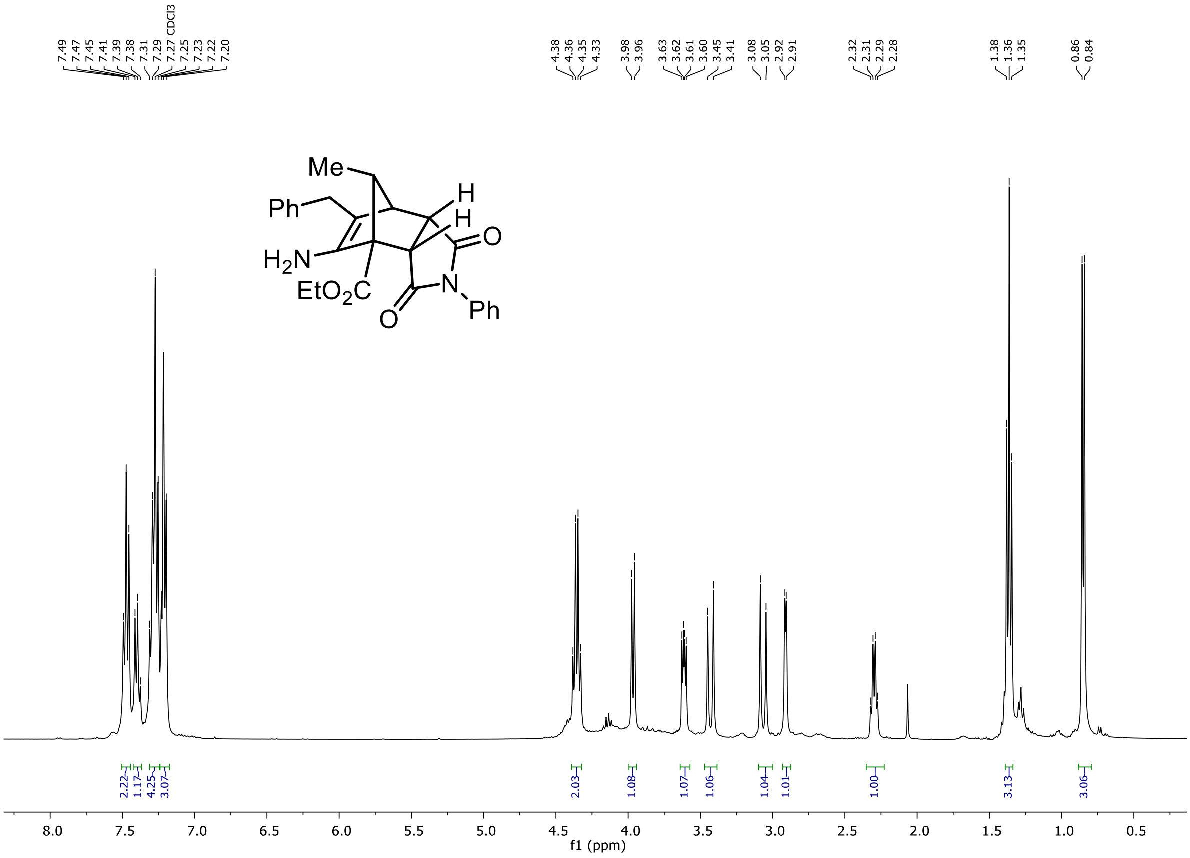

^{13}C NMR

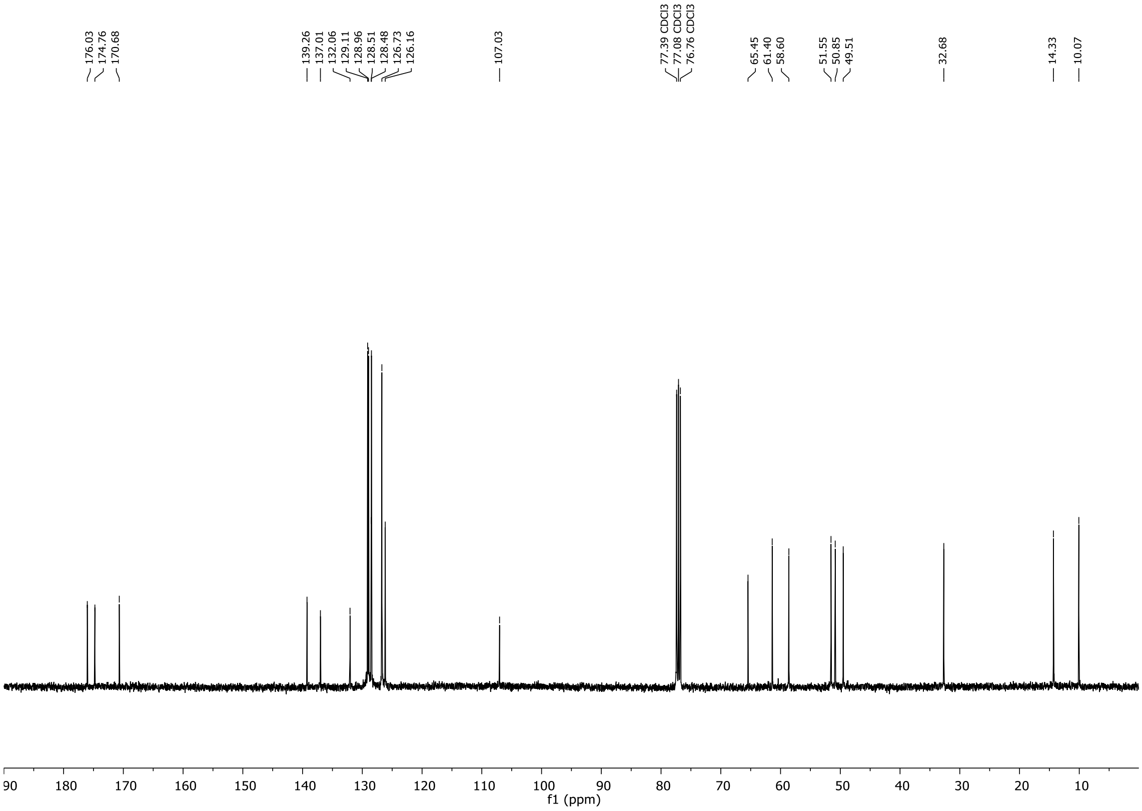

COSY (400 MHZ, Chloroform-*d*)

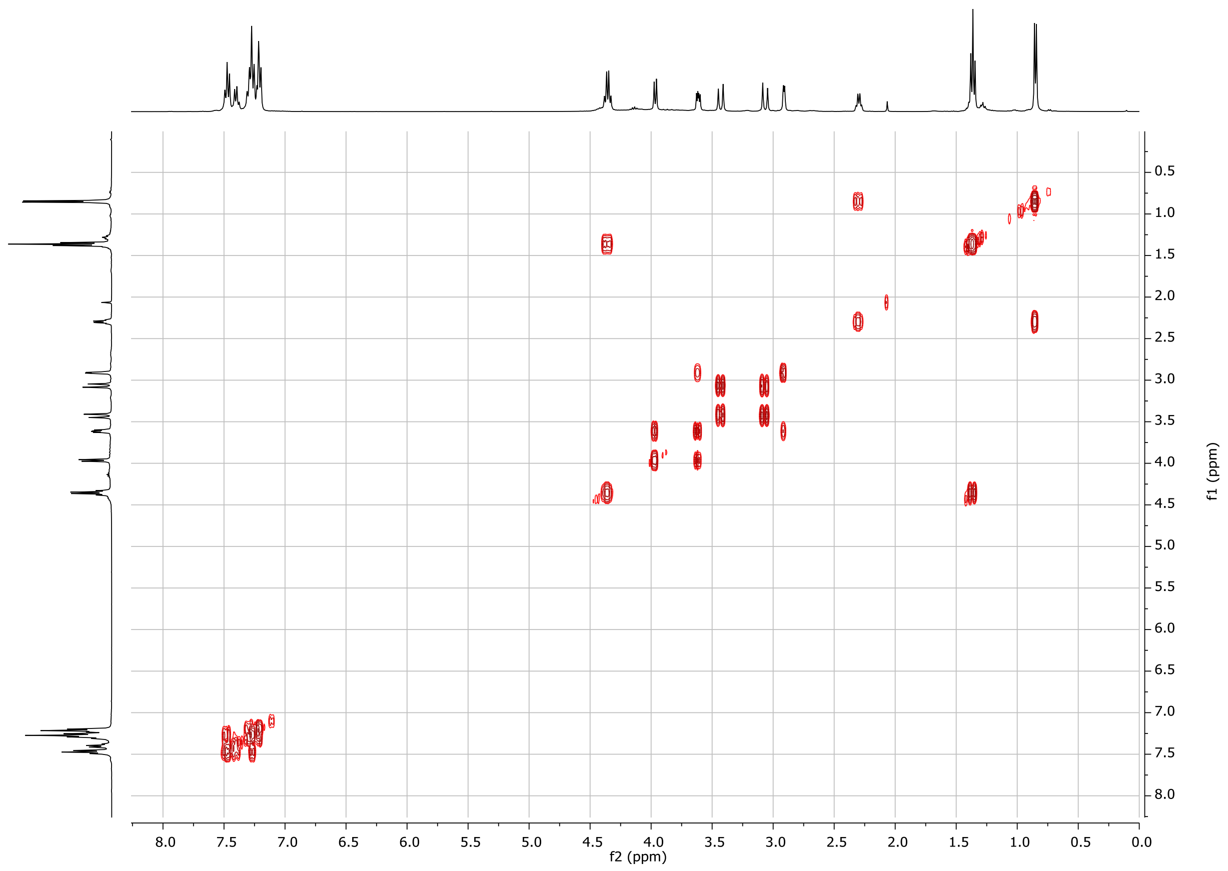

HSQC (400 MHz, Chloroform-*d*)

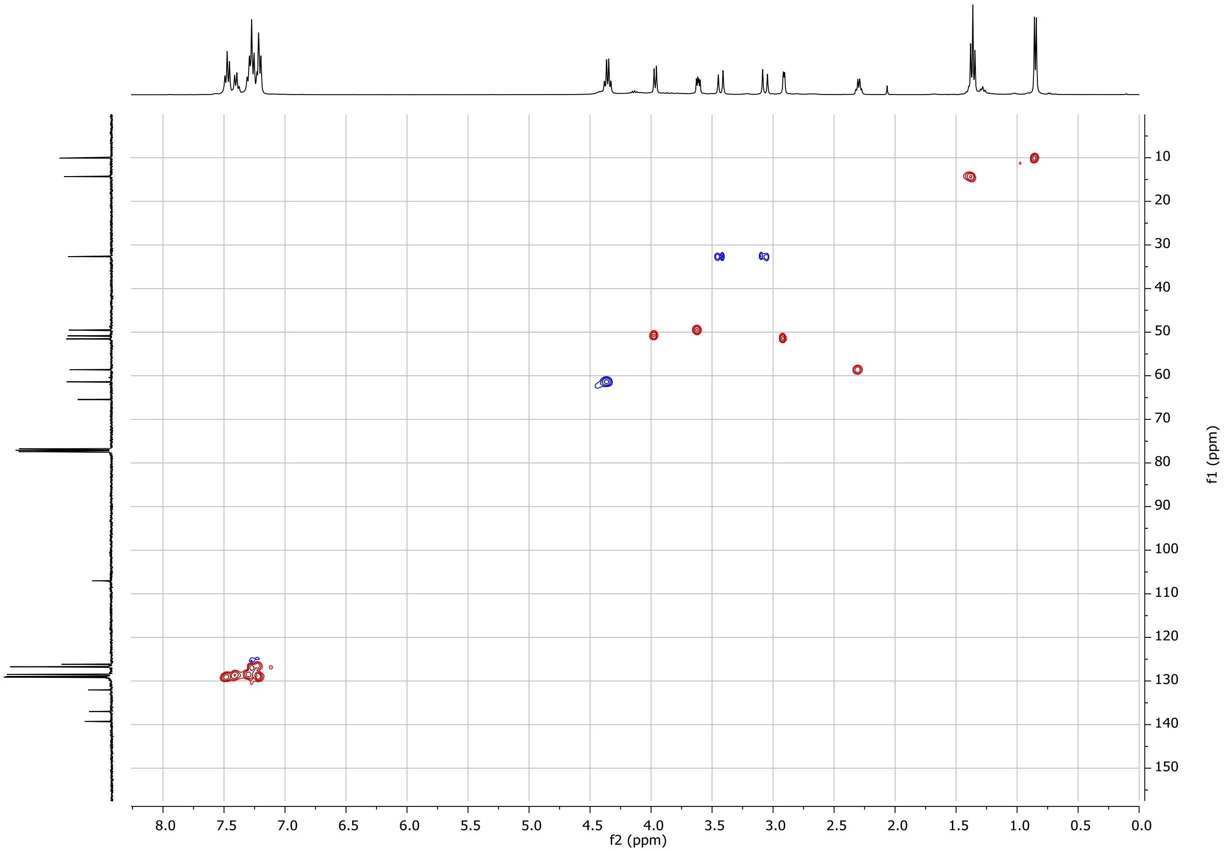

NOESY (400 MHZ, Chloroform-*d*)

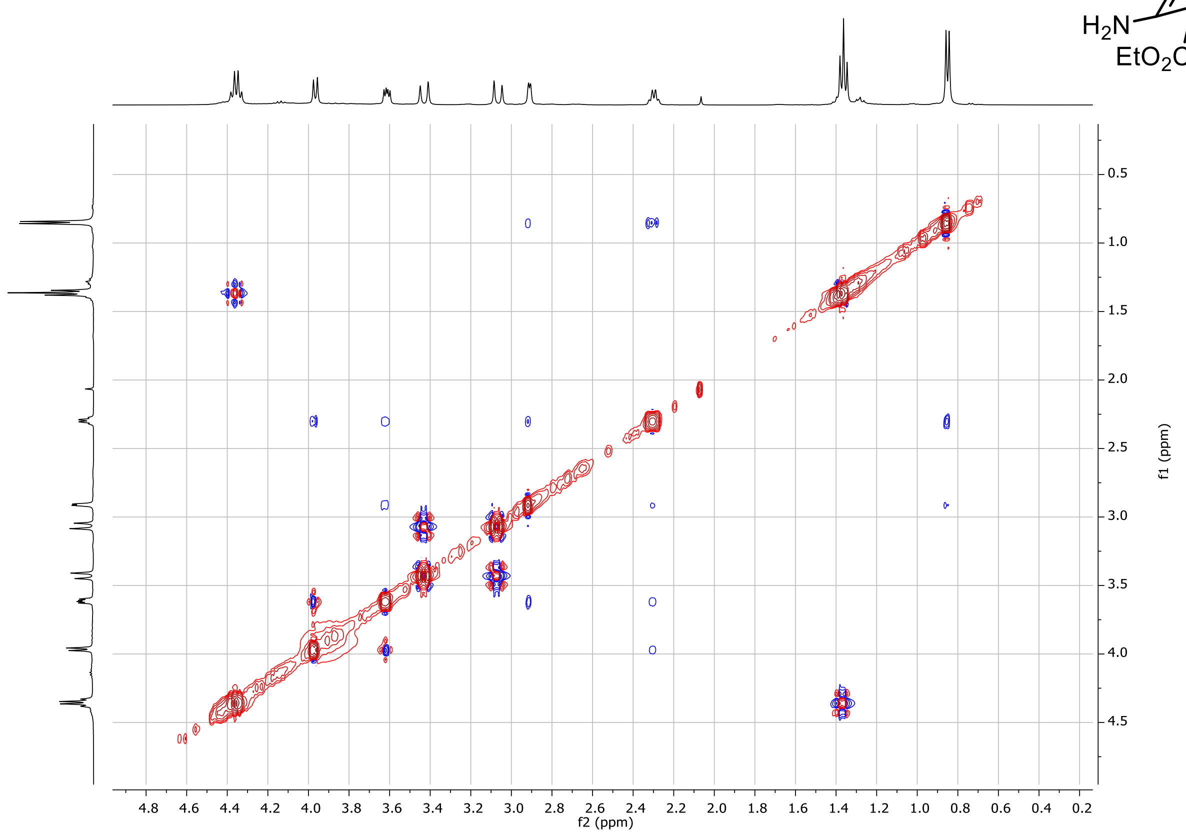

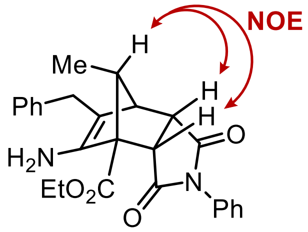

Ethyl (1*S*,3*R*,4*S*,5*R*,7*R*)-5-acetyl-3-benzyl-7-methyl-2-oxobicyclo[2.2.1]heptane-1-carboxylate (9)

^{1}H NMR (*dr*>20:1)

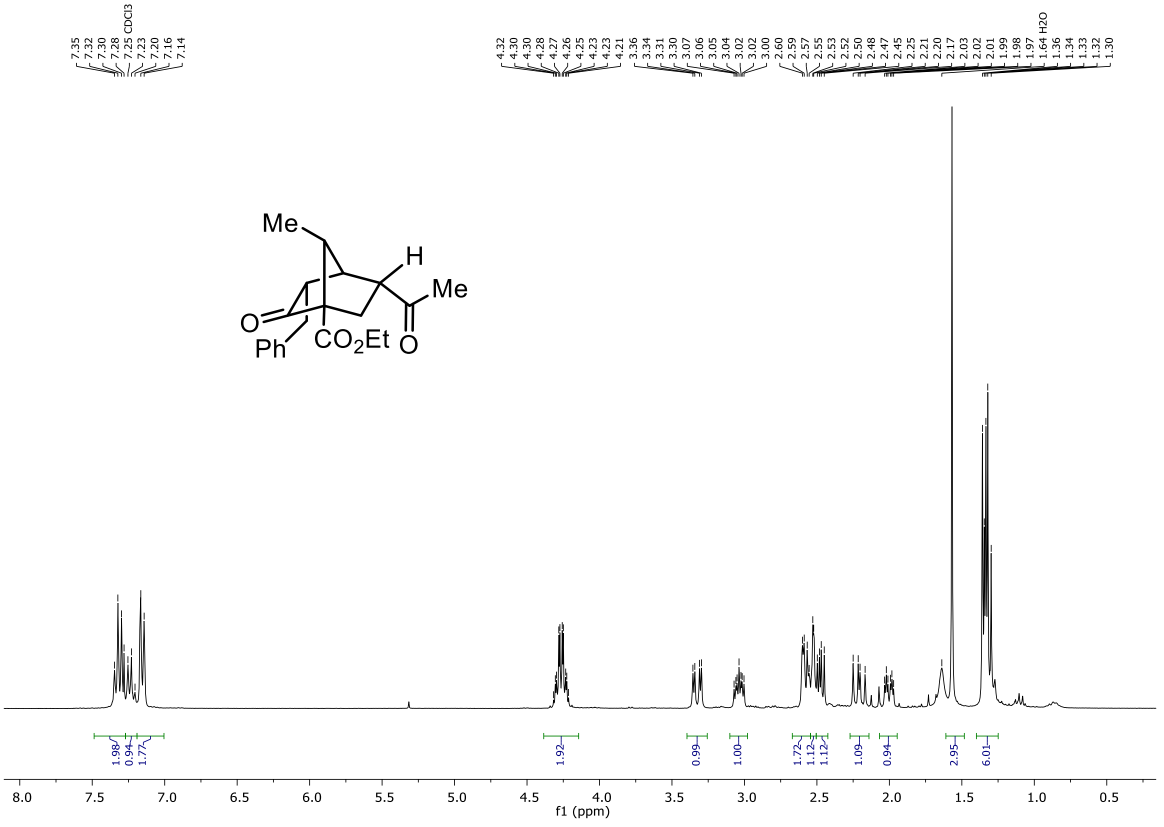

^{13}C NMR

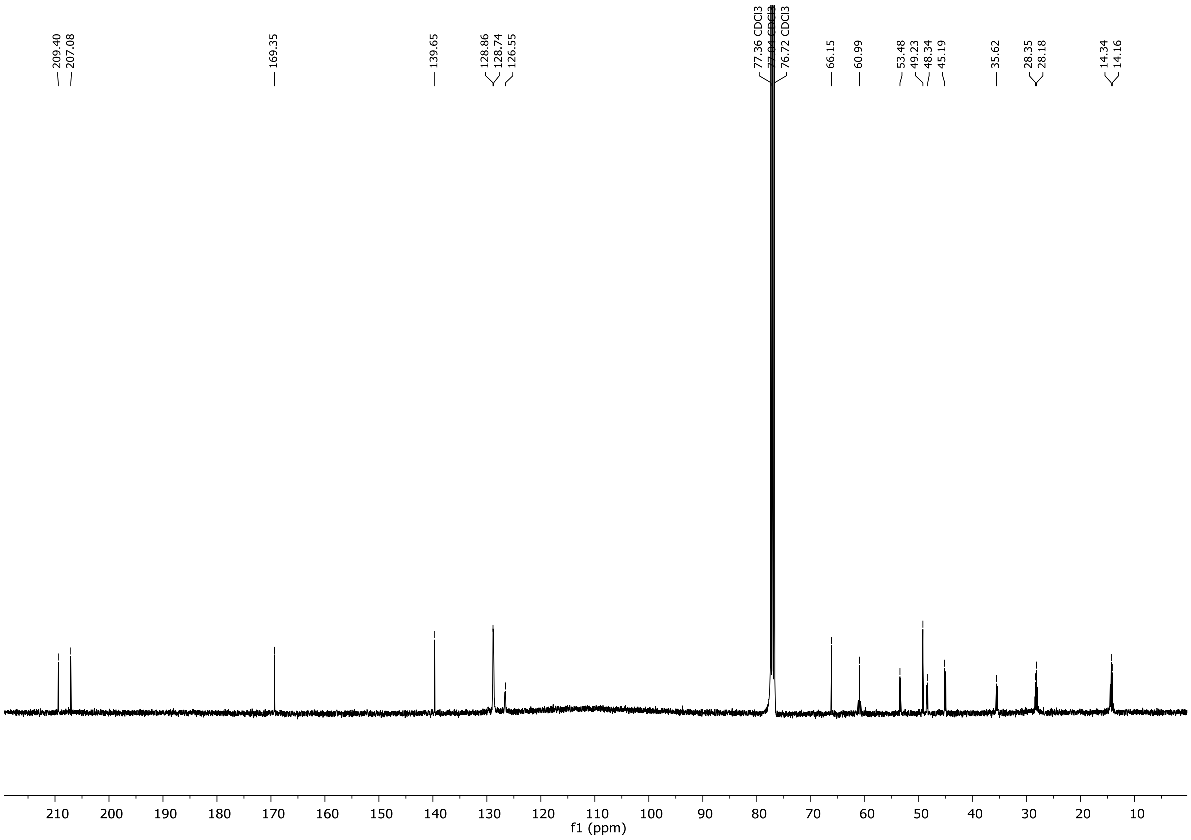

Comparison of the coupling constants (J values) of proton X with reported literature data[54] allowed the assignment of the depicted stereochemistry, corresponding to the kinetically favored *endo* product.

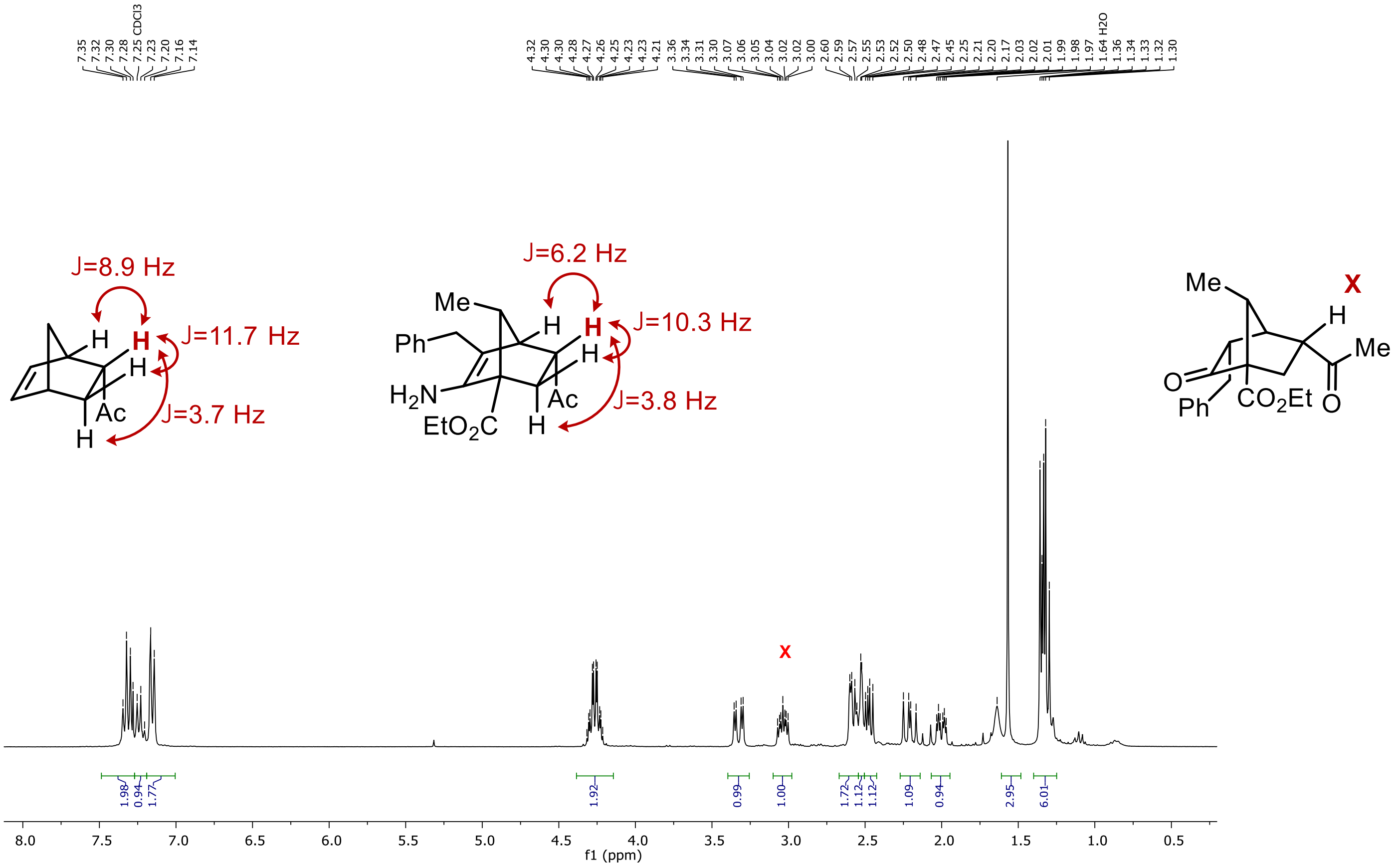

Ethyl (1*S*,4*R*,5*R*)-3-benzyl-4-((dimethoxyphosphoryl)oxy)-1-hydroxy-5-methyl-2-oxocyclopentane-1-carboxylate (11)

^{1}H NMR (*dr*=1:1)

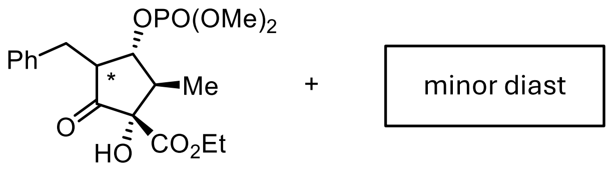

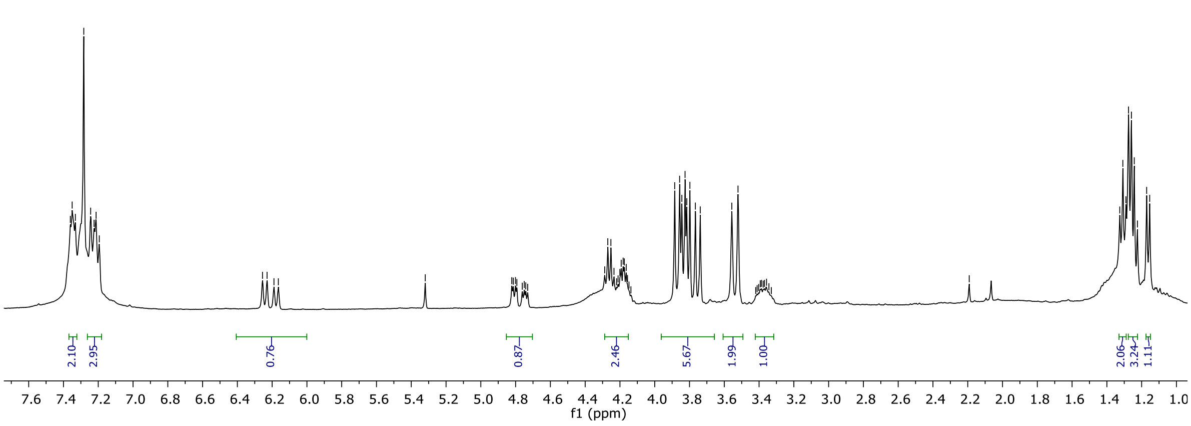

^{13}C NMR

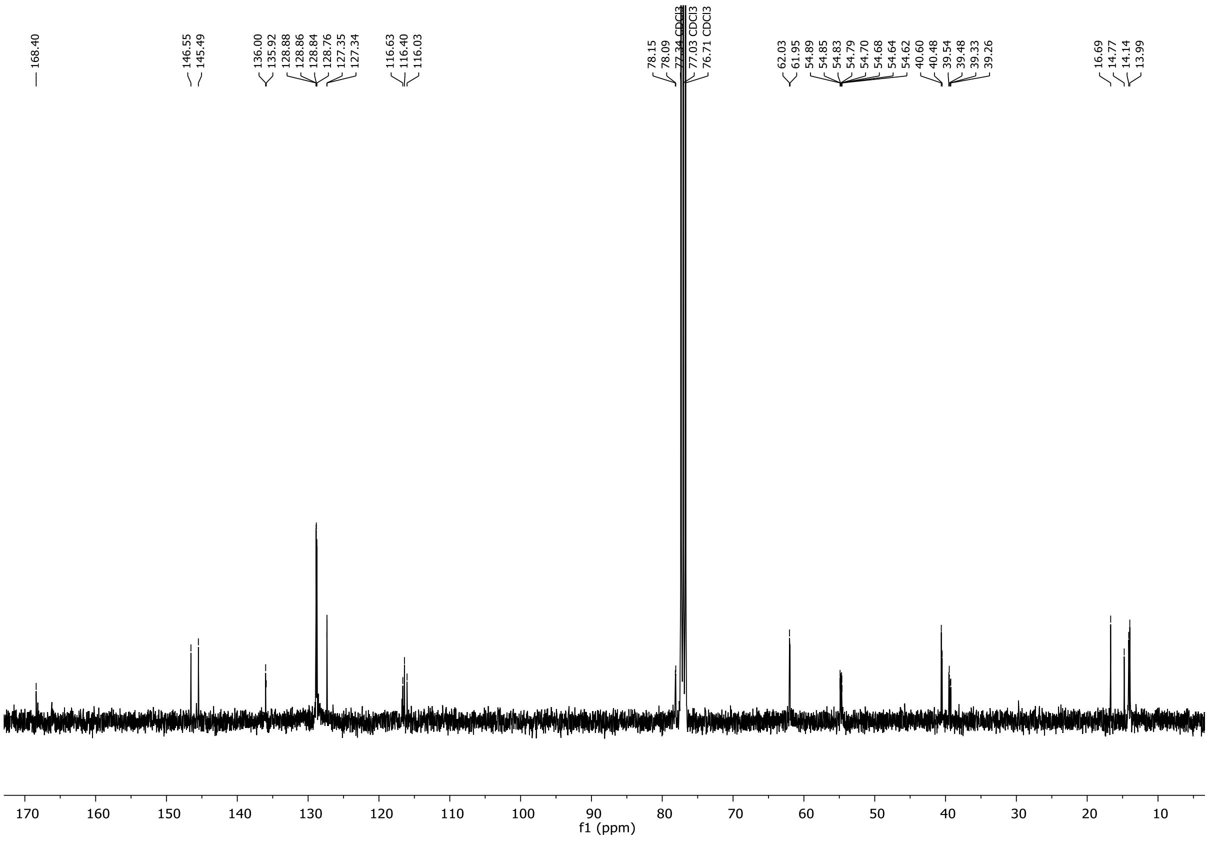

^{31}P NMR

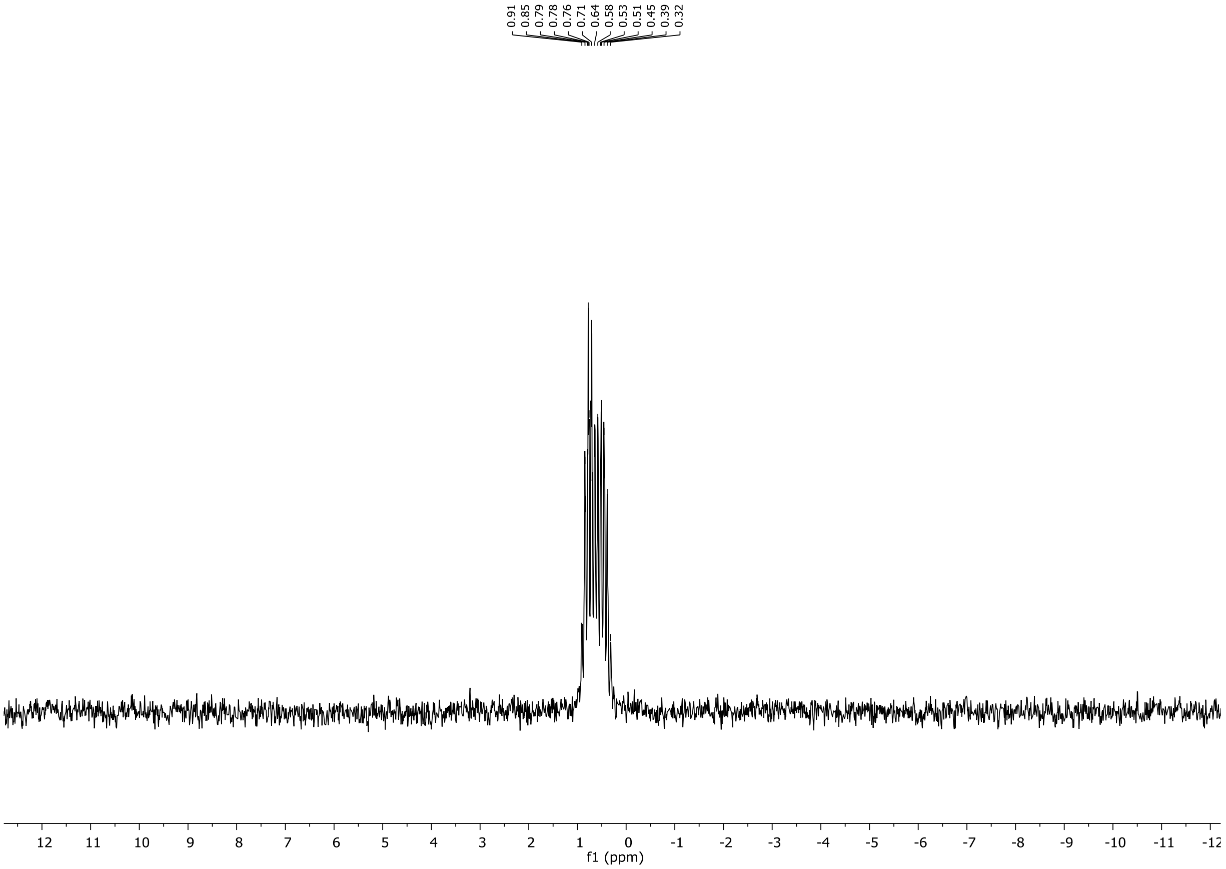

^{31}P NMR, decoupled

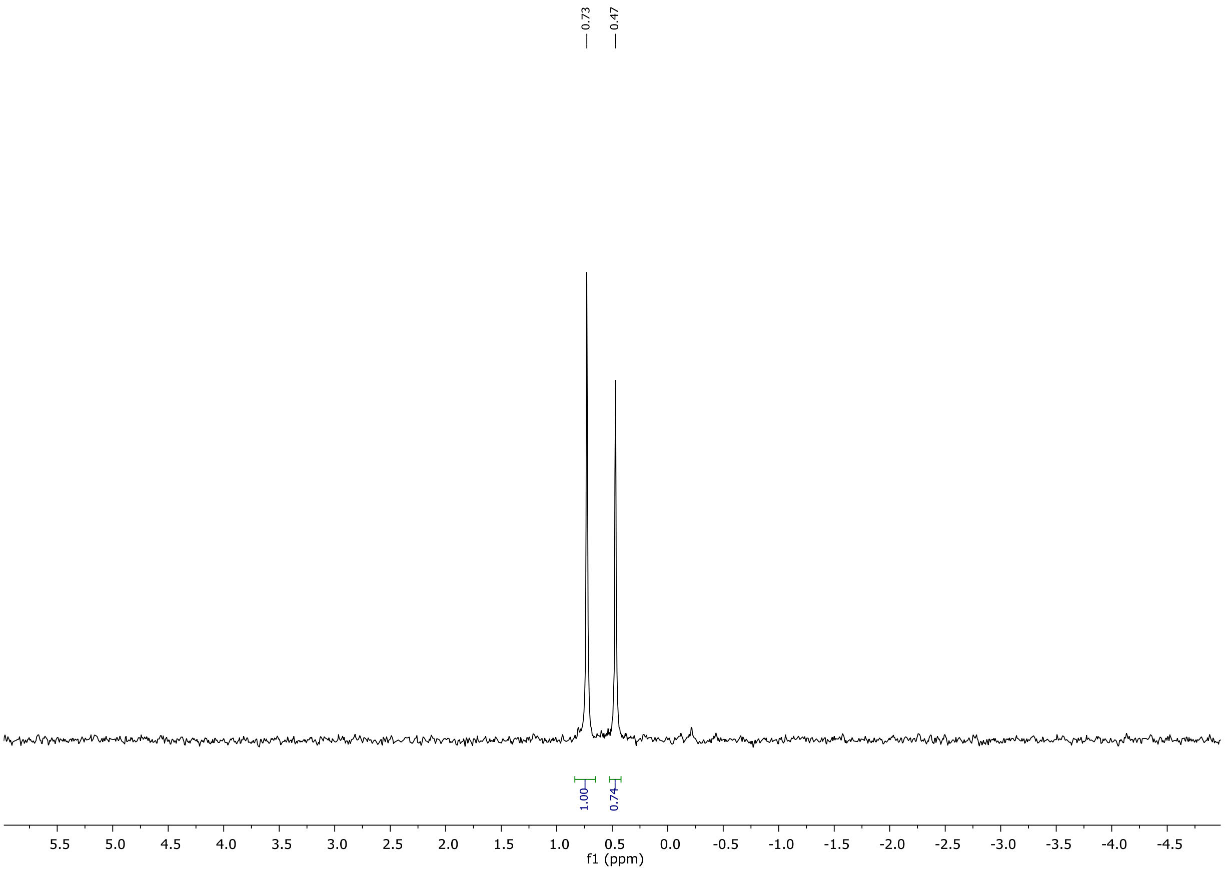

Ethyl (3*R*,5*R*)-2-amino-3-benzyl-5-methylcyclopent-1-ene-1-carboxylate (12)

^{1}H NMR

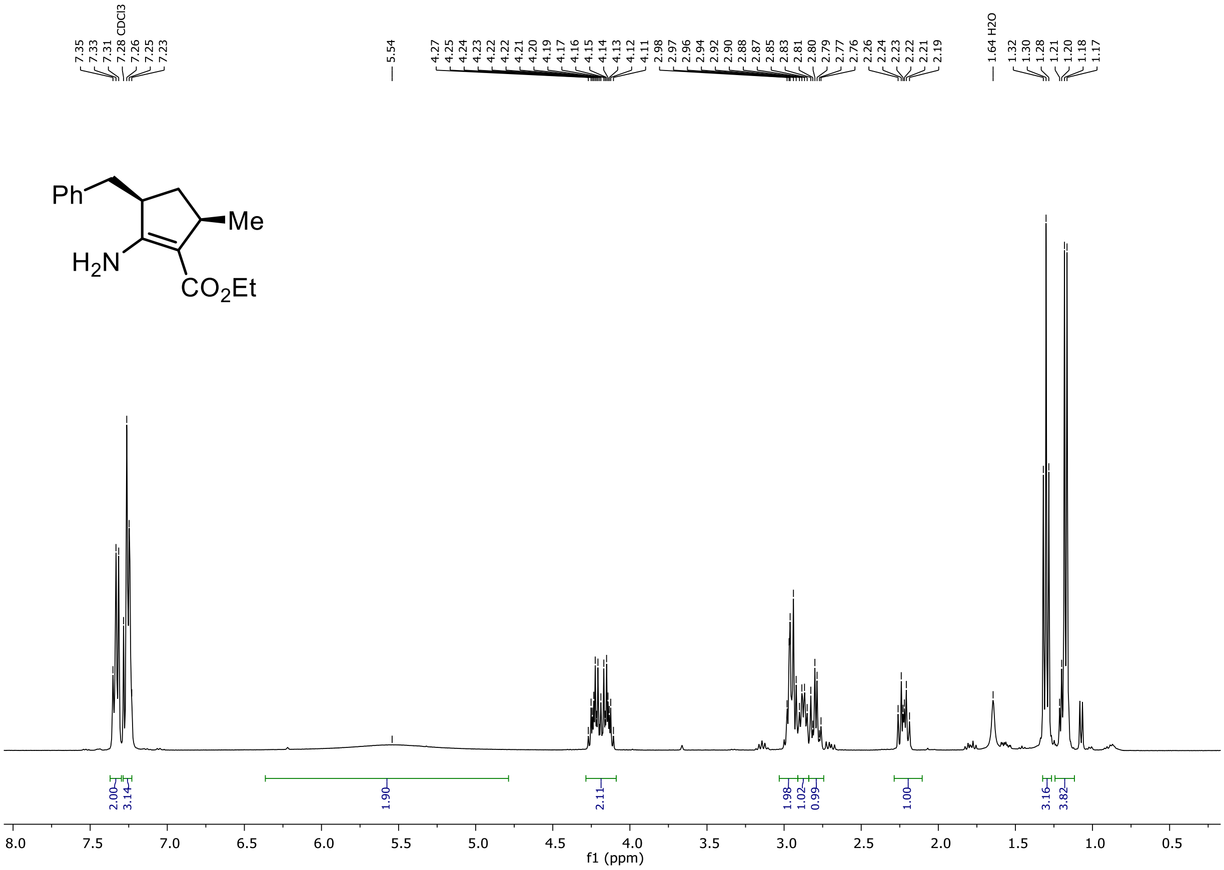

dr=9:1

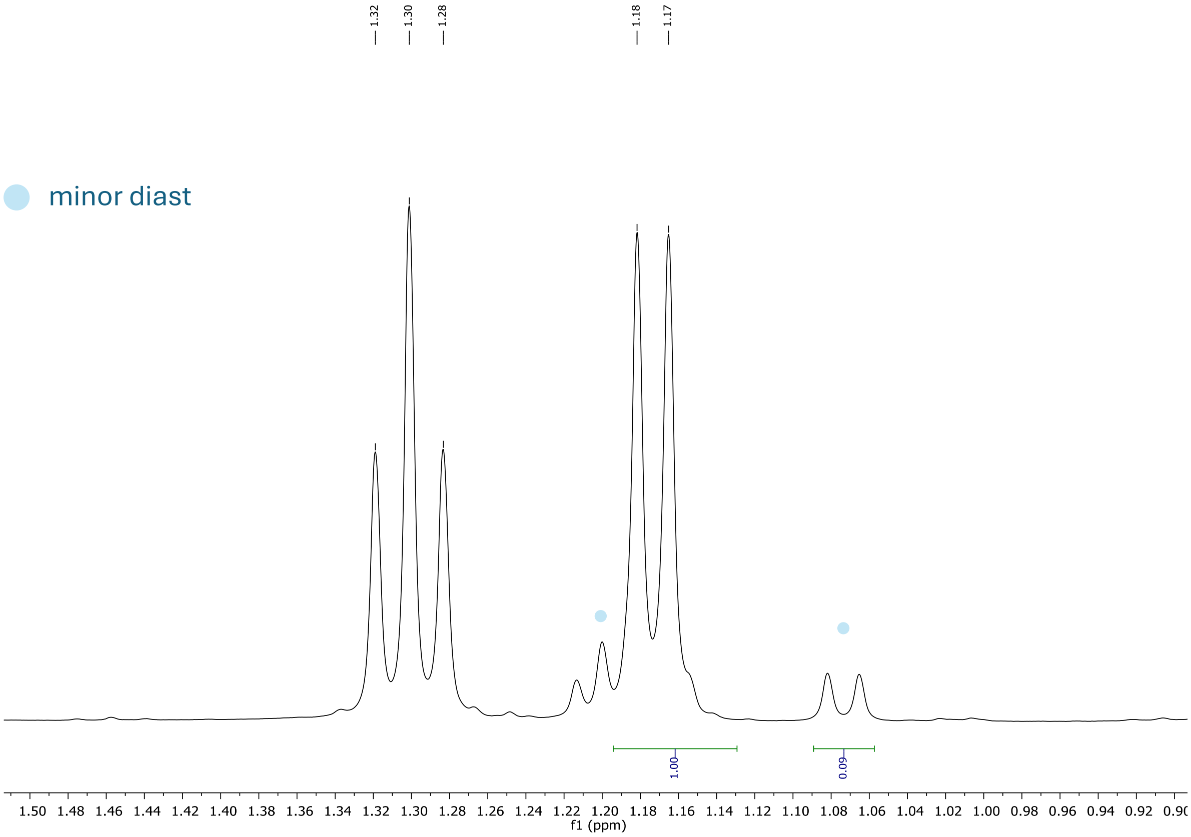

^{13}C NMR

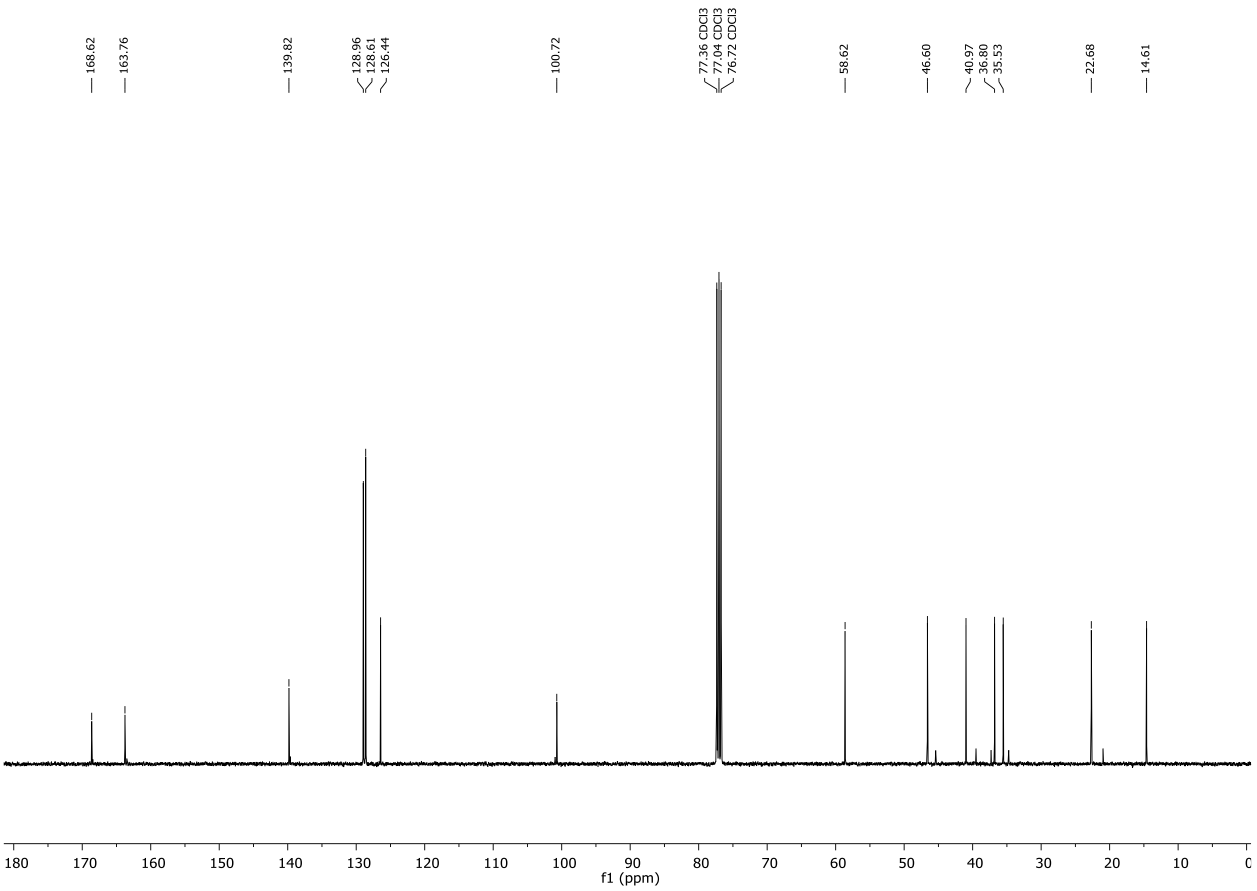

HSQC (400 MHz, Chloroform-*d*)

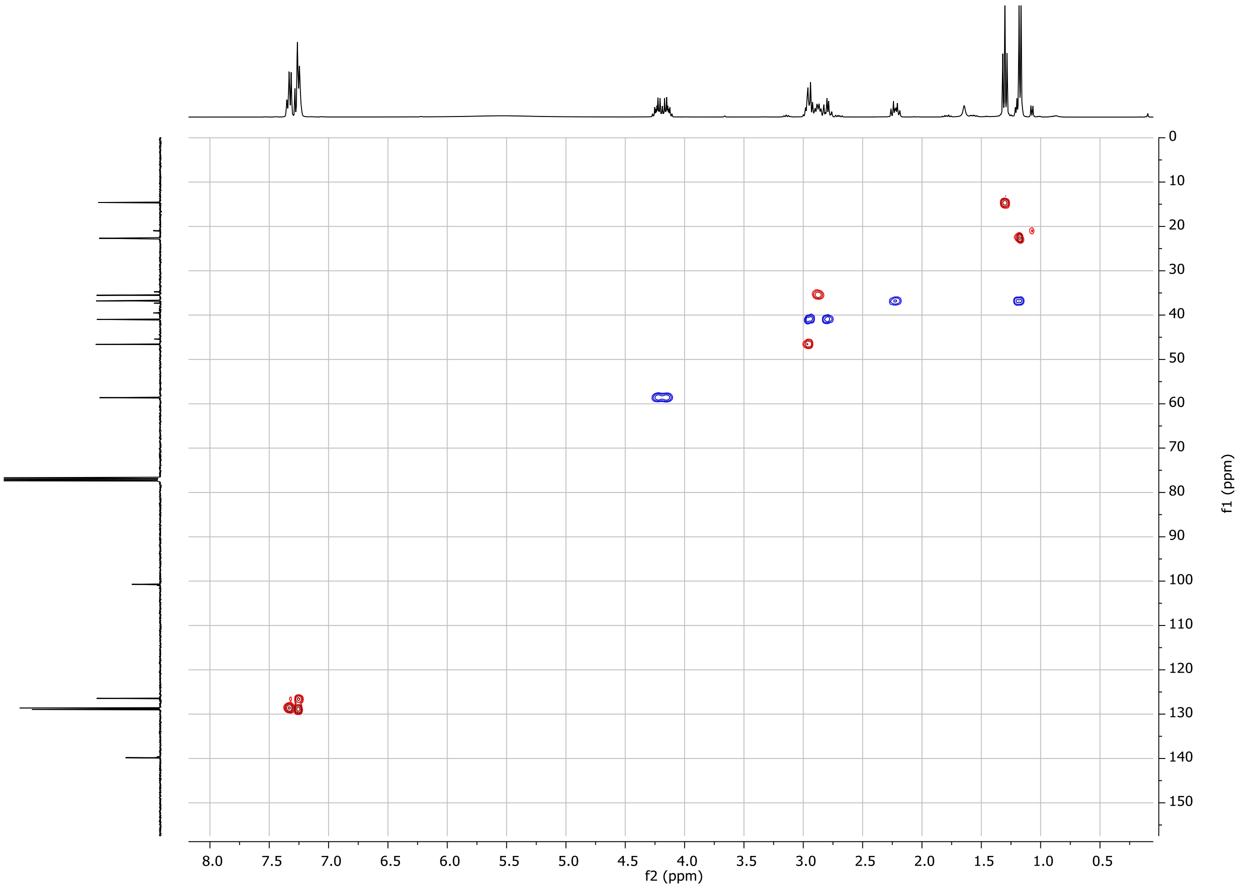

COSY (400 MHz, Chloroform-*d*)

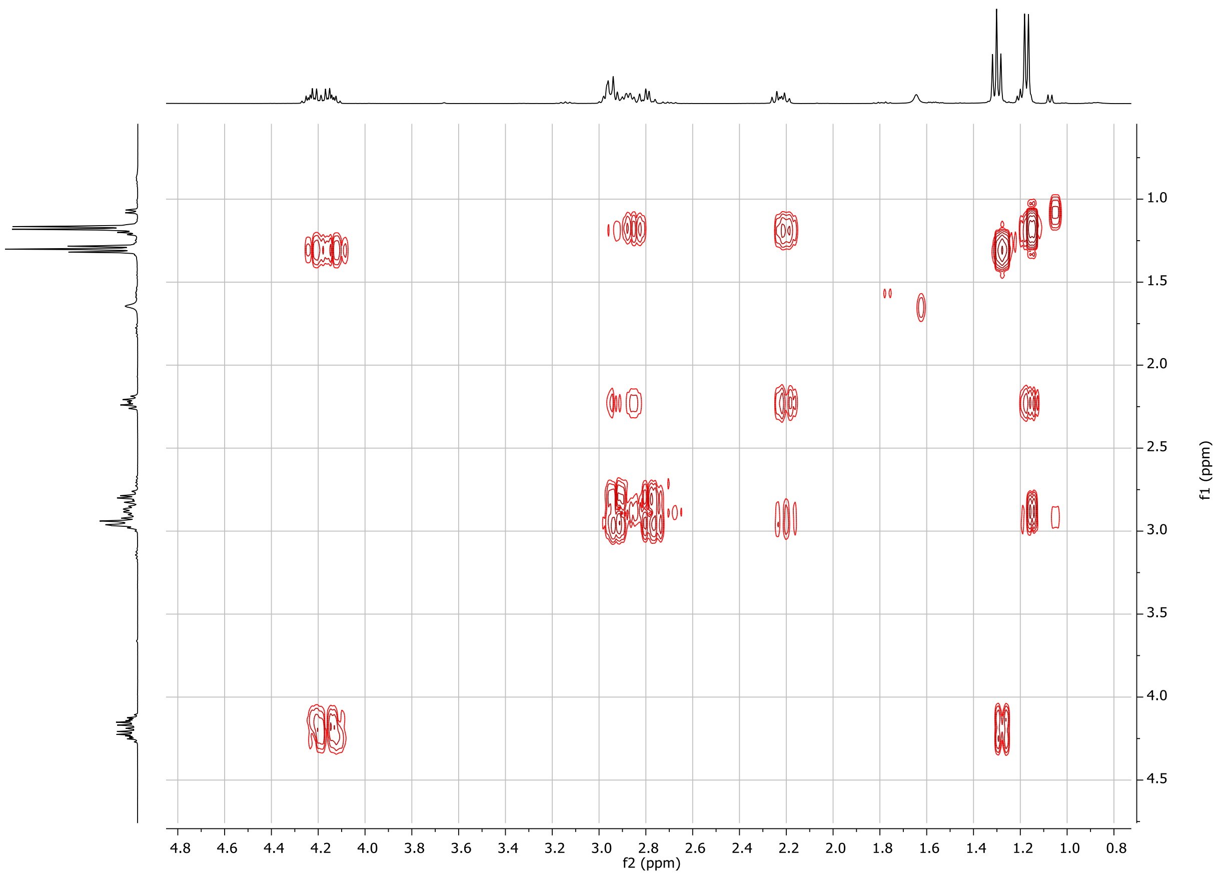

Peak assignments

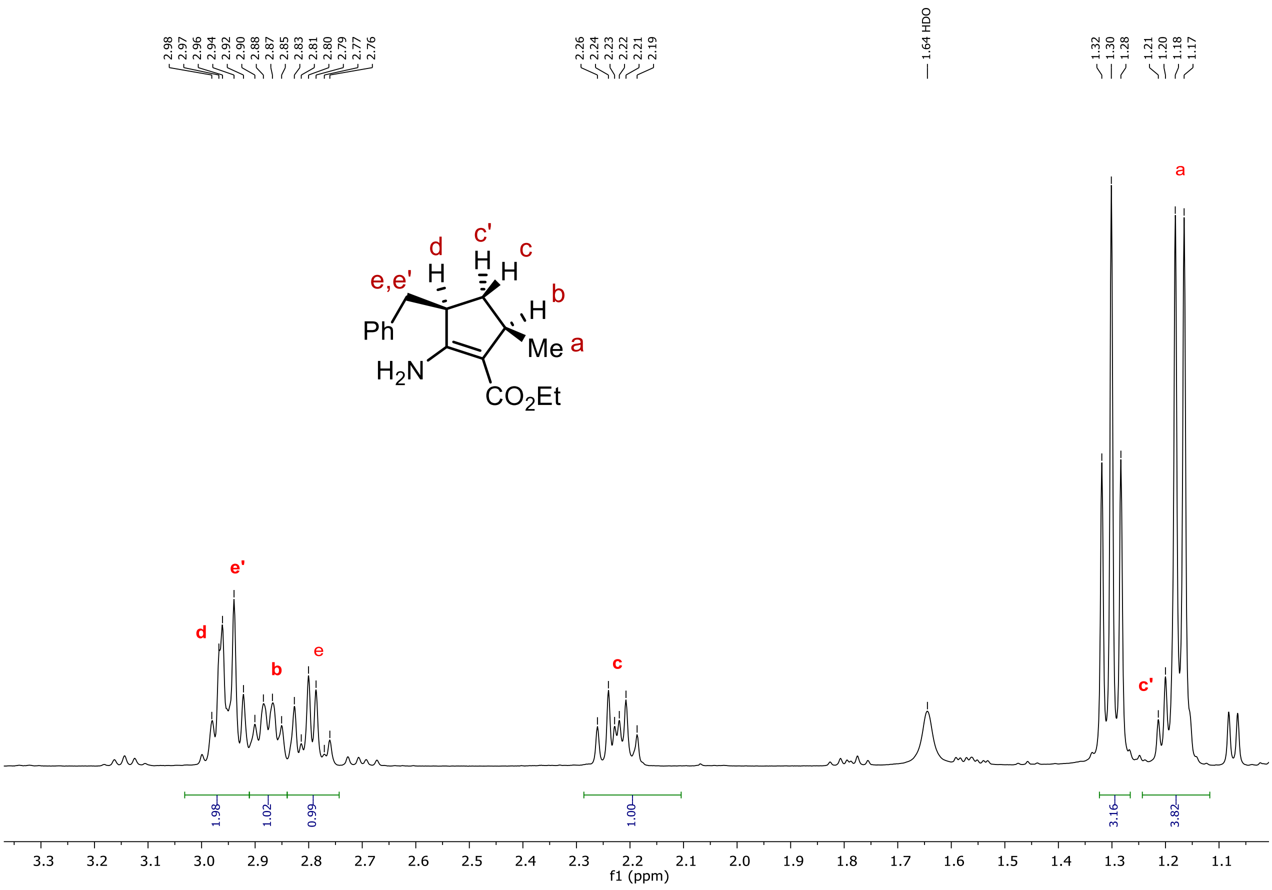

NOESY (400 MHz, Chloroform-*d*)

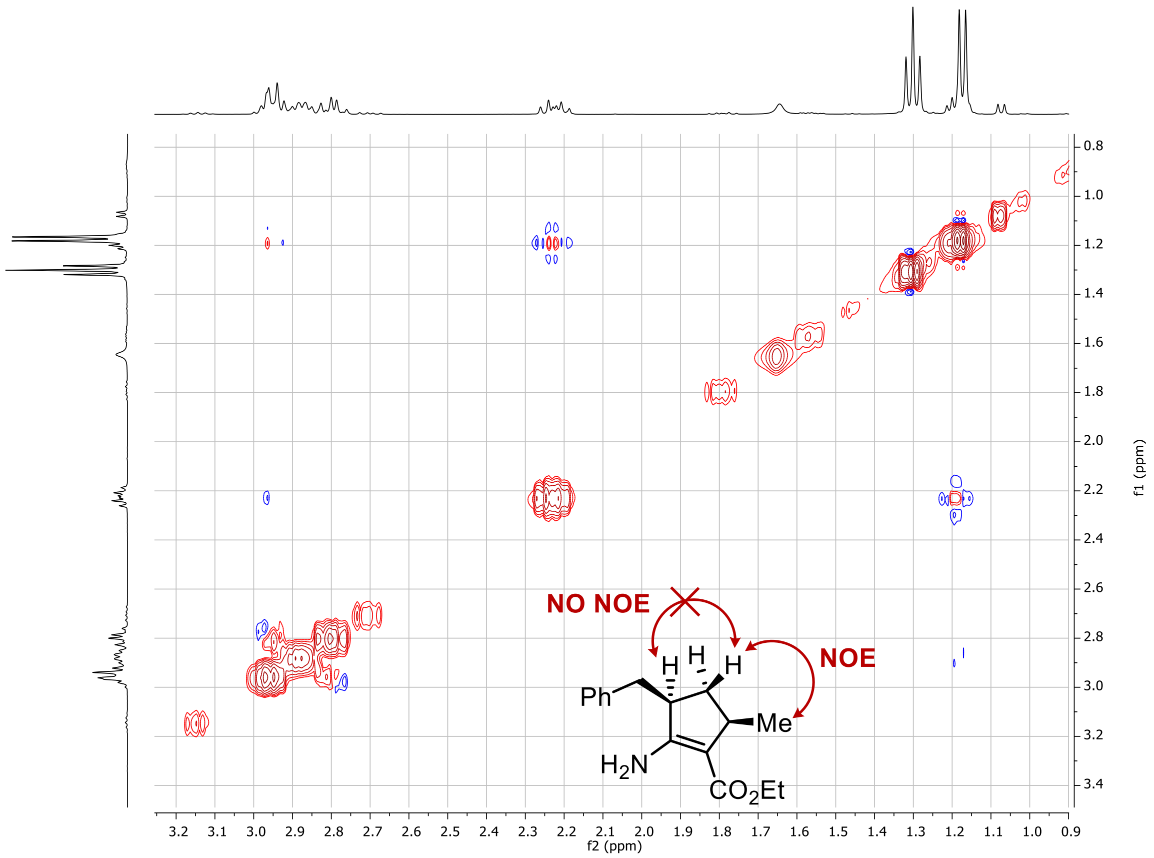

Ethyl (3S,4R,5S)-2-amino-3-benzyl-3-chloro-5-methyl-4-((4-methylphenyl)sulfonamido) cyclopent-1-ene-1-carboxylate (13)

^{1}H NMR

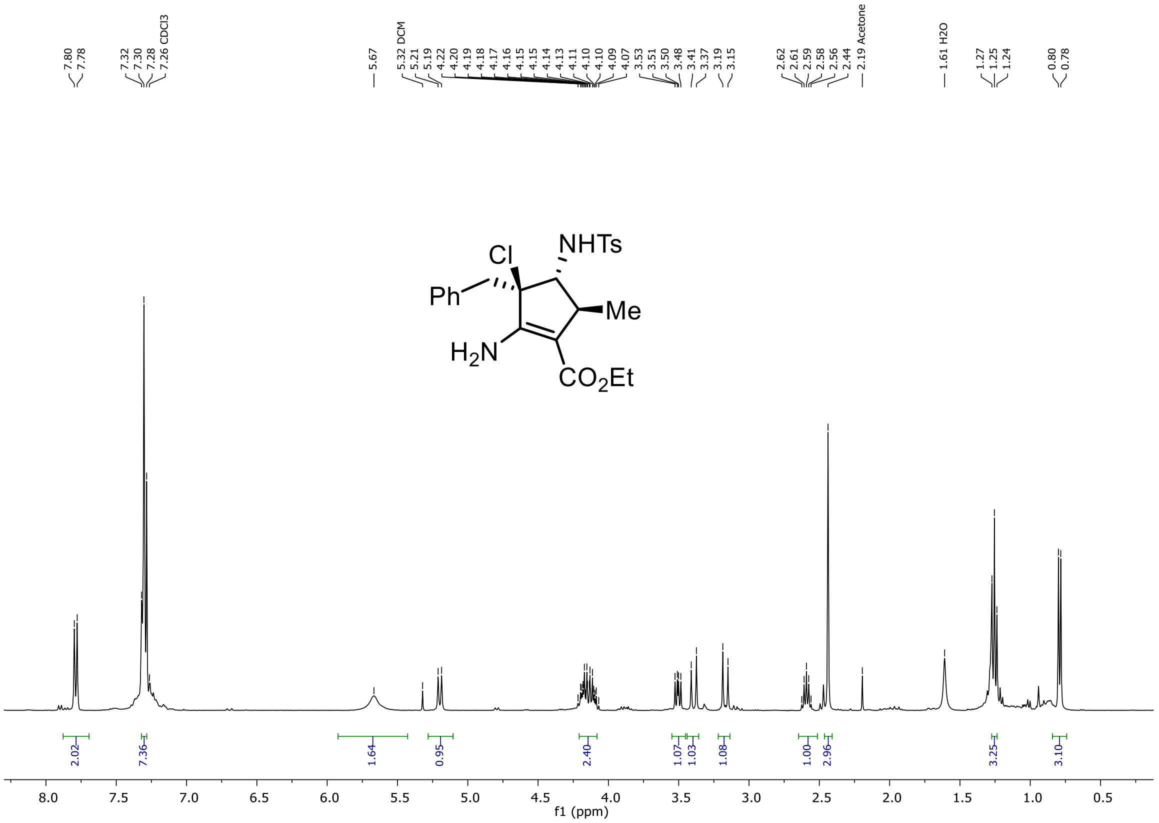

^{13}C NMR

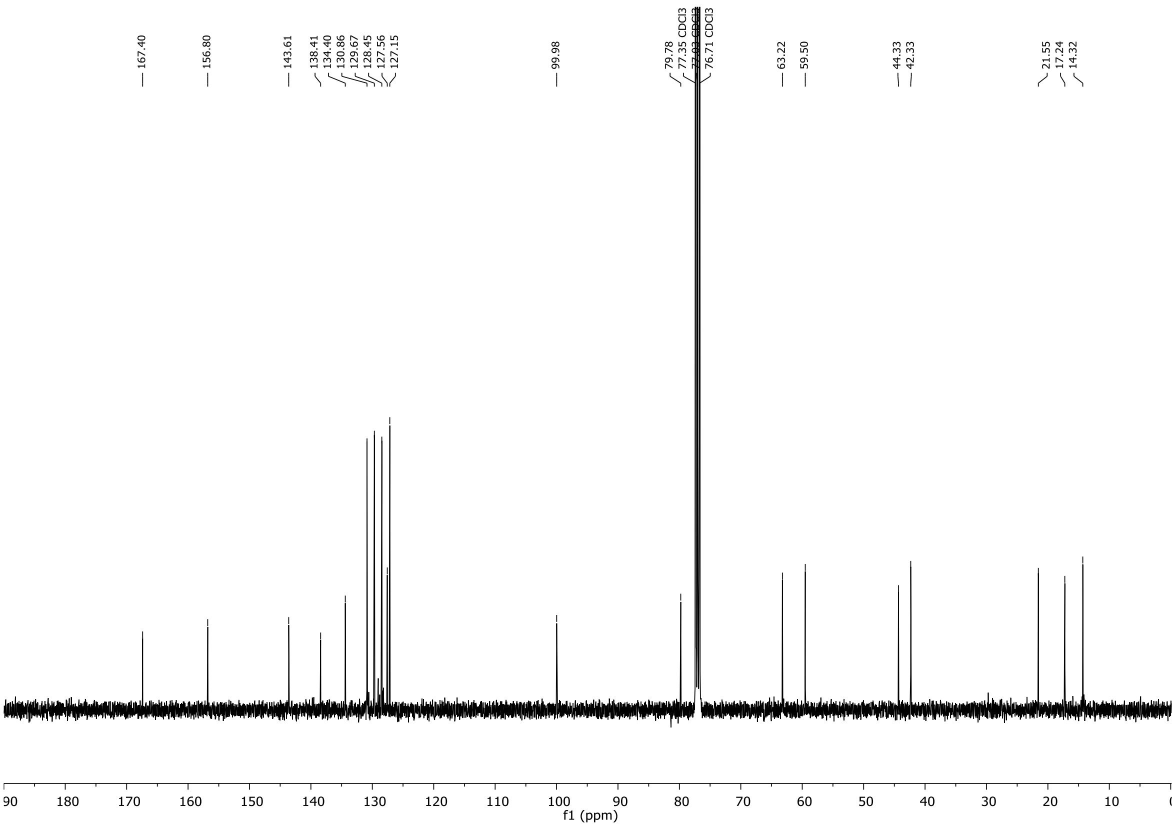

COSY (400 MHz, Chloroform-*d*)

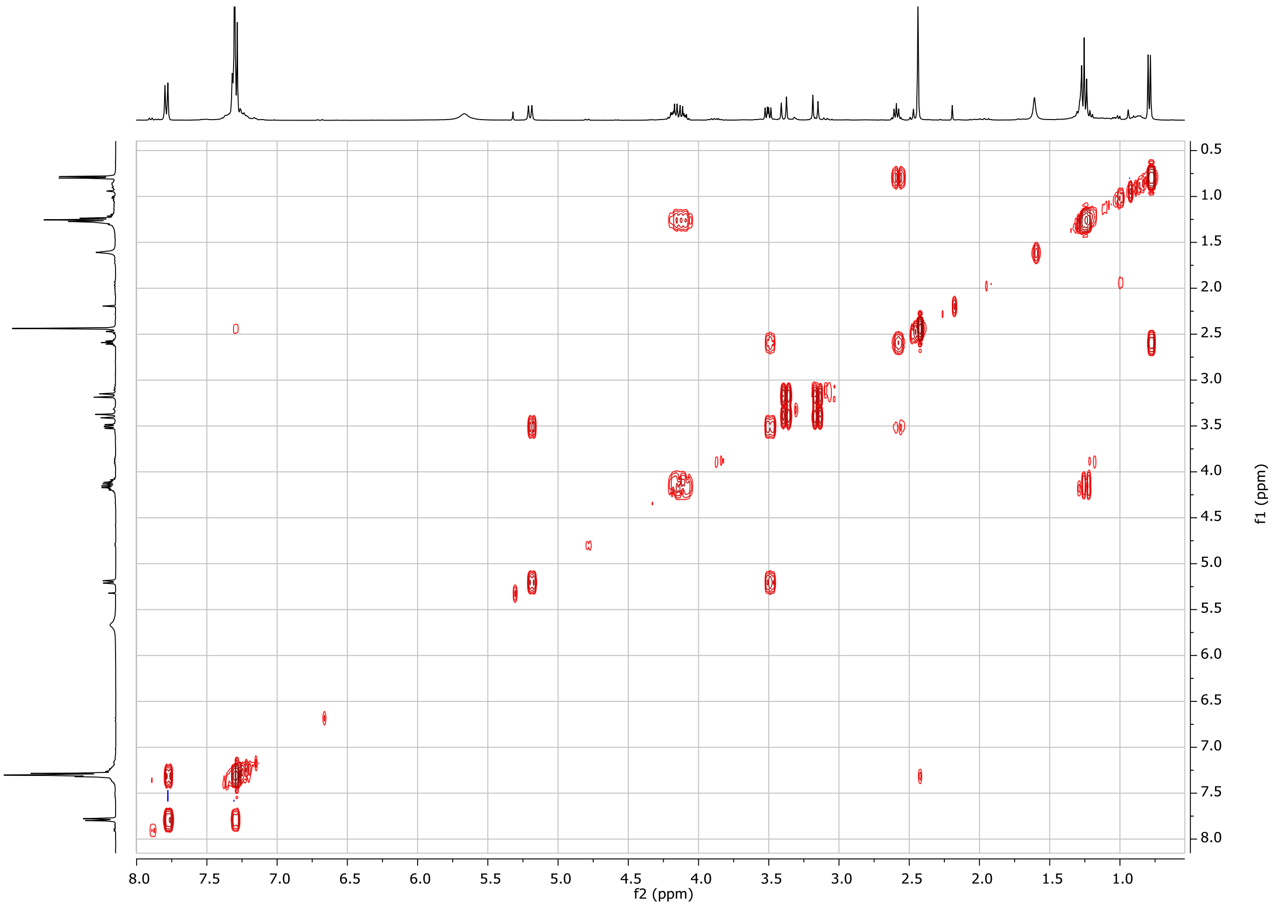

HSQC (400 MHz, Chloroform-*d*)

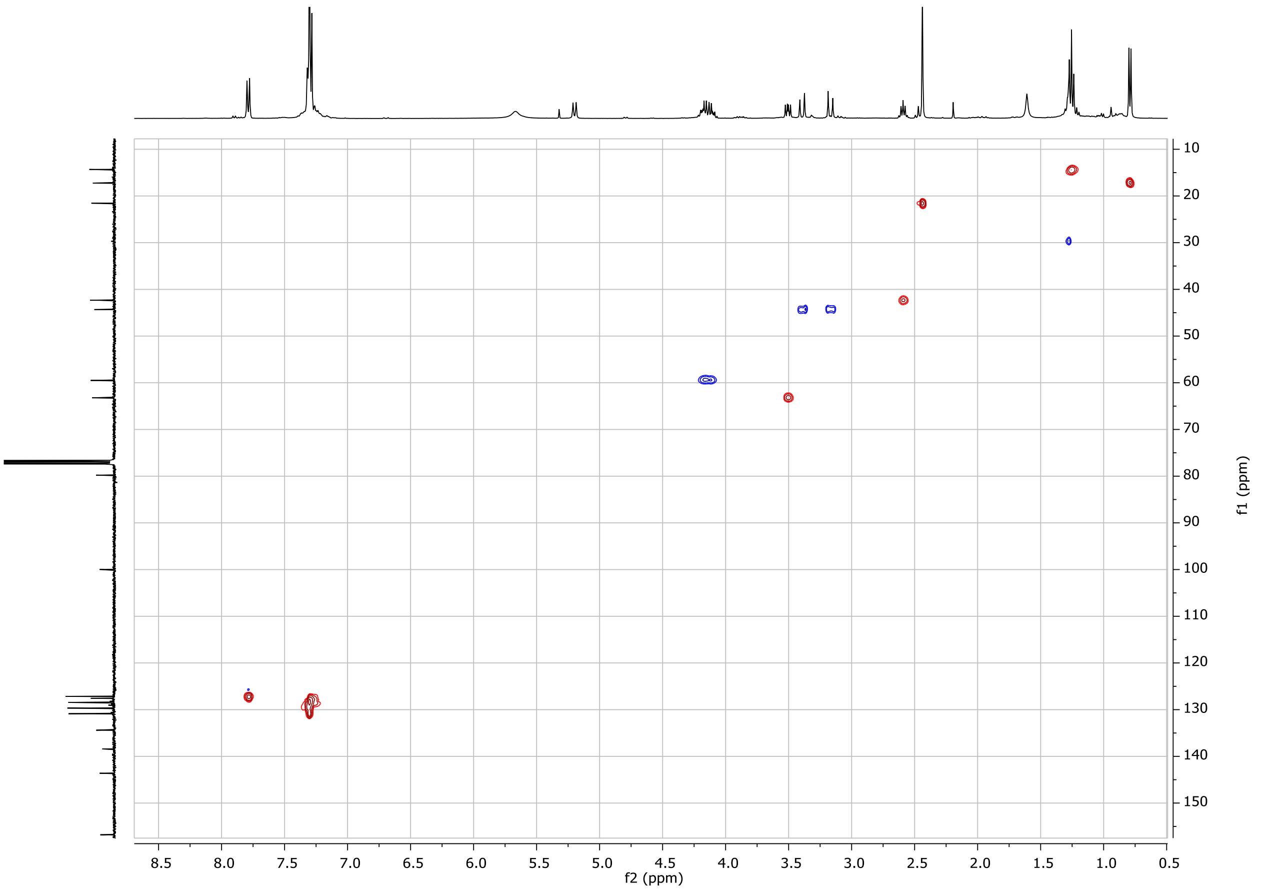

HMBC (400 MHz, Chloroform-*d*)

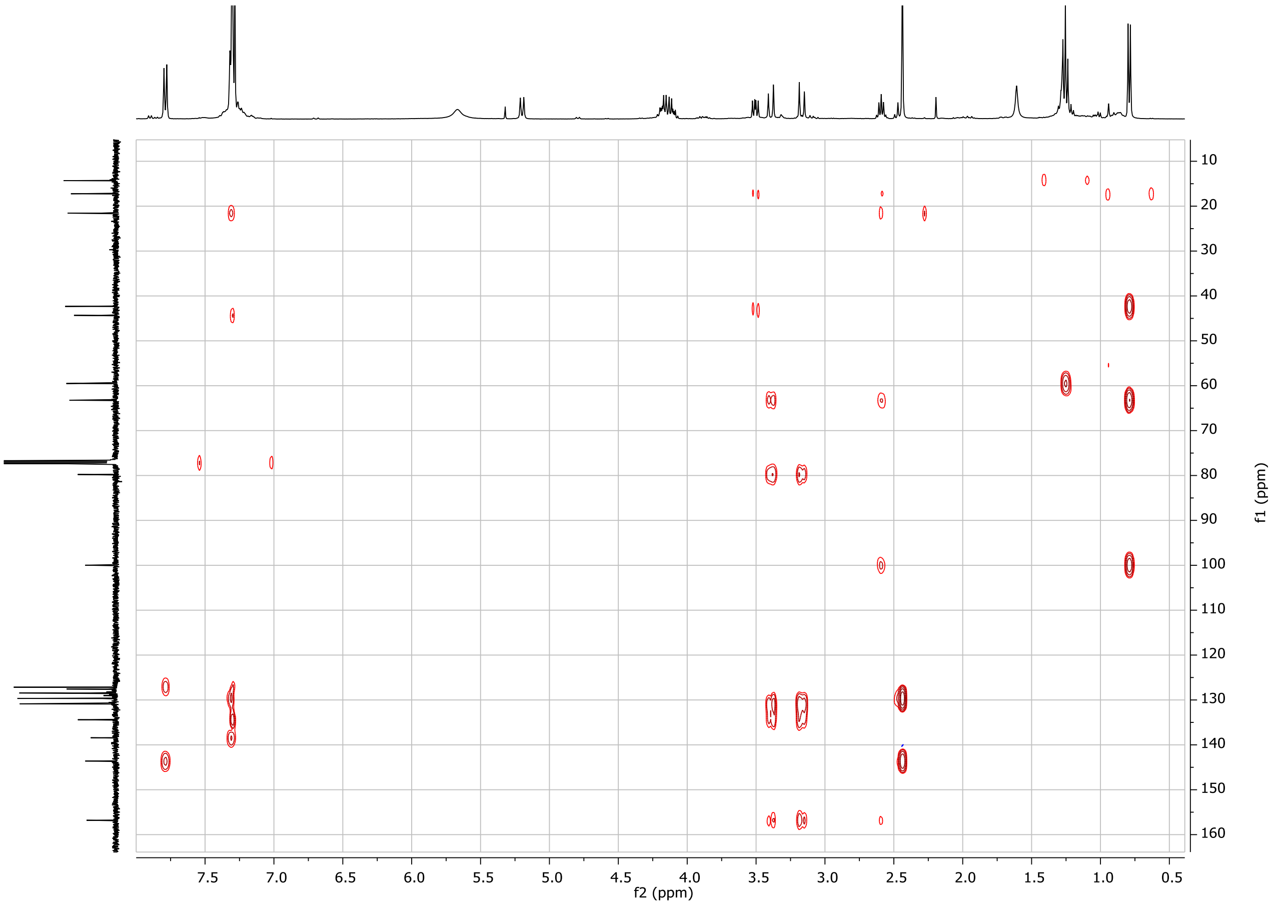

NOESY (600 MHz, Chloroform-*d*)

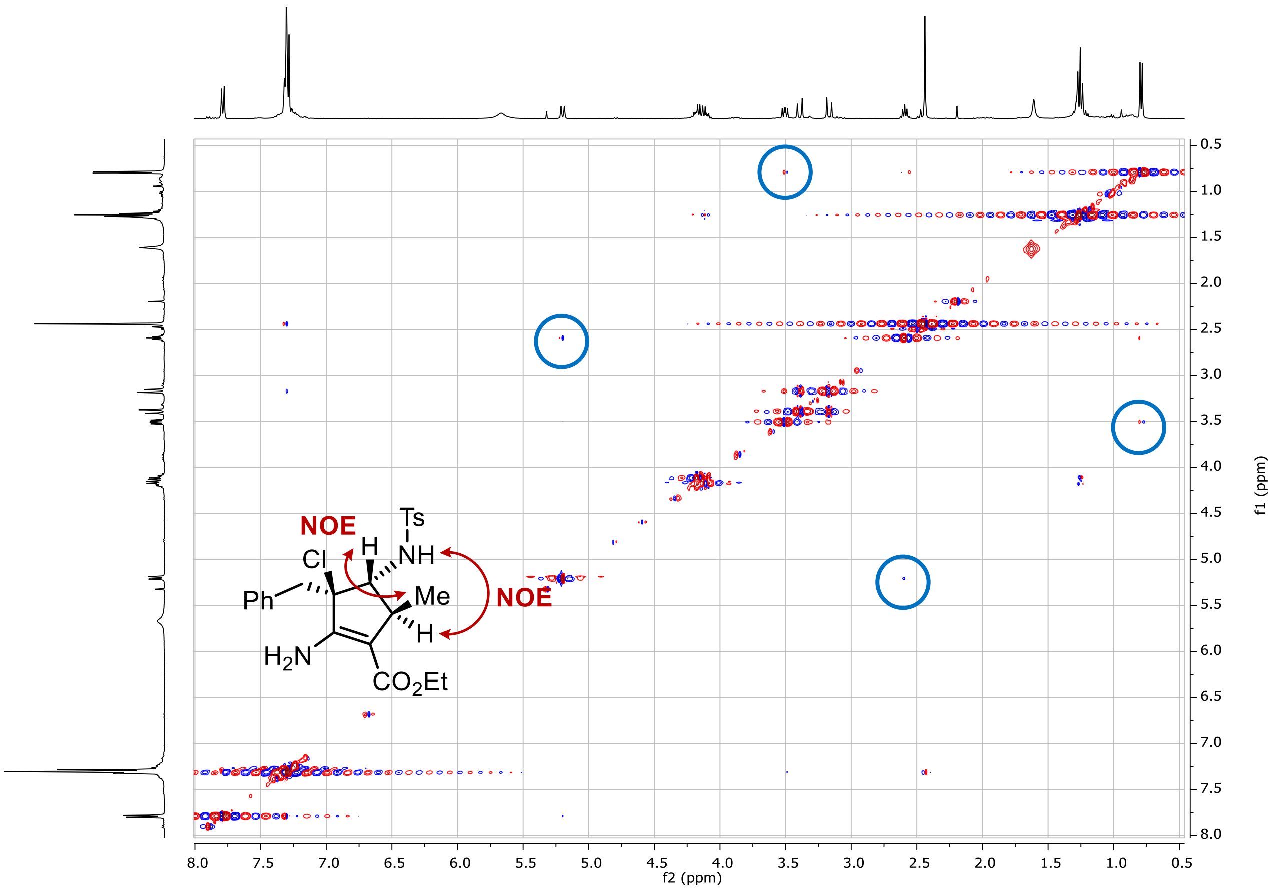

Rhodium Cyclopentadienyl complex Rh(COD)($C_{16}H_{18}NO_2$) (14)

^{1}H NMR

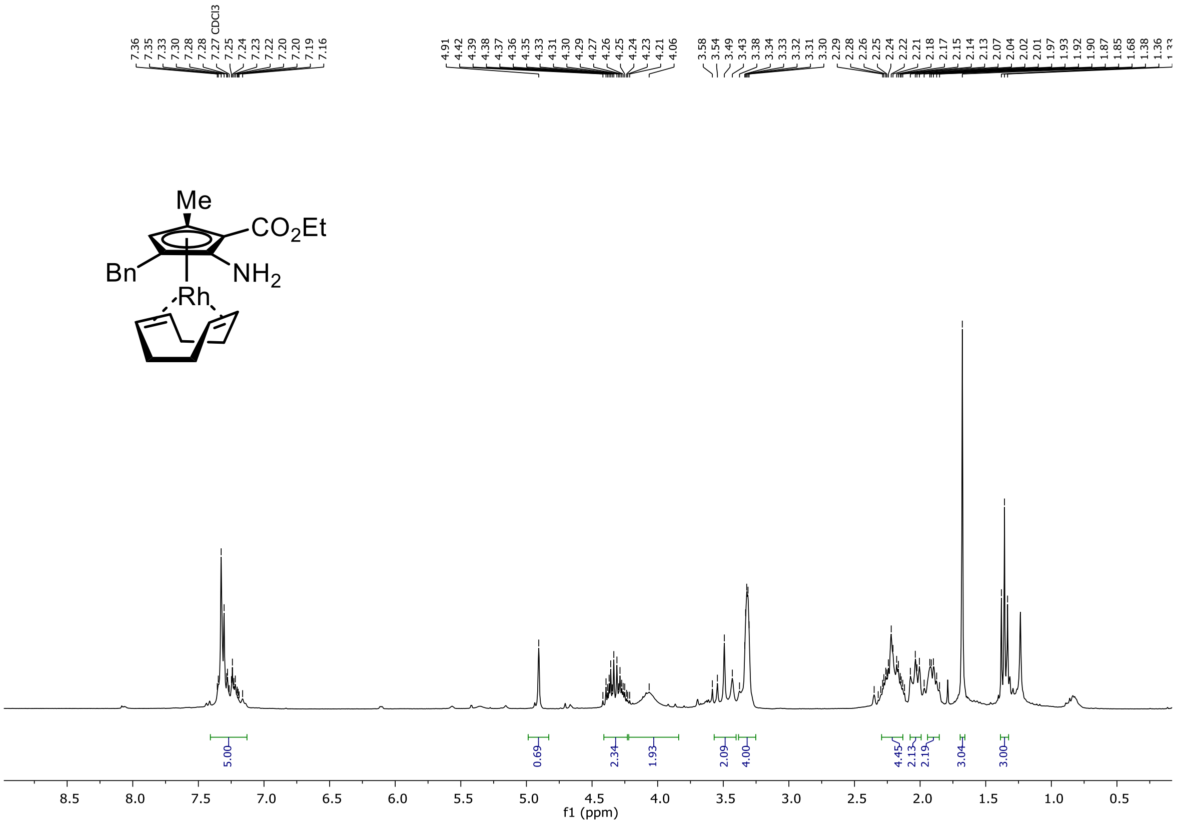

^{13}C NMR

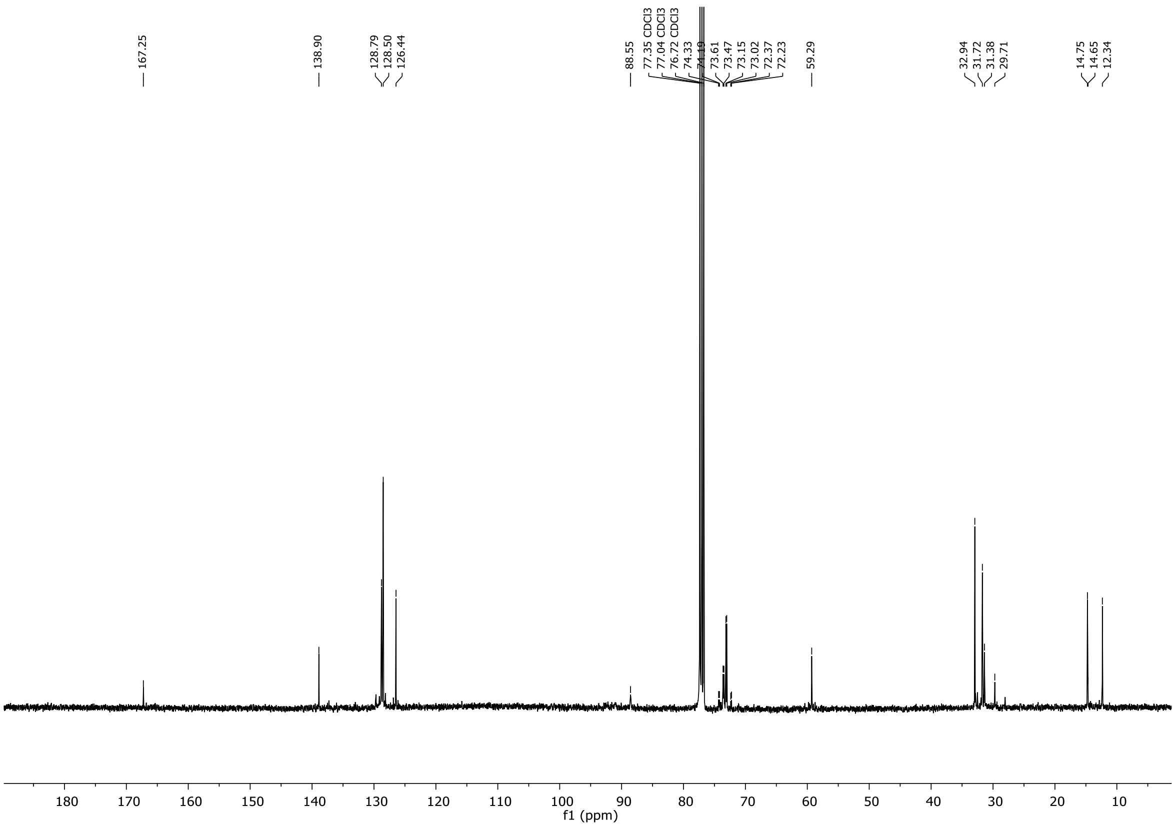

Ethyl 2-amino-3-benzyl-5-methylcyclopenta-1,3-diene-1-carboxylate-5-d (3a-d)

^{1}H NMR

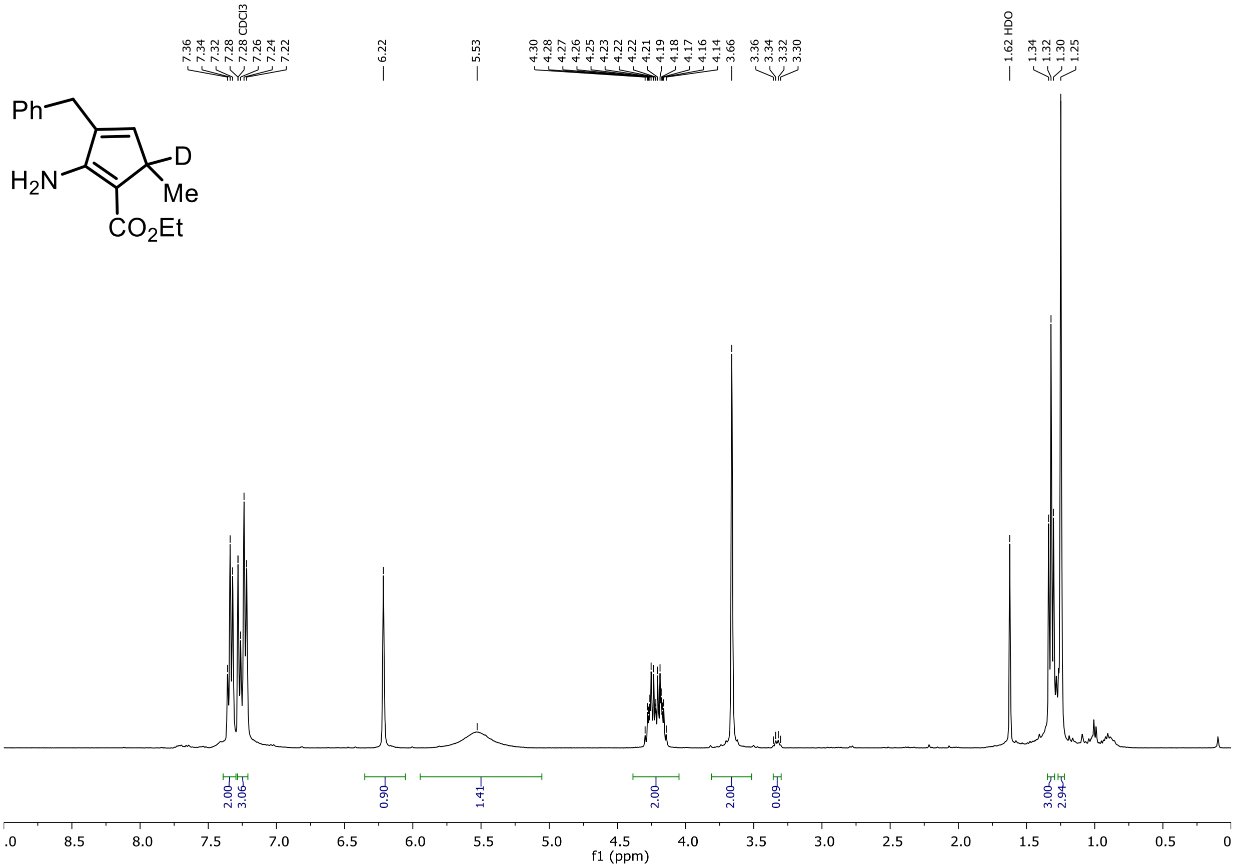

^{13}C NMR

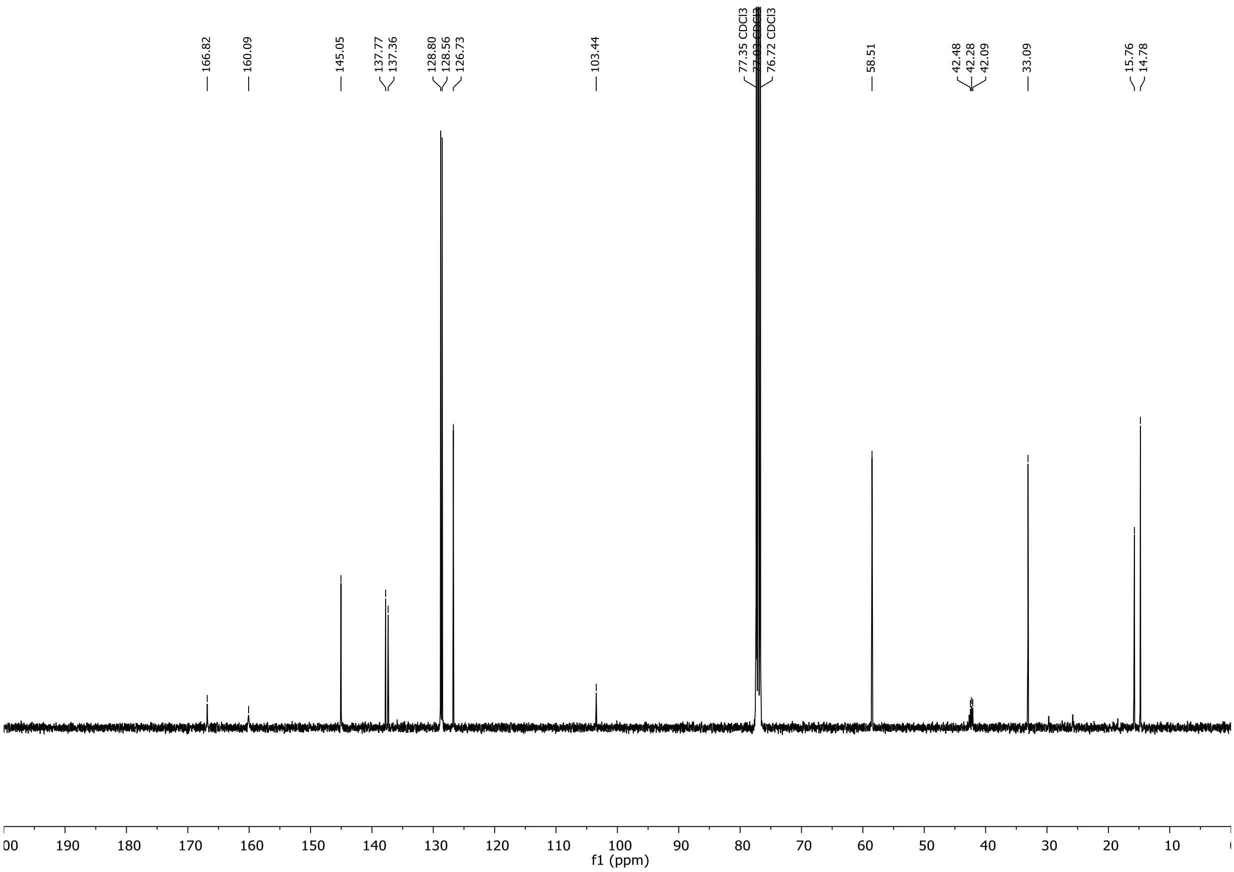

166.82
160.09
145.05
137.77
137.36
128.80
128.56
126.73
103.44
77.35 CDCl3
76.72 CDCl3
58.51
42.48
42.28
42.09
33.09
15.76
14.78
190
180
170
160
150
140
130
120
110
100
90
80
70
60
50
40
30
20
10
f1 (ppm)

11 References

[39] LaLonde, R. T.; Perakyla, H.; Hayes, M. P. "Potentially Mutagenic, Chorine-Substituted 2(5H)-Furanones: Studies of Their Synthesis and NMR Properties" *J. Org. Chem.* **1990**, *55* (9), 2847–2855.

[40] Soppelsa, P.; Vaghi, F.; Licini, G.; Orlandi, M. "Cu-Catalyzed Asymmetric Olefinative Conjugate Addition of Dialkylzinc Reagents" *Org. Lett.* **2025**, *27* (27), 7278–7283.

[41] Ye, Y. S.; Laverny, A.; Wodrich, M. D.; Laplaza, R.; Fadaei-Tirani, F.; Scopelliti, R.; Corminboeuf, C.; Cramer, N. "Enantiospecific Synthesis of Planar Chiral Rhodium and Iridium Cyclopentadienyl Complexes: Enabling Streamlined and Computer-Guided Access to Highly Selective Catalysts for Asymmetric C–H Functionalizations" *J. Am. Chem. Soc.* **2024**, *146* (50), 34786–34795.

[42] Bruker, *SAINT*, *V8.41*, Bruker AXS Inc., Madison, Wisconsin, USA.

[43] Krause, L.; Herbst-Irmer, R.; Sheldrick, G. M.; Stalke, D. "Comparison of Silver and Molybdenum Microfocus X-Ray Sources for Single-Crystal Structure Determination" *J. Appl. Cryst.* **2015**, *48* (1), 3–10.

[44] Sheldrick, G. M. "SHELXT – Integrated Space-Group and Crystal-Structure Determination" *Acta Cryst A* **2015**, *71* (1), 3–8.

[45] Sheldrick, G. M. "Crystal Structure Refinement with SHELXL" *Acta Cryst C* **2015**, *71* (1), 3–8.

[46] Groom, C. R.; Bruno, I. J.; Lightfoot, M. P.; Ward, S. C. "The Cambridge Structural Database" *Acta Cryst B* **2016**, *72* (2), 171–179.

[47] Zhou, R.; Li, J.; Cheo, H. W.; Chua, R.; Zhan, G.; Hou, Z.; Wu, J. "Visible-Light-Mediated Deuteration of Silanes with Deuterium Oxide" *Chem. Sci.* **2019**, *10* (31), 7340–7344.

[48] Gaussian 16, Revision C.01, M. J. Frisch, G. W. Trucks, H. B. Schlegel, G. E. Scuseria, M. A. Robb, J. R. Cheeseman, G. Scalmani, V. Barone, G. A. Petersson, H. Nakatsuji, X. Li, M. Caricato, A. V. Marenich, J. Bloino, B. G. Janesko, R. Gomperts, B. Mennucci, H. P. Hratchian, J. V. Ortiz, A. F. Izmaylov, J. L. Sonnenberg, D. Williams-Young, F. Ding, F. Lipparini, F. Egidi, J. Goings, B. Peng, A. Petrone, T. Henderson, D. Ranasinghe, V. G. Zakrzewski, J. Gao, N. Rega, G. Zheng, W. Liang, M. Hada, M. Ehara, K. Toyota, R. Fukuda, J. Hasegawa, M. Ishida, T. Nakajima, Y. Honda, O. Kitao, H. Nakai, T. Vreven, K. Throssell, J. A. Montgomery, Jr., J. E. Peralta, F. Ogliaro, M. J. Bearpark, J. J. Heyd, E. N. Brothers, K. N. Kudin, V. N. Staroverov, T. A. Keith, R. Kobayashi, J. Normand, K. Raghavachari, A. P. Rendell, J. C. Burant, S. S. Iyengar, J. Tomasi, M. Cossi, J. M. Millam, M. Klene, C. Adamo, R. Cammi, J. W. Ochterski, R. L. Martin, K. Morokuma, O. Farkas, J. B. Foresman, and D. J. Fox, Gaussian, Inc., Wallingford CT, **2016**.

[49] CYLview20; Legault, C. Y., Université de Sherbrooke, **2020** (http://www.cylview.org).

[50] Lonardi, G.; Franco, S.; Sartorello, M.; De Faveri, C.; Stivanello, M.; Licini, G.; Orlandi, M. "Enantioselective Synthesis of Cyclopropanes via CuH-Catalyzed Intramolecular Hydroalkylation" *ACS Catal.* **2024**, *14* (11), 8730–8738.

[51] Cramer, C. J.; Truhlar, D. G. "Density Functional Theory for Transition Metals and Transition Metal Chemistry" *Phys. Chem. Chem. Phys.* **2009**, *11* (46), 10757.

[52] Chai, J.-D.; Head-Gordon, M. “Long-Range Corrected Hybrid Density Functionals with Damped Atom–Atom Dispersion Corrections” *Phys. Chem. Chem. Phys.* **2008**, *10* (44), 6615.

[53] Yang, C.; Xue, X.-S.; Jin, J.-L.; Li, X.; Cheng, J.-P. “Theoretical Study on the Acidities of Chiral Phosphoric Acids in Dimethyl Sulfoxide: Hints for Organocatalysis” *J. Org. Chem.* **2013**, *78* (14), 7076–7085.

[54] Schmidt, R. K.; Müther, K.; Mück-Lichtenfeld, C.; Grimme, S.; Oestreich, M. “Silylium Ion-Catalyzed Challenging Diels–Alder Reactions: The Danger of Hidden Proton Catalysis with Strong Lewis Acids” *J. Am. Chem. Soc.* **2012**, *134* (9), 4421–4428.
